# DNA methylation atlas and machinery in the developing and regenerating annelid *Platynereis dumerilii*

**DOI:** 10.1186/s12915-021-01074-5

**Published:** 2021-08-03

**Authors:** Anabelle Planques, Pierre Kerner, Laure Ferry, Christoph Grunau, Eve Gazave, Michel Vervoort

**Affiliations:** 1 Université de Paris, CNRS, Institut Jacques Monod, F-75006 Paris, France; 2 Université de Paris, CNRS, Epigenetics and Cell Fate, F-75006 Paris, France; 3 IHPE, Univ Montpellier, CNRS, IFREMER, Univ Perpignan Via Domitia, F-66860 Perpignan, France

**Keywords:** Epigenetics, DNA methylation, 5mC, Regeneration, Development, Annelids, Evolution

## Abstract

**Background:**

Methylation of cytosines in DNA (5mC methylation) is a major epigenetic modification that modulates gene expression and constitutes the basis for mechanisms regulating multiple aspects of embryonic development and cell reprogramming in vertebrates. In mammals, 5mC methylation of promoter regions is linked to transcriptional repression. Transcription regulation by 5mC methylation notably involves the nucleosome remodeling and deacetylase complex (NuRD complex) which bridges DNA methylation and histone modifications. However, less is known about regulatory mechanisms involving 5mC methylation and their function in non-vertebrate animals. In this paper, we study 5mC methylation in the marine annelid worm *Platynereis dumerilii*, an emerging evolutionary and developmental biology model capable of regenerating the posterior part of its body post-amputation.

**Results:**

Using in silico and experimental approaches, we show that *P. dumerilii* displays a high level of DNA methylation comparable to that of mammalian somatic cells. 5mC methylation in *P. dumerilii* is dynamic along the life cycle of the animal and markedly decreases at the transition between larval to post-larval stages. We identify a full repertoire of mainly single-copy genes encoding the machinery associated with 5mC methylation or members of the NuRD complex in *P. dumerilii* and show that this repertoire is close to the one inferred for the last common ancestor of bilaterians. These genes are dynamically expressed during *P. dumerilii* development and regeneration. Treatment with the DNA hypomethylating agent Decitabine impairs *P. dumerilii* larval development and regeneration and has long-term effects on post-regenerative growth.

**Conclusions:**

Our data reveal high levels of 5mC methylation in the annelid *P. dumerilii*, highlighting that this feature is not specific to vertebrates in the bilaterian clade. Analysis of DNA methylation levels and machinery gene expression during development and regeneration, as well as the use of a chemical inhibitor of DNA methylation, suggest an involvement of 5mC methylation in *P. dumerilii* development and regeneration. We also present data indicating that *P. dumerilii* constitutes a promising model to study biological roles and mechanisms of DNA methylation in non-vertebrate bilaterians and to provide new knowledge about evolution of the functions of this key epigenetic modification in bilaterian animals.

**Supplementary Information:**

The online version contains supplementary material available at 10.1186/s12915-021-01074-5.

## Background

Epigenetic modifications or marks refer to any transient chemical alterations of nucleic acids or histones, which do not modify the primary nucleic acid sequence and which can be transmitted from one generation of cells (and in some cases of organisms) to the next [1, 2]. Epigenetic marks regulate gene expression and therefore are of paramount importance to most aspects of the biology of living organisms, including during development, regeneration, and stem cell maintenance in animals [3, 4].

DNA methylation is an important epigenetic modification, found in the three domains of life, and has been the subject of intense study for many years [5–9]. In animals, DNA methylation mainly occurs through the covalent addition of a methyl group on position 5 of a cytosine to form 5-methyl-cytosine (5mC). 5mC are mostly (or even exclusively in some species) found in cytosine-guanine dinucleotides, known as CpG sequences [10]. Abundance and distribution of 5mC strongly vary in different animal lineages. For example, in mammals, about 70–80% of CpGs throughout the genome are methylated in somatic tissue types. Unmethylated regions are largely restricted to dense clusters of CpGs, known as CpG islands (CGIs), which account for roughly two-thirds of mammalian gene promoters. In the rare cases where CGI promoters are highly methylated, genes are stably transcriptionally repressed. In many non-vertebrates, methylated CpGs are mostly found within gene bodies (transcribed regions) [11, 12]. The function(s) of this form of DNA methylation, which is referred to as “gene body methylation” (and which is also found in vertebrates), is still largely unknown, but it has been hypothesized that it could be involved in homeostatic regulation of gene transcription [13]. 5mC are also often found in repetitive sequences and have been shown to be important for repressing the activity of transposable elements [7, 9].

DNA 5mC presence and roles rely on the activity of several classes of proteins that can be functionally classified according to their role: methylases that promote addition of methyl groups (“Writers”); proteins that oxidize 5mC and stimulate demethylation (“Modifiers”); and proteins that bind to methylated nucleotides, allowing interpretation of the encoded information, for example in terms of gene expression (“Readers”) [5, 14] (Additional file 1: Fig. S1). Deposition of 5mC marks on DNA requires the action of evolutionarily conserved DNA methyltransferases (Dnmts) [15]. In mammals, three families of Dnmts are found (Dnmt1, 2, and 3), each of which play specific roles [5, 7]. Dnmt3 proteins are involved in the de novo addition of 5mC, while another member of the family, Dnmt1, maintains the methylation pattern during replication. Dnmt1 function involves Uhrf1 (Ubiquitin Like with PHD And Ring Finger Domains 1 protein) which binds to both hemi-methylated DNA and Dnmt1, thereby recruiting Dnmt1 to methylated DNA sites [16]. Dnmt2 (also referred to as tRNA aspartic acid methyltransferase 1) is a tRNA-methylating enzyme seemingly not involved in DNA methylation, at least in mammals [17]. DNA demethylation, which results in the recovery of non-methylated cytosines, occurs either passively during cell division and DNA replication (in the absence of Dnmt1 function) or actively thanks to the Ten-Eleven Translocation (Tet) family enzymes and G/T Mismatch-Specific Thymine DNA Glycosylase (Tdg) proteins [18, 19].

A series of proteins, known as methyl-CpG binding proteins (Mbp), recognize and bind methylated CpGs, acting as readout of DNA methylation by recruiting chromatin remodelers [20, 21]. One key family of Mbp are methyl-CpG-binding domain (Mbd) proteins, which are found in many animals [22]. In mammals, seven members of this family are found, and they mostly promote transcriptional silencing by interacting with a wide array of histone methylases and deacetylases [20, 21]. Mbd2 and Mbd3 are part of the nucleosome remodeling and deacetylase complex (NuRD complex), which bridges DNA methylation and histone modifications and has been shown to regulate gene expression [23–25] (Additional file 1: Fig. S1). In addition to Mbd2 or Mbd3, the NuRD complex is also composed of several other proteins: chromodomain helicase DNA-binding proteins (Chd 3, 4, and 5) which remodel chromatin; class I histone deacetylases (Hdac 1, 2, 3, and 8) that deacetylate histone tails and are associated with chromatin compaction and gene silencing; retinoblastoma-binding protein (Rbbp4/7; also known as RbAp46/48) which is a histone chaperone; GATA-binding protein (Gata2a/b); and metastasis-associated proteins (Mta1/2/3). While Mbd2/3 and Gata2a/b are exclusively found in the NuRD complex, the other proteins can also belong to other proteins complexes and therefore have NuRD-independent functions.

Most of what we know about DNA methylation in metazoans comes from studies conducted in a few model organisms, mainly mammals. While a handful of studies of DNA methylation in non-model organisms from various animal clades have been published recently (e.g., [26–33]), little is known about the importance and roles of 5mC modifications in non-vertebrate species. We therefore decided to study DNA methylation in an emerging developmental biology model system, the marine annelid worm *Platynereis dumerilii*. Annelids belong, together with phyla such as mollusks and platyhelminthes, to lophotrochozoans, one of the three branches of bilaterian animals, distinct from those to which vertebrates (deuterostomes), and arthropods and nematodes (ecdysozoans) belong [34]. *P. dumerilii* has a complex life cycle composed of several phases [35] starting by a three-day-long embryonic development that gives rise to small larvae with three segments bearing appendages (parapodia). These larvae then metamorphose into small juvenile worms that enter a long phase of juvenile growth during which they add additional segments one by one in the posterior part of their body (a process known as posterior growth) [36]. Posterior growth relies on the presence of a subterminal posterior growth zone that contains putative stem cells, the sustained proliferation of which allows the formation of segments over many months [36]. During this phase of juvenile growth, *P. dumerilii* worms also display substantial regenerative abilities. In particular, after posterior amputation (removal of several segments, the posterior growth zone, and the terminal body part containing the anus called the pygidium), *P. dumerilii* worms are able to regenerate the differentiated structures of the pygidium and the stem cells of the growth zone whose activity subsequently allows for the reformation of the amputated segments [37]. This process, called posterior regeneration, involves the formation of a regeneration blastema whose cells likely derive from the dedifferentiation of cells belonging to tissues abutting the amputation plane. Indeed, at 1 and 2 days post-amputation, cells at the amputation site start to express various proliferation and pluripotency stem cell markers [37], suggesting that amputation induces extensive reprogramming of differentiated cells into proliferating progenitor/stem cells.

Given the well-known importance of epigenetic modifications such as DNA methylation during cellular reprogramming events and evidence for their involvement during vertebrate regeneration (e.g., [3, 38–40]), we hypothesized that epigenetic modifications might be important during *P. dumerilii* posterior regeneration and that this process might represent a valuable model to understand how epigenetic regulation influences cellular reprogramming and regeneration. In this study, using in silico and experimental approaches, we found high levels of CpG methylation in the *P. dumerilii* genome, with significant variations during development. Using genomic data and phylogenetic analyses, we identified a full set of *P. dumerilii* writers, modifiers, and readers of 5mC methylation, as well as NuRD components. We subsequently studied the evolution of these proteins in animals. We also found that many of the corresponding genes have dynamic expression during development and regeneration. Strikingly, most investigated genes have expression patterns during regeneration similar to those previously documented for stem cell genes [37]. Treatments with a DNA hypomethylating drug, Decitabine (5-aza-2′-deoxycytidine), impaired larval development, regeneration, and subsequent segment addition, suggesting a requirement of DNA methylation for posterior regeneration and post-regenerative posterior growth in *P. dumerilii*.

## Results

### High level of CpG methylation in *P. dumerilii*

**Fig. 1:**
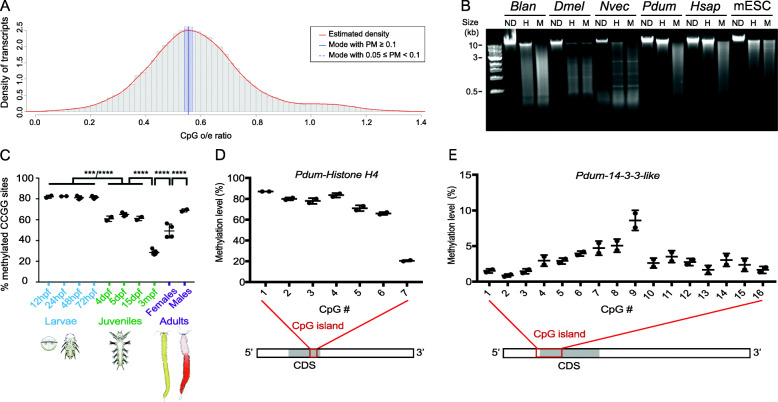
High-level and gene body CpG methylation in *P. dumerilii*. **a** Histogram of CpG o/e ratios of *P. dumerilii* transcripts. The red line indicates the estimated density, the vertical blue bar shows estimated mean value, and the shaded blue bar represents bootstrap confidence intervals of 95%. PM = probability mass. **b** Electrophoresis of non-digested (ND) genomic DNA (gDNA) or digested with *HpaII* (H) or *MspI* (M) from six different animal species with different methylation types. Sizes of fragments, in kilobase pairs (kb), are indicated to the left. Abbreviations: *Blan* = *Branchiostoma lanceolatum*; *Dmel = Drosophila melanogaster*; *Nvec* = *Nematostella vectensis*; *Pdum* = *Platynereis dumerilii*; *Hsap* = *Homo sapiens*; mESC = *Mus musculus naïve embryonic stem cells*. **c** Graphic representation of DNA methylation measured by LUMA at ten different stages of *P. dumerilii* life cycle (four larval stages, four juvenile stages, and adults (male and females); at least two biological replicates per stage and at least two technical replicates per biological replicate). Mean ± SD. One-way ANOVA, Tukey post hoc test (****: p < 0.0001, ***: p < 0.001). The raw data and results of all statistical tests can be found in Additional file 2: Table S2. hpf = hours post-fertilization; dpf = days post-fertilization; mpf = months post-fertilization. Drawings of larvae, juveniles, and adult worms are adapted from [35]. **d, f** Graphic representation of methylation levels of stretches of CpGs (CpG island) in two *P. dumerilii* genes, *Pdum-Histone H4* (**d**) and *Pdum-14-3-3-like* (**e**), as defined by bisulfite pyrosequencing on DNA extracted from 72hpf larvae (two biological replicates and two technical replicates per biological replicate). Mean ± SD of two biological replicates is shown. A schematic representation of the localization of the studied CpG islands in the transcribed region of the two genes is also shown. CDS = coding sequence. Data shown in the graph and methylation levels at other developmental stages can be found in Additional file 2: Table S3

In a first attempt to characterize DNA methylation in *P. dumerilii*, we used a computational approach that allows to evaluate the DNA methylation level and pattern of an organism based on the determination of normalized CpG content (e.g., [41–43]). Indeed, methylated CpGs are hypermutable compared to the other dinucleotides [44]. While deamination of non-methylated cytosine can be efficiently repaired, 5mC deamination gives rise to thymines which are less efficiently processed by DNA repair mechanisms [44, 45]. As a consequence, the mutation rate of 5mC into T is much higher than other transitions [46]. In species with high levels of 5mC in CpGs, there is an increase of the mutation rate from CpG to TpG or CpA, which leads, over several generations of germline mutation accumulation, to low contents of CpGs in the genomes of these species [47]. Determining the CpG observed/expected (o/e) ratios can thus be used to estimate 5mC levels: CpG o/e close to 1 means no methylation while CpG o/e far below 1 suggests that methylation of CpGs is present (e.g., [41–43]). As in non-vertebrates methylated CpGs are mostly found within gene bodies [11, 12], we calculated CpG o/e for *P. dumerilii* gene bodies, by applying Notos, a software that computes CpG o/e ratios based on kernel density estimations [43, 48], on a high-quality *P. dumerilii* reference transcriptome [49]. We found a CpG o/e distribution with a single mode at 0.55 (Fig. 1a), suggesting high-level gene body methylation in *P. dumerilii*. Indeed, based on a large-scale analysis of 147 species from all major eukaryote lineages, four types of gene body methylation have been defined and *P. dumerilii* fits into type 3 to which belong species with high gene body methylation, which is the case for most vertebrates [48]. We used the same approach to calculate CpG o/e for additional species used for phylogenetic analyses of methylation machinery proteins (Additional file 2: Table S1; Additional file 3: Fig. S2)—see below for further discussion.

To further assess CpG methylation in the *P. dumerilii* genome at the experimental level, we performed genomic DNA (gDNA) digestion with the methylation-sensitive enzyme HpaII and its methylation-insensitive isoschizomer MspI, which target CCGG sites [50]. If portions of genomes are methylated, different profiles of restriction fragments are expected from the two enzymatic digestions. To facilitate interpretation of profiles obtained with *P. dumerilii* gDNA, we included gDNA from species with known methylation patterns in our experiment (Fig. 1b). *Drosophila melanogaster* do not have 5mC methylation and, as previously reported [51], similar profiles are obtained for both *HpaII* and *MspI* enzymatic digestions. The cephalochordate *Branchiostoma lanceolatum* and the cnidarian *Nematostella vectensis* have a mosaic pattern of methylation (type 4 in [48]), characterized by the presence of a large number of different cleaved fragments in both digestions and a high molecular weight fraction only found with *HpaII* [50]. Vertebrates such as *Homo sapiens* have global CpG methylation in their genome (type 3 in [48]), and accordingly their gDNA is largely resistant to *HpaII* digestion [50]. An exception are naïve mouse embryonic stem cells (mESC) [52] where similar restriction profiles with both enzymes were observed. In the case of *P. dumerilii*, we found a restriction pattern that is remarkably similar to that of *H. sapiens*, further supporting the hypothesis of high levels of CpG methylation in this species (Fig. 1b).

We next performed LUminometric Methylation Assay (LUMA) [53, 54] to obtain a quantitative assessment of CpG methylation and information about its dynamics during *Platynereis*’s life cycle. LUMA is an efficient method to measure global CpG methylation, based on gDNA digestion (at CCGG sites) by methylation-sensitive restriction enzymes followed by pyrosequencing. LUMA was performed on *P. dumerilii* gDNA extracted from ten different stages (Fig. 1c). Very high and similar methylation levels were found during embryonic/larval development (from 12 to 72 h post-fertilization, hpf; about 80% of CCGG sites are methylated; Additional file 2: Table S2). This level significantly decreases after the end of larval development, as shown in juvenile worms (early stages of post-larval growth; 4, 5, and 15 days post-fertilization, dpf), but nevertheless remains quite high (about 60–65% of methylated CCGG sites). The methylation level further decreases in older juvenile worms (3 months post-fertilization, mpf; about 27–32%) and subsequently increases when worms become sexually mature, significantly more in males (about 67-70%) than in females (about 43–56%).

To confirm the existence of gene body methylation in *P. dumerilii*, we performed bisulfite pyrosequencing [55] on CpG-rich parts of the coding region of two different genes, *Pdum-histone H4* and *Pdum-14-3-3-like*. These two genes were selected because they display stretches of CpGs in their coding region (7 and 16 CpGs for *Pdum-histone H4* and *Pdum-14-3-3-like*, respectively). Additionally, orthologs of these genes in other lophotrochozoan species were shown to have gene body methylation [28, 30]. Using DNA extracted from 72hpf larvae, we found high levels of methylation (between 65 and 87%) for 6 of the 7 CpGs of *Pdum-histone H4*, and low levels for all CpGs of *Pdum-14-3-3-like* (< 10%; Fig. 1d). These data therefore indicate that gene body methylation does indeed occur in *P. dumerilii* and that the level of methylation strongly differs in the two studied genes. In contrast, the level of methylation in the coding region of these two genes remains almost constant throughout the life cycle of the worm, as shown by bisulfite pyrosequencing using DNA extracted from five additional stages (Additional file 2: Table S3).

Taken together, these data indicate high-level CpG methylation in the *P. dumerilii* genome. In addition, the 5mC level is dynamic along the *P. dumerilii* life cycle and is significantly higher during embryonic/larval development as compared to post-larval stages. Striking changes in methylation level also occur during post-larval growth and when the worms reach sexual maturity. We also obtained evidence for gene body methylation in *P. dumerilii* and found that the level of CpG methylation in gene bodies is not uniform from one gene to another.

### *P. dumerilii* possesses a full ancestral-like DNA methylation and NuRD toolkit

Having established the existence of 5mC in *P. dumerilii,* we next aimed to identify proteins involved in writing, modifying, and reading this epigenetic mark, as well as putative NuRD components, in this species. For that purpose, we searched for *P. dumerilii* orthologs of proteins known to exert these functions in mammals (Additional file 1: Fig. S1) through a sequence-similarity approach using reciprocal best BLAST with *Homo sapiens* and *Mus musculus* sequences as queries. We found putative *P. dumerilii* orthologs for all investigated proteins/protein families (Additional file 4: Fig. S3). Sequences of all the identified proteins can be found in Additional File 5. As 5mC and NuRD proteins are often characterized by the presence of particular domains or association of domains, we searched for conserved domains present in the retrieved *P. dumerilii* proteins. In most cases, we found domains that are consistent with orthology relationships inferred from BLAST analyses (Additional file 4: Fig. S3).

**Fig. 2:**
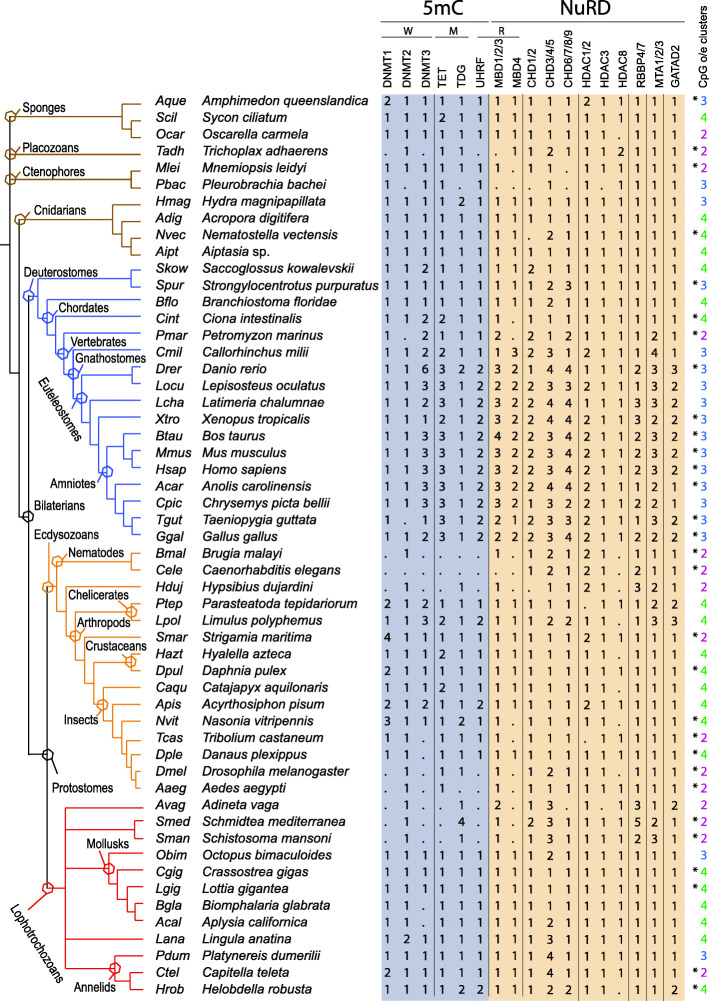
DNA methylation and NuRD toolkit in metazoans. On the left is shown a phylogenetic tree of the 54 metazoan species for which we identified DNA methylation and NuRD genes. Branches of this phylogenetic tree are color-coded (brown for non-bilaterians, blue for deuterostomes, orange for ecdysozoans, and red for lophotrochozoans). Polytomies highlight uncertainties about the relationships between bilaterian and non-bilaterian groups and within lophotrochozoans. Major phylogenetic groups are shown by hexagons placed on the tree nodes defining these groups. Additional taxonomic information: *Saccoglossus kowalevskii* belongs to hemichordates, *Strongylocentrotus purpuratus* to echinoderms, *Branchiostoma floridae* to cephalochordates, *Ciona intestinalis* to urochordates, *Hypsibius dujardini* to tardigrades, *Adineta vaga* to rotifers, *Schmidtea mediterranea* and *Schistosoma mansoni* to platyhelminthes, and *Lingula anatina* to brachiopods. The number of members found for each gene family is indicated for each species. Dots indicate that we failed to identify any members. Chd1/2, Chd6/7/8/9, and Mbd4 gene families which do not encode NuRD members are also indicated. For each species, the type of methylation (from 1 to 4) inferred from CpG o/e clustering [48] is also shown (asterisks indicate data derived from a previous study [48]). Type 1 corresponds to ultra-low gene body methylation, type 2 to low gene body methylation, type 3 to gene body methylation, and type 4 to mosaic DNA methylation (see [48] for details). W = Writers, M = Modifiers, R = Readers. Sequences of the identified proteins, multiple alignments, and phylogenetic trees can be found in Additional file 5 and Additional file 6: Figure S4

**Fig. 3:**
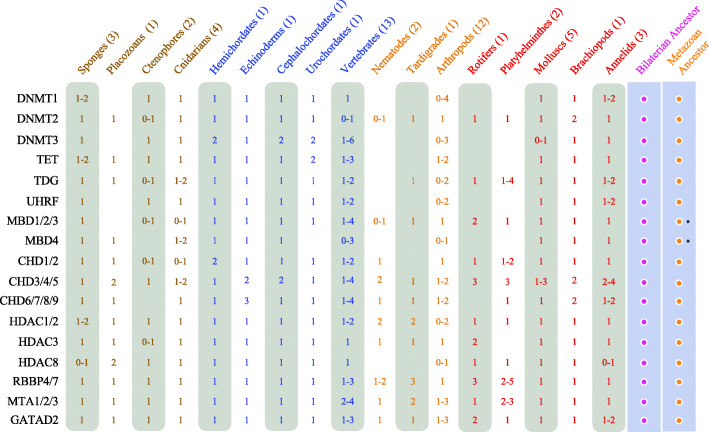
Evolution of DNA methylation and NuRD gene families in metazoans. The number (or range of numbers) of members of each family/subfamily in the indicated phylogenetic groups is shown (none if no members are detected). The number of studied species in each phylogenetic group is indicated next to the group name. Two final columns summarize the putative ancestral set of all studied families/subfamilies in the last common ancestors of metazoans and bilaterians. The putative ancestral set of families/subfamilies in the last common ancestor of eumetazoans (cnidarians + bilaterians) is the same as the one for the bilaterian ancestor and is not shown for sake of clarity. Asterisks highlight the fact that the inference of the presence of MBD1/2/3 and MBD4 families in the metazoan last common ancestor depends on whether we consider sponges as the sister group to all other animals. If ctenophores are considered as the sister group of all other animals, it is possible that only one *Mbd* gene (probably from the MBD1/2/3 subfamily) was present in the last common ancestor of all animals

Since defining orthology relationships only on BLAST analyses can be misleading, in particular when numerous paralogs are present, we turned to phylogenetic analyses to ascertain these relationships. To perform these analyses on a firm basis and to get insight into the evolution of the DNA methylation and NuRD toolkit in animals, we retrieved, by reciprocal BLAST searches using mouse and human sequences as queries, putative orthologs from 51 additional species from diverse animal phylogenetic groups. We ended up with a sample of 54 species from all major animal lineages (Fig. 2). Maximum likelihood (ML) trees were constructed for each protein family and are shown in Additional file 6: Fig. S4. We also searched for members of the different families in species from choanoflagellates, the sister group to animals, and used, when possible, these sequences as outgroups to root the phylogenetic trees. These phylogenetic trees allow us to confirm orthology relationships for all *P. dumerilii* proteins and to define the number of members of all protein families in the 54 investigated animal species (Fig. 2). We summarized the number of members for each defined family and, based on parsimony, we inferred the presence or absence of each protein family in the last common ancestors of animals and bilaterians (Fig. 3). All the identified proteins are listed in Additional file 2: Table S4 and their sequence can be found in Additional file 5.

The *P. dumerilii* genome encodes three Dnmt proteins that can be clearly assigned to the Dnmt 1, 2, and 3 subclasses (Additional file 6: Fig. S4A). The presence of these three subfamilies appears to be ancestral to animals (Fig. 3), as these three subfamilies are found in most non-bilaterians and in many species in the three bilaterian evolutionary lineages (Fig. 2). While only *dnmt2* genes were found in choanoflagellates, *dnmt1* and *dnmt3* genes have been reported in other eukaryotic groups, suggesting an early diversification of the Dnmt family during the evolution of eukaryotes [9]. Only a few gene duplications occurred for *dnmt1* (in particular in some arthropod species) and for *dnmt3* (in particular during vertebrate evolution in agreement with published studies; e.g., [56]). *dnmt* gene losses occurred in some species or lineages such as nematodes, rotifers, tardigrades, placozoans, and platyhelminthes. Absence of both *dnmt1* and *dnmt3* is correlated to the absence or very low abundance of cytosine DNA methylation as shown by CpG o/e ratio calculation (Fig. 2). *P. dumerilii* also possesses single *tet*, *tdg*, and *uhrf* genes, which likely corresponds to the ancestral situation in animals (Figs. 2 and 3; Additional file 6: Fig. S4B-D). Duplications of *tet* genes are infrequent, but two duplications nevertheless occurred in vertebrates [56]. *tet* genes are only absent in species that lack 5mC methylation and *dnmt1* and *dnmt3*, with the exception of dipterans which possess one *tet* gene. *tdg* is present in almost all investigated species in one copy, as expected for a gene involved in DNA repair. *uhrf* has a similar distribution to *tet*, being absent in species lacking 5mC, but in this case including dipterans. A single *uhrf* gene is found in most other species, with the notable exception of euteleostomes (bony vertebrates) that possess two genes (Fig. 2).

Phylogenetic analysis shows the existence of two large groups of Mbd proteins, one which contains Mbd1, Mbd2, and Mbd3 proteins from vertebrates (hereafter named Mbd1/2/3 group) and the other which contains vertebrate Mbd4 and MeCP2 proteins (Mbd4 group; Additional file 6: Fig. S4E). *P. dumerilii*’s genome encodes two Mbd proteins, one belonging to the Mbd1/2/3 group (putative NuRD component) and the other to the Mbd4 group. Presence of both Mbd1/2/3 and Mbd4 is observed in many species belonging to most animal lineages, including non-bilaterians such as sponges and cnidarians, strongly suggesting that the last common ancestor of animals possessed at least two *mbd* genes (Fig. 3). *mbd* gene losses occurred in few species, mainly in those that also lack cytosine DNA methylation. A few gene duplications also occurred, in particular in vertebrates in which both *mbd1/2/3* and *mbd4* ancestral genes underwent gene duplications.

The phylogenetic tree of Chd proteins comprises three large groups: one that includes vertebrate Chd3/4/5 proteins (hereafter named Chd3/4/5 group), the second vertebrate Chd1/2 (Chd1/2 group), and the third vertebrate Chd6/7/8/9 (Chd6/7/8/9 group; Additional file 6: Fig. S4F). Six *chd* genes have been found in *P. dumerilii*, one belonging to the Chd1/2 group, one to the Chd6/7/8/9 group, and four to the Chd3/4/5 group (putative NuRD components). Members of these three groups are found in almost all studied species, including non-bilaterians, indicating that presence of three different types of CHD proteins is ancestral to animals (Fig. 3). Only very few gene losses occurred. Gene duplications are more frequent, in particular in vertebrates and lophotrochozoans, including in annelids in which two to four members are found in the three studied species (Fig. 3).

Previous phylogenetic studies classified Hdac proteins into four classes (I, IIA/B, III, and IV) [57, 58]. Here we focused on class I to which belong *Hdac1*, *Hdac2*, *Hdac3*, and *Hdac8*, genes that encode members of the NuRD complex. Phylogenetic analysis showed the existence of three subgroups, Hdac1/2, Hdac3, and Hdac8 (Additional file 6: Fig. S4G). We found one member of each subgroup in *P. dumerilii*, as well as in almost all other investigated species, indicating that at least three class I *hdac* genes were present in the last common ancestor of all animals (Fig. 3). We found only very few gene losses (e.g., in nematodes). Duplications mainly occurred in arthropods and vertebrates. Single *rbbp4/7*, *mta1/2/3*, and *gatad2* genes are found in *P. dumerilii* (Additional file 6: Fig. S4H-J). At least one member of each of these subfamilies is found in all studied species, indicating that their presence is ancestral to animals (Fig. 3). Gene duplications occurred in vertebrates, ecdysozoans, and lophotrochozoans.

In conclusion, we have identified in *P. dumerilii* a complete set of writers, modifiers, and readers involved in 5mC methylation, as well as putative NuRD components. We additionally provide an animal-wide view of the evolution of the corresponding gene families (Fig. 2), which suggests that the last common ancestor of animals already possessed a complex repertoire of 5mC and NuRD toolkit genes (Fig. 3). Our analysis also indicates that the *P. dumerilii* repertoire is mostly composed of single-copy genes and likely close to the one present in the last common ancestor of bilaterians.

### DNA methylation and NuRD toolkit genes are dynamically expressed during development and regeneration in *P. dumerilii*

We next aimed to characterize the expression of DNA methylation and NuRD genes in *P. dumerilii*. We first took advantage of two previously published transcriptomic datasets corresponding to various developmental stages and adult conditions of *P. dumerilii*, available in a public database (PdumBase) [59]. The first dataset corresponds to embryonic developmental stages, ranging from 2 to 14hpf, with a time point every 2 h [49]. The second dataset comprises major larval stages (24 to 4dpf; five time points), juvenile stages (10dpf to 3mpf; five time points), and adult reproductive stages (males and females) [60]. Altogether, expression data for a total of 19 stages during embryonic and post-embryonic development, as well as male and female adult stages, are available.

**Fig. 4:**
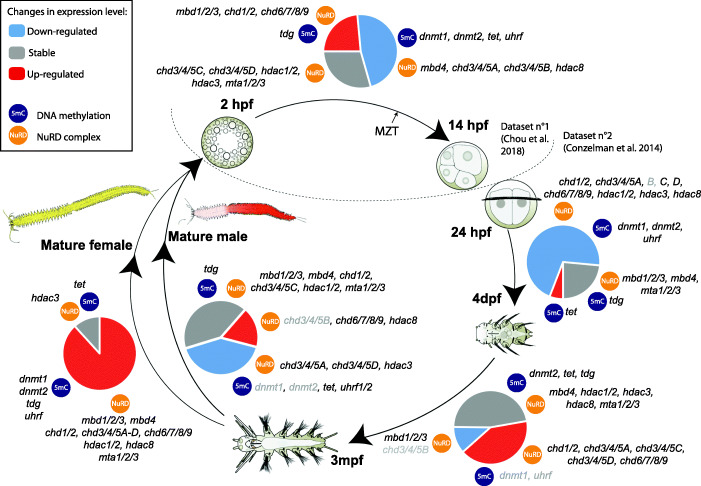
DNA methylation and NuRD genes are dynamically expressed during the *P. dumerilii* life cycle. The main steps of *P. dumerilii* life cycle are shown. Expression changes between consecutive stages are indicated as “downregulated” (expression level decreases with a fold change superior to two), “stable” (fold change inferior to two), and “upregulated” (expression level increases with a fold change superior to two). Proportions of these three categories are represented as color pie charts. Genes encoding 5mC toolkit and NuRD/NuRD-related proteins are mentioned. Gray letterings indicate genes with low expression (< 5 fragments per kilobase million, FPKM) in the compared stages. The two datasets that have been used are indicated [49, 60]. Maternal to zygotic transition (MZT) is shown at around 10hpf as previously suggested [49]. hpf = hours post-fertilization, dpf = days post-fertilization, mpf = months post-fertilization. For sake of clarity, *Pdum-* prefixes have been omitted for the gene names. Drawings of embryos, larvae, juvenile, and adult worms are adapted from [35]. Expression values for all the genes shown in this figure can be found in Additional file 7: Fig. S5

Expression values for most genes (exceptions are *Pdum-dnmt3* absent in the two sets of transcriptomic data and *Pdum-gatad2* and *Pdum-rbbp4/7* only found as chimeric transcripts) were recovered and can be found in Additional file 7: Fig. S5. High transcript levels are found for many studied genes in the earliest developmental stages (2–6hpf) and several of them belong to co-expression clusters defined by Chou et al. [49] as maternal gene clusters (clusters 1–4; Additional file 7: Fig. S5). This indicates that the *P. dumerilii* egg contains a large pool of maternal transcripts coding for DNA methylation proteins that could be used for embryonic development. To further analyze these expression data, we studied changes in expression during the main steps of *P. dumerilii* life cycle (Fig. 4). From 2hpf to 14hpf, a decrease in quantity of transcripts of about half of the genes, including genes coding for DNA methylation maintenance (*Pdum-dnmt1* and *Pdum-uhrf*), as well as putative members of the NuRD complex (*Pdum-chd3/4/5A-B* and *Pdum-hdac8*), is observed. From 24hpf to 4dpf, this decrease is found for most genes, including *Pdum-dnmt1* and *Pdum-uhrf*. In contrast, expression of *Pdum-tet* and *Pdum-tdg* is increased or stable, respectively. This is consistent with the decrease of the CCGG methylation level that we observed at the end of larval development (Fig. 1c). From 4dpf to 3mpf, a majority of genes have stable expression with the exception of the upregulation of every *chd* gene except *chd3/4/5B*, which is downregulated (Fig. 4). Transition from 3mpf to the adult stage is strikingly gender-specific: in males, most genes have stable or downregulated expression, while about 80% of the genes are strongly upregulated in females (Fig. 4; Additional file 7: Fig. S5), suggesting different occurrence and importance of DNA methylation during sexual maturation and gamete production between males and females.

**Fig. 5:**
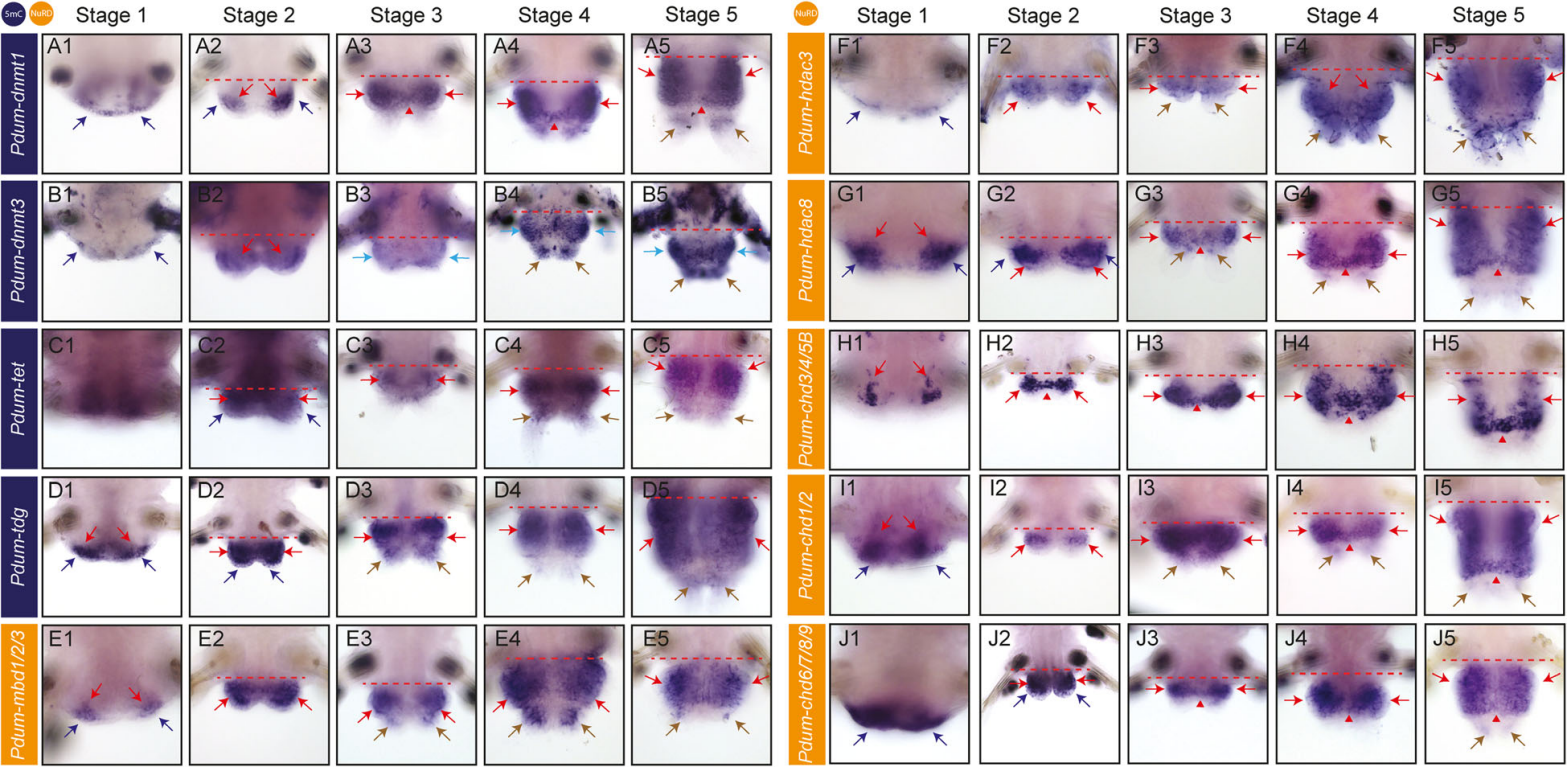
DNA methylation and NuRD genes are expressed during most or all stages of *P. dumerilii* regeneration. Expression patterns obtained by whole-mount in situ hybridization (WMISH) for genes whose name is indicated at the five previously defined stages of posterior regeneration [37] are shown. All panels are ventral views (anterior is up). Red dotted lines indicate the amputation plane in worms at stages 2 to 5, delineating the regenerated region (below the dotted lines) from the segment abutting the amputation plane (above the dotted lines). At stage 1, only a wound epithelium has already formed (dark blue arrows) and red arrows point to internal cells of the segment adjacent to the amputation. At the other stages, dark blue arrows point to epithelium covering the blastema, red arrows point to mesodermal cells of the blastema or developing segments (depending on stages), red arrowheads to the mesodermal part of the growth zone, light blue arrows to ectodermal expression, including segmental ectodermal stripes, and brown arrows to the base of anal cirri. Additional expression patterns are shown in Additional file 9: Fig. S7, Additional file 11: Fig. S9, and Additional file 12: Fig. S10

We next studied the expression of DNA methylation and NuRD genes during *P. dumerilii* posterior regeneration. To characterize in which part(s) and tissue(s) of the regenerated region these genes are expressed, we performed whole-mount RNA in situ hybridizations (WMISH) at all five stages of posterior regeneration (a schematic representation of regeneration stages can be found in Additional file 8: Fig. S6) [37], focusing on a set of ten genes that encode putative writers/modifiers/readers of 5mC or NuRD components. Representative expression patterns are shown in Fig. 5 and Additional file 9: Fig. S7. A schematic representation of the expression patterns can be found in Additional file 10: Fig. S8. We also tried to define the expression of the studied genes in non-amputated worms (to compare to the expression during regeneration) but failed to obtain any signal above the background level with our WMISH protocol, likely due to the presence of a thick cuticle around the fully differentiated segments of these worms [36]. As a proxy of non-amputated worms, we therefore used worms that have regenerated for 15 days (15 days post-amputation, dpa) and which show many well-differentiated segments lacking the thick cuticle that hampers WMISH in non-amputated worms. Representative expression patterns of the studied genes in these worms are shown in Additional file 11: Fig. S9. We were also able to detect the expression of some genes in worms fixed immediately after amputation (hereafter named stage 0), as the wound probably favors the penetration of the probes used for WMISH. Only very weak expression was however observed for most studied genes in stage 0 worms (Additional file 12: Fig. S10), and these expressions will not be further discussed.

*Pdum-dnmt1* and *Pdum-dnmt3* are weakly expressed in the wound epithelium at stage 1 (Fig. 5a1, b1). At stage 2, *Pdum-dnmt1* is strongly expressed in two internal groups of cells and in the lateral ectoderm (Fig. 5a2). Its expression extends in almost the whole blastema at stage 3 and is found in the regenerated growth zone (Fig. 5a3). At the same stages, *Pdum-dnmt3* is very weakly expressed in both mesodermal and ectodermal cells of the regenerated region (Fig. 5b2, b3). At stages 4 and 5, *Pdum-dnmt1* is expressed in the mesoderm of the developing segments, mesodermal and ectodermal growth zone, at the base of the anal cirri, and weakly in the lateral/dorsal ectoderm (Fig. 5a4, a5; Additional file 9: Fig. S7A). *Pdum-dnmt3* is expressed in the ventral ectoderm and at the base of the anal cirri (Fig. 5b4, b5). Broad and diffuse expression in the developing segments was observed for both genes in worms at 15dpa (Additional file 11: Fig. S9A, B). *Pdum-tet* expression is not reliably detected at stage 1 (Fig. 5c1). At stages 2 and 3, a weak expression is found in both internal and superficial blastemal cells (Fig. 5c2, c3), which continues at stages 4 and 5 and at which expression is also observed at the base of anal cirri (Fig. 5c4, c5). At 15dpa, *Pdum-tet* expression is found in both the mesoderm and ectoderm of the developing segments (Additional file 11: Fig. S9C). *Pdum-tdg* is strongly expressed at stage 1 in the wound epithelium and internal cells of the segment abutting the amputation plane (Fig. 5d1). At stages 2 and 3, it is broadly expressed in the whole blastema (Fig. 5d2, d3). From stage 3, *Pdum-tdg* is expressed in mesoderm and ectoderm of the developing segments, and in ectodermal and mesodermal growth zones, as well as weakly at the base of the anal cirri (Fig. 5d4, d5; Additional file 9: Fig. S7B). *Pdum-tdg* is expressed in the ventral ectoderm and in the developing parapodia at 15dpa (Additional file 11: Fig. S9D).

*Pdum-mbd1/2/3, Pdum-hdac3,* and *Pdum-hdac8* have roughly similar expression during posterior regeneration, *Pdum-hdac3* being expressed at most stages weaker than the two other genes. At stage 1, an expression is detected in two lateral patches of cells in and close to the wound epithelium (Fig. 5e1, f1, g1). The three genes are widely expressed in the blastema at stages 2 and 3 (Fig. 5e2, e3, f2, f3, g2, g3). Expression in the mesoderm and ectoderm of the developing segments, growth zone, and at the base of the anal cirri is observed at stages 4 and 5 (Fig. 5e4, e5, f4, f5, g4, g5; Additional file 9: Fig. S7C, D). Broad and diffuse expression patterns in the developing segments were observed for the three genes at 15dpa (Additional file 11: Fig. S9E-G). *Pdum-chd3/4/5B* expression is found at stage 1 in four small patches of internal cells close (but not adjacent) to the wound epithelium, two located ventrally and two dorsally (Fig. 5h1; Additional file 9: Fig. S7E). From 2dpa, we observed an intense expression in the mesodermal part of the regenerated region, including the mesodermal growth zone (Fig. 5h2–h5; Additional file 11: Fig. S9H). *Pdum-chd1/2* and *chd6/7/8/9* are expressed in cells in and close to the wound epithelium at stage 1, the latter having a much broader expression (Fig. 5i1, j1). At stage 2, both genes are expressed in superficial and internal cells of the regenerated region, in most or all cells for *Pdum-chd6/7/8/9* but only in a few cells for *Pdum-chd1/2* (Fig. 5i2, j2). Broad expression in mesodermal cells, including the growth zone, is observed at later stages (Fig. 5i3–i5, j3–j5). At stage 5, *Pdum-chd1/2* is also weakly expressed in the ectodermal growth zone (Additional file 9: Fig. S7F). At 15dpa, *Pdum-chd6/7/8/9* is weakly expressed in the developing segments (Additional file 11: Fig. S9I). We failed to detect significant expression of *Pdum-chd1/2* at 15dpa.

Altogether, we found that *P. dumerilii* DNA methylation and NuRD genes are dynamically expressed during embryonic, larval, and post-larval development, as well as during sexual maturation and regeneration. During this latter process, most genes are expressed from its earliest stages and their expression is later mostly found in blastemal cells, putative mesodermal and ectodermal stem cells of the growth zone, and cells of the developing segments (Additional file 10: Fig. S8). Observed patterns of expression show striking similarities with those previously reported for proliferation (*cycB* and *pcna* genes) and stem cell genes (e.g., *piwi*, *vasa*, *nanos*, and *myc* genes) [37], suggesting that DNA methylation and NuRD genes are mainly expressed in undifferentiated proliferating cells, including stem cells of the regenerated posterior growth zone.

### Decitabine reduces DNA methylation and impairs development, regeneration, and post-regenerative posterior growth in *P. dumerilii*

**Fig. 6:**
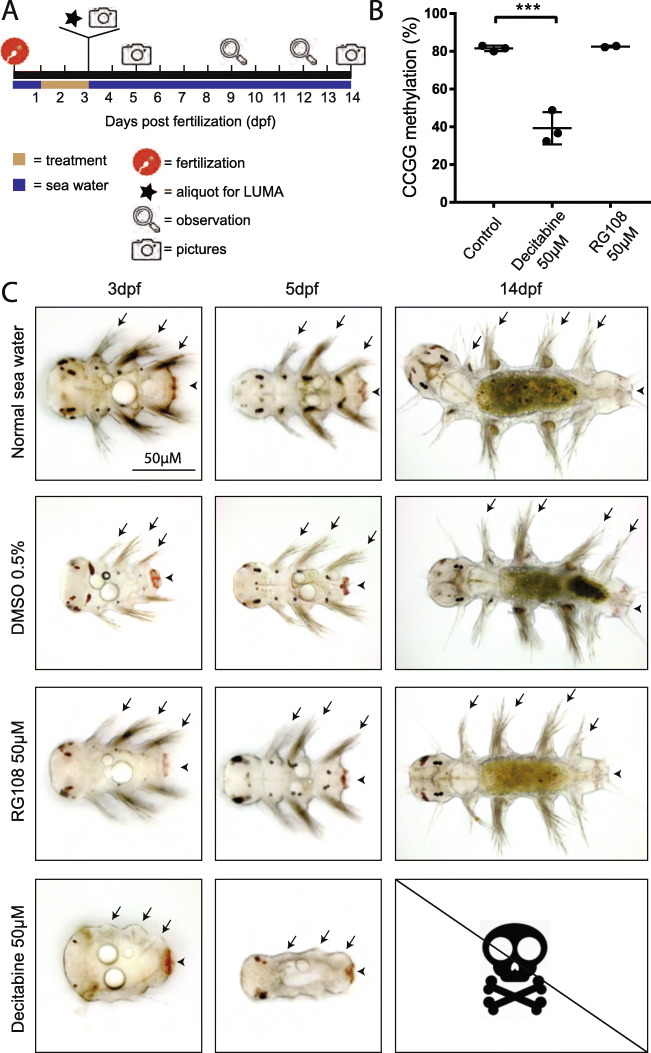
Decitabine treatment decreases DNA methylation level and impairs larval development. **a** Schematic representation of the experimental design. Larvae were treated with Decitabine (50 μM), RG108 (50 μM), or DMSO (0.5%; control) from 1 day post-fertilization (1dpf) to 3dpf. At 3dpf, a part of the batch of larvae was frozen for subsequent DNA methylation measurement with LUMA and remaining larvae were placed and kept in normal sea water until 14dpf. Observations were done at indicated time points and pictures taken at 3, 5, and 14dpf. **b** Graphic representation of CCGG DNA methylation as measured by LUMA for the different conditions (two or three biological replicates per condition and two technical replicates per biological replicate). Mean ± SD. One-way ANOVA, Dunnet post hoc test was performed (***: p < 0.001). The raw data can be found in Additional file 2: Table S2. **c** Morphological observations at 3, 5, and 14dpf. Ventral views of representative larvae/juvenile worms are shown (anterior on the left). At the three time points, RG108-treated larvae/juvenile worms show morphologies similar to those of controls (sea water and DMSO 0.5%). At 14dpf, like the control animals, RG108-treated worms have added a fourth segment. In contrast, at 3 and 5dpf Decitabine-treated larvae/juvenile worms display an abnormal morphology with strongly reduced appendages (parapodia; arrows) and a reduced pygidium (arrowheads). Massive death occurred in the 5 to 9dpf time period, so no Decitabine-treated worms could be observed at 14dpf. These experiments were performed twice using larvae from independent fertilizations

To test a possible role of DNA methylation during *P. dumerilii* regeneration, we tried to reduce 5mC levels using two well-known and widely used hypomethylating agents: Decitabine (5-aza-2′-deoxycytidine) and RG108 (N-Phthalyl-L-Tryptophan) [61–63]. Decitabine is incorporated in DNA and binds Dnmt1 irreversibly, leading to a progressive loss of DNA methylation through cell divisions. RG108 is a specific non-nucleoside inhibitor of Dnmt1, which acts by binding in a reversible manner to the active center of the enzyme. As these two drugs have never been used in *P. dumerilii*, we first tested their activity by treating larvae continuously from 1 to 3dpf with Decitabine or RG108 (Fig. 6a). Neither drugs caused significant lethality during treatment. DNA was extracted from larvae at 3dpf and CCGG methylation level measured using LUMA (Fig. 6b): Decitabine treatment leads to a 2.5-fold decrease of CCGG methylation (from 81.5 to 32.4%) while no significant effects were found for RG108. We also checked for morphological defects (Fig. 6c): larvae were observed either immediately after treatment (at 3dpf) or after washing out the drug and putting larvae in normal sea water until 5 or 14dpf. Larvae that had been treated with Decitabine presented morphological abnormalities at 3dpf, in particular reduced parapodia (worm appendages) bearing very few chaetae (extracellular chitinous structures) and a reduced pygidium (Fig. 6c). While abnormal, these larvae were alive and survived for a few more days. All animals did however die in the following days, possibly because of feeding defect (during this period normal young worms start to eat, dead Decitabine-treated worms consistently showed an empty gut). In contrast, RG108 treatment did not affect larval morphology (Fig. 6c). We therefore conclude that Decitabine can affect DNA methylation levels and larval development in *P. dumerilii*.

**Fig. 7:**
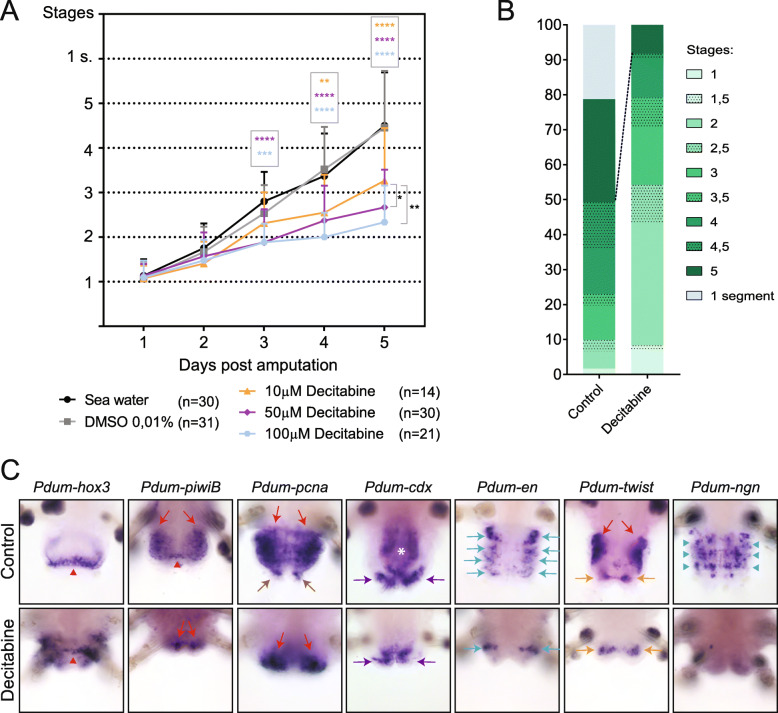
Decitabine treatment impairs posterior regeneration. **a** Graphic representation of the stages reached by control worms (normal sea water and DMSO 0.01%) and worms treated with three different concentrations of Decitabine every day for 5 days. Regeneration is delayed for 50 μM and 100 μM Decitabine conditions, from 3 days post-amputation (dpa) onwards, and for 10 μM Decitabine conditions, from 4dpa onwards. Three experiments, mean ± SD. 2-way ANOVA (p value: time p < 0.0001, treatment p < 0.0001, interaction p < 0.0001) with Tukey post hoc test (**: p < 0.01; ***: p < 0.001; ****: p < 0.0001). The number of worms used for these experiments is indicated in the figure. **b** Proportions of control and Decitabine-treated worms with different regeneration scores at 5dpa. Worms treated with the three different concentrations of Decitabine have been pooled. Most worms treated with Decitabine did not reach stage 5 at 5dpa, while about 50% of control worms reached this stage (some of which already having produced a first new segment). Supporting data values can be found in Additional file 2: Table S5. **c** Ventral views of WMISH at 5dpa of posterior part of control worms and worms treated with 50 μM Decitabine are shown. Expression of marker genes for different structures/tissues/cells during posterior regeneration has been studied, i.e., *Pdum-hox3* (growth zone), *Pdum-piwiB* (growth zone and segmental progenitors), *Pdum-pcna* (proliferating cells), *Pdum-caudal/cdx* (pygidium and growth zone), *Pdum-engrailed* (*Pdum-en*; segmental stripes), *Pdum-twist* (muscle progenitors), and *Pdum-neurogenin* (*Pdum-ngn*; neural progenitors) [37]*.* Red arrowheads point to an expression in the growth zone, red arrows to a mesodermal expression, brown arrows to an expression at the base of the anal cirri, violet arrows to an expression in the pygidium, light blue arrows to a segmental ectodermal expression, light blue arrowheads to an expression in neural progenitors, and orange arrows to an expression in pygidial muscles. White asterisks indicate an expression in the hindgut

To investigate potential consequences of a decrease of DNA methylation on regeneration, we treated worms with three concentrations of Decitabine (10 μM, 50 μM, and 100 μM) immediately after amputation for 5 days and scored the worms every day for the stage that had been reached based on a previously established staging system (Additional file 8: Fig. S6) [37]. We found a small number of deaths at 10 μM and 50 μM concentrations, while a 100 μM concentration appears to be much more harmful to worms (Additional file 2: Table S5). Some worms also underwent spontaneous amputation of their posterior part (autotomy) at some time points (Additional file 2: Table S5). These worms were excluded from the analysis. We found that Decitabine significantly delayed regeneration as compared to controls (DMSO 0.5 % and sea water), in a concentration-dependent manner (Fig. 7a). At 5 days post-amputation (dpa), while most control worms reached stage 4 or more, worms treated with Decitabine were mostly at stages 2 to 3 (Fig. 7b). No major abnormalities were observed at the morphological level in Decitabine-treated worms (not shown). To better understand how regeneration proceeds in the presence of Decitabine, we did Decitabine treatments (at 50 μM as this concentration shows low toxicity and pronounced effect on regeneration) from 0dpa to 5dpa, fixed treated worms at 5dpa, and performed WMISH for some of the genes whose expression was previously studied during normal regeneration [37]. The analyzed genes showed expression at 5dpa in Decitabine-treated worms that are similar to those of stage 2 or 3 in non-treated worms [37], indicating that regeneration is blocked in the presence of Decitabine (Fig. 7c). Abnormal expression patterns, never observed in non-treated animals, were nevertheless found in some treated worms for *Pdum-hox3* (growth zone marker; extended and/or mis-located expression domain), *Pdum-piwiB* (stem cell marker; no or reduced expression), and *Pdum-engrailed* (segment marker; incomplete expression stripes).

**Fig. 8:**
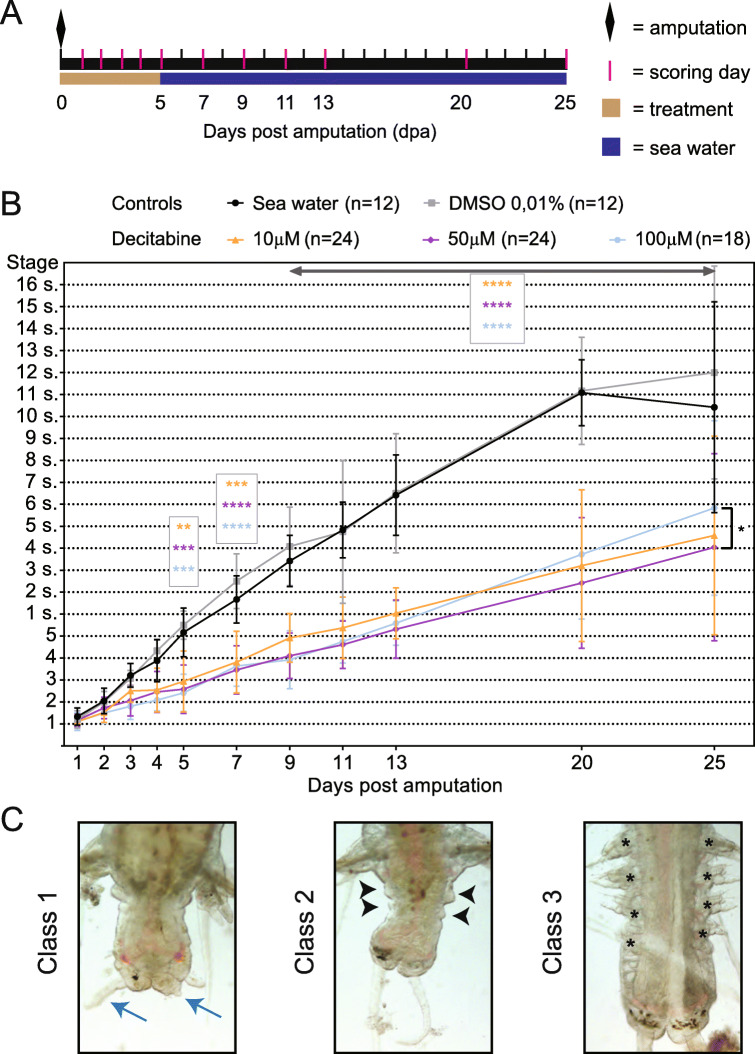
Decitabine treatment during posterior regeneration affects subsequent post-regenerative posterior growth. **a** Schematic representation of the experimental design. Worms were treated with Decitabine (10 μM, 50 μM, or 100 μM) or from amputation to 5 dpa. Control animals were treated with DMSO 0.01% or put in normal seawater. After washing out, worms were kept from 5dpa to 25dpa in normal seawater and observed at several time points until 25dpa. **b** Graphic representation of regeneration stages that have been reached or numbers of newly added segments by Decitabine-treated and control worms. A significant delay in post-regenerative posterior growth is observed in Decitabine-treated worms as compared to controls. Two experiments, mean ± SD, 2-way ANOVA (p value: time p < 0.0001, treatment p < 0.0001, interaction p < 0.0001) with Tukey post hoc test (**: p < 0.01; ***: p < 0.001; ****: p < 0.0001). Only p values corresponding to comparison to normal seawater are shown, as highly similar ones are obtained for comparison to DMSO controls. The number of worms used for these experiments is indicated in the figure. Supporting data values can be found in Additional file 2: Table S6. **c** Representative morphologies at 25dpa of worms belonging to the three defined classes (see main text for details). While class 3 worms showed well-differentiated segments with parapodia (black asterisks), no or reduced parapodia were observed in class 1 and class 2 (black arrowheads) worms, respectively. Small or abnormally shaped anal cirri (blue arrows) were also frequently observed in class 1 and 2 worms

It has been shown that mammalian cells treated with Decitabine only partially recover their initial methylation level, leading to an epigenetic “imprint” of drug exposure [64]. We hypothesized that Decitabine treatment could have long-term impacts in *P. dumerilii* and affect segment formation that follows regeneration (post-regenerative posterior growth [36, 37]). To test this hypothesis, we treated worms with Decitabine from 0 to 5dpa, then washed out the drug, put worms in normal sea water until 25dpa, checking their morphology and counting the number of segments that have been produced at six time points (Fig. 8a). As for the previous experiment, Decitabine treatments induced few worm deaths and autotomies (Additional file 2: Table S6). Most Decitabine worms recovered from the treatment and were able to reach stage 5 and undergo posterior growth (Fig. 8b). Decitabine-treated worms continued to be delayed as compared to control worms, had a reduced number of newly added segments at 25dpa (treated worms had about 4 to 6 segments compared to about 10 to 12 segments for controls), and showed morphological abnormalities (Fig. 8b, c). A reduced number of newly added segments was due not only to a marked delay during regeneration, but also to a reduced rate of segment addition after the drug had been washed out (Additional file 13: Fig. S11A). A high variability was observed among Decitabine-treated animals compared to controls (Additional file 13: Fig. S11B-F). We defined three classes of animals based on their morphology and the number of newly added segments at 25dpa (Fig. 8c; Additional file 14: Fig. S12). Class 1 animals (30.6% of Decitabine-treated worms) show a characteristic bottleneck-like shape with a marked constriction between non-regenerated and regenerated regions, no or few newly added segments, and no or abnormal anal cirri. These worms are prone to undergo autotomy. Class 2 animals (61.6%) have an abnormal body shape, a reduced number of newly added segments, an absence of well-differentiated parapodia on newly added segments, and no or abnormal anal cirri. Class 3 worms (7.9%) have a morphology and number of newly added segments similar to control animals.

Taken together, these observations indicate that Decitabine treatments during regeneration have long-term effects and affect subsequent post-regenerative posterior growth, possibly by affecting growth zone regeneration. Some Decitabine-treated worms were however able to add new segments in an almost normal manner, which led us to hypothesize that the growth zone was not impacted similarly in all animals. To point out a potential link between regeneration of the growth zone and ability to later add segments, we performed a multiple correlation analysis (Additional file 15: Fig. S13). In control worms, as expected, only positive correlations were observed, which means that, for example, worms with numerous segments at 20dpa already had a high number of segments at 11dpa. In contrast, in Decitabine-treated worms, while there were positive correlations for closely related days of scoring (for example: 2 to 3dpa, 3 to 4dpa, …), negative correlations were also found and suggested that treated worms that regenerated faster eventually produced less segments. It has been shown that the growth zone is regenerated and becomes functional, producing news segments, at about 3dpa [37]. Our interpretation is therefore that worms with high regeneration scores (scored at stage 3 or more) at 5dpa regenerated a dysfunctional growth zone in the presence of Decitabine, which later led to a null or reduced production of segments, the few segments produced additionally displaying morphological abnormalities. Worms with low scores (less than 3) probably did not regenerate their growth zone during the Decitabine treatment period and did it after 5dpa in the absence of the drug, which led to the formation of a functional growth zone and therefore to normal segment addition. Our data therefore suggest that Decitabine affects the functionality of the growth zone. Consistently, expression of growth zone, stem cell, and segment markers (*Pdum-hox3*, *Pdum-piwiB* and *Pdum-engrailed*, respectively) is affected in some Decitabine-treated worms (Fig. 7c).

**Fig. 9:**
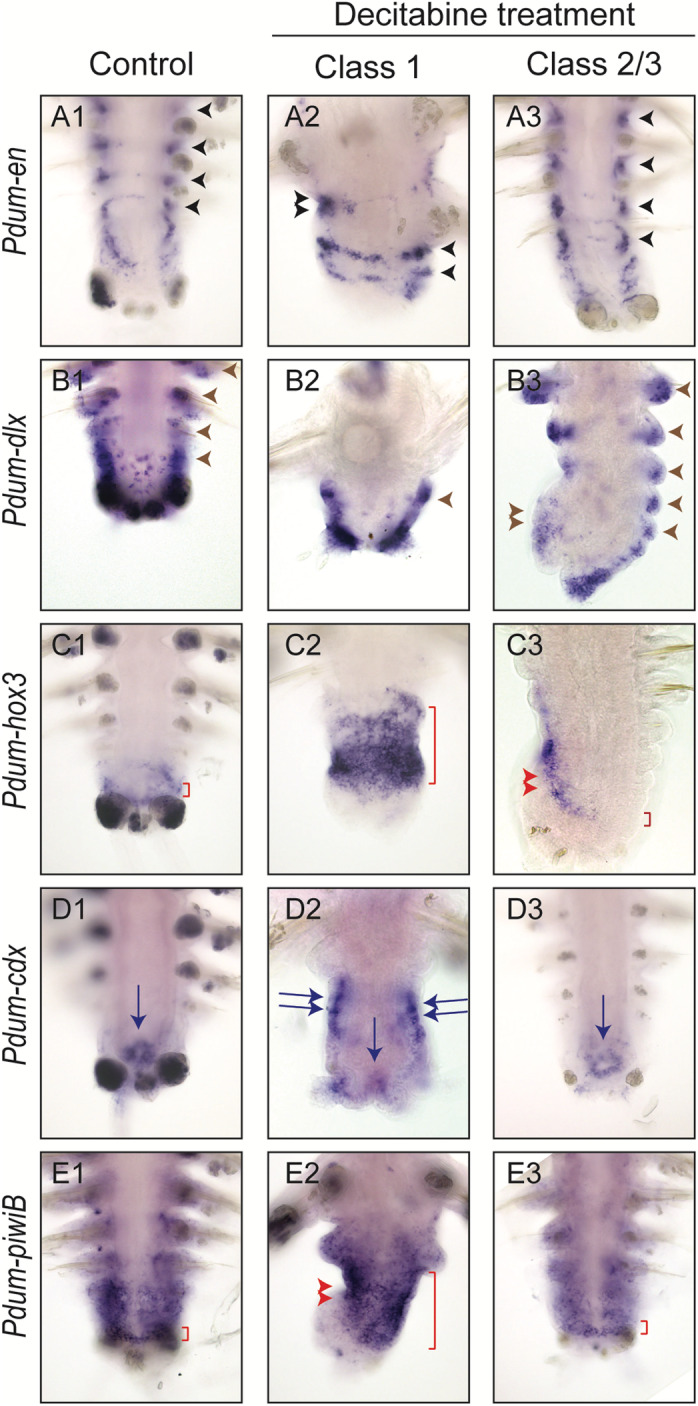
Gene expression at 25 days post-amputation in worms that have been treated with Decitabine during regeneration. WMISH at 25 dpa of posterior parts of control worms and worms treated with 50 μM Decitabine from 0 to 5dpa for selected markers, i.e., *Pdum-engrailed* (*Pdum-en*; segmental stripes), *Pdum-dlx* (parapodia and anal cirri), *Pdum-hox3* (growth zone), *Pdum-caudal/cdx* (pygidium and growth zone), and *Pdum-piwiB* (growth zone and segmental progenitors). Two Decitabine-treated worms with more or less altered morphologies (class 1 and class 2/3) are shown for each gene. In A1–A3, black arrowheads point to segmental stripes of *Pdum-en* expression. In A2, the double black arrowhead points to an incomplete expression stripe. In B1–B3, brown arrowheads point to the expression of *Pdum-dlx* in developing parapodia. In B3, a reduced *Pdum-dlx* expression is observed on one side of the worm (double brown arrowhead). In C1, red bracket delineates the expression of *Pdum-hox3* in the ectodermal growth zone. A very large domain of *Pdum-hox3* is found in class 1 worms (red bracket in C2). An abnormal expression pattern is also observed in class 2/3 worms (red bracket and double red arrowhead in C3). In D1–D3, blue arrows point to the expression of *Pdum-cdx* in the posterior gut region and double blue arrows point to an ectopic expression of the gene in developing segments (D2). In E1–E3, red bracket delineates a strong expression of *Pdum-piwiB* in the mesodermal growth zone. *Pdum-piwiB* is also expressed in the developing mesoderm in a graded manner. In E2, a strong and broad expression is found throughout the regenerated region (red bracket and double red arrowhead)

As described above, worms treated with Decitabine from 0 to 5dpa show morphological abnormalities at 25dpa. To better understand these alterations, we performed WMISH on Decitabine-treated worms at 25dpa for a set of previously studied marker genes [37]. A wide range of abnormal expression patterns were found in Decitabine-treated worms (Fig. 9). This includes a reduced number of segmental stripes of *Pdum-engrailed* in worms with few morphologically visible segments (Fig. 9a1–a3), reduced expression of *Pdum-dlx* (which is normally expressed at the base of anal cirri and in parapodia), on one side of the worm (Fig. 9b1-b3), as well as ectopic expression of *Pdum-hox3*, *Pdum-cdx*, and *Pdum-piwiB* in developing segments (Fig. 9c1–e3), which are consistent with persistent defects in growth zone functionality.

Finally, we investigated whether Decitabine might have effects over an even longer time period. We treated worms with Decitabine from 0 to 5dpa, then put them in normal sea water until 25dpa, performed a second amputation one segment anterior to the first amputation plan (meaning that we eliminated the regenerated region plus one segment), and scored these worms at several time points until 18 days post-second amputation (18dpSa; Additional file 16: Fig. S14A). Control and Decitabine-treated worms regenerated properly and similarly after this second amputation and were able to add new segments at a similar rate (Additional file 16: Fig. S14B). A slight but significant delay, however, was observed for worms treated with 10 μM Decitabine at 18dpSa (Additional file 16: Fig. S14B) and about 10–15% of Decitabine-treated worms showed minor defects at the level of their parapodia (Additional file 16: Fig. S14C). A same proportion of worms that were class 1 or 2 at 25dpa showed abnormalities after the second amputation at 18dpSa (Additional file 16: Fig. S14C). Multiple correlation analysis showed that, for both control and Decitabine-treated worms, only positive correlations were found (Additional file 16: Fig. S14D, E). Therefore, Decitabine treatments after a first amputation have only very minor effects on regeneration and post-regenerative posterior growth occurring after a second amputation.

On the whole, our data show that Decitabine decreases methylation levels in *P. dumerilii* and affects larval development and regeneration. During regeneration, it impairs post-regenerative posterior growth occurring in the absence of the drug, a long-term effect that could be due to defects in the regeneration of the stem cell-containing growth zone.

## Discussion

A wealth of studies, mainly conducted in mammals, pointed out that cytosine DNA methylation modulates gene expression and is of primary importance for the regulation of embryonic development and stem cell properties [7]. Mechanisms and biological roles of this epigenetic modification in non-vertebrate species, and in other processes such as regeneration, has however been much less studied. This is at least in part due to the fact that canonical non-vertebrate developmental models such as *Drosophila melanogaster* and *Caenorhabditis elegans* [9], and canonical regeneration models such as planarians [65], almost entirely lack 5mC and are therefore of no help for understanding its functions in non-vertebrates. In this article, we study cytosine DNA methylation and its roles during development, regeneration, and post-regenerative growth in an emerging developmental and evolutionary biology model species, the marine annelid *Platynereis dumerilii.*

### *P. dumerilii* genome displays a high and dynamic level of CpG methylation

One of the main aims of our study was to define the extent and pattern of DNA methylation in the annelid *P. dumerilii*. Three main types of methylation patterns are classically described in animals: (i) global high-level methylation often presented as characteristic of vertebrates, in which a large majority of CpGs are methylated; (ii) mosaic low-/intermediate-level methylation in which only some genomic regions are methylated (interspaced with non-methylated ones) found in many diverse non-vertebrates; (iii) ultra-low/no methylation found in some species such as *Drosophila melanogaster*, *Caenorhabditis elegans*, and *Schmidtea mediterranea* [11, 48, 50, 66]. Here, we combined in silico (computation of CpG o/e ratios [43, 48]) (Fig. 1a) and experimental (genomic DNA digestion with methylation-sensitive enzymes and LUminometric Methylation Assay (LUMA [53, 54])) (Fig. 1b, c) approaches to identify high levels of CpG methylation in *P. dumerilii* (up to more than 80% at some developmental stages) comparable to those of mammalian somatic cells [5, 67]. While this high-level vertebrate-like methylation may seem surprising in a non-vertebrate species, it has also been demonstrated by whole-genome bisulfite sequencing in the sponge *Amphimedon queenslandic*a (and is supported by the low CpG o/e ratios of this species) [33] and the crustacean *Parhyale hawaiensis* [32], showing that high-level methylation can also occur in non-vertebrate species. While our data indicate gene body methylation, as CpG o/e ratio calculation (Fig. 1a) and bisulfite pyrosequencing (Fig. 1d) were done on coding regions, further analyses, in particular whole-genome bisulfite sequencing, will be required to better characterize *P. dumerilii* genome methylation. Further characterization would include, for example, the determination of whether there is 5mC depletion at gene promoters (like in *A. queenslandica* and vertebrates [33]) and to point out putative 5mC methylation in repetitive DNA (such as transposable elements), which is widely found in eukaryotes [7, 9]. In addition, the question of whether high-level genome methylation could be more widespread in animals than expected deserves to be experimentally addressed, as the patterns and extent of DNA methylation are known in rather few animal species so far and CpG o/e ratio calculations suggested that high-level methylation might exist in other non-vertebrate animals [48] (Fig. 2; Additional file 3: Fig. S2).

An intriguing observation made about *P. dumerilii* DNA methylation is the sharp decrease of the 5mC level, observed by LUMA, from more than 80% of methylated CCGG sites at embryonic/larval stage (12 to 72 h post-fertilization, hpf) to about 60–65% at post-larval stages (4, 5, and 15 days post-fertilization, dpf) (Fig. 1c). It is important to note that for technical reasons we were unable to obtain LUMA data from very early developmental stages, the earliest studied one being 12hpf which roughly corresponds to a late gastrula stage [35]. Later time points (24, 48, and 72hpf) correspond to organogenesis stages during which many cells and tissues are differentiating. We cannot extrapolate methylation levels of earlier 0–12hpf stages, neither can we exclude that demethylation after fertilization may occur followed by remethylation during cleavage/early gastrulation stage, similarly to what has been observed in mammals [7]. In mammals, the methylation level remains high in somatic cells from the epiblast stage onwards and a transient decrease of this level only occurs in the germ cell lineage [7]. In contrast, in *P. dumerilii* a sharp decrease in the DNA methylation level happens in the 72hpf to 4, 5, and 15dpf time period, which corresponds to a major transition in the life cycle of the animal, the metamorphosis of the larva into a benthic feeding juvenile worm that adds new segments by posterior growth [35, 36]. The methylation level further decreases in older juvenile worms (3mpf) and subsequently re-increases at the time of sexual maturation (a process known as epitoky), another key transition in the life cycle of *P. dumerilii*, as it involves dramatic changes in the morphology, anatomy, and behavior of the worm [35]. Such changes of DNA methylation levels at critical developmental transitions have also been reported in other non-vertebrates, for example at the time of metamorphosis in the oyster *Crassostrea gigas* [31] and in *Xenopus* [68], or during caste attribution in social hymenopterans (e.g., [41, 69]). Changes in the DNA methylation level could therefore be important for key developmental transitions in distantly related animals, a tempting hypothesis that nevertheless remains to be experimentally tested.

### *P. dumerilii* possesses an ancestral-like repertoire of DNA methylation and NuRD toolkit genes that show dynamic expression during development and regeneration

We retrieved and analyzed a large dataset of genes encoding putative writers, modifiers, and readers of 5mC, as well as NuRD members and related proteins. This dataset corresponds to 17 gene families/subfamilies for which we reported the presence/absence and number of members in each of the 54 studied species (Fig. 2). Using these data, we were able to infer parsimoniously the likely presence or absence of each gene family in the last common ancestors of metazoans and bilaterians (Fig. 3). Our conclusions are in agreement with, and reinforce, those of previous studies made on a more limited set of gene families and/or studied species (e.g., [22, 27, 30, 33, 56, 70]). A complex DNA methylation and NuRD toolkit was present in the last common ancestor of all animals that probably possessed at least one member of the 17 analyzed families/subfamilies (Fig. 3). This toolkit has been remarkably conserved during animal evolution as shown by the small number of missing orthologs in most species (Fig. 2), a number likely to be overestimated as most species only benefit from draft genome assemblies. Exceptions are species in which gene losses occurred frequently such as nematodes, some insects, and flatworms. In these species, very low/no genome methylation was reported and several genes of the methylation toolkit (mainly *Dnmt* and *Uhrf* genes) are absent. Similar reduced toolkits are found in the placozoan *Trichoplax adhaerens* and the tardigrade *Hypsibius dujardini*, suggesting that these two species may also have no or very reduced genome methylation (as also hinted by CpG o/e values). Besides these extreme cases, there is no clear connection between the number of genes encoding 5mC machinery proteins and the pattern of genome methylation inferred from CpG o/e calculation (Fig. 2) or experimental analysis [64].

We identified members of all 17 gene families/subfamilies in *P. dumerilii* with single-copy members for 16 of them (Fig. 2; the only exception is the CHD3/4/5 subfamily for which four members were found), consistent with the hypothesis that *P. dumerilii* belongs to a slow-evolving lineage in which few gene loss and duplication events occurred [71, 72]. To have a first glimpse of the possible function of these genes in *P. dumerilii*, we looked at their expression at the mRNA level, using transcriptomic data (for developmental and adult stages) and whole-mount in situ hybridization data (for regeneration stages). Most DNA methylation and NuRD genes are expressed at most or all stages of the *P. dumerilii* life cycle (Fig. 4), which is expected for genes encoding epigenetic regulators likely involved in multiple steps of the life cycle of the animal. However, many genes show dynamic expression during embryonic/larval development, at juvenile and adult stages, as well as during regeneration. Interestingly, at the transition between larval/post-larval stages, expression of *Pdum-dnmt1* and *Pdum-uhrf*, which could be involved in DNA methylation maintenance like their vertebrate orthologs [16], decreases, while expression of *Pdum-tet*, likely involved in demethylation [18, 19], increases (Fig. 4). This suggests that the marked decrease in the DNA methylation level observed at this transition (see above) could be due to either passive (Dnmt1-mediated) or active (Tet-mediated) demethylation, or to both mechanisms.

We also characterized the expression of several DNA methylation and NuRD genes during regeneration and found these genes to be expressed at all stages of the process (Fig. 5). Strikingly, all the studied genes are expressed at stage 1 in cells of the wound epithelium and/or cells of the immediately adjacent segment, which would fit with an early role of these genes during regeneration. From stage 2 onwards, all genes are expressed in the regeneration blastema and subsequently in the regenerating segments, in patterns that are reminiscent of those previously reported for stem cell and proliferation genes [37], suggesting an expression in proliferating cells and therefore that DNA methylation could be important for the formation of the regenerated structures from blastemal cells. These expression data are also consistent with the hypothesis that DNA methylation might be involved during multiple steps of regeneration in *P. dumerilii*.

### Decitabine treatments suggest that DNA methylation is involved in *P. dumerilii* development, regeneration, and post-regenerative growth

To study the putative functions of DNA 5mC methylation during *P. dumerilii* development and regeneration, we treated larvae or regenerating worms with Decitabine (5-aza-2'-deoxycytidine), a hypomethylating agent that causes cell-division-dependent DNA demethylation by blocking Dnmt1 activity [61–63]. While widely used in vertebrates, especially humans, this chemical has only been sparsely used in non-vertebrates [73–75] and, to our knowledge, never used during regeneration or in annelids. We therefore first obtained evidence that Decitabine does indeed lead to DNA demethylation in *P. dumerilii* by showing a 2.5-fold decrease of CCGG methylation in 72hpf larvae treated with Decitabine for 2 days, as compared to control animals (Fig. 6b). Decitabine is thus an appropriate tool to study possible roles of DNA methylation in *P. dumerilii.*

We observed three main effects for Decitabine. First, when applied during larval development (from 1 dpf to 3dpf), Decitabine produced significant morphological defects but did not block development or led to larval death (Fig. 6c). Some developmental processes, such as appendage (parapodia) and pygidium formation, are strongly affected (as shown by the extreme reduction of these structures in Decitabine-treated larvae), while others, such as segmentation, are less or not affected. This could indicate differential requirements of DNA methylation for specific developmental process. Alternatively, it could be due to the mode of action of Decitabine that induces a progressive loss of 5mC through cell divisions, meaning that a significant decrease in 5mC level is probably achieved only several hours after the start of Decitabine treatment. In this view, Decitabine treatment may not affect developmental processes that happen before or soon after 24hpf, for example segmentation (segmental stripes of *Pdum-engrailed* expression can already be seen at 18hpf [76]), while strongly affecting processes starting later in development, for example pygidium formation that is still ongoing at the end of the treatment at 3dpf [77]. In the future, it would be interesting to further assess the effects of Decitabine on *P. dumerilii* development, in particular through the use of other temporal windows of treatment, including windows spanning early development (0–24hpf) which could produce much more severe defects, as observed in the oyster *C. gigas* in which gastrulation was severely impacted when Decitabine was applied on early development stages [73]. Effects of Decitabine on germ cell specification and development, which are well characterized in *P. dumerilii* [78], would also be an interesting topic for future examination, given the importance of DNA (de)methylation in the germ cell lineage [7].

A second clear effect of Decitabine is that it strongly delays regeneration when applied on worms for 5 days, from 0 to 5dpa (Fig. 7a, b). Indeed, at day 5 after amputation, Decitabine-treated worms mostly reached stage 2 or 3, while controls worms reached stage 4 or 5. This is similar to what has been observed when applying the proliferation inhibitor hydroxyurea on regenerating worms [37]. On the one hand, this is consistent with the antiproliferative effect of Decitabine observed in humans, for whom this drug has been used for a long time, at a high concentration, as an anticancer cytotoxic drug preventing DNA replication, thereby blocking cell proliferation and leading to cell death [79, 80]. On the other hand, several elements suggest that the effect of Decitabine on regeneration in *P. dumerilii* could be due to demethylation and not to an impairment of DNA replication. The first and most compelling argument is that we used low Decitabine concentrations that effectively led to significant demethylation (Fig. 6b). As this Decitabine-induced demethylation can only occur after several cell divisions, it could not be obtained if Decitabine was used at concentrations that block cell divisions and has a cytotoxic effect [79, 80]. In humans, Decitabine is now used at low dosage to maximize its hypomethylating action, which elicits better anticancer responses than when used at higher concentrations [79]. Secondly, while there are many cell divisions which happens during larval development (1 to 3dpf time period; e.g., [81]), Decitabine treatment leads to 3dpf larvae with a size and a body shape similar to control ones strongly arguing against an antiproliferative effect of Decitabine at the used concentrations. Thirdly, after washing out the drug, Decitabine-treated worms were able to rapidly resume regeneration in a normal manner, which does not support the claim of Decitabine having significant cytotoxic effects. Our current hypothesis is therefore that Decitabine delays regeneration through its hypomethylating effect and therefore that DNA methylation may be required for proper regeneration in *P. dumerilii*. This hypothesis is at odds with what has been described in vertebrates in which demethylation has been suggested to be a driver of regeneration, in the axolotl limb [40], zebrafish fin [39], and the chick retina [82]. Additional experiments will be required to further test the requirement of DNA methylation in *P. dumerilii* regeneration and identify the involved mechanisms. An obvious possibility would be that massive demethylation induced by Decitabine may interfere with the expression of genes involved in regeneration, as suggested by reduced expression of *Pdum-hox3*, *Pdum-piwiB*, and *Pdum-engrailed* in Decitabine-treated worms (Fig. 7c), thereby hampering successful regeneration.

A third compelling effect of Decitabine is that, when applied during 5 days after amputation, it interfered with post-regenerative posterior growth that occurs once regeneration has resumed and been completed in absence of the drug (Fig. 8a, b). These worms showed a reduced rate of segment addition compared to control animals and displayed segments with various abnormalities and severely affected gene expression patterns at 25dpa, i.e., 20 days after drug removal (Figs. 8 and 9). Based on multiple correlation analysis (Additional file 15: Fig. S13) and altered gene expression patterns of stem cell, growth zone, and segmental marker genes in Decitabine-treated worms at 5dpa (Fig. 7c), we suggest that drug-mediated DNA hypomethylation affects gene expression in stem cells of the growth zone, thereby impairing its functionality and subsequent posterior growth. Abnormal expression of growth zone markers is still observed in some worms at 25dpa (Fig. 9), consistent with the hypothesis of an epigenetic modification (hypomethylation) that has been transmitted during the many cell divisions that occur during the 5dpa to 25dpa time period. Along the same line, parapodia formation, which starts after the drug has been removed, is also strongly affected in many Decitabine-treated worms, suggesting that DNA hypomethylation might have been transmitted from growth zone stem cells to parapodial progenitors, leading to altered gene expression during parapodia formation, as shown for *Pdum-dlx* (Fig. 9), and defects in this process.

Our data therefore suggest that 5mC DNA methylation is important for the function of stem cells of the growth zone in *P. dumerilii*, i.e., somatic adult stem cells, and their capability to produce differentiated structures such as segments and appendages. Growth zone stem cells express a set of genes, such as *piwi*, *vasa*, and *nanos*, that constitutes the Germline Multipotency Program (GMP) shared with primordial germ cells and pluripotent/multipotent somatic stem cells in other animals [36, 83]. In mammals, in the absence of DNA methylation, ES cells keep their stem cell identity and their self-renewal ability, but their differentiation is almost completely abolished, due to a failure to upregulate germ-layer-specific genes and to silence pluripotency genes (e.g., [84]). Roles of DNA methylation have also been described in mammalian adult stem cells for both stem cell self-renewal and differentiation capabilities (e.g., [85–87]). A tempting hypothesis is therefore that Decitabine-induced hypomethylation in *P. dumerilii* growth zone stems cells may alter their ability to produce differentiated cell lineages required for segment and appendage formation. Supporting this is our observation of ectopic expression of growth zone markers, *Pdum-hox3*, *Pdum-cdx*, and *Pdum-piwiB*, in developing segments of 25dpf worms that had been treated from 0 to 5dpf with Decitabine (Fig. 9), suggesting that the silencing of stem cell markers in segmental progenitors/differentiated cells might not be properly done in a hypomethylated context. Further tests of this hypothesis would require a specific assessment of methylation levels in growth zone stem cells and their progeny, which is currently not possible due to the lack of tools to isolate and culture these cells.

## Conclusion

We provide data that strongly suggest that the genome of *P. dumerilii* is highly methylated and that the methylation level changes during development. We show that *P. dumerilii* harbors a mostly single-copy repertoire of DNA methylation and NuRD genes that have dynamic expression patterns during development and regeneration. Using the hypomethylating drug Decitabine, we obtained functional data in favor of an involvement of 5mC DNA methylation during development, regeneration, and post-regenerative growth. These data also suggest that Decitabine-induced hypomethylation of growth zone stem cells could alter their capability to produce differentiated segments. However, the mechanisms by which DNA methylation acts in *P. dumerilii* remain elusive and further studies will be required to define whether it controls transcription and/or other aspects of gene expression regulation. The possible presence and roles of 5mC in repetitive DNA such as transposable elements should also be characterized. When considered as a whole, our data provide the first evidence of the roles of the 5mC epigenetic mark during regeneration outside of vertebrates. Our study also lays the groundwork for using *P. dumerilii* as a new non-vertebrate model to study 5mC DNA methylation, and other epigenetic regulations, in particular those involving histone modifications, during development, regeneration, and stem cell-based growth.

## Methods

### *P. dumerilii* gene identification and cloning

Putative *P. dumerilii* orthologs of genes known to encode protein involved in 5mC methylation or to belong to NuRD complex (and related proteins) were identified by BLAST searches [88] on available transcriptomic and genomic data, using *H. sapiens* and *M. musculus* protein sequences as queries. Sequences of all identified genes can be found in Additional file 5. Conserved domains in *P. dumerilii* and *H. sapiens* proteins (Additional file 4: Fig. S3) were identified using InterPro 80.0 [89] and the NCBI CD-Search Tools [90]. High-fidelity PCR with gene-specific primers (listed in Additional file 2: Table S7) was used to amplify gene fragments using as a template cDNA from mixed larval and regenerating stages with, for some genes, a touchdown approach. PCR products were purified (740609, Macherey Nagel), and TA cloned in the pCR2.1 vector (K450001, Thermo Fisher), following the manufacturer’s instructions. Sequences of cloned genes were verified by Sanger sequencing (Eurofins Genomics). GenBank accession numbers for *P. dumerilii* genes: MW250929 to MW250948.

### Phylogenetic analyses and establishment of orthology relationships

Putative members of all studied 5mC machinery gene families were identified by sequence similarity searches (reciprocal best BLAST hit approach [88]) on publicly available genome sequences of 51 different species belonging to all major animal clades (Fig. 2) using *H. sapiens* and *M. musculus* sequences as queries. Redundant sequences were manually discarded and, in some cases, incomplete short sequences were concatenated. To obtain outgroups for subsequent phylogenetic analyses, members of all families were also searched for in two choanoflagellate species, *Monosiga brevicollis* (*Mbre*) and *Salpingoeca rosetta* (*Sros*), as well as, in the case of unsuccessful searches, in available transcriptomes of other choanoflagellate species. For some gene families (DNMT, CHD, and HDAC), sequences from distinct but related families (DNMT5, SNF2L1, and class II/IV HDACs) were retrieved to be used as outgroups for phylogenetic tree rooting. All identified sequences can be found in Additional file 5. Databases used to retrieve these sequences are listed in Additional file 2: Table S8.

Multiple alignments were obtained using MUSCLE 3.8.31 [91] (available on the MPI Bioinformatics Toolkit platform [92]) and subsequently improved either manually or using the BMGE 1.1.2 software [93]. Multiple alignments were visualized using SEAVIEW 5.0.4 [94]. Maximum likelihood (ML) analyses were performed using the PHYML 3.0 software [95, 96]. The amino acid substitution model was defined by the software using SMS with the Akaike Information Criterion [97]. Other parameters (such as proportion of invariable sites and Gamma shape parameter) were estimated from the datasets by the software. The tree improvement method was NNI and statistical supports for internal branches of trees were assessed by approximate likelihood-ratio test (aLRT) [98, 99]. Phylogenetic trees were handled using FigTree 1.4.

### CpG o/e ratio calculations

Notos [43] was used to calculate and model CpG o/e (observed/expected) as described in Aliaga et al. [48] for the *P. dumerilii* reference transcriptome and transcriptomes of some of the 54 studied species for which CpG o/e ratios were not previously determined [48]. The formula (CpG / (C × G)) × (L^2 / L−1) and a minimum length of 200 bp were used.

### *P. dumerilii* breeding culture and posterior amputation procedure

*P. dumerilii* embryos, larvae, and juvenile worms were obtained from a breeding culture established at the Institut Jacques Monod (Paris, France). For regeneration experiments, amputations of posterior parts were performed on juvenile worms of 3–4 months and 30 to 40 segments as previously described [37].

### Study of DNA methylation levels in *P. dumerilii*

Methylation of genomic DNA (gDNA) of *P. dumerilii*, *N. vectensis* (adult), *D. melanogaster* (adult), *B. floridae* (adult), *H. sapiens* (HCT116 cells), and *M. musculus* (embryonic stem cells, mESC) was assessed by digesting 500 to 800 ng of gDNA with 1 μL of restriction enzymes HpaII or MspI in 50 μL containing 1X FastDigest green buffer (FD0514 and FD0544, Thermo Scientific) for 15 min at 37 °C. Then, 300 ng of undigested gDNA and from each restriction reaction were loaded in a 1% agarose gel with molecular weight marker (N3232, NEB). A 160 V current was applied for 30 min in a Midigel tank (370000, Apelex), and ethidium bromide was used with a UV-transilluminator for revelation. This experiment was repeated twice using genomic DNA from independent DNA extractions.

To evaluate global DNA methylation levels, we used a LUminometric Methylation Assay (LUMA) based on enzymatic digestion and pyrosequencing [53, 54]. For each studied stage, at least two biological replicates (larvae and worms from different fertilizations) were analyzed with at least two technical replicates for each biological replicate. Samples were washed twice with 200 mM PBS 0.1% Tween on ice before freezing at − 80 °C. DNA was extracted using the DNA/RNA All prep kit (80204, Qiagen) following the manufacturer’s instructions. DNA was stored at − 20 °C prior to LUMA analysis. Prior to LUMA analysis, DNA integrity and sample purity were assessed using Tapestation (Agilent). Only samples with a DNA Integrity Number superior or equal to 8 were kept for subsequence analysis. LUMA experiments were performed as in Karimi et al. [53] with internal controls (HCT116 WT and DKO (Dnmt1−/−; Dnmt3b−/−)). Then, 250 ng of gDNA was digested with HpaII+EcoRI or MspI+EcoRI for 4 h at 37 °C. Then, samples were analyzed in a PyroMark Q24 (Qiagen). The instrument was programmed to add dNTPs as follows: dATP; dGTP + dCTP; dTTP; H2O as control; dGTP + dCTP; dATP; dTTP. Peak heights were calculated using the PyroMark Q24 software (Qiagen). The HpaII/EcoRI and MspI/EcoRI ratios were calculated as (dGTP + dCTP)/mean (dATP; dTTP) for the respective reactions. The percentage of methylated CCGG sites was defined as: 100 × [1 − (HpaII/EcoRI)/(MspI/EcoRI)].

To measure CpG methylation levels in the *14-3-3-like* and *Histone H4* genes, we used bisulfite pyrosequencing. Pyrosequencing primers (listed in Additional file 2: Table S7) were designed using the PyroMark Assay Design Software 2.0 (Qiagen). Then, 500 ng of genomic DNA was subjected to bisulfite conversion using the EpiTect Bisulfite Kit (Qiagen, Catalog No. 59124). PCR reactions were performed in a final volume of 25 μL, using the Pyromark PCR kit (Qiagen, Catalog No. 978703), with one of the primers biotinylated and containing 12.5 ng of bisulfite-treated DNA. The initial denaturation/activation step was performed at 95 °C, 15 min, followed by 50 cycles of 30 s at 94 °C, 30 s at 54 °C, 45 s at 72 °C, and a final extension step at 72 °C for 10 min. The quality and the size of the PCR products were evaluated by running 5 μL of each PCR product on 1.5% (w/v) agarose gel in a 0.5X TBE buffer. Biotinylated PCR products (20 μL) were immobilized on streptavidin-coated sepharose beads (GE Healthcare, 17-5113-01). DNA strands were separated using the PyroMark Q24 Vacuum Workstation, and biotinylated single strands were annealed with 0.375 μM sequencing primer and used as a template for pyrosequencing. Pyrosequencing was performed using PyroMark Q24 Advanced (Qiagen, Catalog No. 9002270) according to the manufacturer’s instructions, and data about methylation at each CpG was extracted and analyzed using the PyroMark Q24 Advanced 3.0.0 software (Qiagen). For each studied stage, two biological replicates (larvae and worms from different fertilizations) were analyzed with two technical replicates for each biological replicate.

### Whole-mount in situ hybridizations (WMISH) and imaging

For probe synthesis, plasmid containing appropriate cDNA were purified (740588 and 740412, Macherey Nagel), digested (NEB enzyme), and used to produce digoxygenin-labeled RNA antisense probes (Synthesis with Roche reagents: 11093274910 and 10881767001 or 10810274001; Purification with 740955, Macherey Nagel) as previously described [36]. WMISH were done as previously described [37, 100]. As in previous studies that used this WMISH protocol [37, 100], WMISH with sense probes only gave non-specific labeling in the mucus-secreting glands which are found in the pygidium and parapodia. For each stage and each gene, at least five individuals were analyzed. Bright-field images were taken on a Leica microscope. Adjustment of brightness and contrast were performed using Photoshop software.

### Decitabine and RG108 treatments

Stock solution of 200 mM Decitabine (5′-Aza-2′-deoxycytidine) and 10 mM RG108 (N-Phthalyl-L-Tryptophan) in DMSO were diluted in seawater to obtain different concentrations as described in the “Results” section. DMSO controls correspond to a concentration of DMSO in seawater corresponding to that of 100 μM Decitabine condition. In order to assess methylation levels with LUMA after Decitabine or RG108 treatment, larvae were kept 2 days (1 to 3dpf) in 30 ml of Decitabine- or RG108-containing seawater before washing and freezing. Two or three biological replicates (larvae from different fertilizations) were analyzed with two technical replicates for each biological replicate. For morphological studies, larvae were washed out, kept in normal seawater, and fed from 5dpf onwards. Larvae were anesthetized with 7.5 % MgCl_2_ before observations. The experiment was repeated twice using larvae from different fertilizations.

Amputated worms were placed individually in 12-well plates in 2 ml of Decitabine solution or control solution that was changed every day for 5 days. For posterior growth analyses, worms were subsequently placed in normal sea water until 25dpa. For some experiments, at 25dpa, worms were amputated a second time one segment anterior to the first amputation plane, and posterior parts were fixed for WMISH. Numbers of worms used for the different experiments are indicated in the corresponding figures and supplementary figures (and their legends).

### Scoring and statistical analysis

The scoring system established in Planques et al. [37] was used to score worms during posterior regeneration and post-regenerative posterior growth. Graphic representation of transcriptomic, LUMA, and morphological experiments with corresponding statistical analyses were performed using Prism 7 software (GraphPad). Statistical tests that have been used are indicated in the legends of figures and supplementary figures. R was used for multiple correlation computation and representation. Holm correction was applied for significance calculation [101].

## Supplementary Information

**Additional file 1:** **Figure S1**. DNA methylation and NuRD toolkit genes in mammals. (A) Proteins involved in DNA methylation and demethylation include Dnmt3A/B responsible for *de novo* 5-methyl-cytosine (5mC) formation, Dnmt1 required for 5mC maintenance during DNA replication, Uhrf1 which binds 5mC and recruits Dnmt1, and Tet and Tdg which are involved in active demethylation. Passive demethylation through cell divisions is also indicated. (B) The Nucleosome Remodeling and Deacetylase complex (NuRD complex) is recruited on methylated DNA and represses gene transcription. The NuRD complex is composed of two subcomplexes: one made of Mbd2/3 (which binds methylated cytosines), Gatad2a/b, and Chd3/4, and which acts on chromatin remodeling; and the other composed of Rbbp4/7, Mta1/2/3 and Hdac1/2, which stimulates histone deacetylation. For the sake of simplicity, the NuRD complex is depicted in a schematic manner that does not reflect its real stoichiometry.

**Additional file 2:** **Tables S1-S8**. Supplementary Tables. **Table S1**: CpG o/e ratios obtained with Notos. **Table S2**: LUMA data. **Table S3**: Methylation levels of stretches of CpGs in *Pdum-Histone H4* and *Pdum-14-3-3-like* genes defined by bisulfite pyrosequencing at different stages of the *P. dumerilii* life cycle. **Table S4**: Sequences used for phylogenetic analyses. **Table S5**: Lethality and autotomy induced by Decitabine treatment (short-term experiments). **Table S6**: Lethality and autotomy induced by Decitabine treatment (long-term experiments). **Table S7**: Primers used for *P. dumerilii* gene cloning and bisulfite pyrosequencing. **Table S8**: Databases used to retrieve sequences for phylogenetic analyses.

**Additional file 3:** **Figure S2**. Additional CpG o/e ratio calculations. Histograms of CpG o/e ratio for several species (whose name is indicated on top) for which this ratio has not been previously calculated. In each histogram, the red line indicates the estimated density, the vertical blue bar shows the estimated mean value, and the shaded blue bar represents bootstrap confidence intervals of 95%. PM = probability mass. Clusters are those defined in Aliaga et al. [48]. The color code for metazoan groups is indicated and is as in Fig. 2.

**Additional file 4:** **Figure S3**. *P. dumerilii* 5mC and NuRD machinery genes and proteins. All identified *P. dumerilii* genes are listed with the identification of the corresponding gene model in the Pdumbase reference transcriptome [49], excepted for *dnmt3*, identified in our unpublished regeneration transcriptome. Schematic representations of *P. dumerilii* (*Pdum*) and corresponding Human (*Hsap*) proteins are also shown, highlighting conserved domains found in these proteins and the position of these domains. Sequences of the *P. dumerilii* and Human proteins can be found in Additional file 5.

**Additional file 5:** Sequences used for phylogenetic analyses, multiple alignments, and phylogenetic trees. This file contains all protein sequences and multiple alignments used for phylogenetic analyses in fasta format, as well as the obtained phylogenetic trees in Newick format.

**Additional file 6:** **Figure S4**. Phylogenetic trees of 5mC and NuRD toolkit proteins. Maximum likelihood (ML) trees constructed with PhyML are shown. Statistical supports (aLRT values) for all nodes are indicated with a color code provided in the inset. Terminal branches are colored using the shown color code also used in Fig. 2. *P. dumerilii* sequences are in bold and indicated by arrows. (A) DNMT proteins. The three subfamilies DNMT1, 2 and 3 are indicated. In the DNMT3 subfamily, the vertebrate-specific groups DNMT3A, B and -like are also shown. We used a distantly related sequence (Dnmt5) from *Acanthamoeba castellanii* (*Acas*, an amoeba) as outgroup to root the phylogenetic tree. We also retrieved Dnmt sequences from two choanoflagellates species, *Monosiga brevicollis* (*Mbre*) and *Salpingoeca rosetta* (*Sros*), which all belong to the DNMT2 subfamily. (B) TET proteins. The three vertebrate-specific groups TET1, 2 and 3 are indicated. We used midpoint rooting for this tree as we were unable to find *Tet* genes in choanoflagellates or another suitable outgroup. (C) TDG. The phylogenetic tree is rooted using choanoflagellate sequences as outgroup. (D) UHRF. The two vertebrate-specific groups UHRF1 and 2 are indicated. We used midpoint rooting for this tree as we were unable to find *Uhrf* genes in choanoflagellates or another suitable outgroup. (E) MBD proteins. The two subfamilies MBD1/2/3 and MBD4 subfamilies are indicated. Vertebrate-specific groups are also shown. While not found in *S. rosetta* and *M. brevicollis*, a single *Mbd* gene was found in three other choanoflagellates (*Helgoeca nana* (*Hnan*), *Salpingoeca urceolata* (*Surc*), and *Acanthoeca spectabilis* (*Aspe*)) for which extensive transcriptomic data have been produced. Two of these choanoflagellate Mbd sequences (*Hnan* and *Aspe*) form a monophyletic group that was used as outgroup to root the tree, while the third one (*Surc*) clusters with MBD 4 proteins. (F) CHD proteins. The three subfamilies CHD1/2, CHD3/4/5 and CHD6/7/8/9 are indicated. Vertebrate-specific groups are also shown. We used the distantly related SNF2L1 sequence from Human as outgroup to root the phylogenetic tree. (G) Class I HDAC proteins. The three subfamilies HDAC1/2, HDAC3 and HDAC8 are indicated. Vertebrate-specific HDAC1 and 2 groups are also shown. Class II and IV HDACs from Human were used as outgroup to root the phylogenetic tree. We also retrieved HDACs from choanoflagellates, three of which clustering with the outgroup, two with HDAC3 proteins, and two with HDAC1/2 proteins. (H) RBBP4/7 proteins. Vertebrate-specific RBBP4 and 7 groups are shown. The phylogenetic tree is rooted using choanoflagellate sequences as outgroup. (I) MTA1/2/3 proteins. Vertebrate-specific MTA1, 2 and 3 groups are shown. (J) GATAD2 proteins. Vertebrate-specific GATAD2-alpha and GATAD2-beta groups are shown. For (I) and (J), midpoint rooting has been used in the absence of choanoflagellate members of these two gene families and the absence of other suitable outgroups.

**Additional file 7:** **Figure S5**. Expression level of 5mC and NuRD machinery genes during *P. dumerilii* development and along its life cycle. A histogram reporting FPKM values at 19 stages (developmental and adult stages) for all indicated genes is shown. The FPKM values from embryonic stages and those for the other stages cannot be compared, as having been calculated from two independent RNA-seq studies. These two datasets are therefore separated by a black vertical dashed line. Black horizontal dotted lines highlight a 5 FPKM threshold about which a gene can be considered as significantly expressed, as its expression can usually be detected by *in situ* hybridization [49]. Co-expression clusters are those defined by Chou et al. [49]. Hpf: hours post-fertilization; dpf: days post-fertilization; mpf: months post-fertilization.

**Additional file 8:** **Figure S6**. A schematic representation of *P. dumerilii* posterior regeneration. On the top row is depicted a 3-4-month-old juvenile worm (with 30 to 40 segments). Its posterior part (5 segments, growth zone, and pygidum) is eliminated by amputation (the red dotted lines indicate the amputation plane). Regeneration occurs at the posterior extremity of the anterior body region. The region delineated by the dotted black lines corresponds to the part of the regenerating worms that is shown in WMISH pictures (Figs. 5 and S7). It comprises the posterior part of the posteriormost differentiated segment (S), in which parapodia (black arrows) associated with glands (asterisks) can be seen, plus the regenerated region (region indicated by the two-headed arrow; red dotted lines show the position of the amputation plane). Anterior is up and posterior down. The regenerated region increases in size as regeneration proceeds, from stage 1 to 5 of the process [37]. At stage 1 (reached 1 day post-amputation, 1dpa), wound healing is achieved. A small blastema is formed at stage 2 (2dpa) and molecular analyses indicate that the growth zone is already regenerated at this stage. A regenerated anus can be observed at this stage. At stage 3 (3dpa), the regenerated region has increased in size and small anal cirri are present. The use of molecular markers showed that the growth zone has already produced at least one segment and tissue differentiation has started in the pygidium. The large blastema found at stage 4 (4dpa) contains a differentiated pygidium with long anal cirri. Tissue differentiation also starts in the two or more segments that have been produced by the growth zone. Segments are however still not morphologically visible. Stage 5 (5dpa) corresponds to the end of regeneration. At this stage, a fully differentiated pygidium is found, and segmentation of the regenerated region becomes visible at the morphological level (presence of visible parapodial primordia for example). From this stage onwards, post-regenerative posterior growth starts and a growing number of morphologically well visible segments with parapodia and segmental grooves between adjacent segments is observed.

**Additional file 9:** **Figure S7**. Additional expression data of 5mC and NuRD genes during regeneration. Whole-mount *in situ* hybridizations (WMISH) for the genes whose name is indicated are shown. In all panels except E, anterior is up and the regeneration stage for each picture is indicated. In all panels except E, red dotted lines indicate the amputation plane. Dorsal (D) and posterior (P) views are shown. Light blue arrowheads = ectodermal growth zone, light blue arrows = ectoderm of developing segment, red arrows = groups of internal cells in the segment adjacent to the amputation plane.

**Additional file 10:** **Figure S8**. Schematic representation of expression patterns of 5mC and NuRD genes during regeneration. In all panels, anterior is up and the regeneration stage for each drawing is indicated. Ventral schematic representations are shown for all stages and a dorsal schematic representation is also provided for stage 5. The color code for the different tissues is provided in the inset.

**Additional file 11:** **Figure S9**. Expression of 5mC and NuRD genes in worms at 15 days post-amputation. Whole-mount *in situ* hybridizations (WMISH) for the genes whose name is indicated are shown at 15 days post-amputation (15dpa). These worms are used as a proxy for non-amputated worms [36]. In all panels, anterior is up. All images are ventral views. Dark blue arrows point to an ectodermal expression including an expression in the ventral nerve cord, red arrows point to mesodermal cells of the developing segments, and red arrowheads to the mesodermal part of the growth zone. We failed to detect significant expression for *Pdum-chd1/2*.

**Additional file 12:** **Figure S10**. Expression of 5mC and NuRD genes in worms immediately after amputation. Whole-mount *in situ* hybridizations (WMISH) for the genes whose name is indicated are shown. In all panels, anterior is up. Only weak and diffuse expression was found for the genes that are shown and we failed to detect significant expression for the three other genes (*Pdum-mbd1/2/3*, *Pdum-hdac8* and *Pdum-chd1/2*). *Pdum-dnmt1* and *Pduchd3/4/5B* are expressed in a few ectodermal cells (dark blue arrows); *Pdum-dnmt3*, *Pdum-tet* and *Pdum-hdac3* are expressed in mesodermal cells (red arrows); and *Pdum-tdg* and *Pdum-chd6/7/8/9* are largely expressed in the ectoderm including the ventral nerve cord (light blue arrows).

**Additional file 13:** **Figure S11**. Rate of segment addition and individual scoring of Decitabine-treated worms. (A) Graphic representation of the rate of segment addition in controls (DMSO 0.01% and sea water) and Decitabine-treated worms (worms that showed autotomy were excluded). (B-F) Graphic representation of scoring of individual control (B and C) and Decitabine-treated (D-F) worms until 25dpa. Two experiments, mean ± SD. For the analysis of the rate of segment addition, 1-way ANOVA was performed with Dunnett post hoc test (**: p < 0.01; ***: p < 0.001; ****: p < 0.0001). For conditions comparison, 2-way ANOVA was performed (Source of variation: Time p < 0.0001, Treatment p < 0.0001, Interaction p = 0.0875) with Dunnett post hoc test (*: p < 0.05; **: p < 0.01; ***: p < 0.001). The number of worms used for these experiments is indicated in the figure.

**Additional file 14:** **Figure S12**. Classification of Decitabine-treated worms based on morphological defects. Three classes of worms can be defined based on the reported morphological defects. For each class, representative worms at five different time points after amputation are shown. Green arrowheads = characteristic constriction between the non-regenerated and regenerated regions, pink arrows = very reduced or absent anal cirri, blue arrows = reduced/abnormal anal cirri, green arrows = narrowed regenerated region, black arrowheads = abnormal parapodia.

**Additional file 15:** **Figure S13**. Multiple correlation analysis between regeneration and segment addition. (A-B) Statistical analysis of the regeneration score correlation in (A) control and (B) Decitabine-treated worms. Blue dots show positive correlations and red dots negative correlations. The dot size is proportional to the correlation score and significant correlations are highlighted with asterisks. Spearman correlation with Holm post hoc test (* for p < 0.05).

**Additional file 16:** **Figure S14**. Analysis of long-term Decitabine effects on regenerating and growing worms after a second amputation. (A) Schematic representation of the experimental design. Worms were treated with Decitabine (10 μM, 50 μM, or 100 μM), or DMSO (0,01 %; control) or kept in normal sea water (control) for five days following amputation (5 days post-amputation, dpa). Decitabine was washed out and worms were kept in normal sea water until 25dpa (see Fig. 8). A second amputation was performed (which removed the regenerated region) and worms were kept in normal sea water until 18 days post-second amputation (18dpSa). Observations were done at indicated time points (pink bars). (B) Graphic representation of the stages reached by control worms (normal sea water and DMSO 0,01%) and Decitabine-treated worms after the second amputation. A significant delay was observed for worms treated with 10 μM as compared to controls at 18dpSa. Worms treated with the other concentrations of Decitabine regenerated and added segments similarly to controls. Two experiments, mean ± SD, 2-way. ANOVA (p value: Time p < 0.0001, Treatment p = 0.0283, Interaction p = 0.0663) with Tukey post hoc test (*: p < 0.05). Only p values corresponding to the comparison to normal sea water are shown (similar values were obtained for the comparison to DMSO controls). The number of worms used for these experiments is indicated in the figure. (C) Most Decitabine worms that were class 1 or class 2 at 25dpa (after first amputation) regenerated after a second amputation without any morphological abnormalities (84,2% and 88,1%, respectively), but some of them show minor morphological defects in parapodia and chaetae formation (15,8% and 11,9%, respectively). (D and E) Multiple correlation analysis between regeneration/segment addition after first and second amputation. Only positive correlations were observed for both control and Decitabine-treated worms after a second amputation. Blue dots indicate positive correlations and red dots negative correlations. The dot size is proportional to the correlation score and significant correlations are highlighted with stars. Spearman correlation with Holm post hoc test (* for p < 0.05).

## Data Availability

All data generated or analyzed during this study are included in this published article, its supplementary information files and publicly available repositories. Accession numbers for *P. dumerilii* DNA methylation and NuRD tookit genes are GenBank MW250929 to MW250948. Expression values from RNA-seq data were retrieved from PdumBase [59, 102]. Supporting data values can be found in Additional file 2: Table S1-S6. Protein sequences, multiple alignments, and phylogenetic trees can be found in Additional file 5.
