# Supplementary material for: DNA methylation atlas and machinery in the developing and regenerating annelid Platynereis dumerilii
Source: BMC Biol. 2021 Aug 3;19:148. doi: 10.1186/s12915-021-01074-5 (PMC8330077; doi:10.1186/s12915-021-01074-5)
Supplement: Supplementary file 1 — Additional file 1: Figure S1. DNA methylation and NuRD toolkit genes in mammals. (A) Proteins involved in DNA methylation and demethylation include Dnmt3A/B responsible for de novo 5-methyl-cytosine (5mC) formation, Dnmt1 required for 5mC maintenance during DNA replication, Uhrf1 which binds 5mC and recruits Dnmt1, and Tet and Tdg which are involved in active demethylation. Passive demethylation through cell divisions is also indicated. (B) The Nucleosome Remodeling and Deacetylase complex (NuRD complex) is recruited on methylated DNA and represses gene transcription. The NuRD complex is composed of two subcomplexes: one made of Mbd2/3 (which binds methylated cytosines), Gatad2a/b, and Chd3/4, and which acts on chromatin remodeling; and the other composed of Rbbp4/7, Mta1/2/3 and Hdac1/2, which stimulates histone deacetylation. For the sake of simplicity, the NuRD complex is depicted in a schematic manner that does not reflect its real stoichiometry. [file 12915_2021_1074_MOESM1_ESM.pdf]

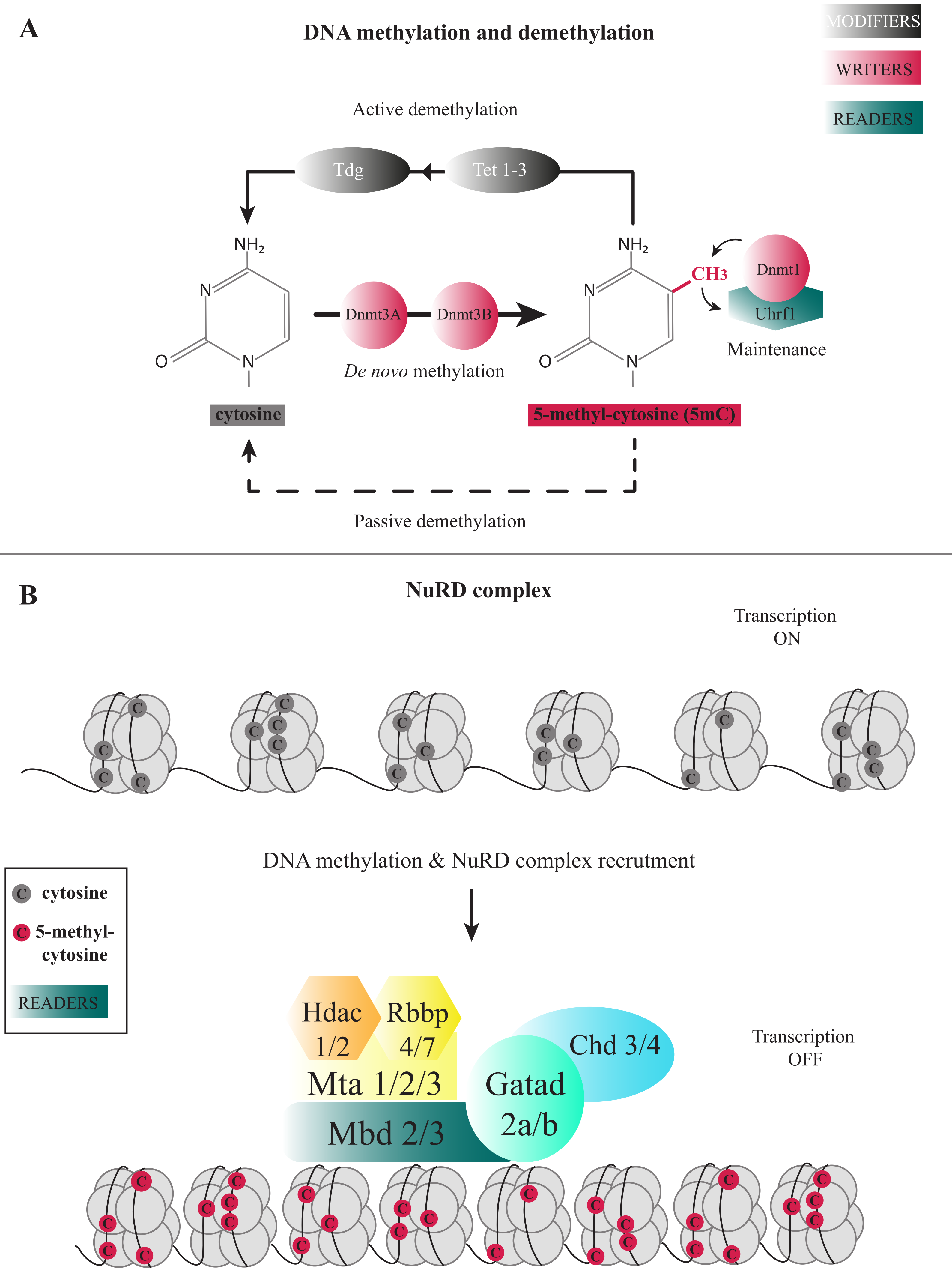
A
DNA methylation and demethylation
MODIFIERS
WRITERS
READERS
Active demethylation
Tdg
Tet 1-3
NH2
N
O
N
NH2
N
O
N
CH3
Dnmt1
Uhrf1
Maintenance
Dnmt3A
Dnmt3B
De novo methylation
cytosine
5-methyl-cytosine (5mC)
Passive demethylation
B
NuRD complex
Transcription ON
DNA methylation & NuRD complex recrutment
cytosine
5-methyl-cytosine
READERS
Hdac 1/2
Rbbp 4/7
Mta 1/2/3
Mbd 2/3
Gatad 2a/b
Chd 3/4
Transcription OFF
