# Supplementary material for: DNA methylation atlas and machinery in the developing and regenerating annelid Platynereis dumerilii
Source: BMC Biol. 2021 Aug 3;19:148. doi: 10.1186/s12915-021-01074-5 (PMC8330077; doi:10.1186/s12915-021-01074-5)
Supplement: Supplementary file 3 — Additional file 3: Figure S2. Additional CpG o/e ratio calculations. Histograms of CpG o/e ratio for several species (whose name is indicated on top) for which this ratio has not been previously calculated. In each histogram, the red line indicates the estimated density, the vertical blue bar shows the estimated mean value, and the shaded blue bar represents bootstrap confidence intervals of 95%. PM = probability mass. Clusters are those defined in Aliaga et al. [48]. The color code for metazoan groups is indicated and is as in Fig. 2. [file 12915_2021_1074_MOESM3_ESM.pdf]

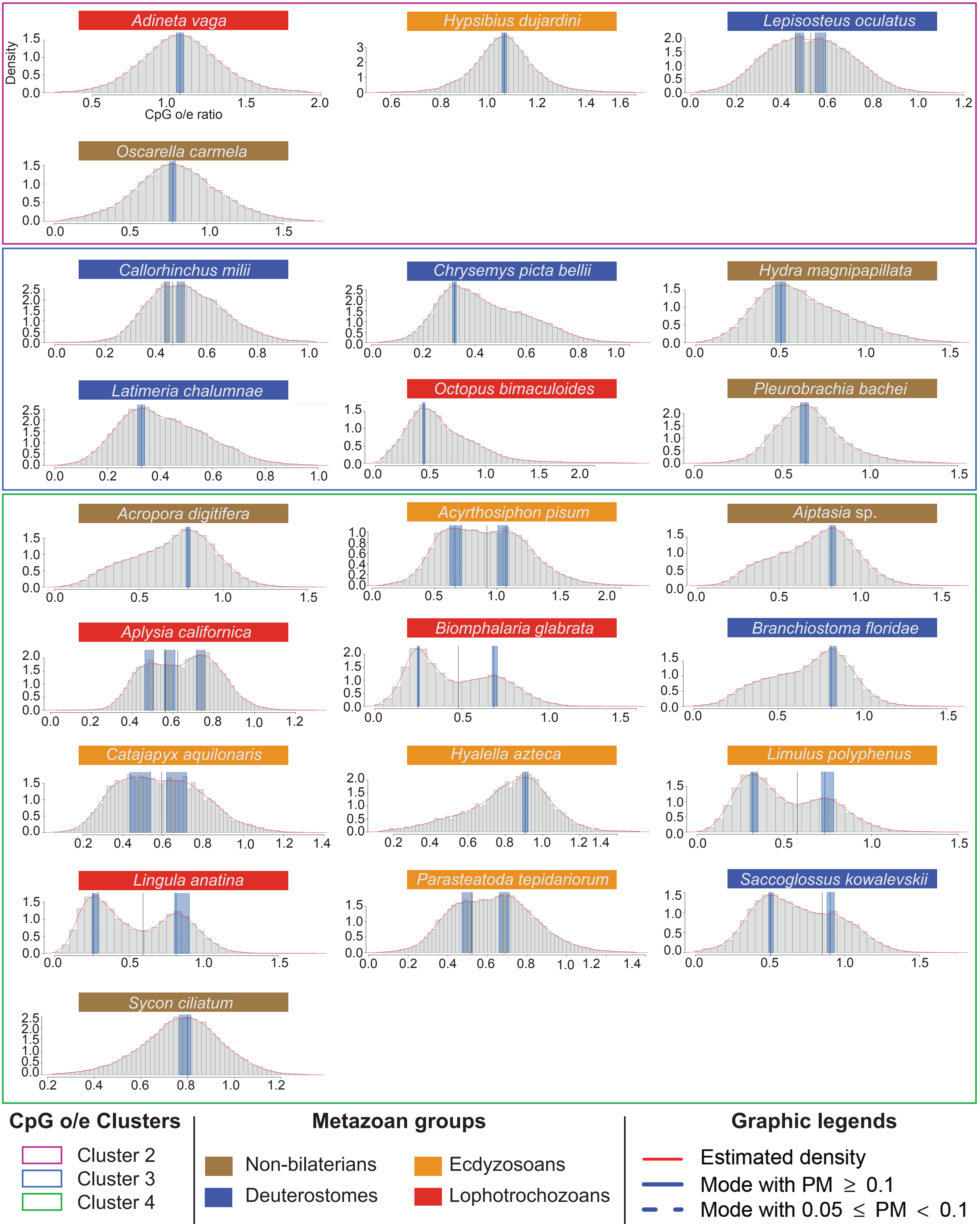

Adineta vaga
Density
CpG o/e ratio
Hypsibius dujardini
Lepisosteus oculatus
Oscarella carmela
Callorhinchus milii
Chrysemys picta bellii
Hydra magnipapillata
Latimeria chalumnae
Octopus bimaculoides
Pleurobrachia bachei
Acropora digitifera
Acyrthosiphon pisum
Aiptasia sp.
Aplysia californica
Biomphalaria glabrata
Branchiostoma floridae
Catajapyx aquilonaris
Hyalella azteca
Limulus polyphenus
Lingula anatina
Parasteatoda tepidariorum
Saccoglossus kowalevskii
Sycon ciliatum
CpG o/e Clusters
Cluster 2
Cluster 3
Cluster 4
Metazoan groups
Non-bilaterians
Ecdyzosoans
Deuterostomes
Lophotrochozoans
Graphic legends
Estimated density
Mode with PM ≥ 0.1
Mode with 0.05 ≤ PM < 0.1
