# Supplementary material for: DNA methylation atlas and machinery in the developing and regenerating annelid Platynereis dumerilii
Source: BMC Biol. 2021 Aug 3;19:148. doi: 10.1186/s12915-021-01074-5 (PMC8330077; doi:10.1186/s12915-021-01074-5)
Supplement: Supplementary file 4 — Additional file 4: Figure S3. P. dumerilii 5mC and NuRD machinery genes and proteins. All identified P. dumerilii genes are listed with the identification of the corresponding gene model in the Pdumbase reference transcriptome [49], excepted for dnmt3, identified in our unpublished regeneration transcriptome. Schematic representations of P. dumerilii (Pdum) and corresponding Human (Hsap) proteins are also shown, highlighting conserved domains found in these proteins and the position of these domains. Sequences of the P. dumerilii and Human proteins can be found in Additional file 5. [file 12915_2021_1074_MOESM4_ESM.pdf]

Families	Protein	Identification in *P. dumerilii* reference transcriptome (Pdumbase)	Protein domains
DNMT	DNMT1	comp223422_c0	*Hsap* DNMT1; *Pdum* DNMT1
	DNMT2	comp216256_c0	*Hsap* DNMT2; *Pdum* DNMT2
	DNMT3	none	*Hsap* DNMT3A; *Hsap* DNMT3B; *Hsap* DNMT3-like; *Pdum* DNMT3
/	TET1/2/3	comp225519_c0	*Hsap* TET1; *Hsap* TET2; *Hsap* TET3; *Pdum* TET1/2/3
/	TDG	comp226234_c1	*Hsap* TDG; *Pdum* TDG
/	UHRF1/2	comp210893_c0	*Hsap* UHRF1; *Hsap* UHRF2; *Pdum* UHRF1/2
MBD	MBD1/2/3	comp219568_c0	*Hsap* MBD1; *Hsap* MBD2; *Hsap* MBD3; *Pdum* MBD1/2/3
	MBD4	comp226112_c0	*Hsap* MBD4; *Hsap* MeCP2; *Pdum* MBD4
CHD	CHD1/2	comp223870_c4	*Hsap* CHD1; *Hsap* CHD2; *Pdum* CHD1/2
	CHD3/4/5A	comp221173_c3	*Hsap* CHD3; *Hsap* CHD4; *Hsap* CHD5; *Pdum* CHD3/4/5A
	CHD3/4/5B	comp216874_c0	*Pdum* CHD3/4/5B
	CHD3/4/5C	comp222390_c1	*Pdum* CHD3/4/5C
	CHD3/4/5D	comp223438_c0	*Pdum* CHD3/4/5D
	CHD6/7/8/9	comp225943_c0	*Hsap* CHD6; *Hsap* CHD7; *Hsap* CHD8; *Hsap* CHD9; *Pdum* CHD6/7/8/9
HDAC class I	HDAC1/2	comp204547_c0	*Hsap* HDAC1; *Hsap* HDAC2; *Pdum* HDAC1/2
	HDAC3	comp212334_c0	*Hsap* HDAC3; *Pdum* HDAC3
	HDAC8	comp217323_c0	*Hsap* HDAC8; *Pdum* HDAC8
/	RBBP4/7	comp223498_c0	*Hsap* RBBP4; *Hsap* RBBP7; *Pdum* RBBP4/7
/	MTA1/2/3	comp223915_c0	*Hsap* MTA1; *Hsap* MTA2; *Hsap* MTA3; *Pdum* MTA1/2/3
/	GATAD2	comp224048_c2	*Hsap* GATAD2-α; *Hsap* GATAD2-β; *Pdum* GATAD2

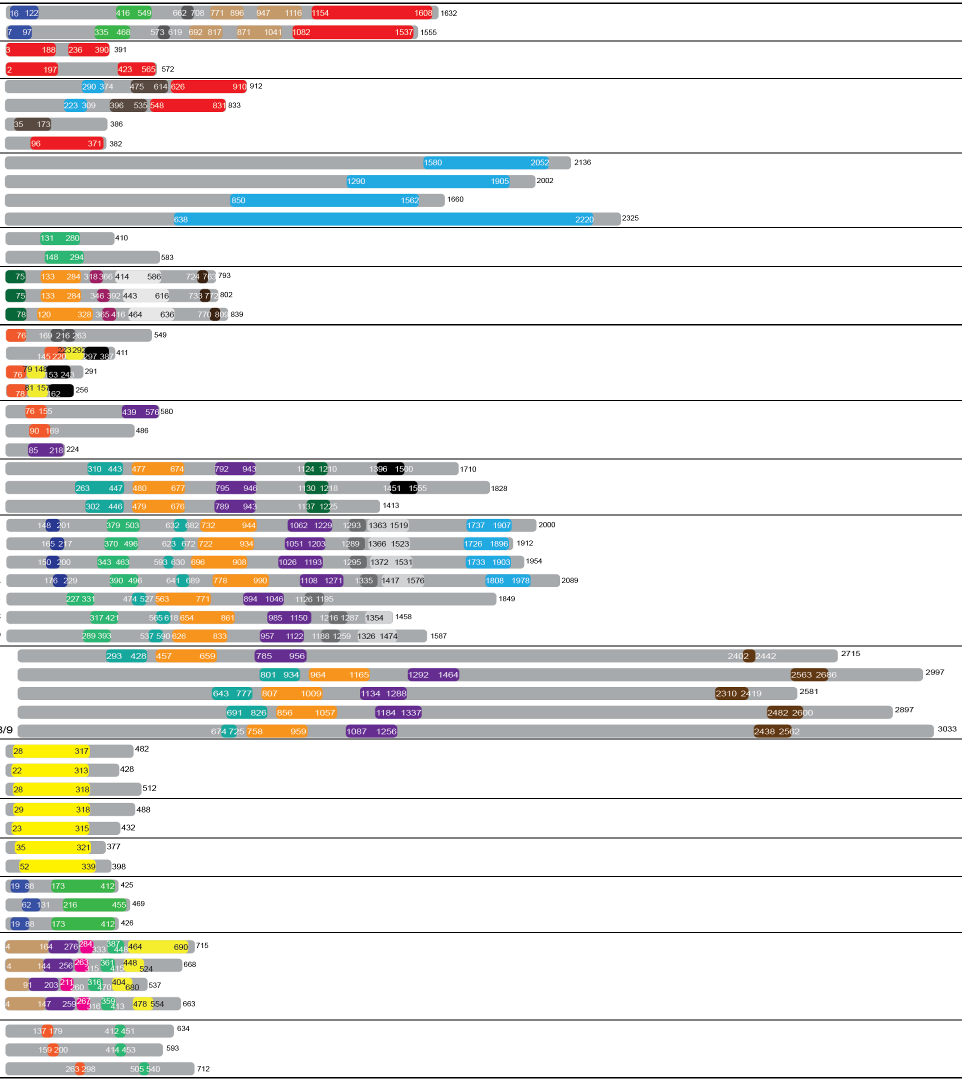

Legend:

- DMAP1-binding domain
- DNA (cytosine-5)-methyltransferase 1, replication foci domain
- Bromo adjacent homology (BAH) domain
- # S-adenosyl-L-methionine-dependent methyltransferase
- PWWP domain
- DNMT3, cysteine rich ADD domain
- 2OGFeDO, oxygenase domain
- Uracil-DNA glycosylase-like
- Ubiquitin domain
- UHRF1, tandem tudor domain
- Zinc finger, PHD-finger
- SRA-YDG
- Zinc finger, RING-type
- Methyl-CpG DNA binding
- Zinc finger, CXXC-type
- Methyl-CpG-binding domain protein 2/3, p55-binding region
- Methyl-CpG binding protein 2/3, C-terminal domain
- # DNA glycosylase
- Chromo domain
- Helicase superfamily 1/2, ATP-binding domain
- BRK domain
- Helicase, C-terminal
- Zinc finger, PHD-finger
- DUF1086
- DUF1087
- CHD, C-terminal 2
- CHD, N-terminal
- CDH1/2, SANT-Helical linker 1
- DUF4208
- HDAC domain
- Histone-binding protein RBBP4, N-terminal
- WD40-repeat-containing domain
- Bromo adjacent homology (BAH) domain
- ELM2 domain
- SANT/Myb domain
- Zinc finger, GATA-type
- Metastasis-associated protein MTA1, R1 domain
- Transcriptional repressor p66, coiled-coil MBD2-interaction domain
- Zinc finger, GATA-type

Superfamily (lack of specific domain in *Hsap* and *Pdum*)
