# Supplementary material for: DNA methylation atlas and machinery in the developing and regenerating annelid Platynereis dumerilii
Source: BMC Biol. 2021 Aug 3;19:148. doi: 10.1186/s12915-021-01074-5 (PMC8330077; doi:10.1186/s12915-021-01074-5)
Supplement: Supplementary file 5 — Additional file 5: Sequences used for phylogenetic analyses, multiple alignments, and phylogenetic trees. This file contains all protein sequences and multiple alignments used for phylogenetic analyses in fasta format, as well as the obtained phylogenetic trees in Newick format. [file 12915_2021_1074_MOESM5_ESM.docx]

**DNMT**

**Protein sequences**

>Pdum_DNMT1_comp223422_c0_seq1

MPVRIDTSPDLSSEVHSRLEELDQELKEDEITEKGYWKRKYSLLEPYLNKSSLEKLKSLKEELKSEEITEKGYQRNLEKILESTVEGLKSEKSENGHVNGKENGTNGHHENGDNGKETEEESSNDVKVKKEPESESMEVDDSQPGSSSSKNGNGSKSEDSQEEGDSQEETDSQDEGGVKKKKGKRKSKSKGGQENGASSGKKTPGRKSKSDSQPSIMSMFSQATNKRKSEGVSQESAQEEKRIKLEEDVKEEVKEEKADTPAEESADVKKEEKKPALKLDKEPPARCKECRQRLDDPDLKMFSGDPADAVEEFIMLTDPKLSLFTGDEADINDGDERPQNKITMFTVYDNNGHMTSFDNGLLEKNKELFFSGYAKPIYDENPSAEDGIATKKMGPINEWWTAGFDGGEKALVGFSTAFSDYILMQPSEAYAPYFNTVTEKIHLTKIVIEFLSEDQDANYEDLLNKIQTTVPPKGISSFSEDSLLRHAQWIVDQVENYDEAADEDETLLITTPCMRALISLAGVTLGKRRAMRKENQKRIPKVKKPAFCMATVSPLVMSLFEKLFEGQIANKSSAPRRQRCGICEVCQQADCGQCRACLDMIKFGGSGRSKQACIYRRCPNIAVKEAEENAGEEEDTNEEAEKLIMDKKEKLKEAAMKRHNFSMQKVTVEWIGEPIKEKNNRKYYSAVNFNSEEVRKGDFLSVKPADNTSPLYIGKVMYLFENKKGDKMFHAHWFNRGSDTILGEVADDHELFMVDDCQDTQLNFCVDKVKVIHKEPGEDWYMQGGKEPENIIEEDDGKTYYYQKWYDVQMARFEDLPEVPEPENESEKLSFCTACFRVKEKELRETPQLFEEVESEKGSALHYGLLTYEGEEYRVGDCVYLDPESFTFKIKPAPAPKIKPDKKREHDDDTYPELYRKGTNIKGSNEFVPSPFCIGQLKDIFIKRAVSGDLEPEQVCLKVRKFYRPENTHKGIMSAYQADLNILYWSDEETTVNLAQVEGKCMVVYGEMLDVSIEEYFKSGANRFYFTEMYDAENKTFSEPPSEATKMSTSKKGKGKGKGKGKGGKGKGDSGDSQDQKKDLPKLRCLDVFSGCGGLSEGFHQSGIAESHWAIEKEEPAAQAYRLNNPSATVFTEDCNELLRLAMQGEKKNSSGQRLPQKGEVELLCGGPPCQGFSGMNRFNSREYSQFKNSLISTYLSYCDFYRPKFFLLENVRNFVSYKKGMVLKLALGALVRMGYQCTFGVLQAGSYGVPQTRRRAIILAAAPGEKLPFYPEPIHTFSPKGMQLTATIDDVKFESCVKHMHSAPFRTISVRDAMSDLPEIRNGAKNDEISYNGDPQSHFQRAIRGHQYQPILRDHICKDMSSLVHSRMQHIPLAPGSDWRDLPNIEVRLADGTRSKKLLYEHKDHKNGRSPSGNLRGVCSCADGKSACDPLDRQFNTLIPWCLPHTGNRHNNWAGLYGRLEWDGFFSTTVTNPEPMGKQGRVLHPEQHRVVSVRECARSQGFPDTYKFFGTILDKHRQVGNAVPPPMSRAIGEEIKKSLQWKQQKGNQKKMETE

>Hsap_NP_001124295.1_dnmt1

MPARTAPARVPTLAVPAISLPDDVRRRLKDLERDSLTEKECVKEKLNLLHEFLQTEIKNQLCDLETKLRKEELSEEGYLAKVKSLLNKDLSLENGAHAYNREVNGRLENGNQARSEARRVGMADANSPPKPLSKPRTPRRSKSDGEAKRSRDPPASASQVTGIRAEPSPSPRITRKSTRQTTITSHFAKGPAKRKPQEESERAKSDESIKEEDKDQDEKRRRVTSRERVARPLPAEEPERAKSGTRTEKEEERDEKEEKRLRSQTKEPTPKQKLKEEPDREARAGVQADEDEDGDEKDEKKHRSQPKDLAAKRRPEEKEPEKVNPQISDEKDEDEKEEKRRKTTPKEPTEKKMARAKTVMNSKTHPPKCIQCGQYLDDPDLKYGQHPPDAVDEPQMLTNEKLSIFDANESGFESYEALPQHKLTCFSVYCKHGHLCPIDTGLIEKNIELFFSGSAKPIYDDDPSLEGGVNGKNLGPINEWWITGFDGGEKALIGFSTSFAEYILMDPSPEYAPIFGLMQEKIYISKIVVEFLQSNSDSTYEDLINKIETTVPPSGLNLNRFTEDSLLRHAQFVVEQVESYDEAGDSDEQPIFLTPCMRDLIKLAGVTLGQRRAQARRQTIRHSTREKDRGPTKATTTKLVYQIFDTFFAEQIEKDDREDKENAFKRRRCGVCEVCQQPECGKCKACKDMVKFGGSGRSKQACQERRCPNMAMKEADDDEEVDDNIPEMPSPKKMHQGKKKKQNKNRISWVGEAVKTDGKKSYYKKVCIDAETLEVGDCVSVIPDDSSKPLYLARVTALWEDSSNGQMFHAHWFCAGTDTVLGATSDPLELFLVDECEDMQLSYIHSKVKVIYKAPSENWAMEGGMDPESLLEGDDGKTYFYQLWYDQDYARFESPPKTQPTEDNKFKFCVSCARLAEMRQKEIPRVLEQLEDLDSRVLYYSATKNGILYRVGDGVYLPPEAFTFNIKLSSPVKRPRKEPVDEDLYPEHYRKYSDYIKGSNLDAPEPYRIGRIKEIFCPKKSNGRPNETDIKIRVNKFYRPENTHKSTPASYHADINLLYWSDEEAVVDFKAVQGRCTVEYGEDLPECVQVYSMGGPNRFYFLEAYNAKSKSFEDPPNHARSPGNKGKGKGKGKGKPKSQACEPSEPEIEIKLPKLRTLDVFSGCGGLSEGFHQAGISDTLWAIEMWDPAAQAFRLNNPGSTVFTEDCNILLKLVMAGETTNSRGQRLPQKGDVEMLCGGPPCQGFSGMNRFNSRTYSKFKNSLVVSFLSYCDYYRPRFFLLENVRNFVSFKRSMVLKLTLRCLVRMGYQCTFGVLQAGQYGVAQTRRRAIILAAAPGEKLPLFPEPLHVFAPRACQLSVVVDDKKFVSNITRLSSGPFRTITVRDTMSDLPEVRNGASALEISYNGEPQSWFQRQLRGAQYQPILRDHICKDMSALVAARMRHIPLAPGSDWRDLPNIEVRLSDGTMARKLRYTHHDRKNGRSSSGALRGVCSCVEAGKACDPAARQFNTLIPWCLPHTGNRHNHWAGLYGRLEWDGFFSTTVTNPEPMGKQGRVLHPEQHRVVSVRECARSQGFPDTYRLFGNILDKHRQVGNAVPPPLAKAIGLEIKLCMLAKARESASAKIKEEEAAKD

>Mmus_NP_001186360.2

MPARTAPARVPALASPAGSLPDHVRRRLKDLERDGLTEKECVREKLNLLHEFLQTEIKSQLCDLETKLHKEELSEEGYLAKVKSLLNKDLSLENGTHTLTQKANGCPANGSRPTWRAEMADSNRSPRSRPKPRGPRRSKSDSDTLSVETSPSSVATRRTTRQTTITAHFTKGPTKRKPKEESEEGNSAESAAEERDQDKKRRVVDTESGAAAAVEKLEEVTAGTQLGPEEPCEQEDDNRSLRRHTRELSLRRKSKEDPDREARPETHLDEDEDGKKDKRSSRPRSQPRDPAAKRRPKEAEPEQVAPETPEDRDEDEREEKRRKTTRKKLESHTVPVQSRSERKAAQSKSVIPKINSPKCPECGQHLDDPNLKYQQHPEDAVDEPQMLTSEKLSIYDSTSTWFDTYEDSPMHRFTSFSVYCSRGHLCPVDTGLIEKNVELYFSGCAKAIHDENPSMEGGINGKNLGPINQWWLSGFDGGEKVLIGFSTAFAEYILMEPSKEYEPIFGLMQEKIYISKIVVEFLQNNPDAVYEDLINKIETTVPPSTINVNRFTEDSLLRHAQFVVSQVESYDEAKDDDETPIFLSPCMRALIHLAGVSLGQRRATRRVMGATKEKDKAPTKATTTKLVYQIFDTFFSEQIEKYDKEDKENAMKRRRCGVCEVCQQPECGKCKACKDMVKFGGTGRSKQACLKRRCPNLAVKEADDDEEADDDVSEMPSPKKLHQGKKKKQNKDRISWLGQPMKIEENRTYYQKVSIDEEMLEVGDCVSVIPDDSSKPLYLARVTALWEDKNGQMMFHAHWFCAGTDTVLGATSDPLELFLVGECENMQLSYIHSKVKVIYKAPSENWAMEGGTDPETTLPGAEDGKTYFFQLWYNQEYARFESPPKTQPTEDNKHKFCLSCIRLAELRQKEMPKVLEQIEEVDGRVYCSSITKNGVVYRLGDSVYLPPEAFTFNIKVASPVKRPKKDPVNETLYPEHYRKYSDYIKGSNLDAPEPYRIGRIKEIHCGKKKGKVNEADIKLRLYKFYRPENTHRSYNGSYHTDINMLYWSDEEAVVNFSDVQGRCTVEYGEDLLESIQDYSQGGPDRFYFLEAYNSKTKNFEDPPNHARSPGNKGKGKGKGKGKGKHQVSEPKEPEAAIKLPKLRTLDVFSGCGGLSEGFHQAGISETLWAIEMWDPAAQAFRLNNPGTTVFTEDCNVLLKLVMAGEVTNSLGQRLPQKGDVEMLCGGPPCQGFSGMNRFNSRTYSKFKNSLVVSFLSYCDYYRPRFFLLENVRNFVSYRRSMVLKLTLRCLVRMGYQCTFGVLQAGQYGVAQTRRRAIILAAAPGEKLPLFPEPLHVFAPRACQLSVVVDDKKFVSNITRLSSGPFRTITVRDTMSDLPEIQNGASNSEIPYNGEPLSWFQRQLRGSHYQPILRDHICKDMSPLVAARMRHIPLFPGSDWRDLPNIQVRLGDGVIAHKLQYTFHDVKNGYSSTGALRGVCSCAEGKACDPESRQFSTLIPWCLPHTGNRHNHWAGLYGRLEWDGFFSTTVTNPEPMGKQGRVLHPEQHRVVSVRECARSQGFPDSYRFFGNILDRHRQVGNAVPPPLAKAIGLEIKLCLLSSARESASAAVKAKEEAATKD

>Aque_15723119

MNLNEESYSIGDCVYLSPHTYSFPNVKKTSGTAANKKQKKEEEEFDESEYPEKYRKVSDYVKGSNIDSPSPFQIGQVLEIFSKSLGGKLIDNNKIVHIKLRMYYRPQDTHKGDEATAQYDLNLLYWSDLVTTVVAGDVVCGKCFVKFKEDITEDIDSYFSNKPNHFYFVEAYCADTKEFEDPPVHAMNKGKGKGVSKGKGKGKGPAKGSKESIATTSTDDDKKDEITPDSSFKKLRMLDVFAGCGGLSEGFHQAGVADSCWAVEIDEPAAQAFRLNNSQTTVFTDDCNILLSLVMEGAKTNSRGQLLPQKGDVELLCGGPPCQGFSGMNRFNSREYSQFKNSLVISYLSFCEYYRPRFFLLENVRNFVSFKKSMVLKLTLRCLVKMGYQCTFGVLQAGQYGVPQTRRRAIILAAAPGEKLPHFPNPTHVFSPRACQLTVVVNDIKYEGSIRMDSAPYRTITVRDSMSDLPHIKNGSAVRSMNYNGEPHCHYQRLMRGNQHQPVLYDHICKEMNPLVAARMRFIPIGPGSDWRDLPNKCIRLSDGTTAPKLQYTHHDKKNGRAKNKSLRGVCPCATGQPCDSSYRQYGTLIPWCLPHTGNRHNHWAGLYGRLEWDGFFSTTVTNPEPMGKQGRVLHPEQHRVVSVRECARSQGFPDTFRFFGTILDKHRQVGNAVPPPLAKAIGLEIKRSVEKKDK

>Aque_15723118

MLGGEEEEELLFQTSSKKKRVPRIIPQKRSKVEEEKEEVSSAIAPTGEEEREEEEGKEKEAKKAKTSETGSSSNGTSAATNGNNKSHDLRPHDDIPANKTSEAPQSSKPPCTYCKRSSDDPRLKIFIGDPPNANDEFITLADPSLSVLSADQESALDDVPQHKITGFSVYDKNHHLCHFDTGLVEKNVELFFSGWVKPIYDENPDPSDGIPTRQLGPINSWWIAGFDGGEKALVGFSTAYAEYILMDASDDYAEIMASVQEKIYLSKILIEYLEEFPAATYEDLLNKIQTSVPPQSIGCTSFTEDSLLRHAQFIVEQVESYDQYCDEDEDLLLVSPCMRALIKLAGVTLGKRLSSRRPHQPKVKKTKQTKATTTPLVRDIFETFFKNQIDNKSSSAPRRRRCGVCETCQQPDCGKCNACADMIKFGGTGRSKQACVNRRCPYMAVQTAEEEEDNDAADPDLKNVKDLKSPSKKIKKIKTKVEWIGEPDFVEGGKSYYTEVLINNKEKVCLYDVVSVCPEVPDDPLYLTRIMSMYEDSNGKKMFHGWWFHRSTDTVLGETGDPRELFLIDDCEDNPLGAIMDKVEVEYKPPVSNWFMCGGEEVSDDEKEIEEDGKTFFVQKYYDQSLARFEDIPSEYIRYLISDDAHPPTDGFIPQCASCERRNHMKTVHSAVPINKIESEKPKITNYR

>Aque_15708686

MQRQLKYYATPIKNYNYKRKRSDKLKFGGPRSKKQACIHRKCLNLIREAAVKSSQPNKRVQSSDSHAPPSKRPLREDGGSGPMLSSPRPCHILPSSQSKTKDNQPVIQLYSPPAAPQSSQPQCTGPYCNRSSNDLTLKTFIGDAPNANDEFIMFADPSLSVFSAESALDDVPQHRITGFSVYDKNHHLYHFDTGLVEKNVELFFSGWVEPIYDENPDPSDGIPMRQLGPINSWWIAGFDGGEKALVGFSTAYAEYILMDASDDYADIMASVQEKIYLSKILIEYLEEFPAVTYEDLLNKIQTSVPPQSIGCTSFTEDSLLRHAQFIVEQVESYDQYCDEDEDFLLVSPCMRALIKLAGVTLGKRLSSRCPHQPKVKKTKQTKAIFETFFKNQIDNKSSSAPHRRRCGVCETCQQPDCGKCNSCADMVKFGGTGRSKQACVNRRCPYMAVQMAEEEEDNDSADPDLKNVKDLKSPSKKIKKIKTKVEWIGKPIFVKGDKSYYTEVLINNKEKVSETGDPLELFLIDDCEDNPLGAIMNKVEVEEKPPVSNWFMCGGEEVPDGEKEIEEDGKTFFVQKYYDQSLARFEDILSEYIRYLIPDDADPPTDGFIPRCASCERRNHMKTVNDT

>Aque_15725529

MAVKHYRPHQSLLRLTVFVNPDGCNEPSFKRFSYQFAKDDKHATVLHYRGDHKVSQKYTNLKKQTCASVRREVEKQEQSPSVVYKKSICKTVPPEYQTVLCPRNPKQVCNLQAKQRQKLRLSHDALYNLHELCYDLETFVQKIVTHPDLIVICGLNSMLKDINRLLSIADSNLHHLLSYDTTFQLQDFYFPVMPVAFLVHERKLRCCHEEMFRVIAKELPSLVNGSQTIPMVTDDEKAFDTVERYLPKVRHLLCWNHIINGAKLWLRQHGVTSREVPVYVSDIHDLLHQPSASEYVTQLDILKQNWSDFFYSQYMNELDKKIKSMCARWNLEEFGLYNPLSGITNNQSEGFNSVLKRMQEWKEIPIDSAVLSLYHLQAFYWNEWQRGLAGLGGYVFALREYQNLYHIAVDKIHLISAISLEDIGRKCTEENNLQLRVDKNTHLLPEEDDHEEIHVPVNESQITSDENGIGSGDSQMTRARMLLERGSLSHDPKLGTFTVKGSSGKLYVVTLYPKQFCSCPATSLCYHIIAVQLSLGMEISQERKKVNLSQLKRNTRNKAHKKSGRKRPTPGFAIHFHFVVFKKHLEDYDIEPAPDSLAKKKPEQPKDSQEDETGLGTLIPKEDSSEEFSYSAISKEVNGTITHACSPKTAPTTRVGKAKRKRCGTCKNCLSTDCGKCKYCLDKPKFGGSGRLRQSCVNKKYQQMLLSKVCNNERKLQVAPNKTEERSGCSVSSPIDVDEIEEMSDHSGGKCEYVATSPFAPIEVSDFATVYKGQWLNDQVINYTLYFIFNEESTERLASDCLFCVPSYVFTKLMAGQADESLNSLLGVYLKKALNSEISVAERDILSSGYLPIARNYPSEKTIEDHDEDNNNTDSLDELLQKESYQVIQKENLLVPTFFNSCDNPDDNKCDIKKVCVQVVSTIMKTIVVPEILTYLIAKKFDLQISSAFNIIKAQTFSCIK

>Ocar_g8727_t1+g6617_t1

MEQEKGAEEKPPEAESTQEASSDPAITGSSEATVKSTKKRKRAVSGSGAKKKAKDGDSPWVVESATKKQTPSEKCKVCGQYLESPDLRLFSGDPEEALDEFAVLAHEQLSLYTGEEEGIGVDEVPQNKITDFSIYDKHFHLCPFDTGLIDKNKELFISGFVKPIYDDNPLPEGGIPAHALGPIGSWWFAGYDGGERGLLGVTTAAASIQDTLFMKGITYYRSVTVDNQEVSINDFVFVQPVNTHVPLYIAQVQYMWQNKRGEKKFHCIWLTRGTETIIGNTADSGELFFADVCSTNPLSSIRSKVEVTYKEVPERWAELGGMEVAEMHYETAQAEERVGEDEDRILYRSFRLFGETFSVNDCSYMMPDAFKFSVRPASSKKTLKRPTDALLRYDEDLYPEMYRRTSDYIKGSAEGVAEPFRIGQIKDIFTKKSHVRKKEEEEEESEEEEESDKEGNSKVFIKITKFYRPENTHLGITASYIANWNHLYWTDEGKCTVVYRDPMAREEVDWEPNTFFFSESYDPTAKSFXXXXXXXRKGKGKGKAKVVASSVTNDPTVPAPVPTTSESKFRPLRSFDVFXXRLPPKRIGRNSMGRRSRRTGSSVISKK

>Scil_17007_1

MLKGQTRSAPSGSAECTAAQLKAAEADFKDGCLTEKGFVKKKWNLLESHLGPAQKKRLSQLQSDFDGGDLGESSFLTTAKEVLGQAEETVFGGATSSKAAEAETEEAPAKLLPTKRTSDASSPEASPAAKRTRVRSRGRAKSSTSTPTRQSSRRRRVVDNSQSGSSSQDDSTVPEADDSVPPAAEVKEALSPIEEKMDTSDSPVVVEKDSKPQVVDSEVTQADTEKPDADTLKPEAADDSDTRSQASQDEGPARCPDCHQLLDSPTLKTYGGDPDNAVDEFVALADPQLCVFEDDGGQEDINETPSHRLTDFTVYDKQGHVCPFDGGLIERNVELFFSGVVKPIYDDTTDVSVGVLTARLGPIDVWWTAGFDGGDSALIGFSTAYADYYLTAPSKEYEHIMSLMVEKIYMSKLVIEFLEENPHESYEDLVDMIQFSTPPKGCNKFSEDTLLRHAQFVVEQVENYDTPDDAQDSYFVLSPCIRALIRLAGVTLGGKRRGIRRPRAPRAPRPKKEKKIASASMAVTTPLVRNIFDVFFQDQIDGKVATSGRRERCNVCEACRQSDCGKCPPCRTMTKFGGDGKSKQCCVQRRCPYMELKAADEDEDLAERKEQDGEDVSDKAKSKQKRPAKQARSDQSVIKTKVQWVGEPAITKGKKKYYKKVLINDEEICVGDCVALCPENPTDPHYIALVAYMWDNEGSKTFHAVWLSRGSESILGEAADPGEVFLVDQCDDNPLGSILFKCALEHRAPSANWRMEGGEEEAAATSGYGDKKFFYQKWYDLEAARFEDPPADMLALLSKSNARPECVACTRQAAEEQRNEPTPGSEIKSEGSTSFYSSFAFEGDTYSVGSGAYFLPTAFSFKYKAPVKKPALPVKKESVDEELYPEYYRKTWGHIKGSNLESPEPFRIARILQIFSQPDVDTLQVRVQKFYRPENAKLSDAERFAADWNILYSSVEECVVSAADIQGSCSILHSQTLTVPLSDYSARSTDNFYFEKEFDGTTKEIADAPKALPESADKTSEKSGKGTKGRKSRTSSSGADFAAALLAESEPTPFEPLRSLDVFAGCGGLSEGFHQSGIAKTVWAIEKDEAAAEAFKLNNKKSIVFTDDCNELLRLVMSGRTENKSGQPLPQKGEVDLLCGGPPCQGFSGMNRFNSREYSQFKNSLVSSYLSYCDYYRPRFFVLENVRNFVSYKQNMVLKLTLRCLLRMGYQCTFGILQAGSYGVPQTRRRAIIMAAAPGEKLPRYPEPTHVFSPRACQLSVMVDDKKYCSNISRAESAPYHTVTVRDAISDLPAVTSGASKQQITYSAESKSPFQRVMRGDKTEGSVGDHICKDMSALVVARMELIPTTPGADWRDLPNKVIKLNGENIPKLRYTHPDRKNGRSSTGAVRGVCSCANGKACDPADRQFNTLVPWCLPHTGNRHNHWAGLYGRLEWDGFFSTTITNPEPMGKQGRVLHPEQNRVVTVRECARSQGFPDWFRFYGNILDRHRQVGNAVPPPMAAAIGREILRSVRQYSAAAAQPVSPQKK*

>Mlei_ML002234a+ML002229a

MVRNVKQTDLRQMFSAKVEKIQKSNEKTNIRTSAVIADEGQSSLTDIASKNVEDNCLAEGEPASKRQKTEKYNREINESTKRKSPALKPVKCDVCKQLLNSEALKVFKGDESGAVEEFIALASDTLSLFDADSNGESEWTEKPQHRITNFTVYDEYGHMCPFDSGLIEKNVPLYFSGYIKPIYDDSPSLEGGVAGSKLGPIDSWYIAGFDGGEKELIGFTTAYADYLLSQPSDQYSPIFDMLREKTYLSKTVIEILVEDSGMTMEDLLNCIQLVVPPENCSKFTEETLLRHAQFLVEQIESYDHAADEDEDLLITIPAVRGLIELAGVTLGGNQQSRARPERPVVKSKAAGKTKSTDISHATVTPLVQSSFEAIFVDQMQATVSFFLSGFSFYRILKFTPTFTSQSKGRKMAVIRAEENAKDETDEPNLAQSNGLKKKLPEVKTEKKVSSNIDGKLTGDPVSKSGGMPVPEKQNEELFFKFWYDSDRARAKISSTKIRKDNVDDEMFPEYYRKTDYIKGNNDLVPELYRIGRIVSFSTKQEGLDGSVVKVTVKKMYRPENIENCRVESADINLLFYSLDQVKIRADKLSGKCTVKNSEDIEDLSRYTTLSDHFYFTESWNSASKCLEDVPSEGRSSNKGKKKRAGIKSEKETQFRKLRTLDVFAGCGGLSAGFHQAGIAESCWAIEFFSEAAQAYKLNNPQAEVFNEDCNAVLRMAMEGVLTNKLGQKIPQKGEVELLCGGPPCQGFSGMNRFNAREYSMFKNSLISSYLSYCEFYRPRYFLLENVRNFVSYKKNMVLKLCLSSLVNMGYQCTFGVLQAGCYGVAQTRRRAFLLAAAPGEVLPQFPEPRHVFSPKAMSLSVTIDNKQYSQHITRFDSAPLRNITVRDIMSDLPAITNGATKRELAYDCEPDSWFQRQIRGNSEVLTDHICKEMNPLVAIRMRHIPLTPGSDWRDLPNISVTLPDGKKTPKLVYTHNDLKNGPMKGVCSCAEGKKCDPMDKQNMTLIPWCLPHTGNRHNNWAGLYGRVFWDGYFSTTITNPEPMGKQGRVLHPEQHRLVSVRECARSQGFPDTYKFYGNLLDKHRQLGCITRSHAFLAWRNSKNSKRASPVDATAVKSDTVAVKRTIKSPSSNVESDTHKLTTSPKKTLTNKSSSSSASKLPKSSSKSVDLNSQSHSLSILSGKRTGSKSKLSERFYNVECFSNGSSTLGSRHYGSYSSSGHSRLASS

>Nvec_v1g125496

PARCKECRQLIDSPDLCLFAGDPADAVEEFVALVDPRLSLFSGDEEQFDSYEDKPQHKITNFSVYDKNTHLCAFDTGLIEKNVELFISGYVKPIYDENPSPEGGVPTKILGPINEWWTAGFDGGENALIGLTTAYGEYFLMNPSKEYAPFIHTMREKIQLSKIVIEFLLNNPEARYEDLLNKVQVSVPPEGCPSFTEDSLLRHAQFLVEQIENYDSAGDTDELPLIVTPCIRDFIKLAGVTLGKRRTARKIKVKSRAELKKKQAPTKATTTELVRHIFDIFFQNQIDGKNGNAPRRKRCGVCETCQQPNCGKCSACRDMVKFGGTGKSKQCCINRRCPNMMVREADDDEGLEDEEKDDNVRDVLIRILNKSSYKNLEWLGEPAWIEGKKKYYSSVLINKKKVSLGDFVKVCPDDPHIPLYIGCVQYMWESSSGEKLFHARWMTRGAETVLGETADSAELFLCDDCDDNPLGSVLETCEVTYKDPSVDWLQGSAGMEIETSDDEHNDNTFFYHKWYHPEMGRFVDPPPEYQTKVDRTQQKFCESCIRNSAEEKVITPDISLFSRKIVTSFTLNGSEFTVSDCVYLDPEAFSFNVRPKESKPHKREGSVDEDLYPEYYRKSSEYVKGSNQNVPEPLRIGRIKSIYVKSSSGKLKGDGDDVMLTVTKFYRPENTHKGANASHQADLNLLYWTDEEAKVSALSVKGKCMVACGEDIHSSIQEYSSKGDCFYYLEAYNSQTKEFEELPPQARDCSSKGKGKGKGKGKGKGKSSADSGPESCMHDQQKEAHVRKLRSLDVFAGCGGLSEGLHQAGVAESLWAIEKEEPAAQAYRLNNPGCTVFTDDCNTLLKLAMEGEATNSTGQKIPQRGEVELLCGGPPCQGFSGMNRFNTREYSLFKNSLVVSYLSYCDFYRPRFFILENVRNFVSFKKSMVLKLTLRCLLRMGYQCTFGVLQAGCYGVPQTRRRAIIMAAAPGEELPLYPEPTHCFSPRTCQLTVMVDEKKFTSNITRTASAPYRTITVRDAMSDLPEIRNGASAAESSYEGEAISHFQRQIRGNQYQPLLRDHICKEMSALVEARMRHIPLAPGSDWRDLPNKEIRLSDGTYSKKLQYTHHDKKNEKGSSQRLRGVCSCAEGRPCEPADRQFNTLIPWCLPHTGNRHNHWAGLYGRLEWDGYFSTTITNPEPMGKQGRVLHPEQHRVVSVRECARSQGFPDTYRFYGSILDKHRQVGNAVPPPLAAAIGREIKKGLELTQGVRKKEESKMDITS

>Aipt_28556+28558

MVKLSTLPDGIADSLESLEKEFEDELLTKKGYIKKKYLLMKAYIPQYAIESIESLEKKSKNSSIEEESYLNQLDEILNEFQPSKTVQKGQGSSSSCSDHEMRDDSEQHVTNGSNGYAESLQNEDTVQEDKHGEDKNRNGNFEENGDTEEETPLNGSSCETEIKTTTRKKRKEQEKSGKKAKTSPHRQPGIAEIFSKVPCKRKGPGNGDDQESASSSGMSGADVDTAETQQEKKIKLDDNGDEKKGSAEPDSSESKRKKITNSKVQIPPRCNECRQLLNNSELRMFSGDGEDAVEEFIALVDPKLSLFTGEEEQFGSYDDKPQHKLTNFSVYDKNTHLCPFDTGLVEKNVELYVSGYVKPVYDENHVLEGGVPTKNMGPINEWWTAGFDGGEKVLIGFTTAFGEYILMQPSEEYKPFMEAMAEKALLSKIVIELLIENADASYEELLYKVQTAIPSDNCTEFTEESLLRHSQFIVEQVENFDEARDSDELPLLISPCMRDFIKLSGVTLGKKRGARKVKIKTEVKKRGPTKATTTPLVRHIFDIFFKNQIEGKGVSAPRRTRCGICEKCQQPDCGKCKYCRDMVKFGGTGRQKQSCLERRCPYRDIKEAEDNETLEEYDKDSELDNRKGANKVHHKIAQDQKIVKTKVKWIGEPVRETNTRRYYSSALINKEEVTVGDFVQVCPYDPHTPLYIACVSYMWENSKGKKMFHARWMTRASDSVLGESADPRELFLADDCDDNPLGSILRKCTVSIIFMKLFILFQSCFSMVRCSLLCRQIHRSILGFPVYNMGECRGG*MVSAALLHILQLTFSWVVLVLCDVYILVDVTYRDPLKHGNKLTTEEEAANDDQHDENHFFYQKWYNSDMGRFTDPPIEYTVSVERAKTNYCEICTRNDAQEKFDTAFVKGEIEDSGSKTGYKTLAYKGITYNIGDYVYLEDGAFTFNVKQKPLVKPTVKSEGTVDEELYPEYYRKSSDYVKGSNQDVPAPYQIGRIVKILTKNVSGKLKSDEEVFLNVTKLYRXEDTHKGPSAGHQADSNLLYWTDEECSVSVSHLRGKCTVVYGEDIDETVETYTANGPDRFYFFEAYDSKRKEFHELSIEAREQLMSKKGKGKEGKGKGKGKGKMAATVKESDDKSKKEEVKKLKSLDVFAGCGGLSAGLHQAGVAESLWAIEKEVPAAQAFRLNNXDCTVFTDDCNELLKLAMDGKETNSKGQKLPLRGEVELLCGGPPCQGFSGMNRFNTREYSLFKNSLXVSYLSYCDFYRPRFFILENVRNFVSFKRSMVLKLTLRCLLQMGYQCTFGVLQAGSYGVPQTRRRAIIIAAAPGEVLPLYPEPTHCFHPRACQLTVKVDDKVFRSNXTRTSSAPYRTVTVRDAMADLPGIDNGASACEISYDGEAISHFQRQIRDNQYQPMLRDHICKEMSALVAARMRHIPLAPGSDWRDLPNIEVRLPDGFSKKLCYTHHDKKNGKGSNGQLRGVCSCAEGQPCDPADRQFNTLVPWCLPHTGNRHNNWAGLYGRLEWDGYFSTTITNPEPMGKQVATNLCCGIPVD*

>Hmag_2.221116+2.221115

MLVDPRLSLLSGNEQDFDAYEDRPQHKVTEFSVYDKCGHLCAFDTGLIEKNVELFFSGYVKPIFDENPDIEGGISTKAMGPINEWWIAGFDGGENALIGFGTAFAEYILMRPSDDYASFMNAVTEKIYMSKAASNHYXMYMSQLIFLFWIVIEFLLNNPDSEYEELLNKLETTVPPENCAKFTEDTLLQHAQFLVEQVESYDSAALDEEDSPRLITSCCMRDLIKLAGVTLGKRRQMRGLKVKEEKKTLGPTLATTTPLVRHVFDTFFKNQIDLKGVTTQRRKRCGVCEICQQPDCGVCRSCKDMVKFGGSGRSKQCCINRRCPNLAVQEAEEDALCSQDEDEPMLKSPSKTDLSPRHHRKGQEKKSFVKWACEDFVERKGKKLYKSVQINNELINIGEFVQVYPTDPSDPLYICRVMYMWEDLNGDKKFHAQWLYRSSETVLGEVGDPSEVFLSDDCDDIKLGAIMSKCNVSSKFASENWFMEGGKEDCIISEENNELFYQKWYDYEDGLFTDPPTEFLLSKFDEKYCPSCERTKTKLKYKQPTLGDELETENSTKSEHCFKIKQKSELPKNKSVDEDEYPELYRKSNDYIKGSNINIVEPFRIGKIISIIKKTDNYGGMKSIMLKVRKFYRPENTHKGMSGTMNCDLNMVYWSNEEATVDFQMVEGKCYVLFADDADMDMFKFTEAGPDRFYFREAYDADKKEFDVPPREAWNSNKGKGKGKSKSTKQQNGLESNFPAYEKVEKLRTLDIFAGCGGLSEGLDQVGVVNSCWAIEFEPSAAQAYRLNNPSAIVFNQDCNNVLKQIMEGKEKDDLGQRLPRRGEVDLLCGGPPCQGFSGMNRFNQREYSMFKNSLVTSYLSYCDYFRPKFFILENVRNFVSFKKSMVLKLTLSCLVKMGYQCEFGVLQAGSYGVPQTRRRAIIIAAAPGEILPKFPEPQHVFASKALSLSVTINSHKYQALKRLHSAPYRTTTVYDAMSDLPEIKNGANKMEIGYDTEPLTHFQKKIRGKHMQVLRDHICKDMSPLVEARMSYIPCIPGSDWRDLPNKFNTLIPWCLPHTGNRHNNWAGLYGRLEWDGFFSTTVTNPEPMGKQGRVLHPEQHRVVSVRECSRSQGFPDSFRFYGNILDKHRQIGNAVAPPMSAAIGQEIRKSIIAKKLRNEQLQSIKEENNHIATFX

>Cgig_EKC23761

QHKITNFSVYDKNTHLCPFDTGLIEKNVFLYFSGVVKPIYDENSSPEGGIRACKMGPINEWWTAGFDGGENALIGFSTAYAEYILMSPSEAYKPYMDTMREKIHMSKVVIEFMQNNQEATYEDLLNKIQTTVPPTGLSSLTEDSLLRHAQFVLDQVQSYDEAAEEDEGLLITTPCMRALIKLAGVTLGKRRQMRKELRKTKDKVKKPAFTMATTTRLVTQIFDSLFQGEIDDKSGQGSKRRRCGICEICQQPDCGKCTACKDMVKFGGSGKAKQACINRRCPNMAMKEADEDDILDDDDTDEKLETTKLSWVGDPVLQDGKNSYYSAVLINDEKVSFGDFISIKPEDVAIPVYIAMVNYLWENASGNKMCHVQWLCRGSDTILGETGDPLELFFVDDCESIKLESSLRKVKVLHKETSPDWFMQGGIEHPEKDFPIEDDSNTFYYQKWYDPDLARFEDIPTSDKPKDQRHHKFCLTCDRLDQLRKKENPSLGPEIESEDSSATRTFYSSVGRDGFEYKVGDCCYMEPSAFGFNVKQPPAKKVKTERKEVDEELYPEGYRKGDYIKGSNESVPEPFRVGRILAIFCKKSSGSKIANLGEIKVKVAKFYRPEDTHKGPSAGYHTDMNMLYWSDEEATVDFKLVKGKCQVTYSENLRSLEEYLCGGLDRFYFTEAYDSETKMFEEPPRQARVSGGKGKGKGKGKGKSSGPSAEEETTEPKKPTYRRLRTLDVFAGCGGISEGFHQAGIAESCWAIEKEEPAGQAFRLNNPGCTVFTDDCNVLLKLVMEGEKQNEMGQKLPQKGDVELLCGGPPCQGFSGMNRFNSREYSRFKNSLIASYLSYCDYYRPRFFLLENVRNFVSFKRSMVLKLALRCLTTMGYQCTFGVLQAGSYGVPQTRRRAIILAAAPGEKLPFFPEPQHVFAPRAMQLSVQVDDKKFMSNITRMESAPFRTITVRDTMSDLPEIRNGAKAEEISYQGDPQSHFQRIRLSGHWAVK

>Lgig_171288

MHKLTNFSVYDKQTHLCAFDTGLVEKNVHLMFSGVIKPIYDENSSPEGGVSATQMGPINEWWTAGFDGGEKVLVGIGSAYADYILMEPSEAYADIWNALQEKVNMTKVVIEFLSTNFDATYEDLLNKIHTTVPPKGIASFSEDSLLRHAQFVVDQVQSYDEAADFDEELIMTTPCMRALIKLAGVTLGRRKAMGRQLKPTKEKVKPTQSKATTTALVESIFDSMFNGQIDDKTGNGGVKRRRCGVCAVCQQPDCGTCTSCMDMIKFGGSGRSKQACKLRRCPNMALKEAEEDAALEDDADDDKENEVINEKESAHHALIHPHKGKRVVKEIKWVGEEIAIEGRKKYFKAVDIDGVEIAIGDHVAVKPEDSSIPFYIAQVTSMWESSSKEKLFHADWFCRGSDTVLGEASDPLELFLVDDCEDSNLEYVLDKVTIIYKEPAKDWRMLGGIMDPDSDNVIEKDDGKTFFYQKWYNPDYVRFEDPPKKPDVPQNQSHKFCISCNRLEELRKKETPVVGDEVDGSEESSNKVFYGYVQRDGFQYNVGDCCYIDPEAFDFNVKPSNKKINISKRKVDELLYPEAYRKSSDYIKGSNNECPDPFRIGQIVGIFCKNSSKSKIANVAEIRLKIRKFYRPENTHGKCKVVFSENLLMDTTEYFNKVEDGFYFTEMYDSEKREFEEPPSSARMMGGKGKGKGKGKGKGKGKAREEPTEESKEVKFEKLQMLDVFAGCGGLSEGFHQAGIAESKWAIEIVEPAAQAFRLNNPGSTVFSQDCNFLLRRAMDGEKTDELGQHLPQKGQVDLLCGGPPCQGFSGMNRFNSREYSKFKNSLIASYLSFCDFYRPRFFLLENVRNFVSFKRSMVLKLALRCLIRMGYQCTFGILQAGCYGVSQTRRRAIILAAAPGEKLPFYPEPLYTFAPRAMQLSVCIDEKKYTSNITRTTSAPYRTVTVRDCMSDLPEIRNGAKAEEISYKDDPQTHYQKMIRGNQHQPILRDHVCKDMSALVHARMQYIPLAPGSDWRDLPNIEARLSDGTKSNKLKYTHHDKKNGRGVDGELRGVCSCSEGKSCDSEHRQFNTLIPWCLPHTGNRHNHWAGLYGRLAWDGFFSTTVTNPEPMGKQGRVLHPEQHRVVSVRECARSQGFPDTYRYFGNILDKHRQVGNAVPPPMSKEIGREIKKCLIMKAKEKDAEEKESKMDVEADEKKEEIKEEVKAEGSSS*

>Bgla_5185

MGPINEWFVSGFDGGENALIGFSTGFAEYILMQPSEAYAPFIDQMKVKVHMSKIVIEFLSNNPDALYEDLLNKIQTTVPPAGLTSFSEDSLLRHAQFIVDQVQSYDDAAENDEFLLITTPCMRALIKLAGVTLGKRFETLPSSICQQPDCGKCTACKDMTKFGGTGRAKQACKERRCPNMAVKSAEDDDLEEETEIDKEIEEEKCISTAPKHKAAKNIKTQVQWIGDSINEKGKRMYYSKAMLNEFEIAPGDYVSVTPSDPTEPYFIAQIQYLYEDASGKYAHINWLYRGSETVLGEASDPLELFISNDCNDNDLVTIHNKVTVIQKTVPIDWNMKGGIDNPDEDSVVKEDDGKSFYYQKFYDPEFGRFEDPPVLKCAEGQIVCSCCERVELHKKLMETSLGTKAEVDNAQSGYTYYSSVSKDGDTYKVGDCCYLDPEAFIFSIKHPKVAPRTTKDKHIDEDIYPEAYRKSEYVKGSNDDCPEPFRVARIVEIFVKQDKNKQSDVQDVKIKVNKFYRIENTHKGQQAVYHSDLNKLYWSDEQVTVTISYIRGKCTVMYQENLLESLDDYFLGTSDRFYFNESYNSQSREFEDPPRIARLMGSKGKGKGKGKGKSSDKQDTQTEESKKVEIHKLRTLDVFAGCGGLSEGLHQAGMAETCWAIEKEQPAAQAFRLNNPGCVVFTDDCNLLLQRVMQGEKTNELGQKLPQKGDVELLCGGPPCQGFSGMNRFNSRDYSKFKNSLIASYLSYCDYFRPRFFLLENVRNFVSFKRSMVLKLTLRSLLRMGYQCTFGVLQAGSYGVAQTRRRAIILAAAPGEKLPMFPEPMHTFAPRAMQLSVMVDEKKFVSNIKNTSSTPYRTITVRDAMSDLPEVKNGAKAEEISYKGDPETHFQRLIRGNQHQPILRDHICKEMNPLVAARMMHIPLAPGSDWRDLPNLELRLSDGNKTKKLLYPYKDKRNGNGSNGQLRGVCTCATGKACEPLDRQFNTLIPWCLPHTGNRHNHWAGLYGRLDWDGFFSTTITNPEPMGKQGRVLHPEQHRVVSVRECARSQGFPDTYRFFGNILDKHRQVDKLE

>Acal_XP_005095276.1

MPGSLATSLAAQRIILTDDSLQRLQSIEKDYNEGDITLKGYWKQKCSALESYFTDNVREQMRSLADDLENEEITEKGYYKKLEKVVTDFLKEVENDGSGMINGSASSRQNGHTGDLKSPGPEPSHTNGHTNGTSATNGTGSVNGTSATNGTGSVNGTSATNGTGSVNGTSATNGTGSVNGTSTVMNGSSAAKKLSKFAAGSSQSETMDEKDALKTESNGHTNGNKSMNGHASPKIKEVDTDVKQSSDSPASEEVMEVDSVEENGVDVNCSPPHKKSKQDKSNESFDSPKQEQTPRRSSRRVSKKSDEMQPSITAMFSPKAKVEEKASSKDKKENSAENGEEEEEEQQASKRIKLQDDEGKQEGIVPVLEPSAPKPLPQRCNECLKYMDDPELKMFPGDADDAVEEFIALTDPKLSLFTGEEDFDKIQEDRPQHKITNFSVYDKNTHLCAFDTGLIERNKYLFFSGVLKPIFEENPSLEGGIPCRNMGPINEWFVSGFDGGENALIGFSTAYAEYILMQPSEAYTPFIEQMKVKVHMSKIVIEFLTNNQDSTYEDLLNKIQTTVPPAGLTSFSEDSLLRHAQFVVDQVQSYDEAAADDEPLLIVTPCMRALIKLAGVTLGKRRQLRKDVGRVKAKKEKPKHTMATVTPLVRNIFDSLFQGQIDEESSKTKKQRCGICEVCQQPDCGKCAACRDMVKFGGTGRAKQACKERRCPNMAIKTADEDEEEDTEIDKVSEENAAVVKKHKAAKNAKAKVTWVGAPVKEEGKRKYFATAKVNNVEITAGDNVSITPADPTQPLYMARVQYLFEERGSMQAHVHWYFRGSETVLGEASDPLELFLVDECDDNVLEAVHAKVEVLFKSIPSDWSMLGGIDDPDVDNVVQEDDGKTFFYQKFYDPTLGRFEDLPMYEQCSESSKVDHCVSCYRLEQRRLLEEVAVGEEAESSSKTASYLYYNSVRRDGLEYHVGDCCYINPEGFDFLIKHPAKSGKQLRLKDKNMDEDMYPESYRKTEYVKGSNDGCPEPFRIGRIVQISAKKMSGSKLPDLAEIKIKVNKFYRVENTHKGQQAVYHSELNKLYWSEEEASVHFSQVCGKCTVLYQDNIAGDLDTFFLDSPDRFFFLESYNAESRSFEDPPAKARLMGSKGKGKGKGKGKGKSSKAEPSKEASEEDKVEIHKLRTLDVFAGCGGLSEGFHQAGLAESRWAIEKEEPAAQAFRLNNPGCTVFTDDCNLLLQRVMLGETTDELGQKLPQKGEVELLCGGPPCQGFSGMNRFNSREYSKFKNSLIASYLSYCDYYRPRFFLLENVRNFVSFKRSMVLKLALRCLVRMGYQCTFGVLQAGSYGVAQTRRRAIILAAAPGEKLPVYPEPQHTFAPRAMQLSVMVDDRKFNSNIKNMTSTPYRTITVRDAMSDLPEIKNGAKAEEISYKGDAQTHFQRLIRGHQHQPILRDHVCKEMSPLVAARMTHIPLVPGSDWRDLPNLEIRLSDGSKAKKLLYTHPDKRNGKGSNGQRRGVCACATGRVCDPLDRQFNTLIPWCLPHTGNRHNHWAGLYGRLEWDGFFSTTVTNPEPMGKQGRVLHPEQHRVVSVRECARSQGFPDTYRFFGNILDKHRQIGNAVPPPMGRAIGLEIKKCLVWKQKNGPEEEKTGSEVEKAKEEEKTEEKDLPESSQEKEEEEKKPTSNGSGGSEEVEDMESESKQESGSKGADSGTAGAGASSSSALA

>Obim_22034501m

MPSRTSSISKTTISSPKLRESNKIVKDLQNKFQTGDITEVDYWNQLCEKFSHVLTGAAKDQIQTLETELGEELLTIKGFCKKMEDILQPLVSNTLPKVPSLKPVLVKPEVVTKDDTTPASKRSVENKNGINDSAENDEEVEPGLSPSKMGKMDEKEHNCEKKESTSVATDAITNNVQSSPPTTRRNGQSSNGRNENGVENGLNFTNGHSTEDGVKFPEAVPSSEPTGNDKNPPSTPESRKSKSKRTRSSGIQPAITSMFERIPEKRKAADEDEESSGVKQEEKKIKTSEINDKDDENKEASSVQNTDTKLAKEQVTPIKCRDCKQLLDDPELVLFHGDPPDAVEEFIALTDPKLSVFSGNDDEVSSFDERPQHKLSNFSIYDKATHLCAFDTGLIEKNVELYFSGVVKPIYDENPSADGGVPCKTMGPINEWWTAGFDGGENSLIGFTTAYADYILMSPSKIYSPFMDIMREKIYLSKIAIEFLQSNLDATYEDLLNKIQITVPPNGITNIPTEDALLRHAQFVVDQVQSYDEAADDDEALLITTPCMRALIKLSGVTLGKRRALRKQLREPKVKSKDTQTMAATTSLVRNIFDSMFQGQIDDNGFSATRRKRCGICEVCQQPDCGKCKACRDMVKFGGSGRSKQCCVQRRCPNIAVAEADEDEIQSDDDENIVKKGDDSEKPIRHVASRNTKTKLEWVGEPVKTTPTNIYYNAVNINGKEVCVGDCVAVKPANPTVPMYIAKVAYMWEDKFKEKKFHALWFCRGSDTILGETADPFELFLVDDCEDSCLEYTISKVNVVYKSIPSNWAMLGGTDEDTNFNNNDSKTFYYQKWYDPELARFEDIPKDPNPSEINSYKFCSSCSRLETLRKKETPCLGEQLENGSKESSRLYYKSVKRDSIEYKLGDCCYVEPSAFSFNVKHVPQKKPKTEKREFDDDAYTEAYRKSSDYIKGSNEYVPQPFHIGRITEIFCRKVNNSRLMDLTDIKIKINKFYRPENTHKGPAASYQADLNVLYWSNEEVTVDFKTVSGKCTVVYSENLKVDKDTYFMGAVDRFYFTEAYNREKRSFEDPPNVACLMGDKGKGKGKGKTKSKSKCLPDEIEPTVTPLAALDVFAGCGGLSEGLYQAGIAKSHWAIEKMEMAAQAFRLNNAGCTVFTDDCNLLLQLAMKGIEKTSKGQKLPKKGEVELLCGGPPCQGFSGMNRFNYRDYSKFKNSLIASYLSYCDFYRPRFFVLENVRNFVSFKKSMVLKLTLRCLTQMGYQCTFGILQAGNYGVAQTRRRAIILAAAPGEKLPFYPDPLHVFAPRGMQLSVVVDDKKYNSTITRTGSAPFRTITVRDTMSDLPEIRNGAKEEEIQYNGEPYSHFQRLIRGKQHQPILRDHICKEMSPLVHARMQHIPLCPGADWRDLPNVVVKLSDGNKTKKLHYSHHDKRNGKGPNNQLRGVCACALDGKCDPMDRQFNTLIPWCLPHTGNRHNHWAGLYGRVEWDGFFSTTVTNPEPMGKQGRVLHSEQHRVVSVRECARSQGFPDTYRFFGSILDKHRQVGNAVPPPMSRQIGFEIKKSLLWKLQNSAEQKPEDQSKREN*

>Ctel_160905

MVVTAVKVAPLKCKECRQLIDDPDLKLYPGDSSEAVEEFIALTDPKLQLFTGDEEDVNAADERPQHKITNFTVYDKNMHVCPFDCGLIEKNTELYFSGSLKPIYDENPSPEDGILTTKLGPINEWYTAGFDGGESALIGFTTAFAEYILMTPSDLYAPTMTVVQEKIYLSKIVIEFLSDDTEATYEDLLNKISTTVPPQGMTSGFTEDSLLRHAQWIVDQVESYDNAGEDEDILLIATPCMRALIKLAGVTLGKRRVISRQGKSRVLKKQKPTFTMATTTSLVKSIFDSIFQDQIEDKLAKVKAQRCGICEVCQAPDCGTCKNCKDMVKFGGSGRSKQACQQRRCPNMAVKDADDNDALDDVDKENVMKPEKQPKGEAKSRMLKFKKQKSQLEWEGEPVLKKSKKTFYASARLNGEILSVGDYVSVKPDDPSTPVYIAQIKYFYEDSDGTMMFHAQWYSRGSDTVLGEASDPKELFVVDECQNTQLAFCVEAVKVHYLTPADNWQMIGGDDRGMNLDDQEEGCAFFFQKWLYDEDLARFEDVPIVENVEKDVTFCSSCVRLAEKKKKESPLLGEKIDSSKSSRCSFSSVNYWGESYSVGDCVYVDPEAYGFHIKPVNHKKSKHEKPCDDELYPEAYRKGDYVKGSNLQNPDPFRIAKIQEIYVKSGVSTAVLYAQYLCRPENTHKGSHGTSHTDLNMLYWSDEDGKAALSQIWGKCQVVHWDDLQVPLEDYFAAGDDRFYFSEMYNTNTKEFEEPPSECRSQVSKGKGKGKGKGKGKGKSSEETPREPEPTEANAQPITRLRGLDVFAGCGGLSEGFHQAGIAESCWAIEKEEPAAQSFRLNNPGATVFTDDCNLLLKAVMEGQTVNDVGQKLPQKGEVELLCGGPPCQGFSGMNRFNSREYSMFKNSLIASYLSYCDYYRPKFFLLENVRNFVSFKRSMVLKLALRCLLRMGYQCTFGVLQAGNYGVPQTRRRAIILAAAPGEKLPFYPEPLHVFAPRGMQLSVQVGDKRYNSCVQRMSSAPFRTISVRDAMSDLPEVKNGAKAEKISYSVEAQSHFQRMIRGHQHQPVCRDHICKDFNPLVHARMQHIPLYPGADWRDLPNIEVRLSDGSKCKKLCYTHRDKKNGRSPSGALRGVCQCADSGSCDAMDRQFNTLIPWCLPHTGNRHNHWAGLYGRLEWDGFFSTTVTNPEPMGKQGRVLHPEQHRVVSVRECARSQGFPDTYRFFGNIQDRHRQVGNAVPPPMAKAIGSEIKKCVLWKMEQDAKKEREKKKQVDKEEGKKQDEKEKKDGDEEMKGDEEERKGDEEERKGDEEEMKGDEEERKGDEEEERMES*

>Ctel_222429

MQEHSEWLAEQVISYDEGRDEDDLQLALCPAFRTFLRVTGITPKSKKLGKKTKPRQSLVIVKKEKMVATTTAAVRQLFSKDFKSLFGAATMSGDLEVIISDDEGEEDGDAPPRSAQLSRNPRGHKNRMLAGEVEWGPLLREDHCSSHYESATVDNVKISPGDYVQIEHDDSAKEPHLGRVISLFERQDSPEPQVHIHWLHYTSTTLLRETGKDNELYFSYDECQDVDLSSVMCRVNVTEVSQLTVNDSHVHYGMSDERLYCVEYVYNDTTGAFTSPAKPKKFTCPSCAVLKARRLKESPSISDGILQYWQNEYSIGCYMFVQTENKENSATLQDKEKVVANSKFPEQWRRQNDSGEPPRVAPLTIGRLLGIHWADDDDAHEVVLTLQRFFRPEETSLEKPWLKDIRLVFPSDQIFEARLDTTWGRCNVINAREYLDSLDEFLKESDNNFYYEKSYLHAEGLFIDFELDAKLLLRRKPIKREMRSKRSTRKRAKLMRFEDNPISIDDDDLIVVMSSDEEELASCQERSISWSSSDSSSDSSSYSVPSPIVSLPNVEAKLRSLDLFSGCGGLSLGLSQAGVSEACWAVERDPVIAEAFKRNFPKCCVYAADCNAMLGSILAGESCDPSGQVYPLKGEVDLISAGPPCQGYSLLNKFTSSEAYQFKNSLVVTALSFCDYYQPKYFVMENVSTFATHKGGNLLKLVIRCLLELGYQCRFGILQAGSYGVPQSRRRFILMAAAQGLLLPAFPEPIHAFPVAFCDVYIDKRKFKPGLERIKIGHLRPLQIRDAFDDLPDVAANSTTAVHIKPTAKSLPWSSDSSVVFDHCVRPVDALNAARFAHIPRVRGADWRDLPNISVPLSDGSQTNILKYPFKGQIEGCVRKGVCACVEGKKCKSERQKNTLIPWWLPHTAQSNNHWKGVYGRLDERGCFRTIITNPQPGVKQGRVLHPSKDRIASVRECARGQGFPDDYIFVGTIEKRYIQVGNAVPPPLAKAIGIEIRKSLISSQYR*

>Hrob_116156

MSMFANQASLKENSPVNNKKRKSLNDNVNRNNKISSSSANDNESEASDEKVIDELVEQAEKKMKLDSENKDKSETDITNKELMTTNTTEQLQLINHPKNKFIIERCTECGQILDEQEIVMYPGDSGEAVEEFVALTDSRLSLFTGDEQEIDEADERPQHRLTNFSVYDRMTHLCPFDSGLLEKNVELYFSGYIKPIYDDNPSIEAGGICTRNMGPINQWWTAGFDGGEVPVVGFSTAFAEYILLSPSDAYKPFWDAVSDKTQLSKVVIEFLKESIEKDEQPSYEDLVNRIQVSDRFFLSSNRFTEDSLLQHAQFIIDQLQSYDDTADTEDVLLIIQPCIRTLVKLTGVTLGRKNKSFGNTQQTNRKTRQQQQIAAKTAQKKKDSKATVTPLVREVFDSLFKGQIEGQSSAATKRRRCGVCEACQMPDCGKCRSCMDMIKFGGSGRSKQACLIRRCPYMSIKEAEDDDLLDDNSGEIIKGKKRKMNHNKNSEKTEAVTWIGSPLATKYANGEKIFYLGAYIGEHIVKIGDHVCVRPDDPAVPMYVAQVRSMWQEVVKRNNSYGKAMFHARWLNRGGDTVLGETSDPKELFLVDECQDSPLHFIAYPIKVDYVIQSEMDFFGSGGGASHIPGYQTQSGLGCGDNKGSSDNNDDGKSFFCRLSYDQSKARFEDFSEFDSLDVVKNFLNTCASVNEKSISETPFLDDEVGVDELLTKFNYTTSTFNKNGNGKFLEFMTTITSSLKILNNRVIKYDEDVYPELYRKTEYIKGSNEQVPEPFRIALVVGFFVKTESNSRKVKVEEVNVVVQKFYRPENTNRGLVWFGNPSSHKDLNLLYWSEDRLTVSFRNYTGKCTVTFFDESDLGLQATDVLTRYFNDGSDRFYFTEMYLPDSKSLQDVPSSQIVKELAFYKGGGKGKGKSKNKSSLAKQENVSTDEKKPLRALKCLDVFAGCGGLSEGLHQAGIADTRWAIEKDESASQAFRLNNPSCTVFSDDCNELLSLAISGSEFNSTGQRLPKRGDVELLCGGPPCQGFSGMNRFNQREYSKFKNSLIASYLSYCDYYRPKYFLLENVRNFVSFKRGVVLKLALRCLISMGYQCTFGVLQAGCYGIPQTRRRAIILASAPGYKLPFYPEPMHSFSVRGMQLTVAVDGRRFESCVSRTSGSAPFRTITVRDAMSDLPEVNNGAGHIETGYGGEPVSHFQKMSRRKIGENSYTTLLQDHVCKEMSALVLARMQHIPLAPGSDWRDLPNLEIRLKDGTVTRKLRYTHDDKRVGKTASGNLRGVCSCVETGHCDPAHRQFNTLIPWCLPHTGNRHNHWSGLYGRLEWDGFFSTTVTNPEPMGKQGRVLHPEQHRVVTVRECARSQGFPDTYRFYGTVLDRHRQVGNAVPPPMARHIGFEIKKSLQLSLEKKENDKKP*

>Lana_g11540.t1

MEVEDLDSKKERKQETSKDLREEIEAPLRKSKKEKKVPINQPTIMSWFEKGVNKRKLSEIKNDSESNDSGSQEEKRIKTEDGGNGSADGDDKDGAKILRCRAEKPEEVKELLNTKACKQTPLKCKECSQLLQDPDLKLFLGDPDDAREEFVTLTDPNLSLFTGEEEEIHSYDERPQHKVTNFSVYDKNTHLCPFDTGMIEKNKELYFSGYVKPIYDENPSIEGGIPTTKMGPINEWWTAGFDGGEHALVGFTTAFADYFLMDPSEAYAPIMNALQEKIFMSKMVIEFLANNQDATYEDLLNKLQVESYDQGADFDENTLLTNSCMRALIKLAGVTLGKRRALRKEVRGAKAVKDKSAHTLAAVTPLVRNIFDSMFDGQIDEKGTAVDIRRQRCGICEVCQQPDCGKCMACRDMTKFGGSGRSKQACQKRRCPNMAVKAADEDDLLEEGNDDTLDTERKAVEPSTPKKKHKLSKKQKSALEWMGKAIKVETKKSYFEAVLINDQKVSCGDFISVSPDDPSEPLYIAKVAYLFEEGGDKMCHAWWFKRATETVLGETADPLEVLQVNECENIQLEFVVSKIQVLFKAPLPDWSMRGGIEEFDSDNVVKEEDGKTFFYQKWYDPELARFEDPPFDSDPKVDSKHKHCASCSRLMELSKKERPKPSDRLSSESDSSSHMYYGTVSRDGVDYKLGDSVYLLPEAFKFNLKAASKKSKQEKKEVDEDVYPEVYRKSSDYIKGSNEFVPEPFRIGTILEIICKKMTCSKIVNEQNVLLKIRKFYRPENTHKGATGSHHTDLNMLYYSNEEVIVDFQLVKGKCMVVFSDDMNISVDEYFSEGPDRFYFSEAYNSETKEFEEPPSKVRRSTAKGKIRRLRSLDVFAGCGGLSEGFHEAGIAECRWAIEKEEPAASAFKLNNPGSTVFTDDCNMLLRLVMDGKTTNATGQKLPLKGDVELLCGGPPCQGFSGMNRFNSREYSMFKNSLIASYLSYCDYYRPRFFLLENVRNFVSFKRSMVLKLALRCLLRMGYQCTFGVLQAGSYGVAQTRRRAIILAAAPGEKLPFYPEPLHVFAPRAMQLSVVVDNRKYQSNISWTTSAPFRTITVRDTMSDLPTIHNGAKGEEISYGGDPLSHFQKMIRGNQYQPILRDHICKEMSPLVYARMKHIPLAPGSDWRELPNIIVRLSDGSHTKKLVYTHRDKKNGHSSTGALRGVCSCAEGGVCDPMDRQFNTIIPWCLPHTGNRHNHWAGLYGRLEWDGFFSTTVTNPEPMGKQGRVLHPEQHRVVSVRECARSQGFPDTYRFYGNILDRHRQIGNAVPPPMAKAIGLEIKKCIIWKEEHLELSSCFTKEEKEIEPSAVSGIKVDNI

>Dple_EHJ76342

MDNATHSTTRKSDRKASTRDGQLKITSMFAKKRSRSPIESAEKDDTKKLKINTESHEHEVFNEKSKVVNGINSKSDDVNSQESEETSPNLVSMKLNNERSLVDEDENHNIEAKTVPEIINTMNGCNQNGDEMTRDGLENQVQQNAIVEQDPPEPKPTAKIPDQHGHLCPIDGGLIESDVRIYMSGYLKSICSDSPDIDEESIAVKDVGPIIEWFIHGFDGGSRNCITLSTEFGEYNLLKPSAEYTPLMDNLYEKIWLSKVVVEYLEEYHYLQPTYEDLLEVIREHSIPDLEDKRMTEEMLHKHAQFVCDQVVSLEADEDNEPLITLPCMRELIKLMGIKFGKRKVRAKIDYKKIDKKAWTKATTTPLVQKTFEHFFANQLDKTNHELVLRRKRCGVCEACQLPDCGECNACRAMLKFGGHGRTKKACVRRLCPNMAVQQAEDSEIEDEEEYQQMAEKRHLDKIDDALPVKLTGGSNKIIRWIGDPVKADATKVYYEKVEIDGSELSLGDFVMVETSQSNIPALVARVTYMWKESINPKSGYFHAEVFIRSSDTVLGEVGDPREVFLGDRCCHGAPLSSILRKAFVEKKETPADWFKLGGKEVVDHFFEDDGKTYFYQKYYERFTARFEDLPNDPECPNALRKHRFCPSCERKTRRDARDIPKISGKLTEKSEIVKEANRFEWTTIRWRDHDYKKGCGVFLKPGTFRFKNSMINSSNGINRVKLDKVDEDIYPEYYRKTDNYLRGSNIDTGEPFCVGYIAAVTAASEGPLVIPQDIYIKVNVMYRPENTNNRFPHHEDVNVVYWSDEIKEISFSAVVGPCNICYVDNIPQQDHIYDWLEKDPSRVYFRMAFNKKSGQVEDVPQHVKYVGRGDKGKDKGKGKGKSSKGAQSTVTVKVDEVKVRPLRTLDVFAGCGGLSEGLHRSGVAECRWAVENLEAAAHAYSINNKNCIVFNEDCNALLKDAMDGATHSAGGLRIPMQGEVELLCGGPPCQGFSGMNRFNSREYSNFKNSLVASYLSFCDFYRPKYFILENVRNFVAFKKGMVLKLTLRALLDMGYQCTFGILQAGNYGVPQTRRRLIILAAAPGYKLPLYPEPTHVFSRRACSLTTTIDGKRFVTNIQWDESAPRRTCTIQDAMSDLPQICNGANRIEIDYGCMPETYFQRLIRSRDESAKLRDHICKNMAPLIQARMSRIPTTPGSDWRDLPNISVALSDGTKCKVLQYRYDDIKNGRSTSGALRGVCACSAGGVCSVADKQENTLIPWCLPHTANRHNNWAGLYGRISWDGYFSTTVTDPEPMGKQGRVLHPEQNRVVSVRECARSQGFPDTYLFAGSIQDKHRQVGNAVPPPLGAALGREIKKALSALS

>Tcas_TC013587

MSLRKRFRESPAQETGDNTKKHKRTSTHSDKCKFCAQTLNQAVVYTNFPDDYAEESVALTSERLNVYNGTENISFEEGDLPSHKITCFSIYDLNDHLCPLDTGLLQKEVYLHIGGYIKPIFDDDPSPENGIATLDMGPIHEWFIAGFDGGEKVLLGIITNYAQYVLMEPSLEYAPIFKSLQEKTVLFKFVIEFLLSNFSRAPSYEELLAAVENSDNPLLTEEFLLQNAQFVCDQIHSFDLDAGDNNDMPLLTMPCVRTLASLAGVSFRKRQLMRNLLGNQRRTKLQLSKATATPLVRQLFEQLFPNQIQTENCQSPKKTRCGICDTCRSPDCGQCVYCKDMVKFGGDGNMKQPCKLRRCPNLVVQELDESDDETVVTPARGREDGRRPGVSRVLSDGPLQNFRESIDIVLGGVNDDDTGILLNIKTGDFVQLSPKSATKPNTIARVVNIYTATEPMVHVYLFYRGNETILGEVANPQELFASNECEDCPVAAIVGQAKVVYKATPDNWADLGGIECLPSGDDRFFYAKQYDSETATFTDYVKVRNELDSCGHCRLNEERKKIETPTFKDNKVQWRNEFYEAGNSVFLDPSVYQFPIPVECTFDDAKEMNESKYPEYYRKREANASCKKMPQPFCIGLIETLCDGPAGVLMAIRVFFRPENTKGGSMLSYQSDINLLFWSTKFITVPFRKVMGKCYVVYCSDEQKAREWSEGGPYRYYFRQQYDPDCGELVPPTYSCTRLALREGDERPFGRPLKCLDVFAGCGGLSQGFHAAGVANTKWAIENDKPALDTFRHNNRTCHVFRDDCNVLLRNVMSGKGGLPPKSEVEMIVGGPPCQGFSGMNRFNEGEYSLFKNSLVVSLLSLCDYYRPLIFVLENVRNFMLYKGGLILKLTLQCLVAIGYQVRISIVEAGEFGVPQARRRFILIASAPGYQLPRIPEPQHVFLQRGSRLDIYVDGVKYTNGNFWTQSAPYRMLHVRDAIADLPAIEHDDNRPQMPYDDDEGTSHFQRKMRGNQEELLDHICKPIAPIVQTRIKLIPPKGGADWRNLPNVRVQLPDGTMTEVLQYRYRTKKQKDGEPNRGVCACSVEKACDPNDRQSNTLIPWCLPHTADRHNNWAGVYGRLDWSGYFATTTTNPEPMGKQGRVIHPDQNRLISVRECARSQGFPDKTKFFGSVTSKHVSNVEDRSPVKRLEEPQRSPKKLRLEIEIDENMSDNSPERNRRANFSNVNGVNRNLNIHSVKPTTKKLIIKNFKSKGEPKLPDDYHETTWEKLKSAVIAIQQSKPNEYLLEELYQAVGNMCSHKMSHILYNGLSHLIEAHVCSNIERFITEPMDRFLFLKKMNDTWQSHCNQMIMIRGIFLYLDRTYVLQNPNISSIWDMGLDLFRKYFMLHTLVQTRVVEGLLMLIEKERQGDKVDRTLLKSLLRMLTDLQIYNKAFEQKFLQATERLYATEGQRLMQELEVPEFLAHVDKRIHEENERVIHYLDCSTKYQLIHTVEKQLLSEHINNILQKGLDNLLEENRLHDLSLLYQLFSRVKNGLHELCLAFNAFIKKKGRTIVIDPEKDKTMVQELLDFKDAMDNIVACCFKKNEMFSNSLKEAFEHFINQRTNKPAELIAKFVDSKLRAGNKEATEEELERLLDKIMVLFRFIHGKDVFEAFYKKDLAKRLLVGKSASVDAEKSMLSKLKQECGGGFTSKLEGMFKDMELSKDINVAFKQHLNISTLDLIPLDMTVNILTMGYWPTYTPMDVTLPPQMVKFQDIFKEFYLSKHNGRKLQWQPTLGHCVLKARFKAGQKELVVSLFQTLVILLFNESDEHSFEYIKAATNIEDGELRRTLQSLACGKARVLNKIPKGREIEDNDKFKFNNDFVNKLFRIKINQIQMKET

>Nvit_NV11649

MLVEANGAVGGAINTNGQAHSDDDDPKKPKEEIQDDENYVEDQQPNKRVKTGKREKQGKKETKKPVRPAEPICEICLQKLNDEDLRLYIGHPNNAVDEYSVLLDPKLCLFNGDELDITEGDARALNKVTCFSVYDKNGHLCPFDGGLIEKNINIYFSGYVKPIYDDDPSIEGGIPGKDMGPIVEWWVSGFDGGEQAIVSFSTEIGDYVLMDPSEEYAAFMISVREKSFMSKTVIEFLLDEESPSYEDLLNKLQTVAMPKGLPKFTEDILLHHAQFICDQIVSFDESATTDDPLLITTPCMRALIDLAGITFNKGKTVRKGKKRNSRRQEDDWRRGLMRKAQKEKKPSWTKATATQLVNNVFDSFFPDQLANNNTDTVALKRRRCGVCEPCQQPDCGACSACKSMIKFGGPGRSKQACVKRRCPNMEIQEANDEDPDDQEGPEPDAEPSIVAYRKKLGNLKSRTGKIEWVGKPIGTDSKGSFYSAVELDSETIEINDFVFIESVDPTVPLQIVKVIYMWENKMGIKMFHATWLWRGSETVLGETSNSSELFLVDDCQDVPVSYVKSKTTVLHRDTPDNWNELGNTSVNNSMDESKEDITSFYYQKRYDPNNARFEDPLPDLECRPEIKYRFCSACTRCTCLQQQNRPQVFDKLEEKSSKEVLYGMVKFKDEEFRVGSAVFLQPGTFKFKYPLPYQKANRTKREVDEDRFPEYYRKFNDRVKGSNFDTPEPFEIGYITSIYATTNVKLLASTSLHIKVKKLYRPENTFKSESMKGRSDLNMLYWSEEEVTLPFTCVVGKCYLTYSENLDISVEEWTVAGPNRFYFTQMYSHKEEEFDEPPSKACSIGKLIKKSDKIKSKSKKADAPKLIDIPPDFPQIKKKLRTLDVFAGCGGLSEGLHQAGVAESSWAIEVDEAAAHAYRLNNPNAAVFTGDCNAYLKKVMDGETMAGGQRLPQRGEVDLLCGGPPCQGFSGMNRFNSRAYSLFKNSLVVSYLSYCDYYRPRFFIMENVRNFVTFKKSMVLKLTLRCLIRMGYQCTFGILQAGNYGVPQTRRRMIILAAAPGEVLPKYPNPLHVFSKSACNLSVVIDNKKYFPTYDWVESAPYKVTTIRDALSDLPSIKSGKNEDVMSYGSEPLSHFQRKLRAGVSDSLLLDHICKDLGPLVEARMAHIPTVSGSDWRDLPNIVIPLSDGNHTNKLKYNYHDKKAGKSSTGALRGVCSCCTGKECNPLDRQDNTLIPWCLPHTGNRHNHWAGLYGRIEWDGFFSTTITNPEPMGKQGRVLHPDQTRVVSVRECARSQGFPDNFRFYGNIQDKHRQVGNAVPPPLAAAIGMEIRKSVSIAESSSNVKKEEN

>Nvit_NV17734

MSSDECNESPGRSRSRSCSTATSMETDELQSLPPPPEEREDTSREADDEEESKPDADGAGPSYLRPRSHSRLLENDEAGDRQATADHSSKRSRTELATKQSRPKMMHTGRVAASRKEPVCEICRQKLQDKDLKMYAGHPNDAVDEYNAVIDEKLCLFNGEEEHVMQHDWRAINKITFFNVYCRNGHLCPFDSGLVEREAYIYLSGYVKPIYSDDSSIEGAVPSKDIGPIVEWFVTGFDGGQDAIIVLSTPLGEYYLMQPSDDYSPFMRCVKEKTFISKTVIECLLDEPNSEYEDLLNKFETIPMPSGLPRFTEELLIHHAQFICDQILSFDESALSDEPQLIHAPCVKSIIDLSGVTFKKRFRRGRRRQFLENDEDWQKGLRLSKQKAPYWSKATTTQQVHDLFESFFPDQLDNTADKLKLKRRRCGACEACLETDCGQCASCKNMIKFGGTGTSKQACVKRRCPNMQLEGDDDDYDEDDDDKANHKESSIEVHRKMIRALKKKKSAVIEWMGEPIQSAKGDFYNAVMINHDVIKKNDYIFIEPINSSVPMQVIKVKYMWENKMGIKILHGTWLWRGSETILGETSLPRELFLVDECQDVPLIYVQAKANVLVREYSNDCTEKGNIIDALKENSMILFYQKRYDHVAARFEDLLPELNPPKGAEHCFCSTCARDILSKMNRTPQLLELLDDTDDKRIKYGIIRYLNEEFRVGSAVYLKPKTFTFEFPNMNQDTSRGQKREIVDEEKYPEAYRKFNDRVKGSNVDTPEPFDIGYITSIYSTSKSKLLAGVNAYVTVKKMYRPENTHRGESLKKKSDMNMLYWSDEECDVRLNCIVGKCYIAYSENLNQSIEEWSASGPHRFYFSEAYDLNNEEYTEPPAHACSVTKSFKKDVKAKSKSKKVETIIEDIPVSLPQISHKLRTLDVFAGCGGLSEGLKQAGVAESLWAIENDTAAAHAYRLNNPKASVFTTDCNSFLEKVINGETSLGGQSLPKKGEVDLLCGGPPCQGFSGMNRFNSRAYSSFKNSLIVSFISFCDYYKPRFFLMENVRNFVSFKKSAVLKLTLSCLSRMGYQCTFGILQAGSYGIPQTRRRMILIAAAPGEILPRFPNPLHVFSKSTCQLTVDVDNVKYFTFTDVHESAPYRAITVYDALSDLPKIKSGSNEVVMRYENDPLTHFQRKMRSGMDDELALYDHICKDLGPLVEARMGLIPTKTGSDWRDLPNIVVRLSDGTCTTKLEYKYNDKKAGVSSTGAPRGICSCSEGKSCSLRDKQVNTLIPWCLPHTANRHNHWAGLYGRIEWDGFFSTTITNPEPMGKQGRVLHPEQTRVVSVRECARSQGFPDSFRFYGSIQDKHRQIGNAVPPPLAKAIGLEIRKSVSLKEANEVLDRVVVEEES

>Nvit_NV14091

MSESTNSTTKEEKDPVEKDEAKPGGSRDDGKEKRTSNRKRKASTSDPVALADEVKAKLKVKISSKKKKGKKSSKVFSKKSIKDKSPKEKKKTGSRSKKARTEPPVEVPQDASVAVHNELLPPEVLICTTAEVHNEMNSAEIVTDANIEICSDLTVEVAQLEDTRVYEAQEPLQSIDNEDDKKEDQDFDSESCNATLAFTDAESETSRSNYEKFMIDTQNRSNEFLLGFNSKLKPELTMIDPEAKEVREDQEEAMDICKNASNCTKDDLDSEKTMIEVQDTIIDEALLDFNNKQELQLAVLATETVNANPVELNQQKDVEVGKDDDEAMDTCEDFNKDLLKENIEANENEEIDVCGDNDDAEEVHHAMELTEVNADSSNKHTDEGSKEQNKIIDSCKHEQNQESKSLKIKTLKLKTSKSSKQSSSKSSKSRDRKKSKNKSRKDSTTATTSPDPYYKVNKQDDISCRICEQTLNYQLKFYTGEPENGYDEELAIIDSRLLLFNGDEENISREDTRAYNKITSFSVYCNNGHLCPFDSGLIERDVEIYFSGYVKPIYSDDPSIENGVPGKNFGPIVEWWSTGFDIGEQPTVAISTEIGDYILMEPSDEYKPFMHSVLIKILIGKIVIEYLVYEPHATYEDLLNKLQSASSKYDILPNMTEDVLLEYAPFIYLQVMSYDETAKLNEPLMLVAPCIRRLIDLAGISFKVNRGVSSRGQFHKYQERRAGEYRKELLRKVAENRGDNSITSQLIDSLLPDVEEKLITLSKPCTRDPCGSCDRCQRSSCKECEPCSQKNYEDCPRRRCFYREIQEANDEDAKDSVNLSQLKGNSSISVFKKTINIFSSFNAKLEWLGDPLIVEGSGNTFYAAVKHEEETVCVGDFVLVRSVDQSVPAQVVKIAYMWEDNVGLAHFHANLYWWGKDTVLGELARPNELFSINKCNNISVNCITERINIDERYMPRNQDVSTDEDIKANNIFCEKSYDPSDGSFHDLTKPEEVSVLPTHRSCINCEGKHRRSERVEPRTVDELGKKEEGTLYRAVIYKDEEYIVGSCVYLKPKSLYFQFPMLLDHAKSSGKISKKIDEEKYPELYRKAEDAVIDSKFDMPAPFDIGYITEIFTTDKLYIKVKKLYRPENTHRNEVLIKQNDMNMLYWSNEVCTVSFNYVVRKCYVAHIKDFSKSVEEWSNLGPDRFYFMQMYHDKKFTDIERNMYGCEDVPLDPPIDYPDVDAMTSLEIFAGCGGLSLGLRESGVIKKLGSWAIESDVDAANTFQLNNPDITVLVGHPDIVLKNAMKGEFEDSDGKLLPRKDDVEFLCATLPCENYKTMTDITVFKHSYIATFLSYCDYYKPSLFILEADEKLIKRSFVLKLTLACLVSMGYQVTFDLMQVGCYGAPQNRRRSVLLGAAPDSVLPKFPERLHVFPKPLCELGIVIDSKKYTQDTFWTESAPYRSVTVQDALIDLPTTKVAITEEMNYDAKSTSLFSKIMRLNMLEPTVFNHVCKDLGPLVECRISQIPLAVGSDWRNLPNIEMQLKDESYAKKLIYNYDDVEAGKSSNGELRGVCSCASGERCDPRDRQHGCLIPWHLVHSARKNKHWAGIYSRLQWDGFFNGAITNPQPLGTQGPVLHPTQPRVVSVRECARAQGFPDEFVLPQKIGIHNMYRIIGRASSPFLGRAIGHEIARSLAARHRK

>Apis_ACYPI073296

MEIIKSNKGGDKGYVYVVKDGATGTKRCSMKCTGELYTNLKIENPEVKTGHSHLKDVDSVKIEKALCAMKSDSHVKVEHMAGGVNGDPEPKGVKRKCEEPSITQEQHDSLSDNNGMHTDIPITNTKVRCKFCHKTNTEDVCIPSSAAPECIVLTNEVLANIYTDNDDVVQHNLTNYSIYDENGHMCSIDSGVMERNVTIYMTGYVKPIYDNSPSIEDGIAARELGPILEWWLSSYDGGTQALIGINTEYADYLLIEPHPKYKKYMTSVTEKIHLSREVIKNILSSEYDDPTYEDILNCIINSVNPATGNKFTEEDLISHAQFVLNQVLEYDLNDTYHLPLAETQCIETLTQLSGAAKPVKALSRRAGQRTQYVKTDINKREFSKATTTELVKDVFENMFAAQLDIDDKENEGNMNKLVKTMFDDVCNTKIKKEISDIRVNANQVLWKDKVLCEKMFVVVKNNVSTIPLIGCIEYFFKTKFKNMAHIQLFMHSYETVLGETADPQEIFSLKNCTNIDIKNVVRVLDVVQKIPSKNWFQLGNSKLQDLLEPVNRKNKNSFFYQLQYDDKFGRFEYPKPLEPNTVITEGYCSNCEDFDLEQKSKEPELYEFIEQDKNISYFYKFKLFDEEYKVGSFIYLQPEIFKTASRSNMIGEEPFELKKTSKNISNQEQKVIDETKYPEYYRKSLENTMRGSNETTPDPFEIAEILAIYFVDWDRKNIKLIVRRMYRAEQIPIEDYAKNSDMNMLFWSEEEFRVTYQNIRGKCYVSFISNIQDPLLWSSGGPDRFYFTKKYDFIFGSIVSEHELPSEAKLTGKEVYKIKPLRGLDIFAGCGGLSRGLEDSGLVISNWAIECDDKAAGAFKLNNPEATVFVEDCNHLLKLAMAGEKSNSKNQNIPQKGEVDFICGGPPCQGFSGMNRFNSGQYSLFKNSLIVSFLSYIDFYRPKYFVMENVRNFVSFKRSMVLKLTLRCITRMGYQCTFGILQAGNFGVPQTRRRLIIMAAAPGEKLPFYPEPINVFNRKSSSLSVQVGDEKFKTNCKYVNSAPLRTLTVYDAWSDLPGISNGAYQERIPYNSTPITHLQKLLRYPDNQYADSTLCDHICKEMSSLVQARMALIPVCEGSDWRDLPNITVQLPEGLKTNTLLYTHHDIKNGYGPNGALRGVCTCASGDKCDPQDRQNNTIIPWCLPHTGNRHNNWAGLYGRLAWSGFCSTTITNPEPMGKQGRVLHPEQHRVVSVRECARSQGFKDSFIFCGSIFDKHRQIGNAVPPPMGTAIGHAIIKAIYQ

>Apis_ACYPI006318

MTDDEQDEDPQISVKRVKRKFTEMPENKKKPSLTPTDIALNETNHFQGKVCCEFCLKTKTEDVDIPTDAESECIVLANEVLASIFTDNFNIIQHKLINYSIYDDNGHMCSIDSGMMEKNLNLYICGYVKPIYNHSLDIKGGIACNAGPILEWWLSSYDGGAQALIGINTEYADYLLVEPHPNYKKYMTSVIEKINLSKIVIEKMLDNHESDDSTYEDILNYVVSSINPATGITFSEEDLITHAQFVLDQITDYDSTGTYDFPLSETQCIETLTQLSGATQSDYAPIRRLGRLGQGVTCIKSDFSQRKFSKATTTELVKDVFENVFAAQLDIDDKESEETMNKLVKTVFDNVFNEDVSNDLSDVNCVENHVMWKDQKLCEKMFVVVKNNDSATPLIGCIQYFFKTKFKNMAHIQLFMHSYETVLGETADPQEIFSLKNCTNIDIKNVVKVLDVVQKIPSKNWFQLGNSKLQNLLEPVNRKNKKSFFYQLQYDDTFGRFEYHKPEKPINDIRNKYCSNCEVIECEQKSKEPDLLEFIEEESGISYFYKFKLFNEEYKVGSFVYLKPGIFKIATRSNMIEEELFEFEEKNTNISNQEEKNVDETVYTEYYRKTLENSMRGSNETTPDPFDIAEIKAIYFVDRDTKNIKLIVRRMYRAEQILAENVARNEDMNVLFWSEEEFRITYQQICGKCYVTCGANILDPLVWSSGGPDRFYFKCKYDCLTNSIVPVYKLPGEAKLVGKEVYVNYNHQYVDEDKLQYIPSYKKIKPLRGLDIFAGCGGLSKGLEDSGLVISNWAIECDDKAAEAFKLNNPGSIMFVEDSNHLLKLAMAGEKTNSKNQNIPQKGEVDFICGGPPCQGFSGMNRFNSGQYSLFKNSLIVSFLSYIDFYRPKYFVMENVRNFVSFKNSMVLKLTLRCITRMGYQCTFGILQAGNFGVPQTRRRLIIMAAAPGEKLPFYPEPINVFNRKSSSLSVQVGDEKFKTNCKYVNSAPLRTLTVYDAWSDLPGISNGAYQERIPYNSTPITHLQKLLRYPDNQYADSTLCDHICKEMSSLVQARMALIPVCEGSDWRDLPNITVQLPEGLKTNTLLYTHHDIKNGYGPNGALRGVCTCASGDKCDPQDRQNNTIIPWCLPHTGNRHNNWAGLYGRLAWSGFCSTTITNPEPMGKQGRVLHPEQHRVVSVRECARSQGFKDSFI

>Caqu_CAQU007561+CAQU009352

MTNFSVYDKEGHLVPFDSGLIEEDVFLFFSGYVKPVWDENPNIEGGIAAKEVGPINEWWISGYDGGEKALVGFSTSCAEYYLMEPSEEYAPFMSQVLEKIFLSKVVIEFVQHHEGAEYEDLLTKLQLENQEEKVSKAPRRKRCGICFNCQKRDCGNCVACKDMVKFSGTGRSKQACLSRRCPNMAVQMADDSDGEDHDNESAQQKPKEKTPTKQAELPLPISPFREMSWIGEPIKIQNDRSYYLAETADFHEVFPSGHCSDWEMEKINYKVNVFYKPIPENWKTLGGVDMGPEEKIDEKTFWYQKWYDTKTGRFMDMTERIPEPDMGKTCPHCTLLLYQRAIWEHLDLAIGDCVYIPPDIYDYKVKPVAPKPEALKKKEDVDEDLYPEYYRKAADGHAKGNHDGTPEPFRIARIEYIMSRGAPDRDNVYLKVRKFYRPEDTHLGVPATAHSDLNLLYWSEEVADVKFSCVMGKCHVVYRENIVGSVDEWSAKGPNRFYFAEIYNTQDKTFEEPPMKAQAMGRIGKGKGKGKGKGKGKSSGASENVEANPAGSGPTLEEWPTVKKLSCLDVFAGCGGLSEGLHSSGVTETKWAIEKEDIAAQAFRLNNPNAIVFSDDCNELLKLVMEGKETNRLHQRLPQKGEVDLMVGGPPCQGFSGMNRFNTKEYSAFKNSLIVSYLSYCDYYRPRFFILENVRNFVAFKNGMVLKLTLRCLLKMGYQCAFGILQAGHFGVPQTRRRAIIFAAAPGEKLPAFPEPQHCFNPRACQLTIMVDEKKIRGKQAQPLLYDHVCKDMGELIEARIAHVPTSGGSDWRDLPNLEVRDRSNNVIAKKLIYSYNDAKSGKSSTGALRGVCRCAIGGKAGCIASDRQDNTLIPWCLPHTGHRHNNWAGLYGRLSWDGYFSTTTTNPEPLGKQGLVLHPEQHRVVSVRECARSQGFPDTYRYAGNILDKHRQVGNAVPPPMGTALGYEILKCLRPEEHLVNGN

>Hazt_HAZT006446

MSSSHLTNGIESQQHESELPKGGLGFSNGEDGFPLPIPSASMKRKDTARQKSITEMFSFIKKDPQPHNTLQNGCKNASEIAIPSAPFNNQHEVEFDSPKTPVSPNKPVLEADIERDCKTLEIKQKKDDLCDADGIVSSHARDAGNYHILSNGNMKDCDANDSASVQEDKEDCDANDAASVQEDKEDSKVSGDYMSLRFFPESGDEMPLRLQPRDLGTRRLCASSPRPGDEMPLQLQPRDLGTTCLCASSPRPKDEMPLRLIPETWGRDAFAPPAKRSGDYMSLDLIPEILGRDASAPPSPRSGDEISKNCGVIPSMSLKEDKEDCDAKDAASVQEDKEDCDANGAASVQEDKEDCDANDAASVQEDKEDCDAKDAASVKKDNITTSWDSDDPGDEFEGFPSEKRLKLAEPERSYHSSQDQKTSQPAAPKSRCPICRQILDSDSLSFYEGHPQDATEEFIALTDPKLAIFMDDDLEERPQHKLTSFTVYDKQGHVCPFDGGLIERNALLYTSGYIKPIFAEDPTPLDGVPVMDVGPINEWWISGFDGGEKALLGFSTGYAEYVLMDPSPVYKQYVDAVMEKIYLSKLVIEFLYDNEDGGYEDLLNLLQTTVPPPDVVLSEDSLLRHAQFVCDQVHNFDVAGDGSDVLITHPCLRTLVDLAGVTLGKRCALKTRGPRIKTVKKMPKWTKATTTPLVRRCFDQFFADQIDSHKGDNSANDTKEKSGPRRMRCGVCEACLKQDCGECSSCKDMIKFGGSGRSKQCCKDRRCPNMMLAEPDDENEEEAVQKLANLRDAAAQTRSHRVKKTTDKLCWVGEASYRTSKRVYYSSARVNEDVVCRGDCVQIEPDAGVKDLPYIARVVSLWEDMKGEKYLHADWYCRGKDTILGDTSDPQELFIIDDCEDIPLASVMKKVRVVPHHPPPNWRLLGGILHPEDLNPLASDDTHSFFCQLAYVPQHARFIHLSSEQVTIYENELATGSRDCPCCERLRLAELRDSCVLMEKSTETDEYNAVQYMDQKFSVGDCILVEPSAFAFKVKLPSPASRAQRQQEHWDEDLFPEYYRLTQKIKGSNEDTSEPFRVAQILSIRPHSKSASCRAGEVFCGSALPPASVTAVVRKFYRPENTHRGAAAAYQAPLNLLYWSDEGSYIRCCAESSVSFSWVCGKCTVVYAENLTCSPDEFFLQGPYRFIFSQAYDATTKELVEPPKSAINLGSSGKGKGKGKGKGSSNSNTTAVLEDYPSITRRLRTLDVFSGCGARDIVAGLSEGFHQAGVAESCWAIEVFEPAANAYKLNNPNATVFTDDCNLLLRMAMENKTTNSKGQTLPRKGDVELLCGGPPCQGFSGMNRFNSRQYSLFKNSLVASYLSYCDFYRPRFFLLENVRNFVSYKCGMVLQLTLRVLVQMGYQCTFGILQAGSYGLCMKKNRAVKEIADAHDSCAALVRGNGDAVRRLAPGEQLPYFPEPLHSFSPHACSVSAAVADVKYKSNCRWTVCGPLRTITVRDALSDLQPIQNGGGREQVAYTTEPESHFQRMVSLGNLYAAERLHYLSNGLQLPIVRASDGLLLDHQCKSLSDLVAARMQRIPTAPGSDWRMLPNKPVRLPDGSWTKKLEYNHDDKRNGRSSEGHLRGVCACASGAPCDPMDRQHNTLIPWCLPHTANRHNHWAGLYGRLEWDGFFSTTVTNPEPMGKQGFPDAYRFYGSLLEKHRQVGNAVPPPMARAIGLEIRKCIAATEKLHCSDAST

>Dpul_304208

MISFPRFDSPDLKYFPGPPADSIEEIIALTDPSLSLFTGEEEVMSELDQRPILKLTQFGIYDEPGHLCHLDGGAIEANHLLFAYGYVKPVWNDNPGVEGGIATKEIGPINEWFISGFDGGEKAIMCFATSYADYYLMSASELYEPIMNELEEKIFLSKTVIELLIEDDNAEYEDLLTRLQNSLMPNGSRVSEDSLLRYAEFVCDRVYHFDAAGAEDEPPLILSPCMRTLIQLSGITLGKRRATRKLEKRRQPKAKKGPNWTKATTTPLVGYVFESFFRDQMEQGEDKFGPTSAPRRTRCGICEACQQTDCGKCNFCRDMVKFGGSGRSKQSCVLRKCPNRAVQVADDDDELDAVLDTEIEVVSLDVDHKKPVKRHAYKIEWDGPELKEVDDLTFYSAAIVNGDMRVFAGGHVTIEPDDPSIPMYIAEVIALWEDGKSGEQFLHARWFCRGTDTVLGETCDDPRELVLIEDCEDLLLSAVVKVVNVKYKPVDPVKWKAEGGSDDPSLFQSEEDHSDTTFWYRYLYHGRTGRFEDPPECPDVVNNIKGCPCCDRLDVIRQRDFAKLGNKLDSGGFDSVTWHELDVKVGEAVFLEPGAYAMRGPDGSIVKKEKIDPEEDDIFGVDYDDEYYPEKYRKTDNIKGSNNDTPDPFCIGYVVGIAYNGILHNNLNAREVCLKVKRIYRPADTHLGRDAGFRSDWNLVYWSDEIHKLELNKVVDKCVLVCSTAIDEPIEEFVRGGPNRMYFNKAYNPAEREFEPPPVEAERIGSSSKGKGGKSLKSAKSIQPTYPSYPKIEPLKTLDIFAGCGGLSEGLHQSGVAKTYWAIECEPTAAQAFRLNNPDAAVFTDDCNTILKMAIDGQLEQNGQVLPPKDGVELLCGGPPCQGFSGMNRFNSRQYSSFRNSLIVSYLSYCDYYRPRFFILENVRNFVSFKRNMVLKLTMRCLVRMGYQCTFGVLQAGNYGVSQTRRRAFIFAAAPGEKLPLYPEPTHVFSRRGCQLSVAVGRDKFYSNCRWLLSAPYRTVTVRDAMSDLPEIPNGAKQEEISYGGDPQSHFQRWMRGTDSEQSSVLRDHISKEMAPLVEARIAFIPSKPGSDWRDLPNTEVRLKDGVMTVKLRYTHEDKNGRSLSGAMRGVCSCAEGRQCDPLDKQHNTLIPWCLPHTGNRHNNWAGLYGRLEWDGFFSTTITNPEPMGKQGRVLHPEQHRVVSVRECARSQGFPDSYRFFGNITDKHRQVGNAVPPPLARAIGLEIHKCVYEGDCKKKKDEPPTIVQC

>Dpul_117816

MAPLFEARIALIPSKPGSDWLDLPNTEVRLKDGVLTDKNGRSLSGAMRGVFSCAVGRQCDPLDKQYNAHIPWCLPHTGNRHNWAGLYGRLEWDGFFSKTITNFESMGEQCTRVCSLARFTDSYRFFGNITDKHRQVGNAVPPSLARAIGLEIHKCVYEGDC

>Dpul_265693

MVPTYQDSLLRYTKFVCDRVGLHHFDAAGAEDEPPLILSPCMRTLIQLSGITLGKKRATTRKLEKRRQLKAKKDLNWTTATSTPLVGYAFESFFRDQMEQKEDKFGPTSAPRRIRCGICEACHQQTDCGKHGEIWRFWSQQTELCPPEVSEWSCSSGRRRDDELDASNIEWDGPELKEVDDLTFYSAAIVNGDTRVFAGGHVTIEPDNPSIPMYIAHVIALLEDGKSGEQFLHARWFCRGTETVLGETCDDPRELVPIEDCEDLVLSAVVKVVNVKYKPVDPIKWKAEGGSDDPSLLQSEEDHSDTTFWYRHLYHGRTGRFEDPPECPDVTNTTTNITQKSTYRKTDNIKGSNNETLGPFCIGYVVGIAYPALIVSYLSYCDYYRPWFFILENVRNFVSFKQSLVLKL

>Dpul_116947

MVPAYQDSLLRYAEFVCDCVGLHHFDAAGAEDEPPLILSPCMHTLIQLSGITLGKRRATTQKLEKRRQPKAKKDLNWTTATTTPLVGYVFESFFRDQMEQGEDKFGPTSAPRRIRCGICEGCHQQTDCGKCNLCHQTELCPPEVFEWSCSSGRRRDDELDASNIEWDGPELKETCASLPEATSPSNPIEPDDPSIPMYIAQVIALWEDGKSGEQFLHARWFCRGTETVLGETCDDPRELVLIEDCEDLLLSAVVKVVNVKYKPMDPIKWKAEGGSDDPSLFQSEEDHSDTMFWYRHLYHGRTGRFEDPPECPDVVKTLKDVPAVIAWMLSANVILPNLATSWIQSVLTASNGTN

>Dpul_267906

MHVWNPQLTRSSTFYFGAWLVIRLRQVTSGLGSSHQWIIFNKYTYFTTIMRIVRTDGDEAAGNEVKVKEEPTELQSNNESSPDTTPVEEEAKPILKPVEEEANKPTSKPVTVLSKTRGVKVRFDSPDLKYFPGPPADSIDEIIALMDPSLSLFTGEEEIMSELDQRPILKLTQFGIYVEPGHLCHLDGGAIEANHLLFAVLGETCDDPRELVLIEDCEDLLLSTSATESLGRSMERNETR

>Smar_10552

MPRPKPKKAAGKKTNEPKKPTQRALRDSTVKKTNISRGEPDEPITVESKKHKMSESEANHEIIPNKRRKYEKYAKKSTENTEDIAYNHVPDVEKENLNGDKKDVTKMTTNSKPQIIRCTECSQIVDEKIGIFPGDSDDAVDEVTALFNPILALEGREMDDYVSSSLPDYKITQFTVYDVNTHICTFDTGLIEKNRKLYISGVIKPVYDDDPSPEGGVLAKTIGPISEWWVAGYDGGNDVLVGVSTELACYYLMTPSEIYASYMKSIMRKTFLAKLVIEFLNSDETGTYEDLINKIQATPIKGCESFTEESLLHYAQFIVEQVDSYDQAADADENLLNNTECMKALINLSGVSREEKKYIRARIRNVQKKPKDTKATTTPLVRELFELMFKDQIETAPKSTLRRKRCGVCEACQRPDCGACNNCKDMIKFGGKGTAKQVCKHRVCKNLGYVENGDVSDEEEVDENVDVNVKPKREFTIPKQKPKVSWVGEKIDFYSKKKTFYKSVKINSETYNVGDHVLVYPNSPEVPMFTAKIMYMFEDNFTKKKIFHAHWFCRCTDTILGETSSDNEVFLVDECENSLLDIIMSKCEIVYRPPPTSWAYLGGEDVAELMESYGEKKFFYKLFYHRRTARFEDPPKEYIEVDDSEDKKCDFCPCCNRLHTFRNLEKLHLGNILSETSKTIFYESVKWHNVEYSVGSQVFISPDAFKFKAKIRVVPDKNVIEEVDEERYPEYYRKTKDRGENIDLQEPFKIGYVEKIYCAASKNGLVNSSDVKLVVKKFYRPENTHRGIDASLYADFNLLYWSDEEAVVDLTVVQGKCHVTYCTDIARCESLWGAAEHHFHFSEAYNVESKMFEDPPNKAKLLGSRGKGKGKGKGKKTEDIQQDSSPKLLYFKRLKAMDIFAGCGGLSEGFHQAGICDTLWAVECDEMAAQAFRLNNPDCTVLNQDCNDLLRMVIEGQTKNNQGQTLPQKGDIELLCGGPPCQGFSGMNRFTAGQCSQFKNSLIVSYLSFCDFYRPKFFLLENVRNFVSYKQNMILKLTLSCLVRMGYQCTFGVLQAGNYGVPQSRRRAFILAAAPGEKLPNFPEPRHVFKHSSCLVTVDENKYVTNVNWMDSAPYRRITVRDSLSDLPAIENGAKTEELSYTPENQSHYQKILRRNNVTRVIHDHVCKEMRPLVAARIRFIPCQPFADWRDLPNIEMTLSDGSRTRKLEYGFEDGKNGRSSTGALRGVCRCAEAKVFKKCDPLHRHPQDSTIIPWCLPHTSNRHNNWSGLYGRLDLDEHFKTTVTNPEPMGKQGKVLHPDQHRVISVRECARSQGFPDAYQFHGNVLDRHKQVGNAVPPPLAREIGLEIRKCLKYVEPNGESDKNEPTQILTDSLAKPGSSKF

>Smar_15548

MVKIKKSKMREVEVLLDRNEVENYLKNIGTNHTNANTFELKELSIVLEDCLSIKRKLPGLCNILNKKGKYEDVQQNETKNSLASRCPNCLQFLDDNLVSLTVREDAVYETDILSDPNLNVDIGHGAVCYDETSKPEYKMVDFAVYDDEMHLCPFDSGLIQNGHVLYLSGCAKPIFSDDPSSEGGVYVSSIGPITEWWSWGYDGGSAVVIGISSEFADYYLVEPSASYAPFMESTKIKVYLSKLVIEFLEGNENGTYADLINKIQVHTPPEGYSNFTEENLIHHAQFIADHLLSYDQVADADENSLCQTKCMKTLIRLAGVTIRDRREITTSSPKTKNSRGLQKVVRLQPTESKATTTPLVRKVFETIFRDQIASEGRLSSKLVKCRGCKKSDCRSCKNGKKNNSRAQTSDSEVSDSDSEVSHSDIESASEVKTTKFTQLKEFDRSSIQVTWIGNKILTIGNKMFYNSAKVNGELIKVGDSVIVNPENPKSQLLLGKVMYMWEDLKNRGKSFHCHWFCHGSDTVLGSTAKKNLLFLIDECENVRLSDIKSKCTAVYKPPPSDWALLGGTEIENDRDEDDDCYFYSKWYQRSCARFEDPPNIYTDKGTNLKCNYCPCCEVEESARKKKMVEIGDEMDSDSQYTYYNSVSWNDVEYGVGDHLFLSPGSFKFKAKPYAPSKINWPVTEVDEEMYPEYYRKSKIAGKGHIQEPFCVGHVETIYCRSTGLSQLVEPKDVKIRIKKMYRPENTHEGVSASFHSDFNLLYWSNEEAVVDFSDVAGKCNVTYCEDESNDQFYENSNEHNFYFNKVYDAHSKTFEEPSSKLRRLGDRYKRINKDETGRPLRALDVFAGAGGLSEGLHQSKMCETLWAIEFVEVAAEAFQLNNPKCNVYNEDCNVLLKMIMEGKTVNAKGQTLPKKGDVEFLCGGPPCQGFSGMNRFTTGQYSQFKNSLIASYLSYCDYYRPKYFLLENVRNFLCFKENMVLKLSLQCLIEMGYQCTFGVLQAGNYGVPQSRSRAFILAAAPGEKLPSFPEPLHVFKSENKCSVVVNDFKYTINTQWIKSAPYRYVTVRDSLSDLPPIKNGAKIEKLPYCPKEQSHFQKLMRRNSKKDKIYDHICKEMKPLVVARMELIPCEPGSDWRDLPNTNFVLSDGNVTNTLIYRYNDAKHGRSKKGDLRGVCQCVEAKVFKKCDPLYRQPQDNTIIPWCLPHTANRHNNWAGLYGRLELDAHFKTTITNPEPMGKQGKVLHPTQHRVISVRECARSQGFPDSYKFCGSVLDRHKQQSDRYSHQQSCHLTTHHLSLDKQPWLWLQH

>Smar_10448

MSSKYETRSKRPRPSVEKWEKFRKKRHEKKVIDEARKSNEIQKCKYCSQMLEDVSSAPSFNSAIDEKNCELIINPSLTKIKSFKVNNFSVYDQMFHMIQLDWGLMEKEEVVTFISGYVKLVCCDDEYDDSEIGQAVENIGPLTEWWITYDEKDAQIGVTTELGYYYLMTPSADYRVYFYPLKEKSFLVHIIINFLEDNEDITYKELIRKIEAATPPQDYEDDIEVTEEAVRLHAEFILEQIASYDESADDVDKLSRTDCIQHLIVLSGKIIPEKKRMQSTSRSTSTNVQPKQTMQQKKFQDKRMKKKTQKECNTKLVNRIFDSIFQDYVCCLEDVNNRENSDFECEIDEIEQFEHDFYEKELKINYFDDFSSTKLEVSFNNVIECRNEVSLFENFKIHGEVFKSISISLGEDNQHSARQNRKQGSASDTILGKAASENEYFLSNYCQTIGFKSIKCKSDIVFKPQLSNWKSIGGEDLEEKSINNAGRRFYYSKFHDQKTCRFEDIPEAFNNLDADSCPICEIKNRETQGTISLPRYTLNEEIELNLKLYNSVKWNNIEYKIKDSVFLLSSAFGFDAVPRSLPTRENFREVDEDKYPEYYRKAKSKESLSTCEPYRIGEIVAIYTLRKEVGVRIRKFYRPENTHLGKQATYAADFNLLYMSNEETNVNISAIVSKCKVTYCRTPSKIELTYDQQHFYYNQTYDARSEELTRSETHDEEVDEEPAETLPLATLDLFCGCGGLSEGLVQSGAAKSCWAVDEDLVAAASFKKNHPDCVVYGYSCSTYLSMLMDPDCELNLPAKGEVEFLCGGPPCQGFTGLNKFKDRLCAKLKNSLITTQLSICDWLRPRFFLMENVQNFIRCNDSMVLKSTLRCLIAIGYQCTFGLLQAGNYGVPQRRRRVFILAAAPGEKLPQFPLPTHVFNSHSTNSITIDGIQMIPRHNWPTLAPYRNVTVRDTLSDLPDIEDTEPNESQEYNAHIQSHYQKILRKNNNTGLIRDHFYKRMNAITTERILRIPRTHGADWRDLPNIEIKLSDGTWTKKLEYNHDDVRNGRSRNGSRRGICRCAEGGRKSGVNCNIYRQKGTLIPWFLVHTANDNNQWNGALGRLCLDDVFPIALTKLTISGKIGRVIHPEENRILSVRECARVQGFPDSYIFCGDIHDRHKQIGNAVPPLLARAIGSEIRKCI

>Smar_7615

SKNTENKRTINEVYDDQDYETIYKLKTCSVVLKDCLSSSKKKRSNQSVGEDGFYSPEKWKNTHDRQDKSFATSSANRVRVPSRCSHCAQFLTDNIISLEEDINAIDEETALQSPTLIIDGEDQDKYCKLTNFTVHDKLNHVCPFDTGLIEKGKQLFVVGNIKPICDENSDDFNGIRASLGPIIQWWVSGFDGGKKVLVGISTKFAHYYLTKPSEIYEPTMKVVMEKTYLTKLVVEFLENKEDPTYESLLDYLENVDSLEGHEKLNEDKLLYYAPFVTEQIETYDSVADDDESALIETECMKQLMEIAGIIIDGKSGAACNIRKTFKGAPKSKVALKPTTTPIVQRVFANEEIEDEHEDAEIINSSPCTRRTEKLRLKTRCKWIGCSIRCNGKLYYNSAEINGTKFSCGDCALVQTEGNSNSSTHPARIISMWEDVFSNLKLFHVQWFAFGADTVLGDIANEYELFILDQCENLPLKHIKCKCEIEFKSTPVNWSWLGGEDINVVQSPKYFYRKFYQPVSGRFEDPPLEYTDYSEKKYIGHCGCCDRLKASAQQKMVQFGPKLSEFDGENDYQFIKWKNEEFKVGDFVLIQPDAFEFNRKQCQKVKIKKIEKVDEKIYPEFYRKAVPKDIEIPDPYKIGFIIRIYCSVLAANNTQCRTHPSAIQVQVQKLYRPENTHLGIAASYHSDLNELYWSEEGAIVNLTQVRGRCFVNYCEDISNSHLYWDAGPNYFYFHEAYDAENRLFHQPPMSICESLSKLVYSDINKIVTPLRTMDVFAGVGGLSKGLHQAGVSETRWAIELDQFAATAFKMNNPAPELYEDNCNEILQLMIDGKRTNSKGQILPVKGDVEFLCGGPPCQGFSGLNRNRNSISSRNNNSLVSTFLSFCDFLRPRFFLLENIPNFIRFQNNSYVRKSFKSLLDMGYQLAFGLLNAASFGVPQKRKRLIVKIFYYCCSARGGIAAFSGTFVC

>Ptep_PTEP001999

LKYKTGNDVLRGAKFLFTFRVFSHLILVYFYLIKMKIKTEPLSPSKSDLPAISDEETEEKVKKQEEGVDNGNCSSESSDKRTTVKVAQSSRKSKKETGQQPSISSFFTKMQNKRKLEEDENSNSNGQSKKSKSNGKVEDDDKRKVEKDENSNGQSKKSKSSDKVSNGQKSSLLPMRCPDCRQHLDGPDLKLYPGDPDEAQEEFIMLTAPILSLHTEGDYDTVDVNERVQHRITNFSVYDKHTHLVPFDQGLIEKNVELFISGYVKPIFVEDPSDTHGGVLARNLGPINEWYVSGFDGGSKALIGLGTDFAEYILLNPSELYAPYMNAVQEKIYLSKCVIECLIENNEASYEDLLNKLENTVPPQGISTFSEDSLLRHAQFIVDQIESYDSAANEYDPILIVSPAIRALIELSGITLGKQHRIKIPTQGRIAKAKKPVHTLATTTPLVHSIFEMFFTGQLEDDKKNSAPRRKRCGVCEACQLPDCGKCNFCRDMVKFGGSGRGKQACALRRCPNMAVQEANEDDILTDNLEDEMDKKATSTVNGTSSRKGTSVTSKFDVSWIGKSSFEAESRKYYFEAKINDFTLKLGDFVFVTPSEPSTPMFIGKVLSMFETPSGKKKCHAMWFERGTDTVLGETSDPHEIFGTSECGDIGLATIRNKCNVIFKAPAPNWHELGGTEDSLFDTVELVDESSFFYQKWYDADTCRFEDPPLYHNSMPSCDENKTICFGCIKYENAEELATVSFIQYDEDKDTKYYKSGRWLGQTIQVGDCVYINPDVFSFPKKKVVKTENTESEKEVDETVYPEAYRKSDYVKGSNLDVPEPFKIGFITKLFQKKRGDPKVTVKAFYRPENTSRSESLNHSADFNLLFWSDEGNQSFLFENI

>Ptep_PTEP029031

LSEGFHEAGVSDTRWAIEKEEPAAQAFRLNFPECSVFTEDCNLLLRLVMDGQETTYKGQNLPQKGEVELLCGGPPCQGFSGMNRFNSRQYSLFKNSLIVSYLSYCDYYRPRFFLLENVRNFVSFKRSMILKLALSTLVRMGYQCTFAVLQAGQYGVPQTRRRAIILAAAPGEKLPSFPEPQHVFSPRATQLTVVVDDKKYRPNIKWTSSAPYRTVTVGDSMSDLPSIKNGAKAEEISYSGDPVTHFQKK

>Ptep_PTEP002001

AYDSSTGTFDEPPSIAQRIGRVGKGKGNIKGKKTTSNEKVESKPEEYPVINEKLKTLDVFSGCGGLSEGFHEAGVSDTRWAIEKEEPAAQAFRLNFPECSVFTEDCNLLLRLVMDCWAIILAAAPGEKLPSFPEPQHVFSPRATQLTVVVDDKKYRPNIKWTSSAPYRTVTVGDSMSDLPSIKNGAKAEEISYSGDPVTHFQKKLRGQTYQPVLRDHICKEMNPLVEARMRHIPTVPGADWRDLPNISVRLSDGSNTKILSSICLLLFQGRVLHPNENRVVSVRECARSQGFPDSYRFFGTILDRHRQIGNAVPPPLAAAIGLEIKKCLAYKESIKQSSDCE

>Lpol_XP_013780573_1

MPGLRSVLANGPINDRSELSTEEEIRVIKRLEDLKQKLEDEDVTLKGYWKEVCHVCSDLLPQSTKDQVKITESRFKQKLITEEQYANEVKDLVACIIEKAVEGVNLVNEKNVIEDSEQSEKSDGLKNMHETLESDYENAAGSNEDNMDKDENVARSSESEEVQSEKEAENLDNQGDNTTSSYSASKKEQLTIKDAFSNISKKRRHEDVESKNISNSMVDRNSKRKKSEDSQEERTTNKEVKPPAQPPLKCKECGQLLDNTDLVMFTGDPENSVEEFIMLTDPHLSLFTGEENEDNMEDISERPQHKITQFSVYDRNTHLCPFDSGLIEKNKELYFSGFVKPIYEEDPSSQAFADYILMEPSESYKPFMDAMKEKIHISKTVTEFLVNNPEATYEDLLNRIQTTVPPQGLTSFTEESLLRHAQFVVDQIQSYDSAAADDEFLIIASPCMRALIKLAGVTLGKRRALRRAEREIKVKRPSFSLATTTPLVHAVFEHFFQEQLQKDNNNKGATVRRKRCGVCEACQQPDCGKCIACKDMLKFGGSSRSKQACSKRRCPNMAVQEAEDDDAQNEEMEDDMDRKAKETSVKTMKKHQTKKQKTVITWVDKPHQIENGNKYYKAVECGDSVSVAPEDPSTPVYIARVVYMWESPSGAKMFHAHWFCRGSDTVLGETSDPQELYVVDECEDSQLDFVICKCEVMHKPVSENWGTQGGFHNEENEGLAHDEEKKKFFYQKWYEPQICRFEDPPDLTIPNSKDVRQLCISCKRMTRLRELEIAQVGEILEDREKKIFYKAVSWNTRDFRVGDCVYLLPEAFSFPHKSKTQGSKMISKKDVDEEMYPEYYRKTDYVKGSNLDVPEPFCIGKINSIFKHNNQLGKSNSIKLIVTKFYRPENIHILKTASYMQDLNLLFWSDEEVEVDFSLLQGKCYVVYSGNLTQSIHDFTLTGPDRFYFNEAYNSKTKTFEEPPSIAMSMGGQGKGKGKGKGKGKGKNKPDLEIFPEVVMETYPAVSQKLRTLDVFAGCGGLSEGFHQAGLVDTLWAIEKEDPAAQAFRLNFPDCTVFSDDCNVLLKMVMEGQTCNEKGQRLPQKGEVELLCGGPPCQGFSGMNRFNSRQYSLFKNSLIVSYLSYCDYYRPKYFLLENVRNFVSFKRSMVLKLTLHCLLQMGYQCTFGVLQAGNYGVPQTRRRAIILAAAPGEKLPYYPEPTHTFAPRACQLSVMVDDKKFLSNAKWTTSAPYRTITVRDAMFDLPEIKNGAKAEEIPYNGEPLSHFQKLMRGKHCSPVLRDHICKEMSSLVEARIRNIPLAPGSDWRDLPNLVLRLSDGSYTRKLRYTHHCKKNGRNSFGGLRGVCSCAERKACDPMDRQDNTLIPWCLPHTGNRHNHWAGLYGRLEWKGFFSTTVTNPEPMGKQGRVVHPEQHRVVSVRECARSQGFPDHYRFFGNILDRHRQIGNAVPPPLAKAIGLEILKCVAYEAEKTPRMHVEFEKSKHVSESTSKEHV

>Cele_T22G5_3

MPAFPLFHLPYVTLKDTVLKTNEMALLNLALSSDKSRNHVKMFKKPINFIWLNIYEDYCLIDVQFTKKRNIYWKIHKSSKPLKGSKRKIDSFVIDNMEIKNRRHQLRDKYVIESYCDNDRIACIKIAEVMLDIFKPNQLNVYISREETDDICNFIAWDRLNQVDDLTFITYFRLTDKEFHLFKVKILPKKKLTLNFFNYENQNSIQLEFNHDNLEICSAPWFTRQDLLNTNCQNLVIDKTKLTNNDLNAYIKKWLDGADTKLESFKIEIEDPDLDKILDGIQTKNAEDVQRKQDLLNNPPSSFEILFGIRNETSLGPLITRSDNRMATIILDKKCVKFSLWNK

>Spur_XP_780273.1

MPSKTLCDRVIPTNVRNRVQELEGDLNDGLITEKGYIKKKSKMLFEHMSSDMQTTLKGLEDELKDEELTEKGYVNKVKSILAKFLDTCTPISNGDIQQENSSKKKLNKDTQSTVKNGSSSISSNGSGDPCKANGHSNGSHVRASDQEGNGSSQSEERMETDTPTSGKGDSKKKKKKGSPGGSEGGKGSKRKVLGDSDREEEEKKGREENKEVEEEEKEEESAGKTTPDANTPRSSKRKRSPKVDAKQPSIMSMFTKKPAKKEEEKVEESLSSVDENETKMENGDDGKKEEEEEEPSGPGGKRIKKEEEEEEKANDEPMSPSRDLRQRSNNETSIAVKAVTKQPPVRCKECRQPLDDPDLKLFPGDPDDAREEYITLTDPRLSLLTGDEEDTMSYDERLQFKITNFSVYDKSTHLCAFDSGLIEKNKELYFSGYVKPIYDENSSIEGGIPTKRMGPINEWFTTGFDGGEKALIGFSTAFAEYIVMSPSEEYKPFWTAVQEKIYMSKILIEFLQSTQDAVYEDLLSQIQTTVPPEGCNRFTEDTLLRHAQFVVEQVESYDEAADLDEVLLITMPCMRDLIKLAGVTLGKRRATRKAAAVKKDKKPVFTMATVTPLVSHIFDAIFKDQIADEMKAAANERKKRCGVCEVCQAPDCGKCTACKGMIKFGGSGKAKQACKERRCPNMAVQEADENDFDEMDDSNKNGKMDDKKAKRGRKLGSPLKKKKRAKVTWIGEPEEVTKDRAFYKAAMIDDQKIENGDCVQIHPDDPTKPLFIARVIYMWQDSDGEMMFHAQWFVYGSETVLGEASDPLELFPIDECQDTYLASVNDNCSVIYKAPSPDWSMLGGIDDPETDHVIKEDDGKTFFYQKWYDPDLARFEDYELLSRPDDVPAHRFCSCCLKMERIQEKESPRPGAKLEDQEDSSRITYSSCHFKGNEFQIGDGVYLLPEAFSFNIKQKVVTKKPISKKDVDEDLYPENYRKHSEYVKGSNLECPEPFRIGKIVAIYTTKSNSKVKLQVNKLYRPENTHKGRTAAYQADLNVLYWSEEEAIVELSDVQGQCSVVCAEDLNVSVDEYSAGGPHKFYFREAYDSKRKCFEDPPSKSRSNRMKGKGKGKGKGKAKGKTEKQEEKESTDNPFHKLKCLDVFAGCGGLSEGFHQAGICESSWAIEKEEPAAQAFRLNNPGSTVFSDDCNELLRLVMQGDKTSRTGQKLPQKGDVELLCGGPPCQGFSGMNRFNSREYSKFKNSLISSYLSYCDYYRPRFFLLENVRNFVSYKKNMVLKLALRCLIRMGYQCTFGILQAGQYGVPQTRRRAIILAAAPGEKLPFYPEPLHVFSPRACSLSVMIGDKKIQSNNQWCLSAPYRTISVRDSMSDLPTISNGAQKLEISYDGEPQSDFQKKIRGNQYQPILRDHMCKDMSALVAARMKHIPLAPGSDWRDLPNIPVPLKDGTTCRKLRYTHRDTKSGKLSTGALKGVCSCAEGEACDPSDRQFSTLIPWCLPHTGNRHNHWAGLYGRLEWDGFFSTTVTNPEPMGKQGRVLHPEQHRVVSVRECARSQGFPDTYRFFGSILDKHRQVGNAVPPPMAAAIGMEIKQCLQAKAKKDQERQALEPVKEEIEESMD

>Bflo_204680

PVKCKECRQLLDDPDLRMFPGDPQEAVDEVEMLTDERLSLLEEDQEYGTYDDRPQHKITNFSVYDKNTHLCAFDTGLIEKNVELYFSGAVKPIYDENPSADGGVLAKNVGPINEWWTAGFDGGENALIGFTTAFAEYIVMQPSEAYAPFMDTMWEKIHMSKLVIEFLVNNQEAAYEDLLNKIQTTVPPQSLRVTSFSEESLLRHAQFVVEQVENFDLAGDADEAPLLTTPCMRALIKLAGVTLGKRRAGRSGIKQPRKVKAKPMRQTKATTTNLVCSIFDTFFKDQIQDDKAGGPRRRRCGVCEVCQQADCGTCVACKDMTKFGGTGKSKQACVNRRCPNMAVQDADDDDDIEDDLDKEFSSTVGHKRTHFSPRKKVNSKVKISWEGKPIKEEGKKTFYSAVMLEEEKIETGDCVTVTSEDPTKPLFIARVMYMWEDSGGDMMCHVGWFCRGSDTVLGETSDPLEVFLVDECEDILIQFVKFKCKVILKTFDKDWFSHGGVEDPDSDYPIKEDDGRTFFCQKWYDPDLARFEDPPVLEEGSGHKFCPLCLRLDHSRKSEIPIVGEQLDDTMGDDNKVYYSLCQKGGLEYKIGDDVFLLPDSFSFSVKVKNSTKKASKKDNYDEDLYPEYYRKRGDYVKGSNEECPEPFRIGRIISIYCKRTVSGIINTHDVRLRVTKFYRPENTHKGKAGSHHTDLNMLYWSDEEAVVDFMSVQGKCQVVYGEDLDVSVEDWSSKGLHRFYFNEAYNTESKQFEEPPNKARSGGKKAKGKGKGKGKGKGKSSEAESKEQQPPSPAFTKLRMLDVFAGCGGLSEGFHQAGIADSKWAVEVMEPAAQAYRLNNPDCTVFTDDCNILLKLVMEGATTNSTGQRLPQKGDVELLCGGPPCQGFSGMNRFNSRQYSQFKNSLVVSFLSYCDFYRPKFFLLENVRNFVSFKSSMVLKLTLRCLIRMGYQCTFGVLQAGNYGVAQTRRRAIILAAAPGEKLPMYPEPQHVFSPRACQLSVMVDEKKYNSNTQWRQSAPYRTITIRDTMSDLPSIRNGHSATEISYDGEPRSYFQKIIRGNQYQPILRDHICKDMNALVEARMRHVPLAPGSDWRDLPNIVVRLSDGSSSKLLQYTHEDKKNGTSSTGALRGVCSCAEGTACDQNDRQFNTLIPWCLPHTGNRHNHWAGLYGRLEWDGFFSTTVTNPEPMGKQGRVLHPEQHRVVSVRECARSQGFPDTYRFYGSILDKHRQVGNAVPPPMAAAIGREIKKCLEDKAKKETQTGETKPVSMEA*

>Cint_XP_002122948.2

MKCRECRQLLDDVAIFGGDVDQAEEEFVLLTHESLSLFVGNEETGRPQHKITDFTVYDKSTHLCPFDAGLIEKNVELYFSGLVKPIYDEDPGVGTGIPGKRLGPINEWFTAGFDGGEKALVGFSSAFADYFLMEPSHAYAPLWASIQEKIHMSKIVIEFLSDVPDADYEDLVNRIQTSVPPPTLGVAKFTEDSLLRHAQFVVEQVESYDNARSDEEDSLLGTTCVRDLIHLAGVTLGKRRVARRSAVVKEKAMKASSTKATTTRLVAEVMEKFFGGTVDVTTGNKIRRRRCGVCEACLKPECGKCTSCKDMVKFGGGGKLKQTCIDRRCPNLGLDLMEDSDPDDKDEKVKPSPSKMKVIKAGKVTTSWVGEALYEEGGKVYYPGVRVGGDVIEGGECVSVKNKDEGGLPYIGRVVYMWGESGGKMVHVDWFMRAADTILQETSQPDELLIIDECDDLSVECIAGKVDVSHRVPHQNWSFMGGVEEQHPTTDGVSSFYYQKMYVPEFARFVDPPSNEPTDDPDFCPSCVRINEGKMRQVAVGKNELKRENGKIFYEFVCKDGVEYGVGDSVYLSVETYSFKSKNKHKKQVLPKSLDEIDEDEYPEFYRKGGYVKGSNLEVAEPFRIGRINEIFGGKKXSISLVVTKFYRPENTNKPLKSNSYCDLNLLYWSNDKVEVDFSFVEGKCEVECELDLSVTVAEYSRKAANRFYFSEVYNEEDGTFTVPPSTARRSGVELLKQKAKGVKPKLTKMNKMMKKDETIVGEDETKIPKLRSLDVFSGCGGLSEGFHQAGIAEPSYAIELWEPAAQAYRLNNPGATVFTEDCNVLLEMVMNGEERSKCGQRLPQKGDVELLCGGPPCQGFSGMNRFNSREYSRFKNSLVVSYLSYCDYYRPRFFLLENVRNFVSFKNCMVLKLSLAALLRMGYQCTFGVLQAGHYGVSQTRRRAIILAAAPGEQLPLYPEPLHTFSTRGGSLSAQVGDTRYTNNIKWSTSAPYRTITVHDAMSDLPKIPNGHAKLEMPYTGEPLTHFQRMMRGKDYQGVVRDHICKDMSPLVAARMALIPLIPGSDWRDLPNKPHKLSDGTTAKVLRYEYHDKKQGRSSTGGMRGVCSCAEGRACDAMDRQFNTLIPWCLPHTGNRHNNWAGLYGRLCWDGFFSTTVTNPEPMGKQGRVLHPQQHRVVSVRECARSQGFPDSYRFFGSILDKHREVGNAVPPPMSKAIGLQIKKSLEWKVQQ

>Pmar_GENSCAN00000023487+GENSCAN00000022101

MAEASSRPSSRRAAPQTSIRESPRFDYRPRLTRPAPHPLISNARFAVQGYMKKKLGLVQPFLSGEVKTQLETLQEELKEEDITEKGYLNKVKTLLLRELPKLEEEGSEKPHQNGAAKNGKVNHGKNGTTHHAATAAAATTATACKTESVEANGDEAATENGDAHPAMANGSPECSGKAVANGDGLVFVKSDAEMDDGEVEGDDAEPTSSSSSSSSAASAGKTRGRKKKQAAEAESSGAELASPRGRGGQRGRKSVGAAGGSQQTIASMFGKGLVSHAHIMNIMIQDRAAYRVREALAGTPPPKCTECKQFLDDRDLKLFQGDPDDALDEPEMLTDERLSVFESNSDAMESYDDLPQHKVTNFRPLIAENCRLIVFAFSLIYARDLVMSECSVYDRKLHLCAFDTGLVEKNVELYLSGVVKPIYDENPCNDGGVSAKKLGPINSWWTTGFDGGEKALVGITTAFADYILMNPSEEYASTFAVMQEKIYMFKIVIEFLQHNRDATYEDLLNKIETTVPPCGLSFTRFTEDSLLRHAQFIVEQVESYDEAGDEDEPPIIITPCMRDLIKLAGVTLGKRRAQRRQTIKHPTKIAKDAKGPSKATTTLLVRQIFDEFFDEQIEQRDKENVIKRRRCGVCEVCQQQDCGKCSTCKVMPKFGGIGRFKQACMNRKCPNMAVKEAEEDENFEESDSEEVNKIHQKKVTDIKKKKKSKSQISWIGEPQKTETERKYFQRVRLDDEELGVGDCVVVCPDDPSKPLFPARITSMWEEENGEKWFHPHWFCRGPDTVLGETSDPLELFLVDECEDMLLSYVYGKVSVTYKAPSPNWFMEGGMETEEEKTSKDDEEKSFFYQLWYDPEYARFESPPEVTPAEENKFKFCASCVRLSELTQRETPCVETRLDDPEDDAIVTYMLALKDGVEYRVGDGVYLMPDAFNFSVKPASPVKRAPRKEDIDEEVHPEYYRKSSDYIKGSNLDAPEPYRVGRISQIFCNKRNNRETDIKFKINKFYXPENTHKGMSAAFHGDVNLLYWSDEETVVEFRNVQGKCRVEYGEDLPESVAEYSQGGPDRFYFLEAYNAKTKSFEDVPYNARSAGCRGKGKGKGKGKGKGKVSVEHSVEKEPEEKVRKLRGLDVFSGCGGLSEGFHQAGITETKWAIEMWEPAAQAFRLNNPGSTVFTEDCNILLKLVMSGEKTNGLGQRLPQKGDVELLCGGPPCQGFSGMNRFNSRTYSMFKNSLVVSYLSGVAIGSIIKPELKVLRLNGHPGWAVRCGADTAQGDNPRSRPGREAAVLPRAPACVRPARLPAQRRGRRQEIRQQRHQVRNARMRVSSAPFRTITVRDTMSDLPEIRNGASALEISYNSEPQSWFQRQIRGMQYQPILRDHICKASTHTLTFSKTGGNPDLSPLVEARMRYIPLAPGSDWRDLPNIEVRLSDGTTSKILRYTHHDRKNGRGSAMLRGVCSCSEAGKPCDSADRQFNTLIPWCLPHTGNRHNHWAGLYGRLEWDGFFSTTVTNPEPMGKQASAMVLHLYLNCISLATHFPCAFTPGRLWLRFIRTLAVVLCAVLDVECLALATIWNLDVYEELTLKIFLHRLDIQNVPQGRVLHPEQHRVVSVRECARSQGFPDTYRLFGNTLDKHRQVGNAVPPPLAKAIGLEIKKCVEQSRKNELAKNVNNSHALGYWM

>Cmil_AFO94067.1

MPARTGSAAAASSPSSSSPLPEDIKRRLQALEDKVDGLTEKECIKEKLSLLHGFLQADVQNNLNELETKFQKDELSEEGYFAKVKVLLSQEPPVENGDSELGPNANGCAENGARSGKKDDDRDKANGVAGERNGQEQNLMETGGEDPTPQAAKSRGKRQSKSNGDNRRTSGSPRITRNSMKQQATITSMFAKAANKRKSEELNDEEQVPVKKEEESEEQEEQDEKRIKVESEETLPEGAETNADRKPKADQTPKTPPPKCVDCKQFLDDPDLKYFQGDPDDALEEPEMLTDERLSLFEGTHDEGFESYDDLPQHKVTSFSIYDRKGHLCPFDTGLIERNVELYFSGVVKPIYDDNPCLDGGVKAKKLGPINAWWITGFDGGEKALIGFTTAFADYILMDPREEYAAIFAVMQEKIHMSKIVIEFVQNNPDSTYEDLLNKIETTVPPAGLSFSRFTEDSLLRHAQFVVEQVESYDEAGDVDEQPIIIIPCMRDLIKLAGVTLGKRRAARRQAIRHPTKIDKDKGPTKATTTSLVYQIFDTFFSEQIDQNDKENGLKRQRCGVCEVCQQPDCGKCRACKDMVKFGGSGRSKQACLQRRCPNLAVKEADDDENDEEDSETVGEVCPKKILQARKKKQSKNRICWIGEAIKSDGRKTYYQKVSVDDELFAINDCVVVSPDDPTKPLYLARITSMWEEGVCKMFHAHWFCRGTDTVLGETSDPLELFLVDECEDMQLSYVDSKVTVIYKGPSKNWELEGEPENHFELKVVDDDGKTYFYQMWYDPEYSRFQMPPSCEPTEENKHKFCASCSRLAEIRQREMPRVMESVDAKDDSKVFYSLASKNGTQYRVGDGVYLLQDSFSFSVKPSSPGKRPIKKDDVDEDLYPEYYRKSSDYIKGSNLDAPDPFRIGRIQEIFCHKRSNGKPNEADIKLRINKYYRPENTHKGLKASYHTDINLLYWSDEEVVVDFKDVQGRCRVEYGEDLTTENIHEYSAGGTDRFYFLEAYNAKTKSYEDPPNRARSSANKGKGKGKGKGKGKSKATAEPEQTEQENAESKFQKLRSLDVFSGCGGLSEGFHQAGISETLWAIEMWEPAAQAFRLNNPGATVFTEDCNVLLKLVMAGEKTNSLGQKLPQRGDVELLCGGPPCQGFSGMNRFNSRTYSKFKNSLVVSYLSYCDYYRPRFFLLENVRNFVSFKRSMVLKLTLRCLVRMGYQCTFGVLQAGQYGVAQTRRRAIVLAAAPGEKLPLFPEPLHVFAPRACQLSVAVDDKKFFSNITRTKSAPYRTITVRDTMSDLPEIRNGASALEISYNGEPQSWFQRQIRGTQYQPILRDHICKDMSALVAGRMRHVPLAPGSDWRDLPNIEVRLSDGTMTKRLRYTHRDKKNGYSSTGALRGVCSCAEGKPCDSADRQFNTLIPWCLPHTGNRHNHWAGLYGRLEWDGFFSTTVTNPEPMGKQGRVLHPEQHRVVSVRECARSQGFPDTYRFFGNILDKHRQVGNAVPPPLAKAIGTEMKLHMTENMKEKENAAEAVKQESMDIAE

>Locu_ENSLOCP00000009053

LFVKQTKFKLEMPAKTSLSLPEDVKKRLQVLDTDGDGLTDEERVRETLSLVLGYLKADAQNQLNNLESKLKKEELSVEGYLSKVKALLGRELCLENGSSELNGQANGCAENGAHGASDEDCGPRNGQGAEGTVEAREEEGLKSPSTGKPRGGRRSKTEGDTKIYVSLSPGVLRGWIPNCVMYTNPFYNQSSFPTGGVPCDTLVGESGKCWRTLAIRKQGGRRRRNSEPGRMLRQISESFSSSRVTRNSVKQPTILSMFAKSSSQPTSLGLLFNVFSIVESPLPSSSKRKSDEVNGEEVAPVTEEGVTDDEEKAQDEKRLKVESEEKTDTKASVQKNPVPPAKTPPPKCPDCRQYLDDPDLKFFQGDPDDALDEPEMLTDERLSLFDANEDGFESYEDLPQHKITNFSIYDKKGHLCPFDSGLIEKNVELYFSCAVKPIYDDNPCMDGGVPAKKLGPINAWWITGFDGGEKALIGFTTAFADYILMDPSEEYAPIFALMQEKIYMSKIVVEFLQKNPEATYEDLLNKIETTVPPAGLNFNRFTEDTLLRHAQFVVEQVESYDEAGDSDEQPIIVTPCMRDLIKLAGVTLGKRRAARRQAIRHPTKIEKDNKGPTKATTTKLVYQIFDTFFSDQIEQNDKESSAKRRRCGVCEVCQAPDCGKCTACQDMIKFGGSGRSKQACLKRRCPNLAVREAEDDENIDEDDVLPVQTTPKKMSQAKKKKQSKSKLSWVGEPVKTVGKKDYYMKVSVEDEILQVGDCVSVSPDDPSKPLYLARITALWEDNNGKMFHAHWFCRGTDTVLGESSDPLELFLVDECEDMQLSYVKGKVNVMYKAPSNNWFMEGGMDSEIKVIDDDGKSFFYQLWYNPEYARFETPPNVSPAEDCEIMFCDSCTRTKELMEQEIPRVLEPLSEDHDSKVFYGLACLRGEQYRVGDGVYMLPESFSFSVKSASPVKRPHRKDDVDEELYPEYYRKSSDYIKGSNLDAPEPFRVGRIKEIFCLKRSNGKPNETEIKLRLYKFYRPENTHKGPKAGYHTDINMLYWSDEEVTVDCKDVQGRCTVEYGEDLIESVQEYSSAGPDRFYFLEAYNSKTKSFEDPPNHARSASHKGKGKGKGKSSSSTVQEQLDQEPQDLKPHKLRTLDVFSGCGGLSEGFHQAGVAETLWAIEMWDPAAQAFRLNNPGTTVFTEDCNVLLKLVMSGEKTNSLGQKLPQKGDVEMLCGGPPCQGFSGMNRFNSRTYSKFKNSLVVSYLSYCDYYRPKFFLLENVRNFVSFKRSMVLKLTLRCLVRMGYQCTFGVLQAGQYGVAQTRRRAIILAAAPGEKLPKYPEPLHVFAPRACSLSVVVDEKRYVSNITRVNSGIYRTITVRDTMSDLPEVRNGASALEISYNGEPQSWFQRQIRGSQYQPILRDQICKDMSALVAARMRHIPLAPGSDWRDLPNIEVRLSDGTTTKKLRYTHSDKKNGRSSSGALRGVCTCAEGKPCDPADRQFNTLIPWCLPHTGNRHNHWAGLYGRLEWDGFFSTTVTNPEPMGKQGRVLHPEQHRVVSVRECARSQGFPDTYRLFGNILDKHRQVSLVDLQKCF

>Drer_ENSDARP00000013243

MPTKTSLSLPEDVKERLQVLDEGGDSLSDEECVKEKLRLLQEFLLADTQDQLKNLEDKLKSSELSTEVYMSEVKAVLKKALGVVKEGDGVEQNGHSNGFSENGSHKDNGEQEGAMDTQEEGDAIKSPSAPKGRGGRRSKADSEPKKSPASSRVTRNTGKQQTIVSMFSRVPKRKSDELNGEPANGDTEIKTEETITEEVREEKRLKTEDEKPEAENAANLKPVSTAKTPPPKCPDCRQYLDDSDLKFFQGDPDDALDEPEMLTDERLSLFDSNEDGFESYEDLPQHKITNFSVYDKRGHLCPFDSGLIEKNVELYFSCAVKPIYDDNPCMDGGVPAKKLGPINAWWITGFDGGEKALIGFTTAFADYILMDPSEEYSAIFALMQEKIYMSKIVVEFLQKNQDATYEDLLNKIETTVPPAGLNFNRFTEDTLLRHAQFVVEQVESYDEAGDSDEQPIIITPCMRDLIKLAGVTLGKRRAARRQAVRHPTKIEKDNKGPTKATTTKLVYLIFDTFFSDQIDQNNKDGGVKRQRCGVCEVCQAPDCGKCSACKDMIKFGGSGRSKQACQKRRCPNLAVKEAEDDENMDEEDVLPVKDTKKMSQTKKKKQTKNKISWVGEPLKTEGKKEYYMKVRVENEVLEVGDCVSVSPDDPSHPLYLARITALWDDGEKMFHAHWFCRGTDTVLGESSDPLELFLVDECEDMQLSFIHGKVNVFYKAPSENWYMEGGMDEDIKVIDDDGESFFYQLHYEGECARFETPPKVTPSEDCKYKFCASCTRNKEREAESVPHAYEPLEDEESDSKVFYGLVNYKGEQYKVGDSVYLPPEAFNFVVKAASPVKRSHRKDDVDEDLYPEYYRKSSDYIKGSNLDAPQPFRIGRIKEIFCNKRSNGKPDTSEIKLRLYKFYRPENTHKGPKGAYHSDINQLYWSDEEATVSMTEVLTRCRVEYAEDLVESVQDYSNKGPDRFYFLEAYNAKTKSFEDPPNHARSAVNKGKGKGKGKGKGKGKAAPQEPQDQEPQEPVVPKLRTLDVFSGCGGLSEGFHQAGISETHWAIEMWDPAAQAFRLNNPGTTVFTEDCNVLLKLVMSGEKTNSLGQKLPQKGDVEMLCGGPPCQGFSGMNRFNSRTYSKFKNSLVVSYLSYCDYYRPKFFLLENVRNFVSFKRSMVLKLTLRCLVRMGYQCTFGVLQAGQYGVAQTRRRAIILAAAPGEKLPRYPEPLHVFAPRACSLSVAVDEKKYVSNVTRGNGGIYRTITVRDTMSDLPEIRNGAAALEISYNGEPQSWFQRQIRGSQYQPILRDHICKDMSALVAARMRNIPLAPGSDWRDLPNFEVRLRDGTTTKKLRYTHPDKKNGRSGTGALRGVCSCSEGKPCDPADRQFNTLIPWCLPHTGNRHNHWAGLYGRLEWDGFFSTTVTNPEPMGKQGRVLHPEQHRVVSVRECARSQGFPDTYRFFGNVLDKHRQVGNAVPPPLSKAIGLEVKKCVLEKMRENATEPVKQEKMELSD

>Lcha_ENSLACP00000012502

SPPPKCTDCRQYLDDPDLKFFQGDPDDALEEPEMLTDERLSLFDANEDGFESYEDLPQHKATYFSVYDKKGHLCPFDTGLIEKNVELYFSGAVKPIYDDNPCLDGNIRVFKKKSNIVYIKGFPKSQQLGNALTLAFADYILMEPSEEYSAIFAVMQEKIYMSKIVVEFLQKNPDATYEDLLNKIETTVPPAGLNFNRFTEDSLLRHAQFVVEQVESYDEAGDIDEQPIIITPCMRDLIKLAGVTLGKRRAARRQAIRHPTKIEKDKGPTKATTTKLVYQIFDIFFSEQIEQNNKENIFKRRRCGVCEVTHLCINGSVYGLESCTKFSKNSVSIYSTPCTKFCLMCPRLGIADVVGEETVTWNFLDFATFTNDLKKKKRTHQKPRIVYIKGSLQSDGKKDYYLKASIDAEVLEVGDCVSVSPDDPTKPFFLARITALWEDDDGKMFHAHWFWRGTDTILGETSDPLELFLVDECEDMQLSYVQNKVNVLYKKPSENWFMEGGMDDDIKVVEDDGKTYFYQMWYDPEYARFESPPSCVSAEDNKYKFCLSCTRLDEIRQKEIPKVLEPIDESDAKVCYGLATKNGAQYRVGDGVYLLPEAFSFNLKPASPAKRPKKEEVDEDLYPENYRRSSDYIKGSNLDAPEPYRIARVKEIFCNKRSNGKPNEADIKLRIYKLYRPENTHKSLKASYHADINLLYWSDEEVTVDFKDVQGRCSVEYGEDLTESIQEYSAGGLDRFYFLEAYSAKSKTFEDPPNHARSAGNKGKGKGKGKGKGKGKASVPSEQTEQETAESKVLKLRTLDVFSGCGGLSEGFHQAEIAETLWAIEMWEPAAQAFRLNNPGTTVFTEDCNVLLKLVMAGEKTNSLGQKLPQKGDVEMLCGGPPCQGFSGMNRFNSRTYSKFKNSLVVSYLSYCDYYRPKFFLLENVRNFVSFKSSMVLKLTLRCLVRMGYQCTFGVLQAGQYGVAQTRRRAIVLASAPGEKLPMYPEPQHVFAPRTCQLSVVVDDKKYVSNISLISSAPFRTITVRDTMSDLPEVRNGASALEIAYNGEPQSWFQRQIRGSQYQPILRDHVCKDMSALVAARMRHIPLAPGSDWRDLPNIEVRLSDGTTTKKLRYTHHDKKNGRSGTGALRGVCSCAEGKPCDSADRQFNTLIPWCLPHTGNRHNHWAGLYGRLEWDGFFSTTVTNPEPMGKQGRVLHPEQHRVVSVRECARSQGFPDTYRLFGNILDKHRQVGNAVPPPLAKAIGLEIKSSMLEKLKANSSDVVKQEKMDTTD

>Xtro_ENSXETP00000047703_3

LTESVSPLLYTAMPAQSASLALPADVRKRLKDLERDQDGMTEKECVQQKLSLVLGFLEADARNKLSDLESKLSSEELSEEGYLTKVKALLRKQLSCENGDLSLNGETNGCSTNGTCGSDEEDVEMSESNTAGVKNRKTRKSKTNGENKTESPARARSSRSTTGKQATILSMFSKGSSDLAMHQHSCNITFDNLITSDGIRRRCSIPFQHIMLEYQFQVDKKRPTSSVKFQWSLALTSNSTESSVLSILKASPFYTITSGLLRSNKRKSSDEEKDTDVSADADQPEEKEKEEKRIKIEVNESENENRSTAEESKKVKPVLPPKTPPPKCMDCRQYLDDPDLKYFQGDPDDALDEPEMLTDERLSLFEANEDGFESYDDLPQHKVTCFSVYDKRGHLCPFDSGLIEKNVELYFSGYVKPIYDDNPSLDGGVRAKKLGPINAWWITGFDGGEKALIGFTTAFADYILMDPSEEYSSIFALMQEKIYMSKIVVEFLQNNPDVSYEDLLNKIETTVPPSGLNFNRFTEDSLLRHAQFVVEQVESYDEAGDSDEQPVIVTPCMRDLIKLAGVTLGKRRAARRQAIRHPTKIEKDNKGPTKATTTKLVYQIFDTFFSEQIEKDADKENGIKRRRCGVCEVCQQPDCGQCKACQDMLKFGGAGRTKQACTQRRCPNLAVKEADEDEEVEDVVPEMPSPKKILQGKKKKLEKKNRISWVGDPIKTEGKKDFYLKVSIDSEILEVGDCVSVSPDNPTEPLYLARITSMWEDACGPMFHAHWFCLGTDTVLGATSDPLELFLVDECEDMQLSYIHGKVKVIYKAPSDNWFMEGGTHTDIKVVEDDGSTYFYQLWYDPEYARFETPPTPQPTEDNKYKFCTSCARLAEIRQREMPRVSCPVEDLDSKVCYSTAIKNDVQYKVGDGVLLLPDAFSFSVKLGSPMKRPQKKDDVDEDLYPEYYRKSSDYIKGSNLDAPEPYRLGRIKEIFCNKRSNGKANESDIKLRIYKFYRPENTHKGVKASYHSDVNMVYWSDEEAVVDFKAVQGHCTVEYGEDLTETIQEYSAGGSDRFYFLEAYSAKTKSFEDPPNHARSAVNKGKGKGKGKGRTQTNSSKGKTASKSENEQQNCGDKLPKLRTLDVFSGCGGLSEGFHQAGISETNWAIEMWEPAAQAFRLNNPGTTVFTEDCNVLLKLVMSGEKTNSLGQRLPQKGDVEMLCGGPPCQGFSGMNRFNSRTYSKFKNSLVVSYLSYCDYYRPKYFLLENVRNFVSFKRSMVLKLTLRCLVRMGYQCTFGVLQAGQYGVAQTRRRAIVLAAAPGEKLPMFPEPLHVFAPRACSLSVVVDEKKYVSNITRTNSSLFRTITVRDTMSDLPEIRNGASALEISYNGEPQSWFQRQIRGSQYQPILRDHVCKDMSALVAARMRHIPLAPGSDWRDLPNMEVRLSDGTTTRKLRYSHHDKKNGRSSTGALRGVCSCSEGKPCDPADRQFNTLIPWCLPHTGNRHNHWAGLYGRLEWDGFFSTTVTNPEPMGKQGRVLHPEQHRVVSVRECARSQGFPDTYRLFGNILDKHRQVGNAVPPPLSKAIGSEIKKCVLSREKENGTETVKAEKMET

>Acar_XP_003226840.1

MEEEFSALEGQRRKRLEEQKVANGLDAEVTTGPSSLSVEALKEVRLKDLERNEDGLGERECVKEKLSLMHGFLQADVQNQLKDLETKFHKEELSEERYLAKVKALLHKELSFENGDNLELGQKTNGCAENCANGSDEEPERMGNKTEEDSGMEIEEAASSSSSTPLASPSVLKARKGRKSKSNSESRKTPSSSRVTRSSGKQPTIVAMFSKGSNKRKSEEVNGKLKQETNLENEEEEEEEQFGEKEPDVKRFKTETKEEGLERKEEATQIKTGTPVKTTPPKCAECRQYLDDPDLKFFQGDPDNALDEPEMLTDERLSIFDANEDGFESYDDLPQHKVTSFSVYDRKGHLCPFDTGLIERNIELYFSGVVKPIYDDNPCLDGGVRARKMGPINAWWITGFDGGEKALIGFTTAFADYILMEPSEEYAPIFALMQEKIYMSKIVVEFLQNNPDVSYEDLLNKIETTVPPAGLNFNRFTEDSLLRHAQFVVEQVESYDEAGDSDEPPVLITPCMRDLIKLAGVTLGKRRAARRQAIRHPTKIDKDKGPTKATTTKLVYLIFDTFFSEQIEKTEKEEDNENVSKRRRCGVCEVCQQPECGKCRACQDMIKFGGSGKTKQACFQRRCPNLAVKEADDDEEVDDNIPEVPSPKKMLQGRKKKQSKSRISWVGHPVKSDGKKDYYQKVCIDSETLQIGDCVSVSPDEPTKALYLARITAMWEDVNSSEPMFHVHWFCRGTDTVLGATSDPLELFLVDECEDMQLSYIHGKVNVLYKAPSENWSLEGGLDVEIKMVEDDGRTYFYQMWYDQEYARFETPPKIQPTEDNIHKFCMSCIRLDEVRQKEIPRVLEPQEESEGKMFYSMAMKNGVQYRVGDGVFLLPDAFSFSLRLASPIKRQKKEAVNEELYPEHYRKYSEYIKGSNQDAPEPYRIGRIKEIYCNIRSSGRPNEAEIKLRVYKLYRPENTHKSVKASYHADINLLYWSDEEASVEFKDVQGRCTVVYGEDLTENIQDYSASSSDRFYFLEAYNAKTKTFEDPPYHARSSGNKGKGKGKGKGKSKGKSSTASEHSEHEAAKMKPPKLRSLDVFSGCGGLSEGFHQAEVSETLWAIEMWEPAAQAFRLNNPGTTVFTEDCNVLLKLVMSGEKTNSLGQKLPQKGDVEMLCGGPPCQGFSGMNRFNSRTYSKFKNSLVVSFLSYCDYYRPRFFLLENVRNFVSFKRSMVLKLTLRCLVRMGYQCTFGVLQAGQYGVAQTRRRAIVLAAAPGEKLPMFPEPLHVFAPRACQLSVVVDDKKFVSNITRTYSGPFRTITVRDTMSDLPEIRNGASALEISYNGEPQSWFQRQIRGSQYQPILRDHICKDMSALVAARMRHIPLAPGSDWRDLPNIEVRLSDGITTRKLRYTHHEKKNGRSSTGALRGVCSCAEGKACDPADRQFNTLIPWCLPHTGNRHNHWAGLYGRLEWDGFFSTTVTNPEPMGKQGRVLHPEQHRVVSVRECARSQGFPDTYRLFGNVLDKHRQVGNAVPPPLAKAIGLEIKSCVSAKIKEGATDTIKREKMDTSD

>Cpic_ENSACAP00000004041_1

SSLSPQECVKEKLNLVHGFLQADAQNQLSDLETRLHREELSEEGYLARVKALLSKELSVENGDAAALSQKSNGCSENGAYGSDEDSERAGHEGEEDSAMETEEAVASSSSSSATMSKTRKARRSKSNGENKKSPASSRVTRSSGRQPTILALFSKGSNKRKSDEVNGEVKQETNPEKEEEELEEKVRLAHV*KQILILMYFVPRSEIKEEATQVKTAPPAKTTPPKCVDCRQYLDDPDLKFFQGDPDNALEEPEMLTDERLSIFDANEDGFESYEDLPQHKVTSFSVYDKKGHLCPFDTGLIERNIELYFSGAVKPIYDDNPCLDGSGGVRAKKLGPINAWWITGFDGGERALIGFTTAFADYILMDPSEEYAPTFALMQEKIYMSKIVVEFLQNNPDVSYEDLLNKIETTVPPAGLNFNRFTEDSLLRHAQFVVEQVESYDEAGDSDEPPVLITPCMRDLIKLAGVTLGKRRAARRQAIRHPTKIDKDKGPTKATTTKLVYLIFDTFFSEQIEKNEREEDKENAMKRRRCGVCEVCQQPECGKCKACQNMIKFGGSGRSKQACLQRRCPNLAVREADEDEEVDDNIPEMPSPKKMLQGRKKKQNKSRISWVGEPIKRDGKKDYYQKVCIDSETLEVGDCVSVSPDEPSKPLYLARVTALWEDSSGQMFHAHWFCQGIDTVLGKTSDSLELFLVDECEDMQLSYIHGKVNVVYKAPSENWALEGGLDMEIKMVEDDGRTYFYQMWYDQEYARFESPPKLQPSEDNKYKFCMSCARLDEVRQKEILKVMEPQEEVDGKMFYGLATKNGVQYRIGDGVFLLPEAFSFSMKLASPAKRPKKEAVDEELYPEHYRKSSEYIKGSNQDAPEPYRVGRIKEIFCSIRSNGKPNEADIKLRLYKFYRPENTHKSVKASYHSDINLLYWSDEEVTLDFKAVQGRCTVEYGEDLTECIQDYSASGSDRFYFLEAYNAKTKSFEDPPNHARRAGNKGKGKGKGKGKGKGKSVSALEQSKQEAAEVKLPKLRTLDVFSGCGGLSEGFHQAGISETLWAIEMWEPAAQAFRLNNPGTTVFTEDCNVLLKLVMSGEKTNSLGQKLPQKGDVEMLCGGPPCQGFSGMNRFNSRTYSKFKNSLVVSFLSYCDYYRPRFFLLENVRNFVSFKRSMVLKLTLRCLVRMGYQCTFGVLQAGQYGVAQTRRRAIVLAAAPGEKLPMFPEPLHVFAPRACQLSVVVDDKKFVSNITRTYSGPFRTITVRDTMSDLPEIRNGASALEISYNGEPQSWFQRQIRGSQYQPILRDHICKDMSALVAARMRHIPLAPGSDWRDLPNIEVRLSDGITTRKLRYTHHEKKNGRSSTGALRGVCSCAEGKPCDPADRQFNTLIPWCLPHTGNRHNHWAGLYGRLEWDGFFSTTVTNPEPMGKQGRVLHPEQHRVVSVRECARSQGFPDTYRLFGNVLDKHRQVGNAVPPPLAKSIGLEIKSCVLAKVKE

>Tgut_ENSTGUP00000014140

RKLGPINAWWITGFDGGEKALIGFSTAFADYILMEPSEEYAPTFALMQEKIYMSKIVVEFLQNNRHVSYKDLLNKIETTVPPAGLNFNRFTEDSLLRHAQFVVEQVESYDEAGDSDEPPVLITPCMRDLIKLAGVTLGKRRAVRRQAIRHPTRIDKDKGPTKATTTKLVYLIFDTFFSEQIEKDEREEDKENATKRRRCGVCEVTEEQPECGKCKARSNMVKFGGSGRSKQACLQRRCPNLAVREADEDEEVDDNIPEMPSPKKMLQGRKKKQNKSRISWVGEPIKVRGTSSVPRKVCLYLEPIQVTEPIQVRGTHPGEPTQVRGVTAMWEDSSGQMFHAHWFCPGSDTVLGATSDPLELFLVDECEDMQLSYIHGKVNVIYKAPSDNWAMEGGLDTEIKMVEDDGRTYFYQMWYDQEYARFESPPGAQPTEDNKYKFCMSCARLDEVRHKEIPKVAEPLEEADGKMFYAVATKNGVQYRVGDGVYLLPDAFSFSVKPASPAKRPKKEAVDEELHPEHYRKYSEYIKGSNLDAPDPFRVGRIKSIFCSLRGNGKPNEADIKLGIYKFYRPENTHKSTKASYHADINLLYWSDEETTVEFRAVQGRCSVVYGEDLTESIQDYSAGGLDRFYFLEQLQVPKLRTLDVFSGWGGLSEGFHQAGVSETLWAVEMWEPAAQAFPVPIPDSHSHSQCPFPFPVPIPQGCRKRSGPDQPRAFGVAILRKKRPCQGYMTTVRGRSYLSMHSQYFTGFLSYCDYYRPRFFLLENVRNFVSFKRSMVLKLTLRCLVRMGYQCTFGVLQAGQYGVAQTRRRAIVLAAAPGEQLPMFPEPLHVFAPRACQLSVVVDDKKFVSNITR

>Ggal_NP_996835.1

MPARSAPPPPALPPALRRRLRDLERDEDSLSEKETLQEKLRLTRGFLRAEVQRRLSALDADVRCRELSEERYLAKVKALLRRELAAENGDAAKLFSRASNGCAGNGEEEWERGGRGEDGAMEVEEAAASSSSSSSSSSSSSSSSSSSSSLLPAPRARKARRSRSNGESKKSPASSRVTRSSGRQPTILSVFSKGSTKRKSEEVNGAVKPEVSAEKDEEEEEELEEKEQDEKRIKIETKEGSEIKDEITQVKTSTPAKTTPPKCVDCRQYLDDPDLKFFQGDPDDALEEPEMLTDERLSIFDANEDGFESYEDLPQHKVTSFSVYDKRGHLCPFDTGLIERNIELYFSGAVKPIYDDNPCLDGGVRAKKLGPINAWWITGFDGGEKALIGFTTAFADYILMEPSEEYAPIFALMQEKIYMSKIVVEFLQNNRDVSYEDLLNKIETTVPPVGLNFNRFTEDSLLRHAQFVVEQVESYDEAGDSDEPPVLITPCMRDLIKLAGVTLGKRRAVRRQAIRHPTRIDKDKGPTKATTTKLVYLIFDTFFSEQIEKDEREDDKENAMKRRRCGVCEVCQQPECGKCKACQNMVKFGGSGRSKQACLQRRCPNLAVREADEDEEVDDNIPEMPSPKKMLQGRKKKQNKSRISWVGEPIKSDGKKDFYQRVCIDSETLEVGDCVSVSPDDPTKPLYLARVTAMWEDSSGQMFHAHWFCPGSDTVLGATSDPLELFLVDECEDMQLSYIHGKVNVIYKPPSENWAMEGGLDMEIKMVEDDGRTYFYQMWYDQEYARFETPPRAQPMEDNKYKFCLSCARLDEVRHKEIPKVAEPLDEGDGKMFYAMATKNGVQYRVGDSVYLLPEAFSFSMKPASPAKRPKKEAVDEDLYPEHYRKYSEYIKGSNLDAPDPYRVGRIKEIFCHIRTNGKPNEADIKLRIWKFYRPENTHKSMKATYHADINLLYWSDEETTVDFCAVQGRCTVVYGEDLTESIQDYSAGGLDRFYFLEAYNAKTKSFEDPPNHARSSGNKGKGKGKGKGKGKGKSSTTCEQSEPEPTELKLPKLRTLDVFSGCGGLSEGFHQAGVSETLWAIEMWEPAAQAFRLNNPGTTVFTEDCNVLLKLVMSGEKTNSLGQKLPQKGDVEMLCGGPPCQGFSGMNRFNSRTYSKFKNSLVVSFLSYCDYYRPRFFLLENVRNFVSFKRSMVLKLTLRCLVRMGYQCTFGVLQAGQYGVAQTRRRAIVLAAAPGEKLPMFPEPLHVFAPRACQLSVVVDDKKFVSNITRTYSGPFRTITVRDTMSDLPEIRNGASALEISYNGEPQSWFQRQIRGSQYQPILRDHICKDMSALVAARMRHIPLAPGSDWRDLPNIEVRLSDGTSTRKLRYTHHEKKNGRSSSGALRGVCSCAEGKPCDPADRQFNTLIPWCLPHTGNRHNHWAGLYGRLEWDGFFSTTVTNPEPMGKQGRVLHPEQHRVVSVRECARSQGFPDTYRLFGNILDKHRQVGNAVPPPLAKAIGLEIRACVGARMREESGAAVAPPAPEKMEMTAAAD

>Btau_XP_015327477.1

MPARTAPARVPALASRAFSLPDDVRRRLKDLERDSLTEKECVKEKLNLLHEFLRTEIKNQLCDLETKLHKEELSEEGYLAKVKSLLNKDLSLENGAHAFSREANGCLENGSQTSGEDCRVVMAEKGKPPKPVSRLYTPRRSKSDGETKSEVSSSPRITRKTTRQTTITSHFPRGPAKRKPEEEPEKVKSDDSVDEEKDQEEKRRRVTSRERVAGLLPAEEPGRVRPGTHMEEEGRDDKEEKRLRSQTKEPTPKHKAKEEPDRDVRPGGAQAEMNEGEDKDEKRHRSQPKDLASKRRPEEKEPERVKPQVSDEKDEDEKFWRIQFTYQSTSREEKRRRTTYRELTEKKMTRTKIAVVSKTNPPKCTECLQYLDDPELRYEQHPPDAVEEIQILTNERLSIFDANESGFESYEDLPQHKLTCFSVYCKRGHLCPIDTGLIEKDVELLFSGSAKPIYEDDPSPEGGINGKNFGPINEWWIAGFDGGEKALLGFSTSFAEYILMDPSPEYAPLFSVMQEKIYISKIVVEFLQSNPDSTYEDLINKIETTVPPCMLNLNRFTEDSLLRHAQFVVEQVESYDRAGDSDEQPIFLSPCMRDLIKLAGVTLGKRRAERRQTIRQPAKEKDKGPTKATTTKLVYQIFDTFFAEQIEKDDKEDKENAFKRRRCGVCEICQQPECGKCKACKDMVKFGGSGRSKQACQKRRCPNMAMKEADDDEEVDDNIPEMPSPKKMHQGKKKKQNKNRISWVGDAVKTDGKKSYYKKVCIDSETLEVGDCVSVIPDDSSKPLYLARVTALWEDSSNGQMFHAHWFCAGTDTVLGATSDPLELFLVDECEDMQLSYIHSKVQVIYKAPSENWAMEGGVDPEALMSEDDGKTYFYQLWYDQDYARFESPPKTQPTEDNKYKFCASCARLAEMRQKEIPRVVEQLQDLEGRVLYSLATKNGVQYRVGDGVYLPPEAFTFNIKLSSPVKRPRKEPVDEALYPEHYRKYSDYIKGSNLDAPEPYRIGRIKEIFCSKKSNGRPNETDIKIRVNKFYRPENTHKSTPASYHADINLLYWSDEEAVVDFKAVQGRCTVEYGEDLPQCLQDFSAGGPDRFYFLEAYNAKSKSFEDPPNHARSTGNKGKGKGKGKNRTKSQTCEPSELETEIKLPKLRTLDVFSGCGGLSEGFHQAGISETLWAIEMWDPAAQAFRLNNPGSTVFTEDCNVLLKLVMAGEVTNSRGQKLPQKGDVEMLCGGPPCQGFSGMNRFNSRTYSKFKNSLVVSFLSYCDYYRPRYFLLENVRNFVSFKRSMVLKLTLRCLVRMGYQCTFGVLQAGQYGVAQTRRRAIILAAAPGEPLPLFPEPLHVFAPRACQLSVVVDDKKFVSNITRLSSGPFRTITVRDTMSDLPEIRNGASALEISYNGEPQSWFQRQLRGSQYQPILRDHICKDMSALVAARMRHIPLAPGSDWRDLPNIEVRLSDGTLARKLRYNYHDKKNGCSSSGALRGVCSCVEGKPCEPAARQFNTLIPWCLPHTGNRHNHWAGLYGRLEWDGFFSTTVTNPEPMGKQGRVLHPEQHRVVSVRECARSQGFPDTYRLFGNILDKHRQVGNAVPPPLAKAIGLEIKRCMLAKARESASGHIKVMFGGFLDGLVVKNLPASAGDTDSIPDPGRSHMLQSN

>Pbac_AFV53350.1

MVRNDAIENQENLPPNSKRRKTDKVENLKEPPRKQVVKPLKCGVCRQLQDKDVLKTFPGDPPGAVEEFIALASDILSLGQTEEGDWEEKPQHRITEFSVYDEFGHLCAFDTGLIDNNVPLYFSGYIKPIYDDTPSTEECGVWASRCGPIDAWYVTGFDGGERELIGFTTVYAEYLLLHPGPEYRSIYDGVREKSFLSKTVIEILEGEPEQSLEDLIQRVQQVIPPFGSSRTGGLTEEDLLKNAQFLLEQIESYEMAADEDEDQLKMTLPAVTGLMSLAGADQIDRKEPKTKRRNKTTGNKPSGPRKTKSTDTSHATVTPLVQATFEGLFFDQLSHADTVEADGGPRKRCGVCVGCKAEDCGTCPTCKDKVKFGGSGKRRQACINRGCVDMQPKEEKDKPVKDSKSRTKTVRSKMQISGDTDGEFTGEVLKTKGGKTYYSTATVEGYSIQLGDYVTLQAEDDSGQEGQWVARIVAMFKRGNVETLHVQWFSSSSDTVLGDCSHDPSEIFITDTCDNVPLGSVTSKLDDISYRPPPDNWNEIGGLPVAKKEENTTLFYRLKYEHSKGRFEVPPPEYLNWKPTEPCPVCTRSKLHDPAPRIEDGVVVAGGERVKVGGYLYLSLDAYTMPNRKRKMASRARKDSVDEDMYPEFYRKTDYIKGNNADVPDPFRVCRLVAIDNQNTVTVRKLYRPENLDKGVCCAEESDINLLYYSAEMVSVSVDKIVGSCTVRHGADIPDLAKYSSRTDHFYFTEQWNAKEKVLEDPPGDVRLSNKGPAKQKVVPEIKPRKLRTLDVFAGCGGLSCGFHQAGVAESKWAIEFVSEAAAAYKLNNPKTTVFNEDCNKILKMAMEGREVDDLGQRIPTKGDVELLCGGPPCQGFSGMNRFNAREYSQFKNSLIASYLSYCEFYRPRFFLLENVRNFVSYKRNMVLKLCISSLVNMGYQCTFGVLQAGSYGVAQTRRRAFILAAAPGEILPQFPEPRHVFSHAGMSLSLTVDGKRFGSHITRFTSAPLRTITVRDTMSDLPAVGNGASVTELAYNSEPTTWFQRQIRGNTTTLTDHICKEMHPLAAIRTKHIPLTPGSDWRDLPNIAVNLPDGTRAPKLIYTHDDRKNGPLRGVCSCAEGRSCDPSDKQNNTLIPWCLPHTGNRHNHWAGLYGRAFWDGFFSTTITNPEPMGKQGRVLHPEQHRLVSVRECARSQGFPDSHTFYGNVLDKHRQVGNAVPPPLAKAIGEEILKYAFRFEG

>Skow-DNMT1-prot-modelPK

MPSKVDDSIPEEVRGRLAKLQKDYEAGSVKDKNFAEQKSELLLEFLPETLQKSLTTLKEDLKDECLTEKGYCKKVLSLLSQYLTDLPAVSTKNGLSESSQSESDSGCQSQTDQSTDQPDGDISFETPECTSQSDMSSKETSQPDEASSQLTADETSQSDMETDQPDGKANGQSMKRNKGRPKGPQLKKKKSPARGKTSDTSDVDDTSPQSSKKSKIKKDSKQPNIALMFAKTPSKRKTSEQSDEAEGGFNCAGVKSEHQHEKRMKIGDGSETFDDDEDEKKFKKDLRPKTENDEISAVAPPVRCTECRQLLDDPDLKTFPGDPDDSVDEFIMLTDPRLSLFTGDEEDVTGYEDRPQHKLTNFSVYDKNTHLCAFDIGLIEKNKELFFSGHVKPIYDENPSIEGGIATKAMGPINEWWTAGFDGGENSLIGFSTAFAEYILMTPSECYAPIYNSMKEKIHMSKIVIEYLQNNQFSTYEDLMNKIQVSVPPANCSTFTEDTLLRHAQFLVEQVESYDEAGDDDEFSLLTTICVRDLIKLSGVTLGIRRAARKAIKKVTKKDVPKHTKATTTVTVRNIFESFFRDELDNEKTELPTRRQRCGRCEVRCVQRLVMKKFGGSGRSKQACQERRCPNMAIKEVEDDECEDLERNDDENQKKPTKKITTPIKKGNRKTKMKWIGKPIAEENKKKYYRSVRINDEEISIGDAVSVSPQDPTKPVLLAFVTYMWEDGNKDQMFHAKWYCRGVDTVLGEMADPLEVFAVDECEDTALEYVMGKSKIMYKAPPENWSMLGGEEVENFITDDDGNTFYYQKWYDPDCARFEDIPVTQRPDDMPSHRYCDSCQRMEQIRKNETPHVSEKLEEDKQNPKVVYYSVLSKGGLDYRVGDSCYLLPGSFYFNVKPAPPPSKVSRKENVDEEVYPEYYRKTSEYVKGSNIECPEPFRIGRIIKIFTKKSPNKLVEEIKLKVVKFYRPENTHKGVTASYTSDLNLLYWSDEEAEIAAYDIQGKCTVMFEEELNESVQEYTRKGRDRFYFLEAYCSKSKEFEEPPSRARTIKGKGKGKGKGKSSTPVIEVKQVAAVKDNFQYRKLRSLDVFAGCGGLSEGFHQSGVAESTWAIEKEEPAAQAFRLNNPGCTVFTDDCNTLLRLVMDGETTNSVGQKLPQKGQVELLCGGPPCQGFSGMNRFNSREYSKFKNSLVVSYLSYCDYYRPKFFLLENVRNFVSFKKSMVLKLTMRSLLSMGYQCTFGVVQAGLYGVAQTRRRAIILAAAPGEKLPFYPEPAHVFSPRACPLSVTIDEKKYESNMARVWSAPYRTITVRDALSDLPEIRNGYNKLEMSYDREAQNGYQKAIRGSQHQPILRDHICKEMSALVEGRMRHIPLAPGSDWRDLPNIAVRLSDGNTCKKLRYLHPDKKNGKGPNGQLRGVCACAMNKPCDPTDRQFNTLIPWCLPHTGNRHNNWAGLYGRVEWDGYFSTTVTNPEPMGKQGRVLHPEQHRVVSVRECARSQGFPDTYRFYGTILDKHRQVGNAVPPPLAKHLGLEIKKCAEVSQRHQQTLEDKK

>Adig_4714

MVQLKDDFPPAISERLSELEDEYNDGDITEKGYVRKKCKLMKPLLANFQQERIQEIEDDLKAGKFSEEQFISHLKELLTEIGHRSSNGCGAKPLPPVCNKDSNNVTLNAEPMETGMEINASDIPYTQASDCHEMEEANDSEGEPSSQSEPGLRQSSCNNSNEPTSSNGAERSQCLLDIKVSLTDLKKSTSPTNKCSQEGNKIKVNKKESKKQPGIKEMFAKNALKRKKTEDSSTDDSAGQAELSSENSEASNYSQDGKRQKTSGDDCDEEESSGDSGIERQSLRESTVGIRSEEKRAQPPAKCKECKQLLNSPDLRLFPGDSNDAVEEFVALTDPRLSLFSGEEEQCDSYSDTPQHKITNFSIYDKNTHLCPFDSGLIEKNVELFFSGYLKPIYDENPSPEGGVPTKNIGPINEWWVAGFDGGENALIGFTTAFAEYILMQASEDYMPFMNIMREKIAMSKIVVEFMQSNPEARYEDLLNKVETSVPPANCSTFTEDTLLRHAQFLVEQVESYDQAADDDELPLLISPCMRDLIKLAGVTLGKRRAARGVKVRTEKKQARPSKATTTPLVRQIFDIFFKDQIDGKEIGAIRRKRCGVCEVCQLPDCGKCKSCKDMVKFGGTGKKKQCCEERRCPNMAVKEADEDDDIGEDIEDAENVTLNKRITKKKSPTPKKTKSKSKVQWIGDPEVHGKQNYYTSVLIDKEEICVGDFVMFRPDENSHLALYIACVRYMWEEPNGDKMFHCRWFSRGSETILGETSDPREVFLLDKCDDNLLGCIKQKCTVTYNKPDADWFMKGGLDEPEGDVSMEENDGNTFFFQKWYDPDHGRFTDPPAEYLHVVNSEDNFRYCESCVRQSAEENLETLSLGEPLEVDSKPSSKSYYKSCSKGGNRYKSGDCVFLEPDAFSFNVKPKESKKGFKKDDMVDEEKYPEYYRKPLEYVKGSNYDVPESFKIGRIINIFTKSSPGKLSKDLDIMLTVGKFYRPENTHKGSSFAYQADLNLLYWSKEEATVPFDAVLGSCTVTCGEDLNCSIAEYTAKAINNFYFLEAYNSETKDFEEPPLEARNAALKGKGKGKGKGKARSACQTEEQNLRQDSKPSETVPKLRCLDVFAGCGGLSEGLHQAGTAESLWAVEKEEPAAHAFSLNNPGCTVFTDDCNLLLKLVMDGEKKNSRGQTLPQKGEVELLCGGPPCQGFSGMNRFSSRDYSQFKNSLVVSYLSYCDYYRPRFFILENVRNFVSFKRSMVLKLTLRCLIKMGYQCTFGVLQAGCYGVPQTRRRAIIMAAAPGEVLPLYPEPTHCFSPRAIQLTVMVDDKKFESNITRLSSAPFRTITVRDSMSDLPEIRNGASNAEISYSGDSISHFQRQVRGSQYQPVLRDHICKEMNPLVAVRMRYIPLAPGSDWRDLPNIEVQLPDGTKTKKLAYTHHDKKNGRSSEGQLRGVCSCAEDKPCDPADRQFNTLVPWCLPHTGNRHNHWAGLYGRLEWDGYFSTTITNPEPMGKQGRVLHPEQHRVVSVRECARSQGFPDTYRFYGSILDKHRQVGNAVPPPLAAAIGREIKKSLQATQQNA

>Pdum_DNMT2_comp216256_c0_seq1

MEKLKVLELYSGIGGMHFALKECGIPHEVVAAADINNVANDIYKFNFKGTKLLQRTIESLSLKELNKLQMDMITMSPPCQPFTRVGLQKDAGDQRTKSFFHVLDLLPKLDKQPKYILLENVKGFDTSATREVLLETLNEAGYHVQEFLVTPLQFGVPNSRLRYYLLAKRKELVFSFPMSDEIITDLPACADQFLHHKVKDGDIKEMPDDGRWDLSAFCTCVDQSSSGELCAKCMKLRYVSKKRKTEMDNEAEKSRLVAKEKVENDVDPCNHSTDSPNDQSSTKKVDSDSSCSAKDKSSSFTGESCTDDRINSTNSTEKTDSGDSIDDSCSSVNKSHDLKSQCSRNENDSNGQHGHISNSDCGASLSPNNSEELTGAQGGPNHSKMDVGVKSNQSNCAGLSQTEGAQKVRTLTPSEIAYVKYRSICRPVSDYLETQPEEYFKDYLVPEKFFKRFWVMDIVKPHARNSCCFTKRYAHHLEGSGSIIQMDLDNSVDLRAYKIERQYNETVAEGVRSMKLRYFTPREISNIMCFPPWFDFPEQFNLIQKYRVLGNSLNVHVVAVLMRLLMLGDDES*

>Mmus_NP_034197.3

MEPLRVLELYSGIGGMHHALRESHIPAHVVAAIDVNTVANEVYKHNFPHTHLLSKTIEGISLEDFDKLSFNMILMSPPCQPFTRIGLQGDMTDPRTTSFLYILDILPRLQKLPKYILLENVKGFEVSSTRGLLIQTIEACGFQYQEFLLSPSSLGIPNSRLRYFLIAKLQSEPFPFQAPGQILMEFPKIVTVEPQKYAVVEESQPRVQRTGPRICAESSSTQSSGKDTILFKLETVEERDRKHQQDSDLSVQMLKDFLEDGDTDEYLLPPKLLLRYALLLDIVKPTSRRSMCFTKGYGSYIEGTGSVLQAAEDAQIENIYKSLPDLPPEEKIAKLSMLKLRYFTPKEIANLQGFPPEFGFPEKTTVKQRYRLLGNSLNVHVVAKLLTVLCEGFGNASESCHKMPLILDSNSKILS

>Hsap_NP_004403.1

MEPLRVLELYSGVGGMHHALRESCIPAQVVAAIDVNTVANEVYKYNFPHTQLLAKTIEGITLEEFDRLSFDMILMSPPCQPFTRIGRQGDMTDSRTNSFLHILDILPRLQKLPKYILLENVKGFEVSSTRDLLIQTIENCGFQYQEFLLSPTSLGIPNSRLRYFLIAKLQSEPLPFQAPGQVLMEFPKIESVHPQKYAMDVENKIQEKNVEPNISFDGSIQCSGKDAILFKLETAEEIHRKNQQDSDLSVKMLKDFLEDDTDVNQYLLPPKSLLRYALLLDIVQPTCRRSVCFTKGYGSYIEGTGSVLQTAEDVQVENIYKSLTNLSQEEQITKLLILKLRYFTPKEIANLLGFPPEFGFPEKITVKQRYRLLGNSLNVHVVAKLIKILYE

>Aque_2.1.21062_001

MAEIISEIIEEIVVKKMQSKTDPEEPLPSKSLGVVEFYSGIGGWHYAIRETGLNLKILAAVDINTTANQVYKHNFPNTKVLQRNILGLSAQELDSFLANLFTLSPPCQPFTRQGKNEDDIDYRTDSFFHLMGILSAMQKPPQYIMMENVKGFELSRTRGHFVHVLKELGYTFQEYLISPKQFGIPNSRLRYYLLARLSPRNFKAAPLQDTVEHLKYYIPTSFHDIHLKCDISSYLDDLSEIDVQQFLVPDRILEKYAQGLDIVSAKSHSSCCFTRGYYHYAVGTGSVLHHDCSVDLALAYQCYAEQKNNCDGIKSLKELKLRYFTPQEVAKLMSFPASHTFPTSVSNKQCYKLLGNSVNVFVVATLLCYLCSQ

>Mlei_ML074813a

MSPAASSTLRVCEFYSGIGGYHIALSQIPHLSFEVAAAFEISSNANCVYRANFPNTTVLETNLCGLTATRLDSIIRKSIGSDSQDEAVTSRDEVMFVMSPPCQPFTRQGIQKDDLDPRSESVLHLFETFSQLTSLPDYFILENVRGFETSNTRNRIMQFFTKHQYRVAEFLINSNQVGIPNSRLRYYLLARKKPFTKAVESALDSSQGFHKIPRLLTSIPGFEVELPTQPLSEYLEPGLTGYDLSDKFLSKWGWVLDLVTPSSTRSCCFTKGYSVKAEGSGSVLQQLSDPSESVGQLSLDGFSGNGGNNERSMCNSRLSKEEFLEHMKSLRLRFFSPREIARIHGIPDSYSFPEVLSEKQLYKLLGNGLNVSVVRLLIEKVLLE

>Nvec_132811

MADSSTFRVVEFYSGIGGMHYALKGCKKNAEVVAALEISTTANTVYGHNFPTTKIWNCNIEVCELCNVTTMPAIYMVMSPPCQPYTWVGLQGASKDPRALSFLHILSLLKRLQHPPKYWLIENVKGFETSDTRFYILLAFCNSFIVSSPQFGIPNSRLRYYLLAKRHPLTFSTAMGNKFSYYEQFLLPTKVLSRFSLVLDIVTAKSRRSCCFTKAYGHYAEGTGSV

>Aipt_Q54JH6

MAAICEKSSDSTFRVVEFYSGIGGMHFAAKGCGFKTEVVAALEINNTANVVYKHNFPDVKLLQRNIEXLTVKDLDGLAADIFLMSPPCQPFTRVGLQGDSSDPRTKSFLHLMDLILRMSRPPDYLLMENVKGFETSHTRSSFYHRISLEFPTQGLDITCLPRENLINFTLKHNRRNRRILAEFLEPEHDDYFQQFLLPERVLSKFALVLDIVTPFSTHSCCFTKAYGHYAEGTGSILKMADIDDVEYFDKYRSLDPGQSDSQRAEVLRDLKLRYFTPREVANIMYFPKSFGFPEGTTSKQKYRLLGNSLXVHVVTELLRCLLHNS

>Hmag_2.233535+2.218066

MGPSLKVLEFYSGIGGVHYALTYAGVSVHVEAAFEINTSANSVYRHNFPQTTLLQKNIEGLNLEDLEHFNADVWTMSPPCQPYTRLGKQEASCDPRAKSFKKLMNILMKMTSPPSFIFLENVKGFEVSDSCEDFLMILNSKGYYTQSFLLSPIDFGIPNSRLRYYLIARYQKKFNFKTTQKPICLNGNLCECEGLDMECACTALASVVQLYKPMKIKDILNVDNSVSSEINSSSFCEPDLKKMLDLLQKRSFPIKFTKISHSILLRYYSLFDIVDSDSNKSCCFTSGYSRFIEGTGSLFSCLPQSDRNLILNKLSITENDNESNIDLLESLQLRFFSPEEIAALLCFPICFSFPDKITEKQKYKLLGNSVNVLVVANVMRYFLFENKIXMTSTWKELLQHHEDDDQFLACQGVNIFKQLNDHVNPSEQKTESPQNNISVENMTPKKTTPKKILTEEIPLKPIFNEKFNKYLQKKKISLEGITKPSTRSKLKAEILSVPSKNKRKARIESEKLSAHAKKNKTVVRRVRCRSCDGCLQKNCDKCRYCLDMIKNGGPGLLKQSCARRFCQNPKLPAYVECYICHNGHKEDERILMECSICSEIVHPECLKKSSPCKMSKKVNNCWECPKCCTDKKAEKFSPQKARGHMSDFKRKQMVFKQDLSNEPRTEEHRHQPIKSLISNTKPLSNKQMINKVQNLKEILNKSKLCVDKKIVVDIKGKNGHSKEMISKGNKNKVTCNDLDSNIELRKLKAPKHKSFDKVTENIRSTLTTHAPIMVVRPGPFTQLETCIMSDGEKHPLDRKLWVQVFVYLNKNDISSCMLVSKAWNQWCLDNCLWSEIDISNRNLSISMLCGIVRRQPSLLKLSSTNPTAKQLEWLLKRLPRLEGLNLSLSTAPTISAIMRVDCSQLKYLNLSWSSAIYDKLMIQLLGPIKLYPVYCVERRLPRLSELILTGCDISDETTTYIFSNLKNLRRLDISYCSRVTVVGVEVLIRNEHVSKDVLQEVLCEGCSFSENLVSFFRSMNIRLIECFVHARAIPRSFTIGVRVAILFSDSDSAENVATPTPTPQPCVQQKSFYX

>Tadh_49657

MSSARGQVMQVVEFYSGIGGMHYALQESNINAKILAAIDINTVANNVYRHNFGNTPVWQREIGKISLKELQELNGDLYTMSPPCQPFTRLGKKADVNDARTSSFLHVIDLLIKMENPPKYILLENVKGFETSAARNEFLLTPLQFGIPNSRLRRGYDGTSEFRPNVVKPDSQRSCCFTKSYFHYAEGTGSVLQITNPILHLKLRYFTPREVANIHCFPVHFNFPENATKKQCYRLLGNSLNVHVASELLKLLIT

>Cgig_XP_011450725.1

MRVLELYSGIGGMHCAVKECGISYEIIAAIDVNTTANKIYKHNFPESNLMDCGIESLSATQLDEMKIDMIVMSPPCQPFTRVGKKLDAEDIRTKSFIHLLSLLPRLNNCPKYILVENVKGFEDSETCLKLRETLMKCNFTYQEFLLTPLQFGIPNSRLRYYLIAKRSPLKFSFANTTEIMTSLPSINSEENSSSTNATNISQPSTACISDESTRTNDHCKNSSHISSSHHMQSSPAKPAKNKETQHGMLGKSINNSDATEKKSPDIPAATCPICRESCLQSAGHNMRQLSEGEEEEEDKYPCCLVQDFLEDQPPEYFQPFMLTRKDLKRFIVMDIVFPCLQKTTCFTKRYGHFMEGAGSIFQMSHSISEMTAASELKDRTLELKNRDQWSDEDYQVLSRLRLRYFTPREIANFLCFPATYNFPQDLSKIQLYRTLGNSLNVRVVSKLIQLMVKEQTSHVQV

>Lgig_XP_009052047.1

MAASIELRVLELYSGIGGMNYSLKESGVKYEIIAAIDINTTANQIYQHNFPDHNLMAFGIEKLTLKQYEKWNINTILMSPPCQPFTRVGNKNDVNDIRTQSFLHILQLIHQCKEKPSYILVENVKGFEESEARNKLIEMLTTNHYHYQEFLLTPLQFGISNCRLRYYMIAKLQTVQFLFTPSSKILTSIPNCPEEWLKYLQCSEETDNPVSNKWDQFESCDQNIHQEDIETDYICDKCLKLKYFLESKSDDYFQDYLVEKKDFKWFIVMDIVHPGLKKSMCFTKRYGHYINGAGPILQMSHDLTCDLKTKITTLNSRDFWTEEETKIIEKLKLRYFTPREIANLLCFPSSFSFPDNLSRIQLYRCLGNSLNVHVVSILIRLLVLEKYAS

>Bgla_XP_013095826.1

MKLKVLELYSGIGGMHFALKESHLEYEIVLAIDINTTANKVYKHNFADTNLQSFGIEKLSLKMLEDLQINTILMSPPCQPFTRVGKKRDCHDVRTKSFLHILDLIKLIKVKPVYILVENVKGFETSETRGQLIECLKYCNYTYQEFLLTPLQFGIPNSRLRYYLTAKWNQCLMTTATGNYSILTELQDFSSFLKDSVTAHEQEAVKNTNFLVNDNSESLKPICARAQSSDLQGVKTQDSQQSSDSLQNEAKSSLKDFGRNMRYCEEDDKFINDKSLSLSEYMETSDNIDLQDYWLSDKELRMFVIMDVVYPELKKSICFTKRYGHYIEGAGSVVQMSSDIQHVIAASDLKHQAVNSLNRNEWGESEMEILRNLHLRYFTPREVANLLCFPKDFSFPDGLTKVQKYRVLGNSLNVFVVTQLIQFMTKIL

>Acal_XP_012941472.1

MVSTMQPKCLRVLELYSGIGGMHYALKESGADFEVVMAVDINTSANSIYKHNFPDTDLHSFGIEKLTLKRLENMNIDAILMSPPCQPFTRVGKKRDCDDVRTKSFLHLLQLLRQLKRKPNYLLLENVKGFEVSLTHAQLMSCLDDCGYHYQQFLLTPLQFGIPNCRLRYYLIARLDQPFSFPMEKEIMTTLPRVGSPVGVSSAPVTSSESNSCSPTQELQGSSGVSVGVDATVSSQQSQVNDHDNGNNNSEQTHRVGDSGDDAPSSSQTVGDRVCQEVCGSNIDSGTSEVCSAKVSRLEVEKDSSDRSQGQGNNWDQTYHTATSSCDRSNNTKIDGAVETGPGDNISTRLCAQDCDSVSDIEALKQAGRNMRYCEEEDPLIGAHSRPLSQFMEQGGEEDFSQYLVPDRELHMFVVMDVVFPVLKKSTCFTKRYSHYIEGAGSVVQMTTNVQDVAAACEFKAKAVKMKNRSEWGTKELQILRNLKLRYFTPREVANLLCFPPEFDFPAGFSPLQLYRVLGNSLNVHVVAMLFTLMTRTV

>Obim_XP_014773951.1

MPFASQKIMAITFSADETTLAFFEAESNIPHEVVAAIDINPVANKIYSYNFPHTNLLETGIEGLTADWLDHLDFNVVLMSPPCQPFTRVGKRLDTKDSRTRSFLHFLDCLQKMKCPPSFILLENVKGFEVSETRHLFLKTLKACNYKYQEFIFTPLQFGIPNSRARYYLIAKKHPGKFYFDLTDEVQEELPACSKQWLHHIEPSEDCGETTCLSQDKDINCLQTCKTEKEEIRDFLDYYNKYSTILHQEEFENGISYPNCKALNDFLETNQSKEYYDQFLVPEKYFSLFVILDIVCPHLRKSVCFTKRYGHYIEGSGSIIQMSTDGQSVKDAYDLKMKTQQLKNRKQWSEEEFNVVRKLKLRFFTPREIANLLCFPHTFDFPPETTLRQKYRVLGNSLNVHVVAVLIKLLSQDCNLML

>Ctel_108989

MGDPLRVLELFSGIGGMHYALQETGINHEVIAAADINTVANDIYKHNFPDCLLMNRCIESIQLSEFSRLRPDLITMSPPCQPFTRVGKQRDIDDPRTKSFLHLLKVISTLERSVKYIFVENVKGFDGSEAHRMLLETLQAADYVMQEFLISPLQCGIPNSRLRYYLMAKKKPLKFQFDTTSQIMTELPACAASYLNHCQSKPDSTASSSVPLYDRCAAMCRPLSSYLQEDMSHDLLPEKFVHRFWVMDIVKPSSTNSCCFTKRYGHHIEGAGSVLQTNTDCEIDLTEYKKTKVYTAETEAAVKQLGLRFFSPREIANLMHFPAHFSFPANFSTVQTYRVLGNSLNVHVVAVLMKLMLIEK

>Hrob_89038

MVLKVLELYSGIGGMHFALNESGIKHEVVAAIDINPVCNAVYRHNFPNTKLIEKCLTSIFLKDFNKMSADMILMSPPCQPFTTIGKQKDVHDERTKSFQYFLSILPKFSKLPGYILMENVKGIESSIMRGEFLKVLSQLNYDYQEFILDPTQFGIPNSRRRYYLLAKLQNVNSSLQTRPDITYSLLRQNDNSTTNISQTDDKSVLRPLRDFLEKQLPEYFEKFRLPKKLYKKYWLMDLVSENSKSCCCFTKGYGRYVEKAGSIFQTSPSEVDLFPYKNHESDEQFVAEVDKLGLRFFTPREIANFLCFPSTFEFPANLNIQQQYRVLGNSLNVHVVSMLLKLLCS

>Avag_GSADVT00036986001

MVLRVLELFSGMGGMHSALDLIEIEYEIIAAIDINPMANLVYSSNFPQVPIINRSIETISIKQWEKWHADLWTMSPPCQPFTRQESKPKYILLENVFGFQDSDAHRLLLETLQLNNYSYEQYLLSPIQFGIPNSRLRYYLIAKQQENPIDTHSSIIHTQISNFERLLTKLHEQFPSKDISSHDETSRHRNDQITDQCSHSSEILIDPNQSMIINDFILSDENAEFELDDKQLKRGLNVCDIITCYAKRSFCFTKSYGKYFEGTGSLFRTQNDHIRYFTPKEIANLHCFPSTYNFPESITTRQAYSLLGNSLNVFVIGVLFENLFER

>Lana_XP_013412655.1

MCERVIEFYSGIGGMHLALKESSIKYKVVAAVDINDVANEVYKYNFQDINLLQRSIESISLKELNKFNPTMLLMSPPCQPFTRVGLQKDVEDARTNSFIYLLETLPKLEKLPNYILIENVKGFECSQTRDKMVRTLRECGYNFQEFLLTPLQFGVPNSRMRYYMVAKKKPLTFVFDTCEDKVFEHIPSCAKPFMTHMKPSGPGDHMVCCYCQNPIKKTDILGSSADTQKNGQTGNNLTDFDKINDTEDSTNEEPKAKAPRLDSGTVNSGNSNEDKDSGDITASKRQSCEKCTLSAGVIASNQDYSPQFCQSIDHYLEKQPEEYLEKFLLPEKDLKRFKVMDIVLPSSRKSCCFTKRYGHYMEGAGSVFQMVLEEMNTSILKDDMIPEKAVEYMDDIKKLKLRYFTPREIANLMCFPKEFEFPNKLSRKQCYRVLGNSLNVHVVAVLIKLMTMNVVQKNDS

>Lana_XP_013412300.1

MAWQGSPLELKRMNHLLQQSRASGVYCRWLATLQRSHKISTAATACTPQKDEKDPMEKLQKNPYFEKYKGKIHDLQQNSPEEFESRLEEFKKKGQPKEKKVEPKAKKQPVSAAASKMSQTSKLSQLKMTKEKTLNDIMKVDLLYDKSAQEIKQIWQQHFSKKKNTVFAVIPVKTYEVIHERSSTYPMFLYTLPRGDGYEFYVASFSGHECYFTPLIAYQAHQADSPVCLTLHHYSELGDEKGIVLMHGEYNSDILNAQDAQLLCQQVQIYYGPESTSYHLVETFNEQPDNFTDLISQLEKLTDNNEDGLNPVMGSVAALVTSIMKSSIKYKVVAAVDINDVANEVYKYNFQDINLLQRSIESISLKELNKLSPTMLLMSPPCQPFTRVGLQKDIEDARTNSFIYLLETLPKLEKLPNYILIENVKGFECSQTRDKMVSTLRECGYNFQEFLLTPLQFGVPNSRMRYYMVAKKKPLTFVFDTCEGKKLQKNPYFEKYKGKIHDLQQNSPEEFESRLEEFKKKGQPKEKKVEPKAKKQPVSAAASKMSQTSKLSQLKMTEEKTLNDIMKVDLLYDKSAQEIKQIWQQHFSKKESTVFAVIPVKIYEVIHERSSTYPMFLYTLPRGDGYEFYVASFSGHECYFTPLIAYQAHQADAPVCLTLHHYSELGDEKGIVLMHGEYNRDILNAQDAQLLCQQVQIYYGPESTSYHLVETFNEQPDNFKHTDLISQLEKVLPLR

>Sman_198180.1

MRVLELYSGIGGMHIAFKESTVKHEVVAAVEINGVATDVYKYNFPNTLTLNRVIESFSPDYVCSLNANVWSLCPPCQPFTRLGKRMCEADNRSSSFFHVLDLISILKPAGIILENVKGFEHSEPWRRLIEVLNSCDYEYRQFLLSPLQFGIPNCRLRFYLLARLRSSSWNSNFKMGKSESIDLRPPIDAPMLPGCQCTSCSGVISHIEHTDDNFTEYIQFCRPISEFLLVPSDSSKELYFLDEKCLQRYFRVLDIVRSCDKKTRCFTKGYSKRLEGTGSVFQTSMENEVSFFY

>Smed_15036408

MKVIEFFSGIGGIKCALNELNIQTDVVAAFDINDLANSVYRYNFTSPCYNKVIESISAEYLISLNADMWTMSPPCQPYTR

NGNMMDLDDPRTVAMKHTLYLISQVRPHYIFFENVKGFESSNGQKMLVSILSESAYSFQEFLLSPLQFGVPNSRLRYYLI

AKLEGKGSLMQDLEAISYKPYFDRKLYQCNCPVCSGRSRSLENDHVNHFERNLEFCDRISAYLEADNLAEPHEGHKLIDE

KVLEKGFSKLDIVTESSNKTCCFIKCYAKKIEGSGSYYQMTSCDEAHQLKQLLLNGDISSLDYAKRLKLRYFSPREIANF

MCFPQSFRFPETVTRAQRYRLLGNSVNVKVVAHVLHWLISA*

>Aaeg_XP_001657555.2

MSATDVTEDPHLPEYQVLELFSGIGGMHFAIERSGKRYKVVSAIDINPVANAIYNHNFGANKASNSNILSLNPDRIQKLGVNVILMSPPCQPFSRNGNFKDVDDRRADPFVHLCDLLDKIPTVQFILLENVKGFERSQACELYKTRLSAAGFRFKEYILSPHDFGVPNTRHRYYCVAKRTEFRNPSDEIVSKPTLQHVGTAKRICDLVEPESEKLNRYLLKDDLLRKRLAIMDICTPDSTNSMCFTKAYTHYAEGTGSVYSPLMRSEFDAIYKQIETTDDDDEKLKLLRSLRVRYFTPAEVAKLMCFPDDFEFPKQTTDKQCYRVLGNSINVLVVSSLFDEMDW

>Dmel_NP_001036355.1

MVFRVLELFSGIGGMHYAFNYAQLDGQIVAALDVNTVANAVYAHNYGSNLVKTRNIQSLSVKEVTKLQANMLLMSPPCQPHTRQGLQRDTEDKRSDALTHLCGLIPECQELEYILMENVKGFESSQARNQFIESLERSGFHWREFILTPTQFNVPNTRYRYYCIARKGADFPFAGGKIWEEMPGAIAQNQGLSQIAEIVEENVSPDFLVPDDVLTKRVLVMDIIHPAQSRSMCFTKGYTHYTEGTGSAYTPLSEDESHRIFELVKEIDTSNQDASKSEKILQQRLDLLHQVRLRYFTPREVARLMSFPENFEFPPETTNRQKYRLLGNSINVKVVGELIKLLTIK

>Dple_DPOGS212925

MVHKILELYSGIGGMHCAWNASELNGEVVTAVDINTVANEVYKHNFPNTPLITKNIQSLSYEDIKKMNVNTILMSPPCQPFTRNGKFLDENDPRTNSFTYLIDLFDELDNIEYILMENVKGFECSTVRNLFINKLKKCNFVYQEFLLCPTSVGVPNSRLRYYCIARKNTLDWSFIRTDEIITKLTKDYGEPHTLEAILETNVPEKYLLTNNFLKRAYLLDICYKHSKRSCCFTKSYTHYVEGTGSVYTDSTPDEVENCIKDAKQYEVGKEEYVDRFQQLKLRFFTPKEVLALMMFPKSYKFPERTTTKQCYRLLGNSVNVKVISELLKILFE

>Tcas_XP_008196999.1

MEILELYSGIGGMHWALKVSGVEGTIKAAVDINPTANSVYKHNFPHINLLNRNVQSLTPQFINKLGVNTILMSPPCQPFTRNGLQEDINDERTKSFIHVLAILPDLKVTRILIENVKGFERSKMRDLLIETLEKCGFNYQEFILTPTQIGIPNTRHRYYCLAKKPPNVFNFKTGVLKTEFPNQQNAPHCFEISKVLEQNELTPYYLTDKVLTNYLETTDIRYSTSRNTCCFTKAYGRYVKGTGSVYSDLPEITPEIFNQLSDHEPGSSAYLKLAHGLKMRFFTPREVGRLMSFPEDFTFPENTSDKQKYMLLGNSINVRVVAELIKLLQ

>Nvit_XP_001602026.3

MRVLELYSGIGGMHYALQESSVTGKIITSIDINTTANKVYRHNFPETANISRNIESVTVEEIAKLHIDCILMSPPCQPFTRIGLKKDSDDNRCLSLLHILQIIPHIESLDYILLENVKGFESSQARNEVILCLEKSGFNYKELILSPCQFGIPNSRHRYYLIAKRKGLKFIFDDASLITSIPEKVLELLPKNRYTSVPLEDGTHSSIKSKGKCFKLKYILESNVVENFLIPGKILLKRGSLLDIRTPESSGSCCFTKAYSHYVEGTGSVFSPSPDFEIKKKFEEINKSSETPEDKMQALLGLKLRYFTPKEVSRLMCFPENFEFPNDLSNKQKYRLLGNSINVHVVSQLIYLLYFENSVSLT

>Apis_XP_001949338.2

MRVIEFFSGIGGMHFALKECNLENFEVVLAVDINTVANAVYRHFFPSTNLRDLNILSLSPEQFDAYHPDILLMSPPCQPFTRNGLVKDINDERTKPLLHIIENIIPKSESLKYILVENVKGFESSLARDKLVNALSQSGFTFKEFLLSPVHFGICNSRLRYYLLAKKKPLDFAISLPNDIITENYWDDKLCSRVQQVSDVLSESDTELEEYLINDKQLLKGGKALDIVTKHSKRSCCFTRSYSSYLCGTGSVYSSLCEENIKEIISNNDDNLEVLKSLKLRFFTPAEVAKFMCFPVSDFPVSKKKAYQLLGNSINVYVVSRLLCLLLP

>Dpul_192688

MEKKQMRILELYSGIGGMHYAAELANVGAEVVFSVDINTSANAVYRHNFKQTNQQARNIESLSAKEINKLRPDIIMMSPPCQPFTRVGLKLDVEDSRCSSFLHLLDILPHLETVSFILMENVVGFETSEMRNAFTKALKNCDFHFREFILSPESIKIPNSRSRYYLVAKKCTDFSFGSENDIMTSFPNSRLCDIEMPVQEKTLDPYLVKDMSDEELARYLLTDKTLFKYWRILDVRQTSDTSSCCFTKAYTHYAEGTGSVLQHDPNEPFHQKFAEFKEDEDIAHLKPLKLRYFTPREVGNLMGFPAEFTFPENTSLKTRYRLLGNSLNVLVVSNLLRILLG

>Smar_SMAR004394

MADIRIHGKMNENTQILRVLELYSGIGGFHYALKDSDLKVEIVAAIDINTNANQVYMHNFPSTPIFQRNIQAINSKHFECFMPDLVWMSPPCQPFTRNGLKKDKMDHRTDSFLNILENLKLIRKKPKFILLENVKGFETSETHDLLTQTLNDCGYELNEYLLCPRQFGIPNSRLRYYMIAKLSEESVIPTRTSVINHTLNHSGEQIRHRNISEFLEDEFSDEFLIPDRILGKYAQAMDIVTSANDHSCCFTKSYGHYATGTGSVLRTNEMIDMEAVFLHEYQHENEKLNALKALQLRYFTPREVANLMGFPKTFGK

>Ptep_aug3.g2887.t1+aug3.g21986.t1

MDVSKCSQHITSCDKQNHCSNYCYRVLELYSGIGGMRYALEASQLLFDVVAAVDMNPVVNEIYRHNFGIKGHYQRNLKSFTVKEIDDMKIDIITMSPPCQPFTRVGLKKDIEDARSDSFLHIMKILVQLQRKPSYIILENVKGFEDSEARNVLIKTLEQCNYRYQEFLLSPIGFGVPNSRLRYYMTAKLESQPITTTDVKIFNIIPHSNYNWKSHCLKCIHSTYNMCSLKHHLEENSPDYFKPYLVPDHVLLKHWTVLDIVDETSNTCCCFTKAYSHYAQGTGSIFVPITDSLKVTEIFKQVACVDDNNSKLELLRNLGLRYFTPKEISNIMCFPDSFNFPSSITLRQKYKSLGNSLNVNYDNENEASEKGYTFEFTNIKGKCYVVFSENLTESVDDYFKGGPHRFYFNEAYDSSTGTFDEPPSIAQRIGRVGKGKGNIKGKKTTSNEKVESKPEEYPVINEKLKTLDVFSGCGGLSEGFHEAGVSDTRWAIEKEEPAAQAFRLNFPECSVFTEDCNLLLRLVMDGQETTYKGQNLPQKGEVELLCGGPPCQGFSGMNRFNSRQYSLFKNSLIVSYLSYCDYYRPRFFLLENVRNFVSFKRSMILKLALSTLVRMGYQCTFAVLQAGQYGVPQTRRRAIILAAAPGEKLPSFPEPQHVFSPRATQLTVVVDDKKYRPNIKWTSSAPYRTVTVGDSMSDLPSIKNGAKAEEISYSGDPVTHFQKK

>Lpol_XP_013779188.1

MVIRVLELFSGIGGMHCSLNATGISFKIEAAVDINTTANQVYMHNFPDTTLLQRNILSLSSKEIDNLKPDLITMSPPCQPFTRQGLKQDCEDVRTQPLLHILQVISDLEYKPYYIFLENVKGFETSRAKEKLVSTLQSCGYKFKEFLLTPTQFGIPNSRLRYYLLAKFRFGTSCVNMTQELTTSLRCEIKKEDMLFFKNSGLDSEIDSSNTAEGVVCRGGNTTDDEKTPLSFYLENKPDLYFQDYLLPDKVLLKFPMILDIVDRRSLQSCCFTKGYGHYVQGTGSVIHAVSHDKFIEVYKTVSLLNKEHPQTVELLKQLKLRYFTPREVANLLHFPSMFSFPSHLKKKQLYQILGNSINVYVVCELLKLLLQ

>Hduj_BV898_04582.p01

MASRDVRQVRRVLELYAGIGGMHFALDEYFKSADEAGEEISIMAVDINPSAKLVYDANFPSAPLRQIDIRALKDAHFDGVWLMTLSPPCQPFSRNGNVLDVEDARCESFLFVLAMLARLTNPPENIIMENVKGFETSEACRQMRDVLRKQGYRFKEYLLSPIHFGIPNSRLRYYLTAWRDPADKSELPKELPPDELSEDSVKTVIDAGTNYSELDLRQFLHHSVEIEHSTPPSLLIPLDILAKYLMVLDIKFPTSTTTNCFTSGYGRYFKGTGSMFCPLPEHVFADLVKEYLAHPDDRSTLAAMKLRYFSPVEVAFLMGFPNCFVIPSVVAPRLAWRLLGNSLNVHVVSLLLKQLLRPTSTITTESVAHFRNKL

>Bmal_CDP98269.1

MIDKQQNASFFCARSGEPLKCLEFFAGIGGFHYALKEIGLHLVIRALDINTVTNAIYKYNFPNTEIQQCNIEKLTTDYYDDYNADIWTLSPPCQPFTRKGLRKDVADHRCNALKKICNALKSMKKPPRYIIVENVCGFEASEAHHLLTDTLINLCYNFEEYIISPTKIGIPNSRPRYYLLAKLANNCIAIPTTSKIIDNWPKDDMAVLRSKVIGEYLCNEANEDNSLVILPEIVQRFGNVMSFVTPYNIHSSCFTKSYYRYVTGTGPILLQFSNNIQDQCISIEQLRDFVLYDNCKNLKYCQIRYFSWREIANLLGFPKTFAKPYEISSKQMYHALGNSITVSGVALLIQHLLKI

>Skow_XP_006813977.1

MGLRVLELYSGIGGMHYAVKESGVSADVVAAVDINTIANEVYQFNFPDCNLMQRSIEGITLKEFMKINADVLLMSPPCQPFTRVGLQADSNDPRTRSFFHLLKTLQSLSHPPNFILVENVKGFETSDTRNNLIETLNVCKYGYQEFLLSPNQFGIPNSRLRYFLLAKRHPYKFNFKLTDQILEELPQKQNLTEMSQDCTQETRPSLVRKSAEKSDGNKFSDCAMDGLSQLLEHCSCISDKVTESCDDDGASSSSSHLLVANSNIDHNDQSIRCVREFLEGDVQDITDYLIPDKILFRFGKVMDIVKPDCRRSCCFTKAYHHYVEGTGSVYQMNTTADTKKAFEKFLKSPSDDDDERISLLKNLQLRYFTPREVANLMCFPPEFSFPESISLKQRYRLLGNSLNVHVVAVLLQKYLLDDCQQKKS

>Spur_XP_011668392.1

MFVMSPPCQPFTRVGLKGDKNDARTNAFFNIMRNLAEMAKKPTYLLVENVKGFDTSETRNFLVETLQKCNYVFQEFLLSPMNIGIPNQRVRYFMLAKQRPLQFKEEHGTQLVQHRVNMPLMPNEIKETSRLVCDNSMVSSQEQTNMELTESRERTGQDESSMVSLNEQSIERKTDEQHLNEKERTDNVKSGSCEDMVSSSEQNEQETGEDDERRKIGDYLQADLSEESMAEYLIPDRILLKYVNVMDIVTVEDTKTRCFTKAYAYYVEGTGSVLRTDLSADMSSAFSSGSCSDDERLERLKALRLRYFSPREVANLHHFPQDFGFPECSTKKQKYRLLGNSLNVTLLAQLISYMVA

>Bflo_XP_002592995.1

MHSENDQTDPLRVVEFYSGVGGMHYAVLESKVPATVVAALDINTTANAVYRHNFPHVNLLQRDITGIKLPEFQSWNADVFMMSPPCQPFTRVGLKGDTADPRTKSFLYILDILRRMAEPPNHILLENVKGFETSETRGNLVTTLDECGYCYQEFLLSPNQFGIPNSRLRYFLLAKRKPLTFVFEHQTEIMKEMPVNSLIGQAPANQDASSSEVPNQSTSQNKQEIPCPYSIFDPEDRDTTNCREISEFLEPHSKEEEESLLLQDKLLDRYWKVLDIVTPSSRRSCCFTKAYGHYVEGTGSVLFSQTDIDAKDIFTKVSKSEDQSERLDLLHQLKMRFFSPREVASLHCLPPEFTFPQATTTKQRYRVLGNSLNAHVVAELFKLLVK

>Cint_XP_002128135.1

MEPELKVLELYSGIGGMHYALLGANLKNCEVVCSVDISPAASLVYKHNFPGTKHWERSIEGFSAKDFDNMGFNTLMMSPPCQPFTRVGLQKDINDPRTRSFIYLMKVLPQLSNPPTYILMENVKGFENSKAHDMFLQVLEQLEYSTAQFLLTPKQFGVPNSRLRYYLLAKRKPLQFPDEVNTPNEVIETMPQALQKCLNSNAGKEQNEKHTIKTLENYLEEAESTLKEHALTEKTLLRYLQVMDIVTPRHQSSTCFTKSYGYYAEGTGSVLNMGGEIKMSNPLHNNLRYFTAREVANIMCFPKDKFHFPENFTRKQKYKLLGNSLNVYVVSCLLKLLITDTKR

>Cmil_XP_007896370.1

MEAAARGEPRPRPGPGPRVLELYSGIGGMHWALRESCIPAEVIAAIDINTVANEVYKHNFPSTHLYPKTIEGMTLDDFNRLDFDMILMSPPCQPFTRIGSQKDVLDPRTKSFLYILDILPRLSKTPKYILLENVKGFETSVARDKLIQTLESCGYNYQEFLLTPTCLGIPNSRLRYFLIAKLQPGAFCFQTVNWILEGFPHHERDDWKQSKLPSSDQTLSTGDHTEKLNNSECGHADNSKCQEMETLIYKLETSGHLERKLKQNNNLSIQMIQDFLEEKLQNPSQYLISPKVLMRYALVLDIVGLTCRRSVCFTKGYGHYAEGTGSVLQTASDVELSTVFQEFETLSENEKLAQLSKLKLRYFTPREIANLHGFPAEFNFPEKISVKQSYRLLGNSLNIHVVAKLIELLVQ

>Locu_XP_006634398.1

MENLKVLELYSGIGGMHYALKESSVPAEVVAAVDVSTTANEVYRHNFPSTPLWAKTIEGVTLSDFNKIGFDMVLMSPPCQPFTRIGLQGDVSDHRTKSFLYFLDILPRLNKLPKYILLENVKGFETSSARNVLVRTLEECKYTYQEFLVSPTCIGIPNARLRYFLIAKAPPNLFSFETTVEIMVDFPSSGSTNVSSYKECSASSEPGHKTGGEQAVVYKLETAKDLERKKSQDSDVSVRRLQDYLEEEPKDLTQYLLPPKTLLRYSMLLDIVKPTCRRSVCFTKGYGYYVEGTGSVLQTCLNTQLEDVFKSLHLLSDEEKLNQLLKLKLRYFTPREIANLLGFPAEFGFPEKITTKKQYRLLGNSLNIHVVSRLIHLMFA

>Drer_NP_001018153.1

MENTERLRVFELYSGIGGMHYALKESLVPAEVVAAVDVNTTANLIYKHNFPTTQLLPKTIEGMTLQDFDRLNFDMILMSPPCQPFTRIGLQGDVADPRTKSFLYILDLLPRLSKRPRFILLENVKGFESSAARDALLQTLRECDYSFQEFLISPTSLGIPNSRLRYFLIAKRAPETFSFPVSTEIIEGFPMSESTDGLSVPYDHPTSSASEREKTIMFKLETEEDLERKRKQNSQESVRRLLDFLQEEEEDMEPYLLPPKTLLRYALVMDIVQPSSRRSVCFTKGYGHYVEGTGSVLQSCMDVDLETVFKNLDQLSEEDKLKQLLRLKLRYFTPREISRLMGFPDHFTFPKHISFKQQYRVLGNSLNVHVVSHLIRLMLSK

>Lcha_XP_005999072.1

MARLRALELYSGIGGMHYALRETCIPAEVVAAIDVNTTANDVYKYNFRDTPLWPKTIEGITLEEFDTLAFDMILMSPPCQPFTRIGLQGDVLDPRTKSFLHILDFLPRLLKLPKYILIENVKGFETSSARDVLIKTLERCAYTYQEFLLSPTCLGIPNSRLRYFLIAKLQPEPFCFQTTNQILEKFPNPHCLDTDGSKPIADFQEQPSLTASEAKPKGPSNLTGRGGRQHHGKEKEAVIFKLETAGEVVKKQEQDNDLCVQMLKDFLEEDVQDLSQYLLPPKSLLRYSLVMDIVKPTCRRSTCFTKGYGHYIEGTGSVLQTALEVEVETAFKSFESLSEEEKLRQLSTLKLRYFTPREIANLQGFPSEFKFLEKITRKQCYRLLGNSLNVHVVAKLITLMCG

>Xtro_NP_001004959.2

MARPLRVLELYSGIGGMHCGLTESGVAAEVVAAVDVNTIANEVYKYNFPYTPLWPKSIEGLSLKELDALSFDMILMSPPCQPFTRIGLQGDISDPRTKSFLYVLDVLPRLKKQPAYILLENVKGFETSEAREALIRTLEKGGYAYQEFLLSPTCLGIPNSRLRYFLIAKLQKEPFAFPTTSKILEEFPSQCPGSKRIIHCSESNPQARVEQKYSSCPLSGTDCDPEKTVLYKLETAVELQRKQTQDNDSSVRMLQDFLEGNVEEMSQYFLPPKSLLRYALILDIVKPTCRRSTCFTKGYGHYVEGTGSVLQTATDVEIDTVYKSLDLLTEEEKLAKLSSLKMRYFTPREISNLHGFPATFGFPEEVTKKQRYRLLGNSLNVHIVSCLISTLLQSP

>Acar_XP_003222002.1

MEPLRVLELYSGIGGMHYALQASNILAEIVAAVDVNTVANEVYTHNFCTTPLWPKTIEGISLKEFNKXILMSPPCQPFTRIGQQRDVSDPRTRSFLYILDILPRLAKLPKYLLLENVKGFETSSARDELIRTLEKCGFIYQEFLLSPTCLGIPNARLRYFLIARLQTEPFPFHVPGKILTDFPNQDQVDSERSRCSTGLMEAGSPLPTRETVSGSDDNDGHSSKRDFLFKLETEEELERKQQQDNDPSLQMLRNFLQGDGEDLSQYLLPPKSLLRYALLLDIVTPSCRRSTCFTKGYGHYIEGTGSVLQTEEDIQLESVFKSFESLSEEEKLMKLSLLKLRYFTPREIANLHGFPPEFGFPDKVTTKQQYRLLGNSLNIQVVAKLISLLVG

>Cpic_XP_005282499.1

MLGENSPVNLGGTGWENCYADCPAEAQICSSACCHSESCISAEVAAAVDVNTVANEVYKHNFPSTPLWAKTIEGITLAELNRLSFDMILMSPPCQPFTRIGLQGDVSDPRTNSFLYILDILPRLQRLPKYILLENVKGFETSSARDKLVETLKKCGFKYQEFLLSPTCLGIPNSRLRYFLIAKLQSELFSFQAPGQILENFPDQEPKASVKHRVAAGLEKESSFMPPKEEYIDPNSGSDCGGGQCSQKEAFVFKLETAKEMEKKRNQDSDLSIQMLKDFLEEDGEEMSQYFLPPKALLRYAFLLDIVTPSCRRSTCFTKGYGHYVEGTGSVLQTAEDVQLESVFKSIEMLSEEEKLMKLSTLKLRYFTPREIANLHGFSPEFGFPDKITMKQRYRLLGNSLNVHVVAKLISLLTR

>Ggal_BAD99025.1

MAALRVLELYSGIGGMHQALKESCICAEVVAAVDVNTLANEVYKHNFPSTPLWAKTIEGITLKEFDRLSFDMILMSPPCQPFTRIGLQGDVSDPRTKSFLYILDILPRLQKCPKYLLLENVKGFESSSARNELLRTLETCGFKYQEFLLSPTCLGIPNSRLRYFLIAKLHQEPFFFHVPGQILTRLPDQNLEELSKDKVVDKTGSXFPSGEKSLDSNTSPDCSSKKGPLPKGAFLFKLETAEQMVRKHRQDNDPSIQMLEDFLEEENEEMSQYFLAPKSLVRYAFLLDIVKPTCRRSTCFTKGYGHYVEGTGSVLQTAVDVELESVFKHIENLTEEEKLMKLSTLKLRYFTPREIANLHGFPPEFGFPDKVSIKQCYRLLGNSLNVRVVAKLISIL >Aaeg_XP_001657555.2

MSATDVTEDPHLPEYQVLELFSGIGGMHFAIERSGKRYKVVSAIDINPVANAIYNHNFGANKASNSNILSLNPDRIQKLGVNVILMSPPCQPFSRNGNFKDVDDRRADPFVHLCDLLDKIPTVQFILLENVKGFERSQACELYKTRLSAAGFRFKEYILSPHDFGVPNTRHRYYCVAKRTEFRNPSDEIVSKPTLQHVGTAKRICDLVEPESEKLNRYLLKDDLLRKRLAIMDICTPDSTNSMCFTKAYTHYAEGTGSVYSPLMRSEFDAIYKQIETTDDDDEKLKLLRSLRVRYFTPAEVAKLMCFPDDFEFPKQTTDKQCYRVLGNSINVLVVSSLFDEMDW

>Acal_XP_005095276.1

MPGSLATSLAAQRIILTDDSLQRLQSIEKDYNEGDITLKGYWKQKCSALESYFTDNVREQMRSLADDLENEEITEKGYYKKLEKVVTDFLKEVENDGSGMINGSASSRQNGHTGDLKSPGPEPSHTNGHTNGTSATNGTGSVNGTSATNGTGSVNGTSATNGTGSVNGTSATNGTGSVNGTSTVMNGSSAAKKLSKFAAGSSQSETMDEKDALKTESNGHTNGNKSMNGHASPKIKEVDTDVKQSSDSPASEEVMEVDSVEENGVDVNCSPPHKKSKQDKSNESFDSPKQEQTPRRSSRRVSKKSDEMQPSITAMFSPKAKVEEKASSKDKKENSAENGEEEEEEQQASKRIKLQDDEGKQEGIVPVLEPSAPKPLPQRCNECLKYMDDPELKMFPGDADDAVEEFIALTDPKLSLFTGEEDFDKIQEDRPQHKITNFSVYDKNTHLCAFDTGLIERNKYLFFSGVLKPIFEENPSLEGGIPCRNMGPINEWFVSGFDGGENALIGFSTAYAEYILMQPSEAYTPFIEQMKVKVHMSKIVIEFLTNNQDSTYEDLLNKIQTTVPPAGLTSFSEDSLLRHAQFVVDQVQSYDEAAADDEPLLIVTPCMRALIKLAGVTLGKRRQLRKDVGRVKAKKEKPKHTMATVTPLVRNIFDSLFQGQIDEESSKTKKQRCGICEVCQQPDCGKCAACRDMVKFGGTGRAKQACKERRCPNMAIKTADEDEEEDTEIDKVSEENAAVVKKHKAAKNAKAKVTWVGAPVKEEGKRKYFATAKVNNVEITAGDNVSITPADPTQPLYMARVQYLFEERGSMQAHVHWYFRGSETVLGEASDPLELFLVDECDDNVLEAVHAKVEVLFKSIPSDWSMLGGIDDPDVDNVVQEDDGKTFFYQKFYDPTLGRFEDLPMYEQCSESSKVDHCVSCYRLEQRRLLEEVAVGEEAESSSKTASYLYYNSVRRDGLEYHVGDCCYINPEGFDFLIKHPAKSGKQLRLKDKNMDEDMYPESYRKTEYVKGSNDGCPEPFRIGRIVQISAKKMSGSKLPDLAEIKIKVNKFYRVENTHKGQQAVYHSELNKLYWSEEEASVHFSQVCGKCTVLYQDNIAGDLDTFFLDSPDRFFFLESYNAESRSFEDPPAKARLMGSKGKGKGKGKGKGKSSKAEPSKEASEEDKVEIHKLRTLDVFAGCGGLSEGFHQAGLAESRWAIEKEEPAAQAFRLNNPGCTVFTDDCNLLLQRVMLGETTDELGQKLPQKGEVELLCGGPPCQGFSGMNRFNSREYSKFKNSLIASYLSYCDYYRPRFFLLENVRNFVSFKRSMVLKLALRCLVRMGYQCTFGVLQAGSYGVAQTRRRAIILAAAPGEKLPVYPEPQHTFAPRAMQLSVMVDDRKFNSNIKNMTSTPYRTITVRDAMSDLPEIKNGAKAEEISYKGDAQTHFQRLIRGHQHQPILRDHVCKEMSPLVAARMTHIPLVPGSDWRDLPNLEIRLSDGSKAKKLLYTHPDKRNGKGSNGQRRGVCACATGRVCDPLDRQFNTLIPWCLPHTGNRHNHWAGLYGRLEWDGFFSTTVTNPEPMGKQGRVLHPEQHRVVSVRECARSQGFPDTYRFFGNILDKHRQIGNAVPPPMGRAIGLEIKKCLVWKQKNGPEEEKTGSEVEKAKEEEKTEEKDLPESSQEKEEEEKKPTSNGSGGSEEVEDMESESKQESGSKGADSGTAGAGASSSSALA

>Acal_XP_012941472.1

MVSTMQPKCLRVLELYSGIGGMHYALKESGADFEVVMAVDINTSANSIYKHNFPDTDLHSFGIEKLTLKRLENMNIDAILMSPPCQPFTRVGKKRDCDDVRTKSFLHLLQLLRQLKRKPNYLLLENVKGFEVSLTHAQLMSCLDDCGYHYQQFLLTPLQFGIPNCRLRYYLIARLDQPFSFPMEKEIMTTLPRVGSPVGVSSAPVTSSESNSCSPTQELQGSSGVSVGVDATVSSQQSQVNDHDNGNNNSEQTHRVGDSGDDAPSSSQTVGDRVCQEVCGSNIDSGTSEVCSAKVSRLEVEKDSSDRSQGQGNNWDQTYHTATSSCDRSNNTKIDGAVETGPGDNISTRLCAQDCDSVSDIEALKQAGRNMRYCEEEDPLIGAHSRPLSQFMEQGGEEDFSQYLVPDRELHMFVVMDVVFPVLKKSTCFTKRYSHYIEGAGSVVQMTTNVQDVAAACEFKAKAVKMKNRSEWGTKELQILRNLKLRYFTPREVANLLCFPPEFDFPAGFSPLQLYRVLGNSLNVHVVAMLFTLMTRTV

>Acar_NP_001280056.1

MKKEKRVCWAMDSEETVVLVNGNCGDPSEDKANAALDVKGKRKKPDSSDAEQLSSKTEEKTDEIPGSPKKWQEVNMKRKKRVLSDTDSEDEVILINISDDESAEEKANANLDVKGKRKKPDADDAGQLSPTREEGQEIRTRGNAGWENCLRQKPPPRIVFQAGPASQDKKSKDDVESVESFKERHKIRYSRRISRNGPINFSEDELSESSRVGSNGIPNGNHDPGLLRGCKEYQDGKPFQMGELVWGKIRGFAWWPAIVVTWKSTSNRPAAPGMRWVKWFGDGMFSEVSADKLVGLVNFRDHFNSSTLQKMASYRQAILHVLEVASSRCGKTFPSNAGDSIEEKLKPMVEWALNGFKPTGGKGLKPPKGEEDVDQEDKSSVEEVVVVLEPSPPPKRVKTAVCNGNGKEQSEENQSRERMLSEVRNNKKSLEDSCLCCGKSNPATFHPLFEGGLCTTCQDRFLEYFYMYDEDGYQTYCSICCEGTELLLCDNASCFRCFCVDCLNTLVGRGAADAAREEEPWSCYMCQPQKQHGLLQRRHDWNTRLKEFFTNERGQEYKAPKIYPAIPAAQRRPIRVLSLFDGIATGYLVLRDLGIKVEKYVASEICEESIAVGTVRHEGNITYVHDVRNITKRNIDEWGPFDLVIGGSPCNDLSIVNPARKGLYEGTGRLFFEFYHLLNYSRPKPEEERPFFWLFENVVAMRVNDRRDISRFLECNPVMIDAIKVSAAHRARYFWGNLPGMNRPLVASKTDKLELQDCLEYSRTAKLKKVQTITTKSNSIRQGKAQMLPVRMNGKDDDLWCTELERIFGFPLHYTDVSNMGRGARQKLLGRSWSVPVIRHLFAPLKDYFACD

>Acar_XP_003222002.1

MEPLRVLELYSGIGGMHYALQASNILAEIVAAVDVNTVANEVYTHNFCTTPLWPKTIEGISLKEFNKILMSPPCQPFTRIGQQRDVSDPRTRSFLYILDILPRLAKLPKYLLLENVKGFETSSARDELIRTLEKCGFIYQEFLLSPTCLGIPNARLRYFLIARLQTEPFPFHVPGKILTDFPNQDQVDSERSRCSTGLMEAGSPLPTRETVSGSDDNDGHSSKRDFLFKLETEEELERKQQQDNDPSLQMLRNFLQGDGEDLSQYLLPPKSLLRYALLLDIVTPSCRRSTCFTKGYGHYIEGTGSVLQTEEDIQLESVFKSFESLSEEEKLMKLSLLKLRYFTPREIANLHGFPPEFGFPDKVTTKQQYRLLGNSLNIQVVAKLISLLVG

>Acar_XP_003226840.1

MEEEFSALEGQRRKRLEEQKVANGLDAEVTTGPSSLSVEALKEVRLKDLERNEDGLGERECVKEKLSLMHGFLQADVQNQLKDLETKFHKEELSEERYLAKVKALLHKELSFENGDNLELGQKTNGCAENCANGSDEEPERMGNKTEEDSGMEIEEAASSSSSTPLASPSVLKARKGRKSKSNSESRKTPSSSRVTRSSGKQPTIVAMFSKGSNKRKSEEVNGKLKQETNLENEEEEEEEQFGEKEPDVKRFKTETKEEGLERKEEATQIKTGTPVKTTPPKCAECRQYLDDPDLKFFQGDPDNALDEPEMLTDERLSIFDANEDGFESYDDLPQHKVTSFSVYDRKGHLCPFDTGLIERNIELYFSGVVKPIYDDNPCLDGGVRARKMGPINAWWITGFDGGEKALIGFTTAFADYILMEPSEEYAPIFALMQEKIYMSKIVVEFLQNNPDVSYEDLLNKIETTVPPAGLNFNRFTEDSLLRHAQFVVEQVESYDEAGDSDEPPVLITPCMRDLIKLAGVTLGKRRAARRQAIRHPTKIDKDKGPTKATTTKLVYLIFDTFFSEQIEKTEKEEDNENVSKRRRCGVCEVCQQPECGKCRACQDMIKFGGSGKTKQACFQRRCPNLAVKEADDDEEVDDNIPEVPSPKKMLQGRKKKQSKSRISWVGHPVKSDGKKDYYQKVCIDSETLQIGDCVSVSPDEPTKALYLARITAMWEDVNSSEPMFHVHWFCRGTDTVLGATSDPLELFLVDECEDMQLSYIHGKVNVLYKAPSENWSLEGGLDVEIKMVEDDGRTYFYQMWYDQEYARFETPPKIQPTEDNIHKFCMSCIRLDEVRQKEIPRVLEPQEESEGKMFYSMAMKNGVQYRVGDGVFLLPDAFSFSLRLASPIKRQKKEAVNEELYPEHYRKYSEYIKGSNQDAPEPYRIGRIKEIYCNIRSSGRPNEAEIKLRVYKLYRPENTHKSVKASYHADINLLYWSDEEASVEFKDVQGRCTVVYGEDLTENIQDYSASSSDRFYFLEAYNAKTKTFEDPPYHARSSGNKGKGKGKGKGKSKGKSSTASEHSEHEAAKMKPPKLRSLDVFSGCGGLSEGFHQAEVSETLWAIEMWEPAAQAFRLNNPGTTVFTEDCNVLLKLVMSGEKTNSLGQKLPQKGDVEMLCGGPPCQGFSGMNRFNSRTYSKFKNSLVVSFLSYCDYYRPRFFLLENVRNFVSFKRSMVLKLTLRCLVRMGYQCTFGVLQAGQYGVAQTRRRAIVLAAAPGEKLPMFPEPLHVFAPRACQLSVVVDDKKFVSNITRTYSGPFRTITVRDTMSDLPEIRNGASALEISYNGEPQSWFQRQIRGSQYQPILRDHICKDMSALVAARMRHIPLAPGSDWRDLPNIEVRLSDGITTRKLRYTHHEKKNGRSSTGALRGVCSCAEGKACDPADRQFNTLIPWCLPHTGNRHNHWAGLYGRLEWDGFFSTTVTNPEPMGKQGRVLHPEQHRVVSVRECARSQGFPDTYRLFGNVLDKHRQVGNAVPPPLAKAIGLEIKSCVSAKIKEGATDTIKREKMDTSD

>Acar_XP_008105653.1

MSSVFTAQEELDVIVVDSDSDLEEVDLPMETAVLLKNRSLSDVISVSSVEEMLPSSPLGPNTIVFLETENSDDVISVKSVEELNPPSLSRGNISYEVNSNRRSIEEICICCGELEIHTQHPLFHGGICAPCTETLLERFFLCDEDGSQADCTICCWGNSLMMCDDPKCHRCFCEECVETLVCPGHSEEIKDTNPWNCFLCVPNNRYGLLRKKTKWREYLKHFYDQESNSLQIYQPLSPWERKPIHVLSLFDNITKELNRFGFLEKNMGNGRLKYLDDVKNVTRTHIEEWGPFDFIFGSTPPVSMSYQDPPAWYFYQYMRILQYGRPPEGSQKPFFWLFLDNLVLDEEDRDTASRFFQVEAVLRYKQHGEIIQNAVHVWSNIPSVNSKYSAASFYMDLSQLAKEILRTRIFSQRPATLIKEFFVPLKDYFRAFS

>Acar_XP_016853309.1

MPSNGPVDTTSSSSVADREAADARKQGEEEADENRNKDEKPDPGIPARKAGRPGRKRKQPVAETSEAPKDTASAPKCPPASAHHSSSSSPMEQVPNGDVEAGSPEKATTAAATAEPKGRPRSESEPSALPERETGRTLENGRCCTPKDSPEAPASEGKEEKEENHYNSLKMEGSRGRLRGGLGCESNLRPRPMPRLTFQAGDPYYISKRKRDEWLARWKRETDNPPATQLDKAEKKAKVIAGMNAVEEALPRSEPQKEEEASPPASQQPTDPASPNVATTPEPLGPDAADKGMSKTADDEPEYEDGRGFGIGELVWGKLRGFSWWPGRIVSWWMTGRSRAAEGTRWVMWFGDGKFSVVCVEKLLPLSSFANAFHQATYNKQPMYRKAIYEVLQVASSRSGKIFPSCPENDETDTSKAVEIQNKQMIEWALGGFQPSGPKGLEPPEEERNPYKEVYTEMWVEPEAAAYAPPPPAKKPRKSTAEKPKVKEIIDERTRERLVYEVRQKCRNIEDICISCGSLNVTLEHPLFIGGMCQNCKNCFLECAYQYDDDGYQSYCTICCGGREVLMCGNNNCCRCFCVECVDLLVGPGAAQAAIKEDPWNCYMCGHKGIYGLLRRRDDWPSRLQMFFANNHDQEFDPPKVYPPVPAERRKPIRVLSLFDGIATGLLVLKDLGIQVDRYIASEVCEDSITVGMVRHQGKIMYVGDVRNVTQKHIQEWGPFDLVIGGSPCNDLSIVNPARKGLYEGTGRLFFEFYRLLHEARPKEGDERPFFWLFENVVAMGVSDKRDISRFLESNPVMIDAKEVSAAHRARYFWGNLPGMNRPLASTVNDKLELQECLEHGRIAKFSKVRTITTRSNSIKQGKDQHFPVFMNEKEDILWCTEMERVFGFPVHYTDVSNMSRLARQRLLGRSWSVPVIRHLFAPLKEYFACV

>Acas_ELR21928.1

MLYPWVQLLKWEGIAPFGPNLAADHAQSMRANTFVPVAHVLFVVESSRPERGWTRFVLRDPTGTVPAGTTDSDVAAKLVVGAAVKLTNVRLVPDSAFCGRLGIVIAWENLGRVVAPKSKPGKPHTNNISTFKETTPFSIYTSLEGVKKDEVADITYRQFRAEWTRRHPSPTAPPPPPPLPTHTTATVSSGDESFSSPSKEVLQQQPVAETVDALHHLVEEEEEDAGESDLSQPSDLPSSQPSEGDSSAGQRRTSAKRRYLKRVESDPTLPDSQPSPQSPRAFPRAEESETAKHDDVDGSDDDEEERKASTNAEAATTAKRTKAKAKKVTKAKAKAKSEAEDELVKRKRGRPKKEAIETEEERIKKHRKSEEHKKKFVELFIKHAKPKVVMTPVVPLDEQARQLLDRCEFQQSQRLSQHQSQRAKSQRIGDNENESEDEDEAEAEADSEEDLTQDINQLEVVLTQCKQLCESALRALKLLGIRVKAYFVVEKDPLAMDVVRANFPPEEHPYIRYLGCVEKITTADLEDIGAAPRLKLRSLRTLERKETGQQLTSCVFPIPRPVDLFIGGSPCTDLSVLGKKKGLDCPEGSSYHFYDFLRILQDLRRLCNSDSHNVFFLLENVASMSRKDKTKISDCMGVEPVRIDSKDITNVRRNRYYWHNIPRNINALPKATFQELLSQDGEALVLVGGCITTHNATMEYLKKSLEELLARPQDLKRYNTIKIKGQDKCRSLKATEMERILGFRTKDSNEREYTKEGRDINGRVYILTKAQRNKLLGQSFDTRVIAHLLTPLLKLCAGEEQLESRILQIRRQVTLSQMHFVQPRPRPAAAAVIIDKGKDRAIDDEDDDADEEEEEDDDFPLTQAG

>Adig_4714

MVQLKDDFPPAISERLSELEDEYNDGDITEKGYVRKKCKLMKPLLANFQQERIQEIEDDLKAGKFSEEQFISHLKELLTEIGHRSSNGCGAKPLPPVCNKDSNNVTLNAEPMETGMEINASDIPYTQASDCHEMEEANDSEGEPSSQSEPGLRQSSCNNSNEPTSSNGAERSQCLLDIKVSLTDLKKSTSPTNKCSQEGNKIKVNKKESKKQPGIKEMFAKNALKRKKTEDSSTDDSAGQAELSSENSEASNYSQDGKRQKTSGDDCDEEESSGDSGIERQSLRESTVGIRSEEKRAQPPAKCKECKQLLNSPDLRLFPGDSNDAVEEFVALTDPRLSLFSGEEEQCDSYSDTPQHKITNFSIYDKNTHLCPFDSGLIEKNVELFFSGYLKPIYDENPSPEGGVPTKNIGPINEWWVAGFDGGENALIGFTTAFAEYILMQASEDYMPFMNIMREKIAMSKIVVEFMQSNPEARYEDLLNKVETSVPPANCSTFTEDTLLRHAQFLVEQVESYDQAADDDELPLLISPCMRDLIKLAGVTLGKRRAARGVKVRTEKKQARPSKATTTPLVRQIFDIFFKDQIDGKEIGAIRRKRCGVCEVCQLPDCGKCKSCKDMVKFGGTGKKKQCCEERRCPNMAVKEADEDDDIGEDIEDAENVTLNKRITKKKSPTPKKTKSKSKVQWIGDPEVHGKQNYYTSVLIDKEEICVGDFVMFRPDENSHLALYIACVRYMWEEPNGDKMFHCRWFSRGSETILGETSDPREVFLLDKCDDNLLGCIKQKCTVTYNKPDADWFMKGGLDEPEGDVSMEENDGNTFFFQKWYDPDHGRFTDPPAEYLHVVNSEDNFRYCESCVRQSAEENLETLSLGEPLEVDSKPSSKSYYKSCSKGGNRYKSGDCVFLEPDAFSFNVKPKESKKGFKKDDMVDEEKYPEYYRKPLEYVKGSNYDVPESFKIGRIINIFTKSSPGKLSKDLDIMLTVGKFYRPENTHKGSSFAYQADLNLLYWSKEEATVPFDAVLGSCTVTCGEDLNCSIAEYTAKAINNFYFLEAYNSETKDFEEPPLEARNAALKGKGKGKGKGKARSACQTEEQNLRQDSKPSETVPKLRCLDVFAGCGGLSEGLHQAGTAESLWAVEKEEPAAHAFSLNNPGCTVFTDDCNLLLKLVMDGEKKNSRGQTLPQKGEVELLCGGPPCQGFSGMNRFSSRDYSQFKNSLVVSYLSYCDYYRPRFFILENVRNFVSFKRSMVLKLTLRCLIKMGYQCTFGVLQAGCYGVPQTRRRAIIMAAAPGEVLPLYPEPTHCFSPRAIQLTVMVDDKKFESNITRLSSAPFRTITVRDSMSDLPEIRNGASNAEISYSGDSISHFQRQVRGSQYQPVLRDHICKEMNPLVAVRMRYIPLAPGSDWRDLPNIEVQLPDGTKTKKLAYTHHDKKNGRSSEGQLRGVCSCAEDKPCDPADRQFNTLVPWCLPHTGNRHNHWAGLYGRLEWDGYFSTTITNPEPMGKQGRVLHPEQHRVVSVRECARSQGFPDTYRFYGSILDKHRQVGNAVPPPLAAAIGREIKKSLQATQQNA

>Adig_DNMT2_modelPK

MAEELHHVVEFYSGIGGMHYALKGCSFPCKVVAALEINTTSNLVYRQNFGSTVLLQKNIEGLTVRDIDNLSADVFVMSPPCQPFTRLGLQAASKDPRTKSFLHILDLLPKLSKPPDYILMENVKGFETSDTREHFLETLKSCGYSYQEFLLSPTQFGIPNSRLRYYLLAKRKPLQFCFAMQDETQITTTILMDNLKRSRKRRRSSDDARERQTVSEHKSGDHVVNKGVKSFWHDIIKKYMEEKGITSFHDGLTPTDTPTLYKICIEDRYWPLSDATAQLIDQMKVFSVKKDKPDEYFEDFLLSEKVLSKFALLLDIVTVHSERSCCFTKAYGHYAEGTGSVLKMADIDDTEIFCKYRDLSNEKQKSQLLSVLRLRYFTPREVANLHGFPTEFCFPASVSVKQKYRLLGNSLNVHVVSELMNCLLQSPSGDTKGLATEITEKVKRCHEDVLIVAGRKAALHPTS

>Adig_v1.01094

MEATPTSEAIQAPKKKPKFKRRLEFSASDYKSKKKIRVKSDVFISVTSVEDNHLATSSDDHLSTSDDENGATFVHGQLIWGRLKGYDWWPGLIVSHLEAQKAPPAPSNHWIKWFGDNKLSLLPFQCLRPFSKFKESLIPSKMRGIYKRAVFDSLEVAVKRSGKVFAKCPIKQTTQTKKKTATRRAKVGAEQIAGLHEDQEPLGERERQEMMVQWAMNGFKPNGPKGFAPPEDDMISDTSVSVTSFMDVISSDDSESTADHKSRFGKPVNVPNLAENIKALFEEVSKGKRDLECICLACGDLRVCAKHPLFKGGLCKECKTSFLGNIYLYDEDGSQMYCTICGDGKEVFMCDNDGCFRSYCNLCIGMLCGVNAVRKIAACETWVCFMCSSKTQGLISPRQDWSARLQEHFMNDKEQEFKPPHTFSAIPPEERRPLRVLSLFDGIASGLQALKELGIEIELYCASEIDENAVQVAKVQHGRKIHHIGDIQKISRDQIQDLGPFDFVFGGSPCNDLSIANPIRRGIYEGTGRLFFDFFRLLEYARPKSELERPFFWLFENVVGMRAEDKAVISRFLECNPVVVDAKEISPAHRARYFWGNLPGMNRPTIPLPGDKLCLQDCLEPNCGRQAKFTKIQTLTTNANSMLQTKKALLPVQYVASDGHEKEDILWITEMERLFGLPSHYTDVSNMGRSQRQRLLGKSWSVPVIRHLLSPLKDYYKCET

>Aipt_28556+28558

MVKLSTLPDGIADSLESLEKEFEDELLTKKGYIKKKYLLMKAYIPQYAIESIESLEKKSKNSSIEEESYLNQLDEILNEFQPSKTVQKGQGSSSSCSDHEMRDDSEQHVTNGSNGYAESLQNEDTVQEDKHGEDKNRNGNFEENGDTEEETPLNGSSCETEIKTTTRKKRKEQEKSGKKAKTSPHRQPGIAEIFSKVPCKRKGPGNGDDQESASSSGMSGADVDTAETQQEKKIKLDDNGDEKKGSAEPDSSESKRKKITNSKVQIPPRCNECRQLLNNSELRMFSGDGEDAVEEFIALVDPKLSLFTGEEEQFGSYDDKPQHKLTNFSVYDKNTHLCPFDTGLVEKNVELYVSGYVKPVYDENHVLEGGVPTKNMGPINEWWTAGFDGGEKVLIGFTTAFGEYILMQPSEEYKPFMEAMAEKALLSKIVIELLIENADASYEELLYKVQTAIPSDNCTEFTEESLLRHSQFIVEQVENFDEARDSDELPLLISPCMRDFIKLSGVTLGKKRGARKVKIKTEVKKRGPTKATTTPLVRHIFDIFFKNQIEGKGVSAPRRTRCGICEKCQQPDCGKCKYCRDMVKFGGTGRQKQSCLERRCPYRDIKEAEDNETLEEYDKDSELDNRKGANKVHHKIAQDQKIVKTKVKWIGEPVRETNTRRYYSSALINKEEVTVGDFVQVCPYDPHTPLYIACVSYMWENSKGKKMFHARWMTRASDSVLGESADPRELFLADDCDDNPLGSILRKCTVSIIFMKLFILFQSCFSMVRCSLLCRQIHRSILGFPVYNMGECRGGMVSAALLHILQLTFSWVVLVLCDVYILVDVTYRDPLKHGNKLTTEEEAANDDQHDENHFFYQKWYNSDMGRFTDPPIEYTVSVERAKTNYCEICTRNDAQEKFDTAFVKGEIEDSGSKTGYKTLAYKGITYNIGDYVYLEDGAFTFNVKQKPLVKPTVKSEGTVDEELYPEYYRKSSDYVKGSNQDVPAPYQIGRIVKILTKNVSGKLKSDEEVFLNVTKLYREDTHKGPSAGHQADSNLLYWTDEECSVSVSHLRGKCTVVYGEDIDETVETYTANGPDRFYFFEAYDSKRKEFHELSIEAREQLMSKKGKGKEGKGKGKGKGKMAATVKESDDKSKKEEVKKLKSLDVFAGCGGLSAGLHQAGVAESLWAIEKEVPAAQAFRLNNDCTVFTDDCNELLKLAMDGKETNSKGQKLPLRGEVELLCGGPPCQGFSGMNRFNTREYSLFKNSLVSYLSYCDFYRPRFFILENVRNFVSFKRSMVLKLTLRCLLQMGYQCTFGVLQAGSYGVPQTRRRAIIIAAAPGEVLPLYPEPTHCFHPRACQLTVKVDDKVFRSNTRTSSAPYRTVTVRDAMADLPGIDNGASACEISYDGEAISHFQRQIRDNQYQPMLRDHICKEMSALVAARMRHIPLAPGSDWRDLPNIEVRLPDGFSKKLCYTHHDKKNGKGSNGQLRGVCSCAEGQPCDPADRQFNTLVPWCLPHTGNRHNNWAGLYGRLEWDGYFSTTITNPEPMGKQVATNLCCGIPVD

>Aipt_O88508

MDRGCINSSAEDTLALPQKPQPEQVLEESLKNASPKKPSKVKRKLNFCDGYVKRKKKRIPQQKQEKSETSEDKIPSSTSKEPSDNLAVLEKGLKTPEKCGTDQKKEEEPLVTFTIGDLVWSKLKGFDWWPGRVVSYLETRLSPPNPGHHWIRWFGDDKFSQMLHMCVRPFHEFEVKFNPSKMRGVYKKAIYEALEMAAKRSKRSFTRADIKPKEPKKKQSKKKSQKPKPSKPLPTEKSKDGPLSEEEFREAIMSWALDGFLPDGPAGFAPSEGCDDDDDNGDNNDDNGDNDGDNNDDNDDDDNDDDNGDNNDDNGDNDGDNDDDDNDDIDDYNDDNNGDNDDNGDNNGDNNDDNDDNDDHTAVYVLTCWLVKGQMRAEDKKTISRFFKTNPVVDAKDVSPAHRARYFWGNLPGMNRPTKPVAGDKLSLQECLEPNCGRKARFTKVQTITTNANSLRQTKNNLLPVSTDSEGVEREDILWCTEMERLFGFPSHYTDVNNMGRQQRQRLLGNWSIPVIRHLVSPLKDYFKCRVETNKMTSSLMTSCW

>Aipt_Q54JH6

MAAICEKSSDSTFRVVEFYSGIGGMHFAAKGCGFKTEVVAALEINNTANVVYKHNFPDVKLLQRNIELTVKDLDGLAADIFLMSPPCQPFTRVGLQGDSSDPRTKSFLHLMDLILRMSRPPDYLLMENVKGFETSHTRSSFYHRISLEFPTQGLDITCLPRENLINFTLKHNRRNRRILAEFLEPEHDDYFQQFLLPERVLSKFALVLDIVTPFSTHSCCFTKAYGHYAEGTGSILKMADIDDVEYFDKYRSLDPGQSDSQRAEVLRDLKLRYFTPREVANIMYFPKSFGFPEGTTSKQKYRLLGNSLVHVVTELLRCLLHNS

>Apis_ACYPI073296

MEIIKSNKGGDKGYVYVVKDGATGTKRCSMKCTGELYTNLKIENPEVKTGHSHLKDVDSVKIEKALCAMKSDSHVKVEHMAGGVNGDPEPKGVKRKCEEPSITQEQHDSLSDNNGMHTDIPITNTKVRCKFCHKTNTEDVCIPSSAAPECIVLTNEVLANIYTDNDDVVQHNLTNYSIYDENGHMCSIDSGVMERNVTIYMTGYVKPIYDNSPSIEDGIAARELGPILEWWLSSYDGGTQALIGINTEYADYLLIEPHPKYKKYMTSVTEKIHLSREVIKNILSSEYDDPTYEDILNCIINSVNPATGNKFTEEDLISHAQFVLNQVLEYDLNDTYHLPLAETQCIETLTQLSGAAKPVKALSRRAGQRTQYVKTDINKREFSKATTTELVKDVFENMFAAQLDIDDKENEGNMNKLVKTMFDDVCNTKIKKEISDIRVNANQVLWKDKVLCEKMFVVVKNNVSTIPLIGCIEYFFKTKFKNMAHIQLFMHSYETVLGETADPQEIFSLKNCTNIDIKNVVRVLDVVQKIPSKNWFQLGNSKLQDLLEPVNRKNKNSFFYQLQYDDKFGRFEYPKPLEPNTVITEGYCSNCEDFDLEQKSKEPELYEFIEQDKNISYFYKFKLFDEEYKVGSFIYLQPEIFKTASRSNMIGEEPFELKKTSKNISNQEQKVIDETKYPEYYRKSLENTMRGSNETTPDPFEIAEILAIYFVDWDRKNIKLIVRRMYRAEQIPIEDYAKNSDMNMLFWSEEEFRVTYQNIRGKCYVSFISNIQDPLLWSSGGPDRFYFTKKYDFIFGSIVSEHELPSEAKLTGKEVYKIKPLRGLDIFAGCGGLSRGLEDSGLVISNWAIECDDKAAGAFKLNNPEATVFVEDCNHLLKLAMAGEKSNSKNQNIPQKGEVDFICGGPPCQGFSGMNRFNSGQYSLFKNSLIVSFLSYIDFYRPKYFVMENVRNFVSFKRSMVLKLTLRCITRMGYQCTFGILQAGNFGVPQTRRRLIIMAAAPGEKLPFYPEPINVFNRKSSSLSVQVGDEKFKTNCKYVNSAPLRTLTVYDAWSDLPGISNGAYQERIPYNSTPITHLQKLLRYPDNQYADSTLCDHICKEMSSLVQARMALIPVCEGSDWRDLPNITVQLPEGLKTNTLLYTHHDIKNGYGPNGALRGVCTCASGDKCDPQDRQNNTIIPWCLPHTGNRHNNWAGLYGRLAWSGFCSTTITNPEPMGKQGRVLHPEQHRVVSVRECARSQGFKDSFIFCGSIFDKHRQIGNAVPPPMGTAIGHAIIKAIYQ

>Apis_XP_001949338.2

MRVIEFFSGIGGMHFALKECNLENFEVVLAVDINTVANAVYRHFFPSTNLRDLNILSLSPEQFDAYHPDILLMSPPCQPFTRNGLVKDINDERTKPLLHIIENIIPKSESLKYILVENVKGFESSLARDKLVNALSQSGFTFKEFLLSPVHFGICNSRLRYYLLAKKKPLDFAISLPNDIITENYWDDKLCSRVQQVSDVLSESDTELEEYLINDKQLLKGGKALDIVTKHSKRSCCFTRSYSSYLCGTGSVYSSLCEENIKEIISNNDDNLEVLKSLKLRFFTPAEVAKFMCFPVSDFPVSKKKAYQLLGNSINVYVVSRLLCLLLP

>Apis_XP_016662566.1

MADNQSSDMEVDGNESTTVYSSKARTSELPWLRSSSRAKTSKLPRITSSSNPKPSKLPRLRVLSLFDGIGTGYYALLKLGFDIEVIYASEIDKDALMVTKYHFSDNIKQLGSVTEITTKMLDQIAPINLLFGGSPCSDLSGVNYRKKGLFDPKGTGILFYDYYRIWNYLSVKAARENTPFYWLYENVASMEIKNKDTISKFFECQPIVLDSLHFSPQRRKRYFWSSLPGITMLPHIQKTNYATNAPKLEDYLEKNLDRQANVEIVGTITSKRSCLQDSKSRNPVCQDGQYTGLFITEIEAIFGLPPHFTDVGDLSISSRQKLLGRAWSVQVIIDILDMLSGLFAKK

>Apis_XP_029348651.1

MFIDAEKQQGQVVWAKMGKKSRLWAAIILSAKEMDLVYFEDKPGKTCVWWLGDDTMSQLQNDLIFEFRSNLKKGIADLIYARKDNFEQAIYILSLQFNFEFPKHGLVKWALVNLPIIMKGKNSRKEEFIIPDQFLKKITMVNNRIKRNIEENKVKNVESSPHGEKMKIDVSQISKMENKYCIACRFPSDELKIKHPLFKGLICNDCFINGKDIILSIAKDKSNDGCCVCLTQKDTLVLCGFCIRAYCNECLEFYCGQKALEKILIDNAWACIACSSTTLKISNLVPRSTKEQLRNIYMLYPANAKKDCAKEQYKIKEIHVLNLKDIQNNVPKVLQTLGFPFKILETSYLNDNNQDNNKSNAKLVVQYYPNRENYDKDKFFDRNDCLKKFFLFLLFKNKMQALQKDDNLFWAFETSAAIKVQEQKIISRFLEIEPIIIGMDTDDVQQRSRFIWTNITILNKDIRKFTNQNIYLHEIPKSIGRKSKKDKRNIDMPWCYTSIYIILSSFINNDIMF

>Aque_XP_003386062.1

MAEGPANCSIPLYRKRKAPDHLASPYTAAKHLELSPSPAPDSTLSPNQPVENYVDPIEELKSLEEDMSNFRRFSGRSKKQTNFFCPIAPASFHRRRSNSATNAAVRALKFPSSTSPVDSGGPAGIAASEEKKESIDNGEGEDKVVAAMEIATGEEVEINKPQKPQQQTVPQEIKKLPIGSLIWGKLPGYEWWPGCIISFDKKKLIEVVKDEDDDDEDTEEDEEEKEEDGGESVWVKWYGDNQLSQISFKKIFPFGSNFLEFFSPNKLRGLYKKAVVNSLKEAARRCSKDIGCTLDERKPSWIFHSSQFQVFLDERKALNNLTQEEAIEATPEKIEQGEKSPVYSPDSSFPNLQQEESSIFTQIESDQESDSSPANGVKEKGGGRGAKKTKTGQFLLSLMEKDLLQWALTGFLPTGPDGFRPRPEHLIDIKMFPFGESPAPGNQFIIKRNELPMERGRAVIDHDYDKKPENKLVLLQQRNKAQFELIKLGQLAIENICIACGSVNISAQHPLFYGGLCKHCKETFMECAYMFDEDGSQMYCTICSGGSQVFMCDTPNCSKVYCNPCIEKLCGPEERMKVQDATEWQCYMCSGEMNGLLRKREDWDKRLHDLFVTDIETEFEPPKFFPPIPAEMRKPMRVLGLFDGIGTGLLVLKELGINVEVYIASEIDPDAIKVSRIKHPEIIHVGAIEQVTEKEVRSWGPFDLVFGGSPCNDLSIVNPARKGIYDGTGKLFFEFFRILSYAKPQPQDERPFFWLYENVVSMRAPDKKIISRFLQCNPVVVDARDISAAHRARFFWGNLPGMNRPAVPLPGDRLTLQDCLEPNCFRHAQFTKLRTITTKMNSIKQTKRAIFPVRVFSGGMFDITGEGEEEEGDVLWCTEMERLFGFPDHYTDVANLGRSGRQKLLGKAWSVPVIRHLLSPLKDYFKSDTTGVPSSLKQTHIPITPVSITTAPPAPVLPVIAPPPKYLSPEPETVIVIMKTDEMD

>Aque_XP_003389338.2

MQSKTDPEEPLPSKSLGVVEFYSGIGGWHYAIRETGLNLKILAAVDINTTANQVYKHNFPNTKVLQRNILGLSAQELDSFLANLFTLSPPCQPFTRQGKNEDDIDYRTDSFFHLMGILSAMQKPPQYIMMENVKGFELSRTRGHFVHVLKELGYTFQEYLISPKQFGIPNSRLRYYLLARLSPRNFKAAPLQDTVEHLKYYIPTSFHDIHLKCDISSYLDDLSEIDVQQFLVPDRILEKYAQGLDIVSAKSHSSCCFTRGYYHYAVGTGSVLHHDCSVDLALAYQCYAEQKNNCDGIKSLKELKLRYFTPQEVAKLMSFPASHTFPTSVSNKQCYKLLGNSVNVFVVATLLCYLCSQ

>Aque_XP_019849850.1

MLGGEEEEELLFQTSSKKKRVPRIIDDDDEMEVSRVTTAKRSNNKRKSSGTGASSNLSGGNSTNKSSKIQSSSTSGRQLGIGSFFNKDPQKRSKVEEEKEEVSSAIAPTGEEEREEEEGKEKEAKKAKTSETGSSSNGTSAATNGNNKSHDLRPHDDIPANKTSEAPQSSKPPCTYCKRSSDDPRLKIFIGDPPNANDEFITLADPSLSVLSADQESALDDVPQHKITGFSVYDKNHHLCHFDTGLVEKNVELFFSGWVKPIYDENPDPSDGIPTRQLGPINSWWIAGFDGGEKALVGFSTAYAEYILMDASDDYAEIMASVQEKIYLSKILIEYLEEFPAATYEDLLNKIQTSVPPQSIGCTSFTEDSLLRHAQFIVEQVESYDQYCDEDEDLLLVSPCMRALIKLAGVTLGKRLSSRRPHQPKVKKTKQTKATTTPLVRDIFETFFKNQIDNKSSSAPRRRRCGVCETCQQPDCGKCNACADMIKFGGTGRSKQACVNRRCPYMAVQTAEEEEDNDAADPDLKNVKDLKSPSKKIKKIKTKVEWIGEPDFVEGGKSYYTEVLINNKEKVCLYDVVSVCPEVPDDPLYLTRIMSMYEDSNGKKMFHGWWFHRSTDTVLGETGDPRELFLIDDCEDNPLGAIMDKVEVEYKPPVSNWFMCGGEEVSDDEKEIEEDGKTFFVQKYYDQSLARFEDIPSEYIRYLISDDAHPPTDGFIPQCASCERRNHMKTVHSAVPINKIESEKPKITNYSSFQLNEESYSIGDCVYLSPHTYSFPNVKKTSGTAANKKQKKEEEEFDESEYPEKYRKVSDYVKGSNIDSPSPFQIGQVLEIFSKSLGGKLIDNNKIVHIKLRMYYRPQDTHKGDEATAQYDLNLLYWSDLVTTVVAGDVVCGKCFVKFKEDITEDIDSYFSNKPNHFYFVEAYCADTKEFEDPPVHAMNKGKGKGVSKGKGKGKGPAKGSKESIATTSTDDDKKDEITPDSSFKKLRMLDVFAGCGGLSEGFHQAGVADSCWAVEIDEPAAQAFRLNNSQTTVFTDDCNILLSLVMEGAKTNSRGQLLPQKGDVELLCGGPPCQGFSGMNRFNSREYSQFKNSLVISYLSFCEYYRPRFFLLENVRNFVSFKKSMVLKLTLRCLVKMGYQCTFGVLQAGQYGVPQTRRRAIILAAAPGEKLPHFPNPTHVFSPRACQLTVVVNDIKYEGSIRMDSAPYRTITVRDSMSDLPHIKNGSAVRSMNYNGEPHCHYQRLMRGNQHQPVLYDHICKEMNPLVAARMRFIPIGPGSDWRDLPNKCIRLSDGTTAPKLQYTHHDKKNGRAKNKSLRGVCPCATGQPCDSSYRQYGTLIPWCLPHTGNRHNHWAGLYGRLEWDGFFSTTVTNPEPMGKQGRVLHPEQHRVVSVRECARSQGFPDTFRFFGTILDKHRQVGNAVPPPLAKAIGLEIKRSVEKKDK

>Aque_XP_019859084.1

MLSSPRPCHILPSSQSKTKDNQPVIQLYSPPAAPQSSQPQCTGPYCNRSSNDLTLKTFIGDAPNANDEFIMFADPSLSVFSAESALDDVPQHRITGFSVYDKNHHLYHFDTGLVEKNVELFFSGWVEPIYDENPDPSDGIPMRQLGPINSWWIAGFDGGEKALVGFSTAYAEYILMDASDDYADIMASVQEKIYLSKILIEYLEEFPAVTYEDLLNKIQTSVPPQSIGCTSFTEDSLLRHAQFIVEQVESYDQYCDEDEDFLLVSPCMRALIKLAGVTLGKRLSSRCPHQPKVKKTKQTKAIFETFFKNQIDNKSSSAPHRRRCGVCETCQQPDCGKCNSCADMVKFGGTGRSKQACVNRRCPYMAVQMAEEEEDNDSADPDLKNVKDLKSPSKKIKKIKTKVEWIGKPIFVKGDKSYYTEVLINNKEKVCLYDVVSVCPEVLDDPLYLTRIMSMYEDSNGKKMFHGWWFHSTGTILGETGDPLELFLIDDCEDNPLGAIMNKVEVEEKPPVSNWFMCGGEEVPDGEKEIEEDGKTFFVQKYYDQSLARFEDILSEYIRYLIPDDADPPTDGFIPRCASCERRNHMKTVHSAVPINKRNKRVREIKDYKLQVIPVNFDESEYPEKYRKVSDYVKGSNIDSPSPFQIGQVLDIFSKSSGGLLIDNNEIVHIKLRMYYRPQDTHKEDEATAQYDLNLLYWSDLVTTVVAGDVVCGKCFVKFKEDITEDIDSYFSNKPNHFYFVETYCADTKEFEDPPIHARNKGKGKGISKGKGKGKGPAKGSKESITTTSTDDDKKDDITPDSSFKKLRMLDVFAGCGGLSEGFHQAGVADSCWAVEIDEPAAQAFRLNYSQTTVFTDDCNILLSLVMEGAKTNSRGQSLPQKGDVELLCGGPPCQGFSGMNIFNSREYSQFKNSLVISYLSFCEYYRPQFFLLESVRNFVSFKKSMVLKLTLRCLVKMGYQCTFGVLQAGQYGVPQTRRRAIILAAAPGEKLPHFPNPTHVFSPRACQLTVVANDIKYEGSIRMDSAPYRTITVRDSMSDLPHIKNGSAVRSMNYNGEPHCHYQRLVRGKSHSGNQHQPVLYGHICKEMSPLVAARMRYLPIGPGSDWRDLPNKCIRLSDGTTAPKLQYTHHDKKNGRHKNKSLRGVCSCATGQPCDSFYRQYGTLIMPWCLPHTGNRHNHWAGLYGRLEWDGFFSTTVTNPEPMGKQGRVLHPEQHRVVSVKECARSQGFPDTFHFFGTILDKHRQVGNAVPPPLAEAIGLEIKRSVEKKDK

>Avag_GSADVT00036986001

MVLRVLELFSGMGGMHSALDLIEIEYEIIAAIDINPMANLVYSSNFPQVPIINRSIETISIKQWEKWHADLWTMSPPCQPFTRQESKPKYILLENVFGFQDSDAHRLLLETLQLNNYSYEQYLLSPIQFGIPNSRLRYYLIAKQQENPIDTHSSIIHTQISNFERLLTKLHEQFPSKDISSHDETSRHRNDQITDQCSHSSEILIDPNQSMIINDFILSDENAEFELDDKQLKRGLNVCDIITCYAKRSFCFTKSYGKYFEGTGSLFRTQNDHIRYFTPKEIANLHCFPSTYNFPESITTRQAYSLLGNSLNVFVIGVLFENLFER

>Bflo_204680

PVKCKECRQLLDDPDLRMFPGDPQEAVDEVEMLTDERLSLLEEDQEYGTYDDRPQHKITNFSVYDKNTHLCAFDTGLIEKNVELYFSGAVKPIYDENPSADGGVLAKNVGPINEWWTAGFDGGENALIGFTTAFAEYIVMQPSEAYAPFMDTMWEKIHMSKLVIEFLVNNQEAAYEDLLNKIQTTVPPQSLRVTSFSEESLLRHAQFVVEQVENFDLAGDADEAPLLTTPCMRALIKLAGVTLGKRRAGRSGIKQPRKVKAKPMRQTKATTTNLVCSIFDTFFKDQIQDDKAGGPRRRRCGVCEVCQQADCGTCVACKDMTKFGGTGKSKQACVNRRCPNMAVQDADDDDDIEDDLDKEFSSTVGHKRTHFSPRKKVNSKVKISWEGKPIKEEGKKTFYSAVMLEEEKIETGDCVTVTSEDPTKPLFIARVMYMWEDSGGDMMCHVGWFCRGSDTVLGETSDPLEVFLVDECEDILIQFVKFKCKVILKTFDKDWFSHGGVEDPDSDYPIKEDDGRTFFCQKWYDPDLARFEDPPVLEEGSGHKFCPLCLRLDHSRKSEIPIVGEQLDDTMGDDNKVYYSLCQKGGLEYKIGDDVFLLPDSFSFSVKVKNSTKKASKKDNYDEDLYPEYYRKRGDYVKGSNEECPEPFRIGRIISIYCKRTVSGIINTHDVRLRVTKFYRPENTHKGKAGSHHTDLNMLYWSDEEAVVDFMSVQGKCQVVYGEDLDVSVEDWSSKGLHRFYFNEAYNTESKQFEEPPNKARSGGKKAKGKGKGKGKGKGKSSEAESKEQQPPSPAFTKLRMLDVFAGCGGLSEGFHQAGIADSKWAVEVMEPAAQAYRLNNPDCTVFTDDCNILLKLVMEGATTNSTGQRLPQKGDVELLCGGPPCQGFSGMNRFNSRQYSQFKNSLVVSFLSYCDFYRPKFFLLENVRNFVSFKSSMVLKLTLRCLIRMGYQCTFGVLQAGNYGVAQTRRRAIILAAAPGEKLPMYPEPQHVFSPRACQLSVMVDEKKYNSNTQWRQSAPYRTITIRDTMSDLPSIRNGHSATEISYDGEPRSYFQKIIRGNQYQPILRDHICKDMNALVEARMRHVPLAPGSDWRDLPNIVVRLSDGSSSKLLQYTHEDKKNGTSSTGALRGVCSCAEGTACDQNDRQFNTLIPWCLPHTGNRHNHWAGLYGRLEWDGFFSTTVTNPEPMGKQGRVLHPEQHRVVSVRECARSQGFPDTYRFYGSILDKHRQVGNAVPPPMAAAIGREIKKCLEDKAKKETQTGETKPVSMEA

>Bflo_DNMT3_model_PK

METPEEEPSGNLHNNMEVDDDATPDPNSLATVTNSDASSKPPHNDASPRTSQPTETVFITEDDMDLGALSELEEAVSDDESSTYSFIPGDHSQGPEVPEDDVEDLLESVNISPIIGSSNAVGGTDCQKKTCRRSLFEYHGDNDESAICKGEMQEDINQNLRLVLNTSRSSEEDWTTPLKPLVADYSSPGMEGMVEMPEPFSQDVEGDQLLSDYMSGIEPILTGHFEVTKGSSFGEEIPPKQNEQEADNLSREVEMQTSQPIDSGTTQIQTVSPLVEQTKEQEYHQQVMIRKTPSGNILRSSRCLSSTTSDDGSEKAAGKGGTPSGGKDENLERETERDAVSSVTKGKDVGQGVLSADPGHAEAATQPPASAQAGVQHTSVTRLKQGQRKRRAHADHSYSSPQQLLLSKKRQAKEEAIQKLTTKGRKDKKSSASCPSAVEVGENNDHSYSKTFKQKKKGGEDERQTGYQKKQQTAQDGQRGLPAQNLLMSKIQQLSGSRLVLPASSASSHVKSVEGRKKIKFLVAMEEKHNALPSGVNMNSSKDRIIENENADAAHTRNVQRGKQKTKTNARMTLTPFSRKTSEAKLIEETETLSETQDTMDNVIPPSQTDDTMKKKYVVSPRGAQGKEKTATSMSGTRHVETQSRDKQITQNDDTIQGQTEEAKEVQKQGIASDEHHKRAAGKRPTQEILERDETGNKKKARTSTPEKNKLEPFHMEHTRGMFGWEDSLRQRLSPVKRFQPEAPPNADDKGKKRKKEEQGSAVKKKMKMQPLAFTTIRSNPAISSKGNLAVPKDAPAASAAASTSSAPVTAPLTPGATSTSSIPENCTPTDKSQEEQLPQEYKEGTYGMGELVFGKLKGYTWWPGRVVSCVETGRDPAPQGSCWVRWFGDGKFSIVQVEDKLEPFGKFSQLFHERTYNRLQTYKRAILESLQVAADRAKKKFLSSPEANKNQDRDRAMVQWALQDFSPAGPDSLKPSAEEARPPDHLVHQWLQVRHLKQLEQVAEGRSSGSGSRRESVDSRLSTAKLFEEVRKRQLSVDQVCLACGSLDVETQHPLFRGGLCEGCRIEFLESSYQFDEDGYQSYCSLCAAGTEVLMCGQDSCCRSFCKDCLDLIVGPGTADQAVLEDPWLCYMCREEPRHGLLHRQQDWSARLQQFFAMDDVEDFGQPKLYPLVPAQQRRPIRVLSLYDGIGTALLVLKELGVTVDVCYASEIDPDAIKVSQLRHAGGIRHLGDIRSIRNTDIPKLGPFDLVLGGSPCNDLSIANPNRAGLYGTGVMFFNFYRLLREAAPAPGDDRPFFWLFENVVAMKHQYKQDISLFLGCNPVVVDAKDVSPAHRARYFWGNLPGMNRPLVPQASDKLALEQCLEKGCGRVAQVKKVRTVTTTLHSVRNQGKQQAAPVLMEGQPDTLWCTELERVFGFPSHYTDVCNMSRCSRQRLLGRAWSIPVIRHLLAPLKDYFQCQDKE

>Bflo_XP_002592995.1

MHSENDQTDPLRVVEFYSGVGGMHYAVLESKVPATVVAALDINTTANAVYRHNFPHVNLLQRDITGIKLPEFQSWNADVFMMSPPCQPFTRVGLKGDTADPRTKSFLYILDILRRMAEPPNHILLENVKGFETSETRGNLVTTLDECGYCYQEFLLSPNQFGIPNSRLRYFLLAKRKPLTFVFEHQTEIMKEMPVNSLIGQAPANQDASSSEVPNQSTSQNKQEIPCPYSIFDPEDRDTTNCREISEFLEPHSKEEEESLLLQDKLLDRYWKVLDIVTPSSRRSCCFTKAYGHYVEGTGSVLFSQTDIDAKDIFTKVSKSEDQSERLDLLHQLKMRFFSPREVASLHCLPPEFTFPQATTTKQRYRVLGNSLNAHVVAELFKLLVK

>Bgla_5185

MGPINEWFVSGFDGGENALIGFSTGFAEYILMQPSEAYAPFIDQMKVKVHMSKIVIEFLSNNPDALYEDLLNKIQTTVPPAGLTSFSEDSLLRHAQFIVDQVQSYDDAAENDEFLLITTPCMRALIKLAGVTLGKRFETLPSSICQQPDCGKCTACKDMTKFGGTGRAKQACKERRCPNMAVKSAEDDDLEEETEIDKEIEEEKCISTAPKHKAAKNIKTQVQWIGDSINEKGKRMYYSKAMLNEFEIAPGDYVSVTPSDPTEPYFIAQIQYLYEDASGKYAHINWLYRGSETVLGEASDPLELFISNDCNDNDLVTIHNKVTVIQKTVPIDWNMKGGIDNPDEDSVVKEDDGKSFYYQKFYDPEFGRFEDPPVLKCAEGQIVCSCCERVELHKKLMETSLGTKAEVDNAQSGYTYYSSVSKDGDTYKVGDCCYLDPEAFIFSIKHPKVAPRTTKDKHIDEDIYPEAYRKSEYVKGSNDDCPEPFRVARIVEIFVKQDKNKQSDVQDVKIKVNKFYRIENTHKGQQAVYHSDLNKLYWSDEQVTVTISYIRGKCTVMYQENLLESLDDYFLGTSDRFYFNESYNSQSREFEDPPRIARLMGSKGKGKGKGKGKSSDKQDTQTEESKKVEIHKLRTLDVFAGCGGLSEGLHQAGMAETCWAIEKEQPAAQAFRLNNPGCVVFTDDCNLLLQRVMQGEKTNELGQKLPQKGDVELLCGGPPCQGFSGMNRFNSRDYSKFKNSLIASYLSYCDYFRPRFFLLENVRNFVSFKRSMVLKLTLRSLLRMGYQCTFGVLQAGSYGVAQTRRRAIILAAAPGEKLPMFPEPMHTFAPRAMQLSVMVDEKKFVSNIKNTSSTPYRTITVRDAMSDLPEVKNGAKAEEISYKGDPETHFQRLIRGNQHQPILRDHICKEMNPLVAARMMHIPLAPGSDWRDLPNLELRLSDGNKTKKLLYPYKDKRNGNGSNGQLRGVCTCATGKACEPLDRQFNTLIPWCLPHTGNRHNHWAGLYGRLDWDGFFSTTITNPEPMGKQGRVLHPEQHRVVSVRECARSQGFPDTYRFFGNILDKHRQVDKLE

>Bgla_XP_013095826.1

MKLKVLELYSGIGGMHFALKESHLEYEIVLAIDINTTANKVYKHNFADTNLQSFGIEKLSLKMLEDLQINTILMSPPCQPFTRVGKKRDCHDVRTKSFLHILDLIKLIKVKPVYILVENVKGFETSETRGQLIECLKYCNYTYQEFLLTPLQFGIPNSRLRYYLTAKWNQCLMTTATGNYSILTELQDFSSFLKDSVTAHEQEAVKNTNFLVNDNSESLKPICARAQSSDLQGVKTQDSQQSSDSLQNEAKSSLKDFGRNMRYCEEDDKFINDKSLSLSEYMETSDNIDLQDYWLSDKELRMFVIMDVVYPELKKSICFTKRYGHYIEGAGSVVQMSSDIQHVIAASDLKHQAVNSLNRNEWGESEMEILRNLHLRYFTPREVANLLCFPKDFSFPDGLTKVQKYRVLGNSLNVFVVTQLIQFMTKIL

>Bmal_CDP98269.1

MIDKQQNASFFCARSGEPLKCLEFFAGIGGFHYALKEIGLHLVIRALDINTVTNAIYKYNFPNTEIQQCNIEKLTTDYYDDYNADIWTLSPPCQPFTRKGLRKDVADHRCNALKKICNALKSMKKPPRYIIVENVCGFEASEAHHLLTDTLINLCYNFEEYIISPTKIGIPNSRPRYYLLAKLANNCIAIPTTSKIIDNWPKDDMAVLRSKVIGEYLCNEANEDNSLVILPEIVQRFGNVMSFVTPYNIHSSCFTKSYYRYVTGTGPILLQFSNNIQDQCISIEQLRDFVLYDNCKNLKYCQIRYFSWREIANLLGFPKTFAKPYEISSKQMYHALGNSITVSGVALLIQHLLKI

>Btau_NP_001193431.1

MPASGPGDTSGSALEREEERKEGEEQEEARAKEERQEPSTTARKVGRPGRKRKHPPVESSDTPKDPAVTSKSLSMAQDSGPSELLPNGDLEKRSEPQPEEGSPAGGQKGGAPAEGEGATETPPEASRAVENGCCTPKDGRGAPAEEGKEQKETNIESMKMEGSRGRLRGGLGWESSLRQRPMPRLTFQAGDPYYISKRKRDEWLARWKREAEKKAKVIAVMNAVEENQGSTESQKVEEASPPAVQQPTDPASPTVATTPEPVGADAGDKNATKAADDEPEYEDGRGFGIGELVWGKLRGFSWWPGRIVSWWMTGRSRAAEGTRWVMWFGDGKFSVVCVEKLMPLSSFCSAFHQATYNKQPMYRKAIYEVLQVASSRAGKLFPMCHDSDESDTAKAVEVQNKQMIEWALGGFQPSGPKGLEPPEEEKNPYKEVYTDMWVEPEAAAYAPPPPAKKPRKSTTEKPKVKEIIDERTRERLVYEVRQKCRNIEDICISCGSLNVTLEHPLFIGGMCQNCKNCFLECAYQYDDDGYQSYCTICCGGREVLMCGNNNCCRCFCVECVDLLVGPGAAQAAIKEDPWNCYMCGHKGTYGLLRRRDDWPSRLQMFFANNHDQEFDPPKVYPPVPAEKRKPIRVLSLFDGIATGLLVLKDLGIQVDRYIASEVCEDSITVGMVRHQGKIMYVGDVRSVTQKHIQEWGPFDLVIGGSPCNDLSIVNPARKGLYEGTGRLFFEFYRLLHDARPKEGDDRPFFWLFENVVVLGVSDKRDISRFLESNPVMIDAKEVSAAHRARYFWGNLPGMNRPLASTVNDKLELQECLEHGRIAKFSKVRTITTRSNSIKQGKDQHFPVFMNEKEDILWCTEMERVFGFPVHYTDVSNMSRLARQRLLGRSWSVPVIRHLFAPLKEYFACV

>Btau_NP_861528.1

MEPLRALELYSGIGGMHQALRESCIPAQVVAAVDVNTVANEVYKYNFPHTQLLAKTIEGITLEEFDRLSFNMILMSPPCQPFTRIGLQGDVTDPRTNSFLHILDILPRLQKLPKYILLENVKGFEMSSTRDLLIQTIENCGFQYQEFLLSPTSLGIPNSRLRYFLIAKLQPEPFPFQAPGQVLMEFPKTESEHPPKYAINAEKKTEEKKTGPKICFDSSTQCSGKEAILFKLETAGEIDRKHQQDSDLSVRMLKDFLEDDIDKHSFFLPPKSLLRYALLLDIVKPTSRRSMCFTKGYGRYIEGTGSVLQTTEDVQIENIYKSLTSLSQEEKIMRLSMLQLRFFTPKEIANLLGFPPEFGFPEMTTVKQRYRLLGNSLNVHVVAKLIKILCD

>Btau_NP_861529.2

MKGVDSLINEDKHANRREDSVITDGAVIAQCCDSKQSPSPRILQSISTLEIIGARGVRGRRSSSRLSKREVSSLLSYTQDLTGDGDGEGEDGDGSDTPVMPKLFRETRTRSESPAVRTRNNSSTSTRERHRPSLRSTQGRQARNHVDESPVAFSTTRSLRRRTGSSAGTPWPSPASPYLTIDLTDEDVVPQSSSTPYARLGQDSQQESMESSQLDADGRDADSTEYQDGKEFGIGDLVSCGGKIKGFSWWPAMVVSWKATSKRQAMSGMRWVQWFGDGKFSEIPADKLVALGLFSQHFNLATFNKLVSYRKAMYHALEKARIRAGKMFPSSPGDSLEDQLKPMLEWAHGGFKPTGVEGLKPNNKQPENKTRRRTADDSATSDYCPPPKRLKTNCYNNGKDRGEEDQSREQMASDVASNKGNLEDSCLSCGRKNPVSFHPLFEGGLCQTCRDRFLELFYMYDDDGYQSYCTVCCEGRELLLCSNTSCCRCFCVECLEVLVGAGTAAEAKLQEPWSCYMCLPQRCHGILRRRKDWSVRLQAFFTSDPGLEYEAPKLYPAIPANRRRPIRVLSLFDGIATGYLVLKELGIKVEKYVASEVCEESIAVGTVKHEGNIKYVNDVRNITKKNIEEWGPFDLVIGGSPCNDLSNVNPARKGLYEGTGRLFFEFYHLLNYTRPKEGEDRPFFWMFENVVAMKVGDKRDISRFLECNPVMIDAIKVSAAHRARYFWGNLPGMNRPVIASKNDKLELQDCLEFNRTAKLKKVQTITTKSNSIRQGKNQLFPVVMNGKEDVLWCTELERIFGFPVHYTDVSNMGRVARQKLLGRSWSVPVIRHLFAPLKDYFACE

>Btau_XP_010822784.1

MGPGPSGCSKPGEGVSEVSSNSLGSGAGGHGGSVSSGWPGSFGRCLAAALEVSTRAPVEFWTFHCAGSFHSSPLPASNPERPGGGLRNHRRAQSPPAFKSSWERPRLDGIWETLGRQEHGVAEGPSAGGTQTEEVPRRTPGPGLKQDSRIPRLRDGGWWGTFSLSAVSPISWFGVPLSATLVLDPEPEHSLDIILVGSSELSSPPSPGPRRDFIAYEVKVNQRDIEDVCICCGSLQLHTQHPLFEGGMCAPCKDKFLECLFLYDDDGYQSYCSICCAGETLLICENPDCTRCYCFECVDTLVGPGTSGKVHAMSNWVCFLCLPFPRSGLLQRRRKWRTWLKAFYDREAESPLVMYKTVPVWKREPIRVLSLFGDIKKELTSLGFLEDGSKPGRLKHLDDVTNIVRRDIDEWGPFDLTYGSTPTLGHTCDHPPGWYVYQFHRILQYARPLPGSPQPFFWMFVDNLVLTEEDLDVATRFLETDPVTIQDVRGRTVQNAVHVWSNIPAVKSRHSALVSQEELSLLAQDRQRVKSPAQGPATLVKNCFLPLREYFKYFSTELTSSL

>Btau_XP_015327477.1

MPARTAPARVPALASRAFSLPDDVRRRLKDLERDSLTEKECVKEKLNLLHEFLRTEIKNQLCDLETKLHKEELSEEGYLAKVKSLLNKDLSLENGAHAFSREANGCLENGSQTSGEDCRVVMAEKGKPPKPVSRLYTPRRSKSDGETKSEVSSSPRITRKTTRQTTITSHFPRGPAKRKPEEEPEKVKSDDSVDEEKDQEEKRRRVTSRERVAGLLPAEEPGRVRPGTHMEEEGRDDKEEKRLRSQTKEPTPKHKAKEEPDRDVRPGGAQAEMNEGEDKDEKRHRSQPKDLASKRRPEEKEPERVKPQVSDEKDEDEKFWRIQFTYQSTSREEKRRRTTYRELTEKKMTRTKIAVVSKTNPPKCTECLQYLDDPELRYEQHPPDAVEEIQILTNERLSIFDANESGFESYEDLPQHKLTCFSVYCKRGHLCPIDTGLIEKDVELLFSGSAKPIYEDDPSPEGGINGKNFGPINEWWIAGFDGGEKALLGFSTSFAEYILMDPSPEYAPLFSVMQEKIYISKIVVEFLQSNPDSTYEDLINKIETTVPPCMLNLNRFTEDSLLRHAQFVVEQVESYDRAGDSDEQPIFLSPCMRDLIKLAGVTLGKRRAERRQTIRQPAKEKDKGPTKATTTKLVYQIFDTFFAEQIEKDDKEDKENAFKRRRCGVCEICQQPECGKCKACKDMVKFGGSGRSKQACQKRRCPNMAMKEADDDEEVDDNIPEMPSPKKMHQGKKKKQNKNRISWVGDAVKTDGKKSYYKKVCIDSETLEVGDCVSVIPDDSSKPLYLARVTALWEDSSNGQMFHAHWFCAGTDTVLGATSDPLELFLVDECEDMQLSYIHSKVQVIYKAPSENWAMEGGVDPEALMSEDDGKTYFYQLWYDQDYARFESPPKTQPTEDNKYKFCASCARLAEMRQKEIPRVVEQLQDLEGRVLYSLATKNGVQYRVGDGVYLPPEAFTFNIKLSSPVKRPRKEPVDEALYPEHYRKYSDYIKGSNLDAPEPYRIGRIKEIFCSKKSNGRPNETDIKIRVNKFYRPENTHKSTPASYHADINLLYWSDEEAVVDFKAVQGRCTVEYGEDLPQCLQDFSAGGPDRFYFLEAYNAKSKSFEDPPNHARSTGNKGKGKGKGKNRTKSQTCEPSELETEIKLPKLRTLDVFSGCGGLSEGFHQAGISETLWAIEMWDPAAQAFRLNNPGSTVFTEDCNVLLKLVMAGEVTNSRGQKLPQKGDVEMLCGGPPCQGFSGMNRFNSRTYSKFKNSLVVSFLSYCDYYRPRYFLLENVRNFVSFKRSMVLKLTLRCLVRMGYQCTFGVLQAGQYGVAQTRRRAIILAAAPGEPLPLFPEPLHVFAPRACQLSVVVDDKKFVSNITRLSSGPFRTITVRDTMSDLPEIRNGASALEISYNGEPQSWFQRQLRGSQYQPILRDHICKDMSALVAARMRHIPLAPGSDWRDLPNIEVRLSDGTLARKLRYNYHDKKNGCSSSGALRGVCSCVEGKPCEPAARQFNTLIPWCLPHTGNRHNHWAGLYGRLEWDGFFSTTVTNPEPMGKQGRVLHPEQHRVVSVRECARSQGFPDTYRLFGNILDKHRQVGNAVPPPLAKAIGLEIKRCMLAKARESASGHIKVMFGGFLDGLVVKNLPASAGDTDSIPDPGRSHMLQSN

>Caqu_C89379_a_6_0_l_1455

MYFRGGGTEDSSSGSSSVAEEPLKVVELYCGIGGMHYALEKAGLNAEVVCAMDINNVTNSVYKRNFTETKLLAKNLESITAEDINRYQPDMIMASPPCQPFTRNGKKLDDKDPRAASLLHLIELIPQLDECVRYILMENVAGFDCSNTHSLLKKELEESKFKTQEFLLSPHVFGIPNSRLRYYLLAKRDGTFSFPDASGLHTTLPYGQPGMKCVSDLLEQLKLHWTEGEWEHCLPIGHFIDHPRDSVKGFDIPGEILRDKGHLMDVVSPDSHRSCCFTRAYTRYIEGTGSVLQEPTKFTRYFTPTEVARIMGFPEEFDMQDMNLRQSYKALGNSLSVVTVAVLALMLLQEPEEGQEPRKTRKQQAGLKNKTNTSVQLRSTQKLDSKVPTKRH

>Caqu_CAQU001464

MGANQTNGNEKEVVLPDDPPKEEEQPAKEKISGQPRPKSHPKKRHWESVRGNSPEPPILTPIEKHEPDLPENTTEENKEEKDPSLTHRPPRLSIAVPKRRHIAKKSSAKSLKEAVDVTDKNSMEKEVSDAKEETEVPKKGGNGRGSNGWEDSLRPRPSQVDHLQSFMLQSILESEVGRIRKPKKKKYSKASKEKKKSKQKPATKTVERNGKAEQVPGHILKELKIPIMKVHVVDKDCLVPGPIDPVKSKEGDQIGSNGEKVCKEQDAAALPNNNNATECLPCPTNTPSDKSAKPSDQESAQMVFTCSSKESGHSAVKKRKKSSHSSGPDSKKLKKKSDKGRKNKKKSSSSLSPLPASEEGKKKHKEKSHKKKDSKKVECDKLVGFTSHFKRKYSKCSFKTFKLALKEALRECANRADPERAGGNYAELLSWASQGFLSEKGPFDAQRFQPNPDNPVPEIVVGHLRKIKRKNLFYYGYQHEALPSVEDPVQLEVPVESVSTKGDAMSKVREGTLKIDEICIACTTTSAEVVGPHPLFVGGVCGKCQDGLIETLYAYDDDGAKVLQLFNPKNQLLIPNALAFEEKRPMRVLSLFDGISTTLHVLEKLGLEVEAYFSCEVDPDAIAVSTSHHHQRVQQLGDIEELLEVQVQKLCPLDLVLGGSPCNDLSLVNPARRGIYDQQGTGKLFFDFYRVLKAAQIGNQGRHLFWLYENVASMPLEYKKVINRFLGCEPAVLDARFFSPQQRVRYFWGNIPGLHTPLQPHLLSRRVSIEAALNLPKLVPERATSPLNNLMPASQQAILTGDGDSMKVTELEESIGLPKHYTEVGNIPPGKRQKLLGKAWSVPVVAHVLKPLRNFFKICATSSNSQEEVHKEDSKADENVESSMEDKVDNIVEDKVESNTENVVETKAPDQVENKVEGEVESKEEAEVKDKSENN

>Caqu_CAQU007561+CAQU009352

MTNFSVYDKEGHLVPFDSGLIEEDVFLFFSGYVKPVWDENPNIEGGIAAKEVGPINEWWISGYDGGEKALVGFSTSCAEYYLMEPSEEYAPFMSQVLEKIFLSKVVIEFVQHHEGAEYEDLLTKLQLENQEEKVSKAPRRKRCGICFNCQKRDCGNCVACKDMVKFSGTGRSKQACLSRRCPNMAVQMADDSDGEDHDNESAQQKPKEKTPTKQAELPLPISPFREMSWIGEPIKIQNDRSYYLAETADFHEVFPSGHCSDWEMEKINYKVNVFYKPIPENWKTLGGVDMGPEEKIDEKTFWYQKWYDTKTGRFMDMTERIPEPDMGKTCPHCTLLLYQRAIWEHLDLAIGDCVYIPPDIYDYKVKPVAPKPEALKKKEDVDEDLYPEYYRKAADGHAKGNHDGTPEPFRIARIEYIMSRGAPDRDNVYLKVRKFYRPEDTHLGVPATAHSDLNLLYWSEEVADVKFSCVMGKCHVVYRENIVGSVDEWSAKGPNRFYFAEIYNTQDKTFEEPPMKAQAMGRIGKGKGKGKGKGKGKSSGASENVEANPAGSGPTLEEWPTVKKLSCLDVFAGCGGLSEGLHSSGVTETKWAIEKEDIAAQAFRLNNPNAIVFSDDCNELLKLVMEGKETNRLHQRLPQKGEVDLMVGGPPCQGFSGMNRFNTKEYSAFKNSLIVSYLSYCDYYRPRFFILENVRNFVAFKNGMVLKLTLRCLLKMGYQCAFGILQAGHFGVPQTRRRAIIFAAAPGEKLPAFPEPQHCFNPRACQLTIMVDEKKIRGKQAQPLLYDHVCKDMGELIEARIAHVPTSGGSDWRDLPNLEVRDRSNNVIAKKLIYSYNDAKSGKSSTGALRGVCRCAIGGKAGCIASDRQDNTLIPWCLPHTGHRHNNWAGLYGRLSWDGYFSTTTTNPEPLGKQGLVLHPEQHRVVSVRECARSQGFPDTYRYAGNILDKHRQVGNAVPPPMGTALGYEILKCLRPEEHLVNGN

>Cgig_EKC23761

QHKITNFSVYDKNTHLCPFDTGLIEKNVFLYFSGVVKPIYDENSSPEGGIRACKMGPINEWWTAGFDGGENALIGFSTAYAEYILMSPSEAYKPYMDTMREKIHMSKVVIEFMQNNQEATYEDLLNKIQTTVPPTGLSSLTEDSLLRHAQFVLDQVQSYDEAAEEDEGLLITTPCMRALIKLAGVTLGKRRQMRKELRKTKDKVKKPAFTMATTTRLVTQIFDSLFQGEIDDKSGQGSKRRRCGICEICQQPDCGKCTACKDMVKFGGSGKAKQACINRRCPNMAMKEADEDDILDDDDTDEKLETTKLSWVGDPVLQDGKNSYYSAVLINDEKVSFGDFISIKPEDVAIPVYIAMVNYLWENASGNKMCHVQWLCRGSDTILGETGDPLELFFVDDCESIKLESSLRKVKVLHKETSPDWFMQGGIEHPEKDFPIEDDSNTFYYQKWYDPDLARFEDIPTSDKPKDQRHHKFCLTCDRLDQLRKKENPSLGPEIESEDSSATRTFYSSVGRDGFEYKVGDCCYMEPSAFGFNVKQPPAKKVKTERKEVDEELYPEGYRKGDYIKGSNESVPEPFRVGRILAIFCKKSSGSKIANLGEIKVKVAKFYRPEDTHKGPSAGYHTDMNMLYWSDEEATVDFKLVKGKCQVTYSENLRSLEEYLCGGLDRFYFTEAYDSETKMFEEPPRQARVSGGKGKGKGKGKGKSSGPSAEEETTEPKKPTYRRLRTLDVFAGCGGISEGFHQAGIAESCWAIEKEEPAGQAFRLNNPGCTVFTDDCNVLLKLVMEGEKQNEMGQKLPQKGDVELLCGGPPCQGFSGMNRFNSREYSRFKNSLIASYLSYCDYYRPRFFLLENVRNFVSFKRSMVLKLALRCLTTMGYQCTFGVLQAGSYGVPQTRRRAIILAAAPGEKLPFFPEPQHVFAPRAMQLSVQVDDKKFMSNITRMESAPFRTITVRDTMSDLPEIRNGAKAEEISYQGDPQSHFQRIRLSGHWAVK

>Cgig_XP_011435619.1

MSEVNKEMKISPLAWLHREFSLPPSWTSQFVMQGKEKFTIFKGFDIPTQARSYSDFSLPNFLHDSCSSSAKSDPNSVSGPIDFPESATAAKLPGKARKSSRDRRFNIVADFASSGKSMRRKPKTDEFRQHRDSRRPFMYRRLKLQKMKLKTTLTNKKLQCHISCTTSLYSVVRRPKGSESSPQKKEGHSDERGTESKSYKEKGGPKKSSQGYSYDNRRKTRSSSESTASSMTDDVASSMTDDAMPSDSESVLESITSSSSAEENQDSQVGNIVFASLKGSASISRWWPAIVIEGKEVNKSEKSGFLWIFWFGDHRISQVMASNIGDFASNFTKFGLCNKAVKNCPNFIKGINEAIQICAERIAHNTEDMTEEELLGWAKEGFPRGQGHFFPDPDDPLPQMVKTYLTKIKDNLKLVDTSKKRSPNKSAVKALRAGEKELSEICLSCDDIENVEVEDHPLFFGGLCKSCKEEWKESIHIYDDDQTNTFCVICSTGGDLVVCEDVNCGKSYCVECIKNYLSEGQLKKIKESQVWNCFLCTLYNKSTHGYLIPRPNWRDKTVHIFDSGYRTDMLPSFFKGPKQAVRALSLFDGISTGKVVLDNIGLDVEEYYASEIDDDAILVSMVNHRDSVQHLGDVRSLTREKLDKLGRIDLLIGGSPCNDLSIVNPARRGLSEEGTGHLVFEFGRVLEYLQSVSRARGQHLFWMFENVASMRADIRDTISECFKCPPALWDAKYVSPASRARYFWGNIPGMYSTPFLKNPDSIKDFHLNDVLYLQNIRSSRYKTVRCITGKRNSLKQGRNEDQFVILMNGREDNMWITEIERVFGFPEHYTDVGNMSVTARHKLIGQSWSVPVVEGILSPLRKFFRTKK

>Cgig_XP_011450725.1

MRVLELYSGIGGMHCAVKECGISYEIIAAIDVNTTANKIYKHNFPESNLMDCGIESLSATQLDEMKIDMIVMSPPCQPFTRVGKKLDAEDIRTKSFIHLLSLLPRLNNCPKYILVENVKGFEDSETCLKLRETLMKCNFTYQEFLLTPLQFGIPNSRLRYYLIAKRSPLKFSFANTTEIMTSLPSINSEENSSSTNATNISQPSTACISDESTRTNDHCKNSSHISSSHHMQSSPAKPAKNKETQHGMLGKSINNSDATEKKSPDIPAATCPICRESCLQSAGHNMRQLSEGEEEEEDKYPCCLVQDFLEDQPPEYFQPFMLTRKDLKRFIVMDIVFPCLQKTTCFTKRYGHFMEGAGSIFQMSHSISEMTAASELKDRTLELKNRDQWSDEDYQVLSRLRLRYFTPREIANFLCFPATYNFPQDLSKIQLYRTLGNSLNVRVVSKLIQLMVKEQTSHVQV

>Cint_XP_002122948.2

MKCRECRQLLDDVAIFGGDVDQAEEEFVLLTHESLSLFVGNEETGRPQHKITDFTVYDKSTHLCPFDAGLIEKNVELYFSGLVKPIYDEDPGVGTGIPGKRLGPINEWFTAGFDGGEKALVGFSSAFADYFLMEPSHAYAPLWASIQEKIHMSKIVIEFLSDVPDADYEDLVNRIQTSVPPPTLGVAKFTEDSLLRHAQFVVEQVESYDNARSDEEDSLLGTTCVRDLIHLAGVTLGKRRVARRSAVVKEKAMKASSTKATTTRLVAEVMEKFFGGTVDVTTGNKIRRRRCGVCEACLKPECGKCTSCKDMVKFGGGGKLKQTCIDRRCPNLGLDLMEDSDPDDKDEKVKPSPSKMKVIKAGKVTTSWVGEALYEEGGKVYYPGVRVGGDVIEGGECVSVKNKDEGGLPYIGRVVYMWGESGGKMVHVDWFMRAADTILQETSQPDELLIIDECDDLSVECIAGKVDVSHRVPHQNWSFMGGVEEQHPTTDGVSSFYYQKMYVPEFARFVDPPSNEPTDDPDFCPSCVRINEGKMRQVAVGKNELKRENGKIFYEFVCKDGVEYGVGDSVYLSVETYSFKSKNKHKKQVLPKSLDEIDEDEYPEFYRKGGYVKGSNLEVAEPFRIGRINEIFGGKKSISLVVTKFYRPENTNKPLKSNSYCDLNLLYWSNDKVEVDFSFVEGKCEVECELDLSVTVAEYSRKAANRFYFSEVYNEEDGTFTVPPSTARRSGVELLKQKAKGVKPKLTKMNKMMKKDETIVGEDETKIPKLRSLDVFSGCGGLSEGFHQAGIAEPSYAIELWEPAAQAYRLNNPGATVFTEDCNVLLEMVMNGEERSKCGQRLPQKGDVELLCGGPPCQGFSGMNRFNSREYSRFKNSLVVSYLSYCDYYRPRFFLLENVRNFVSFKNCMVLKLSLAALLRMGYQCTFGVLQAGHYGVSQTRRRAIILAAAPGEQLPLYPEPLHTFSTRGGSLSAQVGDTRYTNNIKWSTSAPYRTITVHDAMSDLPKIPNGHAKLEMPYTGEPLTHFQRMMRGKDYQGVVRDHICKDMSPLVAARMALIPLIPGSDWRDLPNKPHKLSDGTTAKVLRYEYHDKKQGRSSTGGMRGVCSCAEGRACDAMDRQFNTLIPWCLPHTGNRHNNWAGLYGRLCWDGFFSTTVTNPEPMGKQGRVLHPQQHRVVSVRECARSQGFPDSYRFFGSILDKHREVGNAVPPPMSKAIGLQIKKSLEWKVQQ

>Cint_XP_002128135.1

MEPELKVLELYSGIGGMHYALLGANLKNCEVVCSVDISPAASLVYKHNFPGTKHWERSIEGFSAKDFDNMGFNTLMMSPPCQPFTRVGLQKDINDPRTRSFIYLMKVLPQLSNPPTYILMENVKGFENSKAHDMFLQVLEQLEYSTAQFLLTPKQFGVPNSRLRYYLLAKRKPLQFPDEVNTPNEVIETMPQALQKCLNSNAGKEQNEKHTIKTLENYLEEAESTLKEHALTEKTLLRYLQVMDIVTPRHQSSTCFTKSYGYYAEGTGSVLNMGGEIKMSNPLHNNLRYFTAREVANIMCFPKDKFHFPENFTRKQKYKLLGNSLNVYVVSCLLKLLITDTKR

>Cint_XP_009861613.1

MWSMDKRARRKPKYFEAGSGGKGPHENEKDPKQQYKTKPSPEDVKFVPPRRIIPREDLLVWGKCRGFGWWPGRIANTGELSGPIDPPDDPTRWIKWFGDEKFTETKVDDMAYLHDFNAYFKEKSYNRNRSYNLAVDLAIKEAAHRSGKTLESNASGITWAKEKFYPEGYLSILPKGYTDDQKRNMKGVKQSELNGSPEKKVKTTISKPEMNCQDFTKQRSEVFMKVREGLLDIANTCLGCGNLEVSTQHPMFVGGLCQMCEVDYRESCYLLDDSGYSCYCCVCGGGKRIFLCDNQSCCRSFCFICLELMMGEGTTKTVLHQSPWSCYLCVATEKGLLHRRDNWHNRLSKFVSVGEVEYEIVSLPKSPPAWERKPIRVLSLFDGIATGFVSLKQLGIEVKVYFASEIDHEAICVSEIRHPRVVKHVGDVCEITDKMLSDWGPFDLVIGGSPCNDLSIVNPARKGIWSDKGSGHLFFEYLRILNTCKVLAAKEGRSLFWLFENVVCMDQSSRQTISHYLQRNPIVLDAKEVSPAHRPRCFWGNLPGMRRPMVATKAHKLTLEECLEPGRKATTDKLRTITTKLQSVRGGHGSGGLTSNSLVIQQRDQHDCLWITEIERVFGFPDHYTDVCNLPRTGRLRILGKSWSVPVIKHIFAPLKDYFSSV

>Cint_XP_018670748.1

MCVVRAKLKPVMPLLTLGVWAASSFSWFRFLFCKDLLFSVFLYKILRLWDIISLNMSRKKERRSKKVIVISSESENEQKLKPSSTLEKQYLLTNPTQSNHETQNNNNCDVIDSIVQTLKAKLSKKHKKKKKKRRRSRRLSNSGVEFSETLINEYNPSRGLRKRRKSVELFQADTSCISKHKKRKSTSFNEVQDVDNGIQYLLHNTPVVGVNQLENGTEEEEEASIEFNPNIDAATQLPIEEITILDDVEVCGEADKQDQVEADPPLYDEGLNNKSKHDVGLYFEQKQKVFRKVIEGVLDINDICLTCGSSENITDDHPLFQGGLCETHKTMFQDWFFLCDKDGCQSYCTICCMGEKVVLCDNEDCGRCFCTDCLDLMVGAGQYDKLVPLDKWSCYVCTNDSSCSLIVKREDWQEKLRLLHPEAKYQKLVDVPKPMSVGERKPIRVLSLFDGIATGVVALKQLGLQIQKFVASEIDEAAIRLVQNRHPEVMHVGDITKLTDDDITRYGPFDLVMGGSPCNDLSGANPRRKGLLDQEGTGCLFFDFFRVLRAAEPPRCDCDNKEHQRPFFWLFENVLSMKMVERERISRFLQCQPVVANGRAVSAAARPRLFWGNLPGLQSLTLTPSPGDRLTVQSCLEHGRLATIEKLNTITTRSHCLYQGHTKQAPVMHMGKEDVLWSTEVERIFGLPEHYTDVNNHGPKERLALLGRSWSVPVVKRILAPLRDYFACGEVLEKI

>Cmil_AFO94067.1

MPARTGSAAAASSPSSSSPLPEDIKRRLQALEDKVDGLTEKECIKEKLSLLHGFLQADVQNNLNELETKFQKDELSEEGYFAKVKVLLSQEPPVENGDSELGPNANGCAENGARSGKKDDDRDKANGVAGERNGQEQNLMETGGEDPTPQAAKSRGKRQSKSNGDNRRTSGSPRITRNSMKQQATITSMFAKAANKRKSEELNDEEQVPVKKEEESEEQEEQDEKRIKVESEETLPEGAETNADRKPKADQTPKTPPPKCVDCKQFLDDPDLKYFQGDPDDALEEPEMLTDERLSLFEGTHDEGFESYDDLPQHKVTSFSIYDRKGHLCPFDTGLIERNVELYFSGVVKPIYDDNPCLDGGVKAKKLGPINAWWITGFDGGEKALIGFTTAFADYILMDPREEYAAIFAVMQEKIHMSKIVIEFVQNNPDSTYEDLLNKIETTVPPAGLSFSRFTEDSLLRHAQFVVEQVESYDEAGDVDEQPIIIIPCMRDLIKLAGVTLGKRRAARRQAIRHPTKIDKDKGPTKATTTSLVYQIFDTFFSEQIDQNDKENGLKRQRCGVCEVCQQPDCGKCRACKDMVKFGGSGRSKQACLQRRCPNLAVKEADDDENDEEDSETVGEVCPKKILQARKKKQSKNRICWIGEAIKSDGRKTYYQKVSVDDELFAINDCVVVSPDDPTKPLYLARITSMWEEGVCKMFHAHWFCRGTDTVLGETSDPLELFLVDECEDMQLSYVDSKVTVIYKGPSKNWELEGEPENHFELKVVDDDGKTYFYQMWYDPEYSRFQMPPSCEPTEENKHKFCASCSRLAEIRQREMPRVMESVDAKDDSKVFYSLASKNGTQYRVGDGVYLLQDSFSFSVKPSSPGKRPIKKDDVDEDLYPEYYRKSSDYIKGSNLDAPDPFRIGRIQEIFCHKRSNGKPNEADIKLRINKYYRPENTHKGLKASYHTDINLLYWSDEEVVVDFKDVQGRCRVEYGEDLTTENIHEYSAGGTDRFYFLEAYNAKTKSYEDPPNRARSSANKGKGKGKGKGKGKSKATAEPEQTEQENAESKFQKLRSLDVFSGCGGLSEGFHQAGISETLWAIEMWEPAAQAFRLNNPGATVFTEDCNVLLKLVMAGEKTNSLGQKLPQRGDVELLCGGPPCQGFSGMNRFNSRTYSKFKNSLVVSYLSYCDYYRPRFFLLENVRNFVSFKRSMVLKLTLRCLVRMGYQCTFGVLQAGQYGVAQTRRRAIVLAAAPGEKLPLFPEPLHVFAPRACQLSVAVDDKKFFSNITRTKSAPYRTITVRDTMSDLPEIRNGASALEISYNGEPQSWFQRQIRGTQYQPILRDHICKDMSALVAGRMRHVPLAPGSDWRDLPNIEVRLSDGTMTKRLRYTHRDKKNGYSSTGALRGVCSCAEGKPCDSADRQFNTLIPWCLPHTGNRHNHWAGLYGRLEWDGFFSTTVTNPEPMGKQGRVLHPEQHRVVSVRECARSQGFPDTYRFFGNILDKHRQVGNAVPPPLAKAIGTEMKLHMTENMKEKENAAEAVKQESMDIAE

>Cmil_XP_007883119.1

MKTNECSSGAIGNISKVKPLLNGESDQWEQATPPTPQSPNTNQSESESPADSDMEVMLNDNEEIRNGPAGRLKSKLRCQAGSDEAKRRGVSGWESSLRQRPTPRIIFQAGISFHHKKKKEDNNMKPKKELSRMPREVPAKSLVEGNSIIDLTMEPLESNTKTLNKTEELVCENKVVKGTEVVLLQYQDGKGFGLGELVWGKIKGFSWWPAIVVSWRVPGKRLAISGMRWLQWFGDGKFSEVSTDKLVPLTAFSQYFHSSAYNKLVSYKRAVYQSLEIASTRSEMTFPSNERSTLEDSIKPMLDWAFGGFQPKGYEGLKPKEKAENDAVSEKLTELYVPDFYPPSKKQKTSIYKSKEGTEDEQRGREKILNEVMSKNKSIEEFCLACGSTSVSTFHALFEGGLCAVCKDIYLETSYMYDDDGYQSYCTVCCGGREVLLCGNANCCRCFCVDCIDILVGPGSSEEAKVQDPWRCYMCLPHESYGVLRRRSDWTLKLQQFFASDNGQEYDPPKIYPAVPAEQRRPIKVLSLFDGIATGFLVLRDLGFKVERYIASEICEDSIAVGTVRHEGKITYVHDVRNISRKNIQDWGPFDMVIGGSPCNDLSIVNPARKGLYEGTGRLFFEFYRLLHETRPKEWEDRPFFWLFENVVAMGVNDKRDISRFLECNPVMIDAIDVSAAHRARYFWGNLPGMNRPLVASSADKLELQDCLEHGRIAKFSKVRTITTRSNSIKQGKDQHFPVIMNGKEDILWCTELERIFGFPVHYTDVSNMGRGARQKLLGRSWSVPVIRHLFAPLKDYFACD

>Cmil_XP_007896370.1

MEAAARGEPRPRPGPGPRVLELYSGIGGMHWALRESCIPAEVIAAIDINTVANEVYKHNFPSTHLYPKTIEGMTLDDFNRLDFDMILMSPPCQPFTRIGSQKDVLDPRTKSFLYILDILPRLSKTPKYILLENVKGFETSVARDKLIQTLESCGYNYQEFLLTPTCLGIPNSRLRYFLIAKLQPGAFCFQTVNWILEGFPHHERDDWKQSKLPSSDQTLSTGDHTEKLNNSECGHADNSKCQEMETLIYKLETSGHLERKLKQNNNLSIQMIQDFLEEKLQNPSQYLISPKVLMRYALVLDIVGLTCRRSVCFTKGYGHYAEGTGSVLQTASDVELSTVFQEFETLSENEKLAQLSKLKLRYFTPREIANLHGFPAEFNFPEKISVKQSYRLLGNSLNIHVVAKLIELLVQ

>Cmil_XP_007898625.1

MVVAPKCHSSQDNEASEQVQNGAVDRNQRDTGGSKQNEDKSNPENGDPGSAVPPDQNVKDQGFNEEGTNLDENLEIDLEMENGCHSPKDDDDSDKEESEKLSTSMRKKYKKRQLETAEKDLDDNNESAREPVKVEGTRGRLRGQSGWESSLRQRPMQRMTFQAGDPYYISKRKRDEWLAKWKKESLTSMHPPARRVNKRAAEPIDVSPLNATPLRSQEEALLPWGELERMSYFPRCAKLHSLPQTEAEKKAKLVTVMNAQEDPQTQKEEETSTIPPQQPTDPASPSIATTPEPPVISNEVAEKNNPKVTNDEAEYEDGRGFGIGELVWGKLRGFSWWPGRIVSWWMTGRSRAAEGTRWVMWFGDGKFSVVCVEKLMPLSTFSTAFHQPTYSKQPMYRKAIFEVLQVASSRAGNHFSVQAENDELDTGKAVETQTKQMIDWALEGFLPSGPKGLEPPEEERNPYKEVYTESWVEPEAAAYPSPPPTKKARRNIEKLKVKEIIDERTRERLVYDIRQNGRNIEDFCLACGSQNVLLEHPLFIGGMCQSCKGLRQVKTGNCFLECAYQYDDDGYQSYCTICCGGREVLMCGNNNCCRCFCVECVDLLVGPGAAQAAIKEDPWNCYMCNHKGIFGLLRRRDDWPSRLQLFFANNHDQEFDPPKVYQPIAAEKRKPIKVLSLFDGIATGLLVLKDLGIHVERYIASEVCEDSITVGMVRHQGKIMYVGDVRNVTRKHIKDWGPFDLVIGGSPCNDLSIVNPARKGLFEGTGRLFFEFYRLLHETRPKEGDNRPFFWLFENVVAMGVSDKRDISRFLECNPVMIDAKEVSAAHRARYFWGNLPGMNRPLVATCNDKLELQTCLEHGRTAKFNKVRTITTRSNSVKQGKDQHFPVLMNEKEDILWCTEMERVFGFPVHYTDVSNMSRLARQRLLGRSWSVPVIRHLFAPLKEYFACV

>Cpic_ENSACAP00000004041_1

SSLSPQECVKEKLNLVHGFLQADAQNQLSDLETRLHREELSEEGYLARVKALLSKELSVENGDAAALSQKSNGCSENGAYGSDEDSERAGHEGEEDSAMETEEAVASSSSSSATMSKTRKARRSKSNGENKKSPASSRVTRSSGRQPTILALFSKGSNKRKSDEVNGEVKQETNPEKEEEELEEKVRLAHVKQILILMYFVPRSEIKEEATQVKTAPPAKTTPPKCVDCRQYLDDPDLKFFQGDPDNALEEPEMLTDERLSIFDANEDGFESYEDLPQHKVTSFSVYDKKGHLCPFDTGLIERNIELYFSGAVKPIYDDNPCLDGSGGVRAKKLGPINAWWITGFDGGERALIGFTTAFADYILMDPSEEYAPTFALMQEKIYMSKIVVEFLQNNPDVSYEDLLNKIETTVPPAGLNFNRFTEDSLLRHAQFVVEQVESYDEAGDSDEPPVLITPCMRDLIKLAGVTLGKRRAARRQAIRHPTKIDKDKGPTKATTTKLVYLIFDTFFSEQIEKNEREEDKENAMKRRRCGVCEVCQQPECGKCKACQNMIKFGGSGRSKQACLQRRCPNLAVREADEDEEVDDNIPEMPSPKKMLQGRKKKQNKSRISWVGEPIKRDGKKDYYQKVCIDSETLEVGDCVSVSPDEPSKPLYLARVTALWEDSSGQMFHAHWFCQGIDTVLGKTSDSLELFLVDECEDMQLSYIHGKVNVVYKAPSENWALEGGLDMEIKMVEDDGRTYFYQMWYDQEYARFESPPKLQPSEDNKYKFCMSCARLDEVRQKEILKVMEPQEEVDGKMFYGLATKNGVQYRIGDGVFLLPEAFSFSMKLASPAKRPKKEAVDEELYPEHYRKSSEYIKGSNQDAPEPYRVGRIKEIFCSIRSNGKPNEADIKLRLYKFYRPENTHKSVKASYHSDINLLYWSDEEVTLDFKAVQGRCTVEYGEDLTECIQDYSASGSDRFYFLEAYNAKTKSFEDPPNHARRAGNKGKGKGKGKGKGKGKSVSALEQSKQEAAEVKLPKLRTLDVFSGCGGLSEGFHQAGISETLWAIEMWEPAAQAFRLNNPGTTVFTEDCNVLLKLVMSGEKTNSLGQKLPQKGDVEMLCGGPPCQGFSGMNRFNSRTYSKFKNSLVVSFLSYCDYYRPRFFLLENVRNFVSFKRSMVLKLTLRCLVRMGYQCTFGVLQAGQYGVAQTRRRAIVLAAAPGEKLPMFPEPLHVFAPRACQLSVVVDDKKFVSNITRTYSGPFRTITVRDTMSDLPEIRNGASALEISYNGEPQSWFQRQIRGSQYQPILRDHICKDMSALVAARMRHIPLAPGSDWRDLPNIEVRLSDGITTRKLRYTHHEKKNGRSSTGALRGVCSCAEGKPCDPADRQFNTLIPWCLPHTGNRHNHWAGLYGRLEWDGFFSTTVTNPEPMGKQGRVLHPEQHRVVSVRECARSQGFPDTYRLFGNVLDKHRQVGNAVPPPLAKSIGLEIKSCVLAKVKE

>Cpic_XP_005282499.1

MLGENSPVNLGGTGWENCYADCPAEAQICSSACCHSESCISAEVAAAVDVNTVANEVYKHNFPSTPLWAKTIEGITLAELNRLSFDMILMSPPCQPFTRIGLQGDVSDPRTNSFLYILDILPRLQRLPKYILLENVKGFETSSARDKLVETLKKCGFKYQEFLLSPTCLGIPNSRLRYFLIAKLQSELFSFQAPGQILENFPDQEPKASVKHRVAAGLEKESSFMPPKEEYIDPNSGSDCGGGQCSQKEAFVFKLETAKEMEKKRNQDSDLSIQMLKDFLEEDGEEMSQYFLPPKALLRYAFLLDIVTPSCRRSTCFTKGYGHYVEGTGSVLQTAEDVQLESVFKSIEMLSEEEKLMKLSTLKLRYFTPREIANLHGFSPEFGFPDKITMKQRYRLLGNSLNVHVVAKLISLLTR

>Cpic_XP_005284191.1

MPSNGSIDTSSAAPDRETLDAHKQREEELEENRSKDEKQEPGTATRKAGRPGRKRKHALVESSEASKDTASVPKCHPPCQDAGSTEQVLNGDLETDGAQWKGPEDSDGTPKGRQHGEDETDSLPDSETGQALENGRCTPKDSPDPPADEGELAPSAPQKKRGKRKLLETAGKSKEEKEEANYDSLKMEQGSRGRLRGGLGWESSLRQRPMQRLTFQAGDPYYISKRKRDEWLARWKRETDNPSASQLDKAEKKAKVIAVMNVVEESPRAEPQKEEEASPPASQQPTDPASPNVATTPEPVVADSVDKSTSKSADDEPEYEDGRGFGIGELVWGKLRGFSWWPGRIVSWWMTGRSRAAEGTRWVMWFGDGKFSVVCVEKLLPLSSFSNAFHQATYNKQPMYRKAIYEVLQVASSRAGKIFPACPENDETDTSKAVEIQNKQMIEWALGGFQPSGPKGLEPPEEERNPYKEVYTEMWVEPEAAAYPPPPPAKKPRKSTTEKPKVKEIIDERTRERLVYEVRQKCRNIEDICISCGSLNVTLEHPLFIGGMCQNCKNCFLECAYQYDDDGYQSYCTICCGGREVLMCGNNNCCRCFCVECVDLLVGPGAAQAAIKEDPWNCYMCGHKGIYGLLRRRDDWPSRLQMFFANNHDQEFDPPKVYPPVPAEKRKPIRVLSLFDGIATGLLVLKDLGIQVDRYIASEVCEDSITVGMVRHQGKIMYVGDVRNVTQKHIQEWGPFDLVIGGSPCNDLSIVNPARKGLYEGTGRLFFEFYRLLHEARPKEGDDRPFFWLFENVVAMGVSDKRDISRFLESNPVMIDAKEVSAAHRARYFWGNLPGMNRLVRAWPLASTVNDKLELQECLEHGRIAKFSKVRTITTRSNSIKQGKDQHFPVFMNEKEDILWCTEMERVFGFPVHYTDVSNMSRLARQRLLGRSWSVPVIRHLFAPLKEYFACV

>Cpic_XP_005304927.1

MSVQVAMIDLTEEDSKDSSQSSSTTSASSTQEDQNGSPELVAGEQESRVAGEVVEYQDGKDFGIGELVWGKIKGFSWWPAIIVSYRVTSKRQAISGMRWVQWFGDGKFSEVSADKLVGLMAFRQHFNHSTFNKLVSYRRAVHHALEVARSRAGKTFPGTPGESLEEQLKPMIDWALSGFKPMGCKGLKPPKTPENGVALLKNGIEEVCAPEHCPPAKRLKTNFCNNSKERVEEDQSREQMVSEVTNNNRSLEDSCLSCGRKNPATFHPLFEGGLCQTCRDRFLELFYMYDEDGYQSYCTVCCEGKELLLCSNASCCRCFCVECLEVLVGQGTSAKAKEQEPWSCYMCQPQKCYGVLQRRPDWNVRLQDFFTSDKGQEYDAPKIYPAVPATERRPIRVLSLFDGIATGYLVLKDLGIKVEKYIASEICEDSIAVGTVRHEGNITYVHDVRNITKRNIEEWGPFDLVIGGSPCNDLSIVNPARKGLYEGTGRLFFEFYHLLNYARPKAGEERPFFWMFENVVAMRVNDKRDISRFLECNPVMIDAIKVSAAHRARYFWGNLPGMNRPLVASRTDRVELQDCLEYSRIAKLRKVQTITTKSNSLRQGKSMQLPVLMNGKEDNLWCTELERIFGFPLHYTDVSNMGRGARQKLLGRSWSVPVIRHLFAPLKDYFACE

>Cpic_XP_008169122.1

MPATLSSPNRDLIAHEVNENQRSIEEICICCGSFQIHTQHPLFHGGICTPCTENFLETFFLYDDDGLQAYCTICCSGKTLLMCDDPTCNRCYCLECLDVLVSPGTAEKFKAMNTWLCFMCLPLSSNGLLKRKKRWRAKLKCFYDQESNHLEIYQPLSAWERKPINVLSLFDNITPEMKNLGFLGSSMGDGRLIYLDDVTDVLRTDVKGWGPFDFIFGSTPPVGNSYEHPPAWYFYQYHRILQYGKPPESSQRPFFWMFVDNLVLEKEDRETASRFFKMEAVTIHREHAETVQNAVILWSNIPSVKSKYSLSDLDLDLDLLAKNILRTRLFSQRPATLVRKFFLPLREYFKCFS

>Ctel_108989

MGDPLRVLELFSGIGGMHYALQETGINHEVIAAADINTVANDIYKHNFPDCLLMNRCIESIQLSEFSRLRPDLITMSPPCQPFTRVGKQRDIDDPRTKSFLHLLKVISTLERSVKYIFVENVKGFDGSEAHRMLLETLQAADYVMQEFLISPLQCGIPNSRLRYYLMAKKKPLKFQFDTTSQIMTELPACAASYLNHCQSKPDSTASSSVPLYDRCAAMCRPLSSYLQEDMSHDLLPEKFVHRFWVMDIVKPSSTNSCCFTKRYGHHIEGAGSVLQTNTDCEIDLTEYKKTKVYTAETEAAVKQLGLRFFSPREIANLMHFPAHFSFPANFSTVQTYRVLGNSLNVHVVAVLMKLMLIEK

>Ctel_160905

MVVTAVKVAPLKCKECRQLIDDPDLKLYPGDSSEAVEEFIALTDPKLQLFTGDEEDVNAADERPQHKITNFTVYDKNMHVCPFDCGLIEKNTELYFSGSLKPIYDENPSPEDGILTTKLGPINEWYTAGFDGGESALIGFTTAFAEYILMTPSDLYAPTMTVVQEKIYLSKIVIEFLSDDTEATYEDLLNKISTTVPPQGMTSGFTEDSLLRHAQWIVDQVESYDNAGEDEDILLIATPCMRALIKLAGVTLGKRRVISRQGKSRVLKKQKPTFTMATTTSLVKSIFDSIFQDQIEDKLAKVKAQRCGICEVCQAPDCGTCKNCKDMVKFGGSGRSKQACQQRRCPNMAVKDADDNDALDDVDKENVMKPEKQPKGEAKSRMLKFKKQKSQLEWEGEPVLKKSKKTFYASARLNGEILSVGDYVSVKPDDPSTPVYIAQIKYFYEDSDGTMMFHAQWYSRGSDTVLGEASDPKELFVVDECQNTQLAFCVEAVKVHYLTPADNWQMIGGDDRGMNLDDQEEGCAFFFQKWLYDEDLARFEDVPIVENVEKDVTFCSSCVRLAEKKKKESPLLGEKIDSSKSSRCSFSSVNYWGESYSVGDCVYVDPEAYGFHIKPVNHKKSKHEKPCDDELYPEAYRKGDYVKGSNLQNPDPFRIAKIQEIYVKSGVSTAVLYAQYLCRPENTHKGSHGTSHTDLNMLYWSDEDGKAALSQIWGKCQVVHWDDLQVPLEDYFAAGDDRFYFSEMYNTNTKEFEEPPSECRSQVSKGKGKGKGKGKGKGKSSEETPREPEPTEANAQPITRLRGLDVFAGCGGLSEGFHQAGIAESCWAIEKEEPAAQSFRLNNPGATVFTDDCNLLLKAVMEGQTVNDVGQKLPQKGEVELLCGGPPCQGFSGMNRFNSREYSMFKNSLIASYLSYCDYYRPKFFLLENVRNFVSFKRSMVLKLALRCLLRMGYQCTFGVLQAGNYGVPQTRRRAIILAAAPGEKLPFYPEPLHVFAPRGMQLSVQVGDKRYNSCVQRMSSAPFRTISVRDAMSDLPEVKNGAKAEKISYSVEAQSHFQRMIRGHQHQPVCRDHICKDFNPLVHARMQHIPLYPGADWRDLPNIEVRLSDGSKCKKLCYTHRDKKNGRSPSGALRGVCQCADSGSCDAMDRQFNTLIPWCLPHTGNRHNHWAGLYGRLEWDGFFSTTVTNPEPMGKQGRVLHPEQHRVVSVRECARSQGFPDTYRFFGNIQDRHRQVGNAVPPPMAKAIGSEIKKCVLWKMEQDAKKEREKKKQVDKEEGKKQDEKEKKDGDEEMKGDEEERKGDEEERKGDEEEMKGDEEERKGDEEEERMES

>Ctel_188129

MTLFRSKDRGRKRKLPEIKHYSLRERKVKKKEKREIEEKQEERKDVKKIKKEKKIRESPKKFKAMQSLITKETRIVGALIWCRFGRRMWPGMLIEGSLVGRPPSAVNTEWVFWFGEGTVSQMDASAMRPFIASFRDHYDPKYLSKSYERSIQEAVRVCAQRAGKCRSSASDLLEWAERGFRGSASGPLHDDKSIPEWISARLEKLKKWAQDISTDDDDDEEEEDEDGGDEEEEEEEEEEEADQRPLRSETYEKEKEIWDRVRDGSFSVTDVCISCGGTNEVTHCHPFFIGGHCASCKQDFEESLFLYGSDNISMPKDWNVKRPLRVLSLFDGIGTGKLVLDLLGFEVEAYFASEVNPDCLRLTEVRHPAIKQLGDVTMLTAEKLVEIAPIDLVIGGSPCQDFSIVNPLRKGLMGRVSVVIPWLYHGLLFFDFYRILNELQSLQQANRPLFFLYENVASMNKETKAVMTRFFKRQPNVWDAINFTAQRRKRYFWGNIPGLKGGECVDDEDVTELKEVLMPGRKARMSKVRCVTSRSNSLRQSKHNHKQFPVAMGNHLDTLWIPELERLFGFPQHYTDVANIPVSRRQVLLGNSWSVPVVKRLLLPLKNFFQTKH

>Ctel_222429

MQEHSEWLAEQVISYDEGRDEDDLQLALCPAFRTFLRVTGITPKSKKLGKKTKPRQSLVIVKKEKMVATTTAAVRQLFSKDFKSLFGAATMSGDLEVIISDDEGEEDGDAPPRSAQLSRNPRGHKNRMLAGEVEWGPLLREDHCSSHYESATVDNVKISPGDYVQIEHDDSAKEPHLGRVISLFERQDSPEPQVHIHWLHYTSTTLLRETGKDNELYFSYDECQDVDLSSVMCRVNVTEVSQLTVNDSHVHYGMSDERLYCVEYVYNDTTGAFTSPAKPKKFTCPSCAVLKARRLKESPSISDGILQYWQNEYSIGCYMFVQTENKENSATLQDKEKVVANSKFPEQWRRQNDSGEPPRVAPLTIGRLLGIHWADDDDAHEVVLTLQRFFRPEETSLEKPWLKDIRLVFPSDQIFEARLDTTWGRCNVINAREYLDSLDEFLKESDNNFYYEKSYLHAEGLFIDFELDAKLLLRRKPIKREMRSKRSTRKRAKLMRFEDNPISIDDDDLIVVMSSDEEELASCQERSISWSSSDSSSDSSSYSVPSPIVSLPNVEAKLRSLDLFSGCGGLSLGLSQAGVSEACWAVERDPVIAEAFKRNFPKCCVYAADCNAMLGSILAGESCDPSGQVYPLKGEVDLISAGPPCQGYSLLNKFTSSEAYQFKNSLVVTALSFCDYYQPKYFVMENVSTFATHKGGNLLKLVIRCLLELGYQCRFGILQAGSYGVPQSRRRFILMAAAQGLLLPAFPEPIHAFPVAFCDVYIDKRKFKPGLERIKIGHLRPLQIRDAFDDLPDVAANSTTAVHIKPTAKSLPWSSDSSVVFDHCVRPVDALNAARFAHIPRVRGADWRDLPNISVPLSDGSQTNILKYPFKGQIEGCVRKGVCACVEGKKCKSERQKNTLIPWWLPHTAQSNNHWKGVYGRLDERGCFRTIITNPQPGVKQGRVLHPSKDRIASVRECARGQGFPDDYIFVGTIEKRYIQVGNAVPPPLAKAIGIEIRKSLISSQYR

>Dmel_NP_001036355.1

MVFRVLELFSGIGGMHYAFNYAQLDGQIVAALDVNTVANAVYAHNYGSNLVKTRNIQSLSVKEVTKLQANMLLMSPPCQPHTRQGLQRDTEDKRSDALTHLCGLIPECQELEYILMENVKGFESSQARNQFIESLERSGFHWREFILTPTQFNVPNTRYRYYCIARKGADFPFAGGKIWEEMPGAIAQNQGLSQIAEIVEENVSPDFLVPDDVLTKRVLVMDIIHPAQSRSMCFTKGYTHYTEGTGSAYTPLSEDESHRIFELVKEIDTSNQDASKSEKILQQRLDLLHQVRLRYFTPREVARLMSFPENFEFPPETTNRQKYRLLGNSINVKVVGELIKLLTIK

>Dple_DPOGS212925

MVHKILELYSGIGGMHCAWNASELNGEVVTAVDINTVANEVYKHNFPNTPLITKNIQSLSYEDIKKMNVNTILMSPPCQPFTRNGKFLDENDPRTNSFTYLIDLFDELDNIEYILMENVKGFECSTVRNLFINKLKKCNFVYQEFLLCPTSVGVPNSRLRYYCIARKNTLDWSFIRTDEIITKLTKDYGEPHTLEAILETNVPEKYLLTNNFLKRAYLLDICYKHSKRSCCFTKSYTHYVEGTGSVYTDSTPDEVENCIKDAKQYEVGKEEYVDRFQQLKLRFFTPKEVLALMMFPKSYKFPERTTTKQCYRLLGNSVNVKVISELLKILFE

>Dple_EHJ76342

MDNATHSTTRKSDRKASTRDGQLKITSMFAKKRSRSPIESAEKDDTKKLKINTESHEHEVFNEKSKVVNGINSKSDDVNSQESEETSPNLVSMKLNNERSLVDEDENHNIEAKTVPEIINTMNGCNQNGDEMTRDGLENQVQQNAIVEQDPPEPKPTAKIPDQHGHLCPIDGGLIESDVRIYMSGYLKSICSDSPDIDEESIAVKDVGPIIEWFIHGFDGGSRNCITLSTEFGEYNLLKPSAEYTPLMDNLYEKIWLSKVVVEYLEEYHYLQPTYEDLLEVIREHSIPDLEDKRMTEEMLHKHAQFVCDQVVSLEADEDNEPLITLPCMRELIKLMGIKFGKRKVRAKIDYKKIDKKAWTKATTTPLVQKTFEHFFANQLDKTNHELVLRRKRCGVCEACQLPDCGECNACRAMLKFGGHGRTKKACVRRLCPNMAVQQAEDSEIEDEEEYQQMAEKRHLDKIDDALPVKLTGGSNKIIRWIGDPVKADATKVYYEKVEIDGSELSLGDFVMVETSQSNIPALVARVTYMWKESINPKSGYFHAEVFIRSSDTVLGEVGDPREVFLGDRCCHGAPLSSILRKAFVEKKETPADWFKLGGKEVVDHFFEDDGKTYFYQKYYERFTARFEDLPNDPECPNALRKHRFCPSCERKTRRDARDIPKISGKLTEKSEIVKEANRFEWTTIRWRDHDYKKGCGVFLKPGTFRFKNSMINSSNGINRVKLDKVDEDIYPEYYRKTDNYLRGSNIDTGEPFCVGYIAAVTAASEGPLVIPQDIYIKVNVMYRPENTNNRFPHHEDVNVVYWSDEIKEISFSAVVGPCNICYVDNIPQQDHIYDWLEKDPSRVYFRMAFNKKSGQVEDVPQHVKYVGRGDKGKDKGKGKGKSSKGAQSTVTVKVDEVKVRPLRTLDVFAGCGGLSEGLHRSGVAECRWAVENLEAAAHAYSINNKNCIVFNEDCNALLKDAMDGATHSAGGLRIPMQGEVELLCGGPPCQGFSGMNRFNSREYSNFKNSLVASYLSFCDFYRPKYFILENVRNFVAFKKGMVLKLTLRALLDMGYQCTFGILQAGNYGVPQTRRRLIILAAAPGYKLPLYPEPTHVFSRRACSLTTTIDGKRFVTNIQWDESAPRRTCTIQDAMSDLPQICNGANRIEIDYGCMPETYFQRLIRSRDESAKLRDHICKNMAPLIQARMSRIPTTPGSDWRDLPNISVALSDGTKCKVLQYRYDDIKNGRSTSGALRGVCACSAGGVCSVADKQENTLIPWCLPHTANRHNNWAGLYGRISWDGYFSTTVTDPEPMGKQGRVLHPEQNRVVSVRECARSQGFPDTYLFAGSIQDKHRQVGNAVPPPLGAALGREIKKALSALS

>Dpul_117816

MAPLFEARIALIPSKPGSDWLDLPNTEVRLKDGVLTDKNGRSLSGAMRGVFSCAVGRQCDPLDKQYNAHIPWCLPHTGNRHNWAGLYGRLEWDGFFSKTITNFESMGEQCTRVCSLARFTDSYRFFGNITDKHRQVGNAVPPSLARAIGLEIHKCVYEGDC

>Dpul_192688

MEKKQMRILELYSGIGGMHYAAELANVGAEVVFSVDINTSANAVYRHNFKQTNQQARNIESLSAKEINKLRPDIIMMSPPCQPFTRVGLKLDVEDSRCSSFLHLLDILPHLETVSFILMENVVGFETSEMRNAFTKALKNCDFHFREFILSPESIKIPNSRSRYYLVAKKCTDFSFGSENDIMTSFPNSRLCDIEMPVQEKTLDPYLVKDMSDEELARYLLTDKTLFKYWRILDVRQTSDTSSCCFTKAYTHYAEGTGSVLQHDPNEPFHQKFAEFKEDEDIAHLKPLKLRYFTPREVGNLMGFPAEFTFPENTSLKTRYRLLGNSLNVLVVSNLLRILLG

>Dpul_320126

MNSDSDDCYDSDATYFCGSDDDDGDGNFGVEADGLLLEKWIPPLFYLAKGHLQTIHNDVNKLENVCLACWEKGTCPHPFFVGALCQQCKQRLLRTMFSKTVDGFHLYCTICGSKSNACVSCSNGKCYRKYCVNCLNIWTDHSDKLIKNSDKWFCFLCLPEPNQILQAHHNWAQKVLLFHEPLAVYASRALYWKRQPLRVLSLFDGIGTGLVALRKLGIEVEVYYASEVLTAAATVSRTRLGGVLHHIGSVGEVTQQRLEEISPIHLLIGGSPCNDFSAINRFPKDFYDPRGYSRYFFDFVRVLNLMRKINGQHQHLLWLFENVASMPQHYRETISRHLDCQPAVIDAKNFSPQLRRRLFWGNIPGLFTVHEQQLTQDGESLSLEKSLMPNSGRRAAQEKIRTLTTNTNSLLQGRTENCDEDDNLQTDVLWLQEIEHVFGLPRHFTDVGNMSRTDRQKLLGHAWSVPVIASIFSNLKAYTV

>Dpul_EFX80183.1

MRIVRTDGDEAAGNEVKVKEEPTELQSNNESSPDTTPVEEEAKPILKPVEEEANKPTSKPVTVLSKTRGVKVRYTFDEAAASGEGDDDFLPGNTKKKRRTASRHAKVELPDIGGYRPSRKKKINRKDPNRCHVCRQRFDSPDLKYFPGPPADSIEEIIALTDPSLSLFTGEEEVMSELDQRPILKLTQFGIYDEPGHLCHLDGGAIEANHLLFAYGYVKPVWNDNPGVEGGIATKEIGPINEWFISGFDGGEKAIMCFATSYADYYLMSASELYEPIMNELEEKIFLSKTVIELLIEDDNAEYEDLLTRLQNSLMPNGSRVSEDSLLRYAEFVCDRVYHFDAAGAEDEPPLILSPCMRTLIQLSGITLGKRRATRKLEKRRQPKAKKGPNWTKATTTPLVGYVFESFFRDQMEQGEDKFGPTSAPRRTRCGICEACQQTDCGKCNFCRDMVKFGGSGRSKQSCVLRKCPNRAVQVADDDDELDAVLDTEIEVVSLDVDHKKPVKRHAYKIEWDGPELKEVDDLTFYSAAIVNGDMRVFAGGHVTIEPDDPSIPMYIAEVIALWEDGKSGEQFLHARWFCRGTDTVLGETCDDPRELVLIEDCEDLLLSAVVKVVNVKYKPVDPVKWKAEGGSDDPSLFQSEEDHSDTTFWYRYLYHGRTGRFEDPPECPDVVNNIKGCPCCDRLDVIRQRDFAKLGNKLDSGGFDSVTWHELDVKVGEAVFLEPGAYAMRGPDGSIVKKEKIDPEEDDIFGVDYDDEYYPEKYRKTDNIKGSNNDTPDPFCIGYVVGIAYNGILHNNLNAREVCLKVKRIYRPADTHLGRDAGFRSDWNLVYWSDEIHKLELNKVVDKCVLVCSTAIDEPIEEFVRGGPNRMYFNKAYNPAEREFEPPPVEAERIGSSSKGKGGKSLKSAKSIQPTYPSYPKIEPLKTLDIFAGCGGLSEGLHQSGVAKTYWAIECEPTAAQAFRLNNPDAAVFTDDCNTILKMAIDGQLEQNGQVLPPKDGVELLCGGPPCQGFSGMNRFNSRQYSSFRNSLIVSYLSYCDYYRPRFFILENVRNFVSFKRNMVLKLTMRCLVRMGYQCTFGVLQAGNYGVSQTRRRAFIFAAAPGEKLPLYPEPTHVFSRRGCQLSVAVGRDKFYSNCRWLLSAPYRTVTVRDAMSDLPEIPNGAKQEEISYGGDPQSHFQRWMRGTDSEQSSVLRDHISKEMAPLVEARIAFIPSKPGSDWRDLPNTEVRLKDGVMTVKLRYTHEDKNGRSLSGAMRGVCSCAEGRQCDPLDKQHNTLIPWCLPHTGNRHNNWAGLYGRLEWDGFFSTTITNPEPMGKQGRVLHPEQHRVVSVRECARSQGFPDSYRFFGNITDKHRQVGNAVPPPLARAIGLEIHKCVYEGDCKKKKDEPPTIVQC

>Drer_AAI24099.1

MRKEEIKKSSEIVMPSNKYPSAESDKMTATAAMNRDTSVGDGLSENDSGLEMTSENSPLTPAEPPSPFCPKQNGGAASPADESVNSIRRKRSRKRSDTEEDSAWDSSNSEEKAEVGSGCETGLRQRPRPRTIFQAGLTAHSKPRSRERGHSKEDHSDLVASVPEGPALELMEQDSKDSAQSSTTSTSTTETASQPEYKDNKGFGIGELVWGKIKGFSWWPGMVVTWRATGRRQASHGMRWLQWFGDGKFSEVSADKLDSITAFPKFFNQSSYTKLASYRRAIFQALEVASLRAEKTFPPSEADSLEEQVKPMLDWAHGGFLPKGQEGLKPKENAEYCVFPLASESSTLLESSPPEFPPSAKRARLPLNKAKPGIEEVYSREQMVNEVLKNHRSIEEFCLSCGKTRVATFHPLFEGGLCLTCKDAYLENSYMYDDDGYQSYCTVCCGGREMLLCGNANCCRCICVDCLDILVGAGAANSARNLDPWRCYMCQPLQQYGVLKKRHDWSLKLQEFFVNDSGQEFESPKIYPAVPAEQRRPIRVLSLFDGIATGYLVLRDLGFKVDLYIASEVCEDSISVGAVRHEGKIQYVHDVRNITRKNIAEWGPFDMVIGGSPCNDLSIVNPARKGLYEGTGRLFFEFYRLLSEAKPKEGEDRPFFWMFENVVAMSVNDKRDISRFLECNPVMIDAIEVSAAHRARYFWGNLPGMKRPLCASGMDKLELQDCLEHGRVAKFGKVRTITTRSNSIKQGKDQHFPVMMNGKEDILWCTELERIFGFPVHYTDVSNMGRGARQKLLGRSWSVPVIRHLFAPLKDYFACE

>Drer_ENSDARP00000013243

MPTKTSLSLPEDVKERLQVLDEGGDSLSDEECVKEKLRLLQEFLLADTQDQLKNLEDKLKSSELSTEVYMSEVKAVLKKALGVVKEGDGVEQNGHSNGFSENGSHKDNGEQEGAMDTQEEGDAIKSPSAPKGRGGRRSKADSEPKKSPASSRVTRNTGKQQTIVSMFSRVPKRKSDELNGEPANGDTEIKTEETITEEVREEKRLKTEDEKPEAENAANLKPVSTAKTPPPKCPDCRQYLDDSDLKFFQGDPDDALDEPEMLTDERLSLFDSNEDGFESYEDLPQHKITNFSVYDKRGHLCPFDSGLIEKNVELYFSCAVKPIYDDNPCMDGGVPAKKLGPINAWWITGFDGGEKALIGFTTAFADYILMDPSEEYSAIFALMQEKIYMSKIVVEFLQKNQDATYEDLLNKIETTVPPAGLNFNRFTEDTLLRHAQFVVEQVESYDEAGDSDEQPIIITPCMRDLIKLAGVTLGKRRAARRQAVRHPTKIEKDNKGPTKATTTKLVYLIFDTFFSDQIDQNNKDGGVKRQRCGVCEVCQAPDCGKCSACKDMIKFGGSGRSKQACQKRRCPNLAVKEAEDDENMDEEDVLPVKDTKKMSQTKKKKQTKNKISWVGEPLKTEGKKEYYMKVRVENEVLEVGDCVSVSPDDPSHPLYLARITALWDDGEKMFHAHWFCRGTDTVLGESSDPLELFLVDECEDMQLSFIHGKVNVFYKAPSENWYMEGGMDEDIKVIDDDGESFFYQLHYEGECARFETPPKVTPSEDCKYKFCASCTRNKEREAESVPHAYEPLEDEESDSKVFYGLVNYKGEQYKVGDSVYLPPEAFNFVVKAASPVKRSHRKDDVDEDLYPEYYRKSSDYIKGSNLDAPQPFRIGRIKEIFCNKRSNGKPDTSEIKLRLYKFYRPENTHKGPKGAYHSDINQLYWSDEEATVSMTEVLTRCRVEYAEDLVESVQDYSNKGPDRFYFLEAYNAKTKSFEDPPNHARSAVNKGKGKGKGKGKGKGKAAPQEPQDQEPQEPVVPKLRTLDVFSGCGGLSEGFHQAGISETHWAIEMWDPAAQAFRLNNPGTTVFTEDCNVLLKLVMSGEKTNSLGQKLPQKGDVEMLCGGPPCQGFSGMNRFNSRTYSKFKNSLVVSYLSYCDYYRPKFFLLENVRNFVSFKRSMVLKLTLRCLVRMGYQCTFGVLQAGQYGVAQTRRRAIILAAAPGEKLPRYPEPLHVFAPRACSLSVAVDEKKYVSNVTRGNGGIYRTITVRDTMSDLPEIRNGAAALEISYNGEPQSWFQRQIRGSQYQPILRDHICKDMSALVAARMRNIPLAPGSDWRDLPNFEVRLRDGTTTKKLRYTHPDKKNGRSGTGALRGVCSCSEGKPCDPADRQFNTLIPWCLPHTGNRHNHWAGLYGRLEWDGFFSTTVTNPEPMGKQGRVLHPEQHRVVSVRECARSQGFPDTYRFFGNVLDKHRQVGNAVPPPLSKAIGLEVKKCVLEKMRENATEPVKQEKMELSD

>Drer_NP_001018153.1

MENTERLRVFELYSGIGGMHYALKESLVPAEVVAAVDVNTTANLIYKHNFPTTQLLPKTIEGMTLQDFDRLNFDMILMSPPCQPFTRIGLQGDVADPRTKSFLYILDLLPRLSKRPRFILLENVKGFESSAARDALLQTLRECDYSFQEFLISPTSLGIPNSRLRYFLIAKRAPETFSFPVSTEIIEGFPMSESTDGLSVPYDHPTSSASEREKTIMFKLETEEDLERKRKQNSQESVRRLLDFLQEEEEDMEPYLLPPKTLLRYALVMDIVQPSSRRSVCFTKGYGHYVEGTGSVLQSCMDVDLETVFKNLDQLSEEDKLKQLLRLKLRYFTPREISRLMGFPDHFTFPKHISFKQQYRVLGNSLNVHVVSHLIRLMLSK

>Drer_NP_001315096.1

MPSNTVATAAAAFKSSDFNCDTNGNQMNESSMETMIIDEMPSLCTPVRKVGRPGRKRKHLHVTCYGPLNQLMAKQHKEAELQSSLQSQNGEVERHENKSIEGRHSTLDKRDVENGLFCQSGCKAESTSDSSLSPSLENGFVSSQDSEKCDEETLPTLPRKKRGRRKLERPTKYVEHKEDESGDPSKNEGARGRLRGGVGWEVSLRQRPMPRVTFQAGDPYYISRRTREELLAKWKLEAEKKAKLVSVMNSMEDHGEVESEPKKDVITVPLLFPPPMPPTPPTPPMQQQPPQPQAQHQTNLKPQPSPQQQPPPFQQQQQQQPTDPASPTVATTPEPVSIGDGDKTSPKSTESEYEDGRGFGINELVWGKLRGYSWWPGRVVSWRMTRRSPAAEGTRWVTWFGDGKFSVVCVEKLLPLSSFHNAFHQPTYNKQPMYKKAIFEVLQVASTRAGKTFLSCPASDDTDSSKLVDVENKKMIEWAMSGFQPTGPKGLDPPESEPYEMCEWVEPEAASYLPPPAKKPRKSTVTEKPKVKEMIDERTRERLVFDVRQKCRNIEDICISCGSLYVSLEHPLFIGGMCQSCTNCFLECAYQYDDDGYQSYCTICCGGREVLMCGNNNCCRCFCVECVDLLVGPGAAQAAIKEDPWNCYMCGLKTQYGLLERRADWPCRLQHFFANNHDQEFEPPRLYPPVLAENRKPIRVLSLFDGIATGLLVLRELGIQVERYVASEVCEDSITVGIVRHQGRIMYVGDVRQLTRKNIQEWGPFDLVIGGSPCNDLSIVNPARKGLYEGTGRLFFEFYRLLHEARPKEGDTRPFFWLFENVVAMGVSDKRDISRFLECNPVMIDAKEVSAAHRARYFWGNLPGMNRPMSAMCTDKLDLQDCLEHGRTAKFGKVRTITTRSNSIKQGKDQHFPVFMNDKEDILWCTEMERVFGFPVHYTDVSNMSRLARQRLLGRSWSVPVIRHLFAPLKEYFACVGT

>Drer_NP_571461.1

MVADVKIGDDKQSLCELLDWLNGLLQATFSQVEDTCSGAAFCQLMDIIQPGSIDVTKVNFTAEENLDILNNYNLLQEAFSKAQIQKELELTLLVNGDIMTTCDLLTWFKDMYDHNFAKQKCNPQVAFIKPEVVSLKSSREFETIEKENVSSLYNTEETSSNQKTQHVEKTSQESVSWSPLTSFIRKYGSSTLTDDESNNVNSKDCPGQKSFGDITPFWRQTPYCLYLLHGVELEDDKKASVLLLGFFDKETGENKIRLLDVVYPTKESTEDICNYILDTLRKIGIPLFNMAILYSDFPDHEHLVAGLQLMKAEIVSLCGLTDLTGQVCHSGVEKIEFSDLILNLITEIYKHFPSFPADLQALLEDVEGSDIDNLTSQCSLFWRIIKKIPLAWSHLEKYFGSLGTEEEAVCLLLEDPKIKLNVLFLTHALQPLCDFQEIIDQGASVLQLLQDASKLLRLYTQSFLRPKAAEYFHRRGKTSLVQETVGHLPKGEVAVGEQAADFLQQHSEELFDYLETFHSSIISFYTTVTVNIVKRLPLPDSTLRNLSLVLSPGKKLEVTGKMVQDLGVGFGVCIRPDNVSLLTDEFLEYQLIEGGDTGSVDQPTEKYWQTELRIMGNASNFGKLIVSLLALPKTLKKEIIFKQMFQQTDYLKMMRKEDCEEKDMMEDDVTDSSSYKSAPSHLSPETQGSSISDVIDLTEMDEIGPVEIEDIAPMDVDDIVSISSDSETENQKVNVPHVSIVLDDDDDDDDEMTDDDDDYGCEAGEVMWKYSKNKGNTQNEMTDNTYQGGFSVGEMVLGPIEGFGLWPGLVQSWDSERPCGSMRKVIFFGNGMQTEVQADSLLPFSSLAKCFCSNSFATVMAYKDAIFSSLQVASRRSRMFFSPESESKDELLRVMLNWAFGGFEPLGADGLQPQAEYSVKVKKGKRKNPTGKLFNLTVPLNKIPESLDLNNGSVDLGTTDADKKRLYSKWNGRSMQTVKIRRKYKQRNKNIIPTVQIESRQNSQKRHQMVHEFLKNKRKIEDFCLSCGSMSVDIIHPLFEGKLCTNCKFNFTETLYRYDEDGYQSYCTVCCSGMEVILCGHDSCCRSFCVDCLDILVCQGTFDQLKNVDPWTCYLCAPETSSGALKPRHDWSIRVQEFFANDTGMEFEPHRVYPSIPAIQRRPIRVLSLFDGIATGYLVLRDLGFKVEKYVASEIDEESITISMVNHDGKITQVDDVKNITKKHIEQWGPFDLLIGGSPCNDLSIVNPARKGLYEGTGRLFFEYYRLLNVLKPKEDDPQPFFWLFENVTFMQTHVKADICRFLECNPVLVDAVKVSPAHRARYFWGNIPGMNRPIIASQNDKLCLQECLEPGRTAKYEKVRTITTRQNSLKQGTNDAHFPVTMNGKDDHIWITELEKIFGFPKHYTDVKSMGRPQRQRVLGKSWSVPVIRHLLAPLKDYFACDEFPVK

>Drer_XP_005160915.1

MAKQSPKQEAEQEVCEVSKEEKVTDTPTRRVGRPARKRKAPAVNSCEVVTKSSGCVKQSECNGEADSRVAKKSLQRNDSSPESSHQNGADTPPGEDSAPSTPRKKRGRRKLERTERDIEEDDTSSDTSRGESDGARHRGRQGWDISLRRRPVQRETFQAGDPYHISRREKEELLAHWKKEKEKKVRLVQMSDTMDHQSAVGSQEDEPAMILPTIPIGGSRDKAGKKFEKKVRLVQMSDTMDHQSAVGSQEDEPAMILPTIPIGGSRDKAGKKFEKKVRLVQMSDTMDHQSAVGSQEDEPAMILPTIPQQQQKKVRLVLMSDTMDDQSAVGSQEDEPAMILPAIPQQQHTDPASPTVATTPEPPPVACGEKVLTGIAPIHQEYQDGLGYGIGELVWGKLRGFSWWPGRIVSCWITNRGPAAEGTRWVMWFGDGKLSMMCVEKLLPLSAFSKTFHQPTYNKQPVYRKAIFQVLLTGGLRAGKPFPHCEYTNYTPEGKMEENETQAQQMIKWASTGFLPSGPKGLEPPPAERNPYTEVYPEMWEPEAASYTAPPPAKKPRKNSVEKTKIKEIIDEGTRERLMLEVKQKCRNIEDICISCGSLNVTLEHPLFVGAMCQSCKNSFLECAYQYDEDGYQSFCTICCGGRQVLMCGNNNCCRCYCVECVDLLVGTGCAQAAISEDPWNCYMCCSRSVFGLLRRRDDWTSRLQLFFANNHDQDFEPPALYSPVAPEKRQPIRVLSLFDGIATGLLVLKDLGIQVDRYVASEVCEDSITVGMVRHFERITYVGDIRNITRKHIQEWGPFDLVIGGSPCNDLSIVNPARKGLFEGTGRLFFEFYRLLHEARPKEGDDRPFFWLFENVVAMGVSDKKDISRFLECNPVMIDAKEVSAAHRARYFWGNLPGMNRPLTAMVNDKLDLQDCLEHGRTAKFNKVRTITTRSNSIKQGKDQHYPVYMNNKEDILWCTEMERVFGFPVHYTDVSNMSRLARQRLLGRSWSVPVIRHLFAPLKEYFAC

>Drer_XP_005162096.1

MPRRRSSRLRCPKQQIKNGKQNSNNAFTCNPATMCFIKRYASSTSDCGSVSKNPALDQKYWEDITAICRQTPYCLYLYHGVEIENDKKASVLLLGFFDEDTGKNKIRLLDVVYPTKESTEDICNYILDLLRKIGIPISNMAILYSDFPDHEHLVAGLQLMKAEIVSLCGLTDLTGQVCHSGVEKIEFSDLILNLIIEIYKHFPSFPADLQALLEDVEGSDIDNLTSQCSLFWRIIKKISLAWSHLEKYFGSLDTEEEAVCLLLEDPKIKLNVLFLTHALQPLCDFQEIIDQGASVLQLLQDASKLLRLYTQSFLRPKAAEYFHRRGKTSLVQETVGHLPRGEVAVGEQAADFLQQHSEELSDYLETFHRSIISFYTTVTVNIVKRLPLPDSTLRNLSLVLSPGKKLEVTGKMVQDLGIGFGVCSENVSLLKDEFLEYQFIDESDAQPADQSMKQYWKTELRIMGKASSFGKVILSLLALPTTLKKEQNFERLFQHTDNIKRKKLKDCKKHVSEDDVTDSSSYKSAPSHLSPEMLDVIDLGEEEDVEPMEVEDTLPVSDLDTESQNDTKGNCPNVSSNDDDDDDDKCSSSHDDYSSSDDDGDVIWREKGKRNSCHNNEQTKNPYKGSYIVGEMVWGPLEGFGLWPGLVQSWDSRKPCGSMRKVFFFGNSMQSEIQADDLTPFFSFAKCFSSNSFATVATYKDAIFNSLQVASRRSRMFFSPDTDSMDELLRVMLNWAFGGFKPSGEDGLRPQSECNELVFTPRSVNGNDGPSSNFKDHREKLFNLTVSLNKLPESLDLHRGSIDIGKTHVYRKFMLPKRSLRPLQSREKKYTHWKRYLNDMESMKVDSRQNSQERYEMVHAFLENKMDIEEFCLSCGSTFVDIIHPLFEGSLCVKCKFNFTETLYRYDEDGYQSYCTVCCSGMEVILCGHDSCCRCYCVDCLDILVGKGTFKQLKNVDPWTCYLCAPETSSGALKPRHDWSIRVQELFANNSAMQFEPHRVYPSIPADQRRPVRVLSLFDGIATGYLVLRDLGFKVEKYVASEVDEESVTISMVNHEGKITYVDDVKKITRKHIDKWGPFDLLIGGSPCNDLCTVNPGRKGLFEGSGRLFFEYYRLLNVLKPKEDDPRPFFWLFENVVLMENRVRADISRFLECNPVLIDAVKVSPAHRARYFWGNIPGMNRPIIASQKDKVSLQDCLDGGRTAKYEKIRTITTRPNILRPGIDDRHYPIVMNGKDDSLWITEIEKIFGFPKHYTDVKNLGRMQRQRVLGKSWSVPVIRHLLAPLKDYFACDEFPAK

>Drer_XP_005167049.1

MATNVSLEPNNPDDKCSRYEVLGWINETLQTNFTQVEQCRSGACFCQLIDLLFPGTINLKKVKFESQKRSDFMQNYGLLQAAFRDLEVTEPVPVNELLSGKFRPNFTYLKWFKKFFYANVKQERVYNAFEARDGQEIVPVDDVMKSPKALKSSYESGRAGEESDMEINGGRRSATYDPKWQRNLKWIRASDMGDNYAYCTTCDYNIILLAGFHDLKRHQLTQNHMKHETGRTNLPGRKQIEESISCSETMLLFIQSHCLSSLPSRINRVSQRTARCILGLKYPNDIVSACKLNPYCIYIYGQVPLDVKTGDKTNCHVVLAGFFEEKQARYCIRFLDVFQPEDSASSVSGGLFSILKKFEIPASNMVAVYINDHELTSESVVSQIRELNPQVIDLGGLYSIPDTACSAGLQTHSVQVQELIANIYRHFSTGSTSNDNLKMLFAGIDGLKVHSNPLSNSEEFCVLVKRIHEMWSDLVSYFSSCDENNDNVKQICSQLENPKIRLTLMFLDQALGPLRAFGQHLQQSKSSVRADLVEILREASGLLRSYASSFLRPQAVIRYLKEQDPAILDNEAFCLPAAELSLGGVLEDFISAREEELADFLSTFYNECLAIYKTLTTSIAASLPLSDSVLRAISQLLSPAGRLKVTGKNIVDLAVRFGFCSKPEDSAKLNDEFLEYQLAEEENLSSTHSIERYWCTVLKTFPPTSVFKRLVLCLLVLPSPSLDATKIFAQAIENGDADQLDDSSSESDTDMTKELDSNDDNSLDNSELQISPIKNGIMKKSRRSTSETVQHSNAVKPCVVRLEKITSQREVNLKNDGAKNDGTLTSNTLKEDSIRGIHGWECSLRQKPQARTVFQAGAGTWSKPAIQDVDRKPEAVKNDLSNSTPSPRRGKRDQAYNDGKGFAVGELVWGKVKDFSLWPGLVVPWKGRIVPVSMRRVEWFGDGMFSEIHTDGLLPFGAFSKNFCSKSYEGLPTYKNAIYQILELAAERSGKLFPPSEKKGEEVKAMMDWAFGGFQPMGADGFLPSADSSASNKTESDSSVSDYQPPAKRKYVFKNRPSTQECNRDQMVQEVTSKGRKIEDFCLSCGSSNTEIFHPLFKGSLCIKCKENFTETLYRYDDDGYQSYCTVCCAGLEVILCGNASCCRCFCKDCLNVLVGPGTFDKLKEVDPWSCYVCLPSKCYGVLKLRTDWSVRVQEFFANNSAFEFEPHRVYPSIPAHKRRPIRVLSLFDGIATGYLVLKDLGFKLERYIASEICEDSIAVGMVKHEGKIEYVKDVRTITRKHLAEWGPFDLLIGGSPCNDLSMVNPARKGLFEGTGRLFFEYYRMLTMMRPKEDDDRPFFWLFENVVAMSAHDKADICRFLECNPVMIDAVKVSPAHRARYFWGNLPGMNRPVATSLTDNVDLQDCLESGRTAMFSKVRTITTKSNSIKQGKTGPLPVTMNGKEDYLWCTEMEKIFGFPKHYTDVNNMGRGQRQKVLGRSWSVPVIRHLFAPLKDYFACE

>Ggal_BAD99025.1

MAALRVLELYSGIGGMHQALKESCICAEVVAAVDVNTLANEVYKHNFPSTPLWAKTIEGITLKEFDRLSFDMILMSPPCQPFTRIGLQGDVSDPRTKSFLYILDILPRLQKCPKYLLLENVKGFESSSARNELLRTLETCGFKYQEFLLSPTCLGIPNSRLRYFLIAKLHQEPFFFHVPGQILTRLPDQNLEELSKDKVVDKTGSFPSGEKSLDSNTSPDCSSKKGPLPKGAFLFKLETAEQMVRKHRQDNDPSIQMLEDFLEEENEEMSQYFLAPKSLVRYAFLLDIVKPTCRRSTCFTKGYGHYVEGTGSVLQTAVDVELESVFKHIENLTEEEKLMKLSTLKLRYFTPREIANLHGFPPEFGFPDKVSIKQCYRLLGNSLNVRVVAKLISILLG

>Ggal_NP_001020003.1

MVESSDTPKDTAAVPKCPPPCPEASPAEPLPNGDLEADGAQWKGTEEGGASPKSGRPEEDETESLADGETGRALENGRCTPKEGLDAPADEGELAPSDPQKKRGRRKLLEATEKSKEEKEENNFDSLKMEGSRGRLRGGLGWESSLRQRPMQRHTFQAGDPYYISKRKRDEWLARWKREAEKKAKVIAVMNVVEETPRAEPQKEEEASPPASQQPTDPASPNVATTPEPVVADAVDKNTSKSADDEPEYEDGRGLGIGELVWGKLRGFSWWPGRIVSWWMTGRSRAAEGTRWVMWFGDGKFSVVCVEKLLPLSSFSSAFHQATYNKQPMYRKAIYEVLQVASSRAGKIFPACPENDETDTSKVVEIQNKQMIEWALGGFQPSGPKGLEPPEEERNPYKEVYTEMWVEPEAAAYAPPPPAKKPRKSTTEKPKVKEIIDERTRERLVYEVRQKCRNIEDICISCGSLNVTLEHPLFIGGMCQNCKNCFLECAYQYDDDGYQSYCTICCGGREVLMCGNNNCCRCFCVECVDLLVGPGAAQAAIKEDPWNCYMCGHKGVYGLLRRREDWPSRLQMFFANNHDQEFDPPKVYPPVPAEKRKPIRVLSLFDGIATGLLVLKDLGIQVDRYIASEVCEDSITVGMVRHQGKIMYVGDVRNVTQKHIQEWGPFDLVIGGSPCNDLSIVNPARKGLYEGTGRLFFEFYRLLHEARPKEGDDRPFFWLFENVVAMGVSDKRDISRFLESNPVMIDAKEVSAAHRARYFWGNLPGMNRPLASTVNDKLELQECLEHGRIAKFSKVRTITTRSNSIKQGKDQHFPVFMNEKEDILWCTEMERVFGFPVHYTDVSNMSRLARQRLLGRSWSVPVIRHLFAPLKEYFACV

>Ggal_NP_996835.1

MPARSAPPPPALPPALRRRLRDLERDEDSLSEKETLQEKLRLTRGFLRAEVQRRLSALDADVRCRELSEERYLAKVKALLRRELAAENGDAAKLFSRASNGCAGNGEEEWERGGRGEDGAMEVEEAAASSSSSSSSSSSSSSSSSSSSSLLPAPRARKARRSRSNGESKKSPASSRVTRSSGRQPTILSVFSKGSTKRKSEEVNGAVKPEVSAEKDEEEEEELEEKEQDEKRIKIETKEGSEIKDEITQVKTSTPAKTTPPKCVDCRQYLDDPDLKFFQGDPDDALEEPEMLTDERLSIFDANEDGFESYEDLPQHKVTSFSVYDKRGHLCPFDTGLIERNIELYFSGAVKPIYDDNPCLDGGVRAKKLGPINAWWITGFDGGEKALIGFTTAFADYILMEPSEEYAPIFALMQEKIYMSKIVVEFLQNNRDVSYEDLLNKIETTVPPVGLNFNRFTEDSLLRHAQFVVEQVESYDEAGDSDEPPVLITPCMRDLIKLAGVTLGKRRAVRRQAIRHPTRIDKDKGPTKATTTKLVYLIFDTFFSEQIEKDEREDDKENAMKRRRCGVCEVCQQPECGKCKACQNMVKFGGSGRSKQACLQRRCPNLAVREADEDEEVDDNIPEMPSPKKMLQGRKKKQNKSRISWVGEPIKSDGKKDFYQRVCIDSETLEVGDCVSVSPDDPTKPLYLARVTAMWEDSSGQMFHAHWFCPGSDTVLGATSDPLELFLVDECEDMQLSYIHGKVNVIYKPPSENWAMEGGLDMEIKMVEDDGRTYFYQMWYDQEYARFETPPRAQPMEDNKYKFCLSCARLDEVRHKEIPKVAEPLDEGDGKMFYAMATKNGVQYRVGDSVYLLPEAFSFSMKPASPAKRPKKEAVDEDLYPEHYRKYSEYIKGSNLDAPDPYRVGRIKEIFCHIRTNGKPNEADIKLRIWKFYRPENTHKSMKATYHADINLLYWSDEETTVDFCAVQGRCTVVYGEDLTESIQDYSAGGLDRFYFLEAYNAKTKSFEDPPNHARSSGNKGKGKGKGKGKGKGKSSTTCEQSEPEPTELKLPKLRTLDVFSGCGGLSEGFHQAGVSETLWAIEMWEPAAQAFRLNNPGTTVFTEDCNVLLKLVMSGEKTNSLGQKLPQKGDVEMLCGGPPCQGFSGMNRFNSRTYSKFKNSLVVSFLSYCDYYRPRFFLLENVRNFVSFKRSMVLKLTLRCLVRMGYQCTFGVLQAGQYGVAQTRRRAIVLAAAPGEKLPMFPEPLHVFAPRACQLSVVVDDKKFVSNITRTYSGPFRTITVRDTMSDLPEIRNGASALEISYNGEPQSWFQRQIRGSQYQPILRDHICKDMSALVAARMRHIPLAPGSDWRDLPNIEVRLSDGTSTRKLRYTHHEKKNGRSSSGALRGVCSCAEGKPCDPADRQFNTLIPWCLPHTGNRHNHWAGLYGRLEWDGFFSTTVTNPEPMGKQGRVLHPEQHRVVSVRECARSQGFPDTYRLFGNILDKHRQVGNAVPPPLAKAIGLEIRACVGARMREESGAAVAPPAPEKMEMTAAAD

>Ggal_XP_015151885.1

MSMVTSLERLEQRLAVMVAAQETSQAWSSLGEEGPAAMKKEKSHGRDEADCRAELILFDGDCTDPTKDTAALLLEASGKPGAPDAGSLGLAPLKNKRVSKDLSKEELSWPLTLMEPQEVRPRGSAGWESSLRQKPPVRLIFQAGQTHHEMQIKESSSVEALRELPAPLRSSRRRTAVPVLTTIDLTEEDSRDSSQSSSTLSGSSSQEGQNGSTELVAEEPESRDAGIALEYQDGKEFGIGELVWGKIKGFSWWPAIVVSYRATSKRQAVSGMRWVQWFGDGKFSEVSADKLVGLMAFRQHFNTATFNKLVSYRRAIYHALEVARSRSGKTFTSAPGESLEEQLKPMIDWALTGFKPLGVKGLQPPKSSENGALRNGTEEVLCLEHCPPTKRLKSNPCNSSKEQRMEEDQTREQMVSEVTNNSGSLEDSCLSCGRRNPATFHPLFEGGLCHTCRDRFLELFYMYDEDGYQSYCTVCCEGKELLLCSNASCCRCFCVECLEVLVGRGSSAKAKEQEPWNCYMCQPQRSYGVLQRRQDWSSRLQDFFTSDKGQEYDAPKIYPAVPPAKRRPIRVLSLFDGIATGYLVLKDLGIQVEKYIASEICEDPLAVGTVRHEGNITYVHDVRNITKRNIEEWGPFDLVIGGSPCNDLSLASPPRKAPYGEAAWPHGAGSPQAAWGRRRSCAVAPSGDTCQALSPPGPPEPVPTPQPNLRGRGRSPPNTPAGSPRTEGTGRLFFEFYHLLNYARPKAGEERPFFWMFENVVAMRVNDKRDISRFLECNPVMIDAIKISAAHRARYFWGNLPGMNRIFGFPLHCTDVSNISRGARQKLLGRSWSIPVIQHLFSPLKDYFACE

>Hazt_HAZT003192

LDLLGVKVDVYFASEVDEAAMTVSEKHFHGRVTRLGDVRHVTDDQIAAIAPIDLVLGGSPCSDLSIVNPKRKGLFDPSGTGILFFEFVRILKSVARANKKHPLLWLFENVASMPTEYRNTISKHLHSEPTAVDARAFTPMSRTRLFWGNIPGEEAMAASCVVSIAQLVACVVFDREAQVTELRCVTTQANSTTDIAGHYFPMRMRGRPDAIWITEIEKVFGFPEHFTDVGNLQPRQRQALLGKAWSVDVMLRILEPLKAMFPALRIIISCDWCYVPQPDVDAVWIFWFGDHYVSKSDEKVCPCLGLPKVKLVAVTDFAEDFNRGAAVQRGSLLQQALLEAIRERCYQTGAMHRLNDSPEDLVEWARGEATHRRASSAVDTLPQLPECVVSPFVRRHLARLYSLRRRRASSPSSAAALTTDDDEEVPNADEDTSDEERLPGPDWIEKAEQLREVRALKRRVDDVCIACASVACSVTDTHPVFHGGVCTPCKEALVVVLAELGRDN

>Hazt_HAZT006446

MSSSHLTNGIESQQHESELPKGGLGFSNGEDGFPLPIPSASMKRKDTARQKSITEMFSFIKKDPQPHNTLQNGCKNASEIAIPSAPFNNQHEVEFDSPKTPVSPNKPVLEADIERDCKTLEIKQKKDDLCDADGIVSSHARDAGNYHILSNGNMKDCDANDSASVQEDKEDCDANDAASVQEDKEDSKVSGDYMSLRFFPESGDEMPLRLQPRDLGTRRLCASSPRPGDEMPLQLQPRDLGTTCLCASSPRPKDEMPLRLIPETWGRDAFAPPAKRSGDYMSLDLIPEILGRDASAPPSPRSGDEISKNCGVIPSMSLKEDKEDCDAKDAASVQEDKEDCDANGAASVQEDKEDCDANDAASVQEDKEDCDAKDAASVKKDNITTSWDSDDPGDEFEGFPSEKRLKLAEPERSYHSSQDQKTSQPAAPKSRCPICRQILDSDSLSFYEGHPQDATEEFIALTDPKLAIFMDDDLEERPQHKLTSFTVYDKQGHVCPFDGGLIERNALLYTSGYIKPIFAEDPTPLDGVPVMDVGPINEWWISGFDGGEKALLGFSTGYAEYVLMDPSPVYKQYVDAVMEKIYLSKLVIEFLYDNEDGGYEDLLNLLQTTVPPPDVVLSEDSLLRHAQFVCDQVHNFDVAGDGSDVLITHPCLRTLVDLAGVTLGKRCALKTRGPRIKTVKKMPKWTKATTTPLVRRCFDQFFADQIDSHKGDNSANDTKEKSGPRRMRCGVCEACLKQDCGECSSCKDMIKFGGSGRSKQCCKDRRCPNMMLAEPDDENEEEAVQKLANLRDAAAQTRSHRVKKTTDKLCWVGEASYRTSKRVYYSSARVNEDVVCRGDCVQIEPDAGVKDLPYIARVVSLWEDMKGEKYLHADWYCRGKDTILGDTSDPQELFIIDDCEDIPLASVMKKVRVVPHHPPPNWRLLGGILHPEDLNPLASDDTHSFFCQLAYVPQHARFIHLSSEQVTIYENELATGSRDCPCCERLRLAELRDSCVLMEKSTETDEYNAVQYMDQKFSVGDCILVEPSAFAFKVKLPSPASRAQRQQEHWDEDLFPEYYRLTQKIKGSNEDTSEPFRVAQILSIRPHSKSASCRAGEVFCGSALPPASVTAVVRKFYRPENTHRGAAAAYQAPLNLLYWSDEGSYIRCCAESSVSFSWVCGKCTVVYAENLTCSPDEFFLQGPYRFIFSQAYDATTKELVEPPKSAINLGSSGKGKGKGKGKGSSNSNTTAVLEDYPSITRRLRTLDVFSGCGARDIVAGLSEGFHQAGVAESCWAIEVFEPAANAYKLNNPNATVFTDDCNLLLRMAMENKTTNSKGQTLPRKGDVELLCGGPPCQGFSGMNRFNSRQYSLFKNSLVASYLSYCDFYRPRFFLLENVRNFVSYKCGMVLQLTLRVLVQMGYQCTFGILQAGSYGLCMKKNRAVKEIADAHDSCAALVRGNGDAVRRLAPGEQLPYFPEPLHSFSPHACSVSAAVADVKYKSNCRWTVCGPLRTITVRDALSDLQPIQNGGGREQVAYTTEPESHFQRMVSLGNLYAAERLHYLSNGLQLPIVRASDGLLLDHQCKSLSDLVAARMQRIPTAPGSDWRMLPNKPVRLPDGSWTKKLEYNHDDKRNGRSSEGHLRGVCACASGAPCDPMDRQHNTLIPWCLPHTANRHNHWAGLYGRLEWDGFFSTTVTNPEPMGKQGFPDAYRFYGSLLEKHRQVGNAVPPPMARAIGLEIRKCIAATEKLHCSDAST

>Hazt_XP_018026278.1

MADIFTRAGYAPNSRRGLKMMELFAGIGGMRTAALEAGLELDSVHPYEINPSALLAYCHNFSDQEETKVILPKVGCLYKHKPNSKRSTSPKPRNLLGLTADEWHAVSPDLLTMSPPCQPFTRQGLQLDTSDPRSEPLQHVLTLLQTVRELPAMLLLENVKGFETSSSRNLVTRALRNRGYLFQEFLLCPSQLGVPNSRLRYYLLAILSPDTNQTDQAGSHESLTTESCHIQRKFPVCLCFGQNLKASNYVNKAQECQTCHRIIIHDILSLLSRFHFPSYVSPDKFPCNQSESAGETETFADVTPPIAKFLEKEIFSSPLGSSLQPDNQGNILIKSNPNKTVINSTQNDQLDQIIHDDVQKFSECHRPVLEGGSLTLLNSNSGEQASERLVCPIYATPGNDGSNVTPGPSVEDNSTLMKNMSQFELKPKILVRYAMLLDIVDDTSQRSCCFTKAYGRLVEGTGSVFNPSGRAALDAAFETWRSSKCNNEIVTETKGSITEQNNEGRRPLEPLLDHLSDDEISELNNPLLPLKLRFFSPSECLRLMCFPSSFSFPASLTTRQQYQLIGNSVNVLVVTVLLKYLRYWHLNGDIKQDRP

>Hduj_BV898_04582.p01

MASRDVRQVRRVLELYAGIGGMHFALDEYFKSADEAGEEISIMAVDINPSAKLVYDANFPSAPLRQIDIRALKDAHFDGVWLMTLSPPCQPFSRNGNVLDVEDARCESFLFVLAMLARLTNPPENIIMENVKGFETSEACRQMRDVLRKQGYRFKEYLLSPIHFGIPNSRLRYYLTAWRDPADKSELPKELPPDELSEDSVKTVIDAGTNYSELDLRQFLHHSVEIEHSTPPSLLIPLDILAKYLMVLDIKFPTSTTTNCFTSGYGRYFKGTGSMFCPLPEHVFADLVKEYLAHPDDRSTLAAMKLRYFSPVEVAFLMGFPNCFVIPSVVAPRLAWRLLGNSLNVHVVSLLLKQLLRPTSTITTESVAHFRNKL

>Hmag_XP_002166687.2

MGPSLKVLEFYSGIGGVHYALTYAGVSVHVEAAFEINTSANSVYRHNFPQTTLLQKNIEGLNLEDLEHFNADVWTMSPPCQPYTRLGKQEASCDPRAKSFKKLMNILMKMTSPPSFIFLENVKGFEVSDSCEDFLMILNSKGYYTQSFLLSPIDFGIPNSRLRYYLIARYQKKFNFKTTQKPICLNGNLCECEGLDMECACTALASVVQLYKPMKIKDILNVDNSVSSEINSSSFCEPDLKKMLDLLQKRSFPIKFTKISHSILLRYYSLFDIVDSDSNKSCCFTSGYSRFIEGTGSLFSCLPQSDRNLILNKLSITENDNESNIDLLESLQLRFFSPEEIAALLCFPICFSFPDKITEKQKYKLLGNSVNVLVVANVMRYFLFENKI

>Hmag_XP_012557244.1

MPVLTDGREATDHKACGSSKTNRQSKQSTINALFKKSVKKTKRSISPSKEENENKKKRTDGDIKEKNTSFLTNSEKPGVKTVEKCKECRQLLNSNDIKLFQGDHSDALEEFAMLVDPRLSLLSGNEQDFDAYEDRPQHKVTEFSVYDKCGHLCAFDTGLIEKNVELFFSGYVKPIFDENPDIEGGISTKAMGPINEWWIAGFDGGENALIGFGTAFAEYILMRPSDDYASFMNAVTEKIYMSKIVIEFLLNNPDSEYEELLNKLETTVPPENCAKFTEDTLLQHAQFLVEQVESYDSAALDEEDSPRLITSCCMRDLIKLAGVTLGKRRQMRGLKVKEEKKTLGPTLATTTPLVRHVFDTFFKNQIDLKGVTTQRRKRCGVCEICQQPDCGVCRSCKDMVKFGGSGRSKQCCINRRCPNLAVQEAEEDALCSQDEDEPMLKSPSKTDLSPRHHRKGQEKKSFVKWACEDFVERKGKKLYKSVQINNELINIGEFVQVYPTDPSDPLYICRVMYMWEDLNGDKKFHAQWLYRSSETVLGEVGDPSEVFLSDDCDDIKLGAIMSKCNVSSKFASENWFMEGGKEDCIISEENNELFYQKWYDYEDGLFTDPPTEFLLSKFDEKYCPSCERTKTKLKYKQPTLGDELETENSTKSEHCFKTVTWQGVCYSLGDCVYLDPDAFTFKIKQKSELPKNKSVVKDEDEYPELYRKSNDYIKGSNINIVEPFRIGKIISIIKKTDNYGGMKSIMLKVRKFYRPENTHKGMSGTMNCDLNMVYWSNEEATVDFQMVEGKCYVLFADDADMDMFKFTEAGPDRFYFREAYDADKKEFDVPPREAWNSNKGKGKGKSKSTKQQNGLESNFPAYEKVEKLRTLDIFAGCGGLSEGLDQVGVVNSCWAIEFEPSAAQAYRLNNPSAIVFNQDCNNVLKQIMEGKEKDDLGQRLPRRGEVDLLCGGPPCQGFSGMNRFNQREYSMFKNSLVTSYLSYCDYFRPKFFILENVRNFVSFKKSMVLKLTLSCLVKMGYQCEFGVLQAGSYGVPQTRRRAIIIAAAPGEILPKFPEPQHVFASKALSLSVTINSHKYQALKRLHSAPYRTTTVYDAMSDLPEIKNGANKMEIGYDTEPLTHFQKKIRGKHMQVLRDHICKDMSPLVEARMSYIPCIPGSDWRDLPNKVVKLRDGNTTKLLLYLHKDKKQGPGKNGENRGVCACANGKPCDPSDRQFNTLIPWCLPHTGNRHNNWAGLYGRLEWDGFFSTTVTNPEPMGKQGRVLHPEQHRVVSVRECSRSQGFPDSFRFYGNILDKHRQIGNAVAPPMSAAIGQEIRKSIIAKKLRNEQLQSIKEENNHIATF

>Hmag_XP_012561137.1

MNKTSVKLVAKRKRKSEVDSLFDNLSFIYSEKSCSLPKIDLINAKNDGIESLKTHGGRRWCLDSTLYCDRVIDHNSQKFSMAMEDHSQVNQQSNKDKEDMLFKCVKADKNILQNKNKKNKRKSEIDALFDHLSFIYSDKKSCLPKEDKQARKLPNKELVNDENDKEWSLYNKIAHSSEIIDHDTPRTPEIAEQLQINKIFNNDANNNILFEGNKVKSKNKETLQTQLIGGSKLVKKKNFKVGDVVICYFKYFGWWFAKIIQHNKRAAEGNCWVSSFGDHKILMVPLHSLRHHYDFSSKFNQTKMKKPLYKKAVKEFLSELAEQSNFKIMFGQKGYLNELLEWGLNGFAAHVIKDKNKPCQGNLDFTNTSLNQSIDFVIESSDTSNTESSKLTSKDNSESIDFLLKNYSLSPNLEDSDNYFSSDQHSSINSFKNNEVDSKKSLCKVKNRSKINVLKSSIINHTAQSTNLECQNTVIKNSECENIKKNIENYNAQDWFLNKVDHVRKDCLEKMTHTIQRSPDKILSNIEHNDVTCKDFLCNSKHPSFHSNSTPLQTSCLKENSNQTMRLYKDGIKLDVTLLNEKTPKRACSIKNQTLSSVSPNNVSCASITIGKKQKCVIENKYSSKKNDKTCDREIGSDFFQIKIGDLVLGKLKGYDWWFGMVVSHRVIRQRPAANDCHWIRWYGDHKVSEVHLQNIELLTSFSNRYLPSKMQGLYLRAIKELLEEAARRCCKTDLPDDEKRLGVLVDWALNGFQPYGFDKLSIEETLTLEDDAFIQEGSETDSLTHNEGEPLPSPILSEDVKALFDKAANGNMDLNKICLGCGDLKCIMEHPLFIGGLCKECKESFMETAYLYDEDGSQMYCSICSDGREIILCDVPGCYRSYCSVCLDMFCGVGYSRTVSAGLDWHCFMCTGEKVRLIQRRDDWQQRLKNVFSVNNEGYPLPFFYDPVPFEDRPSLRVLSLFDGLSTGYLALSELGLDILSYQASEIDPLAIKVSKVHHSMRVEQIGDVQKITKQDIENWGPFDLVIGGSPCDELSIANPFRKGIYGTGQLFFEFYRILEYCKPQPLTARPFFWLFENVVGMRYTDRAVISRFLQCHPVIINAKEISAQQRTRYFWGNLPGMNRAMCPLPSDRLKLQQCLEKDCGRVAKFDKVRCITTSQNSLKQTKAAMLPVKWTISRNSQLDDGLWLTEIERIFGFPDHYTDVANMGQRDRQKLLGKSWSVPVLRHLFAPLRNYFRSKVYIDNYNSML

>Hrob_116156

MSMFANQASLKENSPVNNKKRKSLNDNVNRNNKISSSSANDNESEASDEKVIDELVEQAEKKMKLDSENKDKSETDITNKELMTTNTTEQLQLINHPKNKFIIERCTECGQILDEQEIVMYPGDSGEAVEEFVALTDSRLSLFTGDEQEIDEADERPQHRLTNFSVYDRMTHLCPFDSGLLEKNVELYFSGYIKPIYDDNPSIEAGGICTRNMGPINQWWTAGFDGGEVPVVGFSTAFAEYILLSPSDAYKPFWDAVSDKTQLSKVVIEFLKESIEKDEQPSYEDLVNRIQVSDRFFLSSNRFTEDSLLQHAQFIIDQLQSYDDTADTEDVLLIIQPCIRTLVKLTGVTLGRKNKSFGNTQQTNRKTRQQQQIAAKTAQKKKDSKATVTPLVREVFDSLFKGQIEGQSSAATKRRRCGVCEACQMPDCGKCRSCMDMIKFGGSGRSKQACLIRRCPYMSIKEAEDDDLLDDNSGEIIKGKKRKMNHNKNSEKTEAVTWIGSPLATKYANGEKIFYLGAYIGEHIVKIGDHVCVRPDDPAVPMYVAQVRSMWQEVVKRNNSYGKAMFHARWLNRGGDTVLGETSDPKELFLVDECQDSPLHFIAYPIKVDYVIQSEMDFFGSGGGASHIPGYQTQSGLGCGDNKGSSDNNDDGKSFFCRLSYDQSKARFEDFSEFDSLDVVKNFLNTCASVNEKSISETPFLDDEVGVDELLTKFNYTTSTFNKNGNGKFLEFMTTITSSLKILNNRVIKYDEDVYPELYRKTEYIKGSNEQVPEPFRIALVVGFFVKTESNSRKVKVEEVNVVVQKFYRPENTNRGLVWFGNPSSHKDLNLLYWSEDRLTVSFRNYTGKCTVTFFDESDLGLQATDVLTRYFNDGSDRFYFTEMYLPDSKSLQDVPSSQIVKELAFYKGGGKGKGKSKNKSSLAKQENVSTDEKKPLRALKCLDVFAGCGGLSEGLHQAGIADTRWAIEKDESASQAFRLNNPSCTVFSDDCNELLSLAISGSEFNSTGQRLPKRGDVELLCGGPPCQGFSGMNRFNQREYSKFKNSLIASYLSYCDYYRPKYFLLENVRNFVSFKRGVVLKLALRCLISMGYQCTFGVLQAGCYGIPQTRRRAIILASAPGYKLPFYPEPMHSFSVRGMQLTVAVDGRRFESCVSRTSGSAPFRTITVRDAMSDLPEVNNGAGHIETGYGGEPVSHFQKMSRRKIGENSYTTLLQDHVCKEMSALVLARMQHIPLAPGSDWRDLPNLEIRLKDGTVTRKLRYTHDDKRVGKTASGNLRGVCSCVETGHCDPAHRQFNTLIPWCLPHTGNRHNHWSGLYGRLEWDGFFSTTVTNPEPMGKQGRVLHPEQHRVVTVRECARSQGFPDTYRFYGTVLDRHRQVGNAVPPPMARHIGFEIKKSLQLSLEKKENDKKP

>Hrob_162653

MFTFAGCVIDIIEEIQSSLLGLRKVSINVQKSDQCHKDNLEKNFQSPEKKKFKSSQLAKKSLKKFKCTLKSSLLLCDQESSYDKIFEIGQIVWARLYFCSFWPAVIIDGAMCGQKQASHDNYWVFWFGDHKVSEVTLNNVVDFRTTFDQNYNNKNFKKNYEKGVSEACKLYYSRIGKNADSDKIALNWISLYDTNDEHADNMLKETDLSIPDWIIKILDEYSKQNERIRIANDSDEEDDDVNSNHSNHDCRVKLNKDIDSNTANQETWFNWKSEKGACLSCRANIGAYNHPFLLGKLCITCKNRLIDNIFVFDTDGFNMFCVICGQAGEVFLCDKLGCNKVFCCRCMTDILGKSQVHAIKSGEMWTCFICDPTSHHLIGCNGVVENWKEELNYMFQPNPSILPRTRRRMKVLSLFDGISTGKVVLDQLDVDLEIYYASEIDEDSLLVSKGRHGGGVVHWLGDVTNINSEMLEKIHPIDLLIGGSPCNEFSVANPGRKGLSEDGTGVLFFEFYRILKELQALMTPDRPLFWLFENVSSMQKHCKATMTRFFMCHPATLDAKYFSAQNRSRLFWGNIPNMYCPPQYNKNLAPDLLNESLFPGRRSQVVKLRTVTTKQNSLLQGCRNEMPVSMLDNNEESLLWIPELERVFGFPDHYTDIGDLPRTRRQKLIGQAWSVHKLELVVNLIYIRFCLIQLNKKSECLRKDNHQKQFRMFKKSKNLGEKRKA

>Hrob_89038

MVLKVLELYSGIGGMHFALNESGIKHEVVAAIDINPVCNAVYRHNFPNTKLIEKCLTSIFLKDFNKMSADMILMSPPCQPFTTIGKQKDVHDERTKSFQYFLSILPKFSKLPGYILMENVKGIESSIMRGEFLKVLSQLNYDYQEFILDPTQFGIPNSRRRYYLLAKLQNVNSSLQTRPDITYSLLRQNDNSTTNISQTDDKSVLRPLRDFLEKQLPEYFEKFRLPKKLYKKYWLMDLVSENSKSCCCFTKGYGRYVEKAGSIFQTSPSEVDLFPYKNHESDEQFVAEVDKLGLRFFTPREIANFLCFPSTFEFPANLNIQQQYRVLGNSLNVHVVSMLLKLLCS

>Hsap_NP_001124295.1_dnmt1

MPARTAPARVPTLAVPAISLPDDVRRRLKDLERDSLTEKECVKEKLNLLHEFLQTEIKNQLCDLETKLRKEELSEEGYLAKVKSLLNKDLSLENGAHAYNREVNGRLENGNQARSEARRVGMADANSPPKPLSKPRTPRRSKSDGEAKRSRDPPASASQVTGIRAEPSPSPRITRKSTRQTTITSHFAKGPAKRKPQEESERAKSDESIKEEDKDQDEKRRRVTSRERVARPLPAEEPERAKSGTRTEKEEERDEKEEKRLRSQTKEPTPKQKLKEEPDREARAGVQADEDEDGDEKDEKKHRSQPKDLAAKRRPEEKEPEKVNPQISDEKDEDEKEEKRRKTTPKEPTEKKMARAKTVMNSKTHPPKCIQCGQYLDDPDLKYGQHPPDAVDEPQMLTNEKLSIFDANESGFESYEALPQHKLTCFSVYCKHGHLCPIDTGLIEKNIELFFSGSAKPIYDDDPSLEGGVNGKNLGPINEWWITGFDGGEKALIGFSTSFAEYILMDPSPEYAPIFGLMQEKIYISKIVVEFLQSNSDSTYEDLINKIETTVPPSGLNLNRFTEDSLLRHAQFVVEQVESYDEAGDSDEQPIFLTPCMRDLIKLAGVTLGQRRAQARRQTIRHSTREKDRGPTKATTTKLVYQIFDTFFAEQIEKDDREDKENAFKRRRCGVCEVCQQPECGKCKACKDMVKFGGSGRSKQACQERRCPNMAMKEADDDEEVDDNIPEMPSPKKMHQGKKKKQNKNRISWVGEAVKTDGKKSYYKKVCIDAETLEVGDCVSVIPDDSSKPLYLARVTALWEDSSNGQMFHAHWFCAGTDTVLGATSDPLELFLVDECEDMQLSYIHSKVKVIYKAPSENWAMEGGMDPESLLEGDDGKTYFYQLWYDQDYARFESPPKTQPTEDNKFKFCVSCARLAEMRQKEIPRVLEQLEDLDSRVLYYSATKNGILYRVGDGVYLPPEAFTFNIKLSSPVKRPRKEPVDEDLYPEHYRKYSDYIKGSNLDAPEPYRIGRIKEIFCPKKSNGRPNETDIKIRVNKFYRPENTHKSTPASYHADINLLYWSDEEAVVDFKAVQGRCTVEYGEDLPECVQVYSMGGPNRFYFLEAYNAKSKSFEDPPNHARSPGNKGKGKGKGKGKPKSQACEPSEPEIEIKLPKLRTLDVFSGCGGLSEGFHQAGISDTLWAIEMWDPAAQAFRLNNPGSTVFTEDCNILLKLVMAGETTNSRGQRLPQKGDVEMLCGGPPCQGFSGMNRFNSRTYSKFKNSLVVSFLSYCDYYRPRFFLLENVRNFVSFKRSMVLKLTLRCLVRMGYQCTFGVLQAGQYGVAQTRRRAIILAAAPGEKLPLFPEPLHVFAPRACQLSVVVDDKKFVSNITRLSSGPFRTITVRDTMSDLPEVRNGASALEISYNGEPQSWFQRQLRGAQYQPILRDHICKDMSALVAARMRHIPLAPGSDWRDLPNIEVRLSDGTMARKLRYTHHDRKNGRSSSGALRGVCSCVEAGKACDPAARQFNTLIPWCLPHTGNRHNHWAGLYGRLEWDGFFSTTVTNPEPMGKQGRVLHPEQHRVVSVRECARSQGFPDTYRLFGNILDKHRQVGNAVPPPLAKAIGLEIKLCMLAKARESASAKIKEEEAAKD

>Hsap_NP_004403.1

MEPLRVLELYSGVGGMHHALRESCIPAQVVAAIDVNTVANEVYKYNFPHTQLLAKTIEGITLEEFDRLSFDMILMSPPCQPFTRIGRQGDMTDSRTNSFLHILDILPRLQKLPKYILLENVKGFEVSSTRDLLIQTIENCGFQYQEFLLSPTSLGIPNSRLRYFLIAKLQSEPLPFQAPGQVLMEFPKIESVHPQKYAMDVENKIQEKNVEPNISFDGSIQCSGKDAILFKLETAEEIHRKNQQDSDLSVKMLKDFLEDDTDVNQYLLPPKSLLRYALLLDIVQPTCRRSVCFTKGYGSYIEGTGSVLQTAEDVQVENIYKSLTNLSQEEQITKLLILKLRYFTPKEIANLLGFPPEFGFPEKITVKQRYRLLGNSLNVHVVAKLIKILYE

>Hsap_NP_072046.2

MPAMPSSGPGDTSSSAAEREEDRKDGEEQEEPRGKEERQEPSTTARKVGRPGRKRKHPPVESGDTPKDPAVISKSPSMAQDSGASELLPNGDLEKRSEPQPEEGSPAGGQKGGAPAEGEGAAETLPEASRAVENGCCTPKEGRGAPAEAGKEQKETNIESMKMEGSRGRLRGGLGWESSLRQRPMPRLTFQAGDPYYISKRKRDEWLARWKREAEKKAKVIAGMNAVEENQGPGESQKVEEASPPAVQQPTDPASPTVATTPEPVGSDAGDKNATKAGDDEPEYEDGRGFGIGELVWGKLRGFSWWPGRIVSWWMTGRSRAAEGTRWVMWFGDGKFSVVCVEKLMPLSSFCSAFHQATYNKQPMYRKAIYEVLQVASSRAGKLFPVCHDSDESDTAKAVEVQNKPMIEWALGGFQPSGPKGLEPPEEEKNPYKEVYTDMWVEPEAAAYAPPPPAKKPRKSTAEKPKVKEIIDERTRERLVYEVRQKCRNIEDICISCGSLNVTLEHPLFVGGMCQNCKNCFLECAYQYDDDGYQSYCTICCGGREVLMCGNNNCCRCFCVECVDLLVGPGAAQAAIKEDPWNCYMCGHKGTYGLLRRREDWPSRLQMFFANNHDQEFDPPKVYPPVPAEKRKPIRVLSLFDGIATGLLVLKDLGIQVDRYIASEVCEDSITVGMVRHQGKIMYVGDVRSVTQKHIQEWGPFDLVIGGSPCNDLSIVNPARKGLYEGTGRLFFEFYRLLHDARPKEGDDRPFFWLFENVVAMGVSDKRDISRFLESNPVMIDAKEVSAAHRARYFWGNLPGMNRPLASTVNDKLELQECLEHGRIAKFSKVRTITTRSNSIKQGKDQHFPVFMNEKEDILWCTEMERVFGFPVHYTDVSNMSRLARQRLLGRSWSVPVIRHLFAPLKEYFACV

>Hsap_NP_787044.1

MKGDTRHLNGEEDAGGREDSILVNGACSDQSSDSPPILEAIRTPEIRGRRSSSRLSKREVSSLLSYTQDLTGDGDGEDGDGSDTPVMPKLFRETRTRSESPAVRTRNNNSVSSRERHRPSPRSTRGRQGRNHVDESPVEFPATRSLRRRATASAGTPWPSPPSSYLTIDLTDDTEDTHGTPQSSSTPYARLAQDSQQGGMESPQVEADSGDGDSSEYQDGKEFGIGDLVWGKIKGFSWWPAMVVSWKATSKRQAMSGMRWVQWFGDGKFSEVSADKLVALGLFSQHFNLATFNKLVSYRKAMYHALEKARVRAGKTFPSSPGDSLEDQLKPMLEWAHGGFKPTGIEGLKPNNTQPENKTRRRTADDSATSDYCPAPKRLKTNCYNNGKDRGDEDQSREQMASDVANNKSSLEDGCLSCGRKNPVSFHPLFEGGLCQTCRDRFLELFYMYDDDGYQSYCTVCCEGRELLLCSNTSCCRCFCVECLEVLVGTGTAAEAKLQEPWSCYMCLPQRCHGVLRRRKDWNVRLQAFFTSDTGLEYEAPKLYPAIPAARRRPIRVLSLFDGIATGYLVLKELGIKVGKYVASEVCEESIAVGTVKHEGNIKYVNDVRNITKKNIEEWGPFDLVIGGSPCNDLSNVNPARKGLYEGTGRLFFEFYHLLNYSRPKEGDDRPFFWMFENVVAMKVGDKRDISRFLECNPVMIDAIKVSAAHRARYFWGNLPGMNRPVIASKNDKLELQDCLEYNRIAKLKKVQTITTKSNSIKQGKNQLFPVVMNGKEDVLWCTELERIFGFPVHYTDVSNMGRGARQKLLGRSWSVPVIRHLFAPLKDYFACE

>Hsap_NP_787063.1

MAAIPALDPEAEPSMDVILVGSSELSSSVSPGTGRDLIAYEVKANQRNIEDICICCGSLQVHTQHPLFEGGICAPCKDKFLDALFLYDDDGYQSYCSICCSGETLLICGNPDCTRCYCFECVDSLVGPGTSGKVHAMSNWVCYLCLPSSRSGLLQRRRKWRSQLKAFYDRESENPLEMFETVPVWRRQPVRVLSLFEDIKKELTSLGFLESGSDPGQLKHVVDVTDTVRKDVEEWGPFDLVYGATPPLGHTCDRPPSWYLFQFHRLLQYARPKPGSPRPFFWMFVDNLVLNKEDLDVASRFLEMEPVTIPDVHGGSLQNAVRVWSNIPAIRSRHWALVSEEELSLLAQNKQSSKLAAKWPTKLVKNCFLPLREYFKYFSTELTSSL

>Lana_g11540.t1

MEVEDLDSKKERKQETSKDLREEIEAPLRKSKKEKKVPINQPTIMSWFEKGVNKRKLSEIKNDSESNDSGSQEEKRIKTEDGGNGSADGDDKDGAKILRCRAEKPEEVKELLNTKACKQTPLKCKECSQLLQDPDLKLFLGDPDDAREEFVTLTDPNLSLFTGEEEEIHSYDERPQHKVTNFSVYDKNTHLCPFDTGMIEKNKELYFSGYVKPIYDENPSIEGGIPTTKMGPINEWWTAGFDGGEHALVGFTTAFADYFLMDPSEAYAPIMNALQEKIFMSKMVIEFLANNQDATYEDLLNKLQVESYDQGADFDENTLLTNSCMRALIKLAGVTLGKRRALRKEVRGAKAVKDKSAHTLAAVTPLVRNIFDSMFDGQIDEKGTAVDIRRQRCGICEVCQQPDCGKCMACRDMTKFGGSGRSKQACQKRRCPNMAVKAADEDDLLEEGNDDTLDTERKAVEPSTPKKKHKLSKKQKSALEWMGKAIKVETKKSYFEAVLINDQKVSCGDFISVSPDDPSEPLYIAKVAYLFEEGGDKMCHAWWFKRATETVLGETADPLEVLQVNECENIQLEFVVSKIQVLFKAPLPDWSMRGGIEEFDSDNVVKEEDGKTFFYQKWYDPELARFEDPPFDSDPKVDSKHKHCASCSRLMELSKKERPKPSDRLSSESDSSSHMYYGTVSRDGVDYKLGDSVYLLPEAFKFNLKAASKKSKQEKKEVDEDVYPEVYRKSSDYIKGSNEFVPEPFRIGTILEIICKKMTCSKIVNEQNVLLKIRKFYRPENTHKGATGSHHTDLNMLYYSNEEVIVDFQLVKGKCMVVFSDDMNISVDEYFSEGPDRFYFSEAYNSETKEFEEPPSKVRRSTAKGKIRRLRSLDVFAGCGGLSEGFHEAGIAECRWAIEKEEPAASAFKLNNPGSTVFTDDCNMLLRLVMDGKTTNATGQKLPLKGDVELLCGGPPCQGFSGMNRFNSREYSMFKNSLIASYLSYCDYYRPRFFLLENVRNFVSFKRSMVLKLALRCLLRMGYQCTFGVLQAGSYGVAQTRRRAIILAAAPGEKLPFYPEPLHVFAPRAMQLSVVVDNRKYQSNISWTTSAPFRTITVRDTMSDLPTIHNGAKGEEISYGGDPLSHFQKMIRGNQYQPILRDHICKEMSPLVYARMKHIPLAPGSDWRELPNIIVRLSDGSHTKKLVYTHRDKKNGHSSTGALRGVCSCAEGGVCDPMDRQFNTIIPWCLPHTGNRHNHWAGLYGRLEWDGFFSTTVTNPEPMGKQGRVLHPEQHRVVSVRECARSQGFPDTYRFYGNILDRHRQIGNAVPPPMAKAIGLEIKKCIIWKEEHLELSSCFTKEEKEIEPSAVSGIKVDNI

>Lana_XP_013412300.1

MAWQGSPLELKRMNHLLQQSRASGVYCRWLATLQRSHKISTAATACTPQKDEKDPMEKLQKNPYFEKYKGKIHDLQQNSPEEFESRLEEFKKKGQPKEKKVEPKAKKQPVSAAASKMSQTSKLSQLKMTKEKTLNDIMKVDLLYDKSAQEIKQIWQQHFSKKKNTVFAVIPVKTYEVIHERSSTYPMFLYTLPRGDGYEFYVASFSGHECYFTPLIAYQAHQADSPVCLTLHHYSELGDEKGIVLMHGEYNSDILNAQDAQLLCQQVQIYYGPESTSYHLVETFNEQPDNFTDLISQLEKLTDNNEDGLNPVMGSVAALVTSIMKSSIKYKVVAAVDINDVANEVYKYNFQDINLLQRSIESISLKELNKLSPTMLLMSPPCQPFTRVGLQKDIEDARTNSFIYLLETLPKLEKLPNYILIENVKGFECSQTRDKMVSTLRECGYNFQEFLLTPLQFGVPNSRMRYYMVAKKKPLTFVFDTCEGKKLQKNPYFEKYKGKIHDLQQNSPEEFESRLEEFKKKGQPKEKKVEPKAKKQPVSAAASKMSQTSKLSQLKMTEEKTLNDIMKVDLLYDKSAQEIKQIWQQHFSKKESTVFAVIPVKIYEVIHERSSTYPMFLYTLPRGDGYEFYVASFSGHECYFTPLIAYQAHQADAPVCLTLHHYSELGDEKGIVLMHGEYNRDILNAQDAQLLCQQVQIYYGPESTSYHLVETFNEQPDNFKHTDLISQLEKVLPLR

>Lana_XP_013412655.1

MCERVIEFYSGIGGMHLALKESSIKYKVVAAVDINDVANEVYKYNFQDINLLQRSIESISLKELNKFNPTMLLMSPPCQPFTRVGLQKDVEDARTNSFIYLLETLPKLEKLPNYILIENVKGFECSQTRDKMVRTLRECGYNFQEFLLTPLQFGVPNSRMRYYMVAKKKPLTFVFDTCEDKVFEHIPSCAKPFMTHMKPSGPGDHMVCCYCQNPIKKTDILGSSADTQKNGQTGNNLTDFDKINDTEDSTNEEPKAKAPRLDSGTVNSGNSNEDKDSGDITASKRQSCEKCTLSAGVIASNQDYSPQFCQSIDHYLEKQPEEYLEKFLLPEKDLKRFKVMDIVLPSSRKSCCFTKRYGHYMEGAGSVFQMVLEEMNTSILKDDMIPEKAVEYMDDIKKLKLRYFTPREIANLMCFPKEFEFPNKLSRKQCYRVLGNSLNVHVVAVLIKLMTMNVVQKNDS

>Lana_XP_013416790.1

MLKLLVLSRPGPSFAPKQDTRAAPQSGAFSFDEANQWRLLRQCIGNDLLNQKSTTWVTNSYKKTLKHLNCSESQDIYSIPPEKKHGYEYQGDHNYVKVTLPEKIILSHPATDCSAGGHFSEQEEMAKNEQEEDHMDITSALTNCSLTGLSQTDQHKVPKRKSLVGQKELESILNGQDSFMPTRGRLRTRDLSQPKEKEPSITQLLKKQPERGGSNMAYKFRPTPAKVEKFVIDWKNATETSSSWNKGSLQIENAKVKNLHDGEHLSSCEVRIKRLDNKKHGDRPQKGAELPVSNSVAEVTADQPIKTRHSSKTVTHAESSVSTEFDGESNTDDIGQVVWAKFGGQNWWPGMIIKGAYCSVRPARPGSVWVYWFGDHLISELACKHVVPFTEEFSRLNNQCSRKLYRKGVIEAIKVCADRAEKDVSDFDEDQLISWARQGFRVDGKVKHCLFHPPKDHPVPQSVKEYLKKIRDKRNALAEEHEEEETGSQEDITDKKKKDSVLDSVRNGTIRIEDVCIACDDQKEIVGQHPYFNGGLCEDCEEELRVTMFAYGEDGSLTYCIICGNGGVLFVCDVEDCNRKSRVSIRLRRKMLKLLELSRPGPSFAPKQDTRAAPQSGAFSFDEANQRKLLQQCIRSDLVNQKSTIWVTNSYKKTLKHPNCSESQEIYSIPPEKKHGYEYQGDHNYVKVTLPEKIILSHPATDCSAGGHFSEQEEVAKNEQEEDHMDITSALTNCSLTGLSQTDQHKVPKRKSLVGQKELESILNGQDSFMPTRGRLRTRDLSQPKEKEPSITQLLKKQPERGGSNMAYKFRPTPAKVEKFVIDWKNATETSSSWNKGSLQIENAKVKNLHDGEHLSSCEVRIKRLDNKKHGDRSQKGAELPVSDSLTEVTADRSLRKRHSSETVTHAKSSVSTEFDGESDTDDIGQVVWAKFGGQNWWPGMIIKGAYCSVRPARPGSVWVFWFGDHLISEIACKHVIPFTEEFSRLNNQCSRKLYRKGVIEAIKVCADRAEKDVSDFDEDQLISWARQGFRVDGKVKHCLFHPPKDHPVPQSVKEYLKKIRDKRNALAEENGEEETGSREDTADKEKKDSVLDSVRNGTIRIEDVCIACDDQKEIVGQHPYFNGGLCEDCEEELRVTMFAYGEDGSLTYCIICGNGGVLFVCDVEDCNRVYCTRCIESLVGEGSTKVLSEKEMWVCYMCSEYAQETHGLLQRKLDWQQNIMKLYQPSSTVQAPNISDFLPRRAVTVLSLFDGIGTGKVALDSLGIVTDKYYAAEIDDCAVTVTRANHGDSVIQLGDITKLDREKLASIGPIDLVIGGSPCNDLSLVNPARKGIFDGTGQLFFEYYRILTMVKELNQDRHVFWLFENVVSMLREAKTTISRFLGCGPAMWDARYFSAQQRARYFWGNIPGMYSAPQTIHLQNAVNVDGSLTPNCNRRATVKKIRTVTTRSNSLRQKYELFPVKMDGQDDTLWIPELERIFGFPAHYTDVGNLPVAKRHKLLGKTWSVPVIKHLFTPLKQFYLNNNN

>Lcha_ENSLACP00000012502

SPPPKCTDCRQYLDDPDLKFFQGDPDDALEEPEMLTDERLSLFDANEDGFESYEDLPQHKATYFSVYDKKGHLCPFDTGLIEKNVELYFSGAVKPIYDDNPCLDGNIRVFKKKSNIVYIKGFPKSQQLGNALTLAFADYILMEPSEEYSAIFAVMQEKIYMSKIVVEFLQKNPDATYEDLLNKIETTVPPAGLNFNRFTEDSLLRHAQFVVEQVESYDEAGDIDEQPIIITPCMRDLIKLAGVTLGKRRAARRQAIRHPTKIEKDKGPTKATTTKLVYQIFDIFFSEQIEQNNKENIFKRRRCGVCEVTHLCINGSVYGLESCTKFSKNSVSIYSTPCTKFCLMCPRLGIADVVGEETVTWNFLDFATFTNDLKKKKRTHQKPRIVYIKGSLQSDGKKDYYLKASIDAEVLEVGDCVSVSPDDPTKPFFLARITALWEDDDGKMFHAHWFWRGTDTILGETSDPLELFLVDECEDMQLSYVQNKVNVLYKKPSENWFMEGGMDDDIKVVEDDGKTYFYQMWYDPEYARFESPPSCVSAEDNKYKFCLSCTRLDEIRQKEIPKVLEPIDESDAKVCYGLATKNGAQYRVGDGVYLLPEAFSFNLKPASPAKRPKKEEVDEDLYPENYRRSSDYIKGSNLDAPEPYRIARVKEIFCNKRSNGKPNEADIKLRIYKLYRPENTHKSLKASYHADINLLYWSDEEVTVDFKDVQGRCSVEYGEDLTESIQEYSAGGLDRFYFLEAYSAKSKTFEDPPNHARSAGNKGKGKGKGKGKGKGKASVPSEQTEQETAESKVLKLRTLDVFSGCGGLSEGFHQAEIAETLWAIEMWEPAAQAFRLNNPGTTVFTEDCNVLLKLVMAGEKTNSLGQKLPQKGDVEMLCGGPPCQGFSGMNRFNSRTYSKFKNSLVVSYLSYCDYYRPKFFLLENVRNFVSFKSSMVLKLTLRCLVRMGYQCTFGVLQAGQYGVAQTRRRAIVLASAPGEKLPMYPEPQHVFAPRTCQLSVVVDDKKYVSNISLISSAPFRTITVRDTMSDLPEVRNGASALEIAYNGEPQSWFQRQIRGSQYQPILRDHVCKDMSALVAARMRHIPLAPGSDWRDLPNIEVRLSDGTTTKKLRYTHHDKKNGRSGTGALRGVCSCAEGKPCDSADRQFNTLIPWCLPHTGNRHNHWAGLYGRLEWDGFFSTTVTNPEPMGKQGRVLHPEQHRVVSVRECARSQGFPDTYRLFGNILDKHRQVGNAVPPPLAKAIGLEIKSSMLEKLKANSSDVVKQEKMDTTD

>Lcha_XP_005999072.1

MARLRALELYSGIGGMHYALRETCIPAEVVAAIDVNTTANDVYKYNFRDTPLWPKTIEGITLEEFDTLAFDMILMSPPCQPFTRIGLQGDVLDPRTKSFLHILDFLPRLLKLPKYILIENVKGFETSSARDVLIKTLERCAYTYQEFLLSPTCLGIPNSRLRYFLIAKLQPEPFCFQTTNQILEKFPNPHCLDTDGSKPIADFQEQPSLTASEAKPKGPSNLTGRGGRQHHGKEKEAVIFKLETAGEVVKKQEQDNDLCVQMLKDFLEEDVQDLSQYLLPPKSLLRYSLVMDIVKPTCRRSTCFTKGYGHYIEGTGSVLQTALEVEVETAFKSFESLSEEEKLRQLSTLKLRYFTPREIANLQGFPSEFKFLEKITRKQCYRLLGNSLNVHVVAKLITLMCG

>Lcha_XP_014343300.1

MPSNATTPTTTSTSTISTITTTSSPALEQKPSETQKSEEKPEENRNKEEKQGNGTAMKKMGRPGRKRKQLLVESREISKEGVMAPKCHSTQASKGSGQVQNGDLLKSQQETGSSQQREEKSVPGNCVPTEQNITGGECLSDGTHVTGNREVGNSIENGCCSPKDGQDSEKGDSDVPSPPKKKRGRRKLSETAEKKKEERDDPELDSVKTEAEKRARLAAVMNALEEGAESDAQKEEEEASPPTSLQPTAPASPIVAPTPEPVLADAVEKSTSKSVTDDEAEYEDGRGFGIGELVWGKLRGFSWWPGRIVSWWMTGRSRAAEGTRWVMWFGDGKFSVVCVEKLMPLSSFSNAFHQTTYNKQPMYRKAIYEVLQVASTRAGKIFPACPESDETDTSKAIDIQNKQMVEWAFEGFQPTGPKGLEPPEEERNPYKEVYTEMWVESEAAAYAPPPPAKKPRKSTEKPKVKEIIDERTRERLIYEIRQKCRNIEDLCIACGSLNVSLEHPLFIGGMCQNCKNCFLECAYQYDDDGYQSYCTICCGGREVLMCGNNNCCRCFCVECVDLLVGPGAAQAAIKEDPWNCYMCGHKGIYGLLRRREDWPSRLQLFFANNHDQEFDPPKLYPPVPAEKRKPIRVLSLFDGIATGLLVLKDLGIQVERYIASEVCEDSITVGMVRHQGKIMYVGDVRNVTRNHIEEWGPFDIVIGGSPCNDLSIVNPARKGLFEGTGRLFFEFYRLLHEARPKIGDNRPFFWLFENVVAMGVSDKRDISRFLECNPVMIDAKEVSAAHRARYFWGNLPGMNRLVRERPLVATVNDKLELQECLEHGRVAKFSKVRTITTRSNSIKQGKDQHFPVFMNEKEDILWCTEMERVFGFPVHYTDVSNMSRLARQRLLGRSWSVPVIRHLFAPLKEYFACV

>Lcha_XP_014350916.1

MPSSSTSTKIVANGTMTTQGLLNGSLHPAEELTENGKDFSVSVLNGDADVMPALEKKKVNGEATVQDRNLKASPIREGVPWAFSFDEGRQRGGVAGWETSLRQRPPPRTIFQAGLTFQTRKRKEDKKELGGKRAFRQKSSVIVIQDRVNPLKPDEAENAFIDLTEEDSKDSAQSNTTGASGTQEAQNGTVDFVHESQLENAKAETLEYQDSKGFGLGELVWGKIKGFNWWPAIVVTWRAPAKRRATSGMRWLRWFGDGKYSEVSVDKLGPLMSFSQYFNSSALKWNSYKKAVYEALEVASKRAGKEFPEENCESLEQRLKSMMEWALGGFEPMGLEGLKPPENPENGFLKHPFLQEMVPEYNPAFKRQKNTSYKPTKEDSEENPRPSKERLIHEVLENNKCLEDFCMSCGSQNQTTFHPLFEGGLCQSCKDTFLETSYMYDDDGYQSFCTICCGDGELLLCGNMNCCRCFCVYCLETLVGPGASEQAKKLDPWSCYMCLPRKRYGILQRRQDWNLKLQEFFASDKGQEFEAPKIYPAVPAEQKRPIRVLSLFDGIATGYLVLKDLGFNIDLYIASEICEDSIAVGTVRHEGRIKYVHDIKNITRENVREKAPPPKKKTPPQPLMGGLYKGTGRLFFEFYRLLSFAKPEKGENRPFFWLFENVVAMGVSDKRDISRFLECNPVMIDAIEVSAAHRARYFWGNLPGMNRPLTASQTDKLELQDCLEHSRIAKLGKIRTITTRSNSLKQGKDQLLPVLMNGKEDILWCTEMERIFGFPVHYTDVSNMGRGARQKLLGRSWSVPVIRHLFAPLKDYFACE

>Lgig_171288

MHKLTNFSVYDKQTHLCAFDTGLVEKNVHLMFSGVIKPIYDENSSPEGGVSATQMGPINEWWTAGFDGGEKVLVGIGSAYADYILMEPSEAYADIWNALQEKVNMTKVVIEFLSTNFDATYEDLLNKIHTTVPPKGIASFSEDSLLRHAQFVVDQVQSYDEAADFDEELIMTTPCMRALIKLAGVTLGRRKAMGRQLKPTKEKVKPTQSKATTTALVESIFDSMFNGQIDDKTGNGGVKRRRCGVCAVCQQPDCGTCTSCMDMIKFGGSGRSKQACKLRRCPNMALKEAEEDAALEDDADDDKENEVINEKESAHHALIHPHKGKRVVKEIKWVGEEIAIEGRKKYFKAVDIDGVEIAIGDHVAVKPEDSSIPFYIAQVTSMWESSSKEKLFHADWFCRGSDTVLGEASDPLELFLVDDCEDSNLEYVLDKVTIIYKEPAKDWRMLGGIMDPDSDNVIEKDDGKTFFYQKWYNPDYVRFEDPPKKPDVPQNQSHKFCISCNRLEELRKKETPVVGDEVDGSEESSNKVFYGYVQRDGFQYNVGDCCYIDPEAFDFNVKPSNKKINISKRKVDELLYPEAYRKSSDYIKGSNNECPDPFRIGQIVGIFCKNSSKSKIANVAEIRLKIRKFYRPENTHGKCKVVFSENLLMDTTEYFNKVEDGFYFTEMYDSEKREFEEPPSSARMMGGKGKGKGKGKGKGKGKAREEPTEESKEVKFEKLQMLDVFAGCGGLSEGFHQAGIAESKWAIEIVEPAAQAFRLNNPGSTVFSQDCNFLLRRAMDGEKTDELGQHLPQKGQVDLLCGGPPCQGFSGMNRFNSREYSKFKNSLIASYLSFCDFYRPRFFLLENVRNFVSFKRSMVLKLALRCLIRMGYQCTFGILQAGCYGVSQTRRRAIILAAAPGEKLPFYPEPLYTFAPRAMQLSVCIDEKKYTSNITRTTSAPYRTVTVRDCMSDLPEIRNGAKAEEISYKDDPQTHYQKMIRGNQHQPILRDHVCKDMSALVHARMQYIPLAPGSDWRDLPNIEARLSDGTKSNKLKYTHHDKKNGRGVDGELRGVCSCSEGKSCDSEHRQFNTLIPWCLPHTGNRHNHWAGLYGRLAWDGFFSTTVTNPEPMGKQGRVLHPEQHRVVSVRECARSQGFPDTYRYFGNILDKHRQVGNAVPPPMSKEIGREIKKCLIMKAKEKDAEEKESKMDVEADEKKEEIKEEVKAEGSSS

>Lgig_XP_009052047.1

MAASIELRVLELYSGIGGMNYSLKESGVKYEIIAAIDINTTANQIYQHNFPDHNLMAFGIEKLTLKQYEKWNINTILMSPPCQPFTRVGNKNDVNDIRTQSFLHILQLIHQCKEKPSYILVENVKGFEESEARNKLIEMLTTNHYHYQEFLLTPLQFGISNCRLRYYMIAKLQTVQFLFTPSSKILTSIPNCPEEWLKYLQCSEETDNPVSNKWDQFESCDQNIHQEDIETDYICDKCLKLKYFLESKSDDYFQDYLVEKKDFKWFIVMDIVHPGLKKSMCFTKRYGHYINGAGPILQMSHDLTCDLKTKITTLNSRDFWTEEETKIIEKLKLRYFTPREIANLLCFPSSFSFPDNLSRIQLYRCLGNSLNVHVVSILIRLLVLEKYAS

>Lgig_XP_009055961.1+XP_009055960.1

MSSRRQSTFLSHLQNTRIPVPVKYVNVVKRKTCQYNGPQTDSADSLSSGSTQFIKRKNLNKMNRKKEKHCRKLMHMLHDVQDSIRMTRATTKLMEQPTDTETPSSSSPDRQSNLSNLIRYRYPSHKHLLKRLHRKCKNRYYTMAQLARKLAGSESDDSTDISLDSDDDTGIQITDIQGKQVYSVQNTKAMDNMLPLTINTNQDEGIEFNRIDEPVHDLNAVRIFESSESGSEEDKNTELPLQPHMKMVKNKTNKTSFVIGPMRDDSLRPNPVKRQLFQVLNEQPRKRNKMEKKREPTEQYNALSNTPTSSTGKMKKFHIEVLEDDDDDFIEIESQMGRLVFANLVGLQWWPGIIVKGSMCHLYTENKGCSWIFWFGDHKISLVQQERIIPFAANFIEKSSGKGKMFQKALSEIIKVYGERIGLNACTMSNEQLIEWAQTEIRLIKGSKQSEAETDKKIPDWILGKLEEIGADILKKVEGKPLPKSKRDKMEERSATENAIDAVKEGKCEIKDICIACADSSAAIVCQHPLFKGGVCKKCKVDILETIYALDEDGVMAYCSICGHGGTVFLCDKVGCNRVYCVECIDEMAGSIILTEIEQKKNWQCFMCTEFDIKSHGLIEPNKDWQQRIIQFFETEMVHEMFKTKRPIRVLSLFDGIGTGILVLRELGMNVEEYYASEIDSDAMIVTSVHHGDIVQQIGDITEITEEIISSILPIDLLLGASPCNDLSLANPERKGFNFGGTGILFFDFVRILEIIRKLQDKQRHLFWLFENVASMKLEFKKVISRFLKCPAAMWNSSYFSAQNRSRYFWGNIPGLFSTPSVSSYSHEKVMLDNFLTKKCKRQATVDQLRTVTTRTNSLRLGKDDKGFPIKMDGQDAGIWIPELERVFGFPSHYTDVQNLPSTRRQKLLGKAWSVTVLKNILKPLKQYFQCSAEEGKR

>Locu_ENSLOCP00000009053

LFVKQTKFKLEMPAKTSLSLPEDVKKRLQVLDTDGDGLTDEERVRETLSLVLGYLKADAQNQLNNLESKLKKEELSVEGYLSKVKALLGRELCLENGSSELNGQANGCAENGAHGASDEDCGPRNGQGAEGTVEAREEEGLKSPSTGKPRGGRRSKTEGDTKIYVSLSPGVLRGWIPNCVMYTNPFYNQSSFPTGGVPCDTLVGESGKCWRTLAIRKQGGRRRRNSEPGRMLRQISESFSSSRVTRNSVKQPTILSMFAKSSSQPTSLGLLFNVFSIVESPLPSSSKRKSDEVNGEEVAPVTEEGVTDDEEKAQDEKRLKVESEEKTDTKASVQKNPVPPAKTPPPKCPDCRQYLDDPDLKFFQGDPDDALDEPEMLTDERLSLFDANEDGFESYEDLPQHKITNFSIYDKKGHLCPFDSGLIEKNVELYFSCAVKPIYDDNPCMDGGVPAKKLGPINAWWITGFDGGEKALIGFTTAFADYILMDPSEEYAPIFALMQEKIYMSKIVVEFLQKNPEATYEDLLNKIETTVPPAGLNFNRFTEDTLLRHAQFVVEQVESYDEAGDSDEQPIIVTPCMRDLIKLAGVTLGKRRAARRQAIRHPTKIEKDNKGPTKATTTKLVYQIFDTFFSDQIEQNDKESSAKRRRCGVCEVCQAPDCGKCTACQDMIKFGGSGRSKQACLKRRCPNLAVREAEDDENIDEDDVLPVQTTPKKMSQAKKKKQSKSKLSWVGEPVKTVGKKDYYMKVSVEDEILQVGDCVSVSPDDPSKPLYLARITALWEDNNGKMFHAHWFCRGTDTVLGESSDPLELFLVDECEDMQLSYVKGKVNVMYKAPSNNWFMEGGMDSEIKVIDDDGKSFFYQLWYNPEYARFETPPNVSPAEDCEIMFCDSCTRTKELMEQEIPRVLEPLSEDHDSKVFYGLACLRGEQYRVGDGVYMLPESFSFSVKSASPVKRPHRKDDVDEELYPEYYRKSSDYIKGSNLDAPEPFRVGRIKEIFCLKRSNGKPNETEIKLRLYKFYRPENTHKGPKAGYHTDINMLYWSDEEVTVDCKDVQGRCTVEYGEDLIESVQEYSSAGPDRFYFLEAYNSKTKSFEDPPNHARSASHKGKGKGKGKSSSSTVQEQLDQEPQDLKPHKLRTLDVFSGCGGLSEGFHQAGVAETLWAIEMWDPAAQAFRLNNPGTTVFTEDCNVLLKLVMSGEKTNSLGQKLPQKGDVEMLCGGPPCQGFSGMNRFNSRTYSKFKNSLVVSYLSYCDYYRPKFFLLENVRNFVSFKRSMVLKLTLRCLVRMGYQCTFGVLQAGQYGVAQTRRRAIILAAAPGEKLPKYPEPLHVFAPRACSLSVVVDEKRYVSNITRVNSGIYRTITVRDTMSDLPEVRNGASALEISYNGEPQSWFQRQIRGSQYQPILRDQICKDMSALVAARMRHIPLAPGSDWRDLPNIEVRLSDGTTTKKLRYTHSDKKNGRSSSGALRGVCTCAEGKPCDPADRQFNTLIPWCLPHTGNRHNHWAGLYGRLEWDGFFSTTVTNPEPMGKQGRVLHPEQHRVVSVRECARSQGFPDTYRLFGNILDKHRQVSLVDLQKCF

>Locu_XP_006634398.1

MENLKVLELYSGIGGMHYALKESSVPAEVVAAVDVSTTANEVYRHNFPSTPLWAKTIEGVTLSDFNKIGFDMVLMSPPCQPFTRIGLQGDVSDHRTKSFLYFLDILPRLNKLPKYILLENVKGFETSSARNVLVRTLEECKYTYQEFLVSPTCIGIPNARLRYFLIAKAPPNLFSFETTVEIMVDFPSSGSTNVSSYKECSASSEPGHKTGGEQAVVYKLETAKDLERKKSQDSDVSVRRLQDYLEEEPKDLTQYLLPPKTLLRYSMLLDIVKPTCRRSVCFTKGYGYYVEGTGSVLQTCLNTQLEDVFKSLHLLSDEEKLNQLLKLKLRYFTPREIANLLGFPAEFGFPEKITTKKQYRLLGNSLNIHVVSRLIHLMFA

>Locu_XP_015204065.1

MPSNATATTTTFKSSDFDYDAAANKMSEDQEQDRGKEPGNSVPLRKVGRPGRKRKQLPVNSCETSKEDVGSKCHASVESQSSEQVHNGDVDSHPGDTIAASSKQGRNIPENGVPRESVRRSEELPDQEAQGQGYRKGRPDSSLENGFSTPPDDQDSEKDGEQVPPTPRKKRGRRKLEHPEKHTEEQDESSCETVKTEGTRARLRGGLGWETSLRQRPMQRITFQAGDPYYISKRKRDEWLAKWKMEAEKRAKLVAVMNAMEEPAGNEAQKEEEEVVVNQLPPQQHTDPASPTVATTPEPVSAETVDKSIPKSTDVEPEYEDGRGFGIGELVWGKLRGFSWWPGRIVSWWMTGRSRAAEGTRWVMWFGDGKFSVVCVEKLMPLSSFSNAFHQPTYNKQPMYKKAIYEVLQVASSRAGKIFPTCPDTDETDTSKSVDLQNKQMIEWAMGGFQPTGPKGLEPPEEERNPYKEVYTEMWVEPEAAAYTPPPPAKKPRKSTAEKPKIKEIIDERTRERLVYEVRQKCRNIEDICISCGSLNVSLEHPLFLGGMCQNCKNCFLECAYQYDDDGYQSYCTICCGGREVLMCGNNNCCRCFCVECVDLLVGPGAAQAAIKEDPWNCYMCGTKGIYGLLKRREDWPSRLQHFFANNHDQDFDPPKLYPPVSAEKRKPIRVLSLFDGIATGLLVLKDLGIQVERYIASEVCEDSITVGIVRHQGRIMYVGDVRNVTRKHIQEWGPFDLVIGGSPCNDLSIVNPARKGLYEGTGRLFFEFYRLLHEARPKEGDDRPFFWLFENVVAMGVSDKRDISRFLECNPVMIDAKEVSAAHRARYFWGNLPGMNRLVRERPLAAMTNDKLDLQDCLEHGRTAKFSKVRTITTRSNSIKQGKDQHFPVYMNEKEDILWCTEMERVFGFPVHYTDVSNMSRLARQRLLGRSWSVPVIRHLFAPLKEYFACL

>Locu_XP_015220486.1

MKQAREKILGPEMPSNKYSSTIMASDKMTATAAVNSDVAMDGLSEKDSGLELTNGHLGSSPLTASEPPSPLSPKQNGGSISPEINMDKPSSCKKRLRKNSELEDESAWDSSYSEEKSRGVAGWETSLRQRPPPRTIFQAGLSPHGKPRSRDSSLKPKQDESSSLNQNGGSPSGLQAHLPEAPSLELMEQDSKDSAQSSSTSTSQETASSSTESGCQPEYKDNKGFGIGELVWGKIKGFSWWPGIVVTWRATGKRQASPGMRWLQWFGDGKFSEVSADKLDSFMAFPKYFNQASYNKLASYRRAIFQALEMASTRSGKTFPLCENDSPEDQVKPMLDWANGGFQPKGLEGLKPSENTVTGSLNHHVLEVSLPEYFPPAKKQKPCKPKLGSEEDYSREQMVHEVLKNNRSIEEFCLSCGKTRAATFHPLFEGGLCQTCKDVYLEISYMYDDDGYQSYCTVCCGGREVLLCGNANCCRCFCVDCLDILVGAGAAESAKVLDPWRCYMCQPPQQYGVLKRRGDWSVKLQEFFVNDNGQEFESPRTYPAVPAEQRRPIRVLSLFDGIATGYLVLKDLGFKVDQYIASEVCEDSISVGVVRHEGKIQYVHDVRNITRKNIEEWGPFDLVIGGSPCNDLSIVNPARKGLYEGTGRLFFEFYRLLSFAKPKEGEDRPFFWMFENVVAMGVNDKRDISRFLECNPVMIDAIEVSAAHRARYFWGNLPGMNRPLCASGMDKLELQDCLEHGRLAKFGKVRTITTRSNSIKQGKDQHFPVIMNGKEDILWCTELERIFGFPVHYTDVSNMGRVARQKLLGRSWSVPVIRHLFAPLKDYFACE

>Locu_XP_015220711.1

MLGKMAVNVRLTSDAGDKYDRVELLAWLNDSLQTKFTKVEQICSGAAYCQLMDWLFPGSMDLNKIKFQAQEELEFIHNYNLLQKSFRKTGVTKVIPVDELVKGAFQINFQFLKWFKKFFDANFYGQIYNALTAREGQIVLPVTEKPVSPPNTKSPNSFTHAGGKEKVDCDFDEELHDPKTKRITFLTHWQETFSWVRQSKLGDIYAYCEVCDLNITIHHRGQVDLKRHQNSKRHCENLKKRKCLTNKTKDNEDVCGGDSTTCGVATLKFIKKYCTSLKSFTKVRDGSRVSNCRARYILGLKHPKDIASICSQTPYCIYLHRNVDVGDGEQADVVLVGFFDEKAGQNRIRLLDVVRPKTETVASVSSCLVETLQKSGLPVLNLSGFHTDCPPEEGAAVLSRLKEMNPNVICVGSLSHLASLACRAGVAASSKDVSQLISNIHSHYSSCSTANDNLKELFANIEPYQADLPTSTQCLAFTNIVKKISDIWPDLISYFQSCDHKSEKVKLICGQLGDHQLRATFLFLSHALEPLCAFQSKLEKQNGDLAKLLKEASGLLRSYAASLLLPEAVVKLLKGHDSKVLENEKNYLPGTELNVGAEVEEYLTKNQQGLGTVWGDFFKDVSLFYASVTAQIMQNLPLNDTLLRNISILLNPSCKLKITGKVVADLATHLGLCKRPEETTQLIDEFLEYQLLENDEAQVLPPSPLQHWSAVLKTALSGTNEQMSIFRKLILTLLSLPYPSMEATKAFSQAFENGDVSLLDETVTENELDDTRDSDLTAGNEDVILVEDGHLSPETTKHPKHCNSSADVIDLTQMKPCAVLLKKINCTSVDNKNEVFIEDDIVWSSSCQEETTRGICGWESSLRQKPPVRKIFQVGANSLENVKKEDNSGSTQTNSGAPGVKRGNHTSTPNKPPKKSYPYQDGKGFSVGELVWGKVKGFSWWPGLVVGWKSKQIPTAMRKVEWFGDGMFSEIYTERLLPFSAFSKCFCNSSFATLPAYKDAIFQSLEMVSERSKKTFASCKTESKDEKLKPMLEWAFGGFKPKGPDGFKPPVTDNLAAKAALLDTPVPEYQPPTKKQKYVYKNKGGNEQEYSRVQMVREVLQKGKNIEDFCLSCGTPQTEIFHPLFEGSLCLKCKDNFTETLYRYDEDGYQSYCTVCCAGLEVILCGNASCCRCFCLDCLDILVGPGTFDKLKEVDPWSCYMCLPSQRYGVLKRREDWSIRVQEFFVNNSAMEFEPHRVYPSIPANQRRPIRVLSLFDGIATGYLVLKDLGFKVERYIASEICEDSIAVGMIKHEGRIEHVHDVRTITKKHIAEWGPFDLLIGGSPCNDLSIVNPARKGLFEGTGRLFFEYYRLLNILKPKEGDNRPFFWLFENVVAMGVRDKTDICRFLECNPVLIDAVKVSPAHRARYFWGNLPGMNRPLASSQSDKLDLQDCLEHGRQAKFSKVRTITTRPNSIKQGKSEILPVVMNGKEDNLWCTELERIFGFPRHYTDVNNMGRGSRQKVLGRSWSVPVIRHLFAPLKDYFACE

>Lpol_XP_013775600.1

MSPDKTRKKLPKDKLLGLVGAFNDNYIDLGGKFFKRGVLEALQEMAVRAGVDVAEGDSVKLLSWAKHGFRTESRRTNPWAPSVENPIPENVRRRLDRIKTQYLEALLLLESQTSLQPRFSRFAQLENSGSALKKVRERKLSIQDVCVACDSSSEDIVTQHPLIEGGLCQQCKDDIIETMFAYGDDGTNAYCVICGQAGELIICDNTDCNKCYCTGCIDVLISPGTHKKVLAADPWFCFICTEYDPERNGLIQPKLDWQQNVLKLFQPEKSIQLSPSSDDYREKKPIRVLSLFDGIGTGRYVLDQLGIEVEAYYASEMDHDAINISIVQHKCNVTHLGQIEEISDAEVAKLCPVDLVIGGSPCIDPSLVSSGRKGLYDATGTGRLFFDFFRLLKAVQLANKERHVFWLYENVTSIPQEYKSIITRFLQCEPALIDSKYFSPQNQARYFWGNIPGMYAVEEYWSGGEVDNPLQPHFHMLRQAVKLNSSLSSNLNQEIETVSSNTMCNNLPSESKDSLTPGNTSDDKDALRVTDIEQVFGFPKHYTDTGNIPLGRRQQLLAKAWSVPIMKHIFMPLRNFFKTFSLPFVPQLSPASTTDILPDSSSSLTTCTNNCVRP

>Lpol_XP_013778629.1

MHHSGDHKRDLEKVQEEGEKGEEENSEEPLDLTGWKSQENKDGLLEEENTGDGKKMSCCYFGLEDEKDPEDISLACLSPISKHSKQNRKRKKKRKNKSKISFEYKKKKKMRPKAKSKSNECELHFSSESADLRNELVPEDLSLYSKSSGTHSCMSEESDSPSEQVTGAIRDLSLNEHSESPQFSVGNAAKNKCHEESTSDDTRSSLVEQEIKAPVKETFDFLTEQKNFNPNEQRLGQRIEEGDQSIISSLGQKVEAKYTEDSTSSSQLEKTEDSEDQLVPFEISPRSTCKISNNYLVKSSTIRHGSNEREECLRPRPSQSLVMTRKRRIAPSLLEGIIGYKQAKLNMHTITAKTSTSRRMGPINKPSYRNHSSKKSSTCSSSSSSQITVALDIFNPEDRELMSDSSNKEMDQDSAQKLLDASVGEIIWAKLGSTRWWPAIVIWGTDCGQPPAHPGQTWVFWFGDHKISELPRDKLVDFVEEFSSKFSDFAGKMFKRGVVEAIREVAVHAEVEFVEGDSAGMLNWAKNGFRTESSKPNPYAPDIATPISENVSKHLNRIKTQYLQALQSFGSQNSLQPRFSRLEQLENKDSALKKVREGKFRIQDVCIACDSTLVEISTQHPLIEGGLCKQCKNETVETMFAYGEDGTNAYCVICGQAGELLICDNNDCNRCYCTGCIDVLVSPGAHKKVLSTSPWLCYMCHDYDPEINGLIRCKEEWQQNVMQLFQPEKHVHLPPSPEDYKEKRPIRVLSLFDGIGTGKFVLDQLGINVDAYYASEIDQDAINVSIVQHKLSINQLGRIEDVTDAEVAKLCPIDLVIGGSPCNDLSLVNPARKGLYDPQGTGKLFFDFFRVLKAVQLANKGRHVFWLYENVASMPQEYKSTITRFLQCDPAMIDSKYFSPQNRARYFWGNIPGMYAVFGFPRHYTDAGNIPLGRRQQLLGKAWSVPVIKHLFTPLKNFFKLVPSSLSSPTTISTIVTPIATTGENGKRICDVEA

>Lpol_XP_013779188.1

MVIRVLELFSGIGGMHCSLNATGISFKIEAAVDINTTANQVYMHNFPDTTLLQRNILSLSSKEIDNLKPDLITMSPPCQPFTRQGLKQDCEDVRTQPLLHILQVISDLEYKPYYIFLENVKGFETSRAKEKLVSTLQSCGYKFKEFLLTPTQFGIPNSRLRYYLLAKFRFGTSCVNMTQELTTSLRCEIKKEDMLFFKNSGLDSEIDSSNTAEGVVCRGGNTTDDEKTPLSFYLENKPDLYFQDYLLPDKVLLKFPMILDIVDRRSLQSCCFTKGYGHYVQGTGSVIHAVSHDKFIEVYKTVSLLNKEHPQTVELLKQLKLRYFTPREVANLLHFPSMFSFPSHLKKKQLYQILGNSINVYVVCELLKLLLQ

>Lpol_XP_013780573_1

MPGLRSVLANGPINDRSELSTEEEIRVIKRLEDLKQKLEDEDVTLKGYWKEVCHVCSDLLPQSTKDQVKITESRFKQKLITEEQYANEVKDLVACIIEKAVEGVNLVNEKNVIEDSEQSEKSDGLKNMHETLESDYENAAGSNEDNMDKDENVARSSESEEVQSEKEAENLDNQGDNTTSSYSASKKEQLTIKDAFSNISKKRRHEDVESKNISNSMVDRNSKRKKSEDSQEERTTNKEVKPPAQPPLKCKECGQLLDNTDLVMFTGDPENSVEEFIMLTDPHLSLFTGEENEDNMEDISERPQHKITQFSVYDRNTHLCPFDSGLIEKNKELYFSGFVKPIYEEDPSSQAFADYILMEPSESYKPFMDAMKEKIHISKTVTEFLVNNPEATYEDLLNRIQTTVPPQGLTSFTEESLLRHAQFVVDQIQSYDSAAADDEFLIIASPCMRALIKLAGVTLGKRRALRRAEREIKVKRPSFSLATTTPLVHAVFEHFFQEQLQKDNNNKGATVRRKRCGVCEACQQPDCGKCIACKDMLKFGGSSRSKQACSKRRCPNMAVQEAEDDDAQNEEMEDDMDRKAKETSVKTMKKHQTKKQKTVITWVDKPHQIENGNKYYKAVECGDSVSVAPEDPSTPVYIARVVYMWESPSGAKMFHAHWFCRGSDTVLGETSDPQELYVVDECEDSQLDFVICKCEVMHKPVSENWGTQGGFHNEENEGLAHDEEKKKFFYQKWYEPQICRFEDPPDLTIPNSKDVRQLCISCKRMTRLRELEIAQVGEILEDREKKIFYKAVSWNTRDFRVGDCVYLLPEAFSFPHKSKTQGSKMISKKDVDEEMYPEYYRKTDYVKGSNLDVPEPFCIGKINSIFKHNNQLGKSNSIKLIVTKFYRPENIHILKTASYMQDLNLLFWSDEEVEVDFSLLQGKCYVVYSGNLTQSIHDFTLTGPDRFYFNEAYNSKTKTFEEPPSIAMSMGGQGKGKGKGKGKGKGKNKPDLEIFPEVVMETYPAVSQKLRTLDVFAGCGGLSEGFHQAGLVDTLWAIEKEDPAAQAFRLNFPDCTVFSDDCNVLLKMVMEGQTCNEKGQRLPQKGEVELLCGGPPCQGFSGMNRFNSRQYSLFKNSLIVSYLSYCDYYRPKYFLLENVRNFVSFKRSMVLKLTLHCLLQMGYQCTFGVLQAGNYGVPQTRRRAIILAAAPGEKLPYYPEPTHTFAPRACQLSVMVDDKKFLSNAKWTTSAPYRTITVRDAMFDLPEIKNGAKAEEIPYNGEPLSHFQKLMRGKHCSPVLRDHICKEMSSLVEARIRNIPLAPGSDWRDLPNLVLRLSDGSYTRKLRYTHHCKKNGRNSFGGLRGVCSCAERKACDPMDRQDNTLIPWCLPHTGNRHNHWAGLYGRLEWKGFFSTTVTNPEPMGKQGRVVHPEQHRVVSVRECARSQGFPDHYRFFGNILDRHRQIGNAVPPPLAKAIGLEILKCVAYEAEKTPRMHVEFEKSKHVSESTSKEHV

>Lpol_XP_013786105.1

MNKDSFNSEDRELLSDISNQELDQDTTNKLLDASVGEIIWAKLGSTRWWPAIVIWGADCGQPPARPGQTWVFWFGDHKISELPRDKLVDFVDDFSSKFTDFAGKMFKTGVVEAIREVAVRAEVEFVESDSAGMLSWAKNGFHTESQKPNPYTPDAGSPIPENVRKHLNRIKTQYLQALQSLGSQNSLQPRFSRMEQLENKDSALKKVREGIIRIQDVCIACDSTSVEIATQHPLIEGGLCKQCKDETVETMFAYGDDGTNAFFELQAYCVICGQAGELLICDNNDCNRCYCTGCIDVLVAPGAHKKVLNTSPWLCYMCRDYDPEINGLIRCKDEWQQNVMQLFQPEKHVQLPPSPDDYKEKRPIRVLSLFDGIGTGKFVLDQLGIEVDAYYASEIDQDAINVCIVQHKLSVTHLGRIEDVTDAEVAKLCPIDFVIGGSPCNDLSLVNPARKGLYDPTGTGKLFLFFRVLKAVQLANKGRHVFWLYENVASMPQEYKATITRFLQCDPAMIDSKYFSPQNRARYFWGNIPGMYAPLQHHLLHQTISLDSVITPHLNRKAIVEKIRTVTTRSNSLRQGKKCLLPVTMNGESDVLWVTELEQVFGFPRHYTDAGNIPLGRRQQLLGKAWSVPVIKHLFMPLKNFFKLVPSSSSSSPATSSPTVTTTATTVSAVVMPTSS

>Mbre_MONBRDRAFT_1464

VYQANHPDTLIKNRNIQAVPIRQLEKLAADLWLMSPPCQPYSRQGHQRGLEDARSDSFRYLLDALTQMKNPPSVILVENVVGFESSQGREELVATLNTLQYRFQEFWLSPDQFGVPNSRLRYFLLAIRGDRFRAEPSATAADAAFGNSSTLMYHLPGALQFEPGLPTSEFGTFDDAYRVAVEQSQVSDYSYPQQVCIAEACAIGSKKKKNNAGCLTSHPPRMGILSSTHLARHLPGSGRLLLRWGRLLDIVRPEERRSLCFTKGYSHKVEGTGSVFQVCICVAIPMAFEQLDVPSPGAGWPAVEALELRYFTPQEMLRLHGFPTDFVIPSSVTDRQARKAIGNSLCVTIVAHLLDL

>Mlei_ML002234a+ML002229a

MVRNVKQTDLRQMFSAKVEKIQKSNEKTNIRTSAVIADEGQSSLTDIASKNVEDNCLAEGEPASKRQKTEKYNREINESTKRKSPALKPVKCDVCKQLLNSEALKVFKGDESGAVEEFIALASDTLSLFDADSNGESEWTEKPQHRITNFTVYDEYGHMCPFDSGLIEKNVPLYFSGYIKPIYDDSPSLEGGVAGSKLGPIDSWYIAGFDGGEKELIGFTTAYADYLLSQPSDQYSPIFDMLREKTYLSKTVIEILVEDSGMTMEDLLNCIQLVVPPENCSKFTEETLLRHAQFLVEQIESYDHAADEDEDLLITIPAVRGLIELAGVTLGGNQQSRARPERPVVKSKAAGKTKSTDISHATVTPLVQSSFEAIFVDQMQATVSFFLSGFSFYRILKFTPTFTSQSKGRKMAVIRAEENAKDETDEPNLAQSNGLKKKLPEVKTEKKVSSNIDGKLTGDPVSKSGGMPVPEKQNEELFFKFWYDSDRARAKISSTKIRKDNVDDEMFPEYYRKTDYIKGNNDLVPELYRIGRIVSFSTKQEGLDGSVVKVTVKKMYRPENIENCRVESADINLLFYSLDQVKIRADKLSGKCTVKNSEDIEDLSRYTTLSDHFYFTESWNSASKCLEDVPSEGRSSNKGKKKRAGIKSEKETQFRKLRTLDVFAGCGGLSAGFHQAGIAESCWAIEFFSEAAQAYKLNNPQAEVFNEDCNAVLRMAMEGVLTNKLGQKIPQKGEVELLCGGPPCQGFSGMNRFNAREYSMFKNSLISSYLSYCEFYRPRYFLLENVRNFVSYKKNMVLKLCLSSLVNMGYQCTFGVLQAGCYGVAQTRRRAFLLAAAPGEVLPQFPEPRHVFSPKAMSLSVTIDNKQYSQHITRFDSAPLRNITVRDIMSDLPAITNGATKRELAYDCEPDSWFQRQIRGNSEVLTDHICKEMNPLVAIRMRHIPLTPGSDWRDLPNISVTLPDGKKTPKLVYTHNDLKNGPMKGVCSCAEGKKCDPMDKQNMTLIPWCLPHTGNRHNNWAGLYGRVFWDGYFSTTITNPEPMGKQGRVLHPEQHRLVSVRECARSQGFPDTYKFYGNLLDKHRQLGCITRSHAFLAWRNSKNSKRASPVDATAVKSDTVAVKRTIKSPSSNVESDTHKLTTSPKKTLTNKSSSSSASKLPKSSSKSVDLNSQSHSLSILSGKRTGSKSKLSERFYNVECFSNGSSTLGSRHYGSYSSSGHSRLASS

>Mlei_ML074813a

MSPAASSTLRVCEFYSGIGGYHIALSQIPHLSFEVAAAFEISSNANCVYRANFPNTTVLETNLCGLTATRLDSIIRKSIGSDSQDEAVTSRDEVMFVMSPPCQPFTRQGIQKDDLDPRSESVLHLFETFSQLTSLPDYFILENVRGFETSNTRNRIMQFFTKHQYRVAEFLINSNQVGIPNSRLRYYLLARKKPFTKAVESALDSSQGFHKIPRLLTSIPGFEVELPTQPLSEYLEPGLTGYDLSDKFLSKWGWVLDLVTPSSTRSCCFTKGYSVKAEGSGSVLQQLSDPSESVGQLSLDGFSGNGGNNERSMCNSRLSKEEFLEHMKSLRLRFFSPREIARIHGIPDSYSFPEVLSEKQLYKLLGNGLNVSVVRLLIEKVLLE

>Mlei_ML35881a

MEDENDSDKANKDPSTFSVLNQTPLSKKKRGKRRKERGIANLSTLPHTYPLEIDEGAYVFLKGIEVWHPGKIMQVTSPYRIICYSFLDTSITVAARSKLSSFLNYGCKVDFDLLESDETYQKGIIDCIASFAEIHRPDLLKLPDRDLMLLDWARLGFVGCLLIQSNIPQILVDYKTENDLNQPTFCVDEEKPSSSRDWDEDDCTPTKRRKLSLHTEDSGYSSPVTPSVLKSPLATLSIDQLQSSSTSSDHNSEVGCYVLGKLVRYGWWPGLIADPRKSPRPCLPNERLVYWFGDCTKSLIDIKDIAPITKFPDVYQPSKKGIYRVALYDFLSDAAESCNYTEINAPDSVNIDESALTSLIKWAMDGFTPGGPPRKWAPSEHYHSIEKDRFTRRIEMAERTNNGSACVACQNRDSIHPHPLFEGHQLCDACLEDFSQCAYLFGDDGAGSYCSVCNEGGNLFICDQSQCGRPYCPACISRVCGPHSLLQVEKSDPWFCVLCNDTGSEGDLLRRPDWQARLQDLLTRKVAGEEEYQTPLPPPFPPISERSSLRVLSLFDGISTGLVTLKNIGFQIETYVSSEVDEEAIKVSKLHHPEVVQIGDIASLTPQKVSDLGPFDLVMGGSPCNDLSNVNPFRKGIYEGTGRLFFEFYRILSYVKESAGDRPVFWMFENVVSMSRSDRNCISRFLQSSPNVIDAKHFSCMSRARYFWGNLPGMARTPVPLATDRLTLQDCLEQNCGREARFEKINCITTKLTSMKQKKGRTLPVRIAGDEEGPGDWLWCTEMERVFGLPSHYTDVANMSRFARQRLLGKAWSVPVLRHLLSPLRDYFMCQQR

>Mmus_NP_001186360.2

MPARTAPARVPALASPAGSLPDHVRRRLKDLERDGLTEKECVREKLNLLHEFLQTEIKSQLCDLETKLHKEELSEEGYLAKVKSLLNKDLSLENGTHTLTQKANGCPANGSRPTWRAEMADSNRSPRSRPKPRGPRRSKSDSDTLSVETSPSSVATRRTTRQTTITAHFTKGPTKRKPKEESEEGNSAESAAEERDQDKKRRVVDTESGAAAAVEKLEEVTAGTQLGPEEPCEQEDDNRSLRRHTRELSLRRKSKEDPDREARPETHLDEDEDGKKDKRSSRPRSQPRDPAAKRRPKEAEPEQVAPETPEDRDEDEREEKRRKTTRKKLESHTVPVQSRSERKAAQSKSVIPKINSPKCPECGQHLDDPNLKYQQHPEDAVDEPQMLTSEKLSIYDSTSTWFDTYEDSPMHRFTSFSVYCSRGHLCPVDTGLIEKNVELYFSGCAKAIHDENPSMEGGINGKNLGPINQWWLSGFDGGEKVLIGFSTAFAEYILMEPSKEYEPIFGLMQEKIYISKIVVEFLQNNPDAVYEDLINKIETTVPPSTINVNRFTEDSLLRHAQFVVSQVESYDEAKDDDETPIFLSPCMRALIHLAGVSLGQRRATRRVMGATKEKDKAPTKATTTKLVYQIFDTFFSEQIEKYDKEDKENAMKRRRCGVCEVCQQPECGKCKACKDMVKFGGTGRSKQACLKRRCPNLAVKEADDDEEADDDVSEMPSPKKLHQGKKKKQNKDRISWLGQPMKIEENRTYYQKVSIDEEMLEVGDCVSVIPDDSSKPLYLARVTALWEDKNGQMMFHAHWFCAGTDTVLGATSDPLELFLVGECENMQLSYIHSKVKVIYKAPSENWAMEGGTDPETTLPGAEDGKTYFFQLWYNQEYARFESPPKTQPTEDNKHKFCLSCIRLAELRQKEMPKVLEQIEEVDGRVYCSSITKNGVVYRLGDSVYLPPEAFTFNIKVASPVKRPKKDPVNETLYPEHYRKYSDYIKGSNLDAPEPYRIGRIKEIHCGKKKGKVNEADIKLRLYKFYRPENTHRSYNGSYHTDINMLYWSDEEAVVNFSDVQGRCTVEYGEDLLESIQDYSQGGPDRFYFLEAYNSKTKNFEDPPNHARSPGNKGKGKGKGKGKGKHQVSEPKEPEAAIKLPKLRTLDVFSGCGGLSEGFHQAGISETLWAIEMWDPAAQAFRLNNPGTTVFTEDCNVLLKLVMAGEVTNSLGQRLPQKGDVEMLCGGPPCQGFSGMNRFNSRTYSKFKNSLVVSFLSYCDYYRPRFFLLENVRNFVSYRRSMVLKLTLRCLVRMGYQCTFGVLQAGQYGVAQTRRRAIILAAAPGEKLPLFPEPLHVFAPRACQLSVVVDDKKFVSNITRLSSGPFRTITVRDTMSDLPEIQNGASNSEIPYNGEPLSWFQRQLRGSHYQPILRDHICKDMSPLVAARMRHIPLFPGSDWRDLPNIQVRLGDGVIAHKLQYTFHDVKNGYSSTGALRGVCSCAEGKACDPESRQFSTLIPWCLPHTGNRHNHWAGLYGRLEWDGFFSTTVTNPEPMGKQGRVLHPEQHRVVSVRECARSQGFPDSYRFFGNILDRHRQVGNAVPPPLAKAIGLEIKLCLLSSARESASAAVKAKEEAATKD

>Mmus_NP_001258674.1

MKGDSRHLNEEEGASGYEECIIVNGNFSDQSSDTKDAPSPPVLEAICTEPVCTPETRGRRSSSRLSKREVSSLLNYTQDMTGDGDRDDEVDDGNGSDILMPKLTRETKDTRTRSESPAVRTRHSNGTSSLERQRASPRITRGRQGRHHVQEYPVEFPATRSRRRRASSSASTPWSSPASVDFMEEVTPKSVSTPSVDLSQDGDQEGMDTTQVDAESRDGDSTEYQDDKEFGIGDLVWGKIKGFSWWPAMVVSWKATSKRQAMPGMRWVQWFGDGKFSEISADKLVALGLFSQHFNLATFNKLVSYRKAMYHTLEKARVRAGKTFSSSPGESLEDQLKPMLEWAHGGFKPTGIEGLKPNKKQPENKSRRRTTNDSAASESPPPKRLKTNSYGGKDRGEDEESRERMASEVTNNKGNLEDRCLSCGKKNPVSFHPLFEGGLCQSCRDRFLELFYMYDEDGYQSYCTVCCEGRELLLCSNTSCCRCFCVECLEVLVGAGTAEDAKLQEPWSCYMCLPQRCHGVLRRRKDWNMRLQDFFTTDPDLEEFEPPKLYPAIPAAKRRPIRVLSLFDGIATGYLVLKELGIKVEKYIASEVCAESIAVGTVKHEGQIKYVNDVRKITKKNIEEWGPFDLVIGGSPCNDLSNVNPARKGLYEGTGRLFFEFYHLLNYTRPKEGDNRPFFWMFENVVAMKVNDKKDISRFLACNPVMIDAIKVSAAHRARYFWGNLPGMNRPVMASKNDKLELQDCLEFSRTAKLKKVQTITTKSNSIRQGKNQLFPVVMNGKDDVLWCTELERIFGFPAHYTDVSNMGRGARQKLLGRSWSVPVIRHLFAPLKDYFACE

>Mmus_NP_001258682.1

MPSSGPGDTSSSSLEREDDRKEGEEQEENRGKEERQEPSATARKVGRPGRKRKHPPVESSDTPKDPAVTTKSQPMAQDSGPSDLLPNGDLEKRSEPQPEEGSPAAGQKGGAPAEGEGTETPPEASRAVENGCCVTKEGRGASAGEGKEQKQTNIESMKMEGSRGRLRGGLGWESSLRQRPMPRLTFQAGDPYYISKRKRDEWLARWKREAEKKAKVIAVMNAVEENQASGESQKVEEASPPAVQQPTDPASPTVATTPEPVGGDAGDKNATKAADDEPEYEDGRGFGIGELVWGKLRGFSWWPGRIVSWWMTGRSRAAEGTRWVMWFGDGKFSVVCVEKLMPLSSFCSAFHQATYNKQPMYRKAIYEVLQVASSRAGKLFPACHDSDESDSGKAVEVQNKQMIEWALGGFQPSGPKGLEPPEEEKNPYKEVYTDMWVEPEAAAYAPPPPAKKPRKSTTEKPKVKEIIDERTRERLVYEVRQKCRNIEDICISCGSLNVTLEHPLFIGGMCQNCKNCFLECAYQYDDDGYQSYCTICCGGREVLMCGNNNCCRCFCVECVDLLVGPGAAQAAIKEDPWNCYMCGHKGTYGLLRRREDWPSRLQMFFANNHDQEFDPPKVYPPVPAEKRKPIRVLSLFDGIATGLLVLKDLGIQVDRYIASEVCEDSITVGMVRHQGKIMYVGDVRSVTQKHIQEWGPFDLVIGGSPCNDLSIVNPARKGLYEGTGRLFFEFYRLLHDARPKEGDDRPFFWLFENVVAMGVSDKRDISRFLESNPVMIDAKEVSAAHRARYFWGNLPGMNRPLASTVNDKLELQECLEHGRIAKFSKVRTITTRSNSIKQGKDQHFPVFMNEKEDILWCTEMERVFGFPVHYTDVSNMSRLARQRLLGRSWSVPVIRHLFAPLKEYFACV

>Mmus_NP_001271126.1

MGSRETPSSCSKTLETLDLETSDSSSPDADSPLEEQWLKSSPALKEDSVDVVLEDCKEPLSPSSPPTGREMIRYEVKVNRRSIEDICLCCGTLQVYTRHPLFEGGLCAPCKDKFLESLFLYDDDGHQSYCTICCSGGTLFICESPDCTRCYCFECVDILVGPGTSERINAMACWVCFLCLPFSRSGLLQRRKRWRHQLKAFHDQEGAGPMEIYKTVSAWKRQPVRVLSLFRNIDKVLKSLGFLESGSGSGGGTLKYVEDVTNVVRRDVEKWGPFDLVYGSTQPLGSSCDRCPGWYMFQFHRILQYALPRQESQRPFFWIFMDNLLLTEDDQETTTRFLQTEAVTLQDVRGRDYQNAMRVWSNIPGLKSKHAPLTPKEEEYLQAQVRSRSKLDAPKVDLLVKNCLLPLREYFKYFSQNSLPL

>Mmus_NP_034197.3

MEPLRVLELYSGIGGMHHALRESHIPAHVVAAIDVNTVANEVYKHNFPHTHLLSKTIEGISLEDFDKLSFNMILMSPPCQPFTRIGLQGDMTDPRTTSFLYILDILPRLQKLPKYILLENVKGFEVSSTRGLLIQTIEACGFQYQEFLLSPSSLGIPNSRLRYFLIAKLQSEPFPFQAPGQILMEFPKIVTVEPQKYAVVEESQPRVQRTGPRICAESSSTQSSGKDTILFKLETVEERDRKHQQDSDLSVQMLKDFLEDGDTDEYLLPPKLLLRYALLLDIVKPTSRRSMCFTKGYGSYIEGTGSVLQAAEDAQIENIYKSLPDLPPEEKIAKLSMLKLRYFTPKEIANLQGFPPEFGFPEKTTVKQRYRLLGNSLNVHVVAKLLTVLCEGFGNASESCHKMPLILDSNSKILS

>Nvec_127267

GVLVWSKLKGYDWWPGRVVTYMEAGRPPPGPGNHWVKWFGDNKFSQVTYDTVLPFAEFKSNFLVTKMKGLYKKAVLDALEVLKKKLLSKDTETPAEPDEPLSEEEKCDAMVNWALTGFQPQGASGFAPTLEETIFLPPVSSDSDEEEADQTVNGSESTKGRFGKPVKEPNLVQDIKGTCMVFKLIMYVFMLDICLACGDCKVYAQHPLFEGGLCKECKQSFLECTYLFDEDGYQASGGMYCTICGDGQEVFMCDNEGCFRSYCGPCLEMLAGRGTVREIASREKWICYMCSGKGDRLIHRRKDWQSKLHELFLSDREKEYDTPIVYPVVAPEDRKPIRVLALFDGIATGLQALNELGIVSDKYYSAEIDEQAIQVTKVNHGDRITHLGDIKDLTESQIRELGPFDLVIGGSPCQDLSIANPARRGIFEGSGRLFFEFFRLLMHAKPSRTCPSRPFFWLFENVVGMRAEDKKTISRFLQSNPVVVDAKEVSPAHRPRYFWGNLPGMNRPAIPLPGDRLTLQECLEPNCGRKARFTKVQTITTNANSLTQTKKNILPVAVNDDGGQEREDILWCTEMERLFGFPSHYTDVNNMGRTQRQRLLGNAWSVPVVRHLLSPLKDYFKCT

>Nvec_132811

MADSSTFRVVEFYSGIGGMHYALKGCKKNAEVVAALEISTTANTVYGHNFPTTKIWNCNIEVCELCNVTTMPAIYMVMSPPCQPYTWVGLQGASKDPRALSFLHILSLLKRLQHPPKYWLIENVKGFETSDTRFYILLAFCNSFIVSSPQFGIPNSRLRYYLLAKRHPLTFSTAMGNKFSYYEQFLLPTKVLSRFSLVLDIVTAKSRRSCCFTKAYGHYAEGTGSV

>Nvec_v1g125496

PARCKECRQLIDSPDLCLFAGDPADAVEEFVALVDPRLSLFSGDEEQFDSYEDKPQHKITNFSVYDKNTHLCAFDTGLIEKNVELFISGYVKPIYDENPSPEGGVPTKILGPINEWWTAGFDGGENALIGLTTAYGEYFLMNPSKEYAPFIHTMREKIQLSKIVIEFLLNNPEARYEDLLNKVQVSVPPEGCPSFTEDSLLRHAQFLVEQIENYDSAGDTDELPLIVTPCIRDFIKLAGVTLGKRRTARKIKVKSRAELKKKQAPTKATTTELVRHIFDIFFQNQIDGKNGNAPRRKRCGVCETCQQPNCGKCSACRDMVKFGGTGKSKQCCINRRCPNMMVREADDDEGLEDEEKDDNVRDVLIRILNKSSYKNLEWLGEPAWIEGKKKYYSSVLINKKKVSLGDFVKVCPDDPHIPLYIGCVQYMWESSSGEKLFHARWMTRGAETVLGETADSAELFLCDDCDDNPLGSVLETCEVTYKDPSVDWLQGSAGMEIETSDDEHNDNTFFYHKWYHPEMGRFVDPPPEYQTKVDRTQQKFCESCIRNSAEEKVITPDISLFSRKIVTSFTLNGSEFTVSDCVYLDPEAFSFNVRPKESKPHKREGSVDEDLYPEYYRKSSEYVKGSNQNVPEPLRIGRIKSIYVKSSSGKLKGDGDDVMLTVTKFYRPENTHKGANASHQADLNLLYWTDEEAKVSALSVKGKCMVACGEDIHSSIQEYSSKGDCFYYLEAYNSQTKEFEELPPQARDCSSKGKGKGKGKGKGKGKSSADSGPESCMHDQQKEAHVRKLRSLDVFAGCGGLSEGLHQAGVAESLWAIEKEEPAAQAYRLNNPGCTVFTDDCNTLLKLAMEGEATNSTGQKIPQRGEVELLCGGPPCQGFSGMNRFNTREYSLFKNSLVVSYLSYCDFYRPRFFILENVRNFVSFKKSMVLKLTLRCLLRMGYQCTFGVLQAGCYGVPQTRRRAIIMAAAPGEELPLYPEPTHCFSPRTCQLTVMVDEKKFTSNITRTASAPYRTITVRDAMSDLPEIRNGASAAESSYEGEAISHFQRQIRGNQYQPLLRDHICKEMSALVEARMRHIPLAPGSDWRDLPNKEIRLSDGTYSKKLQYTHHDKKNEKGSSQRLRGVCSCAEGRPCEPADRQFNTLIPWCLPHTGNRHNHWAGLYGRLEWDGYFSTTITNPEPMGKQGRVLHPEQHRVVSVRECARSQGFPDTYRFYGSILDKHRQVGNAVPPPLAAAIGREIKKGLELTQGVRKKEESKMDITS

>Nvit_NV11649

MLVEANGAVGGAINTNGQAHSDDDDPKKPKEEIQDDENYVEDQQPNKRVKTGKREKQGKKETKKPVRPAEPICEICLQKLNDEDLRLYIGHPNNAVDEYSVLLDPKLCLFNGDELDITEGDARALNKVTCFSVYDKNGHLCPFDGGLIEKNINIYFSGYVKPIYDDDPSIEGGIPGKDMGPIVEWWVSGFDGGEQAIVSFSTEIGDYVLMDPSEEYAAFMISVREKSFMSKTVIEFLLDEESPSYEDLLNKLQTVAMPKGLPKFTEDILLHHAQFICDQIVSFDESATTDDPLLITTPCMRALIDLAGITFNKGKTVRKGKKRNSRRQEDDWRRGLMRKAQKEKKPSWTKATATQLVNNVFDSFFPDQLANNNTDTVALKRRRCGVCEPCQQPDCGACSACKSMIKFGGPGRSKQACVKRRCPNMEIQEANDEDPDDQEGPEPDAEPSIVAYRKKLGNLKSRTGKIEWVGKPIGTDSKGSFYSAVELDSETIEINDFVFIESVDPTVPLQIVKVIYMWENKMGIKMFHATWLWRGSETVLGETSNSSELFLVDDCQDVPVSYVKSKTTVLHRDTPDNWNELGNTSVNNSMDESKEDITSFYYQKRYDPNNARFEDPLPDLECRPEIKYRFCSACTRCTCLQQQNRPQVFDKLEEKSSKEVLYGMVKFKDEEFRVGSAVFLQPGTFKFKYPLPYQKANRTKREVDEDRFPEYYRKFNDRVKGSNFDTPEPFEIGYITSIYATTNVKLLASTSLHIKVKKLYRPENTFKSESMKGRSDLNMLYWSEEEVTLPFTCVVGKCYLTYSENLDISVEEWTVAGPNRFYFTQMYSHKEEEFDEPPSKACSIGKLIKKSDKIKSKSKKADAPKLIDIPPDFPQIKKKLRTLDVFAGCGGLSEGLHQAGVAESSWAIEVDEAAAHAYRLNNPNAAVFTGDCNAYLKKVMDGETMAGGQRLPQRGEVDLLCGGPPCQGFSGMNRFNSRAYSLFKNSLVVSYLSYCDYYRPRFFIMENVRNFVTFKKSMVLKLTLRCLIRMGYQCTFGILQAGNYGVPQTRRRMIILAAAPGEVLPKYPNPLHVFSKSACNLSVVIDNKKYFPTYDWVESAPYKVTTIRDALSDLPSIKSGKNEDVMSYGSEPLSHFQRKLRAGVSDSLLLDHICKDLGPLVEARMAHIPTVSGSDWRDLPNIVIPLSDGNHTNKLKYNYHDKKAGKSSTGALRGVCSCCTGKECNPLDRQDNTLIPWCLPHTGNRHNHWAGLYGRIEWDGFFSTTITNPEPMGKQGRVLHPDQTRVVSVRECARSQGFPDNFRFYGNIQDKHRQVGNAVPPPLAAAIGMEIRKSVSIAESSSNVKKEEN

>Nvit_NV14091

MSESTNSTTKEEKDPVEKDEAKPGGSRDDGKEKRTSNRKRKASTSDPVALADEVKAKLKVKISSKKKKGKKSSKVFSKKSIKDKSPKEKKKTGSRSKKARTEPPVEVPQDASVAVHNELLPPEVLICTTAEVHNEMNSAEIVTDANIEICSDLTVEVAQLEDTRVYEAQEPLQSIDNEDDKKEDQDFDSESCNATLAFTDAESETSRSNYEKFMIDTQNRSNEFLLGFNSKLKPELTMIDPEAKEVREDQEEAMDICKNASNCTKDDLDSEKTMIEVQDTIIDEALLDFNNKQELQLAVLATETVNANPVELNQQKDVEVGKDDDEAMDTCEDFNKDLLKENIEANENEEIDVCGDNDDAEEVHHAMELTEVNADSSNKHTDEGSKEQNKIIDSCKHEQNQESKSLKIKTLKLKTSKSSKQSSSKSSKSRDRKKSKNKSRKDSTTATTSPDPYYKVNKQDDISCRICEQTLNYQLKFYTGEPENGYDEELAIIDSRLLLFNGDEENISREDTRAYNKITSFSVYCNNGHLCPFDSGLIERDVEIYFSGYVKPIYSDDPSIENGVPGKNFGPIVEWWSTGFDIGEQPTVAISTEIGDYILMEPSDEYKPFMHSVLIKILIGKIVIEYLVYEPHATYEDLLNKLQSASSKYDILPNMTEDVLLEYAPFIYLQVMSYDETAKLNEPLMLVAPCIRRLIDLAGISFKVNRGVSSRGQFHKYQERRAGEYRKELLRKVAENRGDNSITSQLIDSLLPDVEEKLITLSKPCTRDPCGSCDRCQRSSCKECEPCSQKNYEDCPRRRCFYREIQEANDEDAKDSVNLSQLKGNSSISVFKKTINIFSSFNAKLEWLGDPLIVEGSGNTFYAAVKHEEETVCVGDFVLVRSVDQSVPAQVVKIAYMWEDNVGLAHFHANLYWWGKDTVLGELARPNELFSINKCNNISVNCITERINIDERYMPRNQDVSTDEDIKANNIFCEKSYDPSDGSFHDLTKPEEVSVLPTHRSCINCEGKHRRSERVEPRTVDELGKKEEGTLYRAVIYKDEEYIVGSCVYLKPKSLYFQFPMLLDHAKSSGKISKKIDEEKYPELYRKAEDAVIDSKFDMPAPFDIGYITEIFTTDKLYIKVKKLYRPENTHRNEVLIKQNDMNMLYWSNEVCTVSFNYVVRKCYVAHIKDFSKSVEEWSNLGPDRFYFMQMYHDKKFTDIERNMYGCEDVPLDPPIDYPDVDAMTSLEIFAGCGGLSLGLRESGVIKKLGSWAIESDVDAANTFQLNNPDITVLVGHPDIVLKNAMKGEFEDSDGKLLPRKDDVEFLCATLPCENYKTMTDITVFKHSYIATFLSYCDYYKPSLFILEADEKLIKRSFVLKLTLACLVSMGYQVTFDLMQVGCYGAPQNRRRSVLLGAAPDSVLPKFPERLHVFPKPLCELGIVIDSKKYTQDTFWTESAPYRSVTVQDALIDLPTTKVAITEEMNYDAKSTSLFSKIMRLNMLEPTVFNHVCKDLGPLVECRISQIPLAVGSDWRNLPNIEMQLKDESYAKKLIYNYDDVEAGKSSNGELRGVCSCASGERCDPRDRQHGCLIPWHLVHSARKNKHWAGIYSRLQWDGFFNGAITNPQPLGTQGPVLHPTQPRVVSVRECARAQGFPDEFVLPQKIGIHNMYRIIGRASSPFLGRAIGHEIARSLAARHRK

>Nvit_NV17734

MSSDECNESPGRSRSRSCSTATSMETDELQSLPPPPEEREDTSREADDEEESKPDADGAGPSYLRPRSHSRLLENDEAGDRQATADHSSKRSRTELATKQSRPKMMHTGRVAASRKEPVCEICRQKLQDKDLKMYAGHPNDAVDEYNAVIDEKLCLFNGEEEHVMQHDWRAINKITFFNVYCRNGHLCPFDSGLVEREAYIYLSGYVKPIYSDDSSIEGAVPSKDIGPIVEWFVTGFDGGQDAIIVLSTPLGEYYLMQPSDDYSPFMRCVKEKTFISKTVIECLLDEPNSEYEDLLNKFETIPMPSGLPRFTEELLIHHAQFICDQILSFDESALSDEPQLIHAPCVKSIIDLSGVTFKKRFRRGRRRQFLENDEDWQKGLRLSKQKAPYWSKATTTQQVHDLFESFFPDQLDNTADKLKLKRRRCGACEACLETDCGQCASCKNMIKFGGTGTSKQACVKRRCPNMQLEGDDDDYDEDDDDKANHKESSIEVHRKMIRALKKKKSAVIEWMGEPIQSAKGDFYNAVMINHDVIKKNDYIFIEPINSSVPMQVIKVKYMWENKMGIKILHGTWLWRGSETILGETSLPRELFLVDECQDVPLIYVQAKANVLVREYSNDCTEKGNIIDALKENSMILFYQKRYDHVAARFEDLLPELNPPKGAEHCFCSTCARDILSKMNRTPQLLELLDDTDDKRIKYGIIRYLNEEFRVGSAVYLKPKTFTFEFPNMNQDTSRGQKREIVDEEKYPEAYRKFNDRVKGSNVDTPEPFDIGYITSIYSTSKSKLLAGVNAYVTVKKMYRPENTHRGESLKKKSDMNMLYWSDEECDVRLNCIVGKCYIAYSENLNQSIEEWSASGPHRFYFSEAYDLNNEEYTEPPAHACSVTKSFKKDVKAKSKSKKVETIIEDIPVSLPQISHKLRTLDVFAGCGGLSEGLKQAGVAESLWAIENDTAAAHAYRLNNPKASVFTTDCNSFLEKVINGETSLGGQSLPKKGEVDLLCGGPPCQGFSGMNRFNSRAYSSFKNSLIVSFISFCDYYKPRFFLMENVRNFVSFKKSAVLKLTLSCLSRMGYQCTFGILQAGSYGIPQTRRRMILIAAAPGEILPRFPNPLHVFSKSTCQLTVDVDNVKYFTFTDVHESAPYRAITVYDALSDLPKIKSGSNEVVMRYENDPLTHFQRKMRSGMDDELALYDHICKDLGPLVEARMGLIPTKTGSDWRDLPNIVVRLSDGTCTTKLEYKYNDKKAGVSSTGAPRGICSCSEGKSCSLRDKQVNTLIPWCLPHTANRHNHWAGLYGRIEWDGFFSTTITNPEPMGKQGRVLHPEQTRVVSVRECARSQGFPDSFRFYGSIQDKHRQIGNAVPPPLAKAIGLEIRKSVSLKEANEVLDRVVVEEES

>Nvit_XP_001602026.3

MRVLELYSGIGGMHYALQESSVTGKIITSIDINTTANKVYRHNFPETANISRNIESVTVEEIAKLHIDCILMSPPCQPFTRIGLKKDSDDNRCLSLLHILQIIPHIESLDYILLENVKGFESSQARNEVILCLEKSGFNYKELILSPCQFGIPNSRHRYYLIAKRKGLKFIFDDASLITSIPEKVLELLPKNRYTSVPLEDGTHSSIKSKGKCFKLKYILESNVVENFLIPGKILLKRGSLLDIRTPESSGSCCFTKAYSHYVEGTGSVFSPSPDFEIKKKFEEINKSSETPEDKMQALLGLKLRYFTPKEVSRLMCFPENFEFPNDLSNKQKYRLLGNSINVHVVSQLIYLLYFENSVSLT

>Nvit_XP_008204446.1

MPVDGTNLGDDDLMSIHQKISHGSYWSGSLWREMMAMDLFVDHNYCKLNKSNVKIDKAENYDNFNISQENILAYKINNNELMEFDIQNYVEIQCNDEMQNDISEDVINFNSINTNTENRYFNNDATRLNCNEIAKDVNFINTLEDMNFNNISNVINNSIATENEHSMNDSMANSGSQCLINNLPLNNHNSTFTSYSTEDSGIESMDSLLFTESEDSFLLDSRDDHCYSFSKERDGAKKSYFNKRKAESSSAASISTNIENSDVIKDKSEVPAKRNRTTKRSREVIISEESPIIEGKYEESRRVTRLLVKNSDEKPVKYIDNTPGKLVWGYFRSGWWPDLNIVTALIIRAEDAGMIPSSEKIWVSWIGESRISELNAKCIDKFSNHLERRLDNLATNSKTKVTCKKQKDEACFKTIQLLKKHFTGGALVKPYIAWIKNNILPYKNKLDELHFYPYPESLSDKLNNLKIVNSEKNEKYLRQQEKERLCKAFEPKNLIKKIEKFEKPIEKGGINIVDQKYGMIVWAKMQGYSWWPCVIMDYQHLNRKQPHVAHQWVMWYGDYKYSQVQYRQILTFPTGMDRMESKITATKDELFCKAVLQAAKDYCDKLGYLTEPWKIKDVIHLFYKSKDIYKLKNAELTEPNEEDLYSQTIKKQLRKQINNQPISEERKKKILECKNLNLLLSGKLPLESLCISCLESGEELEDHPFFHASMCEKCLEDFSPKIFAYGNDAKCFYCTLCGGDDLVAVCDSMSCPRVFCTACIKYIICPEFYEDILLKHPWYCFLCDPSSISTNNVVIKIRNDWRYKMISLYRINCDEEAPSNLERLDRNRKIRVLSLFDGIGTGLVVLKHLNVNIECYYASEIDPDSMQVSFFNHGNEIIQLGDVRNIDEKKIKEIAPIDLLIGGSPCNELSLANPKRRGLDDPEGTGILFYDYVRIMKLVKKHNKKRHLFWLFENVASMPKKFRNQISKNLGREPKFLDSADFSAQHRPRLYWGNLPWGPYQVNNVVLQDVLRKRCNRQALVKKIMTVTTRTNSLNQTKENLKPVLMDGKKDMLWVTELEKIFGFPMHYTDTNLQKTRRLQLIGKAWSVQTLTAILRPVFLF

>Obim_22034501m

MPSRTSSISKTTISSPKLRESNKIVKDLQNKFQTGDITEVDYWNQLCEKFSHVLTGAAKDQIQTLETELGEELLTIKGFCKKMEDILQPLVSNTLPKVPSLKPVLVKPEVVTKDDTTPASKRSVENKNGINDSAENDEEVEPGLSPSKMGKMDEKEHNCEKKESTSVATDAITNNVQSSPPTTRRNGQSSNGRNENGVENGLNFTNGHSTEDGVKFPEAVPSSEPTGNDKNPPSTPESRKSKSKRTRSSGIQPAITSMFERIPEKRKAADEDEESSGVKQEEKKIKTSEINDKDDENKEASSVQNTDTKLAKEQVTPIKCRDCKQLLDDPELVLFHGDPPDAVEEFIALTDPKLSVFSGNDDEVSSFDERPQHKLSNFSIYDKATHLCAFDTGLIEKNVELYFSGVVKPIYDENPSADGGVPCKTMGPINEWWTAGFDGGENSLIGFTTAYADYILMSPSKIYSPFMDIMREKIYLSKIAIEFLQSNLDATYEDLLNKIQITVPPNGITNIPTEDALLRHAQFVVDQVQSYDEAADDDEALLITTPCMRALIKLSGVTLGKRRALRKQLREPKVKSKDTQTMAATTSLVRNIFDSMFQGQIDDNGFSATRRKRCGICEVCQQPDCGKCKACRDMVKFGGSGRSKQCCVQRRCPNIAVAEADEDEIQSDDDENIVKKGDDSEKPIRHVASRNTKTKLEWVGEPVKTTPTNIYYNAVNINGKEVCVGDCVAVKPANPTVPMYIAKVAYMWEDKFKEKKFHALWFCRGSDTILGETADPFELFLVDDCEDSCLEYTISKVNVVYKSIPSNWAMLGGTDEDTNFNNNDSKTFYYQKWYDPELARFEDIPKDPNPSEINSYKFCSSCSRLETLRKKETPCLGEQLENGSKESSRLYYKSVKRDSIEYKLGDCCYVEPSAFSFNVKHVPQKKPKTEKREFDDDAYTEAYRKSSDYIKGSNEYVPQPFHIGRITEIFCRKVNNSRLMDLTDIKIKINKFYRPENTHKGPAASYQADLNVLYWSNEEVTVDFKTVSGKCTVVYSENLKVDKDTYFMGAVDRFYFTEAYNREKRSFEDPPNVACLMGDKGKGKGKGKTKSKSKCLPDEIEPTVTPLAALDVFAGCGGLSEGLYQAGIAKSHWAIEKMEMAAQAFRLNNAGCTVFTDDCNLLLQLAMKGIEKTSKGQKLPKKGEVELLCGGPPCQGFSGMNRFNYRDYSKFKNSLIASYLSYCDFYRPRFFVLENVRNFVSFKKSMVLKLTLRCLTQMGYQCTFGILQAGNYGVAQTRRRAIILAAAPGEKLPFYPDPLHVFAPRGMQLSVVVDDKKYNSTITRTGSAPFRTITVRDTMSDLPEIRNGAKEEEIQYNGEPYSHFQRLIRGKQHQPILRDHICKEMSPLVHARMQHIPLCPGADWRDLPNVVVKLSDGNKTKKLHYSHHDKRNGKGPNNQLRGVCACALDGKCDPMDRQFNTLIPWCLPHTGNRHNHWAGLYGRVEWDGFFSTTVTNPEPMGKQGRVLHSEQHRVVSVRECARSQGFPDTYRFFGSILDKHRQVGNAVPPPMSRQIGFEIKKSLLWKLQNSAEQKPEDQSKREN

>Obim_XP_014770812.1

MDNTAISTSPPVANTTDILTATALTDINITATTVSINNIETFEDSDEDVKLFDLKRKLHEGCKSNHTTDTKYLETNGLLLEFHSKAKKRRGRKPKMVHPKSCDSEELKEEIEQCLEKKKTKSCKRKLHSYQIPLSPQKDLGNKCFPVGSVIWGKLSGFKPWPGLVINHEDIQKKSSPLTVWVLWFGDYKISELPLSKVTRFDQYFYLNYKNPSKVFSVAVTEALEVCAQRSGLKEDELSSEHLFNWAKHRFMEMVEDKDPFLPLKGDPYPQFVKRKLQPIFKTAENIEEGPVRVSSPPNPLFENNDLINKLKTNQVKIQEICIGCKSSDKTIVAQHPLFEGGLCEQCKENILETIFALDVDNTHAFCSICSQSGQLCICSVPECNRVYCINCIQSWVGCDAWDLILSQNPWSCFLCRTPNKRRACGYLQAKPNWSLNIMHMFNSHQKLAEPNLSYLTKCPERPPIRVLSLFDGIGTGKVALDQLGLTIKEYFSSEVDADALIVKRFTYGQSIKEIGDITELTEVKLSELCPLDLVIGGSPCNDLSLVNPARKGFSEEGTGILFFEFCRILSIVQRLCKDRHVFWLYENVAAMKTSYKNIISRFLGCKPALWDAKFFSAQHRPRYFWGNLPTIYSSQPTTEDDSLYLDKILSPNCNRKARVKQLRTVTTRSNSLRQGKNENILPVEMDGEEDAIWITELEKIFGFPTHYTDVRNLNHSRRQRLLGRAWSVPVIKFLLSPLTKLFHTAT

>Obim_XP_014773951.1

MPFASQKIMAITFSADETTLAFFEAESNIPHEVVAAIDINPVANKIYSYNFPHTNLLETGIEGLTADWLDHLDFNVVLMSPPCQPFTRVGKRLDTKDSRTRSFLHFLDCLQKMKCPPSFILLENVKGFEVSETRHLFLKTLKACNYKYQEFIFTPLQFGIPNSRARYYLIAKKHPGKFYFDLTDEVQEELPACSKQWLHHIEPSEDCGETTCLSQDKDINCLQTCKTEKEEIRDFLDYYNKYSTILHQEEFENGISYPNCKALNDFLETNQSKEYYDQFLVPEKYFSLFVILDIVCPHLRKSVCFTKRYGHYIEGSGSIIQMSTDGQSVKDAYDLKMKTQQLKNRKQWSEEEFNVVRKLKLRFFTPREIANLLCFPHTFDFPPETTLRQKYRVLGNSLNVHVVAVLIKLLSQDCNLML

>Ocar_m.104173

MLYKDAVMTALEIAKRRCGKTFDVSDNEGTETEETETEDTPVNVSAASASATQDNSPSATQDNPPSVSQDSSPSPEKTQITSPPPPVAKEPLPQTQTQKPKSKSKKTKRLKPIRLDDPVEQQLVDWALAGFPPTGPDGFQPSKNEVDGGSSGVAEAEAVVYKEKSVRESSPGQTVKGSGSKLKGAKDLGGSQMENELDKRASCVSKEERERFSDVRNGSRKIESLCLGCASLNPMAEHPLFDGGLCETCKECFEENCYLFDDDGTQMYCAICCDGREVFLCDVMGCSRAYCTFCIEKICGSEEVDKITSDSEWRCFMCEPTKSPHPLKLREDWQKRMQELFSSEQLECDPIPVPQPMSLGERRGIRVLSLFDGISTGYVALKSLGFVIESYVASEIDFDAIKVSWKHHHLPEINHIGDVKKISKKELEKHGPFDLVFGGSPCNDLSVANPNRKGIYEGTGRLFFEYYRILKDMPPPFFWFFENVVSMRSEDRQIISRFLQCNPVVINAIEVSPASRPRYFWGNLPGMSRSLVPLPGDRLTLQDCLEPKRSAQFTKLRTVTTKANSIKQTRDALFPVAQLTPDGSEKGDGLWCTELERLFGFPDHYTDVGSMNKMNRQRLLGQAWSVPVIRHLLSPLKDYFKCNTEPIACSQPTV

>Ocar_m.109426

MEETLQQEKGAEEKPPEAESTQEASSDPAITGSSEATVKSTKKRKRAVSGSGAKKKAKDGDSPWVVESATKKQTPSEKCKVCGQYLESPDLRLFSGDPEEALDEFAVLAHEQLSLYTGEEEGIGVDEVPQNKITDFSIYDKHFHLCPFDTGLIDKNKELFISGFVKPIYDDNPLPEGGIPAHALGPIGSWWFAGYDGGERGLLGVTTVYGEYILMNPSEQYSPIMENVLAKMYLNKIAIEYLTDHLDATYEDLLNKIQTTPPPQSLSVSSFDEDVLQEHALFLVDQVESYDDLADVDQELLLSSPCMKHLIELSSAELSKRKKKVAQRRGEEPIRRKRKPQSKATTTPLVGNVFDVFFKGEMEEKGKQKVSSSAPRRRRCGVCEACQQPECGKCSACHDMVKFGGTGRSKQACVQRRCPNMAVEEGFESQEEDPEVDPKRLTARQSSRTKKGGRRQEVATSWGEPLISMKGITYYRSVTVDNQEVSINDFVFVQPVNTHVPLYIAQVQYMWQNKRGEKKFHCIWLTRGTETIIGNTADSGELFFADVCSTNPLSSIRSKVEVTYKEVPERWAELGGMEVAEMHVDEDRVSFFYQKWYDSDCARFEDLPEEYRKIGEIDRVKEPDRCPSCKRNAQKAEYETAQAEERVGEDEDRILYRSFRLFGETFSVNDCSYMMPDAFKFSVRPASSKKTLKRPTDYDEDLYPEMYRRTSDYIKGSAEGVAEPFRIGQIKDIFTKKSHVRKKEEEEEESEEEEESDKEGNSKVFIKITKFYRPENTHLGITASYIANWNHLYWTDEGLTNTNRHFIDTIAASLSLSLSEATVNCSLIEGKCTVVYRDPMAREEVDWEPNTFFFSESYDPTAKSFGTPPAHAFPMRKGKGKGKAKVVASSVTNDPTVPAPVPTTSESKFRPLRSFDVFAGCGGLSLGFHQSELAETRWAVEVDEPAVQSFRKNNPKAAVFVEDCNKVLQWVLEGRKVDDLGQNLPQKGDVELLCGGPPCQGFSGMNRFNSGSYSRFKNSLVVSLLSYCDYYRPRYLVLENVRNFVTFKRNMVLKLTMCCLLRMGYQCTIGILQAGSYGVAQTRRRAFLLAAAPGETLPLYPEPLHVFSPRACRVSISVDDTKYVSNVRWVSSCPFRTITVRDTMSDLPTIFNGASAREISYRGEPESHFQRKIRGDQTVLMDHVCKKMNSLVEARMRRIPIHPGADWRNLPNEEFKIGSGGDALFVKKLEYRHRDTKNGLSATGALRGVCTCASGKPCEATERQFSTLIPWCLPHTANRHNQWAGLYGRLDWDGYFSTTITNPEPMGKQGRVLHPKEHRVVGVRECARSQGFPDWFRFYGNILDCHRQVGNAVPPPLAAAIALEIKKAASQTKK

>Ocar_m.89599

MQEQEQKPCALRVVEFYSGIGGMHFALEESGLSHEVVACLEINDVANAVYRHNFPGTPIMQRNIESLSVGEISKLNADVFLMSPPCQPFTRVGLKGDVSDARTGSFLHILDLIEKLDRKPGLLLVENVKGFDTSHTRERLIETLENSSYKYQEFLLNPPQFGIPNSRLRYYLVAIHSASSYTFSFKVSKQILTDFSVETVPVEGREIETYLEKSQSDDYFEAFLLHPSVLNRFGMILDIVTSKSRRSCCFTKAYSHYVEGTGSVLQMAESVSMDSVYSKFKAEAGCGDSEKSVLSSLKLRFFSPREVANLLSFPPRFRFPDSISSRQKYRLLGNSLNVHVVMKLLLLCCSKNAFHT

>Pbac_3469897

MFPPDYTICTHPKPRSPPHNPWFQHETSPPSLSELLNGKSKHFSYVQRQEGHVFWLFENVTSMSADDRTTISRFLQCSPLKIDAKHFTCMNRSRLFWGNIPELKRPIVPVANPVTLQECLETRCGRSARVEKINCVTSRKRTETAPAPVLMEGQTDGLWTTEYLTPTPFLVGESIWAGTALYGRRKHVKYKPIEAPGKELECPCYEAYSGTSGTIFPD

>Pbac_AFV53350.1

MVRNDAIENQENLPPNSKRRKTDKVENLKEPPRKQVVKPLKCGVCRQLQDKDVLKTFPGDPPGAVEEFIALASDILSLGQTEEGDWEEKPQHRITEFSVYDEFGHLCAFDTGLIDNNVPLYFSGYIKPIYDDTPSTEECGVWASRCGPIDAWYVTGFDGGERELIGFTTVYAEYLLLHPGPEYRSIYDGVREKSFLSKTVIEILEGEPEQSLEDLIQRVQQVIPPFGSSRTGGLTEEDLLKNAQFLLEQIESYEMAADEDEDQLKMTLPAVTGLMSLAGADQIDRKEPKTKRRNKTTGNKPSGPRKTKSTDTSHATVTPLVQATFEGLFFDQLSHADTVEADGGPRKRCGVCVGCKAEDCGTCPTCKDKVKFGGSGKRRQACINRGCVDMQPKEEKDKPVKDSKSRTKTVRSKMQISGDTDGEFTGEVLKTKGGKTYYSTATVEGYSIQLGDYVTLQAEDDSGQEGQWVARIVAMFKRGNVETLHVQWFSSSSDTVLGDCSHDPSEIFITDTCDNVPLGSVTSKLDDISYRPPPDNWNEIGGLPVAKKEENTTLFYRLKYEHSKGRFEVPPPEYLNWKPTEPCPVCTRSKLHDPAPRIEDGVVVAGGERVKVGGYLYLSLDAYTMPNRKRKMASRARKDSVDEDMYPEFYRKTDYIKGNNADVPDPFRVCRLVAIDNQNTVTVRKLYRPENLDKGVCCAEESDINLLYYSAEMVSVSVDKIVGSCTVRHGADIPDLAKYSSRTDHFYFTEQWNAKEKVLEDPPGDVRLSNKGPAKQKVVPEIKPRKLRTLDVFAGCGGLSCGFHQAGVAESKWAIEFVSEAAAAYKLNNPKTTVFNEDCNKILKMAMEGREVDDLGQRIPTKGDVELLCGGPPCQGFSGMNRFNAREYSQFKNSLIASYLSYCEFYRPRFFLLENVRNFVSYKRNMVLKLCISSLVNMGYQCTFGVLQAGSYGVAQTRRRAFILAAAPGEILPQFPEPRHVFSHAGMSLSLTVDGKRFGSHITRFTSAPLRTITVRDTMSDLPAVGNGASVTELAYNSEPTTWFQRQIRGNTTTLTDHICKEMHPLAAIRTKHIPLTPGSDWRDLPNIAVNLPDGTRAPKLIYTHDDRKNGPLRGVCSCAEGRSCDPSDKQNNTLIPWCLPHTGNRHNHWAGLYGRAFWDGFFSTTITNPEPMGKQGRVLHPEQHRLVSVRECARSQGFPDSHTFYGNVLDKHRQVGNAVPPPLAKAIGEEILKYAFRFEG

>Pdum_DNMT1_comp223422_c0_seq1

MPVRIDTSPDLSSEVHSRLEELDQELKEDEITEKGYWKRKYSLLEPYLNKSSLEKLKSLKEELKSEEITEKGYQRNLEKILESTVEGLKSEKSENGHVNGKENGTNGHHENGDNGKETEEESSNDVKVKKEPESESMEVDDSQPGSSSSKNGNGSKSEDSQEEGDSQEETDSQDEGGVKKKKGKRKSKSKGGQENGASSGKKTPGRKSKSDSQPSIMSMFSQATNKRKSEGVSQESAQEEKRIKLEEDVKEEVKEEKADTPAEESADVKKEEKKPALKLDKEPPARCKECRQRLDDPDLKMFSGDPADAVEEFIMLTDPKLSLFTGDEADINDGDERPQNKITMFTVYDNNGHMTSFDNGLLEKNKELFFSGYAKPIYDENPSAEDGIATKKMGPINEWWTAGFDGGEKALVGFSTAFSDYILMQPSEAYAPYFNTVTEKIHLTKIVIEFLSEDQDANYEDLLNKIQTTVPPKGISSFSEDSLLRHAQWIVDQVENYDEAADEDETLLITTPCMRALISLAGVTLGKRRAMRKENQKRIPKVKKPAFCMATVSPLVMSLFEKLFEGQIANKSSAPRRQRCGICEVCQQADCGQCRACLDMIKFGGSGRSKQACIYRRCPNIAVKEAEENAGEEEDTNEEAEKLIMDKKEKLKEAAMKRHNFSMQKVTVEWIGEPIKEKNNRKYYSAVNFNSEEVRKGDFLSVKPADNTSPLYIGKVMYLFENKKGDKMFHAHWFNRGSDTILGEVADDHELFMVDDCQDTQLNFCVDKVKVIHKEPGEDWYMQGGKEPENIIEEDDGKTYYYQKWYDVQMARFEDLPEVPEPENESEKLSFCTACFRVKEKELRETPQLFEEVESEKGSALHYGLLTYEGEEYRVGDCVYLDPESFTFKIKPAPAPKIKPDKKREHDDDTYPELYRKGTNIKGSNEFVPSPFCIGQLKDIFIKRAVSGDLEPEQVCLKVRKFYRPENTHKGIMSAYQADLNILYWSDEETTVNLAQVEGKCMVVYGEMLDVSIEEYFKSGANRFYFTEMYDAENKTFSEPPSEATKMSTSKKGKGKGKGKGKGGKGKGDSGDSQDQKKDLPKLRCLDVFSGCGGLSEGFHQSGIAESHWAIEKEEPAAQAYRLNNPSATVFTEDCNELLRLAMQGEKKNSSGQRLPQKGEVELLCGGPPCQGFSGMNRFNSREYSQFKNSLISTYLSYCDFYRPKFFLLENVRNFVSYKKGMVLKLALGALVRMGYQCTFGVLQAGSYGVPQTRRRAIILAAAPGEKLPFYPEPIHTFSPKGMQLTATIDDVKFESCVKHMHSAPFRTISVRDAMSDLPEIRNGAKNDEISYNGDPQSHFQRAIRGHQYQPILRDHICKDMSSLVHSRMQHIPLAPGSDWRDLPNIEVRLADGTRSKKLLYEHKDHKNGRSPSGNLRGVCSCADGKSACDPLDRQFNTLIPWCLPHTGNRHNNWAGLYGRLEWDGFFSTTVTNPEPMGKQGRVLHPEQHRVVSVRECARSQGFPDTYKFFGTILDKHRQVGNAVPPPMSRAIGEEIKKSLQWKQQKGNQKKMETE

>Pdum_DNMT2

MEKLRVLELYSGIGGMHFALKECGIPHEVVAAADINNVANDIYKFNFKGTKLLQRTIESLSLKELNKLQMDMITMSPPCQPFTRVGLQKDAGDQRTKSFFHVLDLLPKLDKQPKYILLENVKGFDTSATREVLLETLNEAGYHMQEFLVTPLQFGVPNSRLRYYLLAKRKELAFSFPMSDEIITDLPACAEQFLHQKVKDSDIEDVPDDGRWDLSAFCTCMDQSSSGEICAKCMKLRYVSKKRKTEMDNETEKSRLAAKEEEENDVGPRNLSKDSPNDQSSAKKVDSDSSSPTRNESSSLTKESCADDRINSLHPTKKTDAGDSIDDSCSSVNKSHDLNFQCSRNENDSNSHHGHVSNSDCGSSLSPNNSEELTGAQGGQSHTKMDVGVQSNQSNCAELAQTEGAQKVRTLTPSEIAYVKYRSICRPVSDYLETQPEEYFKDYLVPEKFFKRFWVMDIVKPHARNSCCFTKRYAHHLEGSGSIIQMDLDNSVDLRAYKIERQYNETVAEGVRSMKLRYFTPREISNIMCFPPWFDFPEQFNLIQKYRVLGNSLNVHVVAVLMRLLMLGDDES

>Pdum_DNMT3_prot

IETTIGSVENPQKQASPFHSCPVFCRSSYWRLHLLLKICPLQRSVWPYCSPSSSSDPIKAISDWCILPEGVLELIIFQICKMAAMEIVRDEGAQQKIRVLSMFDGIGTGRLALKTLQFDVGAYYSCETDVDANLVNMIRNPDIIPIGDVREINEKVLKCISPIDLVIGGSPCDEFSIANPWSLGFILGRTGSLFYDFVRVLYKVLEMNKDRRVYWLFENVRSMSEEHRSIMSFFFFDQLPTIWDASVLTPQNRKRYFWGNIPGLKGGWESCRIKSPVVNDVLCPFRKSHVKKVNCLTTNVQSQMEHSGTTFVKPVRTITDEESQFWSVERERLFGFPDHYTDVAGISASSRHDMLGQAWSLPVVTTILSPLKGRFKMLPKTD

>Pmar_ENSPMAT00000007628

MEGPPLLKRKLVDSYVYVDRKRPLLPKKAKPPPIKAKQIKLAGLRDQHIYEVRRKNRNIEDVCIACGSLDVITHHPLFEGGMCQPCKSTFMECAFQYDDDGYQAYCSVCYGGGEVLMCGNSNCCRWGSVECVEMLVSVGAAKSAIAEEPWSCFMCRPKGAHGMLRRRDDWASKLQNLFTNAHSQEYPIPKIYPPILTSQRKAIRVLSLFDGIATGLLVLKDLGIKLERYVASEICEDSIVVGTVRHEGKITYVGDIRNLTRKHILEWGPFDLVIGGSPCNDLSIVNPARKGLYAEGTGRLFFEFYRLLHEAKPKEGEDRPFFWLFENVAAMGVNDKRDISRFLECNPVMIDAKDVSAAHRARYFWGNLPGMNRIFGFPVHYTDVSNMSRLARQRLLGRSWSVPVIRHLFSPLKDYFSCV

>Pmar_ENSPMAT00000008525

RGFAVGELVWGKIRGFSWWPGVLVSWEDTGQCCAAEDTRWVCWFGDCKFSLVAVDKLMPFSHFKENFNQQTYAKKLVYRKAIYQALQVASQRVNKVFPQPEESDTEPGLSLEERISPMMHWAMSGFPPKGAKGLVPLPEQEISSTFFDKYYDRILWGSKSYLAFSPQRSWSRASASCRRSTSSLRRRRVETWVVVDECWREQIIQEVQEDIRTIEGCSLECFEICVHAQHPLFEGGICQRCQITFMECAYQYDDDGYQSYCSICCGGREVLMCGNINCFRLAGCRCFCVECVDLLVGSGTAKTAIEEEPWRCFMCRPSLATCGLLNPRPDWPARLQHLFSNTHSQEFEPPKLYPPITPDHRRPIKVLSLFDGIATGLLVLKELGIRVERYVASEVCQDSINVGTVRHAGDITYIGDIRNLTYSNLQKWGPFDLVIGGSPCSDLSIVNPARKGLYEGTGRLFFEFYRLMNEAKPREGEERIFFWLFENVAAMGNNDKRDISRFLECNPVMIDAKDISAAHRARYFWGNLPGMNRPLAVTAVDKLELQDCLEHGRTARFSKIRTITTRSNSIKQGKDQHFPVMMNGREDILWSTEMER

>Pmar_GENSCAN00000023487+GENSCAN00000022101

MAEASSRPSSRRAAPQTSIRESPRFDYRPRLTRPAPHPLISNARFAVQGYMKKKLGLVQPFLSGEVKTQLETLQEELKEEDITEKGYLNKVKTLLLRELPKLEEEGSEKPHQNGAAKNGKVNHGKNGTTHHAATAAAATTATACKTESVEANGDEAATENGDAHPAMANGSPECSGKAVANGDGLVFVKSDAEMDDGEVEGDDAEPTSSSSSSSSAASAGKTRGRKKKQAAEAESSGAELASPRGRGGQRGRKSVGAAGGSQQTIASMFGKGLVSHAHIMNIMIQDRAAYRVREALAGTPPPKCTECKQFLDDRDLKLFQGDPDDALDEPEMLTDERLSVFESNSDAMESYDDLPQHKVTNFRPLIAENCRLIVFAFSLIYARDLVMSECSVYDRKLHLCAFDTGLVEKNVELYLSGVVKPIYDENPCNDGGVSAKKLGPINSWWTTGFDGGEKALVGITTAFADYILMNPSEEYASTFAVMQEKIYMFKIVIEFLQHNRDATYEDLLNKIETTVPPCGLSFTRFTEDSLLRHAQFIVEQVESYDEAGDEDEPPIIITPCMRDLIKLAGVTLGKRRAQRRQTIKHPTKIAKDAKGPSKATTTLLVRQIFDEFFDEQIEQRDKENVIKRRRCGVCEVCQQQDCGKCSTCKVMPKFGGIGRFKQACMNRKCPNMAVKEAEEDENFEESDSEEVNKIHQKKVTDIKKKKKSKSQISWIGEPQKTETERKYFQRVRLDDEELGVGDCVVVCPDDPSKPLFPARITSMWEEENGEKWFHPHWFCRGPDTVLGETSDPLELFLVDECEDMLLSYVYGKVSVTYKAPSPNWFMEGGMETEEEKTSKDDEEKSFFYQLWYDPEYARFESPPEVTPAEENKFKFCASCVRLSELTQRETPCVETRLDDPEDDAIVTYMLALKDGVEYRVGDGVYLMPDAFNFSVKPASPVKRAPRKEDIDEEVHPEYYRKSSDYIKGSNLDAPEPYRVGRISQIFCNKRNNRETDIKFKINKFYPENTHKGMSAAFHGDVNLLYWSDEETVVEFRNVQGKCRVEYGEDLPESVAEYSQGGPDRFYFLEAYNAKTKSFEDVPYNARSAGCRGKGKGKGKGKGKGKVSVEHSVEKEPEEKVRKLRGLDVFSGCGGLSEGFHQAGITETKWAIEMWEPAAQAFRLNNPGSTVFTEDCNILLKLVMSGEKTNGLGQRLPQKGDVELLCGGPPCQGFSGMNRFNSRTYSMFKNSLVVSYLSGVAIGSIIKPELKVLRLNGHPGWAVRCGADTAQGDNPRSRPGREAAVLPRAPACVRPARLPAQRRGRRQEIRQQRHQVRNARMRVSSAPFRTITVRDTMSDLPEIRNGASALEISYNSEPQSWFQRQIRGMQYQPILRDHICKASTHTLTFSKTGGNPDLSPLVEARMRYIPLAPGSDWRDLPNIEVRLSDGTTSKILRYTHHDRKNGRGSAMLRGVCSCSEAGKPCDSADRQFNTLIPWCLPHTGNRHNHWAGLYGRLEWDGFFSTTVTNPEPMGKQASAMVLHLYLNCISLATHFPCAFTPGRLWLRFIRTLAVVLCAVLDVECLALATIWNLDVYEELTLKIFLHRLDIQNVPQGRVLHPEQHRVVSVRECARSQGFPDTYRLFGNTLDKHRQVGNAVPPPLAKAIGLEIKKCVEQSRKNELAKNVNNSHALGYWM

>Ptep_aug3.g9131.t1

MRGDIVWAKMGGFPWWPAIVIDPKDCGRDDDNNEEKLWLFWFGDYKVSQMPLDKINDFKEEYDTHFLNGKGKNFNRAVQEALTVLAENYNENSLKSSNLFDWAAKGFENQGLFLPDKVKGLPKMVSESLNRKAFKRKLINDSDQEDDSDEDTMEMENLLPDVLNGIKNLEDICISCFKPSACVVKKHPYFVGGFCRTCQRIVEIACDIPFGELQDSCAVCGQGGHLIICSNSMCLKSFCSLCIEYMTPKGTFKKVLRATIWLCYLCELDSKDKCLIRPRRLVTSHQNVVIPILRKKNLINVLAHSDTILSVLKSKRFAIGKFSTEIPPLDSSKFVKFNKFVEDELENLSPDFVCCSYLEDPLNLEFPNGDVHLLFKTFYSFLYTLDEAKAVNERVLFAFLLNAKKSNYLVRERLSHLLEVPPVALTTKRKSLYLWSNILNCSCLSKVFQEQKFADVSSESDFFAPVIELLSKYFKCVK

>Ptep_XP_015906236.1

MCIACRKEDAEVKHPFFKGGLCIACKENWMQTIFAIGDDGKNIVRLTKRGAIPSAEEQNCDGLFSDYPPDNHHLLKESSTVCHKQEDSYCLSCIDVFLGDKRRKKILETSPWLCFFCSKIPGTFEKRENWEEELHFMFESSASSKTCYPLKNTPIRPIRVLSLFDGIGTGKLVLDNLGIDVEVYYASEIDEAALCVSKLQHPDAIEYIGDVTKLLSKKISSMCPIDLLIGGSPCNDLSLVNPLRKGLFDVTGTGCLFFDYYHILKTVKRFNKDRHLFYLFENVAAMTKVNKIKTVTTKKNSLSDMPVEMKGIPDILWITELERVFGFPMHYTDVGNLSPTKRQELLGRSWSVPVITQILNPLQKLFKKSSV

>Ptep_XP_021000750.1

MKIKTEPLSPSKSDLPAISDEETEEKVKKQEEGVDNGNCSSESSDKRTTVKVAQSSRKSKKETGQQPSISSFFTKMQNKRKLEEDENSNSNGQSKKSKSNGKVEDDDKRKVEKDENSNGQSKKSKSSDKVSNGQKSSLLPMRCPDCRQHLDGPDLKLYPGDPDEAQEEFIMLTAPILSLHTEGDYDTVDVNERVQHRITNFSVYDKHTHLVPFDQGLIEKNVELFISGYVKPIFVEDPSDTHGGVLARNLGPINEWYVSGFDGGSKALIGLGTDFAEYILLNPSELYAPYMNAVQEKIYLSKCVIECLIENNEASYEDLLNKLENTVPPQGISTFSEDSLLRHAQFIVDQIESYDSAANEYDPILIVSPAIRALIELSGITLGKQHRIKIPTQGRIAKAKKPVHTLATTTPLVHSIFEMFFTGQLEDDKKNSAPRRKRCGVCEACQLPDCGKCNFCRDMVKFGGSGRGKQACALRRCPNMAVQEANEDDILTDNLEDEMDKKATSTVNGTSSRKGTSVTSKFDVSWIGKSSFEAESRKYYFEAKINDFTLKLGDFVFVTPSEPSTPMFIGKVLSMFETPSGKKKCHAMWFERGTDTVLGETSDPHEIFGTSECGDIGLATIRNKCNVIFKAPAPNWHELGGTEDSLFDTVELVDESSFFYQKWYDADTCRFEDPPLYHNSMPSCDENKTICFGCIKYENAEELATVSFIQYDEDKDTKYYKSGRWLGQTIQVGDCVYINPDVFSFPKKKVVKTENTESEKEVDETVYPEAYRKSDYVKGSNLDVPEPFKIGFITKLFQKKRGDPKVTVKAFYRPENTSRSESLNHSADFNLLFWSDEEYTFEFTNIKGKCYVVFSENLTESVDDYFKGGPHRFYFNEAYDSSTGTFDEPPSIAQRIGRVGKGKGNIKGKKTTSNEKVESKPEEYPVINEKLKTLDVFSGCGGLSEGFHEAGVSDTRWAIEKEEPAAQAFRLNFPECSVFTEDCNLLLRLVMDGQETTYKGQNLPQKGEVELLCGGPPCQGFSGMNRFNSRQYSLFKNSLIVSYLSYCDYYRPRFFLLENVRNFVSFKRSMILKLALSTLVRMGYQCTFAVLQAGQYGVPQTRRRAIILAAAPGEKLPSFPEPQHVFSPRATQLTVVVDDKKYRPNIKWTSSAPYRTVTVGDSMSDLPSIKNGAKAEEISYSGDPVTHFQKKLRGQTYQPVLRDHICKEMNPLVEARMRHIPTVPGADWRDLPNISVRLSDGSNTKILNYTHQDIKNGKSSTGALRGVCACASGKACDPMDRQFGTLIPWCLPHTGNRHNNWSGLYGRLEWDGFFSTTITNPEPMGKQGRVLHPNENRVVSVRECARSQGFPDSYRFFGTILDRHRQIGNAVPPPLAAAIGLEIKKCLAYKESIKQSSDCE

>Ptep_XP_021002349.1

MSSTKRKNNESLLSQQAISATTIPIGCVVWVVSESSGDKEWWPAITLRFTDCYQKPPEEDHTWIFWFADYKISKVSVQDIIEFTAGYTDLIGRVINETHLKALNEALQILAQRSNLDYQNSEELTNWAKSDFEGIIRESNDLPDMVLKKLERKDLGKEFSDDNKSEGNSGVPIFEKIENGLIDIEKMCIACRKEDAEVKHPFFKGGLCIACKENWMQTIFAIGDDGV

>Ptep_XP_021003330.1

MLLMNYRFFLMDVSKCSQHITSCDKQNHCSNYCYRVLELYSGIGGMRYALEASQLLFDVVAAVDMNPVVNEIYRHNFGIKGHYQRNLKSFTVKEIDDMKIDIITMSPPCQPFTRVGLKKDIEDARSDSFLHIMKILVQLQRKPSYIILENVKGFEDSEARNVLIKTLEQCNYRYQEFLLSPIGFGVPNSRLRYYMTAKLESQPITTTDVKIFNIIPHSNYNWKSHCLKCIHSTYNMCSLKHHLEENSPDYFKPYLVPDHVLLKHWTVLDIVDETSNTCCCFTKAYSHYAQGTGSIFVPITDSLKVTEIFKQVACVDDNNSKLELLRNLGLRYFTPKEISNIMCFPDSFNFPSSITLRQKYKSLGNSLNVFVVTCLIQSLLKDEIFSKNCDNENEATEKE

>Scil_006106_1

MQQSTAAPDVAKLQAAGCGQEPKHVPLVVSHVATSSPPPPVLASWDAAAPGASATTPATTEVAVSEKVAATIVPDECVTPAAADGCNEKPGWSWEERAAQLAVVDSSGEIHTGKPPPTPLSDQLSDHSPEVESCLLLAEVAERIRTGRAVQVTESKRHDLLPGHNSRSISCQVPNRPPDSFSPYGEAAAAGTAAQFGRHAEAAEHCELGSLPTRPVRIRKAPRTYISEMEKMDDGPGASKRRKRAIAESDAGGGGTVMNVEQPCSHAQVLLDSSYQKNADNGAPSVSAGAGHTSHSSPSSSTTTSTSTTASPVGCKGKAATDRRRSQASGLSHAAETQAQAIYSSNGVSQAELANSAGWGGNSSSNVDSDLQALHGRLTRSRLSKTRVDCEATASNDSANHGKPGADSSDDEDHGSSSTDRNAQTGGTPKKSTANPTGSERPRPHKRKWRNQYGSSEDATSDIGQARSASSTPALSTMDVTPVAANERPDIDLGIGDFVWGKMRGFDWWPGSIVSAGRANQVSTTPGHTWVCWFGDNQFSQILLTRIRPLTDFRQYLLRRKPKSLYRRAIIECADSAALRANKQFPSEWPGTATVSTEDQSVLDNFCDTPRQRHVLHWALGEFVPEGLDGLRPSELDINNAKRSASGQTLPSSPDGPLSPPYSTSPIGSSPLPSTPRSSTAASRSLSNSVMVTPRQPQQMVQSDTRGSSGQQLERFALLRQGKVSLSNCCLCCGASGECVQQEHPLFAGGICDACVPAFIECAYCFDEDGSQTYCCICADGKEVYLCDSPGCARSYCPLCIEKLCGADIHRHINKLDSWMCFMCDDQPSDQLEVRDDWHARLLTLMSSEDSGLYQLEPTPPPPAPEDRQAIRVLGLFDGIATGMIVLNELGIEVAAYTSSEIDADAVRVSRRHHTNIVHVGAVEEITSQRLKEWGPFDLVIGGSPCNDLSMANPARRGIFEGTGRLFFDFFRLLQLAKPAPSEQRPFFWLFENVVSMRAVDKATISRFLECNPSVVDAKDVSPAHRPRYFWGNLPGMMRGIVPWPGDVLSLQDCVEPNCGRRAKFEKLRTITTKTNSIRQTKQNLFPVSVERRGINGSLEGEDGDMLWCTEMERIFGFPAHYTDVGNMGRVARQRLLGRAWSTPVIRHLLAPLRDYFKTTGKQLPVQQK

>Scil_17007_1

MLKGQTRSAPSGSAECTAAQLKAAEADFKDGCLTEKGFVKKKWNLLESHLGPAQKKRLSQLQSDFDGGDLGESSFLTTAKEVLGQAEETVFGGATSSKAAEAETEEAPAKLLPTKRTSDASSPEASPAAKRTRVRSRGRAKSSTSTPTRQSSRRRRVVDNSQSGSSSQDDSTVPEADDSVPPAAEVKEALSPIEEKMDTSDSPVVVEKDSKPQVVDSEVTQADTEKPDADTLKPEAADDSDTRSQASQDEGPARCPDCHQLLDSPTLKTYGGDPDNAVDEFVALADPQLCVFEDDGGQEDINETPSHRLTDFTVYDKQGHVCPFDGGLIERNVELFFSGVVKPIYDDTTDVSVGVLTARLGPIDVWWTAGFDGGDSALIGFSTAYADYYLTAPSKEYEHIMSLMVEKIYMSKLVIEFLEENPHESYEDLVDMIQFSTPPKGCNKFSEDTLLRHAQFVVEQVENYDTPDDAQDSYFVLSPCIRALIRLAGVTLGGKRRGIRRPRAPRAPRPKKEKKIASASMAVTTPLVRNIFDVFFQDQIDGKVATSGRRERCNVCEACRQSDCGKCPPCRTMTKFGGDGKSKQCCVQRRCPYMELKAADEDEDLAERKEQDGEDVSDKAKSKQKRPAKQARSDQSVIKTKVQWVGEPAITKGKKKYYKKVLINDEEICVGDCVALCPENPTDPHYIALVAYMWDNEGSKTFHAVWLSRGSESILGEAADPGEVFLVDQCDDNPLGSILFKCALEHRAPSANWRMEGGEEEAAATSGYGDKKFFYQKWYDLEAARFEDPPADMLALLSKSNARPECVACTRQAAEEQRNEPTPGSEIKSEGSTSFYSSFAFEGDTYSVGSGAYFLPTAFSFKYKAPVKKPALPVKKESVDEELYPEYYRKTWGHIKGSNLESPEPFRIARILQIFSQPDVDTLQVRVQKFYRPENAKLSDAERFAADWNILYSSVEECVVSAADIQGSCSILHSQTLTVPLSDYSARSTDNFYFEKEFDGTTKEIADAPKALPESADKTSEKSGKGTKGRKSRTSSSGADFAAALLAESEPTPFEPLRSLDVFAGCGGLSEGFHQSGIAKTVWAIEKDEAAAEAFKLNNKKSIVFTDDCNELLRLVMSGRTENKSGQPLPQKGEVDLLCGGPPCQGFSGMNRFNSREYSQFKNSLVSSYLSYCDYYRPRFFVLENVRNFVSYKQNMVLKLTLRCLLRMGYQCTFGILQAGSYGVPQTRRRAIIMAAAPGEKLPRYPEPTHVFSPRACQLSVMVDDKKYCSNISRAESAPYHTVTVRDAISDLPAVTSGASKQQITYSAESKSPFQRVMRGDKTEGSVGDHICKDMSALVVARMELIPTTPGADWRDLPNKVIKLNGENIPKLRYTHPDRKNGRSSTGAVRGVCSCANGKACDPADRQFNTLVPWCLPHTGNRHNHWAGLYGRLEWDGFFSTTITNPEPMGKQGRVLHPEQNRVVTVRECARSQGFPDWFRFYGNILDRHRQVGNAVPPPMAAAIGREILRSVRQYSAAAAQPVSPQKK

>Scil_scpid45473

MAGNGDQADVLRVLEFYSGVGGMHFAAKASGYATTVVSAFDINTLANSVYQHNFCKTPVSSRNIESLSAEELDAFKADAFLMSPPCQPYTRQGLQAGSTDPRSQSFLALLDALPKLKHPPRFLLIENVKGFDTSETRDETIRILESCAYNVEEYLLSPIQCGVPNSRLRYYLLASRNVTSDSSNSCDAPSRAASPPVHTIRSELPPLRSVPMTSPLLREAWLSTAGNGAAQSGGDPGASHTPPAISTATAATTSSIPTPVSTKADCARRGTLQDGSTAACASPSSPDEPDAKRPCQAEQSCSRGAAMKNTATSGSASSWCECGSQFAPEAPVLARVRSVQDFLEHGMAEGEIKKLLLPDSTLSQYRKVLDVVSPSCNHSCCFTKNYGRYLRGTGSVLNCAQPVSVSPVDSNSTSPRPHQAEENLRYFSPQEVSNLMCFPASFAFPDEVTTKQRYRCLGNSLNVLVVAWLLQYLFDGSLVAA

>Skow-DNMT1-prot-modelPK

MPSKVDDSIPEEVRGRLAKLQKDYEAGSVKDKNFAEQKSELLLEFLPETLQKSLTTLKEDLKDECLTEKGYCKKVLSLLSQYLTDLPAVSTKNGLSESSQSESDSGCQSQTDQSTDQPDGDISFETPECTSQSDMSSKETSQPDEASSQLTADETSQSDMETDQPDGKANGQSMKRNKGRPKGPQLKKKKSPARGKTSDTSDVDDTSPQSSKKSKIKKDSKQPNIALMFAKTPSKRKTSEQSDEAEGGFNCAGVKSEHQHEKRMKIGDGSETFDDDEDEKKFKKDLRPKTENDEISAVAPPVRCTECRQLLDDPDLKTFPGDPDDSVDEFIMLTDPRLSLFTGDEEDVTGYEDRPQHKLTNFSVYDKNTHLCAFDIGLIEKNKELFFSGHVKPIYDENPSIEGGIATKAMGPINEWWTAGFDGGENSLIGFSTAFAEYILMTPSECYAPIYNSMKEKIHMSKIVIEYLQNNQFSTYEDLMNKIQVSVPPANCSTFTEDTLLRHAQFLVEQVESYDEAGDDDEFSLLTTICVRDLIKLSGVTLGIRRAARKAIKKVTKKDVPKHTKATTTVTVRNIFESFFRDELDNEKTELPTRRQRCGRCEVRCVQRLVMKKFGGSGRSKQACQERRCPNMAIKEVEDDECEDLERNDDENQKKPTKKITTPIKKGNRKTKMKWIGKPIAEENKKKYYRSVRINDEEISIGDAVSVSPQDPTKPVLLAFVTYMWEDGNKDQMFHAKWYCRGVDTVLGEMADPLEVFAVDECEDTALEYVMGKSKIMYKAPPENWSMLGGEEVENFITDDDGNTFYYQKWYDPDCARFEDIPVTQRPDDMPSHRYCDSCQRMEQIRKNETPHVSEKLEEDKQNPKVVYYSVLSKGGLDYRVGDSCYLLPGSFYFNVKPAPPPSKVSRKENVDEEVYPEYYRKTSEYVKGSNIECPEPFRIGRIIKIFTKKSPNKLVEEIKLKVVKFYRPENTHKGVTASYTSDLNLLYWSDEEAEIAAYDIQGKCTVMFEEELNESVQEYTRKGRDRFYFLEAYCSKSKEFEEPPSRARTIKGKGKGKGKGKSSTPVIEVKQVAAVKDNFQYRKLRSLDVFAGCGGLSEGFHQSGVAESTWAIEKEEPAAQAFRLNNPGCTVFTDDCNTLLRLVMDGETTNSVGQKLPQKGQVELLCGGPPCQGFSGMNRFNSREYSKFKNSLVVSYLSYCDYYRPKFFLLENVRNFVSFKKSMVLKLTMRSLLSMGYQCTFGVVQAGLYGVAQTRRRAIILAAAPGEKLPFYPEPAHVFSPRACPLSVTIDEKKYESNMARVWSAPYRTITVRDALSDLPEIRNGYNKLEMSYDREAQNGYQKAIRGSQHQPILRDHICKEMSALVEGRMRHIPLAPGSDWRDLPNIAVRLSDGNTCKKLRYLHPDKKNGKGPNGQLRGVCACAMNKPCDPTDRQFNTLIPWCLPHTGNRHNNWAGLYGRVEWDGYFSTTVTNPEPMGKQGRVLHPEQHRVVSVRECARSQGFPDTYRFYGTILDKHRQVGNAVPPPLAKHLGLEIKKCAEVSQRHQQTLEDKK

>Skow_XP_006813977.1

MGLRVLELYSGIGGMHYAVKESGVSADVVAAVDINTIANEVYQFNFPDCNLMQRSIEGITLKEFMKINADVLLMSPPCQPFTRVGLQADSNDPRTRSFFHLLKTLQSLSHPPNFILVENVKGFETSDTRNNLIETLNVCKYGYQEFLLSPNQFGIPNSRLRYFLLAKRHPYKFNFKLTDQILEELPQKQNLTEMSQDCTQETRPSLVRKSAEKSDGNKFSDCAMDGLSQLLEHCSCISDKVTESCDDDGASSSSSHLLVANSNIDHNDQSIRCVREFLEGDVQDITDYLIPDKILFRFGKVMDIVKPDCRRSCCFTKAYHHYVEGTGSVYQMNTTADTKKAFEKFLKSPSDDDDERISLLKNLQLRYFTPREVANLMCFPPEFSFPESISLKQRYRLLGNSLNVHVVAVLLQKYLLDDCQQKKS

>Skow_XP_006815704.1

DNVIMTGGKYDHSIRRVVHGLREKPSKVEKFQSPETKKKKKGKGKHEEKQKHGLHRKECIAAVKIESNGQKNSVAATEESQNHSMNEMRFCIGTLVWGKIKGFPWWPAVVISHIEVEKPPPQLIDQRWVRWYGDLKHSLVSLDKLQPFHEFKPYYNDVIFKQQQSYRKGVYQALTMAAKRAERPVVTPDIAPTSDVTPLKTPRGMPIEMVTLCLDLVNWAMEGFLPNGPGAIVPSKAEKMRKVVHGKKTLRDYCIACGDDDVITEHPLFEGEVCDECRQSILETAFLYDDDGYQSFCCICGEGRELVLCSNDGCFHCYCLDCLDCLVGRGATRKVRHNVDNGLLKCRKDWSQRLHQFFSSDDNNSEQFESFPVYCPLPIEERKPMRVLSLFDGIATGGTGCLFFDFYRVMRDLQVENANRPLFWMFENVASMRYEDKIDISRFLK

>Skow_XP_006824674.1

CNPVLVDAKDVSAAHRARYFWGNLPGMNRPSLATESNNSLNLQSCLEPHCNRQALFNKVRTVTSKANSIKQCKEQILPVKMGTKSDALWCTEMERLFGFPEHYTDVGNMSRTARQKLLGQAWSVPVARHLLAPLKEYFACDMYKYANYCT

>Sman_198180.1

MRVLELYSGIGGMHIAFKESTVKHEVVAAVEINGVATDVYKYNFPNTLTLNRVIESFSPDYVCSLNANVWSLCPPCQPFTRLGKRMCEADNRSSSFFHVLDLISILKPAGIILENVKGFEHSEPWRRLIEVLNSCDYEYRQFLLSPLQFGIPNCRLRFYLLARLRSSSWNSNFKMGKSESIDLRPPIDAPMLPGCQCTSCSGVISHIEHTDDNFTEYIQFCRPISEFLLVPSDSSKELYFLDEKCLQRYFRVLDIVRSCDKKTRCFTKGYSKRLEGTGSVFQTSMENEVSFFY

>Smar_10448

MSSKYETRSKRPRPSVEKWEKFRKKRHEKKVIDEARKSNEIQKCKYCSQMLEDVSSAPSFNSAIDEKNCELIINPSLTKIKSFKVNNFSVYDQMFHMIQLDWGLMEKEEVVTFISGYVKLVCCDDEYDDSEIGQAVENIGPLTEWWITYDEKDAQIGVTTELGYYYLMTPSADYRVYFYPLKEKSFLVHIIINFLEDNEDITYKELIRKIEAATPPQDYEDDIEVTEEAVRLHAEFILEQIASYDESADDVDKLSRTDCIQHLIVLSGKIIPEKKRMQSTSRSTSTNVQPKQTMQQKKFQDKRMKKKTQKECNTKLVNRIFDSIFQDYVCCLEDVNNRENSDFECEIDEIEQFEHDFYEKELKINYFDDFSSTKLEVSFNNVIECRNEVSLFENFKIHGEVFKSISISLGEDNQHSARQNRKQGSASDTILGKAASENEYFLSNYCQTIGFKSIKCKSDIVFKPQLSNWKSIGGEDLEEKSINNAGRRFYYSKFHDQKTCRFEDIPEAFNNLDADSCPICEIKNRETQGTISLPRYTLNEEIELNLKLYNSVKWNNIEYKIKDSVFLLSSAFGFDAVPRSLPTRENFREVDEDKYPEYYRKAKSKESLSTCEPYRIGEIVAIYTLRKEVGVRIRKFYRPENTHLGKQATYAADFNLLYMSNEETNVNISAIVSKCKVTYCRTPSKIELTYDQQHFYYNQTYDARSEELTRSETHDEEVDEEPAETLPLATLDLFCGCGGLSEGLVQSGAAKSCWAVDEDLVAAASFKKNHPDCVVYGYSCSTYLSMLMDPDCELNLPAKGEVEFLCGGPPCQGFTGLNKFKDRLCAKLKNSLITTQLSICDWLRPRFFLMENVQNFIRCNDSMVLKSTLRCLIAIGYQCTFGLLQAGNYGVPQRRRRVFILAAAPGEKLPQFPLPTHVFNSHSTNSITIDGIQMIPRHNWPTLAPYRNVTVRDTLSDLPDIEDTEPNESQEYNAHIQSHYQKILRKNNNTGLIRDHFYKRMNAITTERILRIPRTHGADWRDLPNIEIKLSDGTWTKKLEYNHDDVRNGRSRNGSRRGICRCAEGGRKSGVNCNIYRQKGTLIPWFLVHTANDNNQWNGALGRLCLDDVFPIALTKLTISGKIGRVIHPEENRILSVRECARVQGFPDSYIFCGDIHDRHKQIGNAVPPLLARAIGSEIRKCI

>Smar_10552

MPRPKPKKAAGKKTNEPKKPTQRALRDSTVKKTNISRGEPDEPITVESKKHKMSESEANHEIIPNKRRKYEKYAKKSTENTEDIAYNHVPDVEKENLNGDKKDVTKMTTNSKPQIIRCTECSQIVDEKIGIFPGDSDDAVDEVTALFNPILALEGREMDDYVSSSLPDYKITQFTVYDVNTHICTFDTGLIEKNRKLYISGVIKPVYDDDPSPEGGVLAKTIGPISEWWVAGYDGGNDVLVGVSTELACYYLMTPSEIYASYMKSIMRKTFLAKLVIEFLNSDETGTYEDLINKIQATPIKGCESFTEESLLHYAQFIVEQVDSYDQAADADENLLNNTECMKALINLSGVSREEKKYIRARIRNVQKKPKDTKATTTPLVRELFELMFKDQIETAPKSTLRRKRCGVCEACQRPDCGACNNCKDMIKFGGKGTAKQVCKHRVCKNLGYVENGDVSDEEEVDENVDVNVKPKREFTIPKQKPKVSWVGEKIDFYSKKKTFYKSVKINSETYNVGDHVLVYPNSPEVPMFTAKIMYMFEDNFTKKKIFHAHWFCRCTDTILGETSSDNEVFLVDECENSLLDIIMSKCEIVYRPPPTSWAYLGGEDVAELMESYGEKKFFYKLFYHRRTARFEDPPKEYIEVDDSEDKKCDFCPCCNRLHTFRNLEKLHLGNILSETSKTIFYESVKWHNVEYSVGSQVFISPDAFKFKAKIRVVPDKNVIEEVDEERYPEYYRKTKDRGENIDLQEPFKIGYVEKIYCAASKNGLVNSSDVKLVVKKFYRPENTHRGIDASLYADFNLLYWSDEEAVVDLTVVQGKCHVTYCTDIARCESLWGAAEHHFHFSEAYNVESKMFEDPPNKAKLLGSRGKGKGKGKGKKTEDIQQDSSPKLLYFKRLKAMDIFAGCGGLSEGFHQAGICDTLWAVECDEMAAQAFRLNNPDCTVLNQDCNDLLRMVIEGQTKNNQGQTLPQKGDIELLCGGPPCQGFSGMNRFTAGQCSQFKNSLIVSYLSFCDFYRPKFFLLENVRNFVSYKQNMILKLTLSCLVRMGYQCTFGVLQAGNYGVPQSRRRAFILAAAPGEKLPNFPEPRHVFKHSSCLVTVDENKYVTNVNWMDSAPYRRITVRDSLSDLPAIENGAKTEELSYTPENQSHYQKILRRNNVTRVIHDHVCKEMRPLVAARIRFIPCQPFADWRDLPNIEMTLSDGSRTRKLEYGFEDGKNGRSSTGALRGVCRCAEAKVFKKCDPLHRHPQDSTIIPWCLPHTSNRHNNWSGLYGRLDLDEHFKTTVTNPEPMGKQGKVLHPDQHRVISVRECARSQGFPDAYQFHGNVLDRHKQVGNAVPPPLAREIGLEIRKCLKYVEPNGESDKNEPTQILTDSLAKPGSSKF

>Smar_15548

MVKIKKSKMREVEVLLDRNEVENYLKNIGTNHTNANTFELKELSIVLEDCLSIKRKLPGLCNILNKKGKYEDVQQNETKNSLASRCPNCLQFLDDNLVSLTVREDAVYETDILSDPNLNVDIGHGAVCYDETSKPEYKMVDFAVYDDEMHLCPFDSGLIQNGHVLYLSGCAKPIFSDDPSSEGGVYVSSIGPITEWWSWGYDGGSAVVIGISSEFADYYLVEPSASYAPFMESTKIKVYLSKLVIEFLEGNENGTYADLINKIQVHTPPEGYSNFTEENLIHHAQFIADHLLSYDQVADADENSLCQTKCMKTLIRLAGVTIRDRREITTSSPKTKNSRGLQKVVRLQPTESKATTTPLVRKVFETIFRDQIASEGRLSSKLVKCRGCKKSDCRSCKNGKKNNSRAQTSDSEVSDSDSEVSHSDIESASEVKTTKFTQLKEFDRSSIQVTWIGNKILTIGNKMFYNSAKVNGELIKVGDSVIVNPENPKSQLLLGKVMYMWEDLKNRGKSFHCHWFCHGSDTVLGSTAKKNLLFLIDECENVRLSDIKSKCTAVYKPPPSDWALLGGTEIENDRDEDDDCYFYSKWYQRSCARFEDPPNIYTDKGTNLKCNYCPCCEVEESARKKKMVEIGDEMDSDSQYTYYNSVSWNDVEYGVGDHLFLSPGSFKFKAKPYAPSKINWPVTEVDEEMYPEYYRKSKIAGKGHIQEPFCVGHVETIYCRSTGLSQLVEPKDVKIRIKKMYRPENTHEGVSASFHSDFNLLYWSNEEAVVDFSDVAGKCNVTYCEDESNDQFYENSNEHNFYFNKVYDAHSKTFEEPSSKLRRLGDRYKRINKDETGRPLRALDVFAGAGGLSEGLHQSKMCETLWAIEFVEVAAEAFQLNNPKCNVYNEDCNVLLKMIMEGKTVNAKGQTLPKKGDVEFLCGGPPCQGFSGMNRFTTGQYSQFKNSLIASYLSYCDYYRPKYFLLENVRNFLCFKENMVLKLSLQCLIEMGYQCTFGVLQAGNYGVPQSRSRAFILAAAPGEKLPSFPEPLHVFKSENKCSVVVNDFKYTINTQWIKSAPYRYVTVRDSLSDLPPIKNGAKIEKLPYCPKEQSHFQKLMRRNSKKDKIYDHICKEMKPLVVARMELIPCEPGSDWRDLPNTNFVLSDGNVTNTLIYRYNDAKHGRSKKGDLRGVCQCVEAKVFKKCDPLYRQPQDNTIIPWCLPHTANRHNNWAGLYGRLELDAHFKTTITNPEPMGKQGKVLHPTQHRVISVRECARSQGFPDSYKFCGSVLDRHKQQSDRYSHQQSCHLTTHHLSLDKQPWLWLQH

>Smar_7615

SKNTENKRTINEVYDDQDYETIYKLKTCSVVLKDCLSSSKKKRSNQSVGEDGFYSPEKWKNTHDRQDKSFATSSANRVRVPSRCSHCAQFLTDNIISLEEDINAIDEETALQSPTLIIDGEDQDKYCKLTNFTVHDKLNHVCPFDTGLIEKGKQLFVVGNIKPICDENSDDFNGIRASLGPIIQWWVSGFDGGKKVLVGISTKFAHYYLTKPSEIYEPTMKVVMEKTYLTKLVVEFLENKEDPTYESLLDYLENVDSLEGHEKLNEDKLLYYAPFVTEQIETYDSVADDDESALIETECMKQLMEIAGIIIDGKSGAACNIRKTFKGAPKSKVALKPTTTPIVQRVFANEEIEDEHEDAEIINSSPCTRRTEKLRLKTRCKWIGCSIRCNGKLYYNSAEINGTKFSCGDCALVQTEGNSNSSTHPARIISMWEDVFSNLKLFHVQWFAFGADTVLGDIANEYELFILDQCENLPLKHIKCKCEIEFKSTPVNWSWLGGEDINVVQSPKYFYRKFYQPVSGRFEDPPLEYTDYSEKKYIGHCGCCDRLKASAQQKMVQFGPKLSEFDGENDYQFIKWKNEEFKVGDFVLIQPDAFEFNRKQCQKVKIKKIEKVDEKIYPEFYRKAVPKDIEIPDPYKIGFIIRIYCSVLAANNTQCRTHPSAIQVQVQKLYRPENTHLGIAASYHSDLNELYWSEEGAIVNLTQVRGRCFVNYCEDISNSHLYWDAGPNYFYFHEAYDAENRLFHQPPMSICESLSKLVYSDINKIVTPLRTMDVFAGVGGLSKGLHQAGVSETRWAIELDQFAATAFKMNNPAPELYEDNCNEILQLMIDGKRTNSKGQILPVKGDVEFLCGGPPCQGFSGLNRNRNSISSRNNNSLVSTFLSFCDFLRPRFFLLENIPNFIRFQNNSYVRKSFKSLLDMGYQLAFGLLNAASFGVPQKRKRLIVKIFYYCCSARGGIAAFSGTFVC

>Smar_SMAR004394

MADIRIHGKMNENTQILRVLELYSGIGGFHYALKDSDLKVEIVAAIDINTNANQVYMHNFPSTPIFQRNIQAINSKHFECFMPDLVWMSPPCQPFTRNGLKKDKMDHRTDSFLNILENLKLIRKKPKFILLENVKGFETSETHDLLTQTLNDCGYELNEYLLCPRQFGIPNSRLRYYMIAKLSEESVIPTRTSVINHTLNHSGEQIRHRNISEFLEDEFSDEFLIPDRILGKYAQAMDIVTSANDHSCCFTKSYGHYATGTGSVLRTNEMIDMEAVFLHEYQHENEKLNALKALQLRYFTPREVANLMGFPKTFGK

>Smar_SMAR006764

MPLTERVQGSDNMEDDNNQADEEEDSEDYEFDASVPAALEKKIEVDDVWEGEPSNNTDEEDCNFLGFRSRTTTKVNGTDGDIVAPKKRAKAALVRRVRRRRRGRTVTKSLRVAGNSEKAKIIKPKKKHQLSCVSPKRGQNGWEDSLRPRPAQAQHYQKPDEFLEKLQPLPMTKKEICVEELVRDDIDEGMIDETNESSLLELGDLGGKKRGLRGMKRNRKRRGLLMNGDTQQEDAEQSELLLRAQANRSNSRKQVPNGVVVPKSVAVVPKPEGSEQDSETEPRYVSDASVGEVVWAKLGSTRWWPAILIWGSDCGQQPAHAGNTWIFWFGDHKISEIPRDRLADFTVNYNRYYSGGGSKAFHCGVIEAIRECAARAKVDISDSDSNALLEWAEKGFISESKSEGVFLPDPNNSIPANIRKHVNKIKEVNLLYFQGVDTKGHQVAPARTPRNDCLDNKDSGLRKVKEGLIKIEKVCIACDSDRVPITSQHPLFVGGLCKQCKDDIIETMYAFGEDGTNAYCVICGNAGELLICDNNDCNRVYCTGCIEIMVSPEACKKVLETSPWLCYLCTEYNPESNGLIIKKDDWQQNITQLFQPEKHVQIPNIEDYKEKRPMRVLSLFDGIGTGRYVLDSLGIDIEIYYSSEIDLDAVNVATVQHYNKVVQLGNVEELTDAEIAKLCPIDLVIGGSPCNDLSLVNPARKGLYDPTGTGKLFFDFFRILKAVQLANMGRHVFWLYENVASMPQEYKVTISRFLQVPPTTIVSSSSSCDPALLDSKYFSPQNRARYFWGNIPGMYTPLQPHMLQKNITLDNVLTPNLNRKAVVSALNDLRVG

>Smed_15036408

MKVIEFFSGIGGIKCALNELNIQTDVVAAFDINDLANSVYRYNFTSPCYNKVIESISAEYLISLNADMWTMSPPCQPYTRNGNMMDLDDPRTVAMKHTLYLISQVRPHYIFFENVKGFESSNGQKMLVSILSESAYSFQEFLLSPLQFGVPNSRLRYYLIAKLEGKGSLMQDLEAISYKPYFDRKLYQCNCPVCSGRSRSLENDHVNHFERNLEFCDRISAYLEADNLAEPHEGHKLIDEKVLEKGFSKLDIVTESSNKTCCFIKCYAKKIEGSGSYYQMTSCDEAHQLKQLLLNGDISSLDYAKRLKLRYFSPREIANFMCFPQSFRFPETVTRAQRYRLLGNSVNVKVVAHVLHWLISA

>Spur_XP_011668392.1

MFVMSPPCQPFTRVGLKGDKNDARTNAFFNIMRNLAEMAKKPTYLLVENVKGFDTSETRNFLVETLQKCNYVFQEFLLSPMNIGIPNQRVRYFMLAKQRPLQFKEEHGTQLVQHRVNMPLMPNEIKETSRLVCDNSMVSSQEQTNMELTESRERTGQDESSMVSLNEQSIERKTDEQHLNEKERTDNVKSGSCEDMVSSSEQNEQETGEDDERRKIGDYLQADLSEESMAEYLIPDRILLKYVNVMDIVTVEDTKTRCFTKAYAYYVEGTGSVLRTDLSADMSSAFSSGSCSDDERLERLKALRLRYFSPREVANLHHFPQDFGFPECSTKKQKYRLLGNSLNVTLLAQLISYMVA

>Spur_XP_780273.1

MPSKTLCDRVIPTNVRNRVQELEGDLNDGLITEKGYIKKKSKMLFEHMSSDMQTTLKGLEDELKDEELTEKGYVNKVKSILAKFLDTCTPISNGDIQQENSSKKKLNKDTQSTVKNGSSSISSNGSGDPCKANGHSNGSHVRASDQEGNGSSQSEERMETDTPTSGKGDSKKKKKKGSPGGSEGGKGSKRKVLGDSDREEEEKKGREENKEVEEEEKEEESAGKTTPDANTPRSSKRKRSPKVDAKQPSIMSMFTKKPAKKEEEKVEESLSSVDENETKMENGDDGKKEEEEEEPSGPGGKRIKKEEEEEEKANDEPMSPSRDLRQRSNNETSIAVKAVTKQPPVRCKECRQPLDDPDLKLFPGDPDDAREEYITLTDPRLSLLTGDEEDTMSYDERLQFKITNFSVYDKSTHLCAFDSGLIEKNKELYFSGYVKPIYDENSSIEGGIPTKRMGPINEWFTTGFDGGEKALIGFSTAFAEYIVMSPSEEYKPFWTAVQEKIYMSKILIEFLQSTQDAVYEDLLSQIQTTVPPEGCNRFTEDTLLRHAQFVVEQVESYDEAADLDEVLLITMPCMRDLIKLAGVTLGKRRATRKAAAVKKDKKPVFTMATVTPLVSHIFDAIFKDQIADEMKAAANERKKRCGVCEVCQAPDCGKCTACKGMIKFGGSGKAKQACKERRCPNMAVQEADENDFDEMDDSNKNGKMDDKKAKRGRKLGSPLKKKKRAKVTWIGEPEEVTKDRAFYKAAMIDDQKIENGDCVQIHPDDPTKPLFIARVIYMWQDSDGEMMFHAQWFVYGSETVLGEASDPLELFPIDECQDTYLASVNDNCSVIYKAPSPDWSMLGGIDDPETDHVIKEDDGKTFFYQKWYDPDLARFEDYELLSRPDDVPAHRFCSCCLKMERIQEKESPRPGAKLEDQEDSSRITYSSCHFKGNEFQIGDGVYLLPEAFSFNIKQKVVTKKPISKKDVDEDLYPENYRKHSEYVKGSNLECPEPFRIGKIVAIYTTKSNSKVKLQVNKLYRPENTHKGRTAAYQADLNVLYWSEEEAIVELSDVQGQCSVVCAEDLNVSVDEYSAGGPHKFYFREAYDSKRKCFEDPPSKSRSNRMKGKGKGKGKGKAKGKTEKQEEKESTDNPFHKLKCLDVFAGCGGLSEGFHQAGICESSWAIEKEEPAAQAFRLNNPGSTVFSDDCNELLRLVMQGDKTSRTGQKLPQKGDVELLCGGPPCQGFSGMNRFNSREYSKFKNSLISSYLSYCDYYRPRFFLLENVRNFVSYKKNMVLKLALRCLIRMGYQCTFGILQAGQYGVPQTRRRAIILAAAPGEKLPFYPEPLHVFSPRACSLSVMIGDKKIQSNNQWCLSAPYRTISVRDSMSDLPTISNGAQKLEISYDGEPQSDFQKKIRGNQYQPILRDHMCKDMSALVAARMKHIPLAPGSDWRDLPNIPVPLKDGTTCRKLRYTHRDTKSGKLSTGALKGVCSCAEGEACDPSDRQFSTLIPWCLPHTGNRHNHWAGLYGRLEWDGFFSTTVTNPEPMGKQGRVLHPEQHRVVSVRECARSQGFPDTYRFFGSILDKHRQVGNAVPPPMAAAIGMEIKQCLQAKAKKDQERQALEPVKEEIEESMD

>Spur_XP_787412.3

MRLPSKQVLLRMYPTWDISKQKRMSARISLKHGQAFLRLLRSGSSKKRTSLQQRSPSAILKGKHKLSPGGDDRSKKRFKVDTDQVPHGEVSSNIPVKDGEKDIEEQQVRLTRSAAKLQGNGRVSSQSALSLQSGTKSGPSIIAAVKEQMHVHPEGPVDDKGTLEDDSTVSSSFGPQNELFQENLKTSGEQASLSGSESTATNDRESEVSVNVQEHGLPWRHKRKSLCPRKILRKMEQEEASAGVPGNFIQEEWLTQAEDSASSTPTDERNVPESSQEQVRFSDPDKEILSECIETPPARLESPSVMESEEQSGQLNLLLDQDPMLAEGNFSCSSDQTMVPSSSQGAIEEAVSFPGLRFQSIQDFPSKKNDTGDSVEGNSASLLGEGFADVVNSSLIEETVQGDSLDNLLPGLNMLHVGRGVVEGNPLNEKGCRSQEKKDKTPKVKKHKHKSPHKNKRTSSARRLDMQHVGDKEASHDDKESSPPGTKDKLHKKRKVKKNKHKSHEKCTTKSTSPGKFPSSDQSSPLPKSPVFGRILPVSNWASLEDPVTSPCRVQLVDYLTSSQEKLPPKEGDEGNTRVQEVSSEAPQEVHQADQVATTLKEQVSTEDECPGVQLHEQDVHTEEVEKEPSESLSSPISSFKIREEPAEETSAGNRPCSSAVKGQRSPSHQGNDQSRAVRECGTTEGSSPHFPTGQEDSGQVCGSLDADGKIDTPSVGNDGNKRTSDKTKKAVRSRNPRGAGGWEQSLRQRPQKVSPFQVGRDPNTPKKGAKKVSPKKDTSRRLQQDIHSQMLPLPREKSNDVSSISNRPSTPWRKKEPRAVATPPPLDVKGTEGEGTSGSMEVKIKEEAEKYGCIFRGQLVFGKMKGFCWWPGRIVHHYDRSIREPPPPLTRWVQWFGDDKYSLLSLGQIAGLEDFPDYFSVSAFQKWGLYQKACYEALNVAAKRAGKDVSVAMPTPPAYPGASIKERQEYKAKMLERCREMQDWANDKFLPQGRESIQPSEEDKQTPPIPESPPPSPVQDRKRSPIKHSSRNSPEKEPFEDKREERMKDVRGGKWTIEALRIEYLEVAYLYDCEGYQAHCCICAEGNQITLCDNQGCYHSYCTVCMDNLVGPQESKRVSEQEPWSCYLCSADCSHGYLTRRDDWQEQLVDFFNRDQVADFEPLRMYKPLLLSNRRPIRVLSLFDGLGTGMLVLRELGFDVECYYASEVSEEAITVAAVRLKGQIQQIGDVQKITPKELKSWGPFDILIGGSPCNDLSIVNPARKGLAGGTGLLFFEFYRILRDLQPLPDDPRPFFWLFENVVFMGRKDKLNICRFLQCNPVMIDSRHMSPTHRARYYWGNLPGMHRPYVAGADNPLCLQECLEPHCDRQAQFSKVGTITTTSHSIRQTKDAILPVIMNGREDGLWSTELERLFGFPDHYTDVGNLSRTARQKLLGKAWSVPVIRHLMAPLKDYFACSAQNE

>Sros_PTSG_05624

MSKMDADGDGGDGTTQPLRVLEFYSGIGGMHAALKVADPTARVLRAFDINDTANKVYRHNFPETPVWQRLIESIPRERFEGKLQADMYLMSPPCQPFTRTGKQQGIEDKRSTSLRFILDLITTMKTPPRYILVENVKGFESSNARQPLISALQSRDYSFQEFILSPDQFGIPNSRLRYFLVAVRAPLQLPSPPTGTVLYHIPTLGGAFADGYSPQIAAHSFLPPAPQQVPGVPPVRPWTLRPVRAVGAYLSADEGEIAANLVPMKVVLRHGQLFDIVDATSHRTMCFTKAYSHYAEGTGSVVLGAKDATLEACARVFAEVEASKAQPPQPQQSSPPQSHQSSPPQPQQSQPSSSSASASASSKPSDVERPRKQAKKHEGAHDDGDDDDDSHGDDGDDMNEVQEGEGTSTVTAEATATQPTLAATDTAAVAVHTTATTNTVSTTCIDASEALGALRLRWFTPREMLTIHGFADTYTVPADVTAKQMRRCIGNGLNVVVVAELIKFMLASNPAA

>Sros_PTSG_11488

MSSLRELRVVDLYSGIGGLHAALGVAIARINARPTRAGQAPWSVEDVRPFDVNTAANKVYQHNHSIAPSPRGIDALTPRHVRDSNLWLMSPPCQPYTRIGKQLDVEDPRAASFLHMIEMLSKMESPPQLLFLENVKNFEHSETRRRLLHVLHQRNYSVQEFLVSPTQLGIPNTRLRYYLLASRSAGPQDTATAATAATDTSTQQQHRQPDAAPPTTATSSPATAAVASSPVHAFMPRLQHDSNTNDDSTNDDTGHGDAGYQDQTVPMSARGRQLPNPHPQPLPPPRCMLPMGDYIATSLPPALVEELILTPATLQRAGNFRFDVVTLSSLTSTTFAKGYGVKGHLRGSGPVLLLNSGDLTPASTDPSNGDGDDNDDDDMRTKNDAAADAAAADDDDDDDDDDDDDATDDGSQCSKRARRGQLDQVQQQQHQQQVVVGVRDEKRSFRSLQAHEKARLFAVSEMLRLHGFPEAFEMPAGITQKQGRGLVGNSVNVEVVAHLLQYLLENIACVADADASQQP

>Tadh_49657

MSSARGQVMQVVEFYSGIGGMHYALQESNINAKILAAIDINTVANNVYRHNFGNTPVWQREIGKISLKELQELNGDLYTMSPPCQPFTRLGKKADVNDARTSSFLHVIDLLIKMENPPKYILLENVKGFETSAARNEFLLTPLQFGIPNSRLRRGYDGTSEFRPNVVKPDSQRSCCFTKSYFHYAEGTGSVLQITNPILHLKLRYFTPREVANIHCFPVHFNFPENATKKQCYRLLGNSLNVHVASELLKLLIT

>Tcas_TC013587

MSLRKRFRESPAQETGDNTKKHKRTSTHSDKCKFCAQTLNQAVVYTNFPDDYAEESVALTSERLNVYNGTENISFEEGDLPSHKITCFSIYDLNDHLCPLDTGLLQKEVYLHIGGYIKPIFDDDPSPENGIATLDMGPIHEWFIAGFDGGEKVLLGIITNYAQYVLMEPSLEYAPIFKSLQEKTVLFKFVIEFLLSNFSRAPSYEELLAAVENSDNPLLTEEFLLQNAQFVCDQIHSFDLDAGDNNDMPLLTMPCVRTLASLAGVSFRKRQLMRNLLGNQRRTKLQLSKATATPLVRQLFEQLFPNQIQTENCQSPKKTRCGICDTCRSPDCGQCVYCKDMVKFGGDGNMKQPCKLRRCPNLVVQELDESDDETVVTPARGREDGRRPGVSRVLSDGPLQNFRESIDIVLGGVNDDDTGILLNIKTGDFVQLSPKSATKPNTIARVVNIYTATEPMVHVYLFYRGNETILGEVANPQELFASNECEDCPVAAIVGQAKVVYKATPDNWADLGGIECLPSGDDRFFYAKQYDSETATFTDYVKVRNELDSCGHCRLNEERKKIETPTFKDNKVQWRNEFYEAGNSVFLDPSVYQFPIPVECTFDDAKEMNESKYPEYYRKREANASCKKMPQPFCIGLIETLCDGPAGVLMAIRVFFRPENTKGGSMLSYQSDINLLFWSTKFITVPFRKVMGKCYVVYCSDEQKAREWSEGGPYRYYFRQQYDPDCGELVPPTYSCTRLALREGDERPFGRPLKCLDVFAGCGGLSQGFHAAGVANTKWAIENDKPALDTFRHNNRTCHVFRDDCNVLLRNVMSGKGGLPPKSEVEMIVGGPPCQGFSGMNRFNEGEYSLFKNSLVVSLLSLCDYYRPLIFVLENVRNFMLYKGGLILKLTLQCLVAIGYQVRISIVEAGEFGVPQARRRFILIASAPGYQLPRIPEPQHVFLQRGSRLDIYVDGVKYTNGNFWTQSAPYRMLHVRDAIADLPAIEHDDNRPQMPYDDDEGTSHFQRKMRGNQEELLDHICKPIAPIVQTRIKLIPPKGGADWRNLPNVRVQLPDGTMTEVLQYRYRTKKQKDGEPNRGVCACSVEKACDPNDRQSNTLIPWCLPHTADRHNNWAGVYGRLDWSGYFATTTTNPEPMGKQGRVIHPDQNRLISVRECARSQGFPDKTKFFGSVTSKHVSNVEDRSPVKRLEEPQRSPKKLRLEIEIDENMSDNSPERNRRANFSNVNGVNRNLNIHSVKPTTKKLIIKNFKSKGEPKLPDDYHETTWEKLKSAVIAIQQSKPNEYLLEELYQAVGNMCSHKMSHILYNGLSHLIEAHVCSNIERFITEPMDRFLFLKKMNDTWQSHCNQMIMIRGIFLYLDRTYVLQNPNISSIWDMGLDLFRKYFMLHTLVQTRVVEGLLMLIEKERQGDKVDRTLLKSLLRMLTDLQIYNKAFEQKFLQATERLYATEGQRLMQELEVPEFLAHVDKRIHEENERVIHYLDCSTKYQLIHTVEKQLLSEHINNILQKGLDNLLEENRLHDLSLLYQLFSRVKNGLHELCLAFNAFIKKKGRTIVIDPEKDKTMVQELLDFKDAMDNIVACCFKKNEMFSNSLKEAFEHFINQRTNKPAELIAKFVDSKLRAGNKEATEEELERLLDKIMVLFRFIHGKDVFEAFYKKDLAKRLLVGKSASVDAEKSMLSKLKQECGGGFTSKLEGMFKDMELSKDINVAFKQHLNISTLDLIPLDMTVNILTMGYWPTYTPMDVTLPPQMVKFQDIFKEFYLSKHNGRKLQWQPTLGHCVLKARFKAGQKELVVSLFQTLVILLFNESDEHSFEYIKAATNIEDGELRRTLQSLACGKARVLNKIPKGREIEDNDKFKFNNDFVNKLFRIKINQIQMKET

>Tcas_XP_008196999.1

MEILELYSGIGGMHWALKVSGVEGTIKAAVDINPTANSVYKHNFPHINLLNRNVQSLTPQFINKLGVNTILMSPPCQPFTRNGLQEDINDERTKSFIHVLAILPDLKVTRILIENVKGFERSKMRDLLIETLEKCGFNYQEFILTPTQIGIPNTRHRYYCLAKKPPNVFNFKTGVLKTEFPNQQNAPHCFEISKVLEQNELTPYYLTDKVLTNYLETTDIRYSTSRNTCCFTKAYGRYVKGTGSVYSDLPEITPEIFNQLSDHEPGSSAYLKLAHGLKMRFFTPREVGRLMSFPEDFTFPENTSDKQKYMLLGNSINVRVVAELIKLLQ

>Tgut_ENSTGUP00000014140

RKLGPINAWWITGFDGGEKALIGFSTAFADYILMEPSEEYAPTFALMQEKIYMSKIVVEFLQNNRHVSYKDLLNKIETTVPPAGLNFNRFTEDSLLRHAQFVVEQVESYDEAGDSDEPPVLITPCMRDLIKLAGVTLGKRRAVRRQAIRHPTRIDKDKGPTKATTTKLVYLIFDTFFSEQIEKDEREEDKENATKRRRCGVCEVTEEQPECGKCKARSNMVKFGGSGRSKQACLQRRCPNLAVREADEDEEVDDNIPEMPSPKKMLQGRKKKQNKSRISWVGEPIKVRGTSSVPRKVCLYLEPIQVTEPIQVRGTHPGEPTQVRGVTAMWEDSSGQMFHAHWFCPGSDTVLGATSDPLELFLVDECEDMQLSYIHGKVNVIYKAPSDNWAMEGGLDTEIKMVEDDGRTYFYQMWYDQEYARFESPPGAQPTEDNKYKFCMSCARLDEVRHKEIPKVAEPLEEADGKMFYAVATKNGVQYRVGDGVYLLPDAFSFSVKPASPAKRPKKEAVDEELHPEHYRKYSEYIKGSNLDAPDPFRVGRIKSIFCSLRGNGKPNEADIKLGIYKFYRPENTHKSTKASYHADINLLYWSDEETTVEFRAVQGRCSVVYGEDLTESIQDYSAGGLDRFYFLEQLQVPKLRTLDVFSGWGGLSEGFHQAGVSETLWAVEMWEPAAQAFPVPIPDSHSHSQCPFPFPVPIPQGCRKRSGPDQPRAFGVAILRKKRPCQGYMTTVRGRSYLSMHSQYFTGFLSYCDYYRPRFFLLENVRNFVSFKRSMVLKLTLRCLVRMGYQCTFGVLQAGQYGVAQTRRRAIVLAAAPGEQLPMFPEPLHVFAPRACQLSVVVDDKKFVSNITR

>Tgut_XP_002196308.1

MKKDKSHGQDGADCRAKLILINGDNAPSVVLEAKGKRSTPDSRRLGLKKEKNSNGDLSKEDLAWPLDLPVQRQVQLWGSAGWESSLRQKPTLCLMFQAGRTRQEMRIKEAARSVESLRDLPTPVRSSRRRSAVPTPVTIDLTEEDSQDSSRSSSTLSGSSSQEGQNGSADLGAEEAESRDMGMALEYQDGKEFGIGELVWGKIKGFSWWPAIVVSHRATAKRQAVSGMRWVQWFGDGKFSEVSADKLVGLMAFRQHFNSSTFNKLVSYRRAIYHALEVARSRSGKTFTTGPRESLEEQLKPMIDWAITGFKPLGLKGLQPPKGSENGVLRNGTEEVVSLEQCPPTKRLKTYPYNSSKEQRVEEDQTREQMVSEVTSNSGNLEESCLSCGRRNPATFHPLFKGGLCQTCRDRFLEYFYMYDEDGYQSYCTVCCAGKELLLCSNASCCRCFCVECLDVLVGRGTSTRVKEQEPWNCYMCQPQQNHGVLQRRQDWNTRLQDFFTSDKGQEYAAPKIYPTVPPAKRRPIRVLSLFDGVTTGYTVLKDLGIQVEKYIASEICENPIAMGKVRPEGNITYVHDVRNITKRNIEEWGPFDLVIGGSPCDDVSLVNPTRKALFGKAALPLLGCIPWDVPGPWSLLATWGSGSAPGCIADGLSPYSSWWGGQPGPPSPTPHAPAGPNLLLGFLLAEGTGRLFFEFYHLLNYARPKAGEERPFFWMFENVVAMRINDKRDISRFLECNPVMINAIKISAAHRARYFWGNLPGMDRIFGFPLPYTDVSNISRGTRQKLLGGSWSVPVIRHLFSPLKDYFACE

>Xtro_ENSETP00000047703_3

LTESVSPLLYTAMPAQSASLALPADVRKRLKDLERDQDGMTEKECVQQKLSLVLGFLEADARNKLSDLESKLSSEELSEEGYLTKVKALLRKQLSCENGDLSLNGETNGCSTNGTCGSDEEDVEMSESNTAGVKNRKTRKSKTNGENKTESPARARSSRSTTGKQATILSMFSKGSSDLAMHQHSCNITFDNLITSDGIRRRCSIPFQHIMLEYQFQVDKKRPTSSVKFQWSLALTSNSTESSVLSILKASPFYTITSGLLRSNKRKSSDEEKDTDVSADADQPEEKEKEEKRIKIEVNESENENRSTAEESKKVKPVLPPKTPPPKCMDCRQYLDDPDLKYFQGDPDDALDEPEMLTDERLSLFEANEDGFESYDDLPQHKVTCFSVYDKRGHLCPFDSGLIEKNVELYFSGYVKPIYDDNPSLDGGVRAKKLGPINAWWITGFDGGEKALIGFTTAFADYILMDPSEEYSSIFALMQEKIYMSKIVVEFLQNNPDVSYEDLLNKIETTVPPSGLNFNRFTEDSLLRHAQFVVEQVESYDEAGDSDEQPVIVTPCMRDLIKLAGVTLGKRRAARRQAIRHPTKIEKDNKGPTKATTTKLVYQIFDTFFSEQIEKDADKENGIKRRRCGVCEVCQQPDCGQCKACQDMLKFGGAGRTKQACTQRRCPNLAVKEADEDEEVEDVVPEMPSPKKILQGKKKKLEKKNRISWVGDPIKTEGKKDFYLKVSIDSEILEVGDCVSVSPDNPTEPLYLARITSMWEDACGPMFHAHWFCLGTDTVLGATSDPLELFLVDECEDMQLSYIHGKVKVIYKAPSDNWFMEGGTHTDIKVVEDDGSTYFYQLWYDPEYARFETPPTPQPTEDNKYKFCTSCARLAEIRQREMPRVSCPVEDLDSKVCYSTAIKNDVQYKVGDGVLLLPDAFSFSVKLGSPMKRPQKKDDVDEDLYPEYYRKSSDYIKGSNLDAPEPYRLGRIKEIFCNKRSNGKANESDIKLRIYKFYRPENTHKGVKASYHSDVNMVYWSDEEAVVDFKAVQGHCTVEYGEDLTETIQEYSAGGSDRFYFLEAYSAKTKSFEDPPNHARSAVNKGKGKGKGKGRTQTNSSKGKTASKSENEQQNCGDKLPKLRTLDVFSGCGGLSEGFHQAGISETNWAIEMWEPAAQAFRLNNPGTTVFTEDCNVLLKLVMSGEKTNSLGQRLPQKGDVEMLCGGPPCQGFSGMNRFNSRTYSKFKNSLVVSYLSYCDYYRPKYFLLENVRNFVSFKRSMVLKLTLRCLVRMGYQCTFGVLQAGQYGVAQTRRRAIVLAAAPGEKLPMFPEPLHVFAPRACSLSVVVDEKKYVSNITRTNSSLFRTITVRDTMSDLPEIRNGASALEISYNGEPQSWFQRQIRGSQYQPILRDHVCKDMSALVAARMRHIPLAPGSDWRDLPNMEVRLSDGTTTRKLRYSHHDKKNGRSSTGALRGVCSCSEGKPCDPADRQFNTLIPWCLPHTGNRHNHWAGLYGRLEWDGFFSTTVTNPEPMGKQGRVLHPEQHRVVSVRECARSQGFPDTYRLFGNILDKHRQVGNAVPPPLSKAIGSEIKKCVLSREKENGTETVKAEKMET

>Xtro_NP_001004959.2

MARPLRVLELYSGIGGMHCGLTESGVAAEVVAAVDVNTIANEVYKYNFPYTPLWPKSIEGLSLKELDALSFDMILMSPPCQPFTRIGLQGDISDPRTKSFLYVLDVLPRLKKQPAYILLENVKGFETSEAREALIRTLEKGGYAYQEFLLSPTCLGIPNSRLRYFLIAKLQKEPFAFPTTSKILEEFPSQCPGSKRIIHCSESNPQARVEQKYSSCPLSGTDCDPEKTVLYKLETAVELQRKQTQDNDSSVRMLQDFLEGNVEEMSQYFLPPKSLLRYALILDIVKPTCRRSTCFTKGYGHYVEGTGSVLQTATDVEIDTVYKSLDLLTEEEKLAKLSSLKMRYFTPREISNLHGFPATFGFPEEVTKKQRYRLLGNSLNVHIVSCLISTLLQSP

>Xtro_XP_004919670.1

MMPSNGTILNTSNPPAETDAPTHKGDTATEENKSKEEKSDKGVSMKKTGRPGRKRKHSMAESATVSKDIASVPKCHTSPNSTRPDPIHNGEMDSRQHKAKRPTQDRPAPELSPRDTKTCDSPEEVPRDSDVTPVVENGCCTPSDDIDPQDMEDCDVSVISTPKKRGRRSLSSSSEQSKEEREDDNSDSPKEEASTSQGIHRNSHCRGYRGRLRGGLSWETSLRQRPMQRLPFQAGDPYYISKRKRDEWLARWKREAEKKQQLAAVINAMEETVSREPQKEEEPCPPASQQPTDPASPTVATTPEPVAPDAVDKNTSKSADDEAEYEDGRGFGIGELVWGKLRGFSWWPGRIVSWWMTGRSRAAEGTRWVMWFGDSKFSVVCVEKLLPLSSFAGTFHQATYNKQPMYRKAIYEVLQVASTRARKVFPSCPENDESDTSKAVEIQNKQMIEWALGGFQPSGPKGLEPPEEERNPYKEVYTEMWVEPEAAAYTPPPPAKKPRKSTEKPKVKEIIDERTRERLVYEVRQKCRNIEDICISCGSLNVTLEHPLFIGGMCQNCKNCFLECAYQYDDDGYQSYCTICCGGREVLMCGNNNCCRCFCVECVDLLVGPGAAQAAIKEDPWNCYMCGHKGIYGLLKRRDDWPSRLQLFFANNHDQEFDPPKLYPPIAAEKRKPIRVLSLFDGIATGLLVLKDLGIHIERYIASEVCEDSITVGMVRHQGKIMYVGDVRNITRKHIQEWGPFDLVIGGSPCNDLSIVNPARKEGTGRLFFEFYRLLHEARPKEGDNRPFFWLFENVVAMGVSDKRDISRFLESNPVMIDAREVSAAHRARYFWGNLPGMNRPLVSTINDRLELQDCLEHGRLAKFSKVRTITTRSNSIKQGKDQHFPVYMNEKEDILWCTEMERVFGFPVHYTDVSNMSRLARQRLLGRSWSVPVIRHLFAPLKEFYACV

**Multiple alignment**

>Acar_P_008105653.1

---------------------------------------------------------------------------------------------------------------------------------------------------------------------------------------------------------------------------------------------------------------------------------------------------MSSVFTAQEELDVIVVDSDSDLEEVDLPMETAVLL------------------------------------------------------------------------------------------------------------------------------------------------------------------------------------------------------------------------------------------------------------------------------------------------------KNRSLSDVISVSSVEEMLPSSP-------------------------------------------------------------------------------------------------------------------------------------------------------LGPNTIVFLETENSDDVISVKSVEELNPPSLSRGNIS--------------------------------------------------------------------------------------------------------------------------------------------------------------------YEVNSNRRSIEEICICCGELEIHT---QHPLFHG-GICAPCT---ETLLERFFLCDEDGSQADCTICCWGNSLMM-CDDPKC-HRCFCEECVETLVCPGHSEEIKDTNPWNCFLCVPNNRYGLLRKKTKWREYLKHFYDQE------SNSLQIYQPLSPWERK---------------------PIHVLSLFDNIT---KELNRFGF-LE---------------KNMGNG-------------RLKYLDDVKNVTRTHIEEWPFDFIFGSTPPVSMSYQDPPA----------WYFYQYMRILQYGRPPEGSQKPFFWLFLDNLVLDEEDRDTASRFFQ----------VEAVLRYKQHGEIIQNAVHV-WSNIPSVNS-------------------------------------------------------------------------------------KYSA-------------------ASFYMDLSQL--------------------------------------------------------------------------------------------------------------AKEILRTRIFSQR--------------------PATLIKEFFVPLKDYF---

>Cpic_P_008169122.1

------------------------------------------------------------------------------------------------------------------------------------------------------------------------------------------------------------------------------------------------------------------------------------------------------------------------------------------------------------------------------------------------------------------------------------------------------------------------------------------------------------------------------------------------------------------------------------------------------------------------------------------------------------------------------------------------------------------------------------------------------------MPATLSSP----------------------------------------------------------------------------NRDLIAHEVNENQR----------------------------------------------------------------------------------------------------------------------------------------------------------------------------SIEEICICCGSF--QIHT-QHPLFHG-GICTPCT---ENFLETFFLYDDDGLQAYCTICCSGKTLLM-CDDPTC-NRCYCLECLDVLVSPGTAEKFKAMNTWLCFMCLPLSSNGLLKRKKRWRAKLKCF------YDQESNHLEIYQPLSAWERK---------------------PINVLSLFDNIT---PEMKNLGF----------------LGSSMGDG-------------RLIYLDDVTDVLRTDVKGWPFDFIFGSTPPVGNSYEHPPA----------WYFYQYHRILQYGKPPESSQRPFFWMFVDNLVLEKEDRETASRFFK---------MEAVTIHREHAETVQNAVI--LWSNIPSVKS----------KYSLS-----------------------------------------------------------------------------------------------------DL-------DLDLDLL------------------------------------------------------------------------------------------------AKNILRTRLFSQR--------------------PATLVRKFFLPLREYFKCF

>Mmus_NP_001271126.1

----------------------------------------------------------------------------------------------------------------------------------------------------------------------------------------------------------------------------------------------------------------------------------------------------------------------------------------------------------------------------------------------------------------------------------------------------------------------------------------------------------------------------------------------------------------------------------------------------------------------------------------------MGSRETPSSCSKTLETLDLE----------------------------------------------------------------------------------------------------------------TSDSSSPDADSPLEEQWLKS--------------SPALKEDSVDVVLEDCKEPLSPSSPPTGREMIRYEVKVNRRS-----------------------------------------------------------------------------------------------------------------------------------------------------------------------------IEDICLCCGTLQVYT---RHPLFEG-GLCAPCK---DKFLESLFLYDDDGHQSYCTICCSGGTLFI-CESPDC-TRCYCFECVDILVGPGTSERINAMACWVCFLCLPFSRSGLLQRRKRWRHQLKAFHDQEG-----AGPMEIYKTVSAWKRQ---------------------PVRVLSLFRNID---KVLKSLGF-LESG-------------SGSGGG-------------TLKYVEDVTNVVRRDVEKWPFDLVYGST--QPLGSSCDRCPGWY--------MFQFHRILQYALPRQESQRPFFWIFMDNLLLTEDDQETTTRFLQ---------TEAVTLQDVRGRDYQNAMR--VWSNIPGLKS----------KHAPL---------------------------------------------------------------------------------------------TPKEEEYLQA-------QVRSRSK--------------------------------------------------------------------------------------LDAPKVDLLVKNCLLPLREY---FKYFSQ-----------NSLPL-----------------

>Btau_P_010822784.1

-------------------------------------------------------------------------------------------------------------------------------------------------------------------------------------------------------------------------------------------------------------------------------------------------------MGPGPSGCSKPGEGVSEVSSNSLGSGAGG-----------------------------------------------------------------------HGGSVSSGWPGSFGRCLAAALEVST------------------------------------------------------------------------------------------------------------------------------------------------------------------------------------------------------RAPVEFWTFHCAGSFHSSPLPASN-------PERPGGGLRN-----------------------------------------------------------------------------------------------HRRAQSPPAFKSSWERPRLD--------------GIWETLGRQEHGVAEGPSAGGTQ-----TEEVPRRTPGPGLK------------------------------------------QDSRIPRLRDGGWWGTFSL-----------------------------------------------SAVSPISWFGVPLSATLVLDPEPE----HSLDIILVGS-SELSSPPSPGPRRDFIAYEVKVNQRDIEDVCICCGSLQLHT---QHPLFEG-GMCAPCK---DKFLECLFLYDDDGYQSYCSICCAGETLLI-CENPDC-TRCYCFECVDTLVGPGTSGKVHAMSNWVCFLCLPFPRSGLLQRRRKWRTWLKAFYDREA-----ESPLVMYKTVPVWKRE---------------------PIRVLSLFGDIK---KELTSLGF-LE---------------DGSKPG-------------RLKHLDDVTNIVRRDIDEWPFDLTYGSTPTLGHTCDHPPG----------WYVYQFHRILQYARPLPGSPQPFFWMFVDNLVLTEEDLDVATRFLE---------TDPVTIQDVRGRTVQNAVH--VWSNIP--------------AVKSR------------------------------------------------------------------------------------------HSALVSQEELSLL-------AQDRQRV---------KSPAQG---------------------------------------------------------------------------------PATLVKNCFLP----------LREYF----KYFSTELTSSL-----------

>Hsap_NP_787063.1

------------------------------------------------------------------------------------------------------------------------------------------------------------------------------------------------------------------------------------------------------------------------------------------------------------------------------------------------------------------------------------------------------------------------MAAIPALDPEAEPSMDVI---------------------------------------------------------------------------------------------------------------------------------------------------------------------------------------------------------LVGSSELSSSVSPGT------------------------------------------------------------------------------------------------------------------------------------------------------------------------------GRDLIAYEVKANQR----------------------------------------------------------------------------------------------------------------------------------------------------------------------------NIEDICICCGSL--QVHT-QHPLFEG-GICAPCK---DKFLDALFLYDDDGYQSYCSICCSGETLLI-CGNPDC-TRCYCFECVDSLVGPGTSGKVHAMSNWVCYLCLPSSRSGLLQRRRKWRSQLKAFYDRE-----SENPLEMFETVPVWRRQ---------------------PVRVLSLFEDIK---KELTSLGF-LE---------------SGSDPG-------------QLKHVVDVTDTVRKDVEEWPFDLVYGATPPLGHTCDRPPS----------WYLFQFHRLLQYARPKPGSPRPFFWMFVDNLVLNKEDLDVASRFLE---------MEPVTIPDVHGGSLQNAVR--VWSNIP----------------------------------------------------------------------------------------------------------------AIRSRHWALV-------SEEELSL---------LAQNKQ-------------------------------------------------------------------------SSKLAAKWPTKLVKNCFLP----------LREYF----KYFSTELTSSL-----------

>Apis_XP_029348651.1

------------------------------------------------------------------------------------------------------------------------------------------------------------------------------------------------------------------------------------------------------------------------------------------------------------------------------------------------------------------------------------------------------------------------------------------------------------------------------------------------------------------------------------------------------------------------------------------------------------------------------------------------------------------------------------------------------------------------------------MFIDAEKQQGQVVWAKMGKKSR-LWAAIILSA------------KEMDLVYFEDKPGKTCVWWLGDDTMSQLQNDLIFEFRSNLKKGIADLIYARK---------DNFEQAIYILSLQFNFE------------------------------------------PKHGLVKWALVNLPIIMKG-----------------------------------------------------------KNSRKEEFIIPDQFLKNNRIKRNIEE-NKVKNVESSPHGEKMKIDVSQISKME-NKYCIACRFPSDELKI-KHPLFKG-LICNDCFINGDIILSIAKDKSNDG----CCVCLTQKDTLVLCGFCIR---AYCNECLEFYCGQKALEKILIDNAWACIACSSTT-------------------------------LKISNLVPRSTKE---------------------QLRNIYMLYPANAKKDCAKEQYKEIHVLNLKDIQNNVPKVLTLGFPF-------------KILETSYLNDNNQDNNKS-NAKLVVQYYPNRENYDKDKFFDRND------CLKKFFLFLLFKNKMQLQKDDNLFWAFETSAAIKVQEQKIISRFLE---------IEPIIIGMDTDD-VQQRSRFI-WTNIT------------------------------------------------------------------------------------------ILN-----------------------KDIRKF-------TNQNIYL-------------------------------------------------------------------------------------------------HEIPKSIGRKSK-KDKRNIDMPWCY----TSIYIILSSFINNDIMF-----

>Ptep_aug3.g9131.t1

-------------------------------------------------------------------------------------------------------------------------------------------------------------------------------------------------------------------------------------------------------------------------------------------------------------------------------------------------------------------------------------------------------------------------------------------------------------------------------------------------------------------------------------------------------------------------------------------------------------------------------------------------------------------------------------------------------------------------------------------MRGDIVW--AKMGGFPWWPAIVIDP-------------KDCGRDDDNNEEKLWLFWFGDYKVSQMPLDKINDFKEEYDTHFLNGKGKNF------------NRAVQEALTVLAEN----YNENSL--------------------------------KSSNLFDWAAKGFENQGLFLPD--------------------------------------------------KVKGLPKMVSESLNRK------AFKRKLINDS-DQEDDSDEDTMEMENLLPDVLNGIKNLEDICISCFKPSACVVK-KHPYFVG-GFCRTCQRIVEIACDIPFGELQDS----CAVCGQGGHLII-CSNSMC-LKSFCSLCIEYMTPKGTFKKVLRATIWLCYLCELDSKLIRPRRLVTSHQNVVIPILR--------------------------------------------KKNLINVLAHSDTILSVLKSKRF---AIFSTEIPPLDSSKFKFNKFV-----------------EDELENLSPD--------FVCCSYLEDPLNLEFPNGDVHL-------LFKTFYSFLYTLDEAAVNERVLFAFLLNAKKSNYLVRERLSHLLE---------VPPVAL-------TTKRKSLYLWS--------------------------------------------------------------------------------------------NILN-------------------------------------------------------------------------------------------------------------------------------------------CSCLSKVFQ--EQFADVSS--------------ESDFFAPVIELLSKYFKCV

>Acas_ELR21928.1

------------------------------------------------------------------------------------------------------------------------------------------------------------------------------------------------------------------------------------------------------------------------------------------------------MLYPWVQLLKWEGIAPFGPNLAADHAQSMRAN----------------------TFVPVAHVLFVVESSRPERGWTRFV----------------------DPTGTVPAGTTDSDVAAKLVVGAAVKLTNVR-LVPDSAFCGRLGIVIAWENLGRVVA----------------------------------------------------------------------------------------------------------------------------------------------------------------------PKSKPGKPHTNNISTFKETTPF--SIYTSLEGVKKDEVADI------------------------------------------------------------------------TYRQFRAEW--------TRRHPSPTAPPPPPPLPTHTTATVSSGDESFSSPSKEVLQQQPVAETVDALHHLVEEEEEDAGESDLSQPSDLPSSQPSEGDSSAGQRRTSA--------------------------------KRRYLKRVESDPTLPDSQPSPQ-------------------------------------------SPRAFPRAEESETAKHDDVDGSDDDEEERTAKRTKAKAK-KVTKAKAKAKSEAEDELVKRKRGRPKKEAIETEEERIKKHRKSEEHKKKFVELFIKHAK-----------------------------------------PKVVMTPVV-------PLDEQARQLLDRCEFQQSQR-LSQHQSQRAKSQRIGDNENESEDEDEAEAEADSEEDLTQDINQL--------------------EVVLTQCKQLCESALRALKLLGIRVKAYFVVEKDPLAMDVVRANFPP---------EEHPYIRYLGCVEKITTADLEDIPVDLFIGGSPCTDLSVLGKKKGLDC-PEGSSYHFYDFLRILQDLRRLNSDSHNVFFLLENVASMSRKDKTKISDCMG---------VEPVRIDSKDIT-NVRRNRYY-WHNIP-RNINALPKATFQELLSQD-------------------------------------------GEALVLVGGCITTHNATMEYLK-----KSLE--------------ELLARPQDLKRYNTI-----------------------KIKGQD---------------------------------------------------------------------------KCRSLKATEMERILGFRERYTKEGRLTKAQRNKLLGQSFDTRVIAHLLTPLLK--LCA

>Pbac_3469897

----------------------------------------------------------------------------------------------------------------------------------------------------------------------------------------------------------------------------------------------------------------------------------------------------------------------------------------------------------------------------------------------------------------------------------------------------------------------------------------------------------------------------------------------------------------------------------------------------------------------------------------------------------------------------------------------------------------------------------------------------------------------------------------------------------------------------------------------------------------------------------------------------------------------------------------------------------------------------------------------------------------------------------------------------------------------------------------------------------------------------------------------------------------------------------------------------------------------------------------------------------MFPPDYTICTHPKPR---------------------------------------SPPHNPWFQHETSPPSLS-----ELLNGKSKHFSYVQR----QEGHVFWLFENVTSMSADDRTTISRFLQ---------CSPLKIDAKHFT-CMNRSRLF-WGNIPELKRPIVP-VAN--PVTLQ----------------------------------------------------------------------ECLE--------------TRCGRSARVEKINCV-------TSRKRTE---------TAPAPV-----------------------------------------------------------------------LMEGQTDGLWTTEYLTPTPFLVGSIWAGTLYGRRKHEAPGKELECPCYEAYGTIFPD-----

>Pdum_DNMT3_prot

----------------------------------------------------------------------------------------------------------------------------------------------------------------------------------------------------------------------------------------------------------------------------------------------------------------------------------------------------------------------------------------------------------------------------------------------------------------------------------------------------------------------------------------------------------------------------------------------------------------------------------------------------------------------------------------------------------------------------------------------------------------------------------------------------------------------------------------------------------------------------------------------------------------------------------------------------------------------------------------------------------------------------------------------IETTIGSVENPQKQ-ASPFHSCPVFCRSSYWR---------------LHLLLKICPLQRSVWPYCSPSSS--------------------------------------SDPIKAISDWCILPEGVLELIIFQICKMAAMEIVRDEGAQQ-----------------------KIRVLSMFDGIGTGRLALKTLQFDVGAYYSCETDVDANLVNMIRNPD--------------IIPIGDVREINEKVLKCIPIDLVIGGSPCDEFSIANPWSLGFI-LGRTGSLFYDFVRVLYKVLE-MNKDRRVYWLFENVRSMSEEHRSIMSFFFFD--------QLPTIWDASVLT-PQNRKRYF-WGNIPGLKGGWES------CRIKS----------------------------------------------------------------------PVVNDVLCP------------FRKSHVKKVNCL-------TTNVQSQ---------EHSGTTFVKPVRTIT-----------------------------------------------------------------DEESQFWSVERERLFGFPDHYTDVAGISASSRHDMLGQAWSLPVVTTILSPLKGRFMLP

>Dpul_320126

------------------------------------------------------------------------------------------------------------------------------------------------------------------------------------------------------------------------------------------------------------------------------------------------------------------------------------------------------------------------------------------------------------------------------------------------------------------------------------------------------------------------------------------------------------------------------------------------------------------------------------------MNSDSDDCYDSDATYFCGSDDDDG----------------------------------------------------------------------------------------------------------------------------------------------------------DGNFGVEA--------------------------------------------------------------------DGLLLEKWIPPLFYLAKGH------------------------------------------------------------------------------------------------------LQTIHNDVNKLENVCLACWEKG---TC-PHPFFVG-ALCQQCK---QRLLRTMFSKTVDGFHLYCTICGSKSNACVSCSNGKC-YRKYCVNCLNI-WTDHSDKLIKNSDKWFCFLCLPEP-NQILQAHHNWAQKVLLFHEPLAVYA---------SRALYWKRQ---------------------PLRVLSLFDGIGTGLVALRKLGIEVEVYYASEVLTAAATVSRTRLGG-------------VLHHIGSVGEVTQQRLEEIPIHLLIGGSPCNDFSAINRFPKDFYDPRGYSRYFFDFVRVLNLMRKINGQHQHLLWLFENVASMPQHYRETISRHLD---------CQPAVIDAKNFS-PQLRRRLF-WGNIPGLFTVHEQLTQDGESLSLE----------------------------------------------------------------------KSLM--------------PNSGRRAAQEKIRTL-------TTNTNSL---------QGRTEN----------------CDEDDN----------------------------------------------------LQTDVLWLQEIEHVFGLPRHFTDVGNMSRTDRQKLLGHAWSVPVIASIFSNLKAYTV--

>Hrob_162653

-------------------------------------------------------------------------------------------------------------------------------------------------------------------------------------------------------------------------------------------------------------------------------------------------------------MFTFAGCVIDIIEEIQSSLLGLRKV----------------------SINVQKSDQCHKDN-----------------------------------------LEKNFQSPEKKKFKSSQLAKKSL---------------------------------------------------------------------------------------------------------------------------------------------------------------------------------------------------KKFKCTLKSSLLLCDQESSYD----------------------------------------------------------------------KIFEIGQIVWARLYFCS--FWPAVIIDG--------------AMCGQKQASHDNYWVFWFGDHKVSEVTLNNVVDFRTTFDQNYNNKNFKKNYEKGVSEACKLYYSRIGKNADSDKIA-------------------------------------------LNWISLYDTNDEHA------------------------------------------------------DNMLKETDLSIPDWIIKILDEYSKQDEEDDDVNSN-HSNHDCRVKLNKDIDSNTANQETWFNWKSEKGACLSCRANIGAYNHPFLLG-KLCITCK---NRLIDNIFVFDTDGFNMFCVICGQAGEVFL-CDKLGC-NKVFCCRCMTDILGKSQVHAIKSGEMWTCFICDPTSHIGCNGVVENWKEELNYMFQPN-------------PSILPRTRR---------------------RMKVLSLFDGISTGKVVLDQLDVDLEIYYASEIDEDSLLVSKGRHGGG------------VVHWLGDVTNINSEMLEKIPIDLLIGGSPCNEFSVANPGRKGLS-EDGTGVLFFEFYRILKELQALMTPDRPLFWLFENVSSMQKHCKATMTRFFM---------CHPATLDAKYFS-AQNRSRLF-WGNIP----NMYCPPQYNKNLAP-----------------------------------------------------------------------DLLN--------------LFPGRRSQVVKLRTV-------TTKQNSL---------QGCRNEMPVSM------------------------------------------------------------------LDNNEESLLWIPELERVFGFPDHYTDIGDLPRTRRQKLIGQAWSVHKLELVLIYIRFCLQLN

>Caqu_CAQU001464

------------------------------------------------------------------------------------------------------------------------------------------------------------------------------------------------------------------------------NQTNGNEKEVVLPDDPPKEEEQPAKEKISGQPRPKSHPKKRHWESVRGNSPEPPILTPIEKHEPDLPENEENKEEKDPSLTHRPPRLSIAVPKRRHIAKKSSAKSLE-------------------DVTDKNSMEKEVSDAKEETEVPKKG----------------------NGRGSNGWEDSLRPRPSQVDHLQSFMLQSILIRKPKKKKYSKASKEKKKSKQKPATKTVERN---------------------------------------------------------------------------------------------------------GKAEQVPGHILKELKIPIMKVHVVDKDCLVPGPIDPVKSKEGDQIGSNGEKVCKEQDAAALPNNNNATECLPCPTNTPSDKSAKPSDQESAQM----VFTCSSKESGHSAVKKRKKSSHSSGPDSKKLKKKSDKGRKNKKKS--------------------------------------------------SSSLSPLPASEEGKKKHKEKSHKKKDSKKVECDKLVGFTSHFKRKYSKCSFKTFKLALKEALRECANRADPERAGG------------------------------------------NYAELLSWASQGFLSEKGP-------------------------------------------------FDAQRFQPNPDNPVPEIVVGHLRKIKHEALPSVEDP-VQLEVPVESVSTKGDAMSKVREGTLKIDEICIACTTTSAEVVG-PHPLFVG-GVCGKCQ---DGLIETLYAYDDDG------------------------------------------------------------------------AKVLQLFNP-------KNQLLIPNALAFEEKR---------------------PMRVLSLFDGISTTLHVLEKLGLEVEAYFSCEVDPDAIAVSTSHHHQ-------------RVQQLGDIEELLEVQVQKLPLDLVLGGSPCNDLSLVNPARRGIYDQQGTGKLFFDFYRVLKAAQI-GNQGRHLFWLYENVASMPLEYKKVINRFLG---------CEPAVLDARFFS-PQQRVRYF-WGNIPGLHTPLQP---H--LLSRR----------------------------------------------------------------------VSIE-----------AALPKLVPERATSPLNNLMP-----ASQQAIL---------TGDGDS-------------------------------------------------------------------------------MKVTELEESIGLPKHYTEVGNIPPGKRQKLLGKAWSVPVVAHVLKPLRNFFICA

>Lgig_P_009055961.1+P_009055960.1

--------------------------------------------------------------------------------------------------------------------------------------------------------------------------------------------------------------------------------------------------------------------------------MSSRRQSTFLSHLQNTRIPVKYVNVVKRKTCQYNGPQTDSADSLSSGSTQFIKRKNN-------------------KMNRKKEKHCRKLMHMLHDVQDSIR----------------------RATTKLMEQPTDTETPSSSSPDRQSNLSNLI-RYRYPSHKHLLKRLHRKCKNRYYTMAQLARKLAGSESDDSTDISLDSDDDTGIQITDIQGKQVYSVQNTKAMDNMLPLTINTNQDEGIEFNRIDEPVHDLNAVRIFESS--------------------------ESGSEEDKNTELPLQPHMKMVKNKTNKTSFVIGPMRDDSLRPNPVKRQLFQVLNEQPRKRNKMEKKREPTEQYNALSNTP-----------------TSSTGKMKKFHIEVLEDDDDDFIEIESQ-----------------------------MGRLVF--ANLVGLQWWPGIIVKG--------------SMCHLYTENKGCSWIFWFGDHKISLVQQERIIPFAANFI--------EKSSGKGKMF-----QKALSEIIKVYGER--IGLNACTM--------------------------------SNEQLIEWAQTEIRLIKGS----------------------------------------------KQSEAETDKKIPDWILGKLEEIGADI---LKKVEGKPLP-KSKRDKMEERSATENAIDAVKEGKCEIKDICIACADSSAAIVC-QHPLFKG-GVCKKCK---VDILETIYALDEDGVMAYCSICGHGGTVFL-CDKVGC-NRVYCVECIDEMAGSIILTEIEQKKNWQCFMCTEFDIHGLIEPNKDWQQRIIQFFETEMVHEMFKTKR---------------------------------PIRVLSLFDGIGTGILVLRELGMNVEEYYASEIDSDAMIVTSVHHGD-------------IVQQIGDITEITEEIISSIPIDLLLGASPCNDLSLANPERKGFN-FGGTGILFFDFVRILEIIRKLQDKQRHLFWLFENVASMKLEFKKVISRFLK---------CPAAMWNSSYFS-AQNRSRYF-WGNIPGLFS--TPSSYSHEKVMLD----------------------------------------------------------------------NFLT--------------KKCKRQATVDQLRTV-------TTRTNSL-------RLGKDDKGFPIKMD--------------------------------------------------------------------GQDAGIWIPELERVFGFPSHYTDVQNLPSTRRQKLLGKAWSVTVLKNILKPLKQYFQCS

>Cint_P_018670748.1

-----------------------------------------------------------------------------------------------------------------------------------------------------------------------------------------------------------------------------------------------------------------------------------------------------------MCVVRAKLKPVMPLLTLGVWAASSFSWFR--------------------FLFCKDLLFSVFLYKIL------------------------------RLWDIISLNMSRKKERRSKKVIVISSESENEKLKPSSTLEKQYLLTN--------------------------------------------------------------------------------------------------------------------------------------------------------------------------------PTQSNHETQNNNNCDVIDSIVQTLKAKLSKKHKKKKKKRRR--------------------------------------------------------------------------------------------------SRRLSNSGVEFSETLINEYNPSRGLRKRRKSVELFQADTSCISKHKKRKSTSFNEVQDVDNGIQYLLHNTPVV------------------------------------------GVNQLENGTEEEEEASIEFNP-------------------------------------------------NIDAATQLPIEEITILDDVEVCGEPPLYDEGLNN-KSKHDVGLYFEQKQKVFRKVIEGVLDINDICLTCGSS-ENITD-DHPLFQG-GLCETHK---TMFQDWFFLCDKDGCQSYCTICCMGEKVVL-CDNEDC-GRCFCTDCLDLMVGAGQYDKLVPLDKWSCYVCTNDSSCSLIVKREDWQEKLR-LLHPEAKYQKLVDVP---KPMSVGERK---------------------PIRVLSLFDGIATGVVALKQLGLQIQKFVASEIDEAAIRLVQNRHPE--------------VMHVGDITKLTDDDITRYPFDLVMGGSPCNDLSGANPRRKGLLDQEGTGCLFFDFFRVLRAAEPPCDHQRPFFWLFENVLSMKMVERERISRFLQ---------CQPVVANGRAVS-AAARPRLF-WGNLPGLQSTLTPSPGD--RLTVQ----------------------------------------------------------------------SCLE----------------HGRLATIEKLNTI-------TTRSHCL---------QGHTKQ--------------------------------------------------------------------APVMHMGKEDVLWSTEVERIFGLPEHYTDVNNHGPKERLALLGRSWSVPVVKRILAPLRDYFACG

>Nvit_P_008204446.1

----------------------MPVTNLGDDDLMSIHISHGSYWSGSLWREMMAMDLFVDHNYCKL----------------------------------------------------------------------------------------------------------------------------------------------------------------NKSNVKIDKAENYDNFNISQENILAYKINNNELMEFDIQNYVEIQCNDEMQNDISEDVINFNSINTENRYFNNDATRLNCNEIAKDVNFINTLEDMNFNNISVNS-----IATENEHSMN--DSMANSGSQCLINNLPLNNHNSTFT----------------------EDSGIESMDSLLFTESEDSFLLDSRDDHCYSSKERDGAKKSYFNKRKAESSSAASISTNIENSDVIKDKSEVPAKRNRTTKRSREVIISEESPIIEGKYEESRRVTRLLVKNSDEKPVKYIDNTPGKLVWGYFRSGWWPDLNIVTA---------------LIIRAEDAGMIPSSEKIWVSWIGESRISELNAKCIDKFSNHLERRLDNLATNSKTKVTCKKQKDEACFKTIQLLKKHFTGGALVKPPYKNKLDELHFYPYPES-------LSDKLNNLKIVNSEKNEKYLRQQEKERLCKAFEPKNLIIEKGGINIVDQKYGMIVW--AKMQGYSWWPCVIMDY--------------QHLNRKQPHVAHQWVMWYGDYKYS----QVQYRQILTFPTGMDRMESKITATKDELF-----CKAVLQAAKDYCDK------------------------------------------LGYLTEPWKIKDVIHLFYK-------------------------------------------------------SKDIYKLKNAELTEPNEEDLKKQLRKQINN-QPISEERKKKILECKNLNLLLSGKLPLESLCISCLESGEELE--DHPFFHA-SMCEKCL---EDFSPKIFAYGND-AKCFYCTLCGGDDLVAVCDSMSC-PRVFCTACIKYIICPEFYEDILLKHPWYCFLCDPSSINVVIKIRNDWRYKMISLYRINCDEEAP-------SNLERLDRNR--------------------KIRVLSLFDGIGTGLVVLKHLNVNIECYYASEIDPDSMQVSFFNHGN-------------EIIQLGDVRNIDEKKIKEIPIDLLIGGSPCNELSLANPKRRGLDDPEGTGILFYDYVRIMKLVKK-HNKKRHLFWLFENVASMPKKFRNQISKNLG---------REPKFLDSADFS-AQHRPRLY-WGNLP--------GPYQVNNVVLQ----------------------------------------------------------------------DVLR--------------KRCNRQALVKKIMTV-------TTRTNSL--------NQTKENLKPVLMD--------------------------------------------------------------------GKKDMLWVTELEKIFGFPMHYTDTN-LQKTRRLQLIGKAWSVQTLTAILRPVFLF----

>Smar_SMAR006764

------------------------------------------------------------------------------------------------------------------------------------------------------------------------------------------------------------------------------------MPLTERVQGSDNMEDDNNQADEEEDSEDYEFDASVPAALEKKIEVDDVWEGEPSNNTDEEDCNGFRSRTTTKVNGTDGDIVAPKKRAKAALVRRVRRRRRRTKEKAKIIKPKKKHQLSCV-----------------------------------------------PKRGQNGWEDSLRPRPAQAQHYQKPDEFLEK--------LQPLPMTKKEICVEELVRDDIDE-----------------------------------------------------------------------------------------------------------------------------------------GMIDETNESSLLELGDLGGKKRGLRGMKRNRKRRGLLMNGDTQQEDAENRSNSRKQVPNGVVVPK----------SVAVVPKPEGSEQDSETEPR---------------------YVSDASVGEVVW--AKLGSTRWWPAILIWG--------------SDCGQQPAHAGNTWIFWFGDHKISEIPRDRLADF----TVNYNRYYSGGGSKAFHCGVIEAIRECAARAKVDISDS------------------------------------------DSNALLEWAEKGFISESKSEGV-------------------------------------FLPDPNNSIPANIRKHVNKIKEVNLLYFQ------GVDTKGHQVA-PARTPRNDCLDNKDSGLRKVKEGLIKIEKVCIACDSDRVPITS-QHPLFVG-GLCKQCK---DDIIETMYAFGEDGTNAYCVICGNAGELLI-CDNNDC-NRVYCTGCIEIMVSPEACKKVLETSPWLCYLCTEYNPNGLIIKKDDWQQNITQLFQP-------EKHVQIPNIEDYKEKR---------------------PMRVLSLFDGIGTGRYVLDSLGIDIEIYYSSEIDLDAVNVATVQHYN-------------KVVQLGNVEELTDAEIAKLPIDLVIGGSPCNDLSLVNPARKGLYDPTGTGKLFFDFFRILKAVQL-ANMGRHVFWLYENVASMPQEYKVTISRFLQ--VPPTTISCDPALLDSKYFS-PQNRARYF-WGNIPGMYTPLQP---------------------------------------------------------------------------------HMLQ--------------------------KNITLDNVLTNLNRKAV-------------------------------------------------------------------------------------------------------------VSALND-------LRVG----------------------

>Lpol_P_013775600.1

----------------------------------------------------------------------------------------------------------------------------------------------------------------------------------------------------------------------------------------------------------------------------------------------------------------------------------------------------------------------------------------------------------------MSPDKTRKKLPKDKLLGLVGAFNDNY--------------------------------------------------------------------------------------------------------------------------------------------------------------------------------------------------------------------IDLG--------------------------------------------------------------------------------------------------------------------------------------------------GKFFKRGVLEALQEMA------------VRAGVDVAEGDSVK----------------------------------------------LLSWAKHGFRTESRRTNP----------------------------------------WAPSVENPIPENVRRRLDRIKTQYLEA-----LLLLESQTSL-QPRFSRFAQLENSGSALKKVRERKLSIQDVCVACDSSSEDIVT-QHPLIEG-GLCQQCK---DDIIETMFAYGDDGTNAYCVICGQAGELII-CDNTDC-NKCYCTGCIDVLISPGTHKKVLAADPWFCFICTEYDPNGLIQPKLDWQQNVLKLFQP-------EKSIQLSPSSDDYREKK--------------------PIRVLSLFDGIGTGRYVLDQLGIEVEAYYASEMDHDAINISIVQHKC-------------NVTHLGQIEEISDAEVAKLPVDLVIGGSPCIDPSLVSSGRKGLYDATGTGRLFFDFFRLLKAVQL-ANKERHVFWLYENVTSIPQEYKSIITRFLQ---------CEPALIDSKYFS-PQNQARYF-WGNIPGMYA--VEEVDN--PLQPH----------------------------------------------------------------------------------------FHMLRVKLNSSLSSN-LNQEIEVSSNTMC----NNLPSESKDSLTPGNTS--------------------------------------------------------------------DDKDALRVTDIEQVFGFPKHYTDTGNIPLGRRQQLLAKAWSVPIMKHIFMPLRNFVTCT

>Lpol_P_013778629.1

------------------------------------------------------------------------------------------------------------------------------------------------------------------------------------------------------------------------------SGDHKRDLEKVQEEGEKGEEENSEEPLDLTGWKSQENKDGLLEEENTGDGKKMSCCYFGLEDEKDPEDIACLSPISKHSKQNRKRKKKRKNKSKISFEYKKKKKMR--------------------PKAKSKSNECELHFSSESADLRNELTHSCMSEESDSPSEQ-------VTGAIRDLSLNEHSESPQFSVGNAAKNKCHE--ESTSDDTRSSLVEQEIKAPVKETFDFLTEQKNFNPNEQRLGQRIEEGDQSIISSLGQKVE------------AKYTEDSTSSSQLEKTEDSEDQLVPFEISPRSTCKISNNYL-----------------------VKSSTIRHGSNEREECLRPRPSQSLVMTRKRRIAPSLLEGIIGYKQAKLNMHTITAKTSTSRRMGPINKPSYRNHSSKKSSTCSSSSSSQITVAL------------DIFNPEDRELMSDSSNKEMDQDSAQ--------------KLLDASVGEIIW--AKLGSTRWWPAIVIWG--------------TDCGQPPAHPGQTWVFWFGDHKISELPRDKLVDFVEEFSSKFSDFAGKMFKRGV--------VEAIREVAVHAEVE-------------FVEG-------------------------DSAGMLNWAKNGFRTESSKPNP----------------------------------------------YAPDIATPISENVSKHLNRIKTQYLQLQSFGSQNSL-QPRFSRLEQLENKDSALKKVREGKFRIQDVCIACDSTLVEIST-QHPLIEG-GLCKQCK---NETVETMFAYGEDGTNAYCVICGQAGELLI-CDNNDC-NRCYCTGCIDVLVSPGAHKKVLSTSPWLCYMCHDYDPNGLIRCKEEWQQNVMQLFQ--------PEKHVHLPPSPEDYKEKR-------------------PIRVLSLFDGIGTGKFVLDQLGINVDAYYASEIDQDAINVSIVQHKL-------------SINQLGRIEDVTDAEVAKLPIDLVIGGSPCNDLSLVNPARKGLYDPQGTGKLFFDFFRVLKAVQL-ANKGRHVFWLYENVASMPQEYKSTITRFLQ---------CDPAMIDSKYFS-PQNRARYF-WGNIPGMYA------------------------------------------------------------------------------------------------------------------------------------------------------------------------------------------------------------------------------------------VFGFPRHYTDAGNIPLGRRQQLLGKAWSVPVIKHLFTPLKNFFATT

>Lpol_P_013786105.1

-----------------------------------------------------------------------------------------------------------------------------------------------------------------------------------------------------------------------------------------------------------------------------------------------------------------------------------------------------------------------------------------------------------------------------------------------------------------------------------------------------------------------------------------------------------------------------------------------------------------------------------------MNKDSFNSEDRELLSDISNQELDQD-----------------------------------------------------------------TTNKLLDASVGEIIW--AKLGSTRWWPAIVIWG--------------ADCGQPPARPGQTWVFWFGDHKISELPRDKLVDF----VDDFSSKFTDFAGKMFKTGVVEAIREVAVRAEVEFVES------------------------------------------DSAGMLSWAKNGFHTESQKPNP--------------------------------------------------------YTPDAGSPIPENVRKHALQSLGSQNSLQPRFSRMEQLENKDSALKKVREGIIRIQDVCIACDSTSVEIAT-QHPLIEG-GLCKQCK---DETVETMFAYGDDGLQAYCVICGQAGELLI-CDNNDC-NRCYCTGCIDVLVAPGAHKKVLNTSPWLCYMCRDYDPNGLIRCKDEWQQNVMQLFQP-------EKHVQLPPSPDDYKEKR--------------------PIRVLSLFDGIGTGKFVLDQLGIEVDAYYASEIDQDAINVCIVQHKL-------------SVTHLGRIEDVTDAEVAKLPIDFVIGGSPCNDLSLVNPARKGLYDPTGTGKLFL-FFRVLKAVQL-ANKGRHVFWLYENVASMPQEYKATITRFLQ---------CDPAMIDSKYFS-PQNRARYF-WGNIPGMYAPL--HHLLHQTISLD----------------------------------------------------------------------SVIT--------------PHLNRKAIVEKIRTV-------TTRSNSL--------RQGKKCLLPVTMN--------------------------------------------------------------------GESDVLWVTELEQVFGFPRHYTDAGNIPLGRRQQLLGKAWSVPVIKHLFMPLKNFFTAT

>Skow_P_006815704.1

-----------------------------------------------------------------------------------------------------------------------------------------------------------------------------------------------------------------------------------------------------------------------------------------------------------DNVIMTGGKYDHSIRRVVHGLREKPSKVE------------------------------------------------------------------------------KFQSPETKKKKKGKGKHEEK-------------------------------------------------------------------------------------------------------------------------------------------------------------------------------------------------QKHGLHRKECIAAVKIESNGQKNSVAATEESQNHSMNEMR-------------------------------------------------------FCIGTLVW--GKIKGFPWWPAVVISH-------------IEVEKPPPQLIDQRWVRWYGDLKHSLVSLDKLQPFHEFKPYYNDVIFKQQQSYRKGVYQALTMAAKRAERPVVTPDIDVTPLKTPRGMPIEMVT-------------------------LCLDLVNWAMEGFLPNGPG-------------------------------------------------------------------------------AIVPSK-AEKMRK-------------VVHGKKTLRDYCIACGDDDVIT---EHPLFEG-EVCDECR---QSILETAFLYDDDGYQSFCCICGEGRELVL-CSNDGC-FHCYCLDCLDCLVGRGATRKVRHNVD-----------NGLLKCRKDWSQRLHQFFSSDDNNSEQFESFPVYCPLPIEERK---------------------PMRVLSLFDGIA------------------------------------------------------------------------TGGTGC---------------------LFFDFYRVMRDLQV-ENANRPLFWMFENVASMRYEDKIDISRFLK--------------------------------------------------------------------------------------------------------------------------------------------------------------------------------------------------------------------------------------------------------------------------------------------------------------------------------

>Apis_XP_016662566.1

------------------------------------------------------------------------------------------------------------------------------------------------------------------------------------------------------------------------------------------------------------------------------------------------------------------------------------------------------------------------------------------------------------------------------------------------------------------------------------------------------------------------------------------------------------------------------------------------------------------------------------------------------------------------------------------------------------------------------------------------------------------------------------------------------------------------------------------------------------------------------------------------------------------------------------------------------------------------------------------------------------------------------------------MADNQSSDMEVDGNESTT-----------------------------------------------------------VY------------SSKARTSELPWLRSSSRAKT----------------------------SKLPRITSSSNPKPSKLP-------------------RLRVLSLFDGIGTGYYALLKLGFDIEVIYASEIDKDALMVTKYHFSD-------------NIKQLGSVTEITTKMLDQIPINLLFGGSPCSDLSGVNYRKKGLFDPKGTGILFYDYYRIWNYLSVKARENTPFYWLYENVASMEIKNKDTISKFFE---------CQPIVLDSLHFS-PQRRKRYF-WSSLPGITM--LPYATN--APKLE----------------------------------------------------------------------DYLE--------------KNLDRQANVEIVGTI-------TSKRSCL---------QDSKSRNPVCQD--------------------------------------------------------------------GQYTGLFITEIEAIFGLPPHFTDVGDLSISSRQKLLGRAWSVQVIIDILDMLSGLFAKK

>Cgig_P_011435619.1

------------------------------------------------------------------------------------------------------------------------------------------------------------------------------------------------------------------------------------------------------------------------------------------------MSEKEMKISPLAWLHREFSLPPSWTSQFVMQGKEKFTIFKF-------------------DIPTQARSYSDF-----------------------------------SLPNFLHDSCSSSAKSDPNSVSGPIDFPESA----TAAKLPGKARKSSRDRRFNIVADFASS----------------------------------------------------------------------------------------------------------GKSMRRKPKTDEFRQHRDSRRPFMYRRLKLQKMKLKTTLTNKKLQCHISCTTSLYSVVRRPKGSESSPQKKEGHSDERG--TESKSYKEKGGPKKS----SQGYSYDNRRKTRSSSESTASSMTDDVASSMTDDAMPSDSESSAEENQDSQVGNIVFASLKGSASISRWWPAIVIEG---------------KEVNKSEKSGFLWIFWFGDHRISQVMASNIGDFASNFTKFG---LCNKAVKNCPNF-----IKGINEAIQICAER--IAHNTEDM--------------------------------TEEELLGWAKEGFPRGQGH-----------------------------------------------------------FFPDPDDPLPQMVKTY---LTKIKDN-LKLVDTSKKRSPNKSAVKALRAGEKELSEICLSC-DDIENVEVEDHPLFFG-GLCKSCK---EEWKESIHIYDDDQTNTFCVICSTGGDLVV-CEDVNC-GKSYCVECIKNYLSEGQLKKIKESQVWNCFLCTLYNKHGYLIPRPNWRDKTVHIFDSGYRTDML-------PSFFKGPKQ---------------------AVRALSLFDGISTGKVVLDNIGLDVEEYYASEIDDDAILVSMVNHRD-------------SVQHLGDVRSLTREKLDKLRIDLLIGGSPCNDLSIVNPARRGLS-EEGTGHLVFEFGRVLEYLQSVRARGQHLFWMFENVASMRADIRDTISECFK---------CPPALWDAKYVS-PASRARYF-WGNIPGMYSPFLKNPDSIKDFHLN----------------------------------------------------------------------DVLY--------------LQNIRSSRYKTVRCI-------TGKRNSL--------KQGRNED-------------------------------------------------------------------QFVILMNGREDNMWITEIERVFGFPEHYTDVGNMSVTARHKLIGQSWSVPVVEGILSPLRKFFRTK

>Lana_P_013416790.1

PGPSFAPKQDTRAAPQSGAFSFDEAWRLLRQCIGNDLQKSTTWVTNSYKKTLKHLNCSESQDIYSIPPEKKHGYEYQGDHNYVKVTLPEKIIATDCSAGGHFSEQEMTNCSLTGLSQTDQHKVSILNGQDSFMPTRGRLRTRDLSQPKEKEPMAYKFRPTPAKVEKFAKVKNLHDGEHLSSCEVRIKRLDNKKHGDRP---VAEVTADQPIKTRHSSKTVTHIGQVVWAKFGGQNWWPGMIIKGAYCSVRPARPGSVWVYWFGDHLISELACKHVVPFTEEFSRLNNQCSRYRKGVIEAIKVCADRAEKDVSDFDEDQLISWARQGFRDVKCLFHPPKDHPVPQSVKEDKKKKDSVLDSVRNGTIRIEDVCIACDDQKEIVGQHPYFNGLCEDCENGGVLFVCDVEDCNRKSRVSIRLRRKMLKLLLSRPGPSFAPKQDTRAAPQSGAFSFDEANQR----KLLQQCIRSDLVNQKSTIWVTNSYKKTLKHPNCSESQEIYSIPPEKKHGYEYQGDHN------YVKVTLPEKIILSHPATDCSAGGHFSEQEEVAKNEQEEDHMDITSALTNCSLTGLSQTDQHKVPKRKSLVGQKELESILNGQDSFMPTRGRLRTRDLSQPKEKEPSITQLLKKQPERGKFRPTPAKVEKFVIDWKIKRLDNKKHGDRSQKGAELPVSDSLTEVTADRSLRKRHSSETVTHSTEFDGESDTDDIGQVVW--AKFGGQNWWPGMIIKG--------------AYCSVRPARPGSVWVFWFGDHLISEIACKHVIPFTEEFSRLNNQCSRKLY------------RKGVIEAIKVCADR-----AEKDVSDF-----------------------------DEDQLISWARQGFRVDGKV-------------------------------------------------------KHCLFHPPKDHPVPQSVKEYLAEENGEEET-GSREDTADKEKKDSVLDSVRNGTI-RIEDVCIAC-DDQKEIVG-QHPYFNG-GLCEDCE---EELRVTMFAYGEDGSLTYCIICGNGGVLFV-CDVEDC-NRVYCTRCIESLVGEGSTKVLSEKEMWVCYMCSEYAQHGLLQRKLDWQQNIMKLYQPS----STVQAPNISDFLPRR------------------------AVTVLSLFDGIGTGKVALDSLGIVTDKYYAAEIDDCAVTVTRANHGD-------------SVIQLGDITKLDREKLASIPIDLVIGGSPCNDLSLVNPARKGIF--DGTGQLFFEYYRILTMVKE-LNQDRHVFWLFENVVSMLREAKTTISRFLG---------CGPAMWDARYFS-AQQRARYF-WGNIPGMYS----APQ---TIHLQ----------------------------------------------------------------------NAVN----------VDGSPNCNRRATVKKIRTV-------TTRSNSL---------RQKYELFPVKMD--------------------------------------------------------------------GQDDTLWIPELERIFGFPAHYTDVGNLPVAKRHKLLGKTWSVPVIKHLFTPLKQFYNNN

>Hazt_HAZT003192

-------------------------------------------------------------------------------------------------------------------------------------------------------------------------------------------------------------------------------------------------------------------------------------------------------------------------------------------------------------------------------------------------------------------------------------------------------------------------------------------------------------------------------------------------------------------------------------------------------------------------------------------------------------------------------------------------------------------------------------------------------------------------------------------------------------------------------------------------------------------------------------------------------------------------------------------------------------------------------------------------------------------------------------------------------------------------------------------------------------------------------------------------------------------------------------------------------------------------------------------LDLLGVKVDVYFASEVDEAAMTVSEKHFHG-------------RVTRLGDVRHVTDDQIAAIPIDLVLGGSPCSDLSIVNPKRKGLFDPSGTGILFFEFVRILKSVAR-ANKKHPLLWLFENVASMPTEYRNTISKHLH---------SEPTAVDARAFT-PMSRTRLF-WGNIPGEEAMAASQLVACVVFDREVTELRCVTTQANSTTDIAGHYFPMRMRG---EKVFGFPEHFTDVGNLQPRQRQALLGKAWSVDVMPLKAMPALR----------IIISWCYVPQPDVDAVWIFWFGDHYVKSDEKVCAVTDFA---EDFNRGAAVQRGSLLQQALERECYQTGARLNDSPEDLVEWARGEATHRRASSAVDTLPQLPECVVSPFVRRHL-ARLYSLRRRRASSPSSAAALTTDDDEVPNADE-DTSDEERLPGPDWQLREVRALKRRVDDVCACA

>Ctel_188129

--------------------------------------------------------------------------------------------------------------------------------------------------------------------------------------------------------------------------------------------------------------------------------------------------------------------------------------------------------------------------------------------------------------------MTLFRSKDRGRKRKLPEIKHYS-----------------------------------------------------------------------------------------------------------------------------------------------------------------------------------------------LRERKVKKKEKREIEEKQEERKDVKKIKKEKKIRESPKKFKA----------------------------------------------MQSLITKETRIVGALIWCR---FGRRMWPGMLIEG--------------SLVGRPPSAVNTEWVFWFGEGTVSQMDASAMRPFIASFRDHYDPKYLSKSY-----------ERSIQEAVRVCAQR----AGKCRS--------------------------------SASDLLEWAERGFRGSASGPLH----------------------------------------DDKSIPEWISARLEKLKKWAQDISTDDDDDEEEEEEEEEEEA-DQRPLRSETYEKEKEIWDRVRDGSFSVTDVCISCGGTNEVTHC--HPFFIG-GHCASCK---QDFEESLFLYGSD----------------------------------------------------------------NISMPKDWNVKR--------------------------------------------------PLRVLSLFDGIGTGKLVLDLLGFEVEAYFASEVNPDCLRLTEVRHPA--------------IKQLGDVTMLTAEKLVEIPIDLVIGGSPCQDFSIVNPLRKGLMPWLYHGLLFFDFYRILNELQSLQQANRPLFFLYENVASMNKETKAVMTRFFK---------RQPNVWDAINFT-AQRRKRYF-WGNIPGLKG--GECVDDEDVTELK----------------------------------------------------------------------EVLM----------------PGRKARMSKVRCV-------TSRSNSL--------RQSKHNHFPVAMG--------------------------------------------------------------------NHLDTLWIPELERLFGFPQHYTDVANIPVSRRQVLLGNSWSVPVVKRLLLPLKNFFQTK

>Cint_P_009861613.1

------------------------------------------------------------------------------------------------------------------------------------------------------------------------------------------------------------------------------------------------------------------------------------------------------------------------------------------------------------------------------------------------------------------MWSMDKRARRKPKYFEAG------------------------------------------------------------------------------------------------------------------------------------------------------------------------------------------------------SGGKGPHENEKDPKQQYKTKPSPE---------------------------------------------------------------DVKFVPPRRIIPREDLLVWGKCRGFGWWPGRIANTGE------------LSGPIDPPDDPTRWIKWFGDEKFTETKVDDMAYL-----HDFNAYFKEKSYNRNRSY-----NLAVDLAIKEAAHR----------------------------------------SGSNASGITWAKEKFYPEGYLSIL----------------------------------------PKGYTDDQKRNMKGVKQSELNGS---------PEKKVKTTIS-KPEMNCQDFTKQRSEVFMKVREGLLDIANTCLGCGNLEVST---QHPMFVG-GLCQMCE---VDYRESCYLLDDSGYSCYCCVCGGGKRIFL-CDNQSC-CRSFCFICLELMMGEGTTKTVLHQSPWSCYLCVATE-KGLLHRRDNWHNRLSKFVSVGEVE---YEIVSLPKSPPAWERK---------------------PIRVLSLFDGIATGFVSLKQLGIEVKVYFASEIDHEAICVSEIRHPR-------------VVKHVGDVCEITDKMLSDWPFDLVIGGSPCNDLSIVNPARKGIWSDKGSGHLFFEYLRILNTCKVLAKEGRSLFWLFENVVCMDQSSRQTISHYLQ---------RNPIVLDAKEVS-PAHRPRCF-WGNLPGMRRPM--VATKAHKLTLE----------------------------------------------------------------------ECLE----------------PGRKATTDKLRTI-------TTKLQSV--------RGGHGSGTSNSLV----------------------------------------------------------------IQQRDQHDCLWITEIERVFGFPDHYTDVCNLPRTGRLRILGKSWSVPVIKHIFAPLKDYF---

>Obim_P_014770812.1

-------------------------------------------------------------------------------------------------------------------------------------------------------------------------------------------------------------------------------------------------------------------------------------MDNTAISTSPPVANDILTATALTDINITATTVSINNIETFEDSDEDVKLFDK-------------------KLHEGCKSNHTTDTKYLE-----------------------------TNGLLLEFHSKAKKRRGRKPKMVHPKSCDSE-----------------------------------------------------------------------------------------------------------------------------------------------------------------------------------------ELKEEIEQCLEKKKTKSCKRKLHSYQIPLSP----------------------------------------------------------------QKDLGNKCFPVGSVIW--GKLSGFKPWPGLVINHED---------------IQKKSSPLTVWVLWFGDYKISELPLSKVTRF--------DQYFYLNYKNPSKVF-----SVAVTEALEVCAQR--SGLKEDEL--------------------------------SSEHLFNWAKHRFMEMVEDKDP-------------------------------------------------------------FLPLKGDPYPQKTAENIEEGP-VRVSSPPNPLFENNDLINKLKTNQVKIQEICIGCKSSDKTIVA-QHPLFEG-GLCEQCK---ENILETIFALDVDNTHAFCSICSQSGQLCI-CSVPEC-NRVYCINCIQSWVGCDAWDLILSQNPWSCFLCRTPNKCGYLQAKPNWSLNIMHMFNSHQK----LAEPNLSYLTKCPERP---------------------PIRVLSLFDGIGTGKVALDQLGLTIKEYFSSEVDADALIVKRFTYGQ-------------SIKEIGDITELTEVKLSELPLDLVIGGSPCNDLSLVNPARKGFS-EEGTGILFFEFCRILSIVQRLC-KDRHVFWLYENVAAMKTSYKNIISRFLG---------CKPALWDAKFFS-AQHRPRYF-WGNLPTIYSSQ--PTTEDDSLYLD----------------------------------------------------------------------KILS--------------PNCNRKARVKQLRTV-------TTRSNSL-------RQGKNENILPVEMD--------------------------------------------------------------------GEEDAIWITELEKIFGFPTHYTDVRNLNHSRRQRLLGRAWSVPVIKFLLSPLTKLF---

>Ptep_XP_015906236.1

----------------------------------------------------------------------------------------------------------------------------------------------------------------------------------------------------------------------------------------------------------------------------------------------------------------------------------------------------------------------------------------------------------------------------------------------------------------------------------------------------------------------------------------------------------------------------------------------------------------------------------------------------------------------------------------------------------------------------------------------------------------------------------------------------------------------------------------------------------------------------------------------------------------------------------------------------------------------------------------------------------------------------------------------MCIACRKE--DAEV-KHPFFKG-GLCIACK---ENWMQTIFAIGDDGDGLFSDYPPDNHHLLK-ESSTVC-HKSYCLSCIDVFLGDKRRKKILETSPWLCFFCS----PGTFEKRENWEEELHFMFESS-------ASSKTCYPLKNTPIR---------------------PIRVLSLFDGIGTGKLVLDNLGIDVEVYYASEIDEAALCVSKLQHPD-------------AIEYIGDVTKLLSKKISSMPIDLLIGGSPCNDLSLVNPLRKGLF----TGCLFFDYYHILKTVK--FNKDRHLFYLFENVAAM------------------------------------------------------------------------------------------------------------------------------------------------------------------TKVNKIKTV-------TTKKNSL--------------DMPVE--------M-------------------------------------------------------K-----GIPDILWITELERVFGFPMHYTDVGNLSPTKRQELLGRSWSVPVITQILNPLQKLFKKS

>Mlei_ML35881a

----------------------------------------------------------------------------------------------------------------------------------------------------------------------------------------------------------------------------------------------------------------------MEDENDSDKANKDPSTFSVLNQTPLSKKKKRRKERGIANLSTLPHTYPLEIDEGAYVFLKGIEVWHGMQCYSFLDTSITVAARS--KLSSFLNYGCKVDFDLLESDCIASFAEIHRPDLLKLPD---------RDLMLLDWARLGFVGCLLIQSNIPQILVDYK----------------------------------------------------------------------------------------------------------------------------------------TENDLNQPTFCVDEEKPSSSRDWDEDDCTPTKRRKLSLHTEDSGYSSPVTPSVLKSPLATLSIDQLQSSSTSSDHNSEVG------------------------------------------------------------------------------CYVLGKLVRYGWWPGLIADPRK---------------SPRPCLPNERLVYWFGDCTKSLIDIKDIAPI---------TKFPDVYQPSKKGIYRVALYDFLSDAAESCNYT---EINAPDSVNIDES--------------------------ALTSLIKWAMDGFTPGGP-----------------------------------------------------------------------------------PRK-WAPSEHYHSIEKDRFTRRIEMAERTNNGSACVACQNRDSIH---PHPLFEGHQLCDACL---EDFSQCAYLFGDDGAGSYCSVCNEGGNLFI-CDQSQC-GRPYCPACISRVCGPHSLLQVEKSDPWFCVLCNDTGSEGDLLRRPDWQARLQDLLTRKVAGEEEYQTPLPPPFPPISERS---------------------SLRVLSLFDGISTGLVTLKNIGFQIETYVSSEVDEEAIKVSKLHHPE--------------VVQIGDIASLTPQKVSDLPFDLVMGGSPCNDLSNVNPFRKGIY--EGTGRLFFEFYRILSYVKE-SAGDRPVFWMFENVVSMSRSDRNCISRFLQ---------SSPNVIDAKHFS-CMSRARYF-WGNLPGMAR--TPVPLATDRLTLQ----------------------------------------------------------------------DCLE--------------QNCGREARFEKINCI-------TTKLTSM--------KQKKGRTLPVRIAGDEE----------------------------------------------------------------GPGDWLWCTEMERVFGLPSHYTDVANMSRFARQRLLGKAWSVPVLRHLLSPLRDYFMCQ

>Hmag_XP_012561137.1

------------------MNKTSVKAKRKRKSEVDSLNLSFIYSEKSCSLPKIDLINAKNDGIESLKTHGGRRWCLDSTLYCDRVIDHNSQKMEDHSQVNQQSNKDDKNKKNKRKSEIDALFD---------------------------------------------------------------------------------------------------LPNKELVNDENDKEWSLYNKIAHSSEIIDHDTPRTPEIAEQLQINKIFNNDANNNILFEGNKVKSKNKEQTQLIGGSKLVKKKNFKVGDVVICYFKYFGWWFAKIIHRAEGNCWVSSFGDHKILMVPLHSLRHHYDFSSKFNQTKMKKPLYQSNFKIMFGQKG----------YLNELLEWGLNGFAAHVIK------------KNKPCQGNLDFTNTSLNQSIDFVIESSDTSNTESSKLTSKDNSESIDFLLKNYSLSPNLEDSDNYFSSDQHSSINSFKNNEVDSKKSLCKVKNRSKINVLKSSIINHTAQSTNLECQNTVIKNSECENI-------KKNIENYNAQDWFLNKVDHVRKDCLEKMTHTIQRSPDKILSNIEHNDVTCKDFLCNSKHPSFHSNSTPLQTSCLKENSNQTMRLYKDGIKLDVTLLNKTPKRACSIKNQTLSSVSPNNVSCASITIGKKQKCVIENKYSSKKDREIGSDFFQIKIGDLVL--GKLKGYDWWFGMVVSH--------------RVIRQRPAANDCHWIRWYGDHKVSEVHLQNIELLTSFSNRYLPSKMQGLYLRAIKELLEEAARRCCKTDLPDDEKR-------------------------------------------LGVLVDWALNGFQPYGFD----------------------------------------------------------KLSIEETLTLEDDAFIQSETDSLTHNE-GEPLPSPILSEDVKALFDKAANGNMDLNKICLGCGDL--KCIM-EHPLFIG-GLCKECK---ESFMETAYLYDEDGSQMYCSICSDGREIIL-CDVPGC-YRSYCSVCLDMFCGVGYSRTVSAGLDWHCFMCTGEK-VRLIQRRDDWQQRLKNVFSVN---NEGYPLPFFYDPVPFEDRP---------------------SLRVLSLFDGLSTGYLALSELGLDILSYQASEIDPLAIKVSKVHHSM-------------RVEQIGDVQKITKQDIENWPFDLVIGGSPCDELSIANPFRKGIY---GTGQLFFEFYRILEYCKPQPLTARPFFWLFENVVGMRYTDRAVISRFLQ---------CHPVIINAKEIS-AQQRTRYF-WGNLPGMNRAMCPLPSD--RLKLQ----------------------------------------------------------------------QCLE--------------KDCGRVAKFDKVRCI-------TTSQNSL---------QTKAAMLPVKWTISRNSQL---------------------------------------------------------------DDGLWLTEIERIFGFPDHYTDVANMGQRDRQKLLGKSWSVPVLRHLFAPLRNYFSKV

>Drer_NP_571461.1

LNGLLQATFSQVEDTCSGAAFCQLMIQPGSIDVTKVNAEENLDILNNYNLLQEAFSKAQIQKELELTLLVNGDIMTTCDLLTWFKDMYDHNFCNPQVAFIKPEVVSKYNTEETSSNQKTQHVESFIRKYGSSTLTDDESNNVNSKDCPGQKSLLHGVELEDDKKASV-------------------------------------------------------FFDKETGENKIRLLDVVYPTKESTEDICNYILDTLRKIGIPLFNMAILYSDFPDHEHLVAGLQLMKAEILCGLTDLTGQVCHSGVEKIEFSDLILNLITEIYKHFPFDLALLEDVEGSDIDNLTSQSLFWRIIKKIPLAWSHLEKEAVCLLTHALQPLCDFQEIIDASVLQLLDASKLLRLYTQSFLRPKAAEYFHRRGKTSLV-------QETVGHLPKGEVAVGEQAADFLQQ---HSEELFDYLETFHSSIISFYTTVTVNIVKRLPLPDSTLRNLSLVLSPGKKLEVTGKMVQDLGVGFGVCIRPDNVSLLTDEFLEYQLIEGGDTGSVDQPTEKYWQTELRIMGNASNFGKLIVSLLALPKTLKKEIIFKQMFQQTDYLKMMRKEDCEEKDMMEDDVTDSSSYKSAPSHLSPETQTEMDEIGPVEIEDIAPMSETENQKVNVPHVSIVLDDDDDDDDEMTDDDDDYGCEAGEVMWKYEMTDNTYQGGFSVGEMVL--GPIEGFGLWPGLVQSW-----------------DSERPCGSMRKVIFFGNGMQTEVQADSLLPF-----SSLAKCFCSNSFATVMAY-----KDAIFSSLQVASRR-SRMFFSPESESKDE---------------------------LLRVMLNWAFGGFEPLGADGLQ--------------PQAEYSVKVKKGKRKNPTGKLTVPLNKIPESLDLNNGSVDLGTTDADKKRLYSKWNGRYKQRNKNIIP-TVQIESRQNSQKRHQMVHEFLKNKRKIEDFCLSCGSMSVDI---IHPLFEG-KLCTNCK---FNFTETLYRYDEDGYQSYCTVCCSGMEVIL-CGHDSC-CRSFCVDCLDILVCQGTFDQLKNVDPWTCYLCAPETSSGALKPRHDWSIRVQEFFANDTGME--FEPHRVYPSIPAIQRR---------------------PIRVLSLFDGIATGYLVLRDLGFKVEKYVASEIDEESITISMVNHDG-------------KITQVDDVKNITKKHIEQWPFDLLIGGSPCNDLSIVNPARKGLY--EGTGRLFFEYYRLLNVLKPKEDDPQPFFWLFENVTFMQTHVKADICRFLE---------CNPVLVDAVKVS-PAHRARYF-WGNIPGMNRPI--IASQNDKLCLQ----------------------------------------------------------------------ECLE----------------PGRTAKYEKVRTI-------TTRQNSL--------KQGTNDAFPVTMN--------------------------------------------------------------------GKDDHIWITELEKIFGFPKHYTDVKSMGRPQRQRVLGKSWSVPVIRHLLAPLKDYFACD

>Drer_P_005162096.1

------------------MPRRRSSRCPKQQIKNGKQNNAFTCNPATMCFIKRYASSTSDCGSVSKNPALDQKYWEDITAICRQTPYCLYLYIENDKKASVLLLG---------------------------------------------------------------------------------------------------------------------FFDEDTGKNKIRLLDVVYPTKESTEDICNYILDLLRKIGIPISNMAILYSDFPDHEHLVAGLQLMKAEILCGLTDLTGQVCHSGVEKIEFSDLILNLIIEIYKHFPFDLALLEDVEGSDIDNLTSQSLFWRIIKKISLAWSHLEKEAVCLLTHALQPLCDFQEIIDASVLQLLDASKLLRLYTQSFLRPKAAEYFHRRGKTSLV-------QETVGHLPRGEVAVGEQAADFLQQ---HSEELSDYLETFHRSIISFYTTVTVNIVKRLPLPDSTLRNLSLVLSPGKKLEVTGKMVQDLGIGFGVCS--ENVSLLKDEFLEYQFIDESDAQPADQSMKQYWKTELRIMGKASSFGKVILSLLALPTTLKKEQNFERLFQHTDNIKRKKLKDCKKHVSEDDVTDSSSYKSAPSHLSPEMLDEPMEVEDTLPVSDLDTESQNDTKGNCPNVSSNDDDDDDDKCSSSHDDYSSSDDDGDVIWREKEQTKNPYKGSYIVGEMVW--GPLEGFGLWPGLVQSW-----------------DSRKPCGSMRKVFFFGNSMQSEIQADDLTPF-----FSFAKCFSSNSFATVATY-----KDAIFNSLQVASRR-SRMFFSPDTDSMDE---------------------------LLRVMLNWAFGGFKPSGEDGLR---PQSECNELVFTPRSVNGNDGPSSNFKDHREKLTVSLNKLPESLDLHRGSIDIGKTHVYRKFMLPKRSLRHWKRYLNDME-SMKVDSRQNSQERYEMVHAFLENKMDIEEFCLSCGSTFVDI---IHPLFEG-SLCVKCK---FNFTETLYRYDEDGYQSYCTVCCSGMEVIL-CGHDSC-CRCYCVDCLDILVGKGTFKQLKNVDPWTCYLCAPETSSGALKPRHDWSIRVQELFANNSAMQ--FEPHRVYPSIPADQRR---------------------PVRVLSLFDGIATGYLVLRDLGFKVEKYVASEVDEESVTISMVNHEG-------------KITYVDDVKKITRKHIDKWPFDLLIGGSPCNDLCTVNPGRKGLF--EGSGRLFFEYYRLLNVLKPKEDDPRPFFWLFENVVLMENRVRADISRFLE---------CNPVLIDAVKVS-PAHRARYF-WGNIPGMNRPI--IASQKDKVSLQ----------------------------------------------------------------------DCLD----------------GGRTAKYEKIRTI-------TTRPNIL-------RPGIDDRHYPIVMN--------------------------------------------------------------------GKDDSLWITEIEKIFGFPKHYTDVKNLGRMQRQRVLGKSWSVPVIRHLLAPLKDYFACD

>Ocar_m.104173

-------------------------------------------------------------------------------------------------------------------------------------------------------------------------------------------------------------------------------------------------------------------------------------MLYKDAVMTALEIARCGKTFDVSDNEGTETEETETEDTPVNVSAASASATQ-------------------------------------------------------------------------------------------------------------------------------------------------------------------------------------------------------------------------------------------------------------------------------------------------DNSPSATQDNPPSVSQDSSPSPEKT------------------------------------------------------------------------------------------------------------------QITSPPPPVAK--------------------------------------EPLPQTQTQKPKSKSKKTKRLKPIR------------------------------------------VEQQLVDWALAGFPPTGPDGFQ------------------------------------PSKNEVDGGSSGVAEAEAVVYKEKSVRESSPGQTVKDLGGSQMENE-LDKRASCVSKEERERF-SDVRNGSRKIESLCLGCASLNPMA---EHPLFDG-GLCETCK---ECFEENCYLFDDDGTQMYCAICCDGREVFL-CDVMGC-SRAYCTFCIEKICGSEEVDKITSDSEWRCFMCEPTKSPHPLKLREDWQKRMQELFSSEQLE---CDPIPVPQPMSLGERR---------------------GIRVLSLFDGISTGYVALKSLGFVIESYVASEIDFDAIKVSKHHHLP-------------EINHIGDVKKISKKELEKHPFDLVFGGSPCNDLSVANPNRKGIY--EGTGRLFFEYYRIL------KDMPPPFFWFFENVVSMRSEDRQIISRFLQ---------CNPVVINAIEVS-PASRPRYF-WGNLPGMSRSL--VPLPGDRLTLQ----------------------------------------------------------------------DCLE------------------PSAQFTKLRTV-------TTKANSI--------KQTRDALFPVA---------------------------------------------------------------QLTPDGSEKGDGLWCTELERLFGFPDHYTDVGSMNKMNRQRLLGQAWSVPVIRHLLSPLKDYFKCN

>Scil_006106_1

------------------------------------------------------------------------------------------------------------------------------------------------------------------------------------------------------------------------------------MQQSTAAPDVAKLQAAGCGQEPKHVPLVVSHVATSSPPPPVLASWDAAAPGASATTPATTEVAEKVAATIVPDECVTPAAADGCNEKPGWSWEERAAQLAVSG-----------------EIHTGKPPPTPLSDQLSDHSPEVESGRAVQVTESKRHDLLGHNSRSISCQVPNRPPDSFSPYGEAAAAGTAAQFGRHA-EAAEHCELGSLPTRPVRIRKAPRTYISEMEKMDDGPGASKRRKRAIAESDAGGGGTVMNVEQPCSHAQVLLDSSYQKNADNGAPSVSAGAGHTSHSSPSSSTTTSTSTTASPVGCKGKAATDRRRSQASGLSHAAETQAQAIYSSNGVSQAELANSAGWGGNSSSNVDSDLQALHGRLTRSRLSKTRVDCEATASNDSANHGKPGADSSDDEDHGSSSTDRNAQTGGTPKKSTANPTGSERPRPHKRKWRNQYGSSEDATSDIGQARSASSTPALSTVAANERPDIDLGIGDFVW--GKMRGFDWWPGSIVSA--------------GRANQVSTTPGHTWVCWFGDNQFSQILLTRIRPLTDFRQYLLRR-------KPKSLY-----RRAIIECADSAALRANKQFPSEWPGTATVSTEDQSVLDN-----------------RQRHVLHWALGEFVPEGLDGLR----------------------------PSELDINRSASGQTLPSSPDGPLSPPYSTSPIGSSPLPSTPRSSVMVTPRQPQQ-MVQSDTRGSSGQQLERFALLRQGKVSLSNCCLCCGASGECVQQ-EHPLFAG-GICDACV---PAFIECAYCFDEDGSQTYCCICADGKEVYL-CDSPGC-ARSYCPLCIEKLCGADIHRHINKLDSWMCFMCDDQP-SDQLEVRDDWHARLLTLMSSEDSGLYQLEPT--PPPPAPEDRQ---------------------AIRVLGLFDGIATGMIVLNELGIEVAAYTSSEIDADAVRVSRRHHTN--------------IVHVGAVEEITSQRLKEWPFDLVIGGSPCNDLSMANPARRGIF--EGTGRLFFDFFRLLQLAKPAPSEQRPFFWLFENVVSMRAVDKATISRFLE---------CNPSVVDAKDVS-PAHRPRYF-WGNLPGMMRGIVPWPGD--VLSLQ----------------------------------------------------------------------DCVE--------------PNCGRRAKFEKLRTI-------TTKTNSI---------QTKQNLFPVSVERRGINGS----------------------------------------------------------LEGEDGDMLWCTEMERIFGFPAHYTDVGNMGRVARQRLLGRAWSTPVIRHLLAPLRDYFTTG

>Adig_v1.01094

-----------------------------------------------------------------------------------------------------------------------------------------------------------------------------------------------------------------------------------------------------------------------------------------------------------------------------------------------------------------------------------------------------------MEATPTSEAIQAPKKKPKFKRRLEFSASDYK--------------------------------------------------------------------------------------------------------------------------------------------------------------------------------------------------SKKKIRVKSDVFISVTSVEDNHLATSSDDHLSTSDDENG-----------------------------------------------------ATFVHGQLIW--GRLKGYDWWPGLIVSH--------------LEAQKAPPAPSNHWIKWFGDNKLSLLPFQCLRPFSKFKESLIPSKMRGIY------------KRAVFDSLEVAVKRSGKVFAKCPIKQTTQTKKKTATRRAQIAGLHEDQEPLGERE-RQEMMVQWAMNGFKPNGPK--------------------------------------------------------GFAPPEDDMISDTSVSVTSESTADHKSRF-GKPVNVPNLAENIKALFEEVSKGKRDLECICLACGDL--RVCA-KHPLFKG-GLCKECK---TSFLGNIYLYDEDGSQMYCTICGDGKEVFM-CDNDGC-FRSYCNLCIGMLCGVNAVRKIAACETWVCFMCSSKT-QGLISPRQDWSARLQEHFMND--KEQEFKPPHTFSAIPPEERR---------------------PLRVLSLFDGIASGLQALKELGIEIELYCASEIDENAVQVAKVQHGR-------------KIHHIGDIQKISRDQIQDLPFDFVFGGSPCNDLSIANPIRRGIY--EGTGRLFFDFFRLLEYARPKSELERPFFWLFENVVGMRAEDKAVISRFLE---------CNPVVVDAKEIS-PAHRARYF-WGNLPGMNRPTIPLPGD--KLCLQ----------------------------------------------------------------------DCLE--------------PNCGRQAKFTKIQTL-------TTNANSM---------QTKKALLPVQY---------------------------------------------------------------VASDGHEKEDILWITEMERLFGLPSHYTDVSNMGRSQRQRLLGKSWSVPVIRHLLSPLKDYYKCE

>Bflo_DNMT3_model_PK

EPSGNLHNNMEVDDDATPDPNSLATNSDASSKPPHNDPRTSQPTETVFITEDDMDLGALSELEEAVSDDESSTYSFIPGDHSQGPEVPEDDVESVNISPIIGSSNARHGDNDESAICKGEMQERSSEEDWTTPLKPLVADYSSPGMEGMVEMYMSGIEPILTG-----------------------------------------------------------EEIPPKQNEQEADNLSREVEMQTSQPIDSGTTQIQTVSPLVEQTKEQEYHQQVMIRKTPSGNILRSSRCSTTSDDGSEKAAGKGGTPSGGKDENLERETERDAVSSTKDQGVLSADPGHAEAATQPHSYSSPQQLLLSKKRQAKEEAIQKLCPSAVEVGENNDHSYKTFKQKKGLPAQNLLMSKIQQLSGSRLVLPASSASSHVLVAMEEKHNALPSGVNMNSSKDRIIENENADAAHTRNVQRGKQKTKTNARMTLTPFSRKTSEAKLIEETETLSETQDTMDNVIPPSQTDDTMKKKYVVSPRGAQGKEKTATSMSGTRHVETQSRDKQITQNDDTIQGQTEEAKEVQKQGIASDEHHKRAAGKRPTQEILERDETGNKKKARTSTPEKNKLEPFHMEHTRGMFGWEDSLRQRLSPVKDDKGKKRKKEEQGSAVKGNLAVPKDAPAASAAASTSSAPVTAPLTPGATSTSSIPENCTPTDQLPQEYKEGTYGMGELVF--GKLKGYTWWPGRVVSC--------------VETGRDPAPQGSCWVRWFGD--GKFSIVQVEDKLEPFGKFSQ--LFHERTYNRLQTY-----KRAILESLQVAADRAKKKFLSSPEANKNQD--------------------------RDRAMVQWALQDFSPAGPD--------------------------------------------------------SLKPSAEEARPPDHLVHQWQVAEGRSSGS-GSRRESVDSRLSTAKLFEEVRKRQLSVDQVCLACGSL--DVET-QHPLFRG-GLCEGCR---IEFLESSYQFDEDGYQSYCSLCAAGTEVLM-CGQDSC-CRSFCKDCLDLIVGPGTADQAVLEDPWLCYMCREEPRHGLLHRQQDWSARLQQFFAMDDVE--DFGQPKLYPLVPAQQRR---------------------PIRVLSLYDGIGTALLVLKELGVTVDVCYASEIDPDAIKVSQLRHAG-------------GIRHLGDIRSIRNTDIPKLPFDLVLGGSPCNDLSIANPNRAGLY---GTGVMFFNFYRLLREAAPAPGDDRPFFWLFENVVAMKHQYKQDISLFLG---------CNPVVVDAKDVS-PAHRARYF-WGNLPGMNRPLVPQASD--KLALE----------------------------------------------------------------------QCLE--------------KGCGRVAQVKKVRTV-------TTTLHSV---------QGKQQAAPVLME--------------------------------------------------------------------GQPDTLWCTELERVFGFPSHYTDVCNMSRCSRQRLLGRAWSIPVIRHLLAPLKDYFQCQ

>Aipt_O88508

--------------------------------------------------------------------------------------------------------------------------------------------------------------------------------------------------------------------------------------------------------------------------------------------------MGCINSSAEDTLALPQKPQPEQVLEESLKNASP-------------------------KKPSKVKRKLNFCDGYVKR----------------------------KKKRIPQQKQEKSETSEDKIPSSTSKEPSDN---------------------------------------------------------------------------------------------------------------------------------------------------------------------------------------------LAVLEKGLKTPEKCGTDQKKEEEPLVT------------------------------------------------------------------------FTIGDLVW--SKLKGFDWWPGRVVSY--------------LETRLSPPNPGHHWIRWFGDDKFSQMLHMCVRPFHEFEVKFNPSKMRGVY------------KKAIYEALEMAAKR------------------------------------------SKRSFTRADIKPKEPKKKQ---------------------------------------------------------------------------SKKKSQKPKP---------------------------------------------SKPLPTEKSKDGPLS---------------------------------------------------------EEEFREAIMSWA---------------LDGFLPDGPAGFAPSEGCDD--------------------------------------DDDNGDNNDDNG------DNDGD-----NNDDNDDDDNDDDGDNNDD-------------NGDNDGDNDDDDNDDIDDYNDD----NNGDNDDNGDNNGDNNDD------NDDNDDHTAVYVLT--C-------WLVKG--QMRAEDKKTISRFFK---------TNP-VVDAKDVS-PAHRARYF-WGNLPGMNRPT--KPVAGDKLSLQ----------------------------------------------------------------------ECLE--------------PNCGRKARFTKVQTI-------TTNANSL--------RQTKNNLLPVSTDSEGV----------------------------------------------------------------EREDILWCTEMERLFGFPSHYTDVNNMGRQQRQRLLG-NWSIPVIRHLVSPLKDYFKCR

>Aque_XP_003386062.1

--------------------------------------------------------------------------------------------------------------------------------------------------------------------------------------------------------------------------------------------------------------------------------MAEGPANCSIPLYRKRKAPLASPYTAAKHLELSPSPAPDSTLSPNQPVENYVDPI--------------------------------------------------------------------EELKSLEEDMSNFRRFSGRSKKQTNFFCPIA--------------------------------------------------------------------------------------------------------------------------------------------------------------------------------------------------PASFHRRRSNSATNAAVRAL--KFPSSTSPVDSGGPAGI--------------AASEEKKESIDNGEGEDKVVAAMEIATGEEVQQTVPQEIKKLPIGSLIW--GKLPGYEWWPGCIISFDKKKLIDDDEDTEEDEEEKEEDGGESVWVKWYGDNQLSQISFKKIFPFPSWIFHSSQFQVFLDERKALNNLTQEEAIEATPEKIEQGEKSSFPNLQQEESSIFTQIESDQESDSSKEKGGGRGAKKTKTGQFMEKDLLQWALTGFLPTGPD----------------------------------------------GFRPRPEHLIDIKMFPFGESPAPGNQFIIRAVIDHDYDK-KPENKLVLLQQRNKAQFELIKLGQLAIENICIACGSVNISA---QHPLFYG-GLCKHCK---ETFMECAYMFDEDGSQMYCTICSGGSQVFM-CDTPNC-SKVYCNPCIEKLCGPEERMKVQDATEWQCYMCSGEM-NGLLRKREDWDKRLHDLFVTDIETE--FEPPKFFPPIPAEMRK---------------------PMRVLGLFDGIGTGLLVLKELGINVEVYIASEIDPDAIKVSRIKHPE--------------IIHVGAIEQVTEKEVRSWPFDLVFGGSPCNDLSIVNPARKGIY--DGTGKLFFEFFRILSYAKPQPQDERPFFWLYENVVSMRAPDKKIISRFLQ---------CNPVVVDARDIS-AAHRARFF-WGNLPGMNRPAVPLPGD--RLTLQ----------------------------------------------------------------------DCLE--------------PNCFRHAQFTKLRTI-------TTKMNSI--------KQTKRAIVRVFSGGMFDITGG----------------------------------------------------------EEEEGDVLWCTEMERLFGFPDHYTDVANLGRSGRQKLLGKAWSVPVIRHLLSPLKDYFVSI

>Drer_P_005167049.1

INETLQTNFTQVEQCRSGACFCQLILFPGTINLKKVKSQKRSDFMQNYGLLQAAFRDLEVTEPVPVNELLSGKFRPNFTYLKWFKKFFYANVVYNAFEARDGQEIVLGRAGEESDMEINGGRRWIRASDMGDNYAYCTTCDYNIILLAGFHDRTNLPGRKQIEESIS----------------------------------NDIVSACKLNPYCIYIYGQVPFFEEKQARYCIRFLDVFQPEDSASSVSGGLFSILKKFEIPASNMVAVYINDHELTSESVVSQIRELNPQDLGGLYSIPDTACSAGLQTHSVQVQELIANIYRHFSTSNDIDGLKVHSNPLSNSEEFCVLVKRIHEMWSDLVSYFSKQICSQDQALGPLRAFGQHLQKSSVRADEASGLLRSYASSFLRPQAVIRYLKEQDPAILFISAREEELADFLSTFYNECLAIYKTLTTSI-----AASLPLSDSVLRAISQLLSPAGRLKVTGKNIVDLAVRFGFCSKPEDSAKLNDEFLEYQLAEEENLSSTHSIERYWCTVLKTFPPTSVFKRLVLCLLVLPSPSLDATKIFAQAIENGDADQLDDSSSESDTDMTKELDSNDDNSLDNSELQISPIKNGIMKKSRRSTSETVQHSNAVKPCVEVNLKNDGAKNDGTLTSGWECSLRQKPQARTVFQAGAGTWSKPAIQDVDRKPEAVKNDLSNSDQAYN-DGKGFAVGELVW--GKVKDFSLWPGLVVPW-----------------KGRIVPVSMRRVEWFGDGMFSEIHTDGLLPF-----GAFSKNFCSKSYEGLPTY-----KNAIYQILELAAERSGKLFPPSEKKGE-----------------------------EVKAMMDWAFGGFQPMGADGFL------------------------------------------PSADSSASNKTESDSSVSDYQPPAKRKYVF-------------KNRPSTQECNRDQMVQEVTSKGRKIEDFCLSCGSSNTEI---FHPLFKG-SLCIKCK---ENFTETLYRYDDDGYQSYCTVCCAGLEVIL-CGNASC-CRCFCKDCLNVLVGPGTFDKLKEVDPWSCYVCLPSKCYGVLKLRTDWSVRVQEFFANNSAFE--FEPHRVYPSIPAHKRR---------------------PIRVLSLFDGIATGYLVLKDLGFKLERYIASEICEDSIAVGMVKHEG-------------KIEYVKDVRTITRKHLAEWPFDLLIGGSPCNDLSMVNPARKGLF--EGTGRLFFEYYRMLTMMRPKEDDDRPFFWLFENVVAMSAHDKADICRFLE---------CNPVMIDAVKVS-PAHRARYF-WGNLPGMNRPV--ATSLTDNVDLQ----------------------------------------------------------------------DCLE----------------SGRTAMFSKVRTI-------TTKSNSI----------KQGKTLPVTMN--------------------------------------------------------------------GKEDYLWCTEMEKIFGFPKHYTDVNNMGRGQRQKVLGRSWSVPVIRHLFAPLKDYFACE

>Spur_P_787412.3

------------MRLPSKQVLLRMYWDISKQKRMSARLKHGQAFLRLLRSGSSKKRTSLQQRSPSAILKGKHKLSPGGDDRSKKRFKVDTDQEVSSNIPVKDGEKDAGRVSSQSALSLQSGTKHVHPEGPVDDKGTLEDDSTVSSSFGPQNEGSESTATNDRESEVS----------------------------------LPWRHKRKSLCPRKILRKMEQTQAEDSASSTPTDERNVPESSQEQVRFSDPDKEILSECIETPPARLESPSVMESEEQSGQLNLLLDQDPAEGNFSCSSDQTMVPSSSQGAIEEAVSFPGLRFQSIQFKKGNSASLLGEGFADVVNSSLIEETVQGDSLDNLLPGLVEGNPLTPKVKKHKHKSPHKNTSSARRLDKLHKKRKVKKNKHKSHEKCTTKSTSPGKFPRILPVSNWASLEDPVTSPCRVQLVDYLTSSQEKLPPKEGDEGNTRVQEVSSEAPQEVHQADQVATTLKEQVSTEDECPGVQLHEQDVHTEEVEKEPSESLSSPISSFKIREEPAEETSAGNRPCSSAVKGQRSPSHQGNDQSRAVRECGTTEGSSPHFPTGQEDSGQVCGSLDADGKIDTPSVGNDGNKRTSDKTKKAVRSRNPRGAGGWEQSLRQGRDPNTPKKGAKKVSPKMLPLPREKSNDVSSISNRPSTPWRKKEPRAVATPPPLDVKGTEGEIKEEAEKYGCIFRGQLVF--GKMKGFCWWPGRIVHHY-------------DRSIREPPPPLTRWVQWFGDDKYSLLSLGQIAGL-----EDFPDYFSVSAFQKWGLY-----QKACYEALNVAAKRVSVAMPTPPA------------------YPGASIKERQEYKARCREMQDWANDKFLPQGRE---------------------------------------------------------------------------SIQPSEEDKQ-TPPIPE-----------SPPPSPVQDRKRSPIKHSSRNSPEKEPFEDKREERMKDVRGGKWTIEYLEVAYLYDCEGYQAHCCICAEGNQITL-CDNQGC-YHSYCTVCMDNLVGPQESKRVSEQEPWSCYLCSADCSHGYLTRRDDWQEQLVDFFNRDQVAD--FEPLRMYKPLLLSNRR---------------------PIRVLSLFDGLGTGMLVLRELGFDVECYYASEVSEEAITVAAVRLKG-------------QIQQIGDVQKITPKELKSWPFDILIGGSPCNDLSIVNPARKGLA--GGTGLLFFEFYRILRDLQPLPDDPRPFFWLFENVVFMGRKDKLNICRFLQ---------CNPVMIDSRHMS-PTHRARYY-WGNLPGMHRPY--VAGADNPLCLQ----------------------------------------------------------------------ECLE--------------PHCDRQAQFSKVGTI-------TTTSHSI--------RQTKDAILPVIMN--------------------------------------------------------------------GREDGLWSTELERLFGFPDHYTDVGNLSRTARQKLLGKAWSVPVIRHLMAPLKDYFACS

>Nvec_127267

---------------------------------------------------------------------------------------------------------------------------------------------------------------------------------------------------------------------------------------------------------------------------------------------------------------------------------------------------------------------------------------------------------------------------------------------------------------------------------------------------------------------------------------------------------------------------------------------------------------------------------------------------------------------------------------------------------------------------------------------GVLVW--SKLKGYDWWPGRVVTY--------------MEAGRPPPGPGNHWVKWFGDNKFSQVTYDTVLPFAEFKSNFLVTKMKGLY------------KKAVLDALEVLKKKKDTETPAEPDEPLSEEE-------------------------KCDAMVNWALTGFQPQGAS--------------------------------------------------------GFAPTLEETIFLPPVSSDSGSESTKGRFG-KPVKEPNLVQDIKGTCMVFKLIMYVFMLDICLACGDC--KVYA-QHPLFEG-GLCKECK---QSFLECTYLFDEDGGGMYCTICGDGQEVFM-CDNEGC-FRSYCGPCLEMLAGRGTVREIASREKWICYMCSGKG-DRLIHRRKDWQSKLHELFLSD--REKEYDTPIVYPVVAPEDRK---------------------PIRVLALFDGIATGLQALNELGIVSDKYYSAEIDEQAIQVTKVNHGD-------------RITHLGDIKDLTESQIRELPFDLVIGGSPCQDLSIANPARRGIF--EGSGRLFFEFFRLLMHAKPSTCPSRPFFWLFENVVGMRAEDKKTISRFLQ---------SNPVVVDAKEVS-PAHRPRYF-WGNLPGMNRPAIPLPGD--RLTLQ----------------------------------------------------------------------ECLE--------------PNCGRKARFTKVQTI-------TTNANSL---------QTKKNILPVAV---------------------------------------------------------------NDDGGQEREDILWCTEMERLFGFPSHYTDVNNMGRTQRQRLLGNAWSVPVVRHLLSPLKDYFKCT

>Locu_P_015220711.1

LTSDAGDKYDRVELLAWLNDSLQTKKVEQICSGAAYCMDWLFPGSMDLNKIKFQAQEELEFIHNYNLLQKSFRKTGVTKVIPVDELVKGAFQFLKWFKKFFDANFYGTEKPVSPPNTKSPNSFDEELHDPKTKRITFLTHWQETFSWVRQSKIHHRGQVDLKRHQNSCLTNKTKDNEDVCGGDSTTCGVATLKFIKKYCTSLKHPKDIASICSQTPYCIYLHFFDEKAGQNRIRLLDVVRPKTETVASVSSCLVETLQKSGLPVLNLSGFHTDCPPEEGAAVLSRLKEMNPICVGSLSHLASLACRAGVAASSKDVSQLISNIHSHYSCANDNLKELFANIEPYQADLSCDHKSEKVKLICGQLGDHQLRATFSHALEPLCAFQSKLEQNGDLAKEASGLLRSYAASLLLPEAVVKLLKGHDSKVLAEVEEYLTKNQQGLGTVWGDFFKDVSLFYASVTAQIMQNLPLNDTLLRNISILLNPSCKLKITGKVVADLATHLGLCKRPEETTQLIDEFLEYQLLENDEAQVLPPSPLQHWSAVLKTALSGTNEQMSIFRKLILTLLSLPYPSMEATKAFSQAFENGDVSLLDETVTENELDDTRDSDLTAGNEDVILVEDGHLSPETTKHPKHCNSSADVIDLTQMKPCAVLLKKINCTSVKPPVRKIFQVGANSLENVKKEDNSGSTQTNSGAPGVKRGNHTSTPKSYPYQDGKGFSVGELVW--GKVKGFSWWPGLVVGW-----------------KSKQIPTAMRKVEWFGD--GMFSEIYTERLLPFSAF---SKCFCNSSFATLPAY-----KDAIFQSLEMVSERSKKTFASCKTESKDE---------------------------KLKPMLEWAFGGFKPKGPD-------------------------------------------GFKPPVTDNLAAKAALLDTPVPEYQPPTK-------KQKYVY-KNKGGNEQEYSRVQMVREVLQKGK-NIEDFCLSCGTP--QTEI-FHPLFEG-SLCLKCK---DNFTETLYRYDEDGYQSYCTVCCAGLEVIL-CGNASC-CRCFCLDCLDILVGPGTFDKLKEVDPWSCYMCLPSQRYGVLKRREDWSIRVQEFFVNN--SAMEFEPHRVYPSIPANQRR---------------------PIRVLSLFDGIATGYLVLKDLGFKVERYIASEICEDSIAVGMIKHEG-------------RIEHVHDVRTITKKHIAEWPFDLLIGGSPCNDLSIVNPARKGLF--EGTGRLFFEYYRLLNILKPKEGDNRPFFWLFENVVAMGVRDKTDICRFLE---------CNPVLIDAVKVS-PAHRARYF-WGNLPGMNRPLASSQSD--KLDLQ----------------------------------------------------------------------DCLE----------------HGRQAKFSKVRTI-------TTRPNSI---------QGKSEILPVVMN--------------------------------------------------------------------GKEDNLWCTELERIFGFPRHYTDVNNMGRGSRQKVLGRSWSVPVIRHLFAPLKDYFACE

>Lcha_P_014350916.1

-----------------------------------------------------------------------------------------------------------------------------------------------------------------------------------------------------------------------------------------------------------------------MPSSSTSTKIVANGTMTTQGLLNGSLHPELTENGKDFSVSVLNGDADVMPALEKKKVNGEATVQDNAS-----------------PIREGVPWAFSFDEGR-------------------------------QRGGVAGWETSLRQRPPPRTIFQAGLTFQTR----------------------------------------------------------------------------------------------------------------------------------------KRKEDKKELGGKRAFRQKSSVIVIQDRVNPLKPDEAENAFIDLTEEDSKDSAQSNTTGASGTQEAQNGTVDFVHESQLEN--------------------------------------------------------------ETLEYQDSKGFGLGELVW--GKIKGFNWWPAIVVTW--------------RAPAKRRATSGMRWLRWFGDGKYSEVSVDKLGPL-----MSFSQYFNSSALKWNSY------KKAVYEALEVASKRAGKEFPEENCESLEQ---------------------------RLKSMMEWALGGFEPMGLEGLK----------------------------------------PPENPENGFLKHPFLQEMVPEYNPAF------KRQKNTSYKP-TKEDSEENPRPSKERLIHEVLENNKCLEDFCMSCGSQNQTT---FHPLFEG-GLCQSCK---DTFLETSYMYDDDGYQSFCTICCGDGELLL-CGNMNC-CRCFCVYCLETLVGPGASEQAKKLDPWSCYMCLPRKRYGILQRRQDWNLKLQEFFASDKGQE--FEAPKIYPAVPAEQKR---------------------PIRVLSLFDGIATGYLVLKDLGFNIDLYIASEICEDSIAVGTVRHEG-------------RIKYVHDIKNITRENVREK-------APPPKKKTPPQPLMGGLY--KGTGRLFFEFYRLLSFAKPEKGENRPFFWLFENVVAMGVSDKRDISRFLE---------CNPVMIDAIEVS-AAHRARYF-WGNLPGMNRPL--TASQTDKLELQ----------------------------------------------------------------------DCLE----------------HSRIAKLGKIRTI-------TTRSNSL--------KQGKDQLLPVLMN--------------------------------------------------------------------GKEDILWCTEMERIFGFPVHYTDVSNMGRGARQKLLGRSWSVPVIRHLFAPLKDYFACE

>Drer_AAI24099.1

-----------------------------------------------------------------------------------------------------------------------------------------------------------------------------------------------------------------------------------------------------------------------------MRKEEIKKSSEIVMPSNKYPSADKMTATAAMNRDTSVGDGLSENDSGLEMTSENSPL----------------------TPAEPPSPFCPKQNGGAASPADESVTEEDSAWDSSNSEE--------KAEVGSGCETGLRQRPRPRTIFQAGLTAHSK-------------------------------------------------------------------------------------------------------------------------------------------------------PRSRERGHSKEDHSDLVASVPEGPALELM---------EQDSKDSAQSSTTSTSTTETASQPEYK--------------------------------------------------------------------DNKGFGIGELVW--GKIKGFSWWPGMVVTW--------------RATGRRQASHGMRWLQWFGDGKFSEVSADKLDSI-----TAFPKFFNQSSYTKLASY-----RRAIFQALEVASLRAEKTFPPSEADSLEE---------------------------QVKPMLDWAHGGFLPKGQEGLK------------------------------------------------------PKENAEYCVFPLASESSTPSAKRARLPL-NKAKPGIEEVYSREQMVNEVLKNHRSIEEFCLSCGKTRVAT---FHPLFEG-GLCLTCK---DAYLENSYMYDDDGYQSYCTVCCGGREMLL-CGNANC-CRCICVDCLDILVGAGAANSARNLDPWRCYMCQPLQQYGVLKKRHDWSLKLQEFFVNDSGQE--FESPKIYPAVPAEQRR---------------------PIRVLSLFDGIATGYLVLRDLGFKVDLYIASEVCEDSISVGAVRHEG-------------KIQYVHDVRNITRKNIAEWPFDMVIGGSPCNDLSIVNPARKGLY--EGTGRLFFEFYRLLSEAKPKEGEDRPFFWMFENVVAMSVNDKRDISRFLE---------CNPVMIDAIEVS-AAHRARYF-WGNLPGMKRPL--CASGMDKLELQ----------------------------------------------------------------------DCLE----------------HGRVAKFGKVRTI-------TTRSNSI--------KQGKDQHFPVMMN--------------------------------------------------------------------GKEDILWCTELERIFGFPVHYTDVSNMGRGARQKLLGRSWSVPVIRHLFAPLKDYFACE

>Locu_P_015220486.1

--------------------------------------------------------------------------------------------------------------------------------------------------------------------------------------------------------------------------------------------------------------MKQAREKILGPEMPSNKYSSTIMASDKMTATAAVNSDMDGLSEKDSGLELTNGHLGSSPLTASEPPSPLSPKQNGSPEINMDKPSSCKKRL---RKNSELEDESAWDSSYSEE----------------------------KSRGVAGWETSLRQRPPPRTIFQAGLSPHGK----------------------------------------------------------------------------------------------------------------------------------------------PRSRDSSLKPKQDESSSLNQNGGSPSGLQAHLPEAPSLELMEQDSKDSAQSSSTSTSQETASSSTESGCQPEYK--------------------------------------------------------------------DNKGFGIGELVW--GKIKGFSWWPGIVVTW--------------RATGKRQASPGMRWLQWFGDGKFSEVSADKLDSF-----MAFPKYFNQASYNKLASY-----RRAIFQALEMASTRSGKTFPLCENDSPED---------------------------QVKPMLDWANGGFQPKGLEGLK----------------------------------------PSENTVTGSLNHHVLEVSLPEYFPP-----------AKKQKP-CKPKLGSEEDYSREQMVHEVLKNNRSIEEFCLSCGKTRAAT---FHPLFEG-GLCQTCK---DVYLEISYMYDDDGYQSYCTVCCGGREVLL-CGNANC-CRCFCVDCLDILVGAGAAESAKVLDPWRCYMCQPPQQYGVLKRRGDWSVKLQEFFVNDNGQE--FESPRTYPAVPAEQRR---------------------PIRVLSLFDGIATGYLVLKDLGFKVDQYIASEVCEDSISVGVVRHEG-------------KIQYVHDVRNITRKNIEEWPFDLVIGGSPCNDLSIVNPARKGLY--EGTGRLFFEFYRLLSFAKPKEGEDRPFFWMFENVVAMGVNDKRDISRFLE---------CNPVMIDAIEVS-AAHRARYF-WGNLPGMNRPL--CASGMDKLELQ----------------------------------------------------------------------DCLE----------------HGRLAKFGKVRTI-------TTRSNSI--------KQGKDQHFPVIMN--------------------------------------------------------------------GKEDILWCTELERIFGFPVHYTDVSNMGRVARQKLLGRSWSVPVIRHLFAPLKDYFACE

>Pmar_ENSPMAT00000008525

----------------------------------------------------------------------------------------------------------------------------------------------------------------------------------------------------------------------------------------------------------------------------------------------------------------------------------------------------------------------------------------------------------------------------------------------------------------------------------------------------------------------------------------------------------------------------------------------------------------------------------------------------------------------------------------------------------------------------------------RGFAVGELVW--GKIRGFSWWPGVLVSW--------------EDTGQCCAAEDTRWVCWFGDCKFSLVAVDKLMPF-----SHFKENFNQQTYAKKLVY-----RKAIYQALQVASQRVNKVFPQPEESDTEPGLSLEE---------------------RISPMMHWAMSGFPPKGAKGLV-----------------------------------PLPEQEISSTFFDKYYDRILWGSKSYLAFSPQRSWSRRRSTSSLRRR-RVETWVVVDECWREQIIQEVQEDIRTIEGCSLECFEICVHA---QHPLFEG-GICQRCQ---ITFMECAYQYDDDGYQSYCSICCGGREVLM-CGNINCFCRCFCVECVDLLVGSGTAKTAIEEEPWRCFMCRPSLACGLLNPRPDWPARLQHLFSNTHSQE--FEPPKLYPPITPDHRR---------------------PIKVLSLFDGIATGLLVLKELGIRVERYVASEVCQDSINVGTVRHAG-------------DITYIGDIRNLTYSNLQKWPFDLVIGGSPCSDLSIVNPARKGLY--EGTGRLFFEFYRLMNEAKPREGEERIFFWLFENVAAMGNNDKRDISRFLE---------CNPVMIDAKDIS-AAHRARYF-WGNLPGMNRPL--AVTAVDKLELQ----------------------------------------------------------------------DCLE----------------HGRTARFSKIRTI-------TTRSNSI--------KQGKDQHFPVMMN--------------------------------------------------------------------GREDILWSTEMER----------------------------------------------

>Cmil_P_007883119.1

----------------------------------------------------------------------------------------------------------------------------------------------------------------------------------------------------------------------------------------------------------MKTNECSSGAIGNISKVKPLLNGESDQWEQATPPTPQSPNTSESESPADSDMEVMLNDNEEIRNGPAGRLKSKLRCQASA-----------------------------------------------------------------KRRGVSGWESSLRQRPTPRIIFQAGISFHHK----------------------------------------------------------------------------------------------------------------------------------------------------------KKKEDNNMKPKKELSRMPREVPAKSLVEGNSIIDLTMEPLESNTKTLNKTEELVCENKVVKG--------------------------------------------------------------VLLQYQDGKGFGLGELVW--GKIKGFSWWPAIVVSW--------------RVPGKRLAISGMRWLQWFGDGKFSEVSTDKLVPL-----TAFSQYFHSSAYNKLVSY-----KRAVYQSLEIASTRSEMTFPSNERSTLED---------------------------SIKPMLDWAFGGFQPKGYEGLK----------------------------------------PKEKAENDAVSEKLTELYVPDFYP--------PSKKQKTSIY-KSKEGTEDEQRGREKILNEVMSKNKSIEEFCLACGSTSVST---FHALFEG-GLCAVCK---DIYLETSYMYDDDGYQSYCTVCCGGREVLL-CGNANC-CRCFCVDCIDILVGPGSSEEAKVQDPWRCYMCLPHESYGVLRRRSDWTLKLQQFFASDNGQE--YDPPKIYPAVPAEQRR---------------------PIKVLSLFDGIATGFLVLRDLGFKVERYIASEICEDSIAVGTVRHEG-------------KITYVHDVRNISRKNIQDWPFDMVIGGSPCNDLSIVNPARKGLY--EGTGRLFFEFYRLLHETRPKEWEDRPFFWLFENVVAMGVNDKRDISRFLE---------CNPVMIDAIDVS-AAHRARYF-WGNLPGMNRPL--VASSADKLELQ----------------------------------------------------------------------DCLE----------------HGRIAKFSKVRTI-------TTRSNSI--------KQGKDQHFPVIMN--------------------------------------------------------------------GKEDILWCTELERIFGFPVHYTDVSNMGRGARQKLLGRSWSVPVIRHLFAPLKDYFACD

>Acar_NP_001280056.1

------------------------------------------------------------------------------------------------------------------------------------------------------------------------------------------------------------------------------DSEETVVLVNGNCGDPSEDKANAALDVKGKRKKPDSSDAEQLSSKTEEKTDEIPGSPKKWQEVNMKRKKLSDTDSEDEVILINISDDESAEEKANANLDVKGKRKK--------------------PDADDAGQLSPTREEGQEI----------------------------RTRGNAGWENCLRQKPPPRIVFQAGPASQDK----------------------------------------------------------------------------------------------------------------------------------------------------------------KSKDDVESVESFKERHKIRYSRRISRNGPINFSEDELSESSRVGSNGIPNGNHDPGLLRGCKEYQ-----------------------------------------------------------DGKPFQMGELVW--GKIRGFAWWPAIVVTW--------------KSTSNRPAAPGMRWVKWFGDGMFSEVSADKLVGL-----VNFRDHFNSSTLQKMASY-----RQAILHVLEVASSRCGKTFPSNAGDSIEE---------------------------KLKPMVEWALNGFKPTGGKGLK---------------------------------------PPKGEEDVDQEDKSSVEEVVVVLEPSPP-----PKRVKTAVCN-GNGKEQSEENQSRERMLSEVRNNKKSLEDSCLCCGKSNPAT---FHPLFEG-GLCTTCQ---DRFLEYFYMYDEDGYQTYCSICCEGTELLL-CDNASC-FRCFCVDCLNTLVGRGAADAAREEEPWSCYMCQPQKQHGLLQRRHDWNTRLKEFFTNERGQE--YKAPKIYPAIPAAQRR---------------------PIRVLSLFDGIATGYLVLRDLGIKVEKYVASEICEESIAVGTVRHEG-------------NITYVHDVRNITKRNIDEWPFDLVIGGSPCNDLSIVNPARKGLY--EGTGRLFFEFYHLLNYSRPKPEEERPFFWLFENVVAMRVNDRRDISRFLE---------CNPVMIDAIKVS-AAHRARYF-WGNLPGMNRPL--VASKTDKLELQ----------------------------------------------------------------------DCLE----------------YSRTAKLKKVQTI-------TTKSNSI--------RQGKAQMLPVRMN--------------------------------------------------------------------GKDDDLWCTELERIFGFPLHYTDVSNMGRGARQKLLGRSWSVPVIRHLFAPLKDYFACD

>Mmus_NP_001258674.1

---------------------------------------------------------------------------------------------------------------------------------------------------------------------------------------------------------------------------------------------------------------------MKGDSRHLNEEEGASGYEECIIVNGNFSDQDTKDAPSPPVLEAICTEPVCTPETRGRRSSSRLSKR--------------------------------------------------------------------EVSSLLNYTQDMTGDGDRDDEVDDGNGSDIL---------------------------------------------------------------------------------------------------------------------------------------MPKLTRETKDTRTRSESPAVRTRHSNGTSSLERQRASPRITRGRQGRHHVQEYPVEFPATRSRRRRASSSASTPWSSPASV-----------------EVTPKSVSTPSVDLSQDGDQEGMDTTQVDAESRDGDSTEYQ----------DDKEFGIGDLVW--GKIKGFSWWPAMVVSW--------------KATSKRQAMPGMRWVQWFGDGKFSEISADKLVAL-----GLFSQHFNLATFNKLVSY-----RKAMYHTLEKARVRAGKTFSSSPGESLED---------------------------QLKPMLEWAHGGFKPTGIEGLK-----------------------------------------PNKKQPENKSRRRTTNDSAASESPPPK--------RLKTNS-YGGKDRGEDEESRERMASEVTNNKGNLEDRCLSCGKKNPVS---FHPLFEG-GLCQSCR---DRFLELFYMYDEDGYQSYCTVCCEGRELLL-CSNTSC-CRCFCVECLEVLVGAGTAEDAKLQEPWSCYMCLPQRCHGVLRRRKDWNMRLQDFFTTDPDLEE-FEPPKLYPAIPAAKRR---------------------PIRVLSLFDGIATGYLVLKELGIKVEKYIASEVCAESIAVGTVKHEG-------------QIKYVNDVRKITKKNIEEWPFDLVIGGSPCNDLSNVNPARKGLY--EGTGRLFFEFYHLLNYTRPKEGDNRPFFWMFENVVAMKVNDKKDISRFLA---------CNPVMIDAIKVS-AAHRARYF-WGNLPGMNRPV--MASKNDKLELQ----------------------------------------------------------------------DCLE----------------FSRTAKLKKVQTI-------TTKSNSI--------RQGKNQLFPVVMN--------------------------------------------------------------------GKDDVLWCTELERIFGFPAHYTDVSNMGRGARQKLLGRSWSVPVIRHLFAPLKDYFACE

>Btau_NP_861529.2

---------------------------------------------------------------------------------------------------------------------------------------------------------------------------------------------------------------------------------------------------------------------------------------------------------MKGVDSLINEDKHANRREDSVITDGAVIAQ---------------------CCDSKQSPSPRILQSIS------------------------------TLEIIGARGVRGRRSSSRLSKREVSSLLSYT----------------------------------------------------------------------------------QDLTGDGDGEGEDGDGSDTPVMPKLFR-----------------------------------ETRTRSESPAVRTRNNSSTSTRERHRPSLRSTQGRQARNHVDESPVAFSTTRSLRRRTGSSAGTPWPSPASPTIDLTDEDV----VPQS------SSTPYARLGQDSQQESMESSQLDADGRDADSTEYQ----------DGKEFGIGDLVSCGGKIKGFSWWPAMVVSW--------------KATSKRQAMSGMRWVQWFGDGKFSEIPADKLVAL-----GLFSQHFNLATFNKLVSY-----RKAMYHALEKARIRAGKMFPSSPGDSLED---------------------------QLKPMLEWAHGGFKPTGVEGLK---------------------------------------PNNKQPENKTRRRTADDSATSDYCPPP--------KRLKTNCY-NNGKDRGEEDQSREQMASDVASNKGNLEDSCLSCGRKNPVS---FHPLFEG-GLCQTCR---DRFLELFYMYDDDGYQSYCTVCCEGRELLL-CSNTSC-CRCFCVECLEVLVGAGTAAEAKLQEPWSCYMCLPQRCHGILRRRKDWSVRLQAFFTSDPGLE--YEAPKLYPAIPANRRR---------------------PIRVLSLFDGIATGYLVLKELGIKVEKYVASEVCEESIAVGTVKHEG-------------NIKYVNDVRNITKKNIEEWPFDLVIGGSPCNDLSNVNPARKGLY--EGTGRLFFEFYHLLNYTRPKEGEDRPFFWMFENVVAMKVGDKRDISRFLE---------CNPVMIDAIKVS-AAHRARYF-WGNLPGMNRPV--IASKNDKLELQ----------------------------------------------------------------------DCLE----------------FNRTAKLKKVQTI-------TTKSNSI--------RQGKNQLFPVVMN--------------------------------------------------------------------GKEDVLWCTELERIFGFPVHYTDVSNMGRVARQKLLGRSWSVPVIRHLFAPLKDYFACE

>Hsap_NP_787044.1

---------------------------------------------------------------------------------------------------------------------------------------------------------------------------------------------------------------------------------------------------------------------------------------------------------MKGDTRHLNGEEDAGGREDSILVNG--------------------------ACSDQSSDSPP------------------------------------ILEAIRTPEIRGRRSSSRLSKREVSSLLSYT------------------------------------------------------------------------------------QDLTGDGDGEDGDGSDTPVMPKLFR-----------------------------------ETRTRSESPAVRTRNNNSVSSRERHRPSPRSTRGRQGRNHVDESPVEFPATRSLRRRATASAGTPWPSPPSSTIDLTDDTEDTHGTPQS------SSTPYARLAQDSQQGGMESPQVEADSGDGDSSEYQ----------DGKEFGIGDLVW--GKIKGFSWWPAMVVSW--------------KATSKRQAMSGMRWVQWFGDGKFSEVSADKLVAL-----GLFSQHFNLATFNKLVSY-----RKAMYHALEKARVRAGKTFPSSPGDSLED---------------------------QLKPMLEWAHGGFKPTGIEGLK---------------------------------------PNNTQPENKTRRRTADDSATSDYCPAP--------KRLKTNCY-NNGKDRGDEDQSREQMASDVANNKSSLEDGCLSCGRKNPVS---FHPLFEG-GLCQTCR---DRFLELFYMYDDDGYQSYCTVCCEGRELLL-CSNTSC-CRCFCVECLEVLVGTGTAAEAKLQEPWSCYMCLPQRCHGVLRRRKDWNVRLQAFFTSDTGLE--YEAPKLYPAIPAARRR---------------------PIRVLSLFDGIATGYLVLKELGIKVGKYVASEVCEESIAVGTVKHEG-------------NIKYVNDVRNITKKNIEEWPFDLVIGGSPCNDLSNVNPARKGLY--EGTGRLFFEFYHLLNYSRPKEGDDRPFFWMFENVVAMKVGDKRDISRFLE---------CNPVMIDAIKVS-AAHRARYF-WGNLPGMNRPV--IASKNDKLELQ----------------------------------------------------------------------DCLE----------------YNRIAKLKKVQTI-------TTKSNSI--------KQGKNQLFPVVMN--------------------------------------------------------------------GKEDVLWCTELERIFGFPVHYTDVSNMGRGARQKLLGRSWSVPVIRHLFAPLKDYFACE

>Tgut_P_002196308.1

------------------------------------------------------------------------------------------------------------------------------------------------------------------------------------------------------------------------------------------------------------------------------------MKKDKSHGQDGADCRLILINGDNAPSVVLEAKGKRSTPDSRRLGLKKEKNSN--------------------GDLSKEDLAWPLDLPVQRQV---------------------------QLWGSAGWESSLRQKPTLCLMFQAGRTRQEM---------------------------------------------------------------------------------------------------------------------------------------RIKEAARSVESLRDLPTPVRSSRRRSAVPTPVTIDLTE-------------------EDSQDSSRSSSTLSGSSSQEGQNGSADLGAEEAESRDMGMA-----------------------------------------------LEYQDGKEFGIGELVW--GKIKGFSWWPAIVVSH--------------RATAKRQAVSGMRWVQWFGDGKFSEVSADKLVGL-----MAFRQHFNSSTFNKLVSY-----RRAIYHALEVARSRSGKTFTTGPRESLEE---------------------------QLKPMIDWAITGFKPLGLKGLQ----------------------------------------PPKGSENGVLRNGTEEVVSLEQCPP-------TKRLKTYPYN-SSKEQRVEEDQTREQMVSEVTSNSGNLEESCLSCGRRNPAT---FHPLFKG-GLCQTCR---DRFLEYFYMYDEDGYQSYCTVCCAGKELLL-CSNASC-CRCFCVECLDVLVGRGTSTRVKEQEPWNCYMCQPQQNHGVLQRRQDWNTRLQDFFTSDKGQE--YAAPKIYPTVPPAKRR---------------------PIRVLSLFDGVTTGYTVLKDLGIQVEKYIASEICENPIAMGKVRPEG-------------NITYVHDVRNITKRNIEEWPFDLVIGGSPCDDVSLVNPTRKALFLAEGTGRLFFEFYHLLNYARPKAGEERPFFWMFENVVAMRINDKRDISRFLE---------CNPVMINAIKIS-AAHRARYF-WGNLPGMDR------------------------------------------------------------------------------------------------------------------------------------------------------------------------------------------------------------------------------------------IFGFPLPYTDVSNISRGTRQKLLGGSWSVPVIRHLFSPLKDYFACE

>Cpic_P_005304927.1

---------------------------------------------------------------------------------------------------------------------------------------------------------------------------------------------------------------------------------------------------------------------------------------------------------MSVQVAMIDLTEEDSKDSSQSSST---------------------------------------------------------------------------------------------------------------------------------------------------------------------------------------------------------------------------------------------------------------------------------------------------------TSASSTQEDQNGSPELVAGEQESR---VAGEVVEYQ--------------------------------------------------------DGKDFGIGELVW--GKIKGFSWWPAIIVSY--------------RVTSKRQAISGMRWVQWFGDGKFSEVSADKLVGL-----MAFRQHFNHSTFNKLVSY-----RRAVHHALEVARSRAGKTFPGTPGESLEE---------------------------QLKPMIDWALSGFKPMGCKGLK----------------------------------------PPKTPENGVALLKNGIEEVCAPEHCPP------AKRLKTNFC-NNSKERVEEDQSREQMVSEVTNNNRSLEDSCLSCGRKNPAT---FHPLFEG-GLCQTCR---DRFLELFYMYDEDGYQSYCTVCCEGKELLL-CSNASC-CRCFCVECLEVLVGQGTSAKAKEQEPWSCYMCQPQKCYGVLQRRPDWNVRLQDFFTSDKGQE--YDAPKIYPAVPATERR---------------------PIRVLSLFDGIATGYLVLKDLGIKVEKYIASEICEDSIAVGTVRHEG-------------NITYVHDVRNITKRNIEEWPFDLVIGGSPCNDLSIVNPARKGLY--EGTGRLFFEFYHLLNYARPKAGEERPFFWMFENVVAMRVNDKRDISRFLE---------CNPVMIDAIKVS-AAHRARYF-WGNLPGMNRPL--VASRTDRVELQ----------------------------------------------------------------------DCLE----------------YSRIAKLRKVQTI-------TTKSNSL----------RQGKSLPVLMN--------------------------------------------------------------------GKEDNLWCTELERIFGFPLHYTDVSNMGRGARQKLLGRSWSVPVIRHLFAPLKDYFACE

>Ggal_P_015151885.1

---------------------------------------------------------------------------------------------------------------------------------------------------------------------------------------------------------------------------------------------------------MSMVTSLERLEQRLAVMVAAQETSQAWSSLGEEGPAAMKKEKGRDEADCRAELILFDGDCTDPTKDTAALLLEASGKPG--------------------APDAGSLGLAPLKNKRVSKDLSK--EELSWPLTLMEPQEV-------RPRGSAGWESSLRQKPPVRLIFQAGQTHHEM----------------------------------------------------------------------------------------------------------------------------------------QIKESSSVEALRELPAPLRSSRRRTAVPVLTTIDLTEEDSRD-------SSQSSSTLSGSSSQEGQNGSTELVAEEPESR--------------------------------------------------------------IALEYQDGKEFGIGELVW--GKIKGFSWWPAIVVSY--------------RATSKRQAVSGMRWVQWFGDGKFSEVSADKLVGL-----MAFRQHFNTATFNKLVSY-----RRAIYHALEVARSRSGKTFTSAPGESLEE---------------------------QLKPMIDWALTGFKPLGVKGLQ----------------------------------------PPKSSENGALRNGTEEVLCLEHCPP-------TKRLKSNPCN-SSKEQRMEEDQTREQMVSEVTNNSGSLEDSCLSCGRRNPAT---FHPLFEG-GLCHTCR---DRFLELFYMYDEDGYQSYCTVCCEGKELLL-CSNASC-CRCFCVECLEVLVGRGSSAKAKEQEPWNCYMCQPQRSYGVLQRRQDWSSRLQDFFTSDKGQE--YDAPKIYPAVPPAKRR---------------------PIRVLSLFDGIATGYLVLKDLGIQVEKYIASEICEDPLAVGTVRHEG-------------NITYVHDVRNITKRNIEEWPFDLVIGGSPCNDLSLASPPRKAPYRTEGTGRLFFEFYHLLNYARPKAGEERPFFWMFENVVAMRVNDKRDISRFLE---------CNPVMIDAIKIS-AAHRARYF-WGNLPGMNR------------------------------------------------------------------------------------------------------------------------------------------------------------------------------------------------------------------------------------------IFGFPLHCTDVSNISRGARQKLLGRSWSIPVIQHLFSPLKDYFACE

>Drer_P_005160915.1

------------------------------------------------------------------------------------------------------------------------------------------------------------------------------------------------------------------------------KQSPKQEAEQEVCEVSKEEKVTDTPTRRVGRPARKRKAPAVNSCEVVTKSSGCVKQSECNGEADSRVAKLQRNDSSPESSHQNGADTPPGEDSAPSTPRKKRGRRKEER-----------------DIEEDDTSSDTSRGESDGA----------------------------RHRGRQGWDISLRRRPVQRETFQAGDPYHIS-RREKEELLAHWKKEKEKKVRLVQMSDTMDH--------QSAVGSQEDEPAMILPTIPIGGSRDKAGKKFEKKVRLVQMSDTMDHQSAVGSQEDEPAMILPTIPIGGSRDKA-----------GKKFEKKVRLVQMSDTMDHQSAVGSQEDEPAMILPTIPQQQQKKVRLVLMSDTMDDQSAVGSQEDEPAMILPAIPQQQHTDPASPTVATTPEPPPVACGEKVLTGIAPIH-----------------------------------------------QEYQDGLGYGIGELVW--GKLRGFSWWPGRIVSC--------------WITNRGPAAEGTRWVMWFGDGKLSMMCVEKLLPL-----SAFSKTFHQPTYNKQPVY-----RKAIFQVLLTGGLRAGKPFPHCEYTNYTPEGKMEENET------------------QAQQMIKWASTGFLPSGPKGLE-------------------------------------PPPAERNPYTEVYPEMWEPEAASYTAPPP------AKKPRKNSVE-KTKIKEIIDEGTRERLMLEVKQKCRNIEDICISCGSLNVTL---EHPLFVG-AMCQSCK---NSFLECAYQYDEDGYQSFCTICCGGRQVLM-CGNNNC-CRCYCVECVDLLVGTGCAQAAISEDPWNCYMCCSRSVFGLLRRRDDWTSRLQLFFANNHDQD--FEPPALYSPVAPEKRQ---------------------PIRVLSLFDGIATGLLVLKDLGIQVDRYVASEVCEDSITVGMVRHFE-------------RITYVGDIRNITRKHIQEWPFDLVIGGSPCNDLSIVNPARKGLF--EGTGRLFFEFYRLLHEARPKEGDDRPFFWLFENVVAMGVSDKKDISRFLE---------CNPVMIDAKEVS-AAHRARYF-WGNLPGMNRPL--TAMVNDKLDLQ----------------------------------------------------------------------DCLE----------------HGRTAKFNKVRTI-------TTRSNSI--------KQGKDQHYPVYMN--------------------------------------------------------------------NKEDILWCTEMERVFGFPVHYTDVSNMSRLARQRLLGRSWSVPVIRHLFAPLKEYFAC-

>Skow_P_006824674.1

-----------------------------------------------------------------------------------------------------------------------------------------------------------------------------------------------------------------------------------------------------------------------------------------------------------------------------------------------------------------------------------------------------------------------------------------------------------------------------------------------------------------------------------------------------------------------------------------------------------------------------------------------------------------------------------------------------------------------------------------------------------------------------------------------------------------------------------------------------------------------------------------------------------------------------------------------------------------------------------------------------------------------------------------------------------------------------------------------------------------------------------------------------------------------------------------------------------------------------------------------------------------------------------------------------------------------------------------------------------------------------------------------CNPVLVDAKDVS-AAHRARYF-WGNLPGMNRPSLAESNN--SLNLQ----------------------------------------------------------------------SCLE--------------PHCNRQALFNKVRTV-------TSKANSI---------QCKEQILPVKMG--------------------------------------------------------------------TKSDALWCTEMERLFGFPEHYTDVGNMSRTARQKLLGQAWSVPVARHLLAPLKEYFACD

>Pmar_ENSPMAT00000007628

-------------------------------------------------------------------------------------------------------------------------------------------------------------------------------------------------------------------------------------------------------------------------------------------------------------------------------------------------------------------------------------------------------------------------------------------------------------------------------------------------------------------------------------------------------------------------------------------------------------------------------------------------------------------------------------------------------------------------------------------MEGPPLL----------------------------------------------------------------------------------------------KRKLVDSYVYVDRK----------------------------------------------------RPLLPKKAKPPP------------------------------------------------------------------------------------IKAKQIKLAGLRDQHIYEVRRKNRNIEDVCIACGSLDVIT---HHPLFEG-GMCQPCK---STFMECAFQYDDDGYQAYCSVCYGGGEVLM-CGNSNC-CRWGSVECVEMLVSVGAAKSAIAEEPWSCFMCRPKGAHGMLRRRDDWASKLQNLFTNAHSQE--YPIPKIYPPILTSQRK---------------------AIRVLSLFDGIATGLLVLKDLGIKLERYVASEICEDSIVVGTVRHEG-------------KITYVGDIRNLTRKHILEWPFDLVIGGSPCNDLSIVNPARKGLY-AEGTGRLFFEFYRLLHEAKPKEGEDRPFFWLFENVAAMGVNDKRDISRFLE---------CNPVMIDAKDVS-AAHRARYF-WGNLPGMNR------------------------------------------------------------------------------------------------------------------------------------------------------------------------------------------------------------------------------------------IFGFPVHYTDVSNMSRLARQRLLGRSWSVPVIRHLFSPLKDYFSCV

>Drer_NP_001315096.1

------------------------------------------------------------------------------------------------------------------------------------------------------------------------------------------------------------------------------MPSNTVATAAAAFKSSDFNCDTNGNQMNESSMETMIIDEMPSLCTPVRKVGRPGRKRKHLHVTCYGPLNMAKQHKEAELQSSLQSQNGEVERHENKSIEGRHSTLDRENKAESTSDSSL-------SPSLENGFVSSQDSEKCDEETLPTLTKYVEHKEDESGDPSEGARG--RLRGGVGWEVSLRQRPMPRVTFQAGDPYYIS-RRTREELLAKWKLEAEKKAKLVSVMNSMED------------------------------------------------------------------------------------------------------------------HGEVESEPKKDVITVPLLFPPPMPPTPPTPPMQQQPPQPQAQHQTNLKPQPSPQQQPPPFQQQQQQQPTDPTVATTPEPVSIGDGDKT---------------------------------------------TESEYEDGRGFGINELVW--GKLRGYSWWPGRVVSW--------------RMTRRSPAAEGTRWVTWFGDGKFSVVCVEKLLPL-----SSFHNAFHQPTYNKQPMY-----KKAIFEVLQVASTRAGKTFLSCPASDDTDSSKLVDV--------------------ENKKMIEWAMSGFQPTGPKGLD------------------------------------------PPESEPYEMCEWVEPEAASYLPPPA-----KKPRKSTVTE-KPKVKEMIDERTRERLVFDVRQKCRNIEDICISCGSLYVSL---EHPLFIG-GMCQSCT---NCFLECAYQYDDDGYQSYCTICCGGREVLM-CGNNNC-CRCFCVECVDLLVGPGAAQAAIKEDPWNCYMCGLKTQYGLLERRADWPCRLQHFFANNHDQE--FEPPRLYPPVLAENRK---------------------PIRVLSLFDGIATGLLVLRELGIQVERYVASEVCEDSITVGIVRHQG-------------RIMYVGDVRQLTRKNIQEWPFDLVIGGSPCNDLSIVNPARKGLY--EGTGRLFFEFYRLLHEARPKEGDTRPFFWLFENVVAMGVSDKRDISRFLE---------CNPVMIDAKEVS-AAHRARYF-WGNLPGMNRPM--SAMCTDKLDLQ----------------------------------------------------------------------DCLE----------------HGRTAKFGKVRTI-------TTRSNSI--------KQGKDQHFPVFMN--------------------------------------------------------------------DKEDILWCTEMERVFGFPVHYTDVSNMSRLARQRLLGRSWSVPVIRHLFAPLKEYFACV

>Cmil_P_007898625.1

-----------------------------------------------------------------------------------------------------------------------------------------------------------------------------------------------------------------------------------------------------------------------MVVAPKCHSSQDNEASEQVQNGAVDRNQTGGSKQNEDKSNPENGDPGSAVPPDQNVKDQGFNEEGNEN-----------------DLEMENGCHSPKDDDDSDKEESEKLETAEKDLDDNNESARVKVEGTRRLRGQSGWESSLRQRPMQRMTFQAGDPYYIS-KRKRDEWLAKWKKESL-----------------------------------------------------------------------------------------------------------------------TSMHPPARRVNKRAAEPIDVSPLNATPLRSQEEALLPWGELERMSYFPRCAKLHSLPQTEAEKKAKLVTVMNAQEDPQTQQQPTDPASPSIATTPEP---------PVISNEVAEKNNPKVTNDEAEYE-------------------DGRGFGIGELVW--GKLRGFSWWPGRIVSW--------------WMTGRSRAAEGTRWVMWFGDGKFSVVCVEKLMPL-----STFSTAFHQPTYSKQPMY-----RKAIFEVLQVASSRAGNHFSVQAENDELDTGKAVET--------------------QTKQMIDWALEGFLPSGPKG-------------------------------------LEPPEEERNPYKEVYTESWVEPEAAAYPSPPP-----TKKARRNIEK-LKVKEIIDERTRERLVYDIRQNGR-NIEDFCLACGSQNVLL---EHPLFIG-GMCQSCKGLRNCFLECAYQYDDDGYQSYCTICCGGREVLM-CGNNNC-CRCFCVECVDLLVGPGAAQAAIKEDPWNCYMCNHKGIFGLLRRRDDWPSRLQLFFANNHDQE--FDPPKVYQPIAAEKRK---------------------PIKVLSLFDGIATGLLVLKDLGIHVERYIASEVCEDSITVGMVRHQG-------------KIMYVGDVRNVTRKHIKDWPFDLVIGGSPCNDLSIVNPARKGLF--EGTGRLFFEFYRLLHETRPKEGDNRPFFWLFENVVAMGVSDKRDISRFLE---------CNPVMIDAKEVS-AAHRARYF-WGNLPGMNRPL--VATCNDKLELQ----------------------------------------------------------------------TCLE----------------HGRTAKFNKVRTI-------TTRSNSV--------KQGKDQHFPVLMN--------------------------------------------------------------------EKEDILWCTEMERVFGFPVHYTDVSNMSRLARQRLLGRSWSVPVIRHLFAPLKEYFACV

>Locu_P_015204065.1

------------------------------------------------------------------------------------------------------------------------------------------------------------------------------------------------------------------------------MPSNATATTTTFKSSDFDYDAAANKMSEDQEQDRGKEPGNSVPLRKVGRPGRKRKQLPVNSCETSKEDVKCHASVESQSSEQVHNGDVDSHPGDTIAASSKQGRNIEVPELPDQEAQGQGYRKGRPDSSLENGFSTPPDDQDSEKDGEQVPHPEKHTEEQDESSCEKTEGTRARLRGGLGWETSLRQRPMQRITFQAGDPYYIS-KRKRDEWLAKWKMEAEKRAKLVAVMNAMEE---------------------------------------------------------------------------------------------------------------------------------------------------------------PAGNEAQKEEEEVVVNQLPPQQHTDPTVATTPEPVSAETVDKS------------IPKSTDVEPEYE---------------------------DGRGFGIGELVW--GKLRGFSWWPGRIVSW--------------WMTGRSRAAEGTRWVMWFGDGKFSVVCVEKLMPL-----SSFSNAFHQPTYNKQPMY-----KKAIYEVLQVASSRAGKIFPTCPDTDETDTSKSVDL--------------------QNKQMIEWAMGGFQPTGPKGLE-------------------------------------PPEEERNPYKEVYTEMWVEPEAAAYTPPPP-----AKKPRKSTAE-KPKIKEIIDERTRERLVYEVRQKCRNIEDICISCGSLNVSL---EHPLFLG-GMCQNCK---NCFLECAYQYDDDGYQSYCTICCGGREVLM-CGNNNC-CRCFCVECVDLLVGPGAAQAAIKEDPWNCYMCGTKGIYGLLKRREDWPSRLQHFFANNHDQD--FDPPKLYPPVSAEKRK---------------------PIRVLSLFDGIATGLLVLKDLGIQVERYIASEVCEDSITVGIVRHQG-------------RIMYVGDVRNVTRKHIQEWPFDLVIGGSPCNDLSIVNPARKGLY--EGTGRLFFEFYRLLHEARPKEGDDRPFFWLFENVVAMGVSDKRDISRFLE---------CNPVMIDAKEVS-AAHRARYF-WGNLPGMNRPL--AAMTNDKLDLQ----------------------------------------------------------------------DCLE----------------HGRTAKFSKVRTI-------TTRSNSI--------KQGKDQHFPVYMN--------------------------------------------------------------------EKEDILWCTEMERVFGFPVHYTDVSNMSRLARQRLLGRSWSVPVIRHLFAPLKEYFACL

>Lcha_P_014343300.1

------------------------------------------------------------------------------------------------------------------------------------------------------------------------------------------------------------------------------MPSNATTPTTTSTSTISTITTTSSPALEQKPSETQKSEEKPEENRNKEEKQGNGTAMKKMGRPGRKRKQVESREISKEGVMAPKCHSTQASKGSGQVQNGDLLKSQ---QETGSSQQREEKSVPGNTEQNITGGECLSDGTHVTGNR--------------------------EVGNSIENGCCSPKDGQDSEKGDSDVPSPPK-----------------------------------------------------------------------------------------------------------------------------------------------KKRGRRKLSETAEKKKEERDDPELDSVKTEAEKRARLAAVMNALEEGAESDAQKEEEEASPPTSLQPTAPASPIVAPTPEPVLADAVEKS-----------TSKSVTDDEAEYE---------------------------DGRGFGIGELVW--GKLRGFSWWPGRIVSW--------------WMTGRSRAAEGTRWVMWFGDGKFSVVCVEKLMPL-----SSFSNAFHQTTYNKQPMY-----RKAIYEVLQVASTRAGKIFPACPESDETDTSKAIDI--------------------QNKQMVEWAFEGFQPTGPKGLE-------------------------------------PPEEERNPYKEVYTEMWVESEAAAYAPPPP------AKKPRKSTE-KPKVKEIIDERTRERLIYEIRQKCRNIEDLCIACGSLNVSL---EHPLFIG-GMCQNCK---NCFLECAYQYDDDGYQSYCTICCGGREVLM-CGNNNC-CRCFCVECVDLLVGPGAAQAAIKEDPWNCYMCGHKGIYGLLRRREDWPSRLQLFFANNHDQE--FDPPKLYPPVPAEKRK---------------------PIRVLSLFDGIATGLLVLKDLGIQVERYIASEVCEDSITVGMVRHQG-------------KIMYVGDVRNVTRNHIEEWPFDIVIGGSPCNDLSIVNPARKGLF--EGTGRLFFEFYRLLHEARPKIGDNRPFFWLFENVVAMGVSDKRDISRFLE---------CNPVMIDAKEVS-AAHRARYF-WGNLPGMNRPL--VATVNDKLELQ----------------------------------------------------------------------ECLE----------------HGRVAKFSKVRTI-------TTRSNSI--------KQGKDQHFPVFMN--------------------------------------------------------------------EKEDILWCTEMERVFGFPVHYTDVSNMSRLARQRLLGRSWSVPVIRHLFAPLKEYFACV

>tro_P_004919670.1

------------------------------------------------------------------------------------------------------------------------------------------------------------------------------------------------------------------------------ILNTSNPPAETDAPTHKGDTATEENKSKEEKSDKGVSMKKTGRPGRKRKHSMAESATVSKDIASVPKCHPNSTRPDPIHNGEMDSRQHKAKRPTQDRPAPELSPRDKDSDVTPVVENGCCTPSD--DIDPQDMEDCDVSVISTPKKRGRRSDDNSDSPKEEASTSQHRNSHCRRLRGGLSWETSLRQRPMQRLPFQAGDPYYIS-KRKRDEWLARWKREAEKKQQLAAVINAMEE---------------------------------------------------------------------------------------------------------------------------------------------------------------TVSREPQKEEEPCPPASQQPTDPASPTVATTPEPVAPDAVDKN------------TSKSADDEAEYE---------------------------DGRGFGIGELVW--GKLRGFSWWPGRIVSW--------------WMTGRSRAAEGTRWVMWFGDSKFSVVCVEKLLPL-----SSFAGTFHQATYNKQPMY-----RKAIYEVLQVASTRARKVFPSCPENDESDTSKAVEI--------------------QNKQMIEWALGGFQPSGPKGLE-------------------------------------PPEEERNPYKEVYTEMWVEPEAAAYTPPPP------AKKPRKSTE-KPKVKEIIDERTRERLVYEVRQKCRNIEDICISCGSLNVTL---EHPLFIG-GMCQNCK---NCFLECAYQYDDDGYQSYCTICCGGREVLM-CGNNNC-CRCFCVECVDLLVGPGAAQAAIKEDPWNCYMCGHKGIYGLLKRRDDWPSRLQLFFANNHDQE--FDPPKLYPPIAAEKRK---------------------PIRVLSLFDGIATGLLVLKDLGIHIERYIASEVCEDSITVGMVRHQG-------------KIMYVGDVRNITRKHIQEWPFDLVIGGSPCNDLSIVNPARK-----EGTGRLFFEFYRLLHEARPKEGDNRPFFWLFENVVAMGVSDKRDISRFLE---------SNPVMIDAREVS-AAHRARYF-WGNLPGMNRPL--VSTINDRLELQ----------------------------------------------------------------------DCLE----------------HGRLAKFSKVRTI-------TTRSNSI--------KQGKDQHFPVYMN--------------------------------------------------------------------EKEDILWCTEMERVFGFPVHYTDVSNMSRLARQRLLGRSWSVPVIRHLFAPLKEFYACV

>Mmus_NP_001258682.1

------------------------------------------------------------------------------------------------------------------------------------------------------------------------------------------------------------------------------PSSGPGDTSSSSLEREDDRKEGEEQEENRGKEERQEPSATARKVGRPGRKRKHPPVESSDTPKDPAVTTQPMAQDSGPSDLLPNGDLEKRSEPQPEEGSPAAGQKGAE-GEGTETPPEASRAVENGCCVTKEGRGASAGEGKEQKQTNIES----------------------RLRGGLGWESSLRQRPMPRLTFQAGDPYYIS-KRKRDEWLARWKREAEKKAKVIAVMNAVEE--------------------------------------------------------------------------------------------------------------------------------------------------------------NQASGESQKVEEASPPAVQQPTDPASPTVATTPEPVGGDAGDKN------------ATKAADDEPEYE---------------------------DGRGFGIGELVW--GKLRGFSWWPGRIVSW--------------WMTGRSRAAEGTRWVMWFGDGKFSVVCVEKLMPL-----SSFCSAFHQATYNKQPMY-----RKAIYEVLQVASSRAGKLFPACHDSDESDSGKAVEV--------------------QNKQMIEWALGGFQPSGPKGLE-------------------------------------PPEEEKNPYKEVYTDMWVEPEAAAYAPPPP-----AKKPRKSTTE-KPKVKEIIDERTRERLVYEVRQKCRNIEDICISCGSLNVTL---EHPLFIG-GMCQNCK---NCFLECAYQYDDDGYQSYCTICCGGREVLM-CGNNNC-CRCFCVECVDLLVGPGAAQAAIKEDPWNCYMCGHKGTYGLLRRREDWPSRLQMFFANNHDQE--FDPPKVYPPVPAEKRK---------------------PIRVLSLFDGIATGLLVLKDLGIQVDRYIASEVCEDSITVGMVRHQG-------------KIMYVGDVRSVTQKHIQEWPFDLVIGGSPCNDLSIVNPARKGLY--EGTGRLFFEFYRLLHDARPKEGDDRPFFWLFENVVAMGVSDKRDISRFLE---------SNPVMIDAKEVS-AAHRARYF-WGNLPGMNRPL--ASTVNDKLELQ----------------------------------------------------------------------ECLE----------------HGRIAKFSKVRTI-------TTRSNSI--------KQGKDQHFPVFMN--------------------------------------------------------------------EKEDILWCTEMERVFGFPVHYTDVSNMSRLARQRLLGRSWSVPVIRHLFAPLKEYFACV

>Hsap_NP_072046.2

------------------------------------------------------------------------------------------------------------------------------------------------------------------------------------------------------------------------------PSSGPGDTSSSAAEREEDRKDGEEQEEPRGKEERQEPSTTARKVGRPGRKRKHPPVESGDTPKDPAVISPSMAQDSGASELLPNGDLEKRSEPQPEEGSPAGGQKGAEGEGAAETLPEASRAVENGCCTPKEGRGAPAEAGKEQK------ETNIESMKMEGSRG--------RLRGGLGWESSLRQRPMPRLTFQAGDPYYIS-KRKRDEWLARWKREAEKKAKVIAGMNAVEE--------------------------------------------------------------------------------------------------------------------------------------------------------------NQGPGESQKVEEASPPAVQQPTDPASPTVATTPEPVGSDAGDKN------------ATKAGDDEPEYE---------------------------DGRGFGIGELVW--GKLRGFSWWPGRIVSW--------------WMTGRSRAAEGTRWVMWFGDGKFSVVCVEKLMPL-----SSFCSAFHQATYNKQPMY-----RKAIYEVLQVASSRAGKLFPVCHDSDESDTAKAVEV--------------------QNKPMIEWALGGFQPSGPKGLE-------------------------------------PPEEEKNPYKEVYTDMWVEPEAAAYAPPPP------AKKPRKSTAEKPKVKEIIDERTRERLVYEVRQKCRNIEDICISCGSLNVTL---EHPLFVG-GMCQNCK---NCFLECAYQYDDDGYQSYCTICCGGREVLM-CGNNNC-CRCFCVECVDLLVGPGAAQAAIKEDPWNCYMCGHKGTYGLLRRREDWPSRLQMFFANNHDQE--FDPPKVYPPVPAEKRK---------------------PIRVLSLFDGIATGLLVLKDLGIQVDRYIASEVCEDSITVGMVRHQG-------------KIMYVGDVRSVTQKHIQEWPFDLVIGGSPCNDLSIVNPARKGLY--EGTGRLFFEFYRLLHDARPKEGDDRPFFWLFENVVAMGVSDKRDISRFLE---------SNPVMIDAKEVS-AAHRARYF-WGNLPGMNRPL--ASTVNDKLELQ----------------------------------------------------------------------ECLE----------------HGRIAKFSKVRTI-------TTRSNSI--------KQGKDQHFPVFMN--------------------------------------------------------------------EKEDILWCTEMERVFGFPVHYTDVSNMSRLARQRLLGRSWSVPVIRHLFAPLKEYFACV

>Btau_NP_001193431.1

------------------------------------------------------------------------------------------------------------------------------------------------------------------------------------------------------------------------------PASGPGDTSGSALEREEERKEGEEQEEARAKEERQEPSTTARKVGRPGRKRKHPPVESSDTPKDPAVTSLSMAQDSGPSELLPNGDLEKRSEPQPEEGSPAGGQKGAEGEGATETPPEASRAVENGCCTPKDGRGAPAEEGKEQK------ETNIESMKMEGSRG--------RLRGGLGWESSLRQRPMPRLTFQAGDPYYIS-KRKRDEWLARWKREAEKKAKVIAVMNAVEE--------------------------------------------------------------------------------------------------------------------------------------------------------------NQGSTESQKVEEASPPAVQQPTDPASPTVATTPEPVGADAGDKN------------ATKAADDEPEYE---------------------------DGRGFGIGELVW--GKLRGFSWWPGRIVSW--------------WMTGRSRAAEGTRWVMWFGDGKFSVVCVEKLMPL-----SSFCSAFHQATYNKQPMY-----RKAIYEVLQVASSRAGKLFPMCHDSDESDTAKAVEV--------------------QNKQMIEWALGGFQPSGPKGLE-------------------------------------PPEEEKNPYKEVYTDMWVEPEAAAYAPPPP------AKKPRKSTTEKPKVKEIIDERTRERLVYEVRQKCRNIEDICISCGSLNVTL---EHPLFIG-GMCQNCK---NCFLECAYQYDDDGYQSYCTICCGGREVLM-CGNNNC-CRCFCVECVDLLVGPGAAQAAIKEDPWNCYMCGHKGTYGLLRRRDDWPSRLQMFFANNHDQE--FDPPKVYPPVPAEKRK---------------------PIRVLSLFDGIATGLLVLKDLGIQVDRYIASEVCEDSITVGMVRHQG-------------KIMYVGDVRSVTQKHIQEWPFDLVIGGSPCNDLSIVNPARKGLY--EGTGRLFFEFYRLLHDARPKEGDDRPFFWLFENVVVLGVSDKRDISRFLE---------SNPVMIDAKEVS-AAHRARYF-WGNLPGMNRPL--ASTVNDKLELQ----------------------------------------------------------------------ECLE----------------HGRIAKFSKVRTI-------TTRSNSI--------KQGKDQHFPVFMN--------------------------------------------------------------------EKEDILWCTEMERVFGFPVHYTDVSNMSRLARQRLLGRSWSVPVIRHLFAPLKEYFACV

>Acar_P_016853309.1

------------------------------------------------------------------------------------------------------------------------------------------------------------------------------------------------------------------------------TSSSSVADREAADARKQGEEEADENRNKDEKPDPGIPARKAGRPGRKRKQPVAETSEAPKDTASAPKCPSAHHSSSSSPMEQVPNGDVEAGSPEKATTAAATAEPKRSESEPSALPERETGRTLENCCTPKDSPEAPASEG----------KEEKEENHYNSLKMESRG----RLRGGLGCESNLRPRPMPRLTFQAGDPYYIS-KRKRDEWLARWKR---------------------------------------------------------------------------------------------------------------------------------------------------ETDNPPATQLDKAEKKAKVIAGMNAVEEALPRSEPQKEEEASPPASQQPTDPASPNVATTPEPLGPDAADKG------------MSKTADDEPEYE---------------------------DGRGFGIGELVW--GKLRGFSWWPGRIVSW--------------WMTGRSRAAEGTRWVMWFGDGKFSVVCVEKLLPLSSFANA-----FHQATYNKQPMY-----RKAIYEVLQVASSRSGKIFPSCPENDETDTSKAVEI--------------------QNKQMIEWALGGFQPSGPKGLE-------------------------------------PPEEERNPYKEVYTEMWVEPEAAAYAPPPP-----AKKPRKSTAE-KPKVKEIIDERTRERLVYEVRQKCRNIEDICISCGSLNVTL---EHPLFIG-GMCQNCK---NCFLECAYQYDDDGYQSYCTICCGGREVLM-CGNNNC-CRCFCVECVDLLVGPGAAQAAIKEDPWNCYMCGHKGIYGLLRRRDDWPSRLQMFFANN--HDQEFDPPKVYPPVPAERRK---------------------PIRVLSLFDGIATGLLVLKDLGIQVDRYIASEVCEDSITVGMVRHQG-------------KIMYVGDVRNVTQKHIQEWPFDLVIGGSPCNDLSIVNPARKGLY--EGTGRLFFEFYRLLHEARPKEGDERPFFWLFENVVAMGVSDKRDISRFLE---------SNPVMIDAKEVS-AAHRARYF-WGNLPGMNRPLASTVND--KLELQ----------------------------------------------------------------------ECLE----------------HGRIAKFSKVRTI-------TTRSNSI--------KQGKDQHFPVFMN--------------------------------------------------------------------EKEDILWCTEMERVFGFPVHYTDVSNMSRLARQRLLGRSWSVPVIRHLFAPLKEYFACV

>Cpic_P_005284191.1

------------------------------------------------------------------------------------------------------------------------------------------------------------------------------------------------------------------------------AAPDRETLDAHKQREEELEENRSKDEKQEPGTATRKAGRPGRKRKHALVESSEASKDTASVPKCHPPCQGSTEQVLNGDLETDGAQWKGPEDSDGTPKGRQHGE-----DETDSLPDSETGQALENRCTPKDSPDPPADEGELAPSAPQKKKEEKEEANYDSLKMESRG----RLRGGLGWESSLRQRPMQRLTFQAGDPYYIS-KRKRDEWLARWKRETDNPSASQL----------------------------------------------------------------------------------------------------------------------------------------------------DKAEKKAKVIAVMNVVEESPRAEPQKEEEASPPASQQPTDPASPNVATTPEPVVADSVDKS------------TSKSADDEPEYE---------------------------DGRGFGIGELVW--GKLRGFSWWPGRIVSW--------------WMTGRSRAAEGTRWVMWFGDGKFSVVCVEKLLPL-----SSFSNAFHQATYNKQPMY-----RKAIYEVLQVASSRAGKIFPACPENDETDTSKAVEI--------------------QNKQMIEWALGGFQPSGPKGLE-------------------------------------PPEEERNPYKEVYTEMWVEPEAAAYPPPPP-----AKKPRKSTTE-KPKVKEIIDERTRERLVYEVRQKCRNIEDICISCGSLNVTL---EHPLFIG-GMCQNCK---NCFLECAYQYDDDGYQSYCTICCGGREVLM-CGNNNC-CRCFCVECVDLLVGPGAAQAAIKEDPWNCYMCGHKGIYGLLRRRDDWPSRLQMFFANNHDQE--FDPPKVYPPVPAEKRK---------------------PIRVLSLFDGIATGLLVLKDLGIQVDRYIASEVCEDSITVGMVRHQG-------------KIMYVGDVRNVTQKHIQEWPFDLVIGGSPCNDLSIVNPARKGLY--EGTGRLFFEFYRLLHEARPKEGDDRPFFWLFENVVAMGVSDKRDISRFLE---------SNPVMIDAKEVS-AAHRARYF-WGNLPGMNRPL--ASTVNDKLELQ----------------------------------------------------------------------ECLE----------------HGRIAKFSKVRTI-------TTRSNSI--------KQGKDQHFPVFMN--------------------------------------------------------------------EKEDILWCTEMERVFGFPVHYTDVSNMSRLARQRLLGRSWSVPVIRHLFAPLKEYFACV

>Ggal_NP_001020003.1

-----------------------------------------------------------------------------------------------------------------------------------------------------------------------------------------------------------------------------------------------------------------------------MVESSDTPKDTAAVPKCPPPCPSPAEPLPNGDLEADGAQWKGTEEGGASPKSGRPEE-----DETESLADGETGRALENRCTPKEGLDAPADEGELAPSDPQKKKEEKEENNFDSLKMERG-----RLRGGLGWESSLRQRPMQRHTFQAGDPYYIS-KRKRDEWLARWKREAEKKAKVIAVMNVVEE---------------------------------------------------------------------------------------------------------------------------------------------------------------TPRAEPQKEEEASPPASQQPTDPASPNVATTPEPVVADAVDKN------------TSKSADDEPEYE---------------------------DGRGLGIGELVW--GKLRGFSWWPGRIVSW--------------WMTGRSRAAEGTRWVMWFGDGKFSVVCVEKLLPLSSFSSA-----FHQATYNKQPMY-----RKAIYEVLQVASSRAGKIFPACPENDETDTSKVVEI--------------------QNKQMIEWALGGFQPSGPKGLE-------------------------------------PPEEERNPYKEVYTEMWVEPEAAAYAPPPP-----AKKPRKSTTE-KPKVKEIIDERTRERLVYEVRQKCRNIEDICISCGSLNVTL---EHPLFIG-GMCQNCK---NCFLECAYQYDDDGYQSYCTICCGGREVLM-CGNNNC-CRCFCVECVDLLVGPGAAQAAIKEDPWNCYMCGHKGVYGLLRRREDWPSRLQMFFANN--HDQEFDPPKVYPPVPAEKRK---------------------PIRVLSLFDGIATGLLVLKDLGIQVDRYIASEVCEDSITVGMVRHQG-------------KIMYVGDVRNVTQKHIQEWPFDLVIGGSPCNDLSIVNPARKGLY--EGTGRLFFEFYRLLHEARPKEGDDRPFFWLFENVVAMGVSDKRDISRFLE---------SNPVMIDAKEVS-AAHRARYF-WGNLPGMNRPLASTVND--KLELQ----------------------------------------------------------------------ECLE----------------HGRIAKFSKVRTI-------TTRSNSI--------KQGKDQHFPVFMN--------------------------------------------------------------------EKEDILWCTEMERVFGFPVHYTDVSNMSRLARQRLLGRSWSVPVIRHLFAPLKEYFACV

>Ptep_XP_021002349.1

---------------------------------------------------------------------------------------------------------------------------------------------------------------------------------------------------------------------------------------------------------------------------------------------------------------------------------------------------------------------------------------------------------------------------------------------------------------------------------------------------------------------------------------------------------------------------------------------------------------------------------------------------------------------------------------------------------------------------------------------------------------------------------------------------------------------------------------------------------------------------------------------------------------------------------------------------------------------------------------------------------------------------------------------------------------------------------------------------------------------MSSTKRKNNESLLSQQ-------------------------------------------------------------------------------------AISATTIPIGC-----VV----------WVVSESSGDKEWW----PA-----------------------ITLRFTDCY-------KPPEEDHTWI---------------FWFADYKISKVSV--QD-------IIEFTAGYTDLIGRVINETHLKALNE----ALQILAQRSNLDYQNSEELTN-WADFEGIIR----ESNDLPDMVLK----------------------------------------------------------------------KLER-------------------KDLGKE-----------FSDDNKS---------EGNSGVFEKIENGLID----------------------------------------------------------------IEKMCIACRKEDAEVKHP--FFKGGL--CIACK----ENW----MQTIFAIGDDGV---

>Smar_7615

------------------------------------------------------------------------------------------------------------------------------------------------------------------------------------------------------------------------------NKRTINEVYDDQDYETIYKLKTCSVVLKDCLSSSKKKRSNQSVGEDGFYSPEKWKNTHDRQDKSFATSSRVRVPSRCSHCAQFLTDNIISLEEDINAIDEETALQST--LIIDGEDQDKYCKLTNFTVHDKLNHVCPFDTGLIEKGKQLFVVGNIKPICDENSDDFIRA----SLGPIIQWWVSGFDGGKKVLVGISTKFAHYYLTKPSEIYEPTMKVVMEKTYLTKLVVEFLEN------KEDPTYESLLDYLENVDSLEGHEKL----NEDKLLYYAPFVTEQIETYDSVADDDESAL--IETECMKQLMEIAGIII---------------------DGKSGAACNIRKTFKGAPKSKVALKPTTTPIVQRVFANE-----------------EIEDEHEDAEIINSSPCTRRTEKL------------------------------------------------------------------RLKTRCKWIGCSIRCNGKL----YYNSAEING-TKFSCGDCALVQTEGNSNSS-THPARIISMWED-----VFSNLKLFHVQWFAFGADTVLGDIA-NEYELFILDQ-CENLPLKHIKCKCE--IEFKSTPVNWSWLGGEDINVVQS-----------------PKYFYRKF-YQPVSGRFEDPPLEYTDYSEKKYIGHCGCCDRLKASAQQKMVQFGPKL---SEFDGENDYQFIKWKNEEFKVGDFVLIQPDAFEF--------------NRKQCQKVKIKKIEKVDEKIYPEFYRKAVPK-----DIEI-PDPYKIGFIIRIYCS--------VLAANNTQCRTHPSAIQVQVQKLYRPENTHLGIAASYHSDLNELYWSEEGAIVNLTQVRGRCFVNYCED----ISNSHLYWDAGPNYFYFHEAYDAENRLFHQPPMSICESLSKLVYSDINT------------PLRTMDVFAGVGGLSKGLHQAGV-SETRWAIELDQFAATAFKMNNPA-PELYEDNCNEILQLMIDGKRTNSKGQILPVKDVEFLCGGPPCQGFSGLNRNRNSIS------SRNNNSLVSTFLSF--CDFLRPRFFLLENIPNFIRFQNNSYVRKSFKSLLDMGYQLAFGLLNAASFGVPQKRKRLI---------------------------------------------------------------------------------------------------------------------------------------------------------------------------------------------------------------------------------------VKIFYYCCSARGGIAAFS-------------------GTF----------------VC-

>Ctel_222429

-----------------------------------------------------------------------------------------------------------------------------------------------------------------------------------------------------------------------------------------------------------------------------------------------------------------------------------------------------------------------------------------------------------------------------------------------------------------------------MQ-----------------------------------EHSEWLAEQVISYDEGR-DEDDLQL-ALCPAFRTFLRVTGITP--------KSKKLG-------KKTKPRQSLVIVKKEKMV-------ATTTAAVRQLFSKDFKSLF-----------------------GAATMS---------------------------------GDLEVIISDDEGEEDGDAPPRSAQLSRNPRGHKNRM------AGEVEW-GPLLRED---HCSSHYESATVDNVK-ISPGDYVQIEHDDSAKEP--HLGRVISLFERQDSP-----EPQVHIHWLHYTSTTLLRETG-KDNELYFSYDECQDVDLSSVMCRVNTEVSQLTVND-SHVHYG----------------------MSDERLYCVEYVYNDTTGAFTS--------PAKPKKFTCPSCAVLKARRLKESPSISDGI--------------LQYWQNEYSIGCYMFVQTE--------NKENSA-TLQDKE--K--------VVANSKFPEQWRRQNDS----GEPPR-VAPLTIGRLLGIHWADDD---------DAHE-------VVLTLQRFFRPEETSLEK--PWLKDIRLVFPSDQIFEARLDTTWGRCNVINARE-Y-LDS-LDEFLKESDNNFYYEKSYLHAEGLF---IDFELDAKLLLRRKPIKRSKRSTLMRFEISKLRSLDLFSGCGGLSLGLSQAGV-SEACWAVERDPVIAEAFKRNFPK----CCVYAADCNAML-GSILAGESCDPSGQVPLKLISAGPPCQGYSLLNKFTSSEA------YQFKNSLVVTALSF--CDYYQPKYFVMENVSTFATHKGGNLLKLVIRCLLELGY-CRFGILQAGSYGVPQSRRRFILMAAAQGLLLPAFPEPIH--AFPVAFCD--VYIDKRKFKPGL-ERIKIGHLRPLQI-----LPDVAANSTTAVHIKPTAKS-LPWSSDS------SVVFDHCVRPVDALNAARFAHIPRVRGADWRDL-PNISVPLSDGSQTP--FKG---QIEGCVR----KGVCACVEGKKCKS-ERQKNTLIPWWLPHTAQSNNHWKGVYGRLDERGCFRTIITNPQPGVKQGRVLHPSKDRIASVRECARGQGFPDDYIFVGT--IEKRYIQVGNAVPPPLAKAIGIEIRKSLI--

>Nvit_NV14091

ESTNSTTKEEKDPVEKDEAKPGGSRGKEKRTSNRKRKTSDPVALADEVKAKLKVKISSKKKKGKKSSKVFSKKSIKDKSPKEKKKTGSRSKKPPVEVPQDASVAVHTEMNSAEIVTDANIEICRVYEAQEPLQSIDNEDDKKEDQDFDSESCNYEKFMIDTQNRSNEAKEVREDQEEAMDICKNASNCTKDDLDSEKTMIEDNDDAEEVHHAMELTEVNADSSCKHEQNQESKSLKIKTLKLKTSKSSKQSSSKSSKSRDRKKSKNKSRKDSTTATTSPDPYYKVNKQDDIRICEQTLNYQLKFYTGEPENGYDEELAIIDSRLLLFND--ENISREDTRAYNKITSFSVYCNNGHLCPFDSGLIERDVEIYFSGYVKPIYSDDPSIE-NGVPGKNFGPIVEWWSTGFDIGEQPTVAISTEIGDYILMEPSDEYKPFMHSVLIKILIGKIVIEYLVY------EPHATYEDLLNKLQSASSKYDILPNM---TEDVLLEYAPFIYLQVMSYDETA--KLNEPLMLVAPCIRRLIDLAGISF--------KVNRGVSSRGQFHKYQERRAGEYRKELLRKVAENRGDNSITSQLIDSLLPDVEEKLI------------TLSKPCTRDPCGSCDRC-QRSSCKECEPCSQKN--------YEDCPRRRCFYREIQEANDEDAKDSVNLSQLKGNSSISVFKKTI-NIFSSFNAKLEWLGDPLIVEGSGNT--FYAAVKHEE-ETVCVGDFVLVRSVD--QSVPAQVVKIAYMWED------NVGLAHFHANLYWWGKDTVLGELA-RPNELFSINK-CNNISVNCITERINIDERYMPRNQDVSTDEDIK-----------------------ANNIFCEKSYDPSDGSFHDLT-KPEEVSVLPTHRSCINCEGKHRRSERVEPRTVDEL---GKKEEGTLYRAVIYKDEEYIVGSCVYLKPKSLYF-----------QFPMLLDHAKSSGKISKKIDEEKYPELYRKAEDA-VIDSKFDM-PAPFDIGYITEIFTTDK---------------------LYIKVKKLYRPENTHRNE--IKQNDMNMLYWSNEVCTVSFNYVVRKCYVAHIKD---FSKSVEEWSNLGPDRFYFMQMYHDKYGCEDVPLDPPIDYPDVD-------------------AMTSLEIFAGCGGLSLGLRESGV-IKKLWAIESDVDAANTFQLNNPD-ITVLVGHPDIVLKNAMKGEFEDSDGKLLPRKDVEFLCATLPCENYKTMTDI-----------TVFKHSYIATFLSY--CDYYKPSLFILEADEKLI--KRSFVLKLTLACLVSMGYQVTFDLMQVGCYGAPQNRRRSVLLGAAPDSVLPKFPERLH--VFPKPLCELGIVIDSKKYTQDT-FWTESAPYRSVTVQDALIDLPT-TKVAITEEMNYDAKSTSLFSKIMR-----NMLENHVCKDLGPLVECRISQIPLAVGSDWRNL-PNIEMQLKDESYAKKLIYNYDDVEAGKSSNGELRGVCSCAS-GECDPRDRQHGCLIPWHLVHSARKNKHWAGIYSRLQWDGFFNGAITNPQPLGTQGPVLHPTQPRVVSVRECARAQGFPDEFVLPQKIGIHNMYRIIGRASSPFLGRAIGHEIARSLAAR

>Smar_10448

--------------------------------------------------------------------------------------------------------------------------------------------------------------------------------------------------------------------------------------------------------MSSKYETRSKRPRPSVEKWEKFRKKRHEKKVIDEARKSNEIQKYCSQMLEDVSSAPSFNSAIDEKNCELIINPSLTKIKSKNF-----------------SVYDQMFHMIQLDWGLMEK------SGYVKLVCCDDEYDDIGQAVE-NIGPLTEWWITYDEK--DAQIGVTTELGYYYLMTPSADYRVYFYPLKEKSFLVHIIINFLED------NEDITYKELIRKIEAATPPQDYEDDIEV-TEEAVRLHAEFILEQIASYDESADDVDKLSR---TDCIQHLIVLSGKII-------PEKKRMQSTSRSTSTNVQPKQTMQQKKFQDKRMKKKTQKECNTKLVNRIFDSIFQDYV-------CCLEDVNNRENSDFECEIDEIEQFEHDFYDFSSTKLEVSFNNVIEC-------------------------------------------------------------------RNEVSLFENFKIHG----------------------EVFKSISISLGEDNQHSARQNRKQGS------ASDTILGKAA--SENEYFLSNYCQTIGFKSIKCKSD---IVFKPQLSNWKSIG-----------------GEDLEEKSGRRFYYSKFHDQKTCRFEDIPE--------AFNNLDADSCPICEIKNRETQGTISLPLNEEIELNLKLYNSVKWNNIEYKIKDSVFLLSSAFGF--------------DAVPRSLPTRENFREVDEDKYPEYYRKAKSKESLST-----CEPYRIGEIVAIYTLRKE--------------------VGVRIRKFYRPENTHLGKQATYAADFNLLYMSNEETNVNISAIVSKCKVTYCRTP------SKIELTYDQQHFYYNQTYDARSEELTRSETHDEEVDEEPAETL---------------PLATLDLFCGCGGLSEGLVQSGA-AKSCWAVDEDLVAAASFKKNHPD-CVVYGYSCSTYLSMLMDPDC----ELNLPAKEVEFLCGGPPCQGFTGLNKFKDRLC------AKLKNSLITTQLSI--CDWLRPRFFLMENVQNFIRCNDSMVLKSTLRCLIAIGYQCTFGLLQAGNYGVPQRRRRVFILAAAPGEKLPQFPLPTH--VFNSH--STNSITIDGIQMIPRHNWPTLAPYRNVTVRDTLSDLPDIEDTEPNESQEYNAHIQSHYQKILRGLIRDHFYK-----RMNAITTERILRIPRTHGADWRDL-PNIEIKLSDGTWTKKLEYNHDDVRNGRSRNGSRRGICRCAEGSGNCNIYRQKGTLIPWFLVHTANDNNQWNGALGRLCLDDVFPIALTKLTISGKIGRVIHPEENRILSVRECARVQGFPDSYIFCGD--IHDRHKQIGNAVPPLLARAIGSEIRKCI---

>Tcas_TC013587

------------------------------------------------------------------------------------------------------------------------------------------------------------------------------------------------------------------------------SPAQETGDNTK----------------------------------KHKRTSTHSD----------KCK-CAQTLNQ-AVV--YTNFPDDYAEESVALTSERLNVYNGTENISFEEGDLPSHKITCFSIYDLNDHLCPLDTGLLQK-EVYLHGGYIKPIFDDDPSPE-NGIATLDMGPIHEWFIAGFDGGEKVLLGIITNYAQYV-MEPSLEYAPIFKSLQEKTVLFKFVIEFLLS----NFSRAPSYEELLAAVENSDNP-------LL-TEEFLLQNAQFVCDQIHSFDLDAGDNNDMPL-LTMPCVRTLASLAGVSF--------RKRQLM-------RNLLGNQRRTK---LQLSK------ATATPLVRQLFEQLFPNQI-----QT-----ENCQSPKKTRCGICDTC-RSPDCGQCVYCKDMVKFGGDGNMKQPCKLRRCPNLVVQELDESDDETVVTPARGREDGRRPGVSRVLS------DGPLQNFRESIDIVLG-GVNDDDTGILLN----IKTGDFVQLSPKS-ATKP-NTIARVVNIYTA--------TEPMVHVYLFYRGNETILGEVA-NPQELFASNE-CEDCPVAAIVGQAK--VVYKATPD-NWADLG--------GIECLP--------SGDDRFFYAKQ-YDSETATFTD---YVKV---RNELDSCGHCRLNEERKKIETPTFKDNK--------------VQWRNEFYEAGNSVFLDPSVYQFPIPVECTFDD-AK---------------EMNESKYPEYYRKRE---ANASCKKM-PQPFCIGLIETLCDGPAG--------------------VLMAIRVFFRPENTKGGSMLSYQSDINLLFWSTKFITVPFRKVMGKCYVVYCSD---EQK-AREWSEGGPYRYYFRQQYDPDCGEL----VPPTYSCTRLALREGDERPFG--------PLKCLDVFAGCGGLSQGFHAAGV-ANTKWAIENDKPALDTFRHNNRT----CHVFRDDCNVLL-RNVMSGKG------GPPKMIVGGPPCQGFSGMNRFNEGEY------SLFKNSLVVSLLSL--CDYYRPLIFVLENVRNFMLYKGGLILKLTLQCLVAIGY-VRISIVEAGEFGVPQARRRFILIASAPGYQLPRIPEPQH--VFLQRGSRLDIYVDGVKYTNGN-FWTQSAPYRMLHV-----LPAI-EHDDNRPQMPYDDDEGTSHFQRKM-GN----LLDHICKPIAPIVQTRIKLIPPKGGADWRNL-PNVRVQLPDGTMTR--YRT---KKQKDGEPN--RGVCACSVEKACDPNDRQSNTLIPWCLPHTADRHNNWAGVYGRLDWSGYFATTTTNPEPMGKQGRVIHPDQNRLISVRECARSQGFPDKTKFFGS--VTSKHQAVGNMCSHKMSHILGDKVDRTLLKS

>Smar_15548

------------------------------------------------------------------------------------------------------------------------------------------------------------------------------------------------------------------------------DRNEVENYLKNIGTNHTNANTFELKELSIVLEDCLSIKRKLPGLCNILNKKGKYEDVQQNETKNSLASRNCLQFLDDNLVSLTV--REDAVYETDILSDPNLNVDIH--AVCYDETSKPEYKMVDFAVYDDEMHLCPFDSGLIQNGHVLYLSGCAKPIFSDDPSSE-GGVYVSSIGPITEWWSWGYDGGSAVVIGISSEFADYYLVEPSASYAPFMESTKIKVYLSKLVIEFLEG------NENGTYADLINKIQVHTPPEGYSNF----TEENLIHHAQFIADHLLSYDQVADADENSL--CQTKCMKTLIRLAGVTI---------------RDRREITTSSPKTKNSRGLQKVVRLQPTESKATTTPLVRKVFETIFRDQI----------ASEGRLSSKLVKCRGCKKS-------DCRSCKNGKKNNSRAQT-----------------------SDSEVSDSDSEVSHSDIESASEKEFDRSSIQVTWIGNKIL---TIGNKMFYNSAKVNGELIKVG---DSVIVNPENPKSQLLLGKVMYMWED-----LKNRGKSFHCHWFCHGSDTVLGSTA-KKNLLFLIDE-CENVRLSDIKSKCT---AVYKPPPSDWALLG-----------------GTEIENDRDCYFYSKW-YQRSCARFEDPPNIYTDKGTNLKCNYCPCCEVEESARKKKMVEIGDEM---DSDSQYTYYNSVSWNDVEYGVGDHLFLSPGSFKF-------------KAKPYAPSKINWPVTEVDEEMYPEYYRKSKIAGKGHI-----QEPFCVGHVETIYCRSTG----LSQLVEPKD-------VKIRIKKMYRPENTHEGVSASFHSDFNLLYWSNEEAVVDFSDVAGKCNVTYCED----ESNDQFYENSNEHNFYFNKVYDAHSKTF---EEPSSKLRRLGDRYKRIETGR---------PLRALDVFAGAGGLSEGLHQSKM-CETLWAIEFVEVAAEAFQLNNPK-CNVYNEDCNVLLKMIMEGKTVNAKGQTLPKKDVEFLCGGPPCQGFSGMNRFTTGQY------SQFKNSLIASYLSY--CDYYRPKYFLLENVRNFLCFKENMVLKLSLQCLIEMGYQCTFGVLQAGNYGVPQSRSRAFILAAAPGEKLPSFPEPLHVFKSENK---CSVVVNDFKYTINT-QWIKSAPYRYVTVRDSLSDLPPIKNGAKIEKLPYCPKEQSHFQKLMR-----DKIYDHICKEMKPLVVARMELIPCEPGSDWRDL-PNTNFVLSDGNVTNTLIYRYNDAKHGRSKKGDLRGVCQCVEKKKCDPLYRQDNTIIPWCLPHTANRHNNWAGLYGRLELDAHFKTTITNPEPMGKQGKVLHPTQHRVISVRECARSQGFPDSYKFCGS--VLDRHKQQSDRYSHQQSCHLWLWLQH-----

>Ocar_m.109426

-----------------------------------MELQQEKGAEEKPPEAESTQEASSDPAITGSSEATV-------------------------------------------------------------------------------------------------------------------------------------------------------KSTKKRKRAVSGSGAKKKAKDG---------------------------DSPWVVESATKKQTPSEKCKVCGQYLESPDLRLFSGDPEEALDEFAVLAHEQLSLYT-GEEEGIGVDEVPQNKITDFSIYDKHFHLCPFDTGLIDKNKELFISGFVKPIYDDNPLPE-GGIPAHALGPIGSWWFAGYDGGERGLLGVTTVYGEYILMNPSEQYSPIMENVLAKMYLNKIAIEYLTD----HL--DATYEDLLNKIQTTPPPQSLSVS-SF-DEDVLQEHALFLVDQVESYDDLA-DVDQELL-LSSPCMKHLIELSSAEL--------SKRKKK-------VAQRRGEEPIRRKRKPQSK------ATTTPLVGNVFDVFFKGEM-----EEKGKQKVSSSAPRRRRCGVCEAC-QQPECGKCSACHDMVKFGGTGRSKQACVQRRCPNMAVEEGFESQEEDPEVDPKRLTARQSSRTKKGGR---RQ-EVATSW-GEPLISM---KGITYYRSVTVDNQE-VSINDFVFVQPVN-THVP-LYIAQVQYMWQN--KR----GEKKFHCIWLTRGTETIIGNTA-DSGELFFADV-CSTNPLSSIRSKVE--VTYKEVPE-RWAELG--------GMEVAEMHVD----EDRVSFFYQKW-YDSDCARFEDLPEKIGEIDRVKEPDRCPSCKRNAQKAEYETAQAEERVED------RILYRSFRLFGETFSVNDCSYMMPDAFKFSVR-PASSKK--TLKRP--T--------DYDEDLYPEMYRRTSDY-IKGSAEGV-AEPFRIGQIKDIFTKKSHEEEEESEEEEESDK-EGNSKVFIKITKFYRPENTHLGITASYIANWNHLYWTDEEATVNCSLIEGKCTVVYRDP-M-----AREEVDWEPNTFFFSESYDPTAKSF---GTPPAHAFPMRK-----GKGKGKAKVVASSPLRSFDVFAGCGGLSLGFHQSEL-AETRWAVEVDEPAVQSFRKNNPK-AAVFVEDCNKVLQWVLEGRKVDDLGQNLPQKDVELLCGGPPCQGFSGMNRFNSGSY------SRFKNSLVVSLLSY--CDYYRPRYLVLENVRNFVTFKRNMVLKLTMCCLLRMGYQCTIGILQAGSYGVAQTRRRAFLLAAAPGETLPLYPEPLH--VFSPRACRVSISVDDTKYVSNV-RWVSSCPFRTITVRDTMSDLPTIFNGASAREISYRGEPESHFQRKIRGDQ--TVLMDHVCKKMNSLVEARMRRIPIHPGADWRNL-PNEEFKIGSGGDAKKLEYRHRDTKNGLSATGALRGVCTCASGKPCEATERQFSTLIPWCLPHTANRHNQWAGLYGRLDWDGYFSTTITNPEPMGKQGRVLHPKEHRVVGVRECARSQGFPDWFRFYGN--ILDCHRQVGNAVPPPLAAAIALEIKKAAQTK

>Smar_10552

------------MPRPKPKKAAGKKEPKKPTQRALRDVKKTNISRGEPDEPITVESKKHKMSESEANHEIIPNKRRKYEKYAKKSTENTEDI----------------------------------------------------------------------------------------------------------------------------------AYNHVPDVEKENLNGDKKDVTK----------------------------------MTTNSKPQIIRCTECSQIVDE-KIGIFPGDSDDAVDEVTALFNPILALEG-REMDDYVSSSLPDYKITQFTVYDVNTHICTFDTGLIEKNRKLYISGVIKPVYDDDPSPE-GGVLAKTIGPISEWWVAGYDGGNDVLVGVSTELACYYLMTPSEIYASYMKSIMRKTFLAKLVIEFLNS----DE--TGTYEDLINKIQAT-PIKGCE---SF-TEESLLHYAQFIVEQVDSYDQAA-DADENLL-NNTECMKALINLSGVSR--------EEKKYI-------RARIRNVQKK----PKDTK------ATTTPLVRELFELMFKDQI-----ET-----APKSTLRRKRCGVCEAC-QRPDCGACNNCKDMIKFGGKGTAKQVCKHRVCKNLGYVENGDVSDEEEVDENVDVNVKPKREFTIPKQ------KPKVSWVGEKIDFYS--KKKTFYKSVKINSET-YNVGDHVLVYPNS-PEVP-MFTAKIMYMFEDNFTK-----KKIFHAHWFCRCTDTILGETS-SDNEVFLVDE-CENSLLDIIMSKCE--IVYRPPPT-SWAYLG--------GEDVAELMESYGE----KKFFYKLF-YHRRTARFEDPPKEVDD-SEDKKCDFCPCCNRLHTFRNLEKLHLGNIL---SETSKTIFYESVKWHNVEYSVGSQVFISPDAFKFKAKIRVVPDK-NVIE-------------EVDEERYPEYYRKTKDR---GENIDL-QEPFKIGYVEKIYCAASK----NGLVNS-SD-------VKLVVKKFYRPENTHRGIDASLYADFNLLYWSDEEAVVDLTVVQGKCHVTYCTD---IAR-CESLWGAAEHHFHFSEAYNVESKMF---EDPPNKAKLLGSRGKGKGKGKGK-------RLKAMDIFAGCGGLSEGFHQAGI-CDTLWAVECDEMAAQAFRLNNPD-CTVLNQDCNDLLRMVIEGQTKNNQGQTLPQKDIELLCGGPPCQGFSGMNRFTAGQC------SQFKNSLIVSYLSF--CDFYRPKFFLLENVRNFVSYKQNMILKLTLSCLVRMGYQCTFGVLQAGNYGVPQSRRRAFILAAAPGEKLPNFPEPRH--VFKHSSCL--VTVDENKYVTNV-NWMDSAPYRRITVRDSLSDLPAIENGAKTEELSYTPENQSHYQKILRRNNVTRVIHDHVCKEMRPLVAARIRFIPCQPFADWRDL-PNIEMTLSDGSRTRKLEYGFEDGKNGRSSTGALRGVCRCAEKKKCDPLHRQDSTIIPWCLPHTSNRHNNWSGLYGRLDLDEHFKTTVTNPEPMGKQGKVLHPDQHRVISVRECARSQGFPDAYQFHGN--VLDRHKQVGNAVPPPLAREIGLEIRKCLYVE

>Hrob_116156

-------------------------------MSMFANSLKENSPVNNKKRKSLNDNVNRNNKISSSSANDNESEASDEKVIDELVEQAEKKM----------------------------------------------------------------------------------------------------------------------------------LDSENKDKSETDITNKELMTTN--------------------------TTEQLQLINHPKNKFIIERCTECGQILDEQEIVMYPGDSGEAVEEFVALTDSRLSLFTGDEQEIDEADERPQHRLTNFSVYDRMTHLCPFDSGLLEKNVELYFSGYIKPIYDDNPSIEAGGICTRNMGPINQWWTAGFDGGEVPVVGFSTAFAEYILLSPSDAYKPFWDAVSDKTQLSKVVIEFLKESIEKDE--QPSYEDLVNRIQ--VSDRFFLSSNRF-TEDSLLQHAQFIIDQLQSYDDTA-DTEDVLL-IIQPCIRTLVKLTGVTL--------GRKNKSFGNTQQTNRKTRQQQQIAAKTAQKKKDSK---ATVTPLVREVFDSLFKGQI-----EG-----QSSAATKRRRCGVCEAC-QMPDCGKCRSCMDMIKFGGSGRSKQACLIRRCPYMSIKEAEDDDLLDDNSGEIIKGKKRKMNHNKNSE------TEAVTWIGSPLATKYANGEKIFYLGAYIGEHI-VKIGDHVCVRPDD-PAVP-MYVAQVRSMWQEVVKRNNSYGKAMFHARWLNRGGDTVLGETS-DPKELFLVDE-CQDSPLHFIAYPIK--VDYVIQSEMDFFGSGGGASHIPGGLGCGDNKGSSDNNDDGKSFFCRLS-YDQSKARFED---FSEFDSLDVVKNFLNTCASVNEKSISETPFLDDEV-GVDELLTKFNYTTSTFN----KNGNGKFLE------FMTTITSSLK-ILNNRV--I--------KYDEDVYPELYRK-TEY-IKGSNEQV-PEPFRIALVVGFFVKTES----NSRKVKVEE-------VNVVVQKFYRPENTNRGLNPSSHKDLNLLYWSEDRLTVSFRNYTGKCTVTFFDESLQATDVLTRYFNDGSDRFYFTEMYLPDSKSL--QDVPSSQIVKELAFYKGGGKGKGK-----SKALKCLDVFAGCGGLSEGLHQAGI-ADTRWAIEKDESASQAFRLNNPS-CTVFSDDCNELLSLAISGSEFNSTGQRLPKRDVELLCGGPPCQGFSGMNRFNQREY------SKFKNSLIASYLSY--CDYYRPKYFLLENVRNFVSFKRGVVLKLALRCLISMGYQCTFGVLQAGCYGIPQTRRRAIILASAPGYKLPFYPEPMH--SFSVRGMQLTVAVDGRRFESCVSRTSGSAPFRTITVRDAMSDLPEVNNGAGHIETGYGGEPVSHFQKMSRENSYTTLLQDHVCKEMSALVLARMQHIPLAPGSDWRDL-PNLEIRLKDGTVTRKLRYTHDDKRVGKTASGNLRGVCSCVEGH-CDPAHRQFNTLIPWCLPHTGNRHNHWSGLYGRLEWDGFFSTTVTNPEPMGKQGRVLHPEQHRVVTVRECARSQGFPDTYRFYGT--VLDRHRQVGNAVPPPMARHIGFEIKKSLLSL

>Dple_EHJ76342

--------------MDNATHSTTRKRKASTRDGQLKIMFAKKRSRSPIESAEKDDTKKLKINTESHEHEVFNEKSKVVNGINSKSDDVNSQE----------------------------------------------------------------------------------------------------------------------------------SEETSPNLVSMKLNNERSLVDE-----------------------------------------------DENHNIEAKTVPEIINTMNGCNQNGDEMTRDGLENQV--QQNAIVEQDPPEPKPTA-KIPDQHGHLCPIDGGLIESDVRIYMSGYLKSICSDSPDIDEESIAVKDVGPIIEWFIHGFDGGSRNCITLSTEFGEYNLLKPSAEYTPLMDNLYEKIWLSKVVVEYLEE----YHYLQPTYEDLLEVIREHSIPDLEDK--RM-TEEMLHKHAQFVCDQVVSLEA---DEDNEPL-ITLPCMRELIKLMGIKF--------GKRK---------VRAKIDYKKIDK--KAWTK------ATTTPLVQKTFEHFFANQL-----DKT----NHELVLRRKRCGVCEAC-QLPDCGECNACRAMLKFGGHGRTKKACVRRLCPNMAVQQAEDSEIEDEEEYQQMAEKRHLDKIDDALPKLTGGSNKIIRWIGDPVKAD---ATKVYYEKVEIDGSE-LSLGDFVMVETSQ-SNIP-ALVARVTYMWKESINP----KSGYFHAEVFIRSSDTVLGEVG-DPREVFLGDRCCHGAPLSSILRKAF--VEKKETPA-DWFKLG--------GKEVVDHFFE----DDGKTYFYQKY-YERFTARFEDLPNDPECPNALRKHRFCPSCERKTRRDARDIPKISGKLSEIVKEANRFEWTTIRWRDHDYKKGCGVFLKPGTFRFKNSMINSSNG-INRVKL--D--------KVDEDIYPEYYRKTDNY-LRGSNIDT-GEPFCVGYIAAVTAAS------EGPLVIPQD-------IYIKVNVMYRPENTNN--RFPHHEDVNVVYWSDEIKEISFSAVVGPCNICYVDN-IPQQDHIYDWLEKDPSRVYFRMAFNKKSGQV---EDVPQHVKYVGRGDKGKDKGKGK--GKS--PLRTLDVFAGCGGLSEGLHRSGV-AECRWAVENLEAAAHAYSINNKN-CIVFNEDCNALLKDAMDGATHSAGGLRIPMQEVELLCGGPPCQGFSGMNRFNSREY------SNFKNSLVASYLSF--CDFYRPKYFILENVRNFVAFKKGMVLKLTLRALLDMGYQCTFGILQAGNYGVPQTRRRLIILAAAPGYKLPLYPEPTH--VFSRRACSLTTTIDGKRFVTNI-QWDESAPRRTCTIQDAMSDLPQICNGANRIEIDYGCMPETYFQRLIRSRDESAKLRDHICKNMAPLIQARMSRIPTTPGSDWRDL-PNISVALSDGTKCKVLQYRYDDIKNGRSTSGALRGVCACSAGGVCSVADKQENTLIPWCLPHTANRHNNWAGLYGRISWDGYFSTTVTDPEPMGKQGRVLHPEQNRVVSVRECARSQGFPDTYLFAGS--IQDKHRQVGNAVPPPLGAALGREIKKALALS

>Caqu_CAQU007561+CAQU009352

--------------------------------------------------------------------------------------------------------------------------------------------------------------------------------------------------------------------------------------------------------------------------------------------------------------------------------------------------------MTNFSVYDKEGHLVPFDSGLIEEDVFLFFSGYVKPVWDENPNIE-GGIAAKEVGPINEWWISGYDGGEKALVGFSTSCAEYYLMEPSEEYAPFMSQVLEKIFLSKVVIEFVQH----HE--GAEYEDLLTKLQL--------------------------------------ENQEEKV-------------------------------------------------------------------------------------------------SKAPRRKRCGICFNC-QKRDCGNCVACKDMVKFSGTGRSKQACLSRRCPNMAVQMADDSDGEDHDNESAQQKPKEKTPTKQAELPLPISPFREMSWIGEPIKIQ---NDRSYY------------------------------------------------------------------LAETA-DFHEVFPSGH-CSDWEMEKINYKVN--VFYKPIPE-NWKTLG--------GV-----DMGPEEKIDEKTFWYQKW-YDTKTGRFMD---MTERIPEPDMGKTCPHC-------------------------TLLLYQRAIWEHLDLAIGDCVYIPPDIYDYKVK-PVAPKP-EALKKK--E--------DVDEDLYPEYYRKAADGHAKGNHDGT-PEPFRIARIEYIMSRGAP---------DRDN-------VYLKVRKFYRPEDTHLGVPATAHSDLNLLYWSEEVADVKFSCVMGKCHVVYREN-I-VGS-VDEWSAKGPNRFYFAEIYNTQDKTF---EEPPMKAQAMGRIGKGKGKGKGKGKGKSSEKLSCLDVFAGCGGLSEGLHSSGV-TETKWAIEKEDIAAQAFRLNNPN-AIVFSDDCNELLKLVMEGKETNRLHQRLPQKEVDLMVGGPPCQGFSGMNRFNTKEY------SAFKNSLIVSYLSY--CDYYRPRFFILENVRNFVAFKNGMVLKLTLRCLLKMGYQCAFGILQAGHFGVPQTRRRAIIFAAAPGEKLPAFPEPQH--CFNPRACQLTIMVDEKK---------------------------------------------------IRGKQAQPLLYDHVCKDMGELIEARIAHVPTSGGSDWRDL-PNLEVRRSNNVIAKKLIYSYNDAKSGKSSTGALRGVCRCAIGKACIASDRQDNTLIPWCLPHTGHRHNNWAGLYGRLSWDGYFSTTTTNPEPLGKQGLVLHPEQHRVVSVRECARSQGFPDTYRYAGN--ILDKHRQVGNAVPPPMGTALGYEILKCLPEE

>Apis_ACYPI073296

-------------------MEIIKSGGDKGYVYVVKDTGTKRCSMKCTGELYTNLKIENPEVKTGHSHLKDVDSVKIEKALCAMKSDSHVKVGGVNGD----------------------------------------------------------------------------------------------------------------------------PEPKGVKRKCEEPSITQEQHDS--------------------------LSDNNGMHTDIPITNTKVRCKFCHK-TNTEDVCI----PSSAAPECIVLTNEVLANIY------TDNDDVVQHNLTNYSIYDENGHMCSIDSGVMERNVTIYMTGYVKPIYDNSPSIE-DGIAARELGPILEWWLSSYDGGTQALIGINTEYADYLLIEPHPKYKKYMTSVTEKIHLSREVIKNILS----SEYDDPTYEDILNCIINSVNPATGN---KF-TEEDLISHAQFVLNQVLEYDLN--DTYHLPL-AETQCIETLTQLSGAAK--------PVKALSRR-----AGQRTQYVKTDINKREFSK------ATTTELVKDVFENMFAAQL-----DIDDKE-NEGNMNKLVKTMFDDVC-------------------------NTKIKKEISDIRVN------------------------------------ANQVLWKDKV-----------------------LCEKMFVVVKNNV-STIP--LIGCIEYFFKT---K----FKNMAHIQLFMHSYETVLGETA-DPQEIFSLKN-CTNIDIKNVVRVLD--VVQKIPSK-NWFQLG--------NSKLQDLLEPVNR-KNKNSFFYQLQ-YDDKFGRFEYPKP-LEP-NTVITEGYCSNCEDFDLEQKSKEPELYEFIDK-----NISYFYKFKLFDEEYKVGSFIYLQPEIFKTPFELKKTSKN-ISNQEQ--K--------VIDETKYPEYYRKSLENTMRGSNETT-PDPFEIAEILAIYFVDWD----------RKN-------IKLIVRRMYRAEQIPIEDYAK-NSDMNMLFWSEEEFRVTYQNIRGKCYVSFISN-I--QD-PLLWSSGGPDRFYFTKKYDFIFGSIVSEHELPSEAKLTGK------------------PLRGLDIFAGCGGLSRGLEDSGL-VISNWAIECDDKAAGAFKLNNPE-ATVFVEDCNHLLKLAMAGEKSNSKNQNIPQKEVDFICGGPPCQGFSGMNRFNSGQY------SLFKNSLIVSFLSY--IDFYRPKYFVMENVRNFVSFKRSMVLKLTLRCITRMGYQCTFGILQAGNFGVPQTRRRLIIMAAAPGEKLPFYPEPIN--VFNRKSSSLSVQVGDEKFKTNC-KYVNSAPLRTLTVYDAWSDLPGISNGAYQERIPYNSTPITHLQKLLRDNQYASTLCDHICKEMSSLVQARMALIPVCEGSDWRDL-PNITVQLPEGLKTNTLLYTHHDIKNGYGPNGALRGVCTCASGDKCDPQDRQNNTIIPWCLPHTGNRHNNWAGLYGRLAWSGFCSTTITNPEPMGKQGRVLHPEQHRVVSVRECARSQGFKDSFIFCGS--IFDKHRQIGNAVPPPMGTAIGHAIIKAIQ--

>Scil_17007_1

---------MLKGQTRSAPSGSAECAQLKAAEADFKDLTEKGFVKKKWNLLESHLGPAQKKRLSQLQSDFDGGDLGESSFLTTAKEVLGQAEGGATSSKAAEAETE-------------------EAPAKLLPTKRTSDASSPEASPAAKRTRQSSRRRRVVDNSQSQDDSTVPEADDSVPPAAEVKEALSPIEEKMDTSD---------------------SKPQVVDSEVTQADTEKPDADT----------------------LKPEAADDSDTRSQASQDEGPARCPDCHQLLDSPTLKTYGGDPDNAVDEFVALADPQLCVFE-DDGGQEDINETPSHRLTDFTVYDKQGHVCPFDGGLIERNVELFFSGVVKPIYDDTTDVS-VGVLTARLGPIDVWWTAGFDGGDSALIGFSTAYADYYLTAPSKEYEHIMSLMVEKIYMSKLVIEFLEE----NP--HESYEDLVDMIQFSTPPKGCN---KF-SEDTLLRHAQFVVEQVENYDTPD-DAQDSYF-VLSPCIRALIRLAGVTL-------GGKRRGIRRP----RAPRAPRPKKEKKIASASM------AVTTPLVRNIFDVFFQDQI-----DG-----KVATSGRRERCNVCEAC-RQSDCGKCPPCRTMTKFGGDGKSKQCCVQRRCPYMELKAADEDEDLAERKEQDGEDVSDKAKSKQKRPSDQSVIKTKVQWVGEPAITK---GKKKYYKKVLINDEE-ICVGDCVALCPEN-PTDP-HYIALVAYMWDN---E----GSKTFHAVWLSRGSESILGEAA-DPGEVFLVDQ-CDDNPLGSILFKCA--LEHRAPSA-NWRMEG--------GEEEAAATSGYGD----KKFFYQKW-YDLEAARFEDPPAMLALLSKSNARPECVACTRQAAEEQRNEPTPGSEIEG-----STSFYSSFAFEGDTYSVGSGAYFLPTAFSFKYKAPVKKPA--LPVKK--E--------SVDEELYPEYYRKTWGH-IKGSNLES-PEPFRIARILQIFSQPDV-----------DT-------LQVRVQKFYRPENAKLSDAERFAADWNILYSSVEECVVSAADIQGSCSILHSQT-L-TVP-LSDYSARSTDNFYFEKEFDGTTKEI---ADAPKALPESADKTSEK-SGKGTGRKSRTSPLRSLDVFAGCGGLSEGFHQSGI-AKTVWAIEKDEAAAEAFKLNNKK-SIVFTDDCNELLRLVMSGRTENKSGQPLPQKEVDLLCGGPPCQGFSGMNRFNSREY------SQFKNSLVSSYLSY--CDYYRPRFFVLENVRNFVSYKQNMVLKLTLRCLLRMGYQCTFGILQAGSYGVPQTRRRAIIMAAAPGEKLPRYPEPTH--VFSPRACQLSVMVDDKKYCSNI-SRAESAPYHTVTVRDAISDLPAVTSGASKQQITYSAESKSPFQRVMRGDKTEGSVGDHICKDMSALVVARMELIPTTPGADWRDL-PNKVIKL-NGENIPKLRYTHPDRKNGRSSTGAVRGVCSCANGKACDPADRQFNTLVPWCLPHTGNRHNHWAGLYGRLEWDGFFSTTITNPEPMGKQGRVLHPEQNRVVTVRECARSQGFPDWFRFYGN--ILDRHRQVGNAVPPPMAAAIGREILRSVQYS

>Hmag_XP_012557244.1

---------------------------MPVLTDGREAHKACGSSKTNRQSKQSTINALFKKSVKKTKRSIS-------------------------------------------------------------------------------------------------------------------------------------------------------PSKEENENKKKRTDGDIKEKNT-----------------------------SFLTNSEKPGVKTVEKCKECRQLLNSNDIKLFQGDHSDALEEFAMLVDPRLSLLSGNEQDFDAYEDRPQHKVTEFSVYDKCGHLCAFDTGLIEKNVELFFSGYVKPIFDENPDIE-GGISTKAMGPINEWWIAGFDGGENALIGFGTAFAEYILMRPSDDYASFMNAVTEKIYMSKIVIEFLLN----NP--DSEYEELLNKLETTVPPENCA---KF-TEDTLLQHAQFLVEQVESYDSAALDEEDSPRLITSCCMRDLIKLAGVTL--------GKRRQM----------RGLKVKEEKKTLGPTL------ATTTPLVRHVFDTFFKNQI-----DL-----KGVTTQRRKRCGVCEIC-QQPDCGVCRSCKDMVKFGGSGRSKQCCINRRCPNLAVQEAEEDALCSQDEDEPMLKSPSKTDLSPRHHKGQEK-KSFVKWACEDFVER---KGKKLYKSVQINNEL-INIGEFVQVYPTD-PSDP-LYICRVMYMWED--LN----GDKKFHAQWLYRSSETVLGEVG-DPSEVFLSDD-CDDIKLGAIMSKCN--VSSKFASE-NWFMEG--------GK-----EDCIIS-EENNELFYQKW-YDYEDGLFTD-PPTEFL-LSKFDEKYCPSCERTKTKLKYKQPTLGDELETENSTKSEHCFKTVTWQGVCYSLGDCVYLDPDAFTFKIKQKSELPK-NKSVVK--D-----------EDEYPELYRKSNDY-IKGSNINI-VEPFRIGKIISIIKKTDN-------YGGMKS-------IMLKVRKFYRPENTHKGMSGTMNCDLNMVYWSNEEATVDFQMVEGKCYVLFADD-A-DMD-MFKFTEAGPDRFYFREAYDADKKEF---DVPPREAWNS-------NKGKGKGKSKSTKKLRTLDIFAGCGGLSEGLDQVGV-VNSCWAIEFEPSAAQAYRLNNPS-AIVFNQDCNNVLKQIMEGKEKDDLGQRLPRREVDLLCGGPPCQGFSGMNRFNQREY------SMFKNSLVTSYLSY--CDYFRPKFFILENVRNFVSFKKSMVLKLTLSCLVKMGYQCEFGVLQAGSYGVPQTRRRAIIIAAAPGEILPKFPEPQH--VFASKALSLSVTINSHKYQAL--KRLHSAPYRTTTVYDAMSDLPEIKNGANKMEIGYDTEPLTHFQKKIRGKHMQ-VLRDHICKDMSPLVEARMSYIPCIPGSDWRDL-PNKVVKLRDGNTTKLLLYLHKDKKQGPGKNGENRGVCACANGKPCDPSDRQFNTLIPWCLPHTGNRHNNWAGLYGRLEWDGFFSTTVTNPEPMGKQGRVLHPEQHRVVSVRECSRSQGFPDSFRFYGN--ILDKHRQIGNAVAPPMSAAIGQEIRKSIRNE

>Aque_XP_019849850.1

-----------------------------MLGGEEEELFQTSSKKKRVPRIIDDDDEMEVSRVTTAKRSNNKRKSSGTGASSNLS----------------------------------------------------------------------------------TNKSSKIQSSSTSGRQLGIGSFFNKDPQKRSKVE---------------------APTGEEEREEEEGKEKEAKKAKTSETGSSSNGTSAATNGNNKSHDLRPHDDIPANKTSEAPQSSKPPCTYCKRSSDDPRLKIFIGDPPNANDEFITLADPSLSVLS--ADQESALDDVPQHKITGFSVYDKNHHLCHFDTGLVEKNVELFFSGWVKPIYDENPDPS-DGIPTRQLGPINSWWIAGFDGGEKALVGFSTAYAEYILMDASDDYAEIMASVQEKIYLSKILIEYLEE----FP--AATYEDLLNKIQTSVPPQSIGCT-SF-TEDSLLRHAQFIVEQVESYDQYC-DEDEDLL-LVSPCMRALIKLAGVTL--------GKRLSS---------RRPHQPKVKK--TKQTK------ATTTPLVRDIFETFFKNQI-----DN-----KSSSAPRRRRCGVCETC-QQPDCGKCNACADMIKFGGTGRSKQACVNRRCPYMAVQTAEEEEDN----DAADPDLKNVKDLKSPSKKIKKI-KTKVEWIGEPDFVE---GGKSYYTEVLINNKEKVCLYDVVSVCPEV-PDDP-LYLTRIMSMYED--SN----GKKMFHGWWFHRSTDTVLGETG-DPRELFLIDD-CEDNPLGAIMDKVE--VEYKPPVS-NWFMCG--------GEEVSDDEKEIE--EDGKTFFVQKY-YDQSLARFEDIPSDAHP-PTDGFIPQCASCERRNHMKTVHSAVPINKIEK----PKITNYSSFQLNEESYSIGDCVYLSPHTYSFPNVKKTSGTA-ANKKQK--KEEE-----EFDESEYPEKYRKVSDY-VKGSNIDS-PSPFQIGQVLEIFSKSLG----GKLIDNNKI-------VHIKLRMYYRPQDTHKGDEATAQYDLNLLYWSDLTTVVAGDVVCGKCFVKFKED-I-TED-IDSYFSNKPNHFYFVEAYCADTKEF---EDPPVHAMNKG-KGKGVSKGKGKKGSKESIKLRMLDVFAGCGGLSEGFHQAGV-ADSCWAVEIDEPAAQAFRLNNSQ-TTVFTDDCNILLSLVMEGAKTNSRGQLLPQKDVELLCGGPPCQGFSGMNRFNSREY------SQFKNSLVISYLSF--CEYYRPRFFLLENVRNFVSFKKSMVLKLTLRCLVKMGYQCTFGVLQAGQYGVPQTRRRAIILAAAPGEKLPHFPNPTH--VFSPRACQLTVVVNDIKYEGSI--RMDSAPYRTITVRDSMSDLPHIKNGSAVRSMNYNGEPHCHYQRLMRGNQHQPVLYDHICKEMNPLVAARMRFIPIGPGSDWRDL-PNKCIRLSDGTTAPKLQYTHHDKKNGRAKNKSLRGVCPCATGQPCDSSYRQYGTLIPWCLPHTGNRHNHWAGLYGRLEWDGFFSTTVTNPEPMGKQGRVLHPEQHRVVSVRECARSQGFPDTFRFFGT--ILDKHRQVGNAVPPPLAKAIGLEIKRSVKKD

>Aque_XP_019859084.1

---------------------------------------------------------------------------------------------------------------------------------------------------------------------------------------------------------------------------------------------------------MLSSPRPCHILPSSQSKTKDNQPVIQLYSPPAAPQSSQPQCTYCNRSSNDLTLKTFIGDAPNANDEFIMFADPSLSVF----SAESALDDVPQHRITGFSVYDKNHHLYHFDTGLVEKNVELFFSGWVEPIYDENPDPS-DGIPMRQLGPINSWWIAGFDGGEKALVGFSTAYAEYILMDASDDYADIMASVQEKIYLSKILIEYLEE------FPAVTYEDLLNKIQTSVPPQSIGCTSF--TEDSLLRHAQFIVEQVESYDQYCDEDEDFL--LVSPCMRALIKLAGVTL---------------------------------GKRLSSRCPHQPKVKKTKQTKAIFETFFKNQI----------DNKSSSAPHRRRCGVCETC-QQPDCGKCNSCADMVKFGGTGRSKQACVNRRCPYMAVQMAEEEEDNDSADPDLKNVKDLKSPSKKIKK------KTKVEWIGKPIFVK---GDKSYYTEVLINNKEKVCL--YDVVSVCPEVLDDPLYLTRIMSMYED------SNGKKMFH-GWWFHSTGTILGETG-DPLELFLIDD-CEDNPLGAIMNKVE---VEEKPPVSNWFMCG---------------GEEVPDGEKEGKTFFVQKYYDQSLARFEDILS--------------------------------------------EYIRYLIPDDADPPTDGFIPRCASCERRTVHSAVPINKRNKRVREIKDYKLQVIPVNFDESEYPEKYRKVSDY-VKGSNIDS-PSPFQIGQVLDIFSKSS-----GGLLIDNNEI------VHIKLRMYYRPQDTHKEDEATAQYDLNLLYWSDLTTVVAGDVVCGKCFVKFKED---ITEDIDSYFSNKPNHFYFVETYCADTKEF---EDPPIHARNKGKGKGISGKGKGPESITTDDKLRMLDVFAGCGGLSEGFHQAGV-ADSCWAVEIDEPAAQAFRLNYSQ-TTVFTDDCNILLSLVMEGAKTNSRGQSLPQKDVELLCGGPPCQGFSGMNIFNSREY------SQFKNSLVISYLSF--CEYYRPQFFLLESVRNFVSFKKSMVLKLTLRCLVKMGYQCTFGVLQAGQYGVPQTRRRAIILAAAPGEKLPHFPNPTH--VFSPRACQLTVVANDIKYEGSI--RMDSAPYRTITVRDSMSDLPHIKNGSAVRSMNYNGEPHCHYQRLVRGNQHQPVLYGHICKEMSPLVAARMRYLPIGPGSDWRDL-PNKCIRLSDGTTAPKLQYTHHDKKNGRHKNKSLRGVCSCAT-GQCDSFYRYGTLIMPWCLPHTGNRHNHWAGLYGRLEWDGFFSTTVTNPEPMGKQGRVLHPEQHRVVSVKECARSQGFPDTFHFFGT--ILDKHRQVGNAVPPPLAEAIGLEIKR--SVE

>Mlei_ML002234a+ML002229a

-----------------------MVVKQTDLRQMFSAEKIQKSNEKTNIRTSAVIADEGQSSLTDIASKNVEDNCLAEG--------------------------------------------------------------------------------------------------------------------------------------------------EPASKRQKTEKYNREINES---------------------------------TKRKSPALKPVKCDVCKQLLNSEALKVFKGDESGAVEEFIALASDTLSLFDADSNGESEWTEKPQHRITNFTVYDEYGHMCPFDSGLIEKNVPLYFSGYIKPIYDDSPSLE-GGVAGSKLGPIDSWYIAGFDGGEKELIGFTTAYADYLLSQPSDQYSPIFDMLREKTYLSKTVIEILVE----DS--GMTMEDLLNCIQLVVPPENCS---KF-TEETLLRHAQFLVEQIESYDHAA-DEDEDLL-ITIPAVRGLIELAGVTL--------GGNQQS-------RARPERPVVKSKA-AGKTKSTDISHATVTPLVQSSFEAIFVDQM-----------------------------------------QATVSFFLSGFS------------------------------------------------------------------------FYRIL---------------------KFTP-TFTSQ---------SK----GRKMA----VIRAEENAKDETD-EP------NL-AQSNGLKKKLPEVK--TEKKVSSNIDGKLTG--------DPVSKSGGMPVPE-KQNEELFFKFW-YDSDRARA-----KISS--------------------------------------------------------------------------------TKIRK--D--------NVDDEMFPEYYRK-TDY-IKGNNDLV-PELYRIGRIVSFSTKQEG--------LDGSV-------VKVTVKKMYRPENIENCRVES--ADINLLFYSLDQVKIRADKLSGKCTVKNSED-I--ED-LSRYTTLS-DHFYFTESWNSASKCL---EDVPSEGRSS-------NKGKKK---RAGIKLRTLDVFAGCGGLSAGFHQAGI-AESCWAIEFFSEAAQAYKLNNPQ-AEVFNEDCNAVLRMAMEGVLTNKLGQKIPQKEVELLCGGPPCQGFSGMNRFNAREY------SMFKNSLISSYLSY--CEFYRPRYFLLENVRNFVSYKKNMVLKLCLSSLVNMGYQCTFGVLQAGCYGVAQTRRRAFLLAAAPGEVLPQFPEPRH--VFSPKAMSLSVTIDNKQYSQHI-TRFDSAPLRNITVRDIMSDLPAITNGATKRELAYDCEPDSWFQRQIRGNS--EVLTDHICKEMNPLVAIRMRHIPLTPGSDWRDL-PNISVTLPDGKKTPKLVYTHNDLKNG-----PMKGVCSCAEGKKCDPMDKQNMTLIPWCLPHTGNRHNNWAGLYGRVFWDGYFSTTITNPEPMGKQGRVLHPEQHRLVSVRECARSQGFPDTYKFYGN--LLDKHRQLGSPVDATAVKSDTVAVKRTITTS

>Hazt_HAZT006446

KSITEMFSFIKKDPQPHNTLQNGCKSEIAIPSAPFNNEVEFDSPKTPVSPNKPVLEADIERDCKTLEIKQKKDDLCDADGIVSSHARDAGNYNGNMKDCDANDSAS-EDCDANDAASVQEDKEFPESGDEMPLRLQPRDLGTRRLCASSPRPCLCASSPRPKDEMPLSAPPSPRSGDEISKNCGVIPSMSLKEDKEDCDAKEDKEDCDANDAASVQEDKEDCDSDDPGDEFEGFPSEKRLKLAE-----------------------PERSYHSSQDQKTSQPAAPKSRCPICRQILDSDSLSFYEGHPQDATEEFIALTDPKLAIFM-DDD----LEERPQHKLTSFTVYDKQGHVCPFDGGLIERNALLYTSGYIKPIFAEDPTPL-DGVPVMDVGPINEWWISGFDGGEKALLGFSTGYAEYVLMDPSPVYKQYVDAVMEKIYLSKLVIEFLYD----NE--DGGYEDLLNLLQTTVPPPDV----VL-SEDSLLRHAQFVCDQVHNFDVAG-DGSDVL--ITHPCLRTLVDLAGVTL--------GKRCAL-------KTRGPRIKTVKKM-PKWTK------ATTTPLVRRCFDQFFADQIDSHKGDNSANDTKEKSGPRRMRCGVCEAC-LKQDCGECSSCKDMIKFGGSGRSKQCCKDRRCPNMMLAEPDDENEEEAVQKLANLRDAAAQTRSHRVK---KT-TDKLCWVGEASYRT---SKRVYYSSARVNEDV-VCRGDCVQIEPDA-GVKDLPYIARVVSLWED--MK----GEKYLHADWYCRGKDTILGDTS-DPQELFIIDD-CEDIPLASVMKKVR--VVPHHPPP-NWRLLG--------GILHPEDLNPLAS-DDTHSFFCQLA-YVPQHARFIHLSSTIYENELATGSRDCPCCERLRLAELRDSCVLMEKSTD--------EYNAVQYMDQKFSVGDCILVEPSAFAFKVKLPSPASR--AQRQQ--E--------HWDEDLFPEYYRL-TQK-IKGSNEDT-SEPFRVAQILSIRPHSKSGEVFCGSALPPAS-------VTAVVRKFYRPENTHRGAAAAYQAPLNLLYWSDEESSVSFSWVCGKCTVVYAEN-L-TCS-PDEFFLQGPYRFIFSQAYDATTKEL---VEPPKSAINLGSSGKGKGKGKGKGSSNSNTRLRTLDVFSGCGGLSEGFHQAGV-AESCWAIEVFEPAANAYKLNNPN-ATVFTDDCNLLLRMAMENKTTNSKGQTLPRKDVELLCGGPPCQGFSGMNRFNSRQY------SLFKNSLVASYLSY--CDFYRPRFFLLENVRNFVSYKCGMVLQLTLRVLVQMGYQCTFGILQAGSYGLCMKKNRAVVRRLAPGEQLPYFPEPLH--SFSPHACSVSAAVADVKYKSNC-RWTVCGPLRTITVRDALSDLQPIQNGGGREQVAYTTEPESHFQRMVSGNLYAPIVRDHQCKSLSDLVAARMQRIPTAPGSDWRML-PNKPVRLPDGSWTKKLEYNHDDKRNGRSSEGHLRGVCACASGAPCDPMDRQHNTLIPWCLPHTANRHNHWAGLYGRLEWDGFFSTTVTNPEPMGK---------------------QGFPDAYRFYGS--LLEKHRQVGNAVPPPMARAIGLEIRKCIATE

>Nvit_NV17734

---------------------MSSDNESPGRSRSRSCATSMETDELQSLPPPPEEREDTSREADDEEESKPDADGAGPSYL-----------------------------------------------------------------------RPRSHSRLLENDEAG-------------------------------------------------------DRQATADHSSKRSRTELATKQS---------------------------RPKMMHTGRVAASRKEPVCEICRQKLQDKDLKMYAGHPNDAVDEYNAVIDEKLCLFNGEEEHVMQHDWRAINKITFFNVYCRNGHLCPFDSGLVEREAYIYLSGYVKPIYSDDSSIE-GAVPSKDIGPIVEWFVTGFDGGQDAIIVLSTPLGEYYLMQPSDDYSPFMRCVKEKTFISKTVIECLLD----EP--NSEYEDLLNKFETIPMPSGLP---RF-TEELLIHHAQFICDQILSFDESA-LSDEPQL-IHAPCVKSIIDLSGVTF--KKRFRRGRRRQFLENDEDWQKGLRLSKQKA---PYWSK------ATTTQQVHDLFESFFPDQL-----DNT----ADKLKLKRRRCGACEAC-LETDCGQCASCKNMIKFGGTGTSKQACVKRRCPNMQLEGDDDDYDEDDDDKANHKESSIEVHRKMIRALKKKK-SAVIEWMGEPIQS----AKGDFYNAVMINHDV-IKKNDYIFIEPIN-SSVP-MQVIKVKYMWEN--KM----GIKILHGTWLWRGSETILGETS-LPRELFLVDE-CQDVPLIYVQAKAN--VLVREYSN-DCTEKG--------NIIDALK-------ENSMILFYQKR-YDHVAARFEDLLPELNP-PKGAEHCFCSTCARDILSKMNRTPQLLELLTD----DKRIKYGIIRYLNEEFRVGSAVYLKPKTFTFEFP-NMNQDT-SRGQKR--E--------IVDEEKYPEAYRKFNDR-VKGSNVDT-PEPFDIGYITSIYSTSKS----KLLAG--VN-------AYVTVKKMYRPENTHRGESLKKKSDMNMLYWSDEECDVRLNCIVGKCYIAYSEN-L-NQS-IEEWSASGPHRFYFSEAYDLNNEEY---TEPPAHACSVTKSFKKDAKSKSK-------KLRTLDVFAGCGGLSEGLKQAGV-AESLWAIENDTAAAHAYRLNNPK-ASVFTTDCNSFLEKVINGETSLG-GQSLPKKEVDLLCGGPPCQGFSGMNRFNSRAY------SSFKNSLIVSFISF--CDYYKPRFFLMENVRNFVSFKKSAVLKLTLSCLSRMGYQCTFGILQAGSYGIPQTRRRMILIAAAPGEILPRFPNPLH--VFSKSTCQLTVDVDNVKYFTFT-DVHESAPYRAITVYDALSDLPKIKSGSNEVVMRYENDPLTHFQRKMRGMDDELALYDHICKDLGPLVEARMGLIPTKTGSDWRDL-PNIVVRLSDGTCTTKLEYKYNDKKAGVSSTGAPRGICSCSEGKSCSLRDKQVNTLIPWCLPHTANRHNHWAGLYGRIEWDGFFSTTITNPEPMGKQGRVLHPEQTRVVSVRECARSQGFPDSFRFYGS--IQDKHRQIGNAVPPPLAKAIGLEIRKSVLKE

>Nvit_NV11649
[truncated: 2,022,356 more chars]
