# Supplementary material for: DNA methylation atlas and machinery in the developing and regenerating annelid Platynereis dumerilii
Source: BMC Biol. 2021 Aug 3;19:148. doi: 10.1186/s12915-021-01074-5 (PMC8330077; doi:10.1186/s12915-021-01074-5)

A. DNMT Proteins

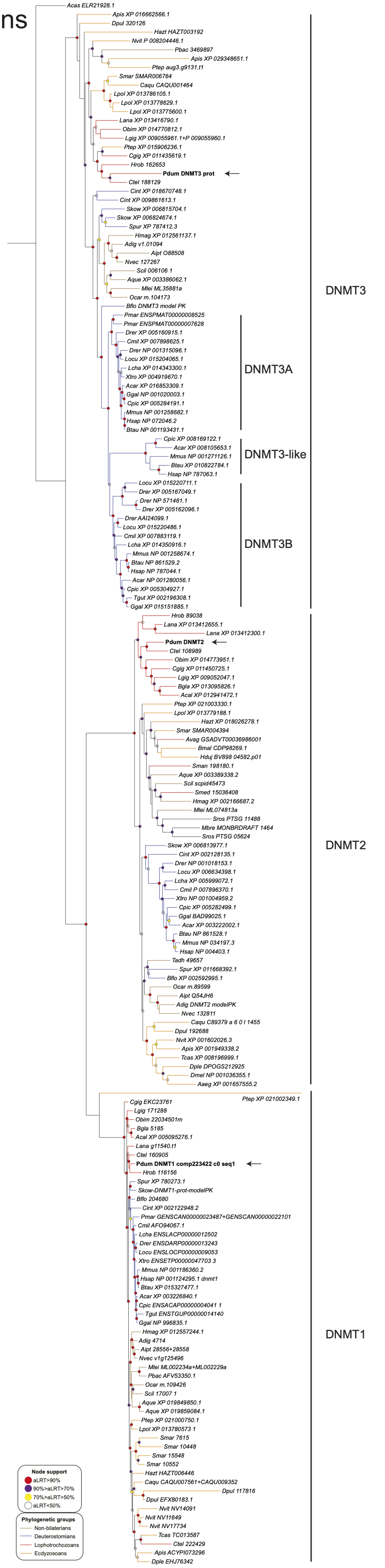

B. TET Proteins

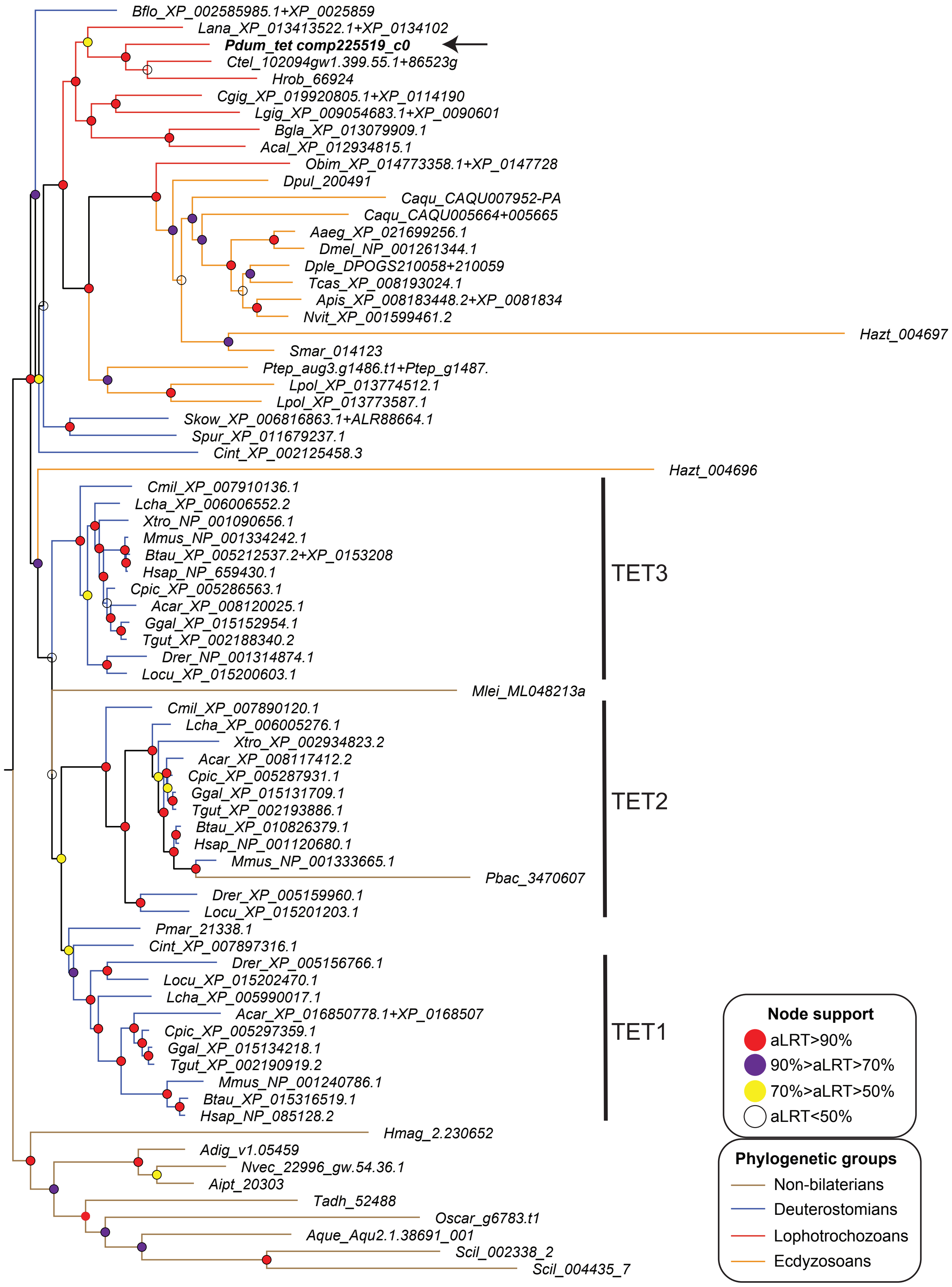

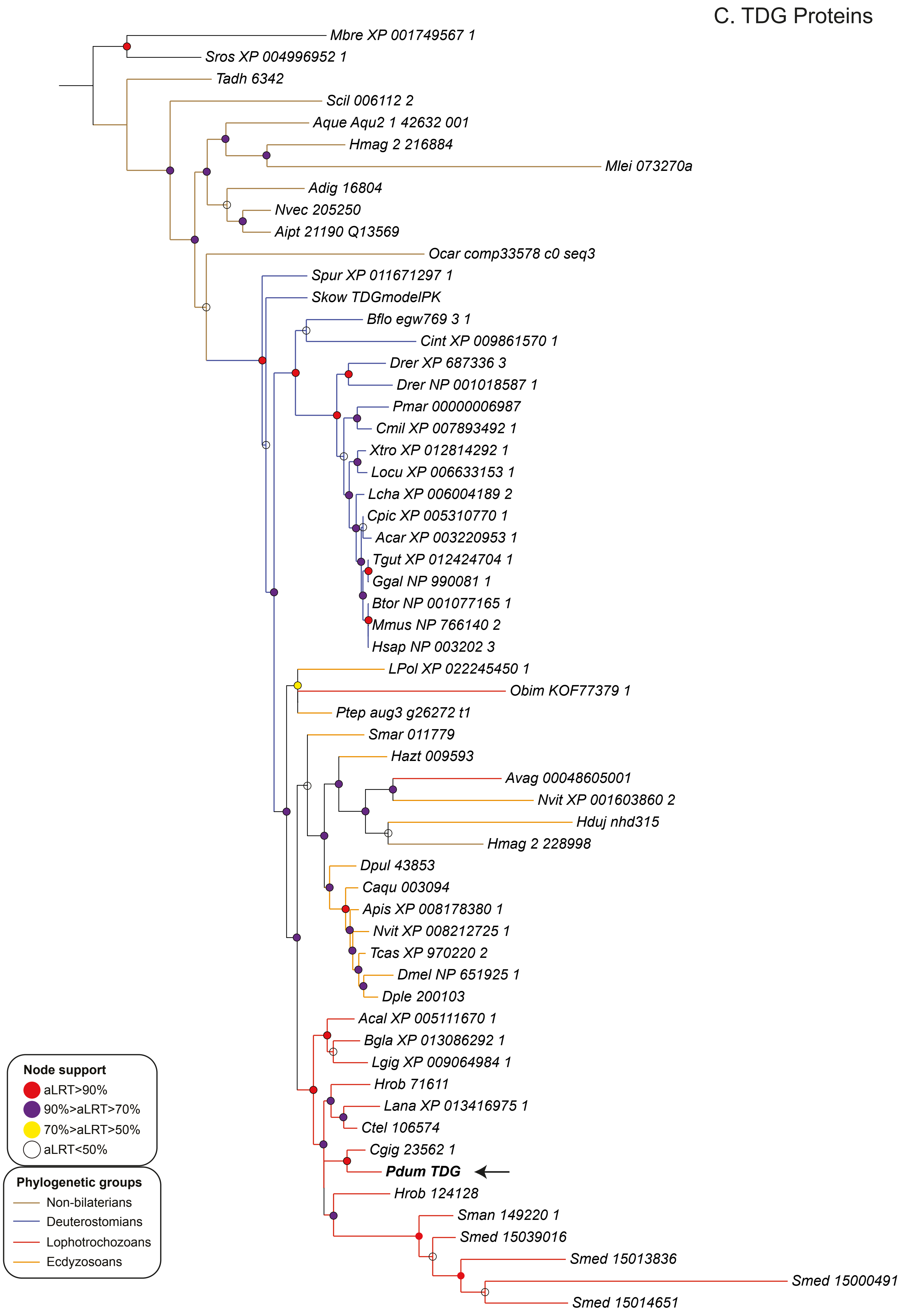
C. TDG Proteins
Mbre XP 001749567 1
Sros XP 004996952 1
Tadh 6342
Scil 006112 2
Aque Aqu2 1 42632 001
Hmag 2 216884
Mlei 073270a
Adig 16804
Nvec 205250
Aipt 21190 Q13569
Ocar comp33578 c0 seq3
Spur XP 011671297 1
Skow TDGmodelPK
Bflo egw769 3 1
Cint XP 009861570 1
Drer XP 687336 3
Drer NP 001018587 1
Pmar 00000006987
Cmil XP 007893492 1
Xtro XP 012814292 1
Locu XP 006633153 1
Lcha XP 006004189 2
Cpic XP 005310770 1
Acar XP 003220953 1
Tgut XP 012424704 1
Ggal NP 990081 1
Btor NP 001077165 1
Mmus NP 766140 2
Hsap NP 003202 3
LPol XP 022245450 1
Obim KOF77379 1
Ptep aug3 g26272 t1
Smar 011779
Hazt 009593
Avag 00048605001
Nvit XP 001603860 2
Hduj nhd315
Hmag 2 228998
Dpul 43853
Caqu 003094
Apis XP 008178380 1
Nvit XP 008212725 1
Tcas XP 970220 2
Dmel NP 651925 1
Dple 200103
Acal XP 005111670 1
Bgla XP 013086292 1
Lgig XP 009064984 1
Hrob 71611
Lana XP 013416975 1
Ctel 106574
Cgig 23562 1
Pdum TDG
Hrob 124128
Sman 149220 1
Smed 15039016
Smed 15013836
Smed 15000491
Smed 15014651
Node support
aLRT>90%
90%>aLRT>70%
70%>aLRT>50%
aLRT<50%
Phylogenetic groups
Non-bilaterians
Deuterostomians
Lophotrochozoans
Ecdyzosoans

D. UHRF Proteins

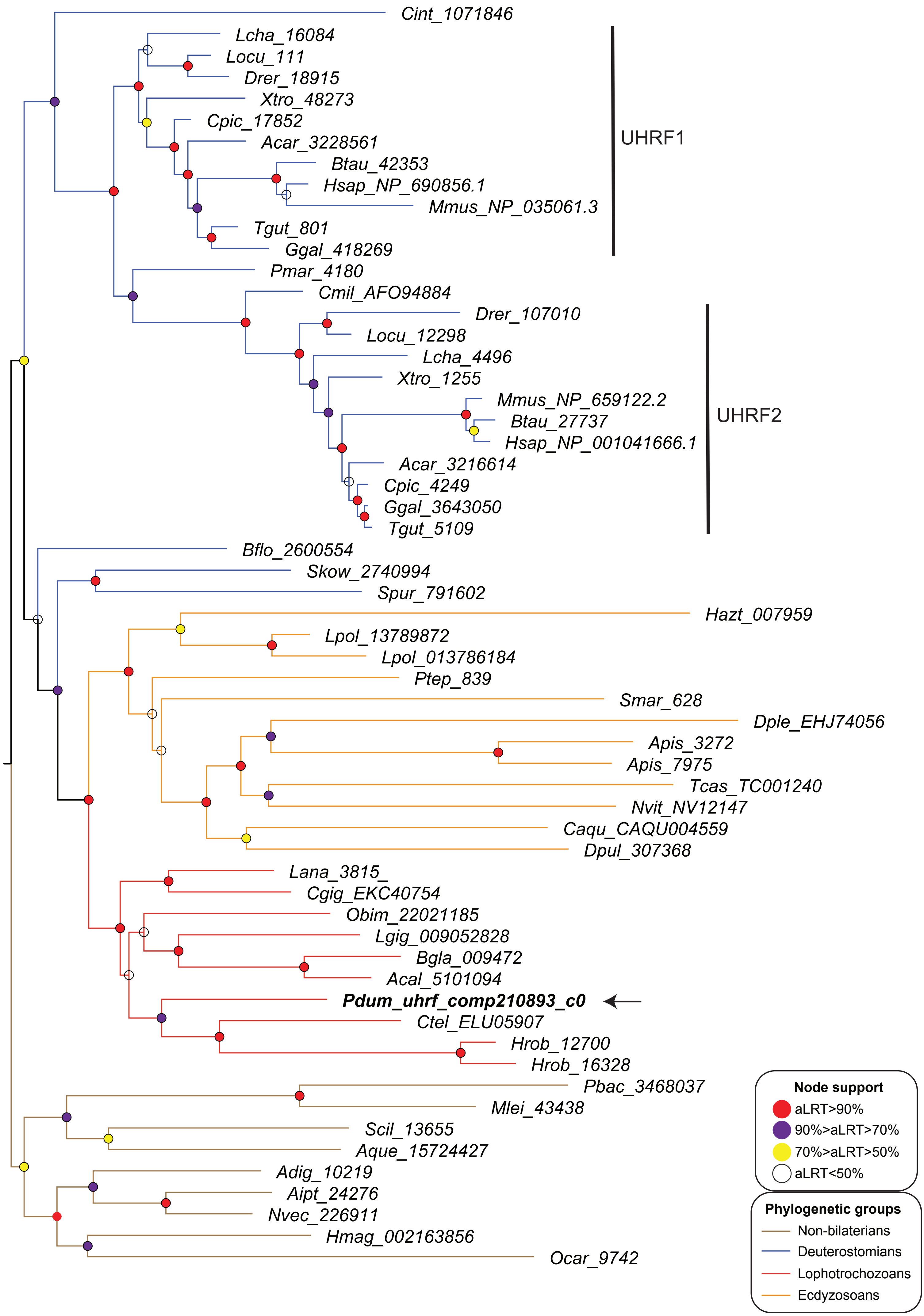

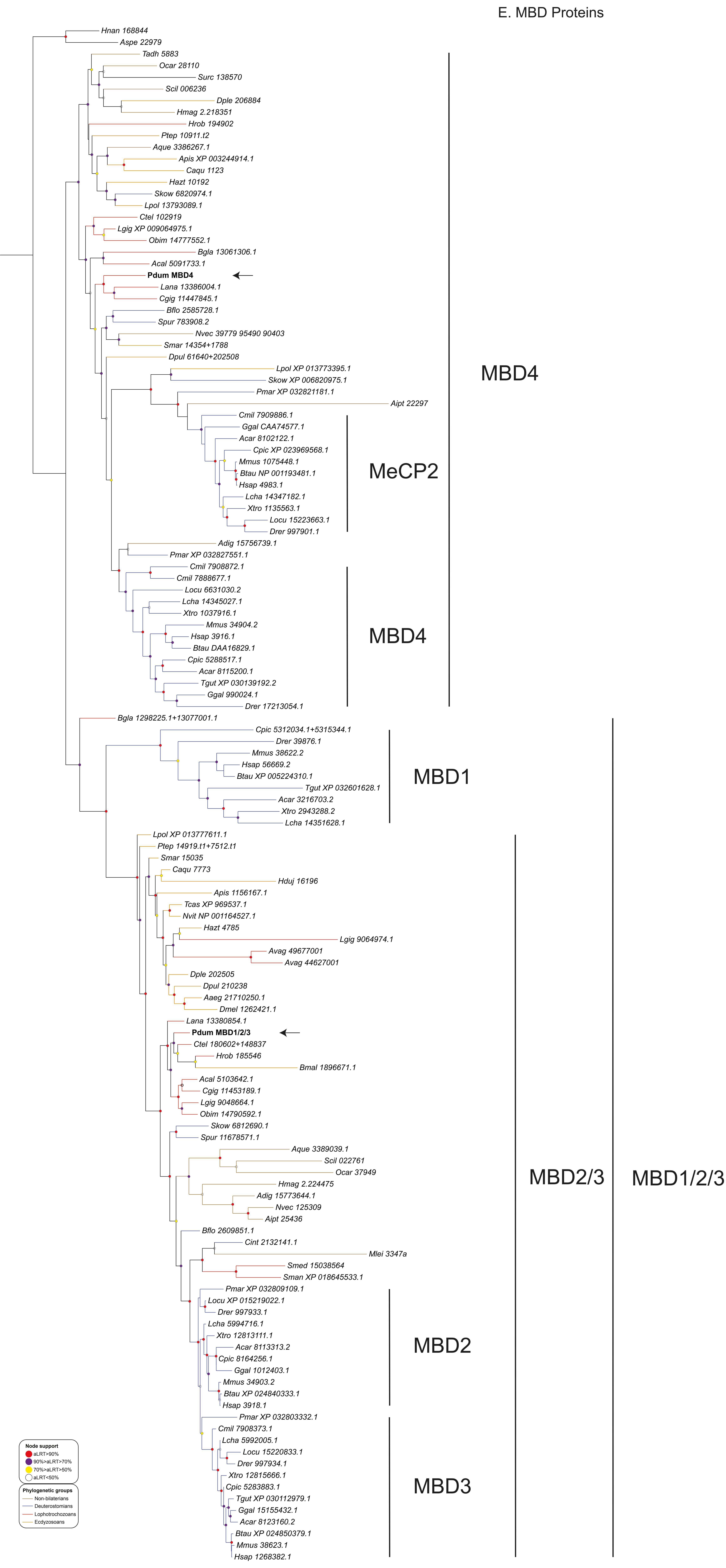
E. MBD Proteins
Hnan 168844
Aspe 22979
Tadh 5883
Ocar 28110
Surc 138570
Scil 006236
Dple 206884
Hmag 2.218351
Hrob 194902
Ptep 10911.t2
Aque 3386267.1
Apis XP 003244914.1
Caqu 1123
Hazt 10192
Skow 6820974.1
Lpol 13793089.1
Ctel 102919
Lgig XP 009064975.1
Obim 14777552.1
Bgla 13061306.1
Acal 5091733.1
Pdum MBD4
Lana 13386004.1
Cgig 11447845.1
Bflo 2585728.1
Spur 783908.2
Nvec 39779 95490 90403
Smar 14354+1788
Dpul 61640+202508
Lpol XP 013773395.1
Skow XP 006820975.1
Pmar XP 032821181.1
Aipt 22297
Cmil 7909886.1
Ggal CAA74577.1
Acar 8102122.1
Cpic XP 023969568.1
Mmus 1075448.1
Btau NP 001193481.1
Hsap 4983.1
Lcha 14347182.1
Xtro 1135563.1
Locu 15223663.1
Drer 997901.1
MeCP2
MBD4
Adig 15756739.1
Pmar XP 032827551.1
Cmil 7908872.1
Cmil 7888677.1
Locu 6631030.2
Lcha 14345027.1
Xtro 1037916.1
Mmus 34904.2
Hsap 3916.1
Btau DAA16829.1
Cpic 5288517.1
Acar 8115200.1
Tgut XP 030139192.2
Ggal 990024.1
Drer 17213054.1
MBD4
Bgla 1298225.1+13077001.1
Cpic 5312034.1+5315344.1
Drer 39876.1
Mmus 38622.2
Hsap 56669.2
Btau XP 005224310.1
Tgut XP 032601628.1
Acar 3216703.2
Xtro 2943288.2
Lcha 14351628.1
MBD1
Lpol XP 013777611.1
Ptep 14919.t1+7512.t1
Smar 15035
Caqu 7773
Hduj 16196
Apis 1156167.1
Tcas XP 969537.1
Nvit NP 001164527.1
Hazt 4785
Lgig 9064974.1
Avag 49677001
Avag 44627001
Dple 202505
Dpul 210238
Aaeg 21710250.1
Dmel 1262421.1
Lana 13380854.1
Pdum MBD1/2/3
Ctel 180602+148837
Hrob 185546
Bmal 1896671.1
Acal 5103642.1
Cgig 11453189.1
Lgig 9048664.1
Obim 14790592.1
Skow 6812690.1
Spur 11678571.1
Aque 3389039.1
Scil 022761
Ocar 37949
Hmag 2.224475
Adig 15773644.1
Nvec 125309
Aipt 25436
Bflo 2609851.1
Cint 2132141.1
Mlei 3347a
Smed 15038564
Sman XP 018645533.1
Pmar XP 032809109.1
Locu XP 015219022.1
Drer 997933.1
Lcha 5994716.1
Xtro 12813111.1
Acar 8113313.2
Cpic 8164256.1
Ggal 1012403.1
Mmus 34903.2
Btau XP 024840333.1
Hsap 3918.1
MBD2
Pmar XP 032803332.1
Cmil 7908373.1
Lcha 5992005.1
Locu 15220833.1
Drer 997934.1
Xtro 12815666.1
Cpic 5283883.1
Tgut XP 030112979.1
Ggal 15155432.1
Acar 8123160.2
Btau XP 024850379.1
Mmus 38623.1
Hsap 1268382.1
MBD3
MBD2/3
MBD1/2/3
Node support
aLRT>90%
90%>aLRT>70%
70%>aLRT>50%
aLRT<50%
Phylogenetic groups
Non-bilaterians
Deuterostomians
Lophotrochozoans
Ecdyzosoans

F. CHD Proteins

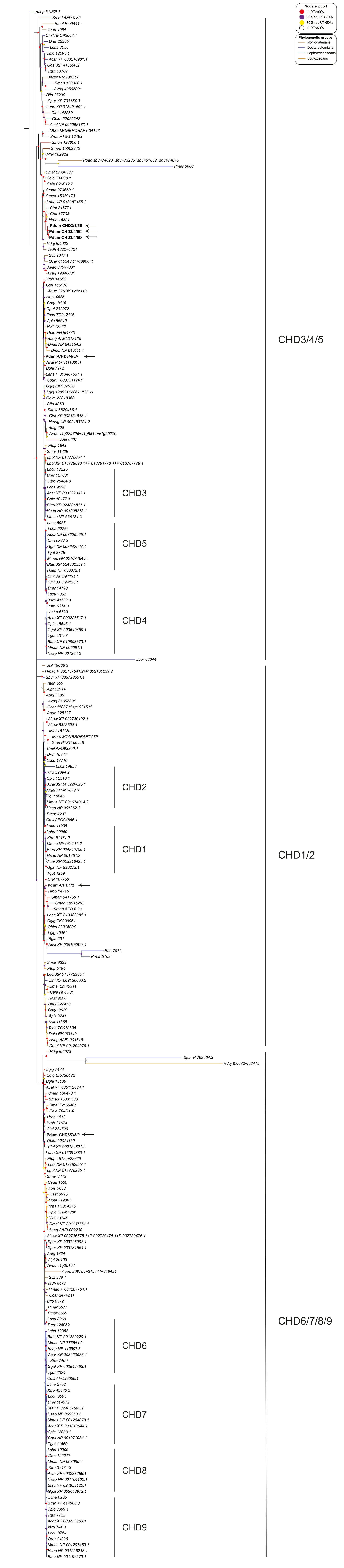

G. HDAC Proteins

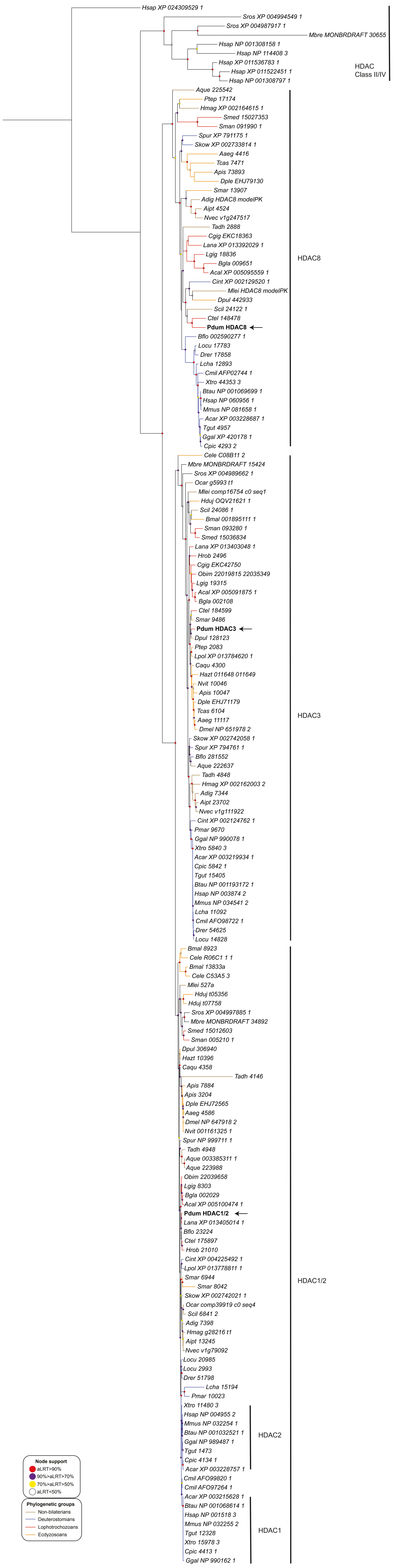

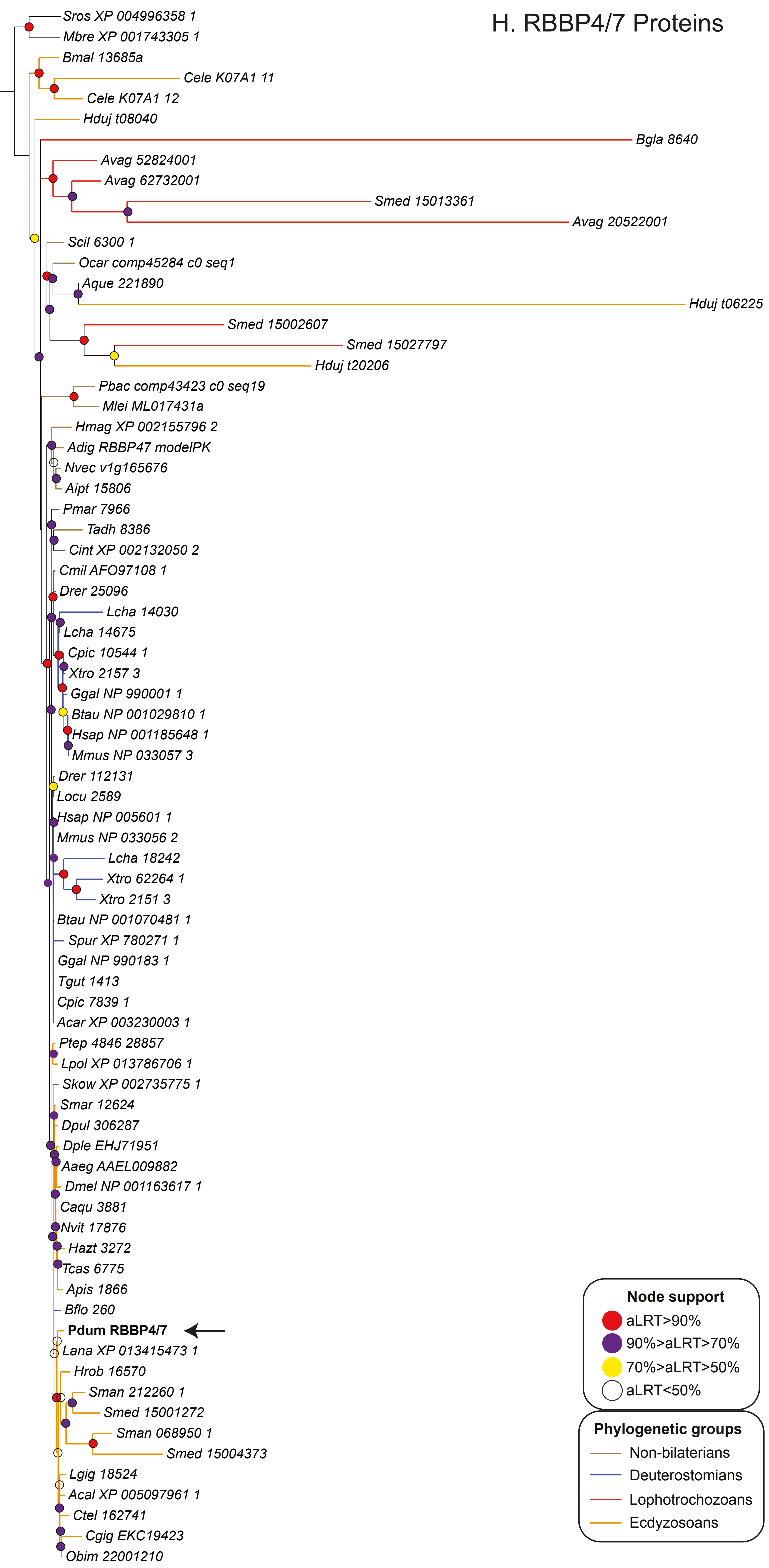
H. RBBP4/7 Proteins
Sros XP 004996358 1
Mbre XP 001743305 1
Bmal 13685a
Cele K07A1 11
Cele K07A1 12
Hduj t08040
Bgla 8640
Avag 52824001
Avag 62732001
Smed 15013361
Avag 20522001
Scil 6300 1
Ocar comp45284 c0 seq1
Aque 221890
Hduj t06225
Smed 15002607
Smed 15027797
Hduj t20206
Pbac comp43423 c0 seq19
Mlei ML017431a
Hmag XP 002155796 2
Adig RBBP47 modelPK
Nvec v1g165676
Aipt 15806
Pmar 7966
Tadh 8386
Cint XP 002132050 2
Cmil AFO97108 1
Drer 25096
Lcha 14030
Lcha 14675
Cpic 10544 1
Xtro 2157 3
Ggal NP 990001 1
Btau NP 001029810 1
Hsap NP 001185648 1
Mmus NP 033057 3
Drer 112131
Locu 2589
Hsap NP 005601 1
Mmus NP 033056 2
Lcha 18242
Xtro 62264 1
Xtro 2151 3
Btau NP 001070481 1
Spur XP 780271 1
Ggal NP 990183 1
Tgut 1413
Cpic 7839 1
Acar XP 003230003 1
Ptep 4846 28857
Lpol XP 013786706 1
Skow XP 002735775 1
Smar 12624
Dpul 306287
Dple EHJ71951
Aaeg AAEL009882
Dmel NP 001163617 1
Caqu 3881
Nvit 17876
Hazt 3272
Tcas 6775
Apis 1866
Bflo 260
Pdum RBBP4/7
Lana XP 013415473 1
Hrob 16570
Sman 212260 1
Smed 15001272
Sman 068950 1
Smed 15004373
Lgig 18524
Acal XP 005097961 1
Ctel 162741
Cgig EKC19423
Obim 22001210
Node support
aLRT>90%
90%>aLRT>70%
70%>aLRT>50%
aLRT<50%
Phylogenetic groups
Non-bilaterians
Deuterostomians
Lophotrochozoans
Ecdyzosoans

I. MTA1/2/3 Proteins

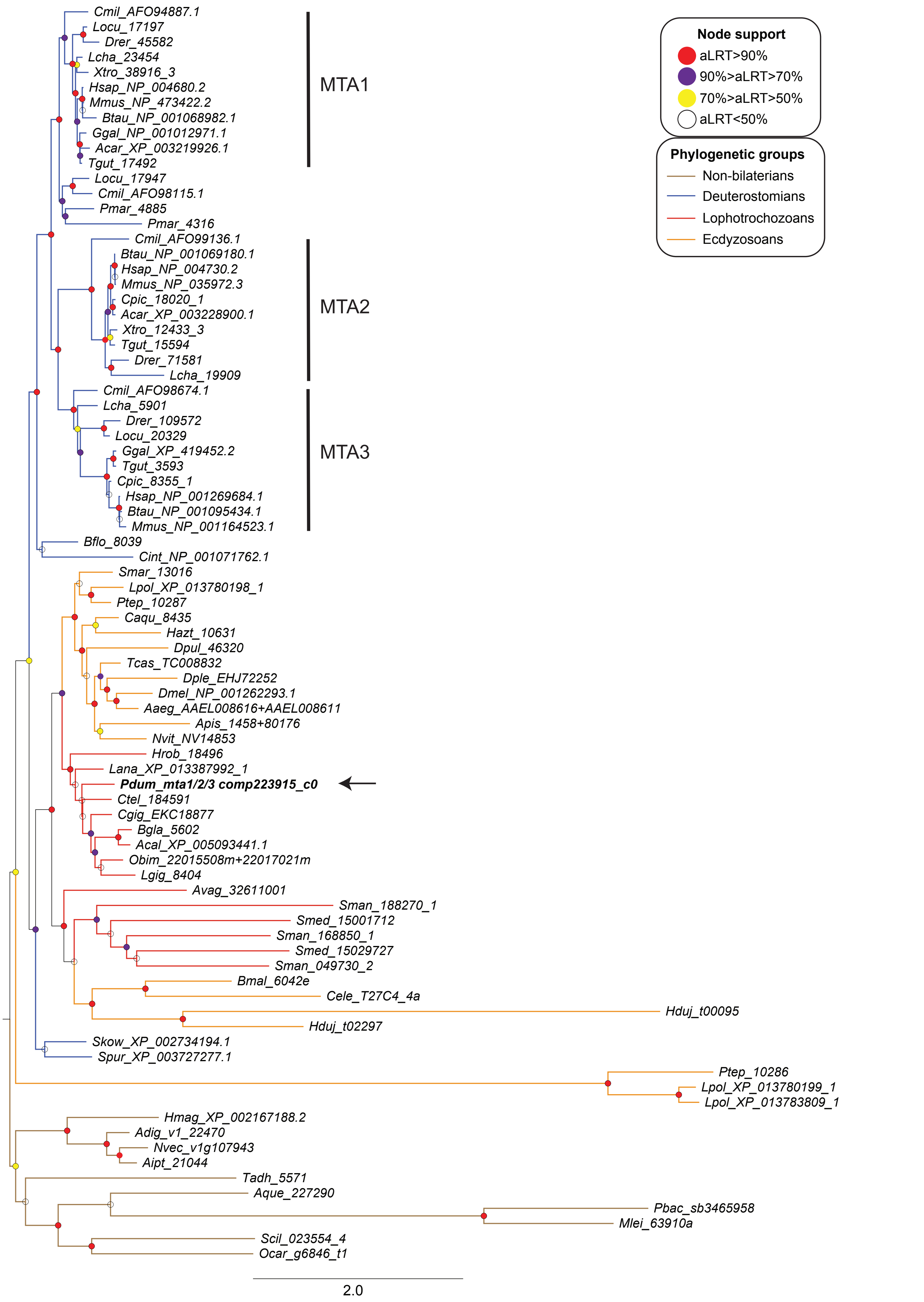

J. GATAD2 Proteins

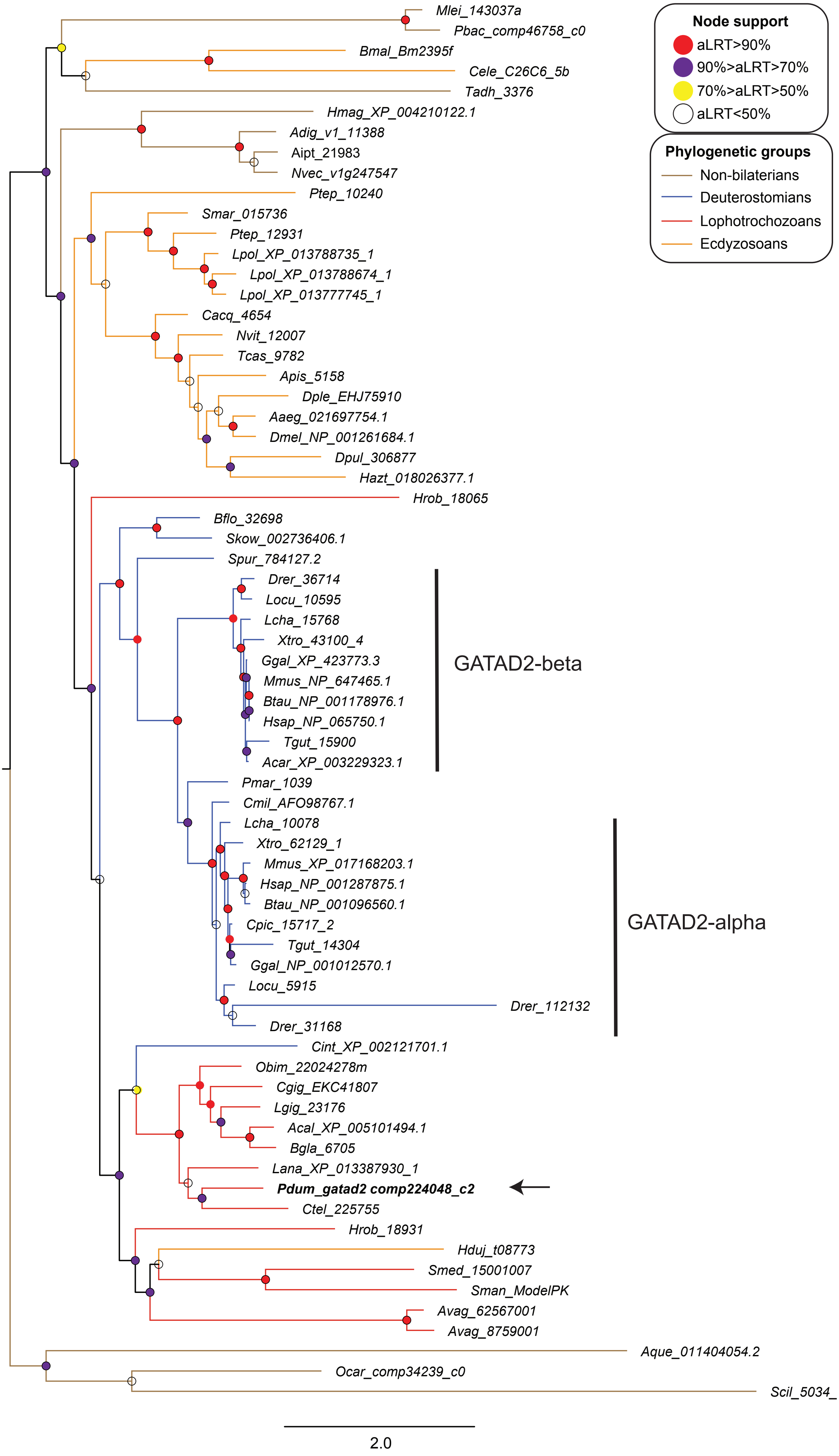

Supplement: Supplementary file 6 — Additional file 6: Figure S4. Phylogenetic trees of 5mC and NuRD toolkit proteins. Maximum likelihood (ML) trees constructed with PhyML are shown. Statistical supports (aLRT values) for all nodes are indicated with a color code provided in the inset. Terminal branches are colored using the shown color code also used in Fig. 2. P. dumerilii sequences are in bold and indicated by arrows. (A) DNMT proteins. The three subfamilies DNMT1, 2 and 3 are indicated. In the DNMT3 subfamily, the vertebrate-specific groups DNMT3A, B and -like are also shown. We used a distantly related sequence (Dnmt5) from Acanthamoeba castellanii (Acas, an amoeba) as outgroup to root the phylogenetic tree. We also retrieved Dnmt sequences from two choanoflagellates species, Monosiga brevicollis (Mbre) and Salpingoeca rosetta (Sros), which all belong to the DNMT2 subfamily. (B) TET proteins. The three vertebrate-specific groups TET1, 2 and 3 are indicated. We used midpoint rooting for this tree as we were unable to find Tet genes in choanoflagellates or another suitable outgroup. (C) TDG. The phylogenetic tree is rooted using choanoflagellate sequences as outgroup. (D) UHRF. The two vertebrate-specific groups UHRF1 and 2 are indicated. We used midpoint rooting for this tree as we were unable to find Uhrf genes in choanoflagellates or another suitable outgroup. (E) MBD proteins. The two subfamilies MBD1/2/3 and MBD4 subfamilies are indicated. Vertebrate-specific groups are also shown. While not found in S. rosetta and M. brevicollis, a single Mbd gene was found in three other choanoflagellates (Helgoeca nana (Hnan), Salpingoeca urceolata (Surc), and Acanthoeca spectabilis (Aspe)) for which extensive transcriptomic data have been produced. Two of these choanoflagellate Mbd sequences (Hnan and Aspe) form a monophyletic group that was used as outgroup to root the tree, while the third one (Surc) clusters with MBD 4 proteins. (F) CHD proteins. The three subfamilies CHD1/2, CHD3/4/5 and CHD6/7/8/9 are [file 12915_2021_1074_MOESM6_ESM.pdf]
