# Supplementary figures and images for: DNA methylation atlas and machinery in the developing and regenerating annelid Platynereis dumerilii
Source: BMC Biol. 2021 Aug 3;19:148. doi: 10.1186/s12915-021-01074-5 (PMC8330077; doi:10.1186/s12915-021-01074-5)

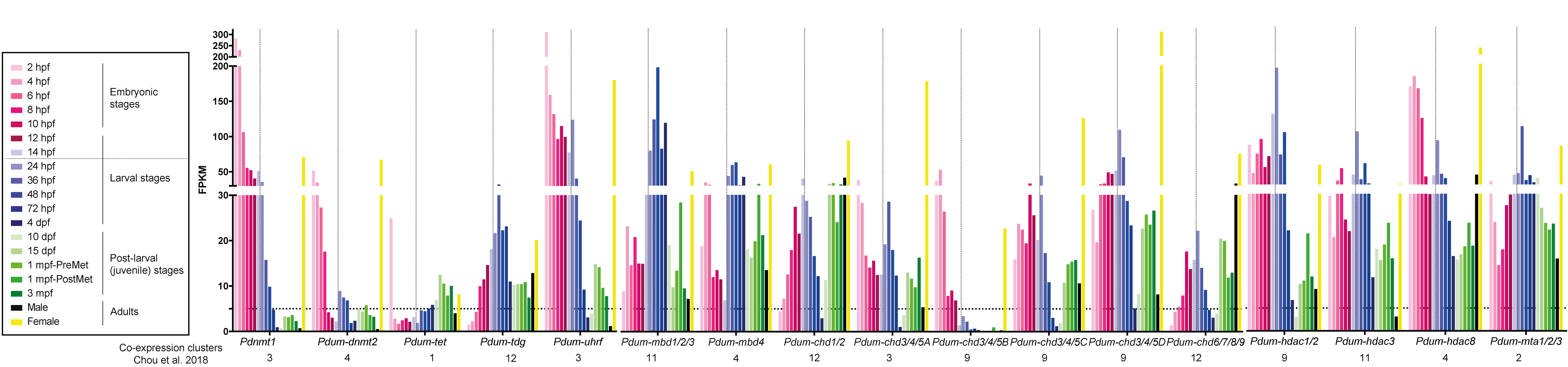

Supplement: Supplementary file 7 — Additional file 7: Figure S5. Expression level of 5mC and NuRD machinery genes during P. dumerilii development and along its life cycle. A histogram reporting FPKM values at 19 stages (developmental and adult stages) for all indicated genes is shown. The FPKM values from embryonic stages and those for the other stages cannot be compared, as having been calculated from two independent RNA-seq studies. These two datasets are therefore separated by a black vertical dashed line. Black horizontal dotted lines highlight a 5 FPKM threshold about which a gene can be considered as significantly expressed, as its expression can usually be detected by in situ hybridization [49]. Co-expression clusters are those defined by Chou et al. [49]. Hpf: hours post-fertilization; dpf: days post-fertilization; mpf: months post-fertilization. [file 12915_2021_1074_MOESM7_ESM.tif]

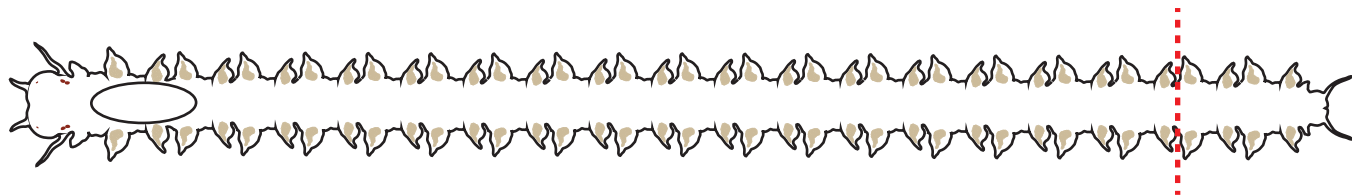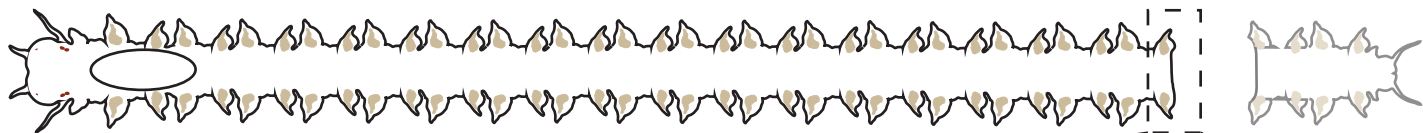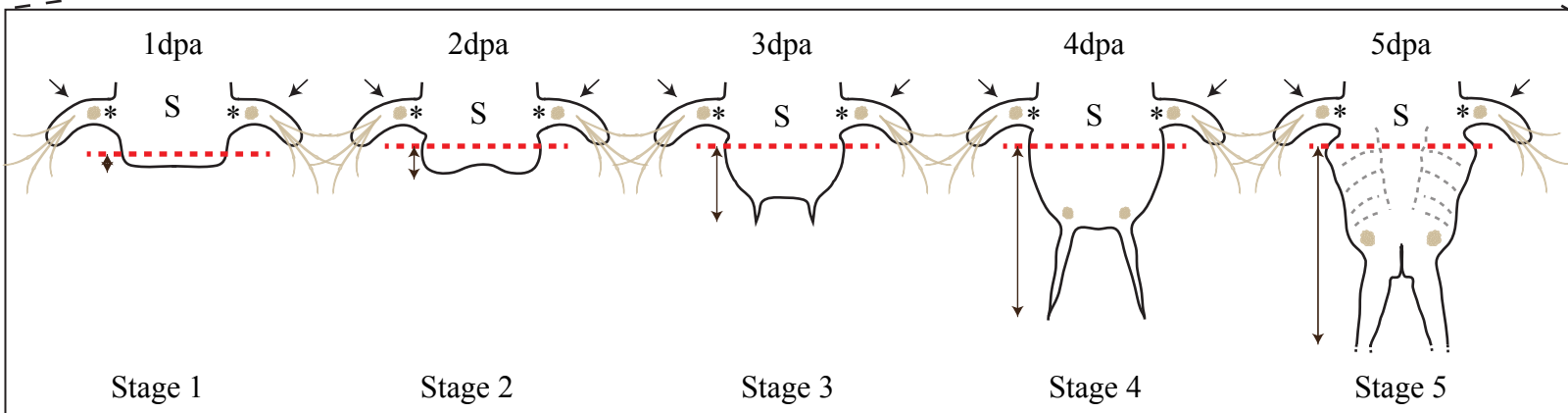

Supplement: Supplementary file 8 — Additional file 8: Figure S6. A schematic representation of P. dumerilii posterior regeneration. On the top row is depicted a 3-4-month-old juvenile worm (with 30 to 40 segments). Its posterior part (5 segments, growth zone, and pygidum) is eliminated by amputation (the red dotted lines indicate the amputation plane). Regeneration occurs at the posterior extremity of the anterior body region. The region delineated by the dotted black lines corresponds to the part of the regenerating worms that is shown in WMISH pictures (Figs. 5 and S7). It comprises the posterior part of the posteriormost differentiated segment (S), in which parapodia (black arrows) associated with glands (asterisks) can be seen, plus the regenerated region (region indicated by the two-headed arrow; red dotted lines show the position of the amputation plane). Anterior is up and posterior down. The regenerated region increases in size as regeneration proceeds, from stage 1 to 5 of the process [37]. At stage 1 (reached 1 day post-amputation, 1dpa), wound healing is achieved. A small blastema is formed at stage 2 (2dpa) and molecular analyses indicate that the growth zone is already regenerated at this stage. A regenerated anus can be observed at this stage. At stage 3 (3dpa), the regenerated region has increased in size and small anal cirri are present. The use of molecular markers showed that the growth zone has already produced at least one segment and tissue differentiation has started in the pygidium. The large blastema found at stage 4 (4dpa) contains a differentiated pygidium with long anal cirri. Tissue differentiation also starts in the two or more segments that have been produced by the growth zone. Segments are however still not morphologically visible. Stage 5 (5dpa) corresponds to the end of regeneration. At this stage, a fully differentiated pygidium is found, and segmentation of the regenerated region becomes visible at the morphological level (presence of visible parapodial primordia f [file 12915_2021_1074_MOESM8_ESM.pdf]

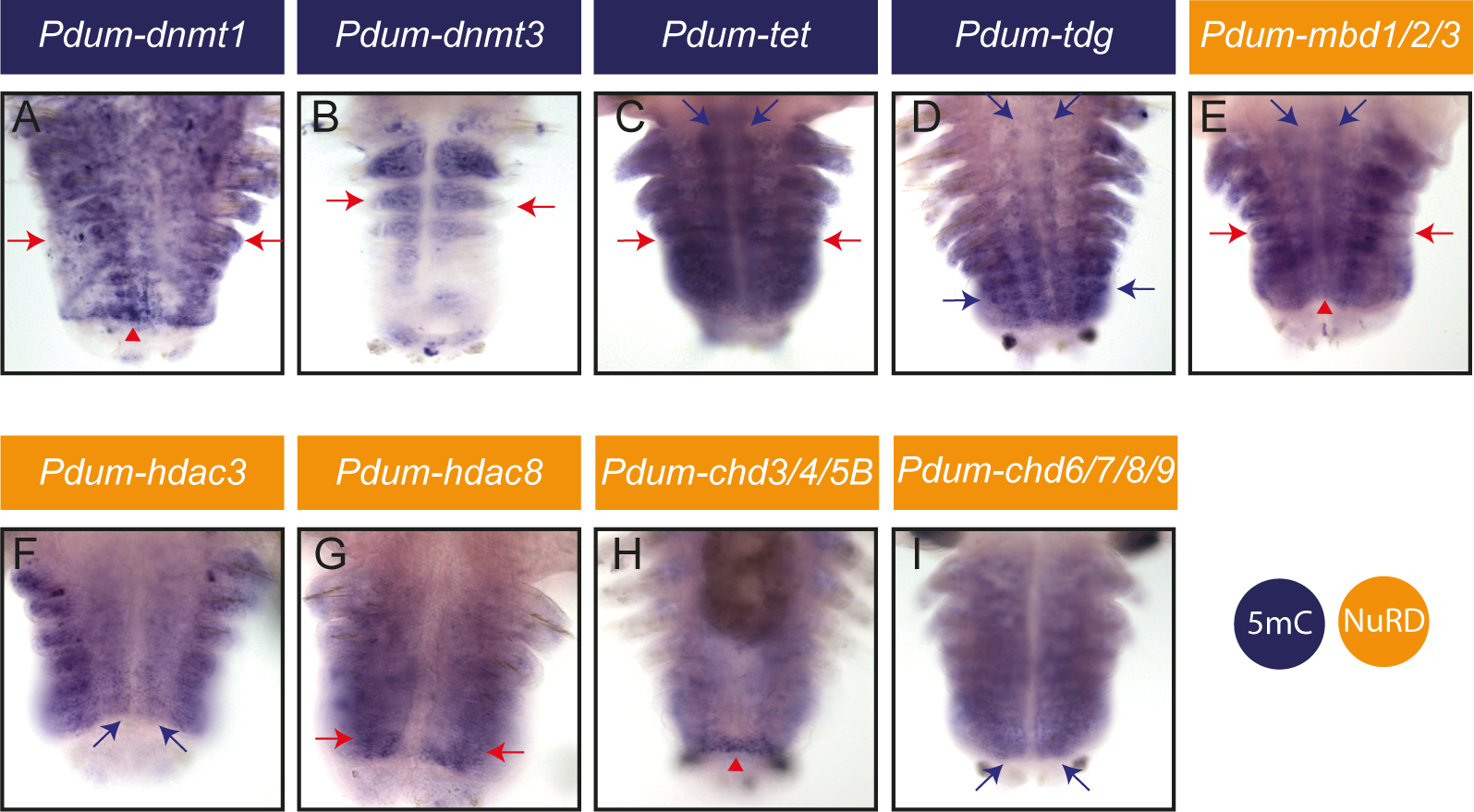

Supplement: Supplementary file 11 — Additional file 11: Figure S9. Expression of 5mC and NuRD genes in worms at 15 days post-amputation. Whole-mount in situ hybridizations (WMISH) for the genes whose name is indicated are shown at 15 days post-amputation (15dpa). These worms are used as a proxy for non-amputated worms [36]. In all panels, anterior is up. All images are ventral views. Dark blue arrows point to an ectodermal expression including an expression in the ventral nerve cord, red arrows point to mesodermal cells of the developing segments, and red arrowheads to the mesodermal part of the growth zone. We failed to detect significant expression for Pdum-chd1/2. [file 12915_2021_1074_MOESM11_ESM.tif]

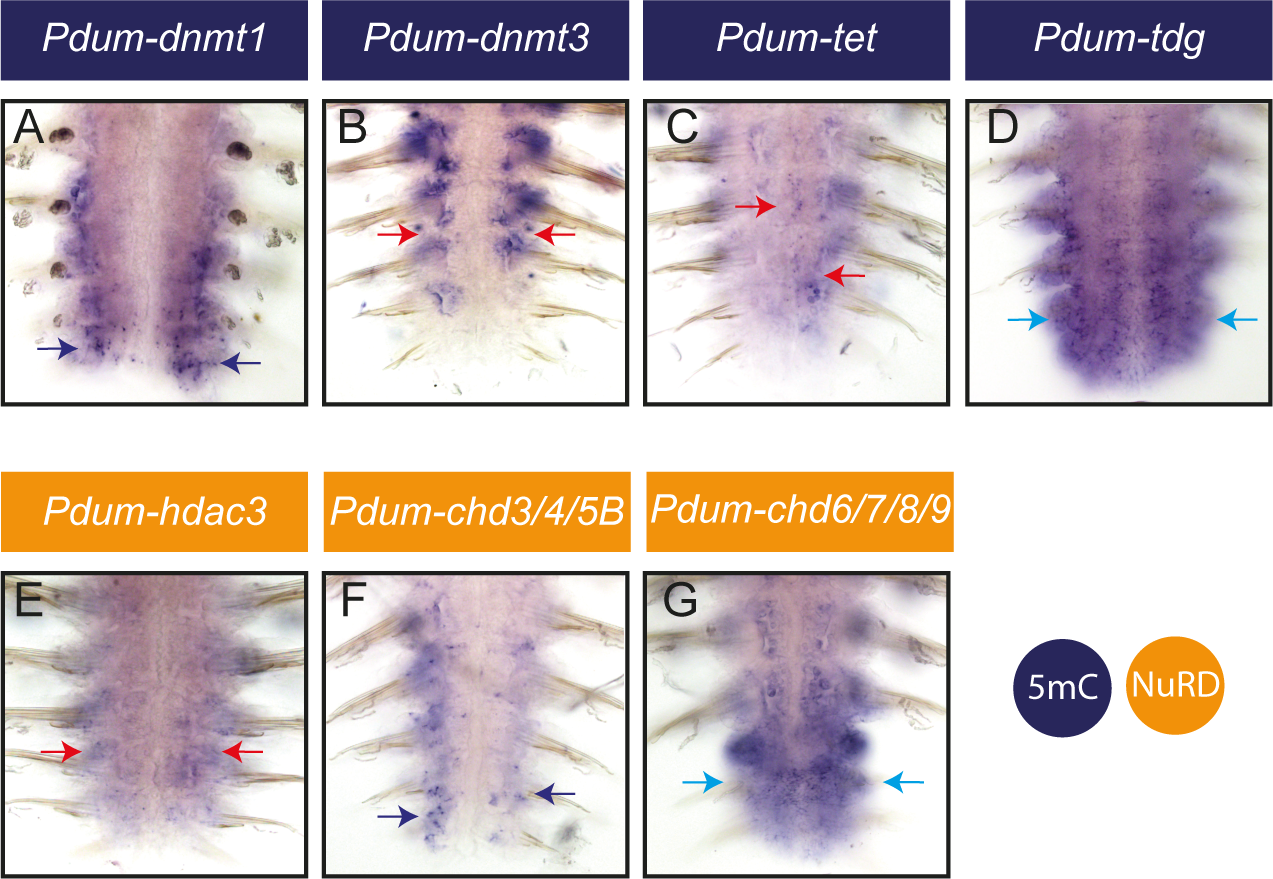

Supplement: Supplementary file 12 — Additional file 12: Figure S10. Expression of 5mC and NuRD genes in worms immediately after amputation. Whole-mount in situ hybridizations (WMISH) for the genes whose name is indicated are shown. In all panels, anterior is up. Only weak and diffuse expression was found for the genes that are shown and we failed to detect significant expression for the three other genes (Pdum-mbd1/2/3, Pdum-hdac8 and Pdum-chd1/2). Pdum-dnmt1 and Pduchd3/4/5B are expressed in a few ectodermal cells (dark blue arrows); Pdum-dnmt3, Pdum-tet and Pdum-hdac3 are expressed in mesodermal cells (red arrows); and Pdum-tdg and Pdum-chd6/7/8/9 are largely expressed in the ectoderm including the ventral nerve cord (light blue arrows). [file 12915_2021_1074_MOESM12_ESM.tif]

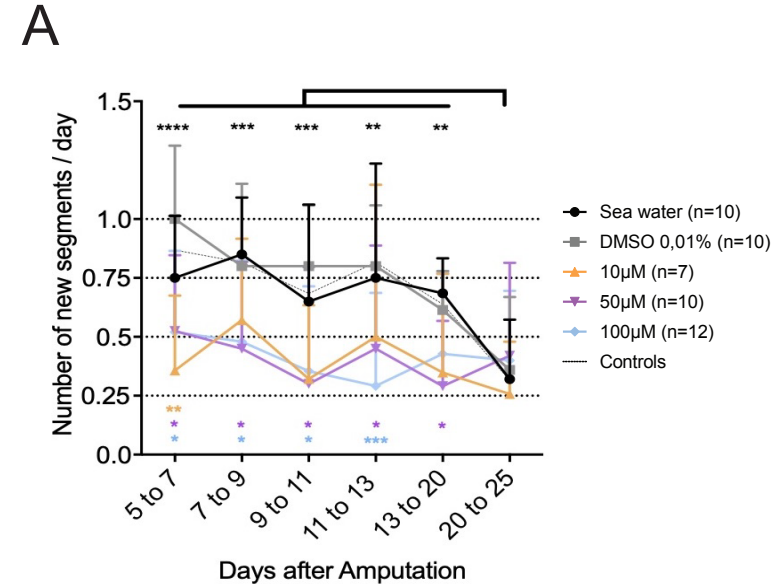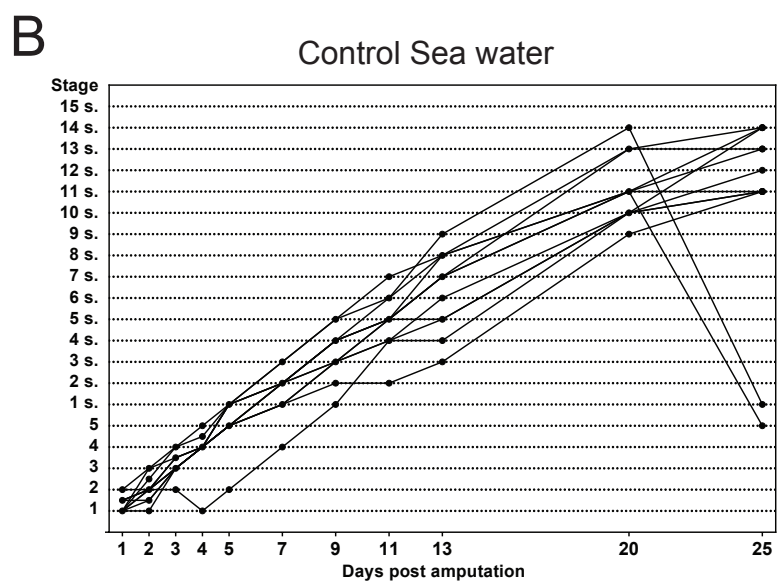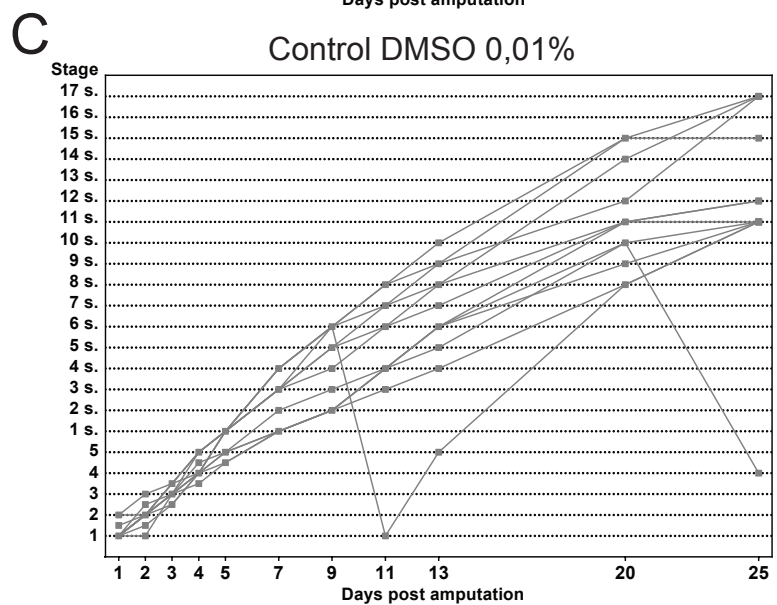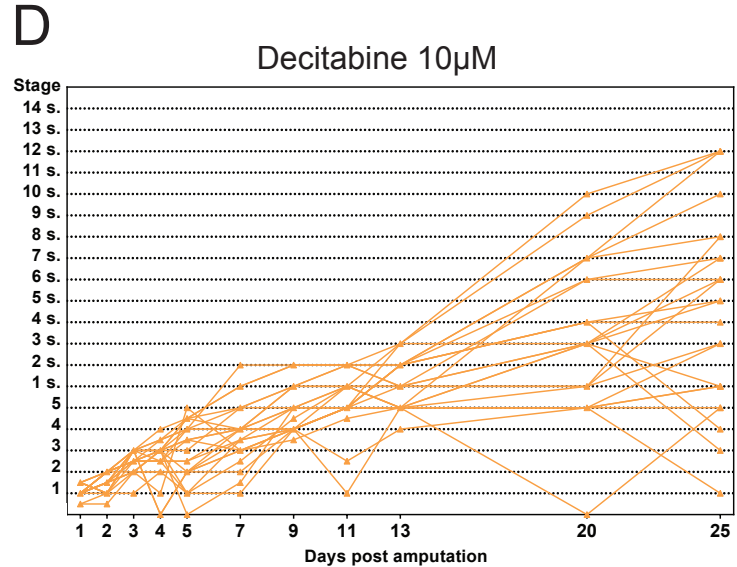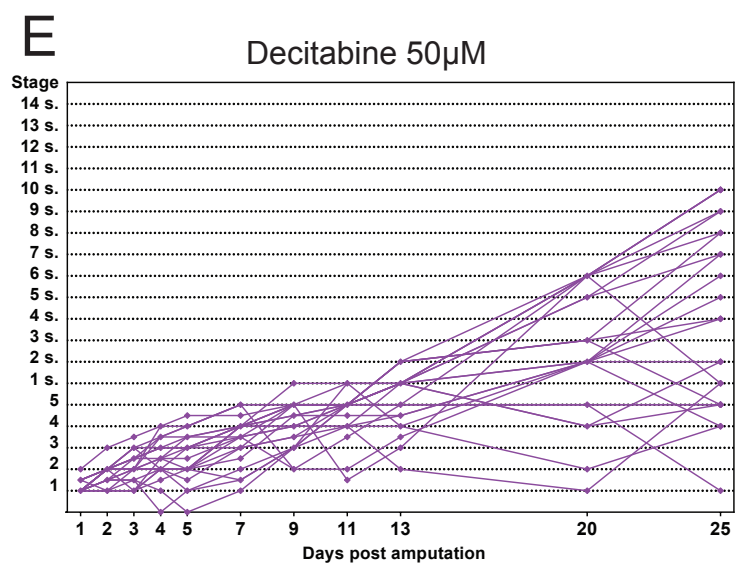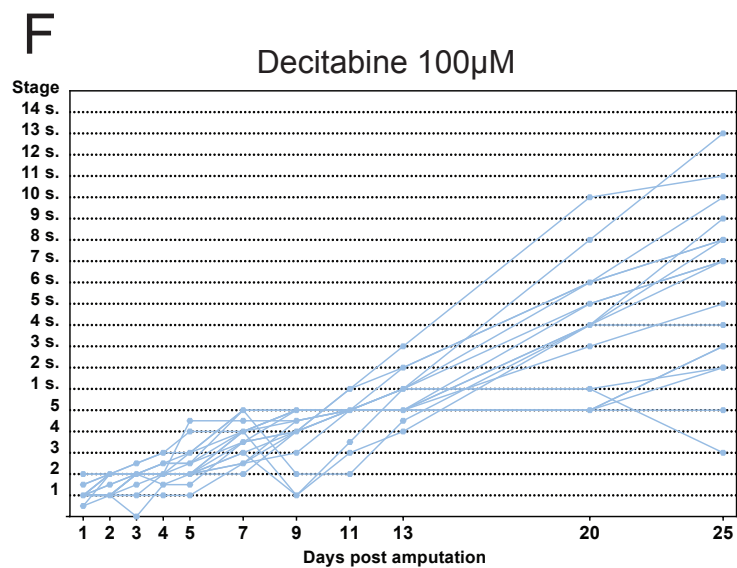

Supplement: Supplementary file 13 — Additional file 13: Figure S11. Rate of segment addition and individual scoring of Decitabine-treated worms. (A) Graphic representation of the rate of segment addition in controls (DMSO 0.01% and sea water) and Decitabine-treated worms (worms that showed autotomy were excluded). (B-F) Graphic representation of scoring of individual control (B and C) and Decitabine-treated (D-F) worms until 25dpa. Two experiments, mean ± SD. For the analysis of the rate of segment addition, 1-way ANOVA was performed with Dunnett post hoc test (**: p < 0.01; ***: p < 0.001; ****: p < 0.0001). For conditions comparison, 2-way ANOVA was performed (Source of variation: Time p < 0.0001, Treatment p < 0.0001, Interaction p = 0.0875) with Dunnett post hoc test (*: p < 0.05; **: p < 0.01; ***: p < 0.001). The number of worms used for these experiments is indicated in the figure. [file 12915_2021_1074_MOESM13_ESM.pdf]

A

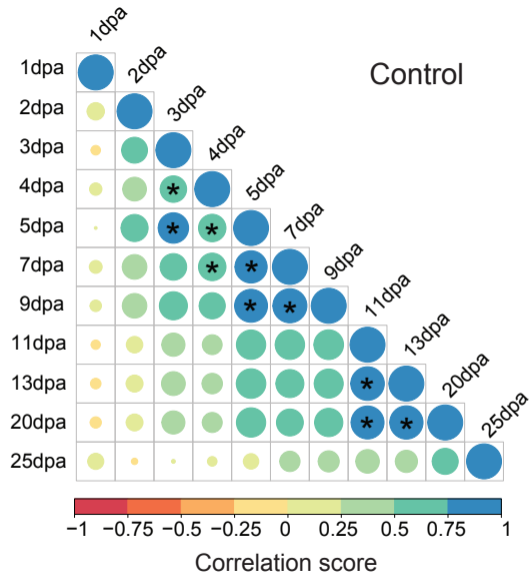

# B

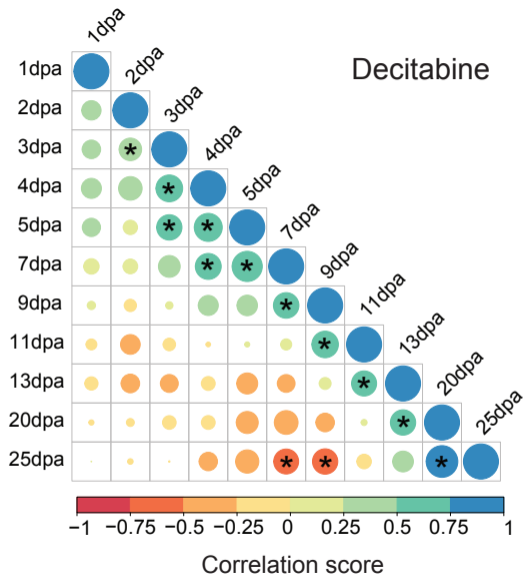

Supplement: Supplementary file 15 — Additional file 15: Figure S13. Multiple correlation analysis between regeneration and segment addition. (A-B) Statistical analysis of the regeneration score correlation in (A) control and (B) Decitabine-treated worms. Blue dots show positive correlations and red dots negative correlations. The dot size is proportional to the correlation score and significant correlations are highlighted with asterisks. Spearman correlation with Holm post hoc test (* for p < 0.05). [file 12915_2021_1074_MOESM15_ESM.pdf]

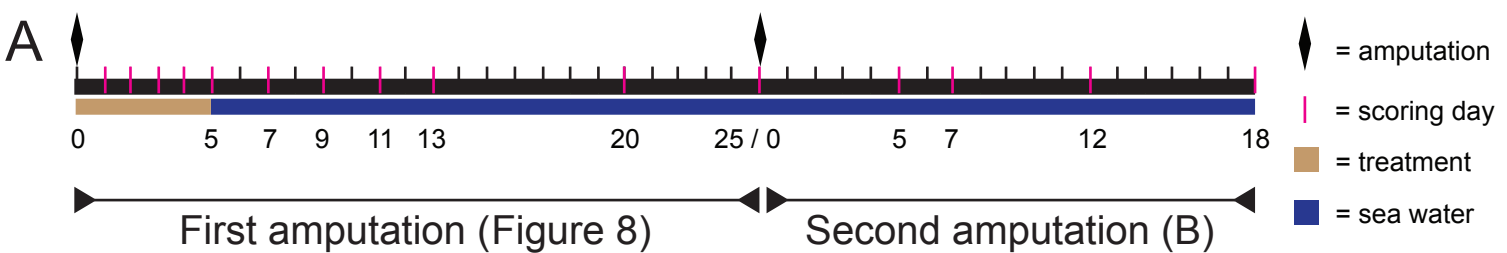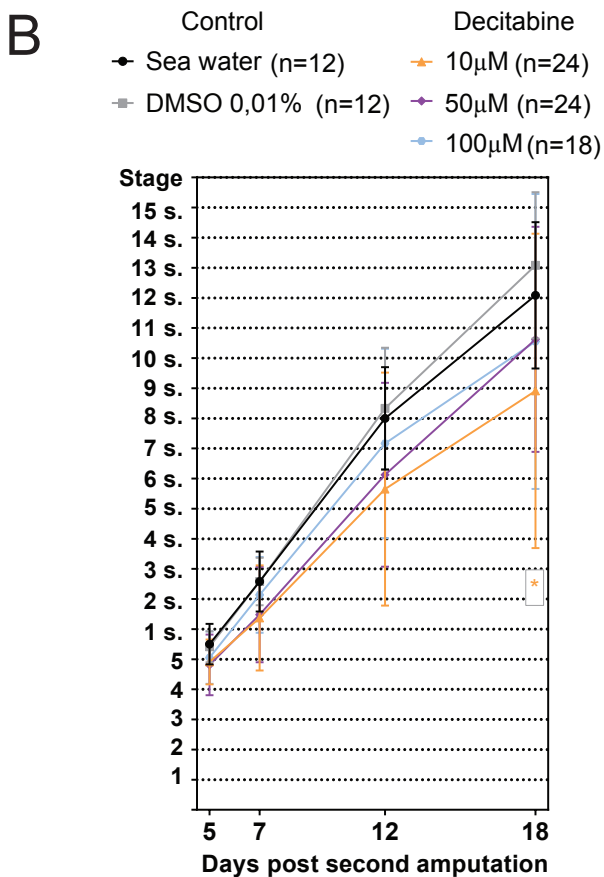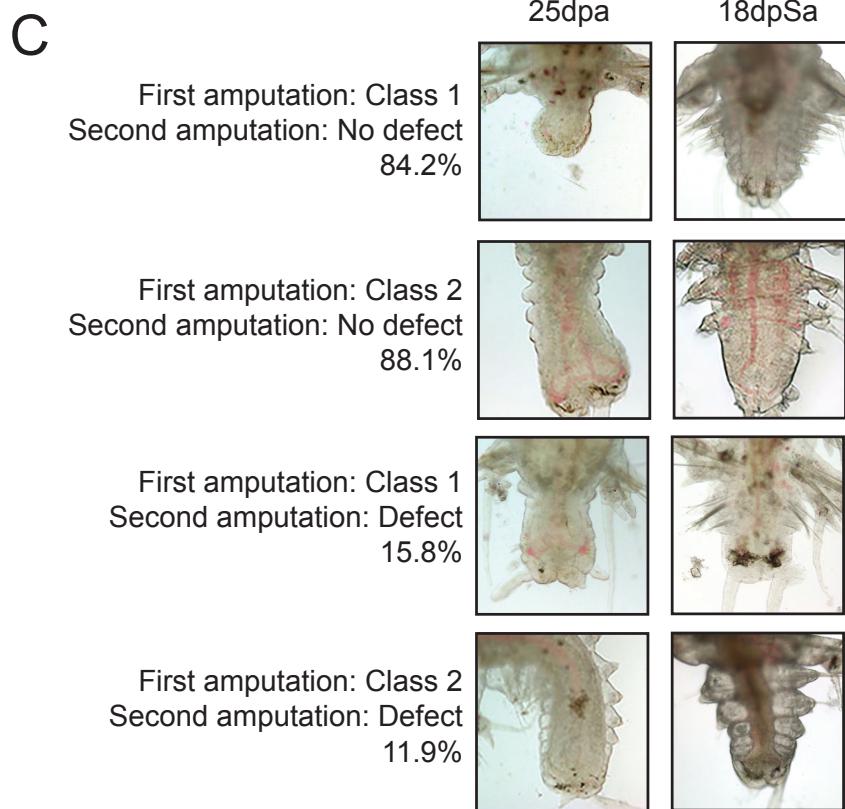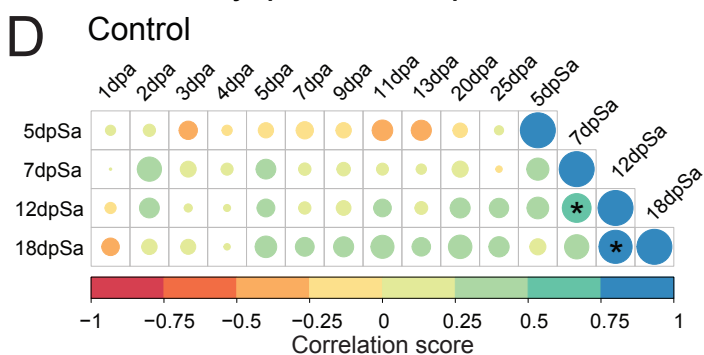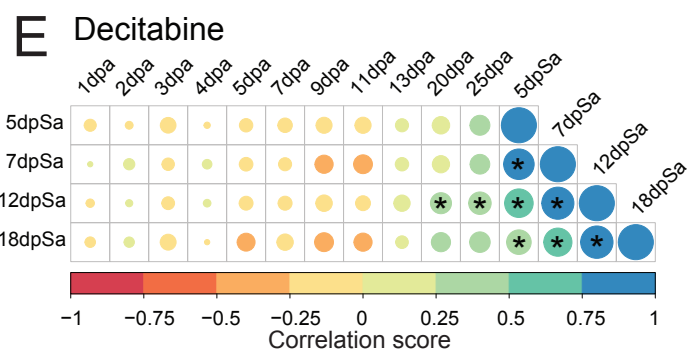

Supplement: Supplementary file 16 — Additional file 16: Figure S14. Analysis of long-term Decitabine effects on regenerating and growing worms after a second amputation. (A) Schematic representation of the experimental design. Worms were treated with Decitabine (10 μM, 50 μM, or 100 μM), or DMSO (0,01 %; control) or kept in normal sea water (control) for five days following amputation (5 days post-amputation, dpa). Decitabine was washed out and worms were kept in normal sea water until 25dpa (see Fig. 8). A second amputation was performed (which removed the regenerated region) and worms were kept in normal sea water until 18 days post-second amputation (18dpSa). Observations were done at indicated time points (pink bars). (B) Graphic representation of the stages reached by control worms (normal sea water and DMSO 0,01%) and Decitabine-treated worms after the second amputation. A significant delay was observed for worms treated with 10 μM as compared to controls at 18dpSa. Worms treated with the other concentrations of Decitabine regenerated and added segments similarly to controls. Two experiments, mean ± SD, 2-way. ANOVA (p value: Time p < 0.0001, Treatment p = 0.0283, Interaction p = 0.0663) with Tukey post hoc test (*: p < 0.05). Only p values corresponding to the comparison to normal sea water are shown (similar values were obtained for the comparison to DMSO controls). The number of worms used for these experiments is indicated in the figure. (C) Most Decitabine worms that were class 1 or class 2 at 25dpa (after first amputation) regenerated after a second amputation without any morphological abnormalities (84,2% and 88,1%, respectively), but some of them show minor morphological defects in parapodia and chaetae formation (15,8% and 11,9%, respectively). (D and E) Multiple correlation analysis between regeneration/segment addition after first and second amputation. Only positive correlations were observed for both control and Decitabine-treated worms after a second amputation. Blue dots indicate [file 12915_2021_1074_MOESM16_ESM.pdf]
