# Supplementary material for: DNA methylation atlas and machinery in the developing and regenerating annelid Platynereis dumerilii
Source: BMC Biol. 2021 Aug 3;19:148. doi: 10.1186/s12915-021-01074-5 (PMC8330077; doi:10.1186/s12915-021-01074-5)
Supplement: Supplementary file 9 — Additional file 9: Figure S7. Additional expression data of 5mC and NuRD genes during regeneration. Whole-mount in situ hybridizations (WMISH) for the genes whose name is indicated are shown. In all panels except E, anterior is up and the regeneration stage for each picture is indicated. In all panels except E, red dotted lines indicate the amputation plane. Dorsal (D) and posterior (P) views are shown. Light blue arrowheads = ectodermal growth zone, light blue arrows = ectoderm of developing segment, red arrows = groups of internal cells in the segment adjacent to the amputation plane. [file 12915_2021_1074_MOESM9_ESM.pdf]

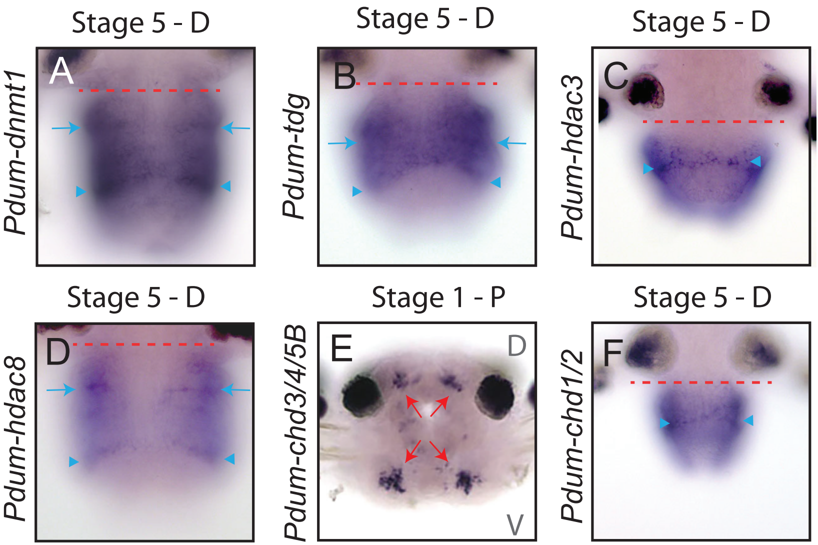
Stage 5 - D
A
Pdum-dnmt1
Stage 5 - D
B
Pdum-tdg
Stage 5 - D
C
Pdum-hdac3
Stage 5 - D
D
Pdum-hdac8
Stage 1 - P
E
D
V
Pdum-chd3/4/5B
Stage 5 - D
F
Pdum-chd1/2
