# Supplementary material for: DNA methylation atlas and machinery in the developing and regenerating annelid Platynereis dumerilii
Source: BMC Biol. 2021 Aug 3;19:148. doi: 10.1186/s12915-021-01074-5 (PMC8330077; doi:10.1186/s12915-021-01074-5)
Supplement: Supplementary file 10 — Additional file 10: Figure S8. Schematic representation of expression patterns of 5mC and NuRD genes during regeneration. In all panels, anterior is up and the regeneration stage for each drawing is indicated. Ventral schematic representations are shown for all stages and a dorsal schematic representation is also provided for stage 5. The color code for the different tissues is provided in the inset. [file 12915_2021_1074_MOESM10_ESM.pdf]

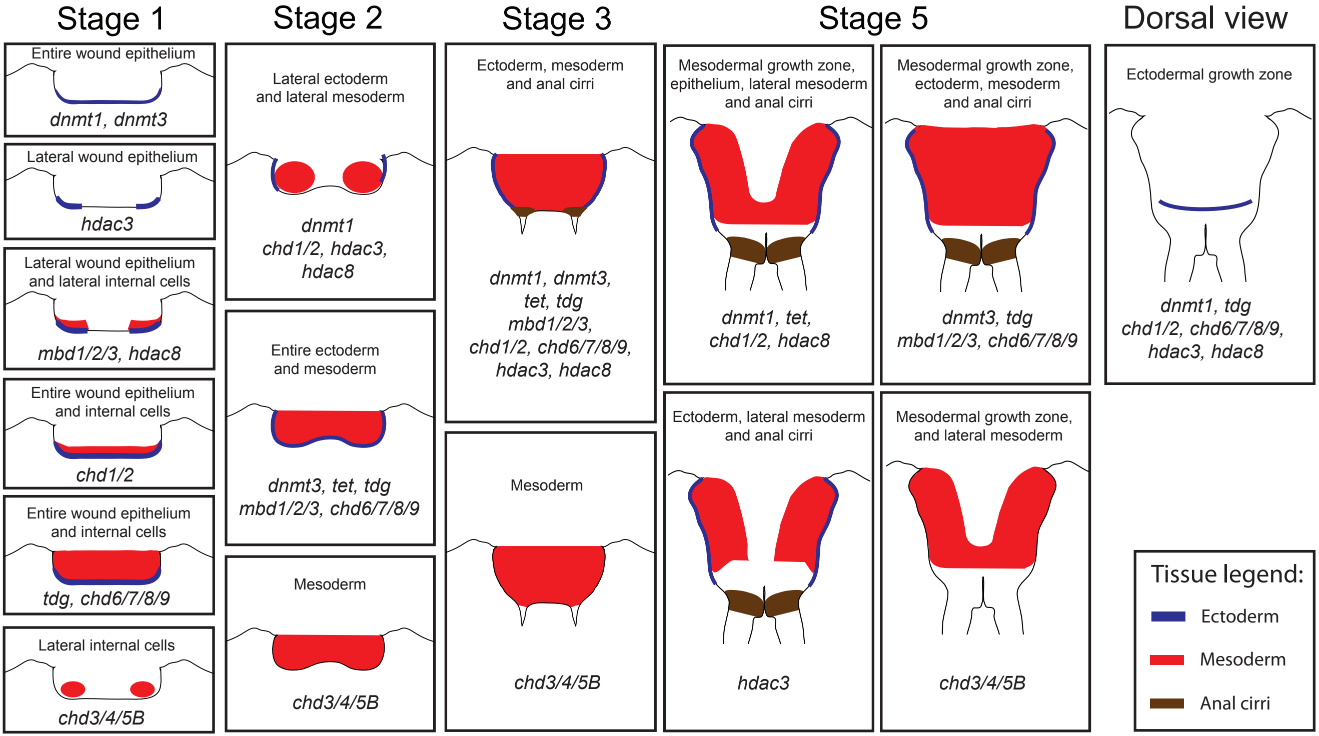
Stage 1
Stage 2
Stage 3
Stage 5
Dorsal view
Entire wound epithelium
dnmt1, dnmt3
Lateral wound epithelium
hdac3
Lateral wound epithelium and lateral internal cells
mbd1/2/3, hdac8
Entire wound epithelium and internal cells
chd1/2
Entire wound epithelium and internal cells
tdg, chd6/7/8/9
Lateral internal cells
chd3/4/5B
Lateral ectoderm and lateral mesoderm
dnmt1 chd1/2, hdac3, hdac8
Entire ectoderm and mesoderm
dnmt3, tet, tdg mbd1/2/3, chd6/7/8/9
Mesoderm
chd3/4/5B
Ectoderm, mesoderm and anal cirri
dnmt1, dnmt3, tet, tdg mbd1/2/3, chd1/2, chd6/7/8/9, hdac3, hdac8
Mesoderm
chd3/4/5B
Mesodermal growth zone, epithelium, lateral mesoderm and anal cirri
dnmt1, tet, chd1/2, hdac8
Mesodermal growth zone, ectoderm, mesoderm and anal cirri
dnmt3, tdg mbd1/2/3, chd6/7/8/9
Ectoderm, lateral mesoderm and anal cirri
hdac3
Mesodermal growth zone, and lateral mesoderm
chd3/4/5B
Ectodermal growth zone
dnmt1, tdg chd1/2, chd6/7/8/9, hdac3, hdac8
Tissue legend:
Ectoderm
Mesoderm
Anal cirri
