# Supplementary material for: DNA methylation atlas and machinery in the developing and regenerating annelid Platynereis dumerilii
Source: BMC Biol. 2021 Aug 3;19:148. doi: 10.1186/s12915-021-01074-5 (PMC8330077; doi:10.1186/s12915-021-01074-5)
Supplement: Supplementary file 14 — Additional file 14: Figure S12. Classification of Decitabine-treated worms based on morphological defects. Three classes of worms can be defined based on the reported morphological defects. For each class, representative worms at five different time points after amputation are shown. Green arrowheads = characteristic constriction between the non-regenerated and regenerated regions, pink arrows = very reduced or absent anal cirri, blue arrows = reduced/abnormal anal cirri, green arrows = narrowed regenerated region, black arrowheads = abnormal parapodia. [file 12915_2021_1074_MOESM14_ESM.pdf]

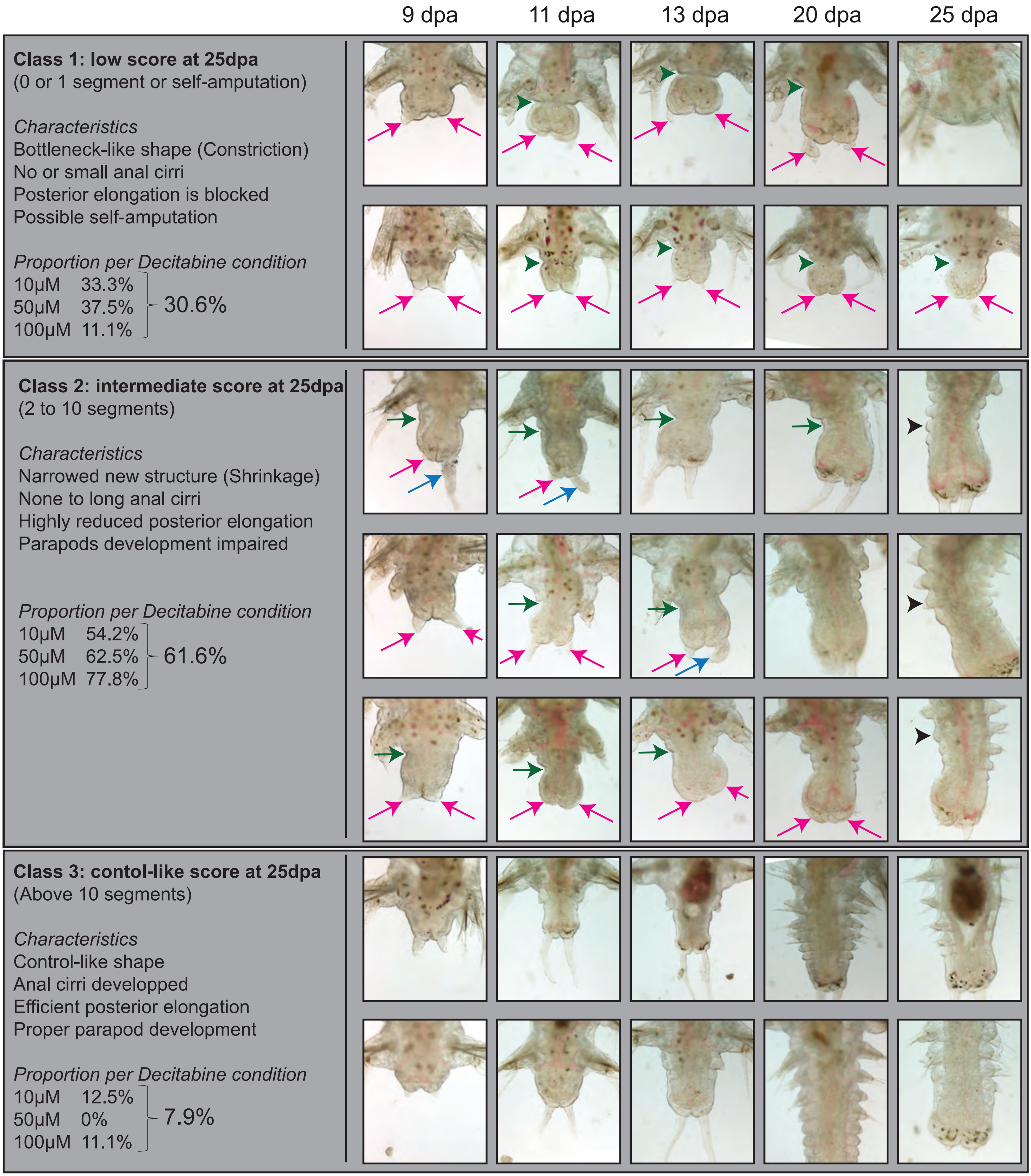

9 dpa
11 dpa
13 dpa
20 dpa
25 dpa
Class 1: low score at 25dpa
(0 or 1 segment or self-amputation)
Characteristics
Bottleneck-like shape (Constriction)
No or small anal cirri
Posterior elongation is blocked
Possible self-amputation
Proportion per Decitabine condition
10µM 33.3%
50µM 37.5%
100µM 11.1%
30.6%
Class 2: intermediate score at 25dpa
(2 to 10 segments)
Characteristics
Narrowed new structure (Shrinkage)
None to long anal cirri
Highly reduced posterior elongation
Parapods development impaired
Proportion per Decitabine condition
10µM 54.2%
50µM 62.5%
100µM 77.8%
61.6%
Class 3: contol-like score at 25dpa
(Above 10 segments)
Characteristics
Control-like shape
Anal cirri developped
Efficient posterior elongation
Proper parapod development
Proportion per Decitabine condition
10µM 12.5%
50µM 0%
100µM 11.1%
7.9%
